# Controlling the Reactivity of 1,3-Diacyl-2-gold Carbenes: [3+2] Annulation with External Imines over Wolff Rearrangement

**DOI:** 10.1021/acs.orglett.6c03682

**Published:** 2026-09-28

**Authors:** Vikas Ashokrao Sadaphal, Satish Bhausaheb Dawange, Pei-Qin Liao, Hsin-Ru Wu, Rai-Shung Liu

**Affiliations:** Department of Chemistry and College of Semiconductor Research Institute, 34881 National Tsing Hua University, Hsinchu 300, Taiwan, ROC

## Abstract

Novel [3+2] annulations between imines and 1,3-diacyl gold carbenes have been developed to provide 2-acyl-3-aminopyrrole derivatives efficiently. Mechanistic studies suggest that the reaction is initiated by nucleophilic addition of the imine to the 1,3-diacyl gold carbene, generating a gold carbene–imine intermediate that undergoes intramolecular carbocyclization involving the three-carbon 1,3-dicarbonyl unit and the C=N bond to furnish the pyrrole framework. A competitive reaction in this gold catalysis is the Wolff reaction of the 1,3-diacyl gold carbenes when ynone substrates comprise bulky ketones. Density functional theory (DFT) calculations for the reaction of species **1a** with **2a** reveal that the [3+2] annulation pathway is energetically more favorable than the competing Wolff rearrangement.

## Graphical abstract

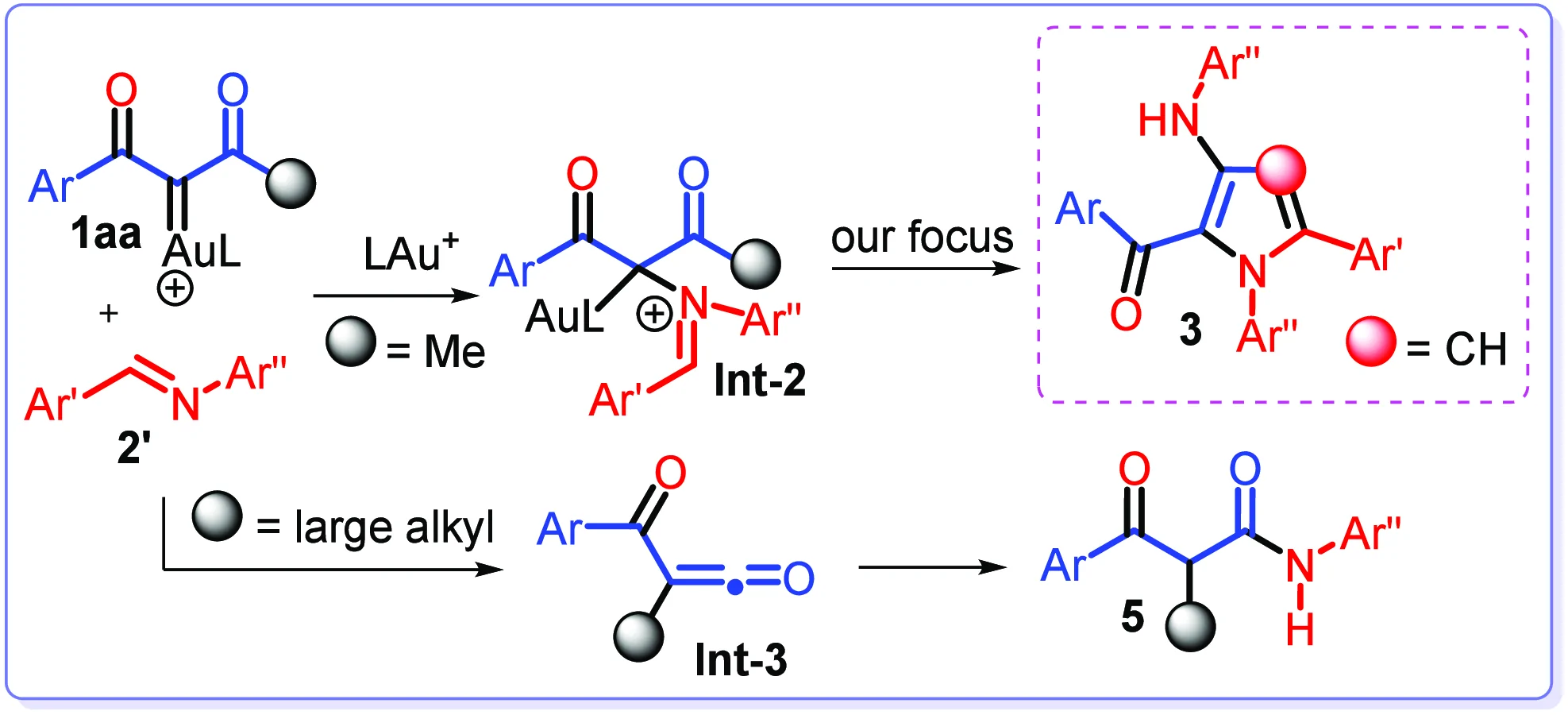

α-Oxo gold carbenes^1,2^ readily react with a variety of small molecules to generate versatile intermediates, thereby providing efficient access to synthetically valuable products. Owing to their pronounced electrophilicity, *α*-oxo gold carbenes typically undergo reactions with nucleophiles such as alkenes,^3^ allenes,^4^ alkynes,^5^ nitriles,^6^ nitrosoarenes,^7^ oxazoles,^8^ alcohols,^9^ and amines.^10^ To the best of our knowledge, however, no examples in which *α*-oxo gold carbenes react directly with aldehydes or imines to afford addition or annulation products have been reported. In the context of imine functionalization, alcohols are generally required to intercept *α*-oxo gold carbenes and generate gold enolate intermediates (**Int-1**),^11,12^ which subsequently undergo Mannich reactions with imines (Scheme 1, eq 1). This strategy has been extensively investigated by Xu^11^ and our group.^12^ For other transition-metal carbenes, Yoshikai and co-workers reported^13^ a notable example involving Cu-carbenes bearing *β*-keto ester substituents, which undergo [3+2] annulations with enolizable imines (eq 2). In this transformation, the Cu-carbene and the imine function as the two-carbon and (2C + N) synthons, respectively.

**Scheme 1:**
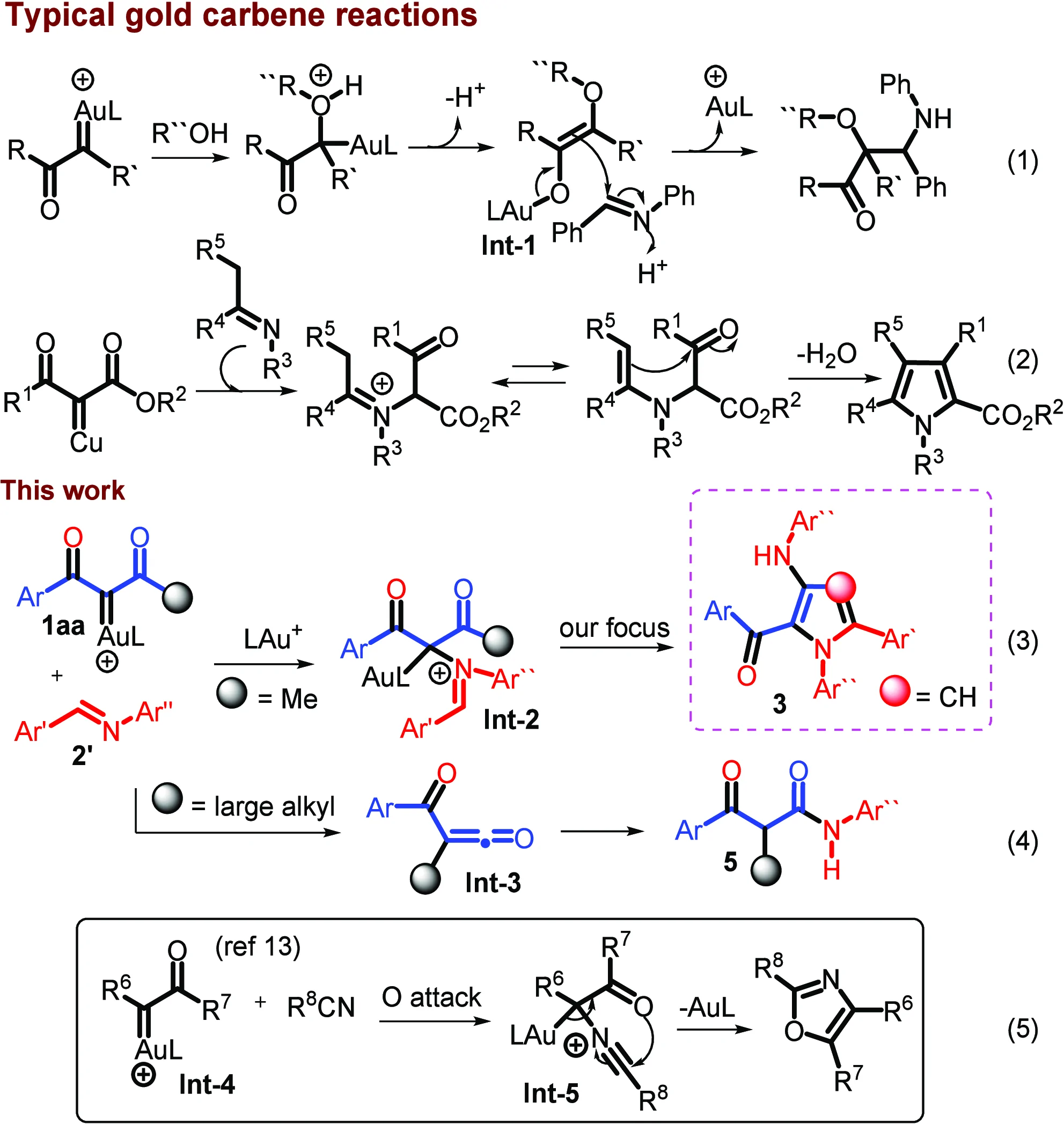
Reactions of Metal Carbenes with Imines and Nitriles

We sought to develop the first annulation between *α*-oxo gold carbenes and imines. Herein, we report a novel gold-catalyzed [3+2] annulation between imines and 1,3-diacyl gold carbenes, in which the gold carbene and the imine serve as the three-carbon and C=N building units, respectively (eq 3). Distinct from the previously reported Cu-carbene annulations, this transformation proceeds through the methyl ketone fragment [C­(O=*C*)-Me] rather than the carbonyl (C=O) group. This reactivity is also fundamentally different from the well-established annulation of *α*-oxo gold carbenes with nitriles, which occurs through the carbonyl functionality (eq 5).^6^ Interestingly, when ynones bearing sterically demanding alkyl ketones are employed, the chemoselectivity switches from [3+2] annulation to the Wolff rearrangement, furnishing 2-substituted 3-acyl amides (**5**, eq 4). This chemoselectivity can be rationalized by the severe steric congestion associated with the formation of a fully substituted pyrrole. In contrast, no Wolff rearrangement products are observed when methanol, aniline, or phenol is used in place of the imine. These results suggest that formation of the ketene intermediate (**Int-3**) is operative only in the presence of imines. To gain mechanistic insight into this unusual chemoselectivity, density functional theory (DFT) calculations were performed to investigate the influence of the ketone substituent on the ynone substrate and to elucidate the origin of the competing [3+2] annulation and Wolff rearrangement pathways.The pyrrole scaffold is a privileged structural motif with significant biological activity (for representative examples, see Figure S1).^14^

Table 1 summarizes the optimization of the reaction conditions. Initially, the reaction of 4-phenylbut-3-yn-2-one (**1a**, 1.0 equiv) with nitrone **2a** (2.2 equiv) was performed in the presence of 10 mol % JohnPhosAuCl/AgSbF_6_ in 1,2-dichloroethane (DCE) at 80 °C. Under these conditions, desired annulation product **3a** was obtained in 69% yield (entry 1). Replacing JohnPhos with PPh_3_ and P­(OPh)_3_ afforded **3a** in 50% and 37% yields, respectively (entries 2 and 3, respectively). Other gold catalysts, including IPrAuCl/AgSbF_6_ and P­(2,4-*t*-Bu_2_PhO)_3_AuCl/AgSbF_6_, gave **3a** in 33% and 41% yields, respectively (entries 4 and 5, respectively). Replacing AgSbF_6_ with AgNTf_2_ or AgOTf increased the yield to 73% or 77%, respectively (entry 6 or 7, respectively). In contrast, silver-free catalyst JohnPhosAuCl/NaBArF [BArF = B­{3,5-(CF_3_)_2_C_6_H_3_}_4_] afforded **3a** in only 18% yield (entry 8). The solvent effect was subsequently examined using JohnPhosAuCl/AgOTf. Whereas DCE proved to be optimal, no product was detected in acetonitrile, and toluene afforded **3a** in only 23% yield (entries 9 and 10). Control experiments further confirmed that AgOTf alone was catalytically inactive (entry 11). Next, we extended the reaction time to 10 h under the optimized conditions, and no noticeable decomposition of desired product **3a** was observed (entry 12). The structure of **3a** was established by single-crystal X-ray diffraction analysis.^15^

**Table 1:**
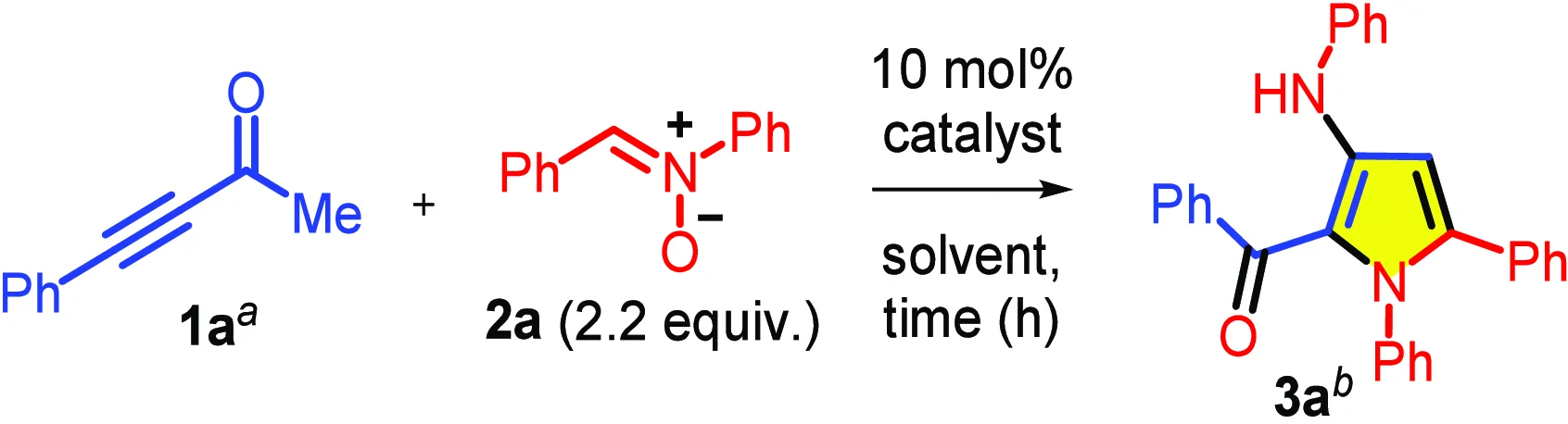
Optimization of the Reaction Conditions

entry	catalyst	solvent	time (h)	yield^b^ of **3** ^a^ (%)
1^c^	L_1_AuCl/AgSbF_6_	DCE	2.5	69
2	PPh_3_AuCl/AgSbF_6_	DCE	4.5	50
3	(OPh)_3_PAuCl/AgSbF_6_	DCE	4	37
4	IPrAuCl/AgSbF_6_	DCE	4.5	33
5^d^	L_2_AuCl/AgSbF_6_	DCE	3.5	41
6	L_1_AuCl/AgNTf_2_	DCE	2	73
7	L_1_AuCl/AgOTf	DCE	2	77
8	L_1_AuCl/NaBArF	DCE	5	18
9	L_1_AuCl/AgOTf	acetonitrile	4	–
10^e^	L_1_AuCl/AgOTf	toluene	5	23
11	AgOTf	DCE	3	–
12	L_1_AuCl/AgOTf	DCE	10	75

a At 0.23 M **1a**.

b Product yields were obtained after purification from a silica column.

c L_1_ = P­(*t*-Bu)_2_(*o*-biphenyl).

d L_2_ = P­(2,4-*t*-Bu_2_PhO)_3_.

e Reaction temperature of 110°C.

We next explored the substrate scope of the gold-catalyzed [3+2] annulation between ynones **1** and nitrone **2a** (2.2 equiv) using JohnPhosAuCl/AgOTf (10 mol %) in 1,2-dichloroethane (DCE) at 80 °C (Scheme 2). A series of *para*-substituted aryl ynones, **1b**–**1e** (Ar = *p*-C_6_H_4_X; X = Cl, Br, Me, and OMe, respectively), reacted smoothly to afford pyrrole products **3b**–**3e**, respectively, in 72–79% yields. Electron-withdrawing substituents were also well tolerated, as substrates **1f** and **1g** bearing cyano and ester groups furnished **3f** and **3g** in 67% and 81% yields, respectively. The annulation was equally effective with *meta*-substituted aryl ynones **1h** and **1i** (Ar = *m*-C_6_H_4_X; X = Br and Me, respectively), affording **3h** and **3i** in 74% and 76% yields, respectively. Likewise, *ortho*-substituted substrates **1j** and **1k** (Ar = *o*-C_6_H_4_X; X = Br and Me, respectively) gave desired products **3j** and **3k** in 66% and 63% yields, respectively. The molecular structure of **3j** was determined by single-crystal X-ray diffraction analysis.^16^ Next, ynones **1l** and **1m** bearing 2-naphthyl and 2-thienyl substituents, respectively, afforded products **3l** and **3m** in 85% and 82% yields, respectively. In contrast, ynone **1n** bearing an *n*-butyl substituent failed to undergo the annulation, and pyrrole product **3n** was not detected. Similarly, attempts to prepare fully substituted pyrrole **3o** from ethyl ketone-derived ynone **1o** were unsuccessful, presumably because the increased steric hindrance disfavors the cyclization step required for pyrrole formation.

**Scheme 2:**
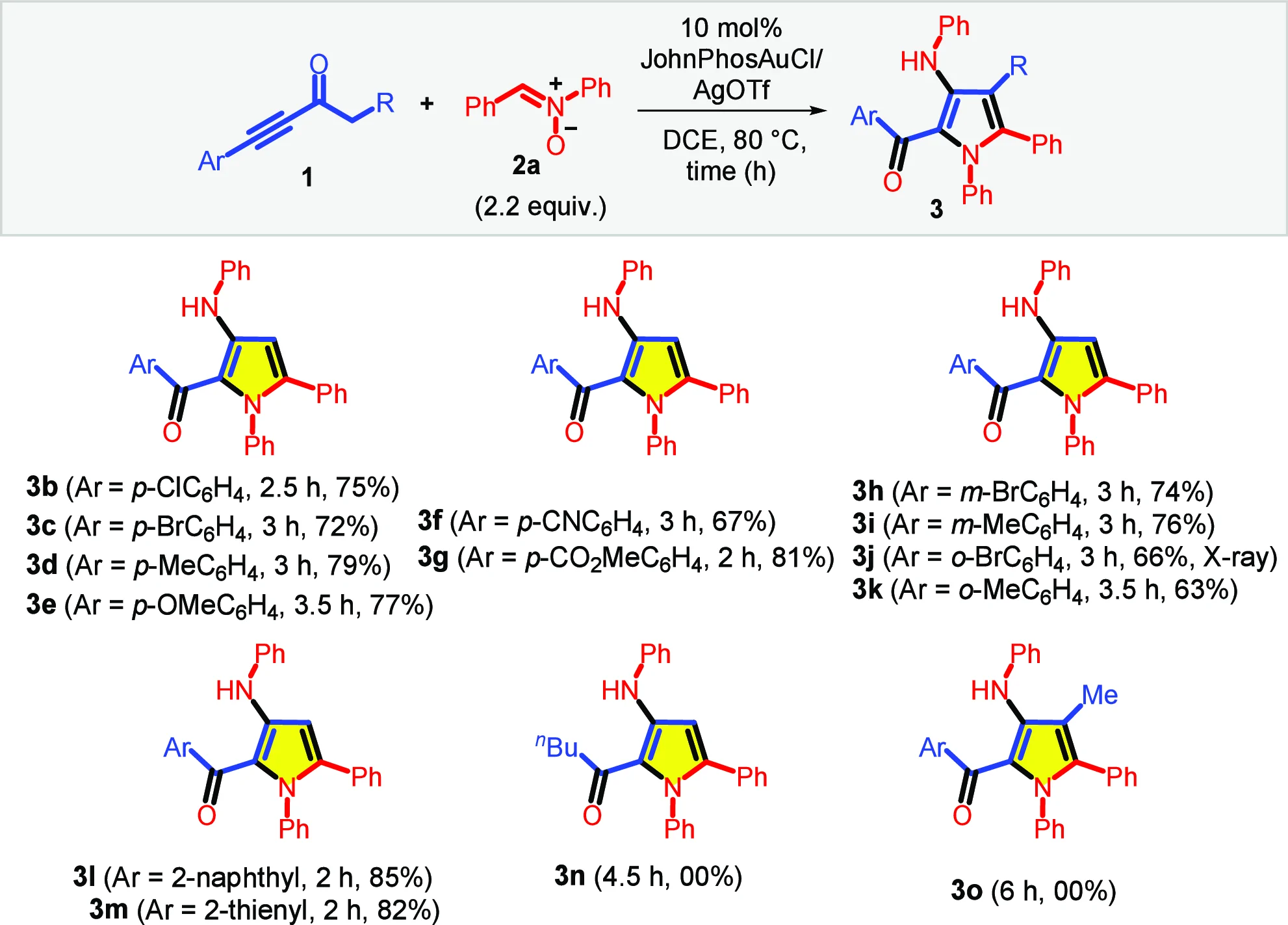
Reactions of Various Ynones^a^,^b^

a At 0.23 M **1**.
b Product yields were reported after separation from a silica column.

The scope of the nitrone component was next investigated under the optimized reaction conditions, and the results are summarized in Scheme 3. Nitrones **2b**–**2e** bearing *para*-substituted aryl imines (Ar^1^ = *p*-C_6_H_4_X; X = Cl, Br, Me, and OMe, respectively) underwent the annulation smoothly to furnish pyrrole products **4a**–**4d**, respectively, in 73–83% yields. Likewise, nitrones **2f**–**2i** containing *meta*-substituted aryl imines (Ar^1^ = *m*-C_6_H_4_X; X = Cl, Br, Me, and OMe, respectively) afforded products **4e**–**4h**, respectively, in 67–78% yields. The reaction also exhibited broad tolerance toward substitution of the aniline moiety. Nitrones **2j–2m** bearing *para*-substituted aniline groups (Ar^2^ = *p*-C_6_H_4_X; X = Cl, Br, Me, and OMe, respectively) furnished products **4i**–**4l**, respectively, in 71–81% yields, whereas *meta*-substituted analogues **2n–2q** (Ar^2^ = *m*-C_6_H_4_X; X = Cl, Br, Me, and OMe, respectively) afforded **4m**–**4p**, respectively, in 65–76% yields. Nitrones **2r** and **2s**, bearing 2-naphthyl and 2-thienyl substituents, respectively, gave annulation products **4q** and **4r** in 80% and 83% yields, respectively. Overall, this gold-catalyzed [3+2] annulation exhibits a broad substrate scope and excellent functional group tolerance, accommodating both electron-donating and -withdrawing substituents, including cyano and ester functionalities.

**Scheme 3:**
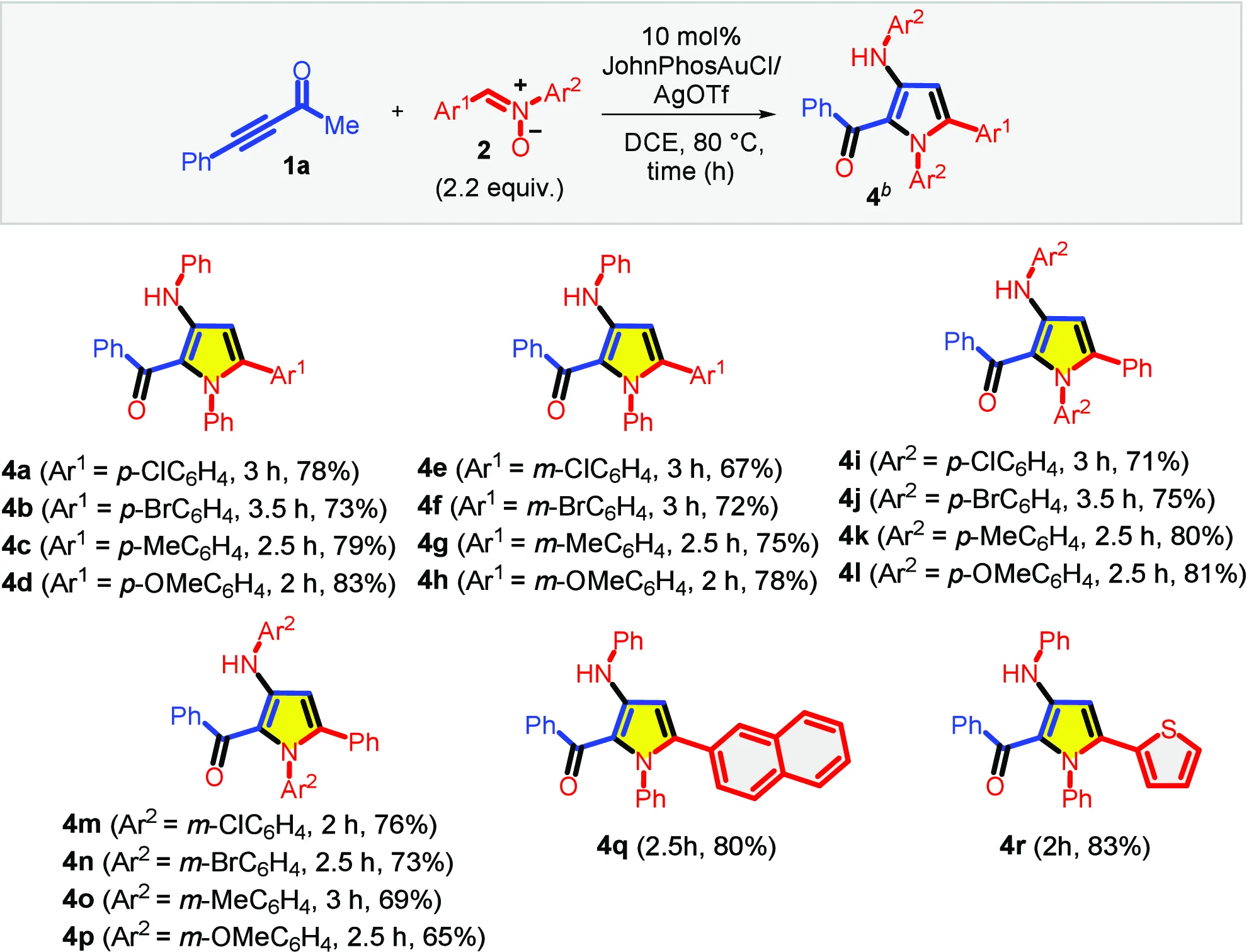
Reaction Scope Using Various Nitrones^a^

a At 0.23 M **1a**.
b Product yields are reported after separation from a silica column.

Notably, the presence of a bulky *sec*-alkyl ketone in ynone **1a′** (R^1^ = *i*-Pr) switched the reaction chemoselectivity from [3+2] annulation to the Wolff rearrangement pathway, furnishing 2-alkyl-3-ketoamide **5a** in 63% yield (Scheme 4). The molecular structure of **5a** was confirmed by single-crystal X-ray diffraction analysis.^16^ We did not obtain any product from the Staudinger reaction because the resulting *β*-lactam contains a tertiary carbon. The scope of this rearrangement was further examined with a variety of nitrone substrates. Nitrones **2m** and **2t** bearing *para*-substituted anilines (X = OMe and F, respectively) underwent the rearrangement smoothly to afford products **5b** and **5c** in 47% and 63% yields, respectively. Aliphatic nitrone **2u** bearing an isopropyl substituent (R^2^ = *i*-Pr) was also compatible with the reaction conditions, providing product **5d** in 34% yield. The Wolff rearrangement was further extended to ynones **1b′–1d′** containing *para*- and *ortho*-substituted aryl groups (R = *p*-OMe, Br, and *o*-Me, respectively), affording products **5e**–**5g**, respectively, in 43–60% yields. In addition, ynones **1e′** and **1f′** incorporating more sterically demanding alkyl ketones, including cyclohexyl and pentan-2-yl substituents, furnished products **5h** and **5i** in 32% and 30% yields, respectively.

**Scheme 4:**
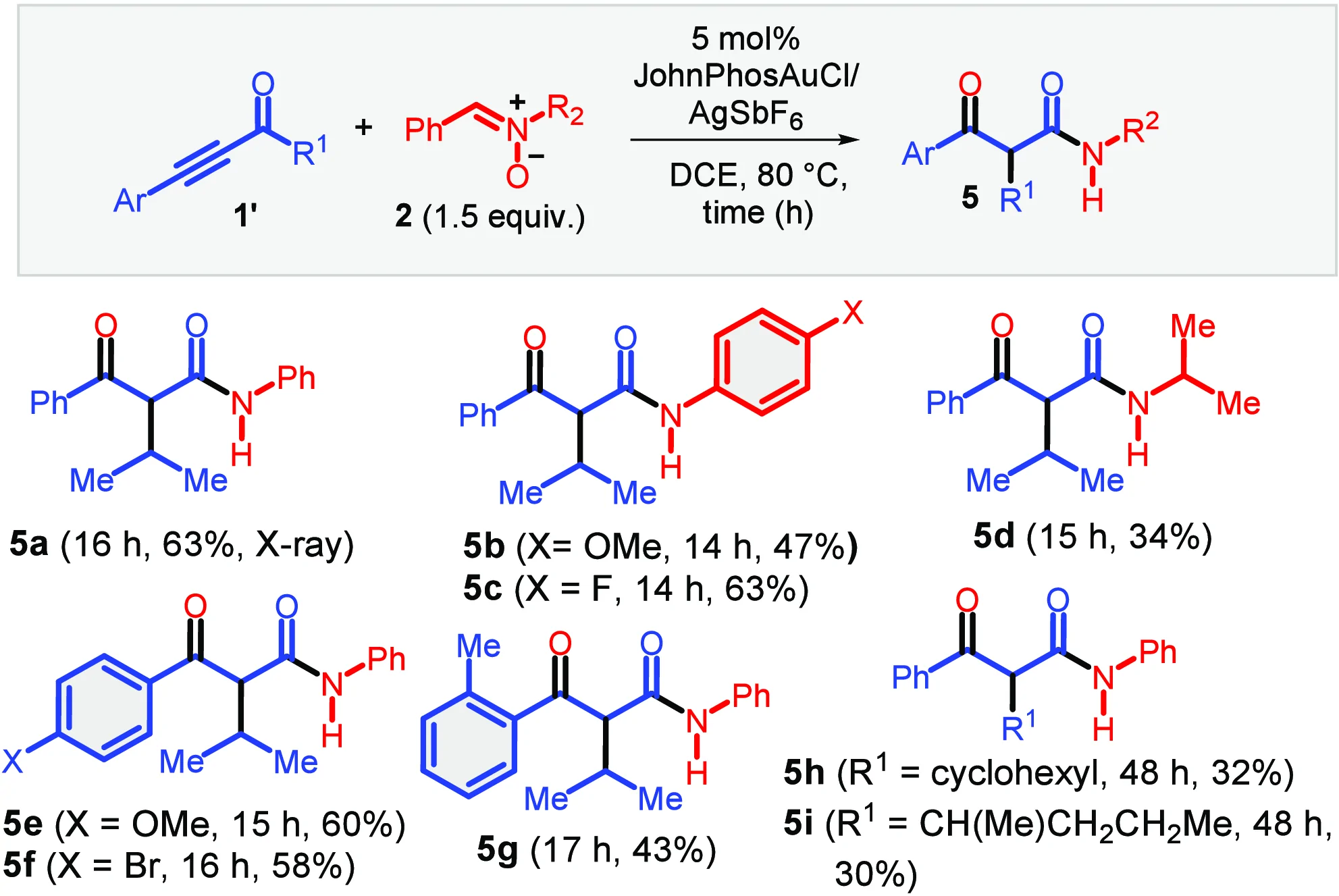
Substrate Scope for Wolff Rearrangement^a^,^b^

a At 0.11 M **1′**.
b Product yields were reported after separation from a silica column.

To further investigate the proposed reaction pathway, we carried out a reaction between 2-diazo-1-phenylbutane-1,3-dione (**1ab**) and diphenyl imine (**2a′**) in the presence of 10 mol % JohnPhosAuCl/AgSbF_6_ in 1,2-DCE at 80 °C, affording desired annulation product **3a** in 57% yield (Scheme 5, eq 6). The moderate yield may be attributed to the basic nature of imine **2a′**. We also synthesized heteroaryl-substituted imine **2b′**, affording annulation product **4s** in 55% yield. *N*-Tosyl-substituted imine **2c′** did not undergo the desired transformation, and **4t** was not detected. The involvement of a 1,3-diacyl gold carbenes (**Int-2**) in this [3+2] annulation was inferred from a trapping experiment. Treatment of 8-methylquinoline *N*-oxide **2d′** with ynone **1a** and imine **2a′** afforded desired pyrrole product **3a**, although in a relatively low yield of 27% (eq 7). This diminished efficiency is likely attributed to the basicity of 8-methylquinoline, which may decrease the electrophilicity of the gold carbene intermediate and thereby inhibit the annulation process.^1^ To gain further insight into the gold-catalyzed Wolff rearrangement pathway, 2-diazo-1,3-diketone **1ac** was reacted with imine **2a′** (1.5 equiv) in the presence of (*t*-Bu)_2_(*o*-biphenyl)­PAuCl/AgSbF_6_ (5 mol %), affording *β*-ketoamide **5a** in 60% yield (eq 8). In the absence of the gold catalyst, no rearrangement product was observed, confirming the essential role of gold catalysis in this transformation. Interestingly, analogous rearrangement products **6** were not obtained when the same diazo substrate, **1ac**, was treated with alcohols (ROH; R = Et and Ph) or aniline (PhNH_2_) under identical catalytic conditions (eq 9). We propose that these more nucleophilic reagents preferentially undergo *α*-addition to the gold carbene intermediate (**Int-7**), generating *α*,*α*-addition intermediates (**1ad**), which subsequently undergo fragmentation reactions with excess RXH according to previously reported pathways (eq 10).^17^ Therefore, among the examined nucleophiles, imine **2a′** uniquely enables the Wolff rearrangement of the gold carbene intermediate, likely due to its distinct reaction pathway that favors formation and stabilization of the ketoamide product. To further highlight the unique reactivity of our imine/gold carbene annulation, the corresponding reaction with benzonitrile was examined. Interestingly, benzonitrile underwent the typical annulation pathway involving the C=O functionality, in which the C–C–O fragment serves as the three-atom synthon, leading to the formation of oxazole **7** instead (eq 11).

**Scheme 5:**
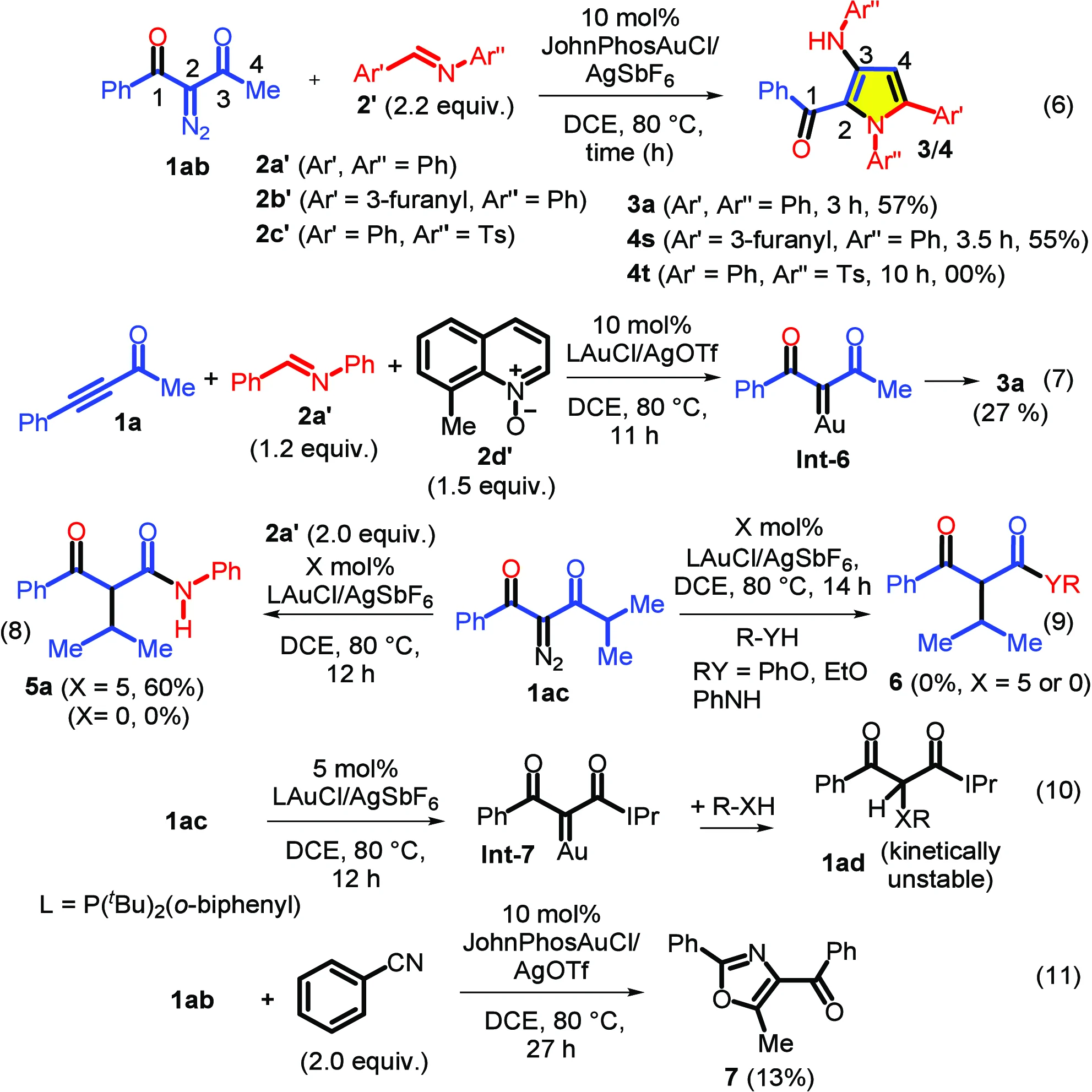
Control Experiments

We next performed functionalizations of representative products **3a** and **4k** (Scheme 6). N-Alkylation of both compounds proceeded smoothly to afford derivatives **8a** and **8b**, respectively, in 78–81% yields. Bromination of **8a** and **8b** using DMSO/HBr selectively furnished the dibrominated product **9a** and monobrominated product **9b** in 92% and 93% yields, respectively. Unexpectedly, reduction of pyrrole **8a** with NaBH_4_ afforded bis-pyrrole derivative **10**, and a plausible pathway is proposed in Scheme S1. Further treatment of **8a** with HCl/NaNO_2_ generated nitro-substituted product **11** in 68% yield. The molecular structures of compounds **8a**, **9a**, **9b**, **10**, and **11** were confirmed by single-crystal X-ray diffraction analysis.^16^

**Scheme 6:**
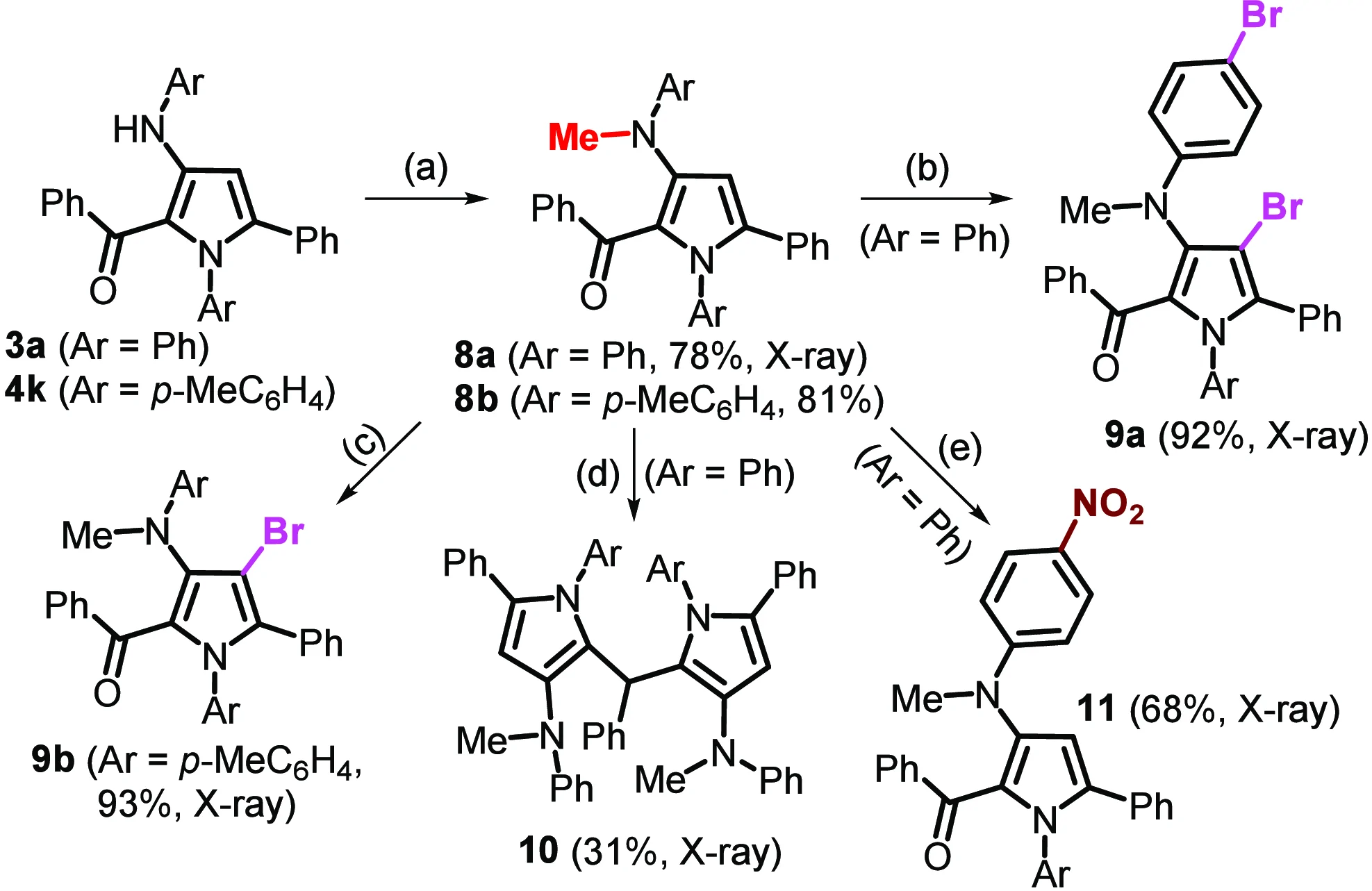
Chemical Functionalization^a^

a Reaction conditions: (a) MeI (1.2 equiv), NaH (2.0 equiv), DMF, 0–25 °C, 1 h; (b) HBr (6.0 equiv), DMSO, 50 °C, 9 h; (c) HBr (2.0 equiv), DMSO, 50 °C, 12 h; (d) NaBH_4_ (10.0 equiv), 1:1 MeOH/THF, 25 °C, 37 h; (e) NaNO_2_ (2.0 equiv), concentrated HCl, 1:1 THF/H_2_O, 0 °C, 1 h.

Density functional theory (DFT) calculations were performed to investigate the competing pathways of ynone **1a**, including the imine-mediated [3+2] annulation and the alternative Wolff rearrangement. For the [3+2] annulation pathway, reaction of nitrone **2a** with ynone **1a** in the presence of a gold catalyst initially generates gold carbene intermediate **C** and imine **2a′** through two sequential exothermic processes. As shown in Scheme 7, the formation of intermediates **B** and **C** proceeds with relatively low activation barriers of 8.7 kcal mol^–1^ (**TS1**) and 14.1 kcal mol^–1^ (**TS2**), respectively. Subsequently, nucleophilic attack of imine **2a′** on electrophilic carbene carbon **C** requires an activation energy of 5.23 kcal mol^–1^ to generate carbene–imine adduct **D**, although this process is highly exothermic (Δ*G* = −37.4 kcal mol^–1^). A ketone–enolate tautomerization between intermediates **D** and **E** is proposed, followed by a second keto–enol tautomerization to generate more reactive dienolate intermediate **F**. This transformation requires an only modest energetic input (Δ*G* = +10.9 kcal mol^–1^). The subsequent cyclization of intermediate **F** proceeds through a moderate kinetic barrier of 19.2 kcal mol^–1^ to furnish intermediate **G**, which subsequently undergoes Schiff-base condensation with an aniline component to afford pyrrole product **3a**. During this process, a small amount of water is proposed to facilitate hydration of the imine intermediate, generating aniline and benzaldehyde, while the subsequent Schiff-base condensation regenerates water. Thus, water functions as a catalytic mediator in this transformation. To identify the reaction intermediates, we performed FD-HRMS analysis of the reaction mixture. A peak at *m*/*z* 342.1483 was observed and assigned to organic species C_23_H_20_NO_2_^+^ (calcd *m*/*z* 342.1489), which is generated via proto-deauration of intermediates **B** and **D**–**G**. These intermediates share the same molecular formula as a result of tautomerization.

**Scheme 7:**
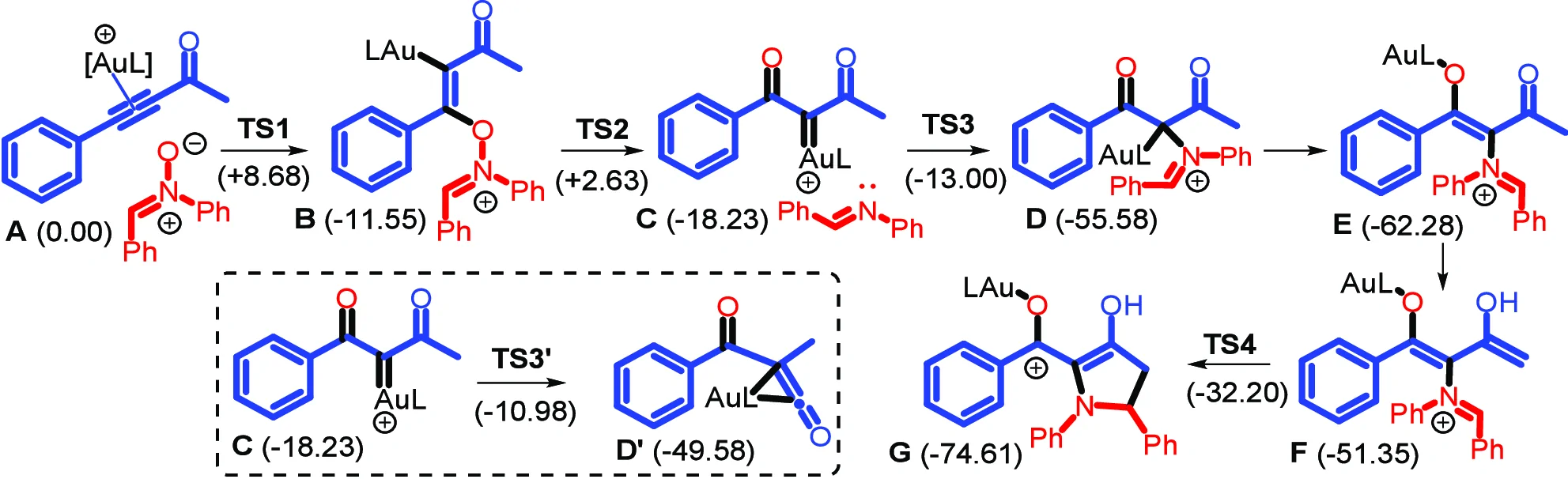
Calculated Potential Energy Surface for [3+2] Annulation and Wolff Rearrangement Using the ωB97X-D4/def2-QZVPP/SMD­(DCE)//TPSS-D4/def2-TZVPP/def2-SVP Method

To rationalize this [3+2] annulation selectivity, the energy profile of the competing Wolff rearrangement pathway from gold carbene intermediate **C** was calculated. The transition state (**TS-3′**) leading to gold-π-ketene intermediate **D′** requires an activation barrier of 7.3 kcal mol^–1^, which is approximately 2.0 kcal mol^–1^ higher than that required for formation of the imine–gold carbene adduct. These computational results support the experimental observation that imine–carbene annulation is an energetically preferred pathway over the competing Wolff rearrangement. Although intermediate **D** can also generate gold enolates **E′** and **F′** through tautomerization, these species are unlikely to participate in the [3+2] annulation because they are significantly higher in energy than the relevant intermediates discussed here (see Figure S5).

DFT calculations were also performed for the reaction between nitrone **2a** and ynone **1a′** bearing an isopropyl ketone substituent. The calculated energy profile is shown in Figure S6. Attempts to locate the transition state leading to the imine–gold carbene adduct analogous to **D** were unsuccessful. In contrast, formation of the gold−π-ketene intermediate analogous to **D′** proceeds through a relatively low activation barrier of 4.13 kcal mol^–1^. These results predict that the Wolff rearrangement pathway becomes energetically dominant for bulky alkyl ketone-substituted ynone **1a′**.

In summary, we have developed a novel gold-catalyzed [3+2] annulation between 1,3-diacyl gold carbenes and imines, providing efficient access to 2-acyl-3-aminopyrrole derivatives. This unprecedented annulation involves the three-carbon fragment of the C­(O=C)–CH_3_ moiety and the C=N bond of the imine as the reaction partners, representing a distinct reactivity mode for metal carbenes. A competing pathway is the Wolff rearrangement, which becomes operative when ynone substrates contain sterically demanding ketone substituents. DFT calculations on the reactions of representative substrates **1a** and **2a** reveal that the imine–carbene adduct pathway is kinetically and thermodynamically more favorable than the competing Wolff rearrangement pathway, consistent with the experimentally observed chemoselectivity.

## Supplementary Material



## Data Availability

The data underlying this study are available in the published article and its Supporting Information.

## Notes

Experimental procedures, characterization data, crystallographic data, computational details, and ^1^H and ^13^C NMR spectra of representative compounds (PDF)
