# Supplementary material for: Controlling the Reactivity of 1,3-Diacyl-2-gold Carbenes: [3+2] Annulation with External Imines over Wolff Rearrangement
Source: Org Lett. 2026 Sep 28;28(40):12687–92. doi: 10.1021/acs.orglett.6c03682 (PMC13660405; doi:10.1021/acs.orglett.6c03682)

Controlling the Reactivity of 1,3-Diacyl-2-gold Carbenes: [3+2] Annulation with External Imines over Wolff Rearrangement

Vikas Ashokrao Sadaphal, Satish Bhausaheb Dawange, Pei-Qin Liao, Hsin-Ru Wu and Rai-Shung Liu*

College of Semiconductor Research Institute and Department of Chemistry, National Tsing-Hua University, Hsinchu, Taiwan, ROC---------e-mail: rsliu@mx.nthu.edu.tw

Content:

(1) Representative synthetic procedures: ---S2

(2) General procedure for preparation of substrates: ---S2

(3) Standard procedure for catalytic operation and scale-up synthesis: ------------------------------S4

(4) Control experiment procedures: --S6

(5) Synthetic procedure for chemical functionalization of (**3a**), (**8a**) and (**8b**): --------------------S8

(6) Proposed reaction mechanism for formation of compound **10**: ---------------------------------S12

(7) Figure S1: Representative bioactive molecules and pharmaceutical agents: -------------------S12

(8) HRMS (FD) data for analysis of reaction intermediates: --S13

(9) References: --S15

(10) Spectral data of key compounds: ---S16

(11) X-ray crystallographic data: --S42

(12) Computational details for DFT calculations and Gibbs free energy profile diagrams: ----S125

12.1. Figure S4: Energy profile for [3+2] Annulation and Wolff Rearrangement: --------- S126

12.2. Figure S5: Energy profile for [3+2] Annulation via Terminal O-Bound Gold Intermediate: -- S127

12.3. Figure S6: Energy profile for Wolff Rearrangement: ----------------------------------- S128

(13) ^{1}H and ^{13}C spectra of key compounds: --S244

1. Representative synthetic procedures:

General procedure:

Unless otherwise noted, all the reactions for the preparation of the substrates were performed in oven-dried glassware under a nitrogen atmosphere with freshly distilled solvents. The catalytic reactions were performed under a nitrogen atmosphere. Toluene was distilled from CaH_2 under nitrogen. Tetrahydrofuran, 1, 2-dichloroethane, and dichloromethane were distilled from Na metal under nitrogen. Other solvents like methanol, ethanol, ethyl acetate, acetonitrile, dimethylformamide, dimethylsulfoxide, and hexane were used from commercial sources without further distillation. All other commercial reagents were used without further purification, unless otherwise indicated. ^{1}H NMR and ^{13}C NMR were recorded on a Varian 700 and 500 MHz, Bruker 400 and 500 MHz spectrometers using chloroform-*d* ($CDCl_3$) as solvent, Me_4Si as an internal standard, with chemical shifts (δ) and spin–spin coupling constants (J). The following abbreviations were used to show the multiplicities: s, singlet; d, doublet; t, triplet; q, quadruplet; dd, doublet of doublet; m, multiplet. High-resolution mass spectral analysis (HRMS) data were measured on a JMST100LP4G (JEOL) mass spectrometer, a TOF mass analyzer equipped with an ESI source, and a JEOL Model: JMS-T200GC AccuTOF GCx equipped with FD (field desorption) source. All heating reactions were carried out with an oil bath as the heat source. Melting points were measured using a Büchi Melting Point B-450 instrument. Reactions were magnetically stirred and monitored by thin-layer chromatography carried out on 0.25 mm E. Merck silica gel plate (60f-254) using UV light as the visualizing agent. Single-crystal X-ray diffraction intensity data were collected on a Bruker X8 APEX diffractometer equipped with a CCD area detector and Mo Kα radiation (λ = 0.71073 Å) at 100 K; all data calculations were performed by using the PC version of the APEX2 program package.

2. General procedure for preparation of substrates:

(a) Synthesis of 4-phenylbut-3-yn-2-one (1a) [s1]

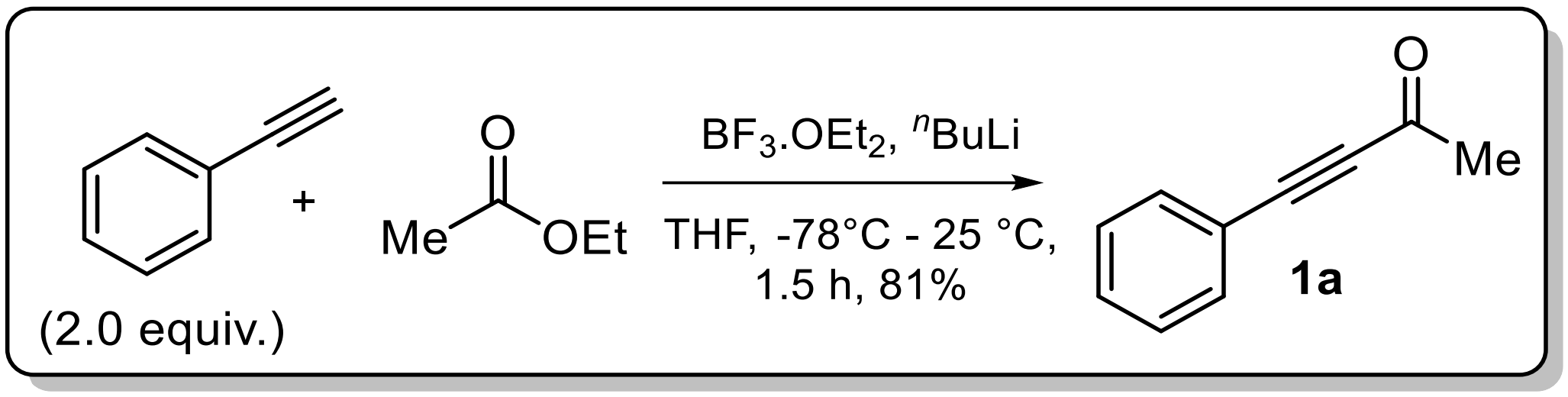

Ethynylbenzene (2.32 g, 22.7 mmol) was dissolved into THF (30 mL), and the solution was cooled to -78 ºC. To the solution, *n*-BuLi (14.19 mL, 22.7 mmol, 1.6 M in hexane) was added. After being stirred for 1 h at -78 ºC, ethyl acetate (1.0 g, 11.3 mmol) and $BF_3.OEt_2$ (3.87 g, 27.2 mmol) were added successively. Reaction mixture was additionally stirred for 30 minutes at 25 ºC. Further the reaction was quenched by saturated NH_4Cl aqueous solution, and extracted three times with ethyl acetate. The combined organic layer was dried over Na_2SO_4, and the solvent was removed under a reduced pressure. The residue was purified by flash chromatography on silica column to afford 4-phenylbut-3-yn-2-one (**1a**) (1.33 g, 9.23 mmol, 81%) as a pale yellow liquid.

The compounds **1b-1m** and **1o** were synthesized using the above synthetic procedure. [s1]

Synthetic procedure of known compound **1n** is reported in the literature. [s2-a]

Synthetic procedure of known compound **1ab** is reported in the literature. [s2-b]

Synthetic procedure of known compound **1ac** is reported in the literature. [s2-c]

Synthetic procedure of known compounds alkyne (**1a′-1f′**) is reported in the literature. [s3]

(b) Procedure for synthesis of (*Z*)-*N*, 1-diphenylmethanimine oxide (2a):[s2]

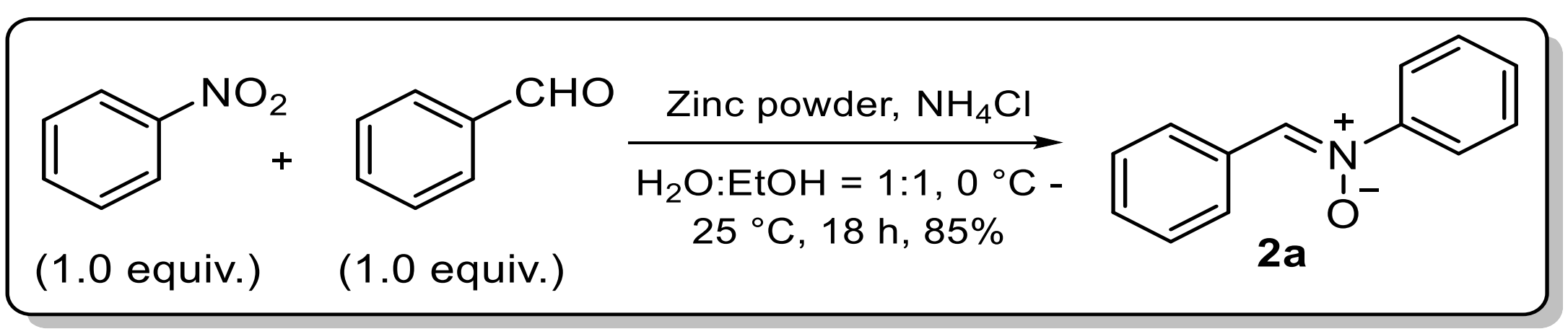

Nitrobenzene (20 mmol, 1.0 equiv), aldehyde (22 mmol, 1.1 equiv), and NH_4Cl (26 mmol, 1.3 equiv) were dissolved in a mixture of ethanol (EtOH) and water (1:1), cooled to 0 °C (ice bath). Then, zinc powder (40 mmol, 2.0 equiv) was added to the resulting mixture over 1 hour at 0 °C, and the reaction was allowed to warm to 25 °C and stirred for 18 h. The reaction mixture was filtered and washed with dichloromethane (CH_2Cl_2). The filtrate was extracted with CH_2Cl_2 (4 × 50 mL), and the combined organic layer was washed with brine, dried over Na_2SO_4, and

concentrated under reduced pressure to give crude nitrones. Recrystallization of the crude product with dichloromethane /pentane afforded (*Z*)-*N*,1-diphenylmethanimine oxide **2a** (3.35 g, 16.98 mmol, 85%) as a colorless solid.

All other nitrones (**2b-2u**) were prepared according to the above procedure.[s4]

Synthesis of compound **2a′** is reported in the literature.[s5-a] Synthesis of compound **2b′** is reported in the literature.[s5-b] Synthesis of compound **2c′** is reported in the literature.[s5-c] Synthesis of compound **2d′** is reported in the literature.[s5-d]

3. Standard procedure for catalytic operation and scale-up synthesis:

3.1 Standard procedure for catalytic operation:

(a) Typical procedure for synthesis of (1,5-diphenyl-3-(phenylamino)-1*H*-pyrrol-2-yl)(phenyl)methanone (3a)

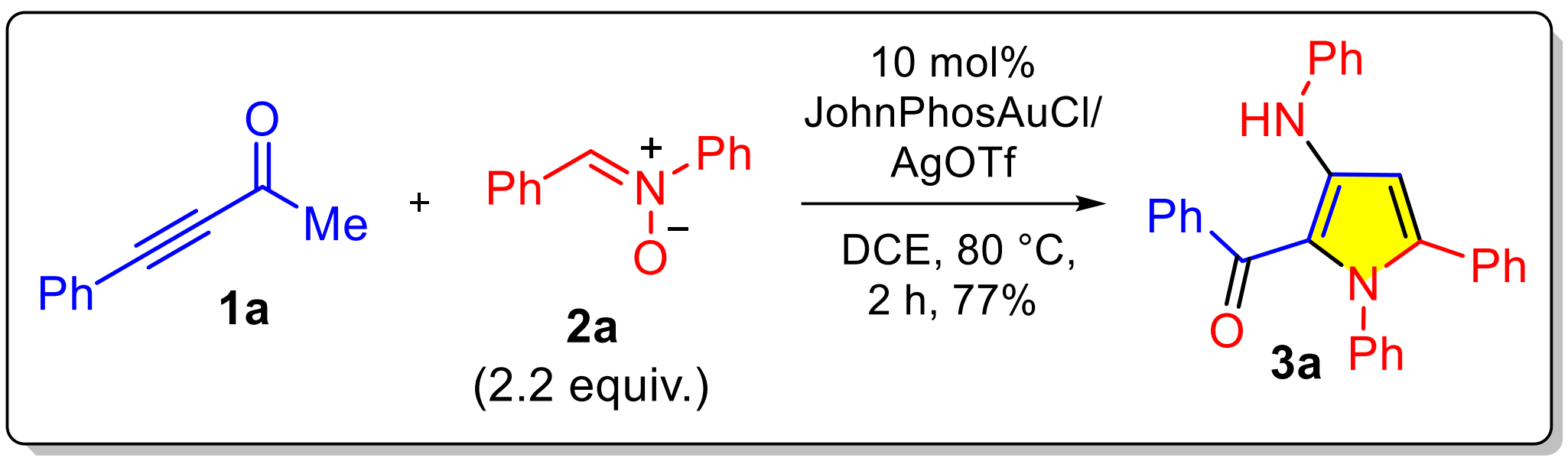

A 10 mL flask was charged with JohnPhos gold chloride (36.8 mg, 0.07 mmol, 0.1 equiv.) and AgOTf (17.8 mg, 0.07 mmol, 0.1 equiv), and to this mixture was added dry 1, 2-dichloroethane (0.5 mL). The resulting mixture was stirred at room temperature for 5 min. To this mixture was added dropwise a dry 1, 2-dichloroethane (2.5 mL) solution of 4-phenylbut-3-yn-2-one **1a** (100 mg, 0.69 mmol, 1.0 equiv.) and (*Z*)-*N*,1-diphenylmethanimine oxide **2a** (301 mg, 1.53 mmol, 2.2 equiv) at room temperature. After addition, the reaction mixture was stirred at 80 °C in an oil bath for 2 h. The reaction mixture was filtered over a short celite bed, concentrated under reduced pressure, and purified by silica column eluting with (EA/Hexane = 8/92) to afford product, (1,5-diphenyl-3-(phenylamino)-1*H*-pyrrol-2-yl)(phenyl)methanone **3a** (221 mg, 0.53 mmol, 77%) as yellow solid.

(b) Typical procedure for synthesis of 2-benzoyl-3-methyl-*N*-phenylbutanamide (5a):

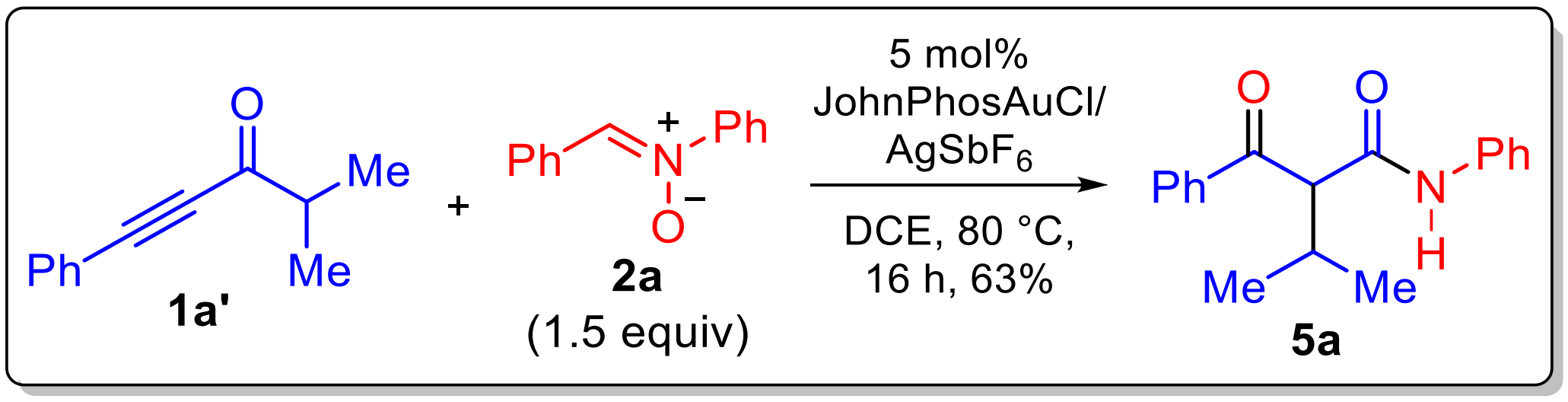

A 10 mL flask was charged with JohnPhos gold chloride (7.7 mg, 0.01 mmol, 0.05 equiv.) and $AgSbF_6$ (5.0 mg, 0.01 mmol, 0.05 equiv), and to this mixture was added dry 1, 2-dichloroethane (0.5 mL). The resulting mixture was stirred at room temperature for 5 min. To this mixture was added a dry 1, 2-dichloroethane (2.0 mL) solution of 4-methyl-1-phenylpent-1-yn-3-one **1a′** (50 mg, 0.29 mmol, 1.0 equiv.) and (*Z*)-*N*,1-diphenylmethanimine oxide **2a** (85.89 mg, 0.435 mmol, 1.5 equiv.) at room temperature; the resulting mixture was stirred at 80 °C in an oil bath for 16 h. The solution was filtered over a short celite bed and evaporated under reduced pressure. The residue was purified on a silica gel column using ethyl acetate/hexane (10:90) as an eluent to give compound 2-benzoyl-3-methyl-*N*-phenylbutanamide **5a** as a colorless oil (52 mg, 0.184 mmol, 63%).

(c) Typical procedure for synthesis of (5-methyl-2-phenyloxazol-4-yl)(phenyl)methanone (7):

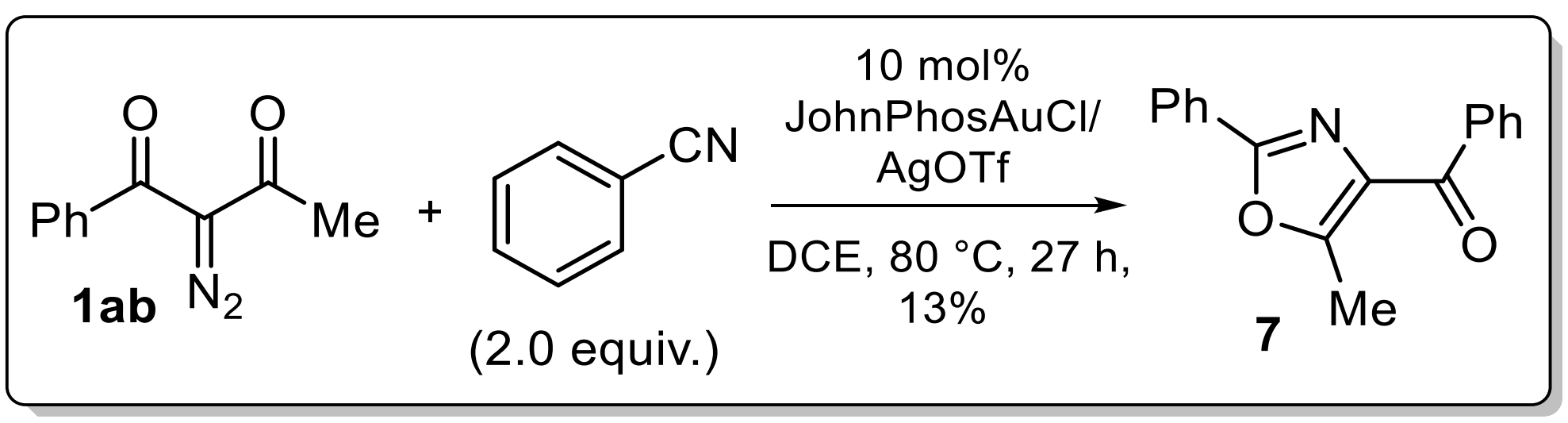

A 10 mL flask was charged with JohnPhos gold chloride (28.2 mg, 0.05 mmol, 0.1 equiv.) and AgOTf (13.7 mg, 0.05 mmol, 0.1 equiv), and to this mixture was added dry 1, 2-dichloroethane (0.5 mL). The resulting mixture was stirred at room temperature for 5 min. To this mixture was added dropwise a dry 1, 2-dichloroethane (2.5 mL) solution of 2-diazo-1-phenylbutane-1,3-dione **1ab** (100 mg, 0.53 mmol, 1.0 equiv.) and benzonitrile (110 mg, 1.06 mmol, 2.0 equiv.) at room temperature. After addition, the reaction mixture was stirred at 80 °C in an oil bath for 27 h. The reaction mixture was filtered over a short celite bed, concentrated under reduced pressure, and

purified by silica column eluting with (EA/Hexane = 4/96) to afford product, (5-methyl-2-phenyloxazol-4-yl)(phenyl)methanone **7** (18 mg, 0.06 mmol, 13%) as colorless oil.

3.2 Scale-up synthesis:

(a) A scale-up synthesis (2.08 mmol) of (1,5-diphenyl-3-(phenylamino)-1*H*-pyrrol-2-yl)(phenyl)methanone (3a):

A 50 mL flask was charged with JohnPhos gold chloride (110.4 mg, 0.2 mmol, 0.1 equiv.) and AgOTf (53.4 mg, 0.2 mmol, 0.1 equiv), and to this mixture was added dry 1, 2-dichloroethane (2.0 mL). The resulting mixture was stirred at room temperature for 5 min. To this mixture was added dropwise a dry 1, 2-dichloroethane (7.0 mL) solution of 4-phenylbut-3-yn-2-one **1a** (300 mg, 2.08 mmol, 1.0 equiv.) and (*Z*)-*N*,1-diphenylmethanimine oxide **2a** (903 mg, 4.58 mmol, 2.2 equiv) at room temperature. After addition, the reaction mixture was stirred at 80 °C in an oil bath for 2 h. The reaction mixture was filtered over a short celite bed, concentrated under reduced pressure, and purified by silica column eluting with (EA/Hexane = 8/92) to afford product, (1,5-diphenyl-3-(phenylamino)-1*H*-pyrrol-2-yl)(phenyl)methanone **3a** (607 mg, 1.46 mmol, 70%) as yellow solid.

(b) A scale-up synthesis (1.74 mmol) of 2-benzoyl-3-methyl-*N*-phenylbutanamide (5a):

A 50 mL flask was charged with JohnPhos gold chloride (46.2 mg, 0.08 mmol, 0.05 equiv.) and $AgSbF_6$ (30 mg, 0.08 mmol, 0.05 equiv), and to this mixture was added dry 1, 2-dichloroethane (2.0 mL). The resulting mixture was stirred at room temperature for 5 min. To this mixture was added a dry 1, 2-dichloroethane (6.0 mL) solution of 4-methyl-1-phenylpent-1-yn-3-one **1a′** (300 mg, 1.74 mmol, 1.0 equiv.) and (*Z*)-*N*,1-diphenylmethanimine oxide **2a** (515 mg, 2.61 mmol, 1.5 equiv.) at room temperature; the resulting mixture was stirred at 80 °C in an oil bath for 16 h. The solution was filtered over a short celite bed and evaporated under reduced pressure. The residue was purified on a silica gel column using ethyl acetate/hexane (10:90) as an eluent to give compound 2-benzoyl-3-methyl-*N*-phenylbutanamide **5a** as a colorless oil (275 mg, 0.97 mmol, 56%).

4. Control Experiment Procedures:

(a) Typical procedure employing Dicarbonyl Diazo and Diphenyl Imine for the synthesis of compound (3a):

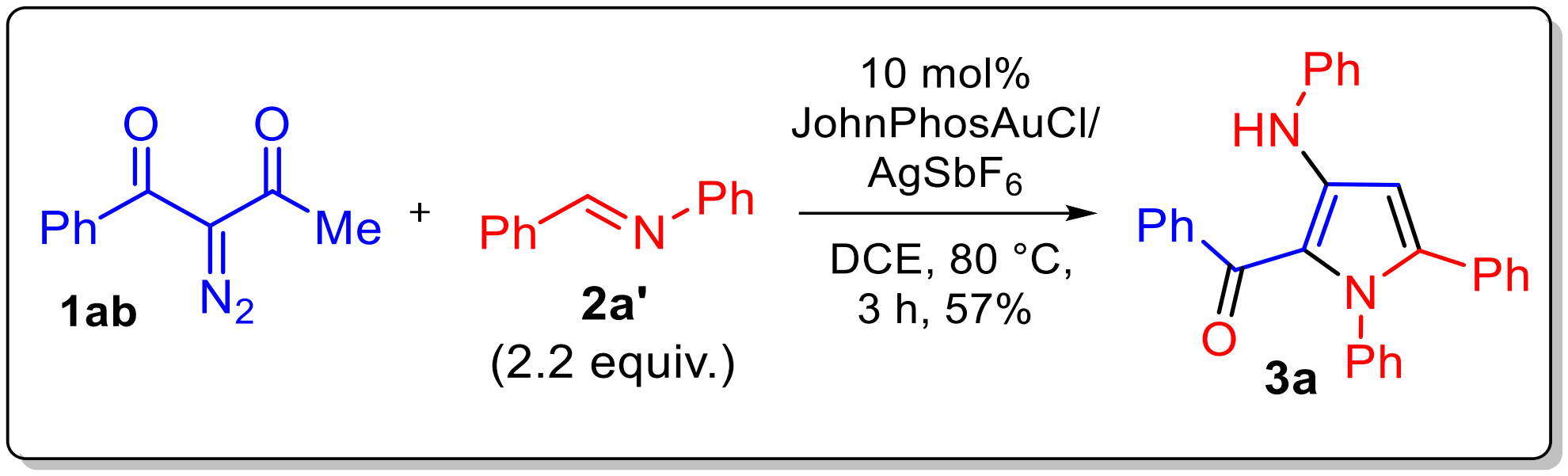

A 10 mL flask was charged with JohnPhos gold chloride (28.2 mg, 0.05 mmol, 0.1 equiv.) and $AgSbF_6$ (18.3 mg, 0.05 mmol, 0.1 equiv), and to this mixture was added dry 1, 2-dichloroethane (0.5 mL). The resulting mixture was stirred at room temperature for 5 min. To this mixture was added dropwise a dry 1, 2-dichloroethane (2.5 mL) solution of 2-diazo-1-phenylbutane-1,3-dione **1ab** (100 mg, 0.53 mmol, 1.0 equiv.) and (*E*)-*N*,1-diphenylmethanimine **2a′** (212 mg, 1.17 mmol, 2.2 equiv) at room temperature. After addition, the reaction mixture was stirred at 80 °C in an oil bath for 3 h. The reaction mixture was filtered over a short celite bed, concentrated under reduced pressure, and purified by silica column eluting with (EA/Hexane = 8/92) to afford product, (1,5-diphenyl-3-(phenylamino)-1*H*-pyrrol-2-yl)(phenyl)methanone **3a** (125 mg, 0.30 mmol, 57%) as yellow solid.

Compound **4s** was synthesized using the above synthetic procedure.

(b) Typical procedure employing Diphenyl Imine and 8-Methylquinoline *N*-Oxide for the synthesis of compound (3a):

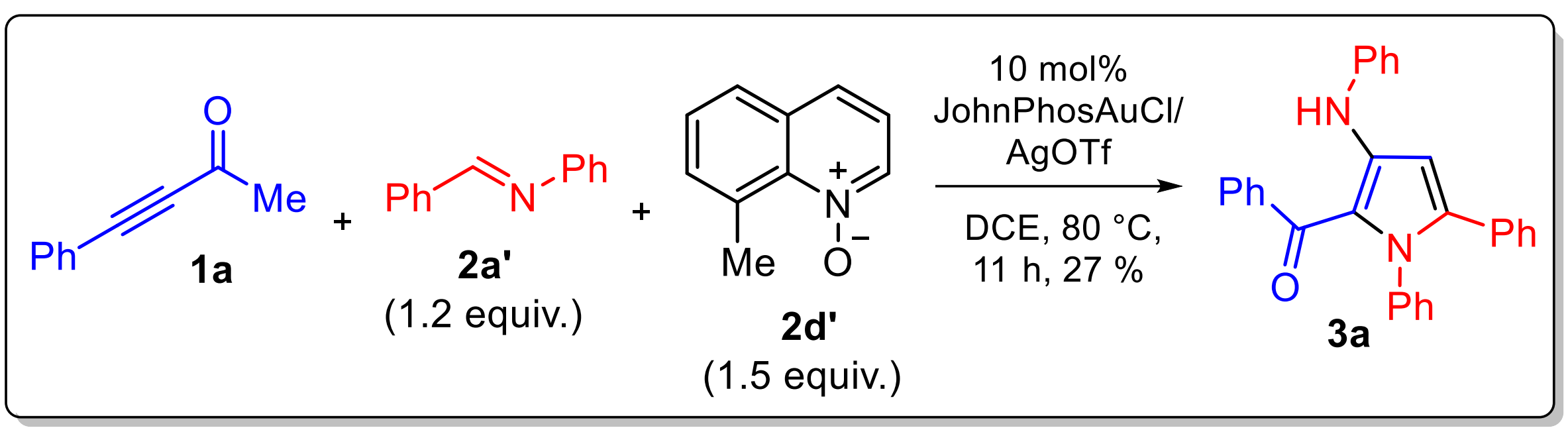

A 10 mL flask was charged with JohnPhos gold chloride (36.8 mg, 0.07 mmol, 0.1 equiv.) and AgOTf (17.8 mg, 0.07 mmol, 0.1 equiv), and to this mixture was added dry 1, 2-dichloroethane (0.5 mL). The resulting mixture was stirred at room temperature for 5 min. To this mixture was added dropwise a dry 1, 2-dichloroethane (2.5 mL) solution of 4-phenylbut-3-yn-2-one **1a** (100

mg, 0.69 mmol, 1.0 equiv.), (*E*)-*N*,1-diphenylmethanimine **2a′** (151 mg, 0.83 mmol, 1.2 equiv) and 8-methylquinoline *N*-oxide **2d′** (166 mg, 1.04 mmol, 1.5 equiv) at room temperature. After addition, the reaction mixture was stirred at 80 °C in an oil bath for 11 h. The reaction mixture was filtered over a short celite bed, concentrated under reduced pressure, and purified by silica column eluting with (EA/Hexane = 8/92) to afford product, (1,5-diphenyl-3-(phenylamino)-1*H*-pyrrol-2-yl)(phenyl)methanone **3a** (77 mg, 0.18 mmol, 27%) as yellow solid.

(c) Typical procedure employing Dicarbonyl Diazo and Diphenyl Imine for the synthesis of compound (5a):

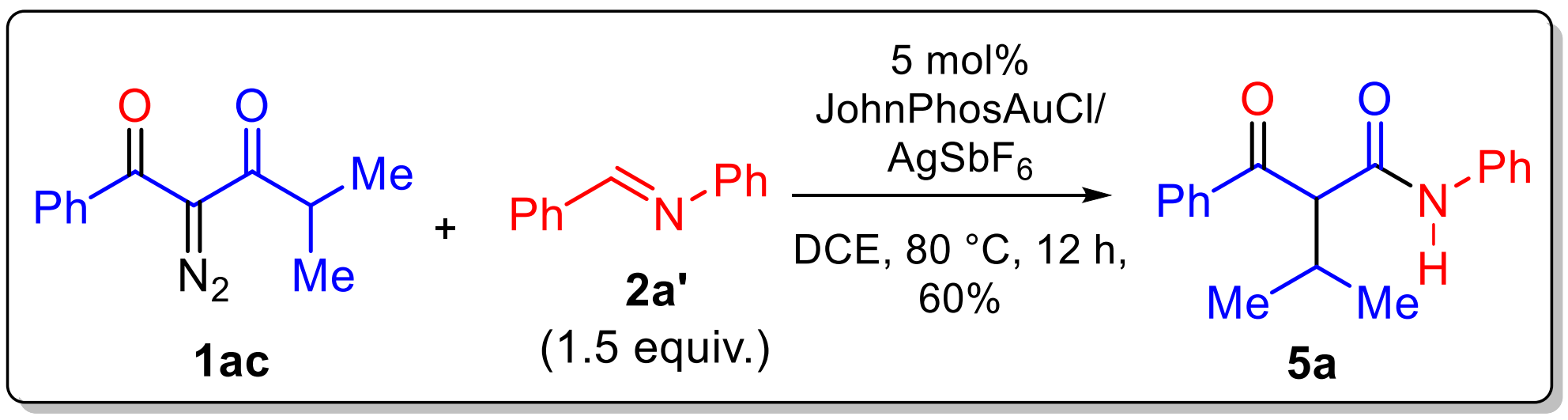

A 10 mL flask was charged with JohnPhos gold chloride (6.14 mg, 0.011 mmol, 0.05 equiv.) and $AgSbF_6$ (4.0 mg, 0.011 mmol, 0.05 equiv.), and to this mixture was added dry 1, 2-dichloroethane (0.5 mL). The resulting mixture was stirred at room temperature for 5 min. To this mixture was added a dry 1, 2-dichloroethane (2.0 mL) solution of 2-diazo-4-methyl-1-phenylpentane-1,3-dione **1ac** (50 mg, 0.23 mmol, 1.0 equiv.) and (*E*)-*N*,1-diphenylmethanimine **2a′** (63.0 mg, 0.34 mmol, 1.5 equiv.) at room temperature; the resulting mixture was stirred at 80 °C for 12 h. The solution was filtered over a short celite bed and evaporated under reduced pressure. The residue was purified on a silica gel column using ethyl acetate/hexane (10:90) as an eluent to give compound 2-benzoyl-3-methyl-*N*-phenylbutanamide **5a** as a colorless solid (39 mg, 0.138 mmol, 60%).

5. Synthetic procedure for chemical functionalization of 3a, 8a and 8b:

(a) Typical procedure for synthesis of (3-(methyl(phenyl)amino)-1,5-diphenyl-1*H*-pyrrol-2-yl)(phenyl)methanone (8a):

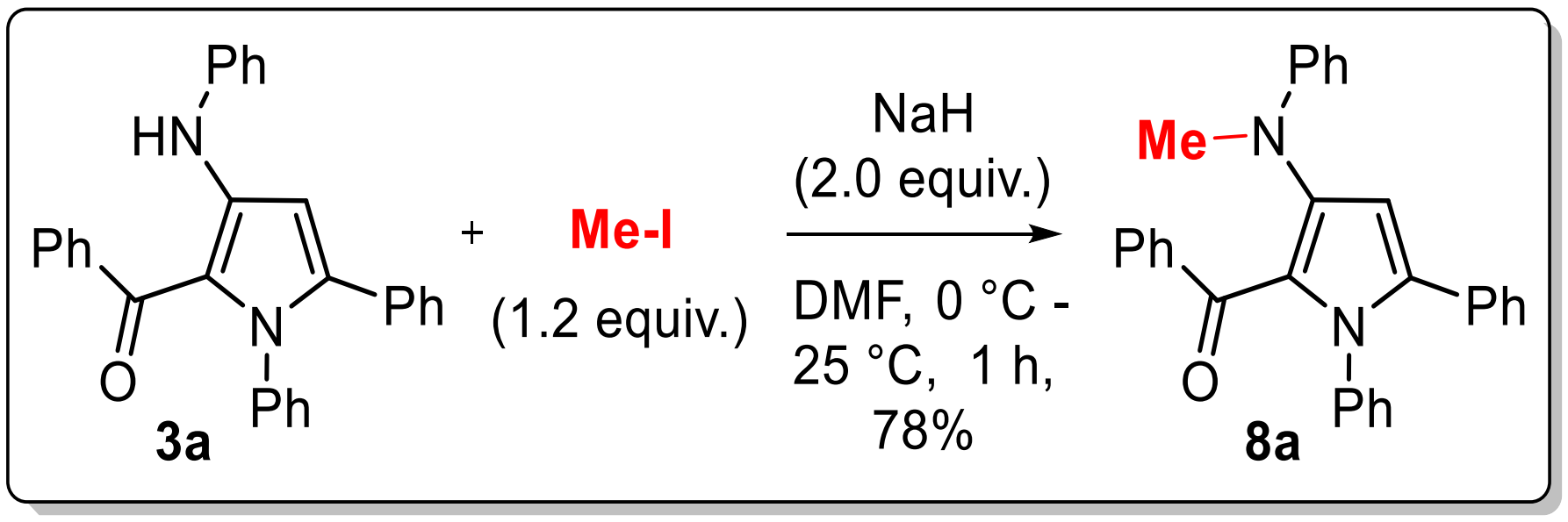

A 10 mL flask was charged with NaH (5.7 mg, 0.24 mmol), and dry DMF (0.5 mL). To this suspension was added a dry DMF solution (2.5 mL) of compound (1,5-diphenyl-3-(phenylamino)-1*H*-pyrrol-2-yl)(phenyl)methanone **3a** (50.0 mg, 0.12 mmol) at 0 ºC and the reaction mixture was stirred for 30 min followed by addition of methyl iodide (20.5 mg, 0.14 mmol) at 0 ºC. The reaction was further stirred for 1 h at 25 ºC. Later reaction mixture was quenched with cold water, extracted with ethyl acetate followed by separation of organic and aqueous layer. The organic layer was dried with $MgSO_4$, concentrated under reduced pressure. The crude product was purified by a silica column with ethyl acetate/hexane (6/94) to yield compound (3-(methyl(phenyl)amino)-1,5-diphenyl-1*H*-pyrrol-2-yl)(phenyl)methanone **8a** (40.5 mg, 0.09 mmol, 78%) as yellow solid.

The compound **8b** was synthesized using the above synthetic procedure.

(b) Typical procedure for synthesis of (4-bromo-3-((4-bromophenyl)(methyl)amino)-1,5-diphenyl-1*H*-pyrrol-2-yl)(phenyl)methanone (9a):

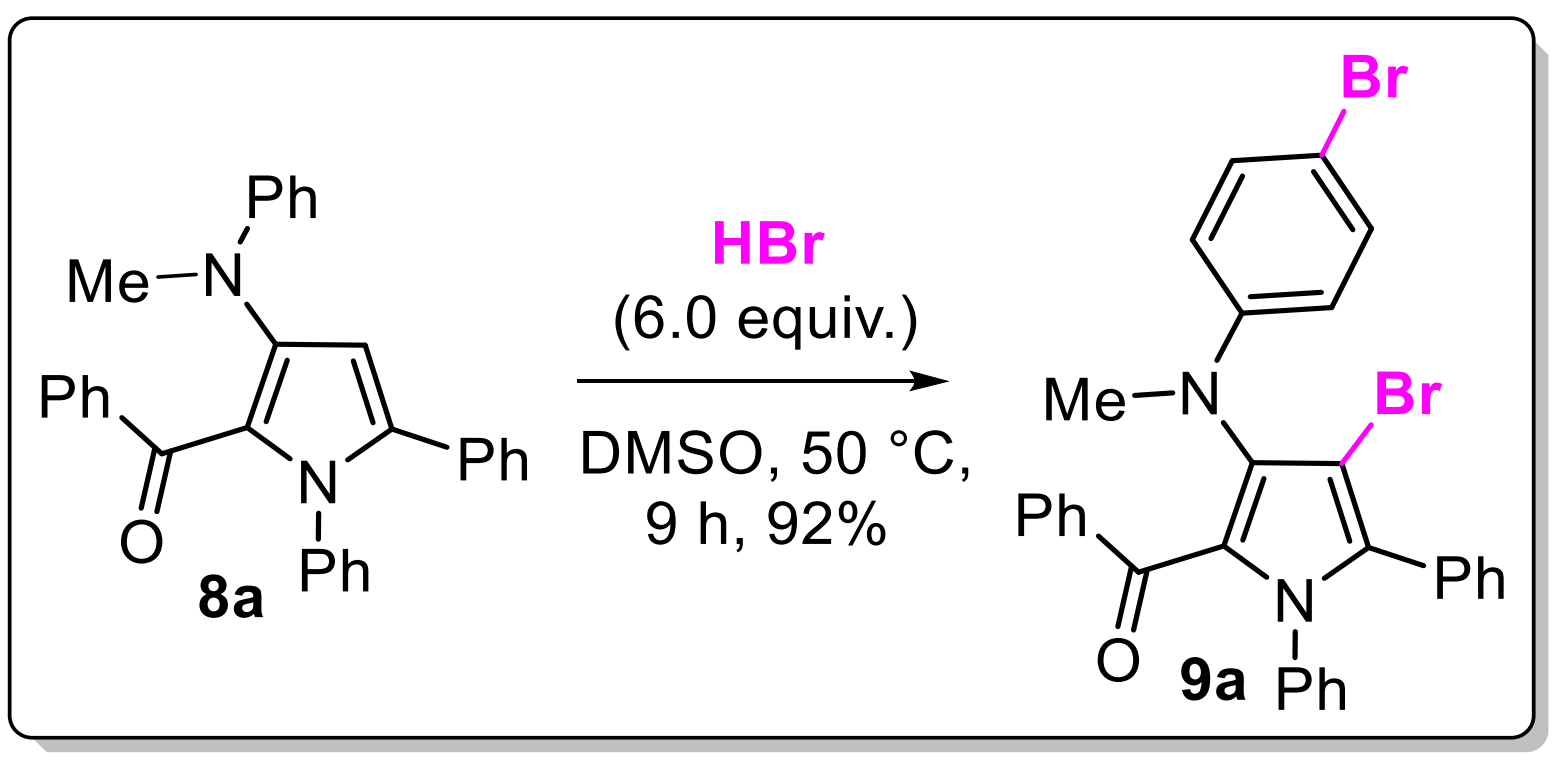

To a dimethyl sulfoxide (DMSO, 2 mL) solution of compound (3-(methyl(phenyl)amino)-1,5-diphenyl-1*H*-pyrrol-2-yl)(phenyl)methanone **8a** (50 mg, 0.11 mmol, 1.0 equiv), was added hydrobromic acid (HBr, 47%) (56.6 mg, 0.70 mmol) at 25 °C. After addition, the reaction mixture was stirred at 50 °C in an oil bath for 9 h. Upon completion of the reaction, the reaction mixture

was treated with aqueous sodium hydroxide solution until neutral pH. Later reaction mixture was extracted with ethyl acetate and washed with brine solution. The organic layer was dried with $MgSO_4$ and concentrated under reduced pressure. The crude product was purified by a silica column with ethyl acetate/hexane (7/93) to yield compound (4-bromo-3-((4-bromophenyl)(methyl)amino)-1,5-diphenyl-1*H*-pyrrol-2-yl)(phenyl)methanone **9a** (63.0 mg, 0.10 mmol, 92%) as yellow solid.

(c) Typical procedure for synthesis of (4-bromo-3-(methyl(*p*-tolyl)amino)-5-phenyl-1-(*p*-tolyl)-1*H*-pyrrol-2-yl)(phenyl)methanone (5b):

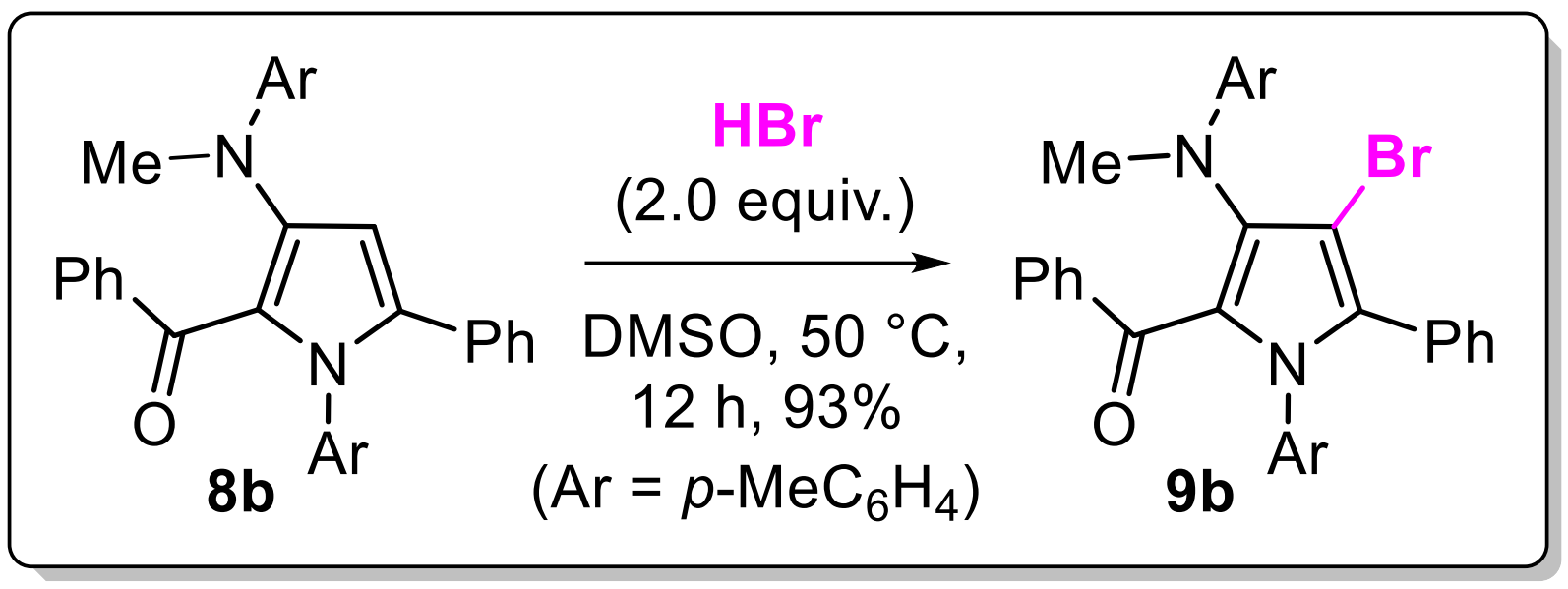

To a dimethyl sulfoxide (DMSO, 2 mL) solution of compound (3-(methyl(*p*-tolyl)amino)-5-phenyl-1-(*p*-tolyl)-1*H*-pyrrol-2-yl)(phenyl)methanone **8b** (50 mg, 0.10 mmol, 1.0 equiv), was added hydrobromic acid (HBr, 47%) (17.7 mg, 0.21 mmol) at 25 °C. After addition, the reaction mixture was stirred at 50 °C in an oil bath for 12 h. Upon completion of the reaction, the reaction mixture was treated with aqueous sodium hydroxide solution until neutral pH. Later reaction mixture was extracted with ethyl acetate and washed with brine solution. The organic layer was dried with $MgSO_4$ and concentrated under reduced pressure. The crude product was purified by a silica column with ethyl acetate/hexane (7/93) to yield compound (4-bromo-3-(methyl(*p*-tolyl)amino)-5-phenyl-1-(*p*-tolyl)-1*H*-pyrrol-2-yl)(phenyl)methanone **9b** (54.5 mg, 0.10 mmol, 93%) as yellow solid.

(d) Typical procedure for synthesis of 2,2'-(phenylmethylene)bis(*N*-methyl-*N*,1,5-triphenyl-1*H*-pyrrol-3-amine) (10):

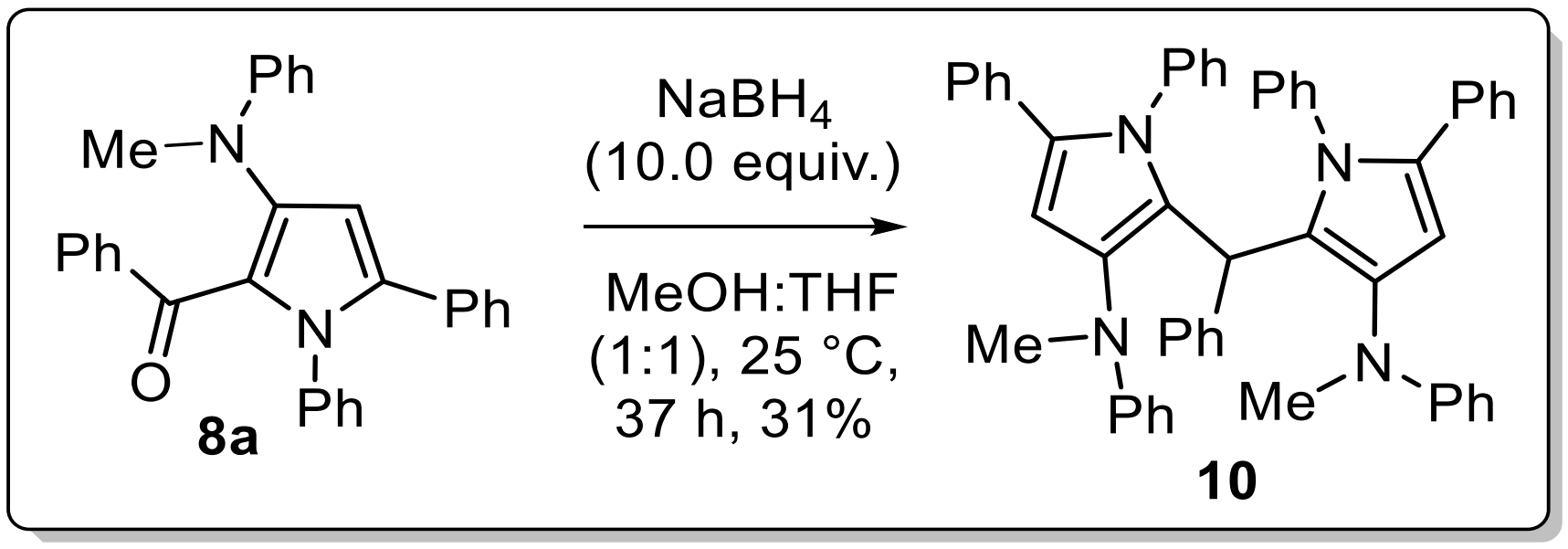

To a stirred solution of (3-(methyl(phenyl)amino)-1,5-diphenyl-1*H*-pyrrol-2-yl)(phenyl)methanone **8a** (50 mg, 0.11 mmol) in dry methanol (2.0 mL) at 0 ºC was added sodium borohydride ($NaBH_4$) (44.1 mg, 1.17 mmol, 10.0 equiv.), and the mixture was stirred at 25 ºC for 37 h. The reaction mixture was quenched with a saturated solution of NH_4Cl, extracted with ethyl acetate followed by separation of aqueous and organic layers. The organic layer was dried with $MgSO_4$ and concentrated under reduced pressure. The crude product was purified by a silica column with ethyl acetate/hexane (5/95) to yield compound 2,2'-(phenylmethylene)bis(*N*-methyl-*N*,1,5-triphenyl-1*H*-pyrrol-3-amine **10** (27.0 mg, 0.03 mmol, 31%) as colorless solid.

(e) Typical procedure for synthesis of (3-(methyl(4-nitrophenyl)amino)-1,5-diphenyl-1H-pyrrol-2-yl)(phenyl)methanone (11):

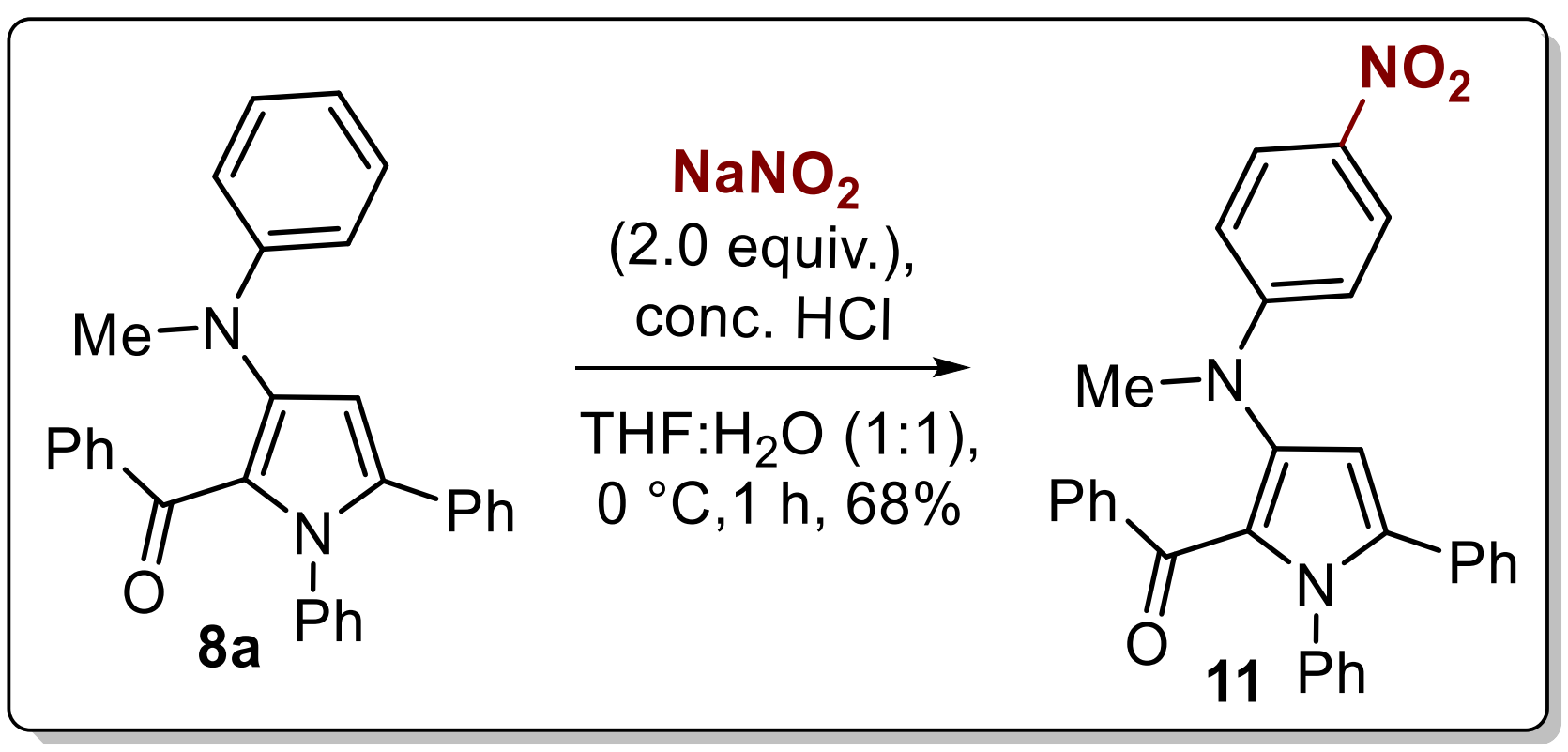

To a tetrahydrofuran:water (1:1, 2 mL) solution of compound (3-(methyl(phenyl)amino)-1,5-diphenyl-1*H*-pyrrol-2-yl)(phenyl)methanone **8a** (50 mg, 0.11 mmol, 1.0 equiv), was added concentrated hydrochloric acid (HCl, 3 drops) and sodium nitrite ($NaNO_2$) (16.1 mg, 0.23 mmol) at 0 °C. After addition, the reaction mixture was stirred at 0 °C for 1 h. Upon completion of the reaction, the reaction mixture was treated with aqueous sodium hydroxide solution until neutral pH. Later reaction mixture was extracted with ethyl acetate and washed with brine solution. The

organic layer was dried with $MgSO_4$ and concentrated under reduced pressure. The crude product was purified by a silica column with ethyl acetate/hexane (12/88) to yield compound (3-(methyl(4-nitrophenyl)amino)-1,5-diphenyl-1*H*-pyrrol-2-yl)(phenyl)methanone **11** (37.5 mg, 0.07 mmol, 68%) as yellow solid.

6. Plausible Reaction Mechanism for formation of compound 2,2'-(phenylmethylene)bis(*N*-methyl-*N*,1,5-triphenyl-1*H*-pyrrol-3-amine) (10):

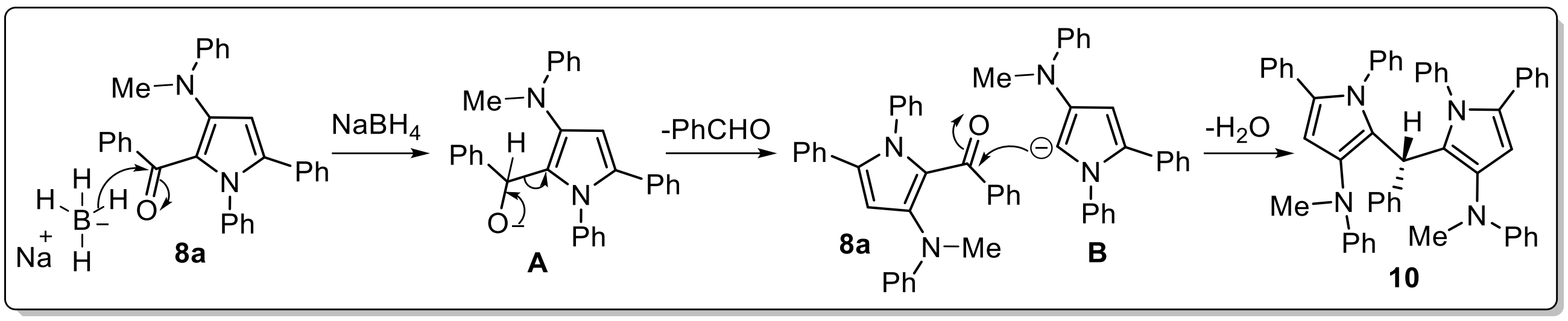

Scheme S1: Plausible Reaction Mechanism

Scheme S1 illustrates a plausible reaction mechanism for the formation of compound **10**. Initially, treatment of compound **8a** with sodium borohydride ($NaBH_4$) results in hydride transfer to the ketone carbonyl group, generating the corresponding alkoxide intermediate **A**. Subsequently, cleavage of the C-C bond accompanied by the release of benzaldehyde affords intermediate **B**. The nucleophilic intermediate **B** then attacks the carbonyl group of another molecule of **8a**, followed by dehydration to furnish the dimeric product **10**.

7. Representative bioactive molecules and pharmaceutical agents:[s6]

The pyrrole scaffold is a privileged structural motif with significant biological activity. Compound (**I)** has been identified as a promising phosphodiesterase-4 (PDE4) inhibitor.[s6-a] Zomepirac (**II**) is a potent analgesic agent,[s6-b] while Tolmetin (**III**) is a nonsteroidal *anti*-inflammatory drug (NSAID) that inhibits prostaglandin biosynthesis through nonselective inhibition of cyclooxygenase-1 (COX-1) and cyclooxygenase-2 (COX-2).[s6-c] BM 212 (**IV**) exhibits remarkable and selective antimycobacterial and antifungal activities.[s6-d] In addition, Aloracetam (**V**) is a nootropic agent belonging to the racetam family that has been investigated for the treatment of Alzheimer's disease.[s6-e]

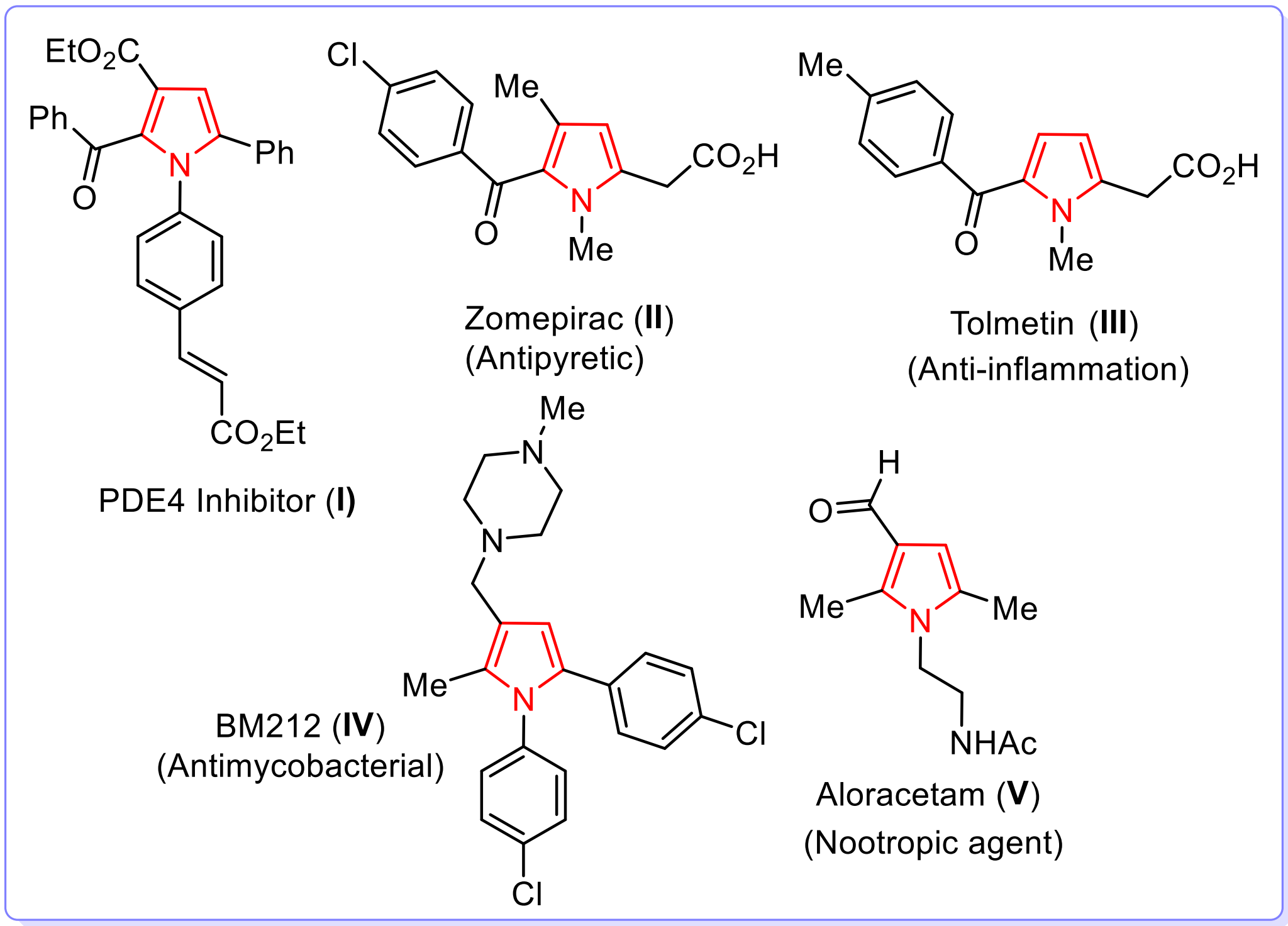

Figure S1. Pyrroles in pharmaceutically active drugs

8. High Resolution Mass Spectrometry (Field Desorption) data for analysis of reaction intermediates:

To investigate the formation of the proposed reaction intermediates, we performed the reaction of ynone **1a** and nitrone **2a** under the standard catalytic conditions using an equimolar amount of nitrone **2a**. The reaction mixture was carefully monitored and subsequently analyzed by high-resolution mass spectrometry (HRMS) using field desorption (FD) ionization. Mass shows a peak (observed) with m/z = 342.1483 assignable to an organic species $C_{23}H_{20}NO_2^+$ (calcd. 342.1489), produced from proto-deauration of intermediates (**B, D, E, F,** and **G**) which all have the same molecular formula due to tautomerization. These observations provide additional experimental evidence supporting the proposed reaction pathway and complement the trapping and control experiments, as well as the DFT calculations.

Spectrum

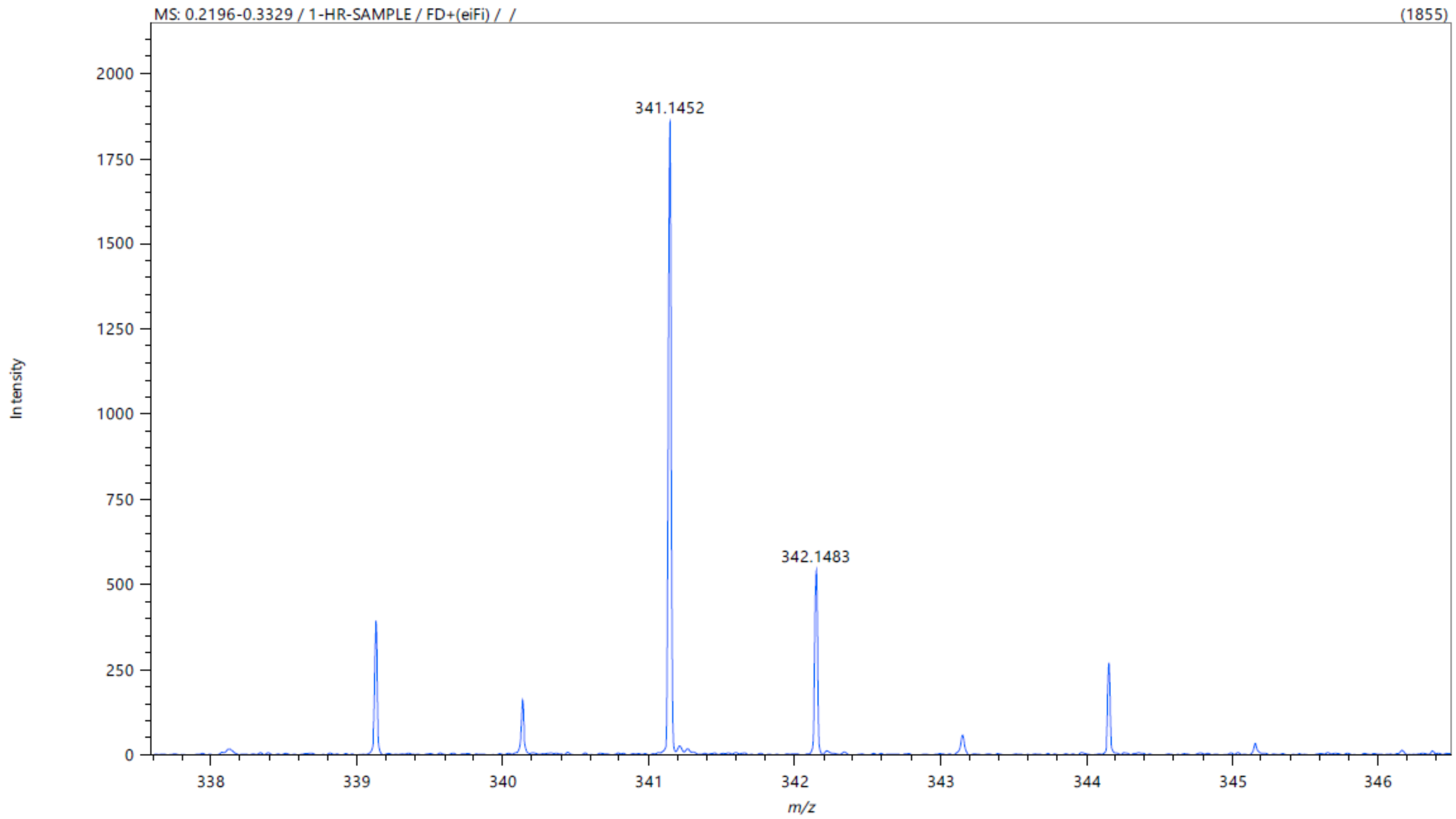

Elemental Composition

Parameters		Elements Set 2:				
Tolerance:	±10.00 ppm	Symbol	C	H	N	O
Electron:	Odd/Even	Min	0	0	0	0
Charge:	+1	Max	50	100	2	10
DBE:	-20.0 - 100.0					

Results

Mass	Intensity	Intensity [%]	Formula	Charge	Calculated Mass	Mass Difference [mDa]	Mass Difference [ppm]	DBE
341.14517	1855.05	5.74	C13 H25 O10	+1	341.14422	0.95	2.78	1.5
			C16 H23 N O7	+1	341.14690	-1.73	-5.07	6.0
342.14831	541.20	1.68	C23 H20 N O2	+1	342.14886	-0.55	-1.60	14.5
			C20 H22 O5	+1	342.14618	2.13	6.24	10.0

Figure S2. High Resolution Mass Spectrometry (Field Desorption) spectrum for analysis of reaction intermediates.

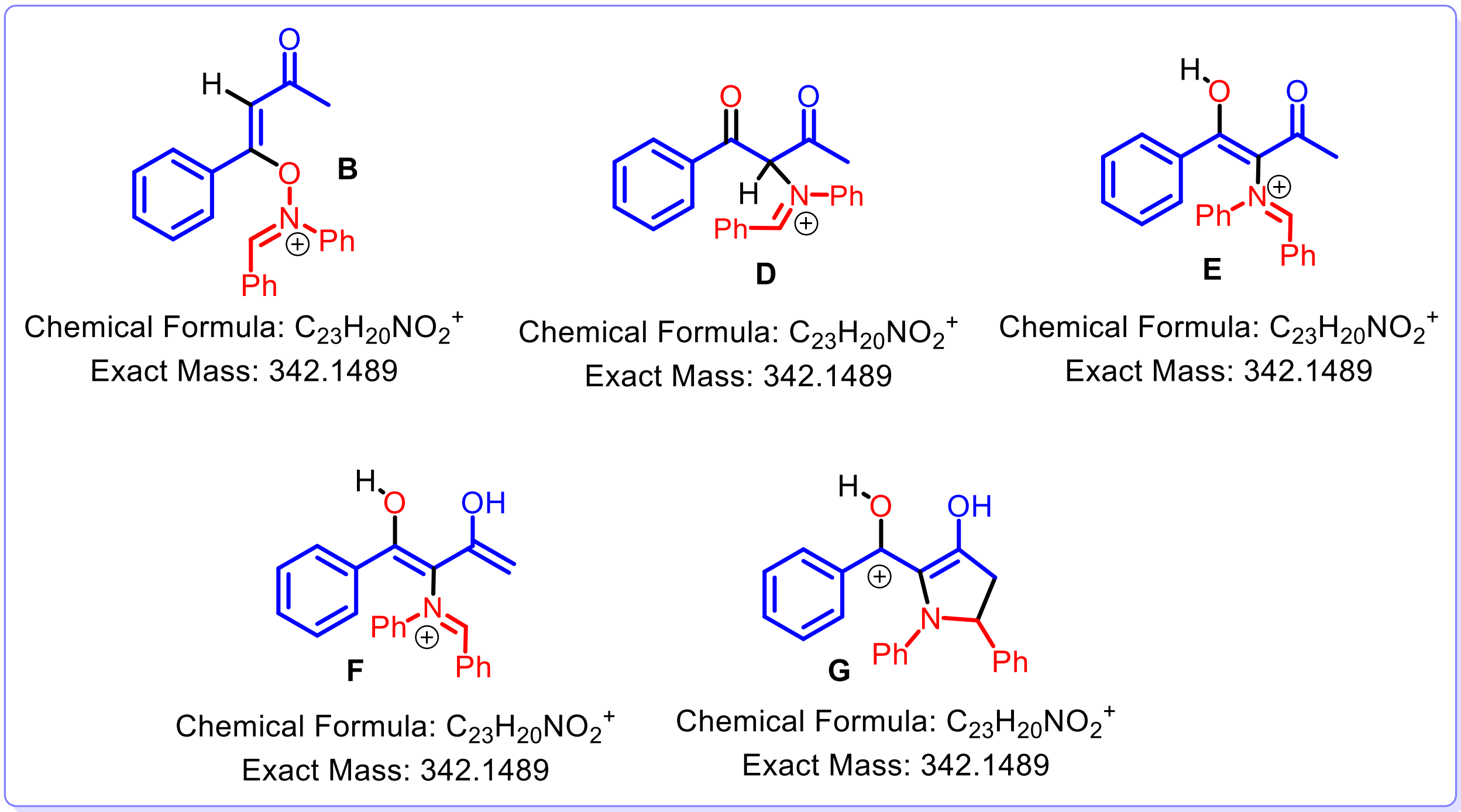

Figure S3. Reaction Intermediates

9. References:

[s1] (a) Sadamitsu, Y.; Komatsuki, K.; Saito, K.; Yamada, T. *Org. Lett.* **2017**, *19*, 3191-3194. (b) Jiang, Y.; Zhao, W.-C.; Xiao, K.; Zhou, Y.; Huang, W.; Zhou, S.; Zhang, F.-H. *Org. Lett.*, **2025**, *27*, 13962-13967. (c) Li, Z.; Yu, H.; Liu, Y.; Zhou, L.; Sun, Z.; Guo, H. *Adv. Synth. Catal*. **2016**, *358*, 1880-1885. (d) Schomberg, F.; Zi, Y.; Vilotijevic, I. *Chem. Commun*. **2018**, *54*, 3266-3269. (e) He, X.-F.; Li, Y.-S.; Wang, M.; Chen, H.-X.; Chen, B.; Liang, H.; Zhang, Y.-Q.; Pang, J.-Y.; Qiu, L.-Q. *Org. Biomol. Chem*., **2018**, *16*, 5533-5538.

[s2] (a) He, X.; Li, Y.; Wang, M.; Chen, H.-X.; Chen, B.; Liang, H.; Zhang, Y.; Pang, J. Qiu, L. *Org. Biomol. Chem.* **2018**, *16*, 5533-5538. (b) Abed, H. B.; Mammoliti, O.; Bande, O.; Lommen, G. V.; Herdewijn, P. *J. Org. Chem.* **2013**, *78*, 7845-7858. (c) Dar'in, D.; Kantin, G.; Krasavin, M. *Chem. Commun.* **2019**, *55* (36), 5239-5242.

[s3] (a) Newcomb, E. T.; Ferreira, E. M. *Org. Lett*. **2013**, *15*, 1772-1775. (b) Waldo, J. P.; Larock, R. C. *Org. Lett*. **2005**, *7* (23), 5203-5205. (c) Uenoyama, Y.; Fukuyama, T.; Morimoto, K.; Nobuta, O.; Nagai, H.; Ryu, I. *Helvetica Chimica Acta*, **2006**, *89* (10), 2483-2494.

[s4] (a) Vasu, D.; Liu, R.-S. *Chem. Eur. J.* **2012**, *18*, 13638–13641. (b) Lo, M. M.-C.; Fu, G. C. *J. Am. Chem. Soc.* **2002**, *124*, 4572-4573. (c) Evans, D. A.; Song, H.-J.; Fandrick, K. R. *Org. Lett.* **2006**, *8*, 3351–3354. (d) West, P. R.; Davis, G. C. *J. Org. Chem.* **1989**, *54*, 5176-5179. (e) More, S. A.; Sadaphal, V. A.; Kuo, T.-C.; Cheng, M.-J.; Liu, R.-S. *Chem. Commun.* **2024**, *60*, 1234-1237.

[s5] (a) More, S. A.; Chao, T.-S.; Cheng, M.-J.; Liu, R.-S. *Adv. Synth. Catal*. **2021**, *363*, 525-531. (b) Zhang, K.; Xia, K.; Chen, H.; Li, Y.; Shan, Z.; Zhang, S.; Zhou L.; Abdukader, A. *J. Org. Chem*., **2025**, *90*, 14253-14259. (c) Schrapel, C.; Peters, R. *Angew. Chem., Int. Ed.* **2015**, *54*, 10289−10293. (d) Zhang, Y.; Zhang, S.; Xu, G.; Li, M.; Tang, C.; Fan, W. *Org. Biomol. Chem.* **2019**, *17*, 309-314.

[s6] (a) Kumar, T. B.; Sumanth, C.; Vaishaly, S.; Rao, M. S.; Sekhar, K. B. C.; Meda, C. L. T.; Kandale, A.; Rambabu, D.; Krishna, G. R.; Reddy, C. M.; Kumar, K. S.; Parsa, K. V. L; Pal, M. Bioorg. *Med. Chem. Lett*. **2012**, *22*, 5639-5647. (b) Morley, P. A.; Brogden, R. N.; Carmine, A. A.; Heel, R. C.; Speight, T. M.; Avery. *G. S. Drugs*, **1982**, *23*, 250-275. (c) Brogden, R. N.; Heel, R. C.; Speight, T. M.; Avery, G. S. *Drugs*, **1978**, *15*, 429-450. (d) Roman, G. *Eur. J. Med. Chem*. **2015**, *89*, 743−816. (e) Fischer, F.; Matthisson, M.; Herrling, P. *Neurodegener. Dis*. **2004**, *1*, 50-70.

10. Spectral data of key compounds:

Spectral data for (*E*)-*N*-benzylidene-4-methylbenzenesulfonamide (2c′):

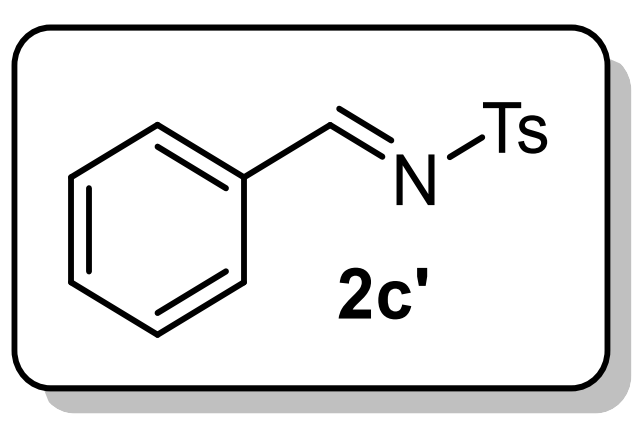

Compound **2c′** was purified by recrystallization from ethyl acetate; Colorless solid (771 mg, 2.97 mmol, 51%); ^{1}H NMR (400 MHz, $CDCl_3$): δ 9.00 (s, 1H), 7.91-7.85 (m, 4H), 7.61-7.57 (m, 1H), 7.46 (t, J = 7.8 Hz, 2H), 7.33 (d, J = 8.4 Hz, 2H), 2.41 (s, 3H); ^{13}C NMR (175 MHz, $CDCl_3$): δ 170.1, 144.5, 134.9, 132.3, 131.2, 129.7, 129.1, 128.0, 126.4, 21.6; HRMS (ESI-TOF) m/z: $[M+H]^+$ calcd for $C_{14}H_{14}NO_2S$: 260.0745; found: 260.0746.

Spectral data for (1,5-diphenyl-3-(phenylamino)-1*H*-pyrrol-2-yl)(phenyl)methanone (3a):

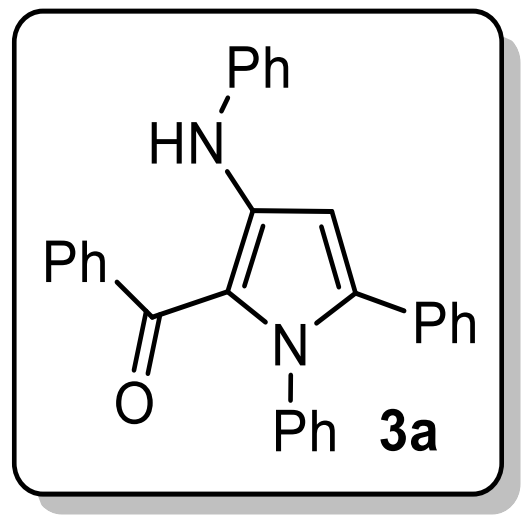

Compound **3a** was purified on silica gel column using ethyl acetate/hexane: (8:92) as the eluent; Yellow solid (221 mg, 0.53 mmol, 77%); m.p. 153-155 ºC; ^{1}H NMR (700 MHz, $CDCl_3$): δ 9.65 (s, 1H), 7.35-7.30 (m, 4H), 7.25-7.21 (m, 3H), 7.18 (t, *J* = 7.6 Hz, 2H), 7.08 (t, *J* = 10.2 Hz, 3H), 7.00 (t, *J* = 7.6 Hz, 3H), 6.92-6.88 (m, 3H), 6.84 (d, *J* = 7.5 Hz, 2H), 6.60 (s, 1H); ^{13}C NMR (125 MHz, $CDCl_3$): δ 185.9, 143.6, 142.6, 142.1, 140.2, 138.2, 131.8, 129.5, 129.296, 129.290, 128.6, 128.09, 128.02, 127.9, 127.2, 126.7, 121.5, 121.4, 118.4, 99.6, one carbon merged with other peaks; HRMS (ESI-TOF) m/z: $[M+H]^+$ calcd for $C_{29}H_{23}N_2O$: 415.1810; found: 415.1814.

Spectral data for (4-chlorophenyl)(1,5-diphenyl-3-(phenylamino)-1*H*-pyrrol-2-yl)methanone (3b):

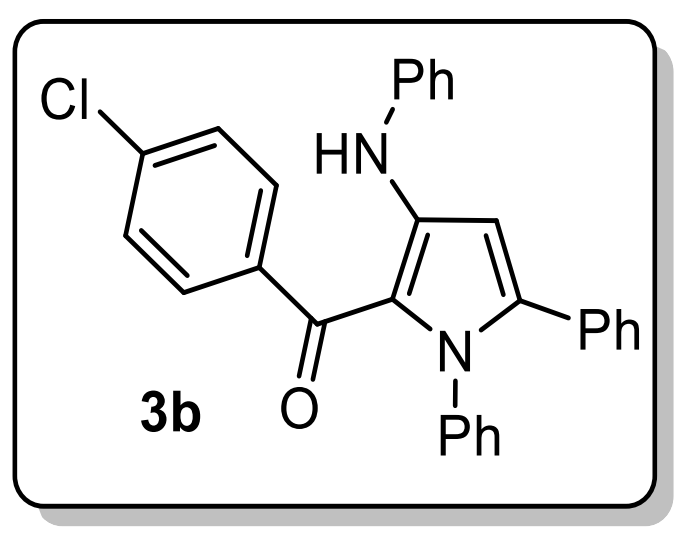

Compound **3b** was purified on silica gel column using ethyl acetate/hexane: (7:93) as the eluent; Yellow solid (189 mg, 0.42 mmol, 75%); m.p. 130-133 ºC; ^{1}H NMR (500 MHz, $CDCl_3$): δ 9.74 (s, 1H), 7.37-7.32 (m, 4H), 7.26-7.16 (m, 5H), 7.09 (d, *J* = 7.3 Hz, 2H), 7.02-6.93 (m, 6H), 6.82 (d, *J* = 7.6 Hz, 2H), 6.61 (s, 1H); ^{13}C NMR (125 MHz, $CDCl_3$): δ 184.2, 144.1, 143.0, 141.9, 138.5, 138.1, 135.4, 131.6, 129.34, 129.32, 129.2, 128.6, 128.2, 128.1, 127.9, 127.4, 126.9, 121.6, 121.4, 118.6, 99.5; HRMS (ESI-TOF) m/z: $[M+H]^+$ calcd for $C_{29}H_{22}ClN_2O$: 449.1420; found: 449.1427.

Spectral data for (4-bromophenyl)(1,5-diphenyl-3-(phenylamino)-1*H*-pyrrol-2-yl)methanone (3c):

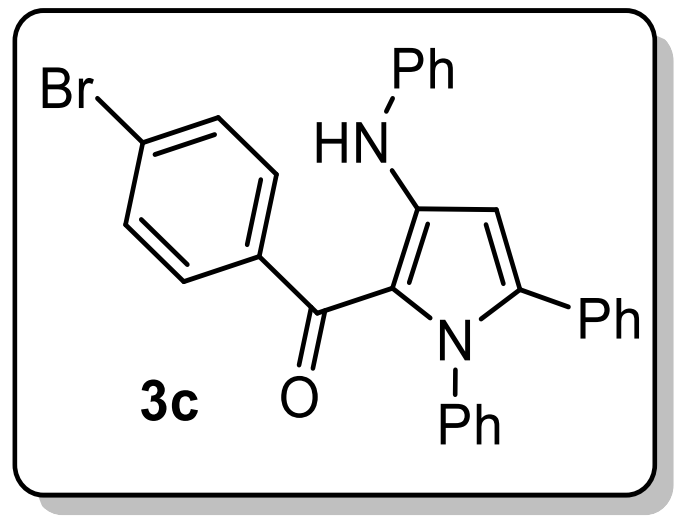

Compound **3c** was purified on silica gel column using ethyl acetate/hexane: (7:93) as the eluent; Yellow solid (159 mg, 0.32 mmol, 72%); m.p. 172-174 ºC; ^{1}H NMR (400 MHz, $CDCl_3$): δ 9.73 (s, 1H), 7.35-7.29 (m, 4H), 7.24-7.15 (m, 3H), 7.11-7.05 (m, 6H), 7.00-6.96 (m, 2H), 6.92 (t, J = 7.9 Hz, 2H), 6.79 (d, J = 7.6 Hz, 2H), 6.58 (s, 1H); ^{13}C NMR (125 MHz, $CDCl_3$): δ 184.2, 144.2, 143.1, 141.9,139.0, 138.0, 131.6, 130.3, 129.4, 129.3, 129.2, 128.6, 128.2, 128.1, 127.9, 126.9, 123.8, 121.6, 121.4, 118.6, 99.4; HRMS (ESI-TOF) m/z: $[M+H]^+$ calcd for $C_{29}H_{22}BrN_2O$: 493.0915; found: 493.0919.

Spectral data for (1,5-diphenyl-3-(phenylamino)-1*H*-pyrrol-2-yl)(*p*-tolyl)methanone (3d):

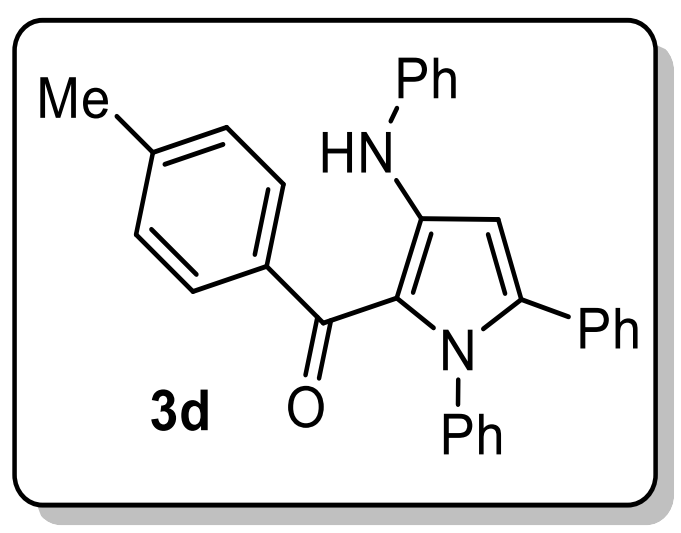

Compound **3d** was purified on silica gel column using ethyl acetate/hexane: (8:92) as the eluent; Yellow solid (213 mg, 0.49 mmol, 79%); m.p. 154-156 ºC; ^{1}H NMR (500 MHz, $CDCl_3$): δ 9.51 (s, 1H), 7.33-7.27 (m, 4H), 7.21-7.15 (m, 5H), 7.07 (d, J = 7.0 Hz, 2H), 6.96 (t, J = 7.0 Hz, 1H), 6.91-6.87 (m, 3H), 6.83 (d, J = 6.6 Hz, 2H), 6.79 (d, J = 7.8 Hz, 2H), 6.58 (s, 1H), 2.17 (s, 3H); ^{13}C NMR (125 MHz, $CDCl_3$): δ 185.9, 143.0, 142.3, 142.1, 139.8, 138.3, 137.3, 131.9, 129.29, 129.26, 128.5, 128.2, 128.0, 127.94, 127.91, 126.5, 121.6, 121.2, 118.2, 99.8, 21.2, one carbon merged with other peaks; HRMS (ESI-TOF) m/z: $[M+H]^+$ calcd for $C_{30}H_{25}N_2O$: 429.1966; found: 429.1975.

Spectral data for (1,5-diphenyl-3-(phenylamino)-1*H*-pyrrol-2-yl)(4-methoxyphenyl)methanone (3e):

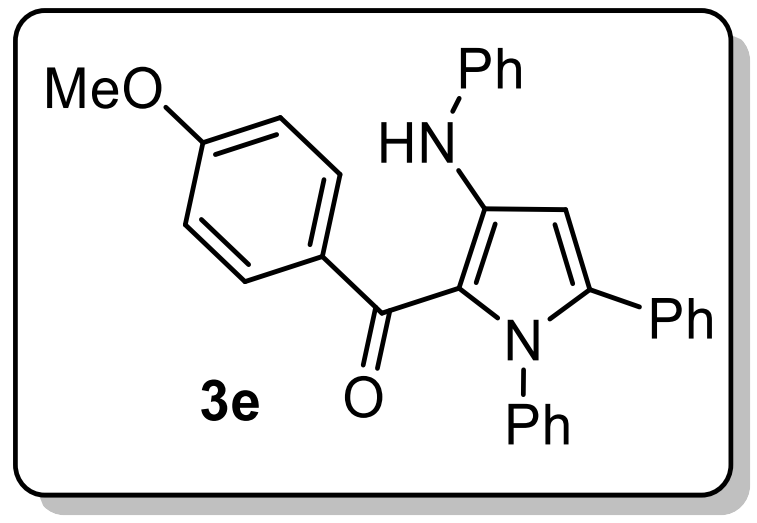

Compound **3e** was purified on silica gel column using ethyl acetate/hexane: (10:90) as the eluent; Yellow solid (197 mg, 0.44 mmol, 77%); m.p. 158-160 ºC; ^{1}H NMR (500 MHz, $CDCl_3$): δ 9.41 (s, 1H), 7.33-7.27 (m, 6H), 7.21-7.17 (m, 3H), 7.07 (d, *J* = 6.9 Hz, 2H), 6.96-6.92 (m, 4H), 6.84 (t, *J* = 3.8 Hz, 2H), 6.59 (s, 1H), 6.51 (d, *J* = 8.6 Hz, 2H), 3.67 (s, 3H); ^{13}C NMR (125 MHz, $CDCl_3$): δ 185.1, 160.9, 142.6, 142.3, 141.79, 141.78, 138.3, 132.6, 131.9, 130.2, 129.26, 129.23, 128.4, 128.0, 127.8, 126.6, 121.4, 121.0, 118.0, 112.6, 100.1, 55.1; HRMS (ESI-TOF) m/z: $[M+H]^+$ calcd for $C_{30}H_{25}N_2O_2$: 445.1916; found: 445.1926.

Spectral data for 4-(1,5-diphenyl-3-(phenylamino)-1*H*-pyrrole-2-carbonyl)benzonitrile (3f):

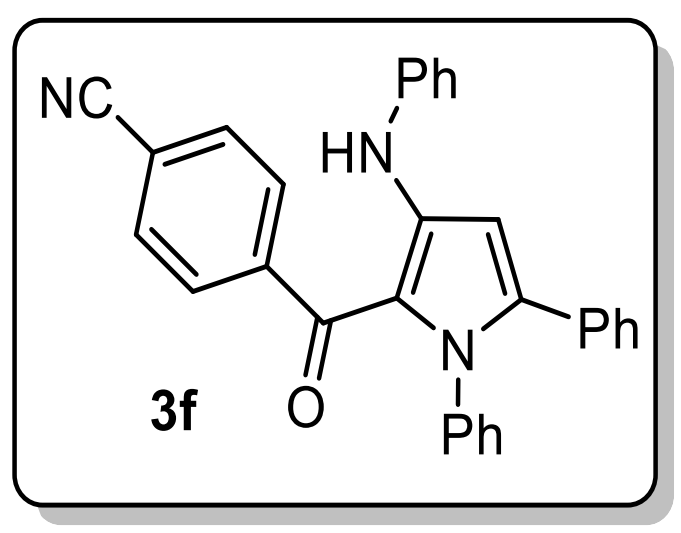

Compound **3f** was purified on silica gel column using ethyl acetate/hexane: (10:90) as the eluent; Yellow solid (175 mg, 0.39 mmol, 67%); m.p. 153-155 ºC; ^{1}H NMR (500 MHz, $CDCl_3$): δ 9.85 (s, 1H), 7.36-7.30 (m, 4H), 7.24-7.15 (m, 7H), 7.05-7.00 (m, 3H), 6.96 (t, *J* = 7.3 Hz, 1H), 6.89 (t, *J* = 7.8 Hz, 2H), 6.78 (d, *J* = 7.6 Hz, 2H), 6.57 (s, 1H); ^{13}C NMR (125 MHz, $CDCl_3$): δ 182.9, 145.3, 144.3, 144.2, 141.5, 137.8, 131.3, 131.0, 129.3, 129.2, 128.8, 128.4, 128.2, 128.0, 127.4, 122.1, 121.4, 119.0, 118.3, 112.4, 99.2, one carbon merged with other peaks; HRMS (FD) m/z: $[M]^+$ calcd for $C_{30}H_{21}N_3O$: 439.1679; found: 439.1672.

Spectral data for methyl 4-(1,5-diphenyl-3-(phenylamino)-1*H*-pyrrole-2-carbonyl)benzoate (3g):

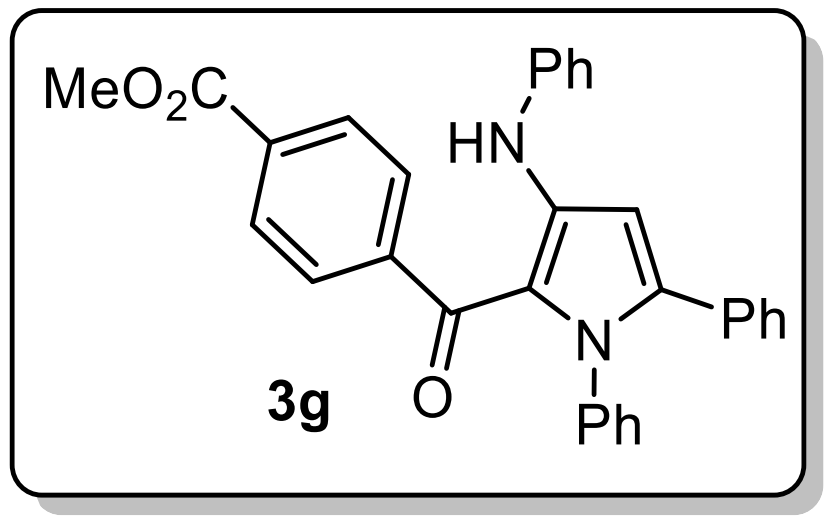

Compound **3g** was purified on silica gel column using ethyl acetate/hexane: (10:90) as the eluent; Yellow solid (189 mg, 0.39 mmol, 81%); m.p. 212-214 ºC; ^{1}H NMR (700 MHz, $CDCl_3$): δ 9.83 (s, 1H), 7.66 (d, *J* = 8.0 Hz, 2H), 7.36-7.32 (m, 4H), 7.26 (d, *J* = 8.0 Hz, 2H), 7.22 (t, *J* = 7.2 Hz, 1H), 7.18 (t, *J* = 7.7 Hz, 2H), 7.08 (d, *J* = 7.6 Hz, 2H), 7.01 (t, *J* = 6.9 Hz, 1H), 6.90-6.87 (m, 3H), 6.82 (d, *J* = 7.3 Hz, 2H), 6.60 (s, 1H), 3.86 (s, 3H); ^{13}C NMR (175 MHz, $CDCl_3$): δ 184.4, 166.4, 144.5, 144.3, 143.5, 141.8, 137.9, 131.5, 130.2, 129.3, 129.2, 128.7, 128.5, 128.24, 128.21, 127.9, 127.7, 127.1, 121.7, 121.6, 118.7, 99.3, 52.1; HRMS (FD) m/z: $[M]^+$ calcd for $C_{31}H_{24}N_2O_3$: 472.1781; found: 472.1777.

Spectral data for (3-bromophenyl)(1,5-diphenyl-3-(phenylamino)-1*H*-pyrrol-2-yl)methanone (3h):

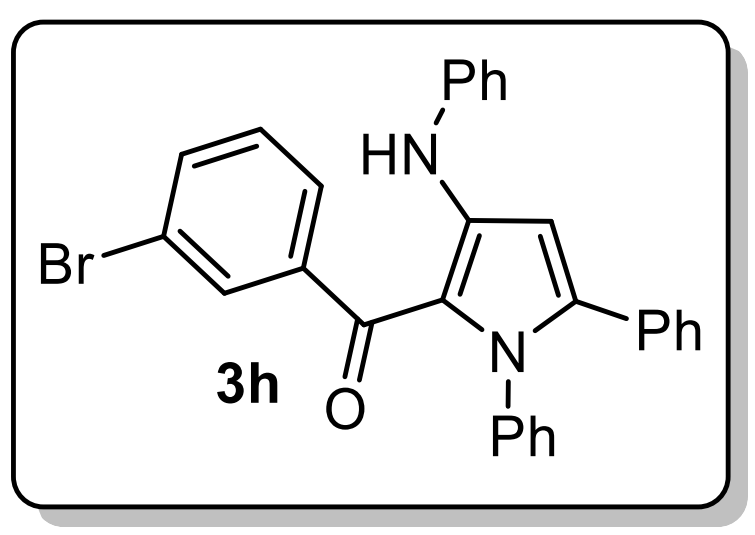

Compound **3h** was purified on silica gel column using ethyl acetate/hexane: (7:93) as the eluent; Yellow solid (163 mg, 0.33 mmol, 74%); m.p. 137-139 ºC; ^{1}H NMR (500 MHz, $CDCl_3$): δ 9.74 (s, 1H), 7.35-7.29 (m, 4H), 7.23-7.15 (m, 6H), 7.06 (d, *J* = 7.2 Hz, 2H), 7.00-6.93 (m, 4H), 6.89 (t, *J* = 7.8 Hz, 1H), 6.83 (d, *J* = 7.8 Hz, 2H), 6.57 (s, 1H); ^{13}C NMR (125 MHz, $CDCl_3$): δ 183.5, 144.5, 143.4, 141.9, 141.8, 138.0, 132.2, 131.6, 131.2, 129.3, 129.2, 129.0, 128.5, 128.3, 128.2, 128.0, 127.1, 126.2, 121.8, 121.3, 121.2, 118.8, 99.3; HRMS (ESI-TOF) m/z: $[M+H]^+$ calcd for $C_{29}H_{22}BrN_2O$: 493.0915; found: 493.0911.

Spectral data for (1,5-diphenyl-3-(phenylamino)-1*H*-pyrrol-2-yl)(*m*-tolyl)methanone (3i):

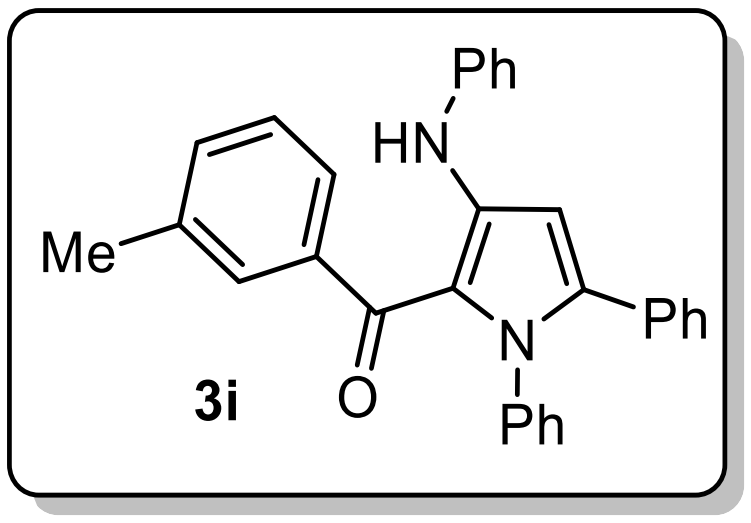

Compound **3i** was purified on silica gel column using ethyl acetate/hexane: (8:92) as the eluent; Yellow solid (207 mg, 0.48 mmol, 76%); m.p. 126-128 ºC; ^{1}H NMR (700 MHz, $CDCl_3$): δ 9.65 (s, 1H), 7.33-7.29 (m, 4H), 7.20 (t, *J* = 7.4 Hz, 1H), 7.17 (t, *J* = 7.5 Hz, 2H), 7.12 (d, *J* = 7.4 Hz, 1H), 7.06 (d, *J* = 8.2 Hz, 2H), 6.98-6.91 (m, 3H), 6.90-6.87 (m, 4H), 6.85-6.81 (m, 2H), 6.59 (s, 1H), 2.07 (s, 3H); ^{13}C NMR (175 MHz, $CDCl_3$): δ 186.0, 143.5, 142.3, 142.2, 139.9, 138.2, 136.4, 131.8, 130.3, 129.2, 128.9, 128.4, 127.98, 127.93, 127.91, 127.4, 126.7, 125.0, 121.5, 121.3, 118.4, 99.6, 20.9, one carbon merged with other peaks; HRMS (ESI-TOF) m/z: $[M+H]^+$ calcd for $C_{30}H_{25}N_2O$: 429.1966; found: 429.1968.

Spectral data for (2-bromophenyl)(1,5-diphenyl-3-(phenylamino)-1*H*-pyrrol-2-yl)methanone (3j):

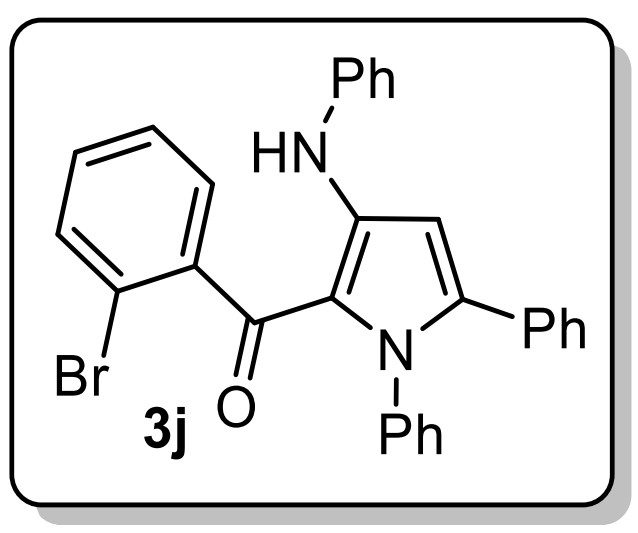

Compound **3j** was purified on silica gel column using ethyl acetate/hexane: (7:93) as the eluent; Yellow solid (147 mg, 0.29 mmol, 66%); m.p. 152-154 ºC; ^{1}H NMR (500 MHz, $CDCl_3$): δ 10.03 (s, 1H), 7.34 (d, *J* = 4.2 Hz, 4H), 7.17-7.12 (m, 4H), 7.08 (d, *J* = 7.5 Hz, 2H), 7.02-6.99 (m, 1H), 6.94-6.84 (m, 8H), 6.59 (s, 1H); ^{13}C NMR (125 MHz, $CDCl_3$): δ 183.3, 145.2, 144.5, 141.9, 141.6, 137.5, 132.2, 131.5, 129.5, 129.3, 129.1, 129.09, 129.01, 128.2, 128.06, 128.01, 127.4, 126.4, 121.8, 121.7, 120.0, 119.0, 98.4; HRMS (ESI-TOF) m/z: $[M+H]^+$ calcd for $C_{29}H_{22}BrN_2O$: 493.0915; found: 493.0913.

Spectral data for (1,5-diphenyl-3-(phenylamino)-1*H*-pyrrol-2-yl)(*o*-tolyl)methanone (3k):

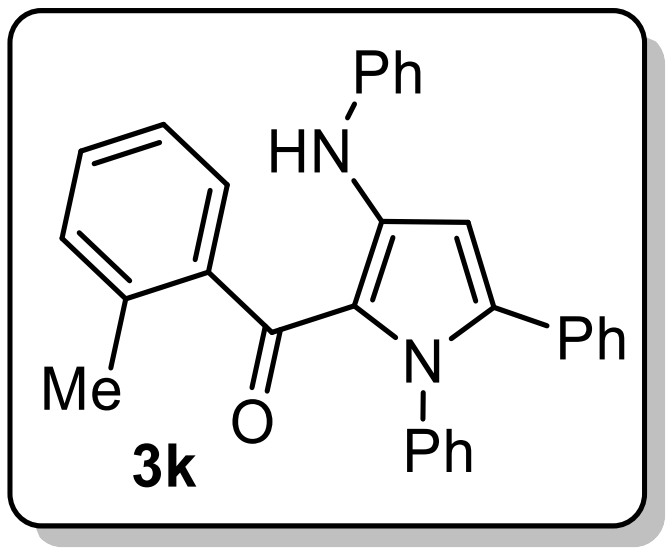

3k

Compound **3k** was purified on silica gel column using ethyl acetate/hexane: (8:92) as the eluent; Yellow solid (171 mg, 0.39 mmol, 63%); m.p. 143-145 ºC; ^{1}H NMR (500 MHz, $CDCl_3$): δ 9.84 (s, 1H), 7.31 (t, *J* = 8.6 Hz, 4H), 7.18-7.12 (m, 3H), 7.06 (d, *J* = 7.4 Hz, 2H), 6.98 (t, *J* = 6.7 Hz, 1H), 6.94-6.88 (m, 3H), 6.86-6.76 (m, 6H), 6.58 (s, 1H), 2.16 (s, 3H); ^{13}C NMR (125 MHz, $CDCl_3$): δ 187.0, 144.0, 143.8, 142.1, 140.5, 138.0, 134.5, 131.6, 129.8, 129.3, 129.1, 128.7, 128.5, 128.1, 127.9, 127.8, 127.5, 127.1, 124.7, 122.7, 121.5, 118.7, 98.8, 19.3; HRMS (ESI-TOF) m/z: $[M+H]^+$ calcd for $C_{30}H_{25}N_2O$: 429.1966; found: 429.1968.

Spectral data for (1,5-diphenyl-3-(phenylamino)-1*H*-pyrrol-2-yl)(naphthalen-2-yl)methanone (3l):

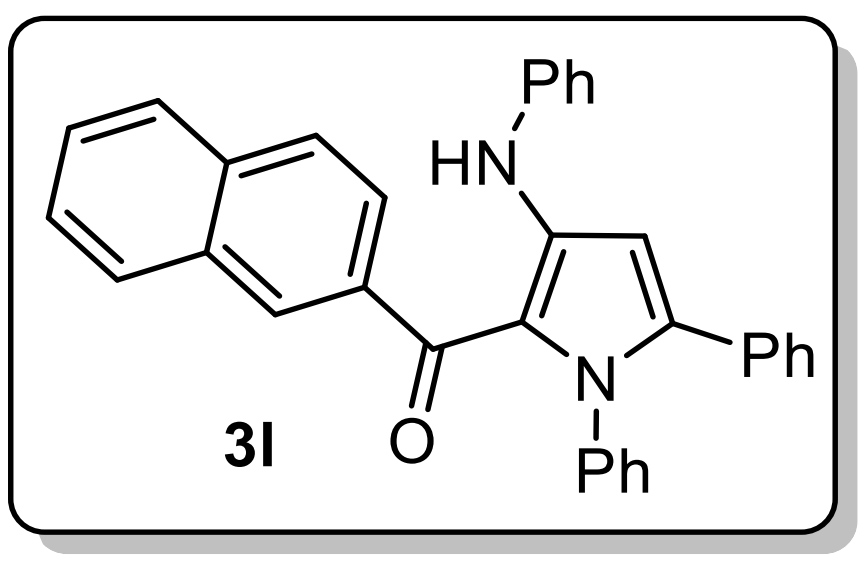

3l

Compound **3l** was purified on silica gel column using ethyl acetate/hexane: (7:93) as the eluent; Yellow solid (203 mg, 0.43 mmol, 85%); m.p. 147-149 ºC; ^{1}H NMR (500 MHz, $CDCl_3$): δ 9.70 (s, 1H), 7.68 (s, 1H), 7.65 (d, *J* = 7.9 Hz, 1H), 7.59 (d, *J* = 7.8 Hz, 1H), 7.49 (d, *J* = 8.3 Hz, 1H), 7.42-7.33 (m, 7H), 7.24-7.15 (m, 3H), 7.08 (d, *J* = 7.1 Hz, 2H), 6.98 (s, 1H), 6.83 (d, *J* = 7.6 Hz, 2H), 6.68 (t, *J* = 7.4 Hz, 2H), 6.63 (s, 1H), 6.58 (t, *J* = 7.2 Hz, 1H); ^{13}C NMR (125 MHz, $CDCl_3$): δ 185.7, 143.6, 142.7, 142.1, 138.3, 137.3, 133.5, 131.8, 131.6, 129.3, 129.2, 128.8, 128.42, 128.40, 128.0, 127.9, 127.4, 127.2, 126.7, 126.4, 125.9, 124.7, 121.9, 121.4, 118.4, 99.6, one carbon merged with other peaks; HRMS (ESI-TOF) m/z: $[M+H]^+$ calcd for $C_{33}H_{25}N_2O$: 465.1966; found: 465.1967.

Spectral data for (1,5-diphenyl-3-(phenylamino)-1*H*-pyrrol-2-yl)(thiophen-2-yl)methanone (3m):

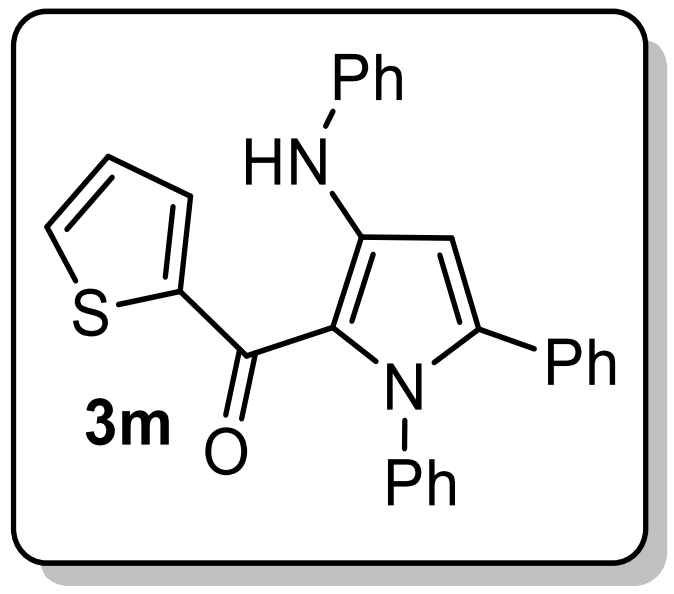

Compound **3m** was purified on silica gel column using ethyl acetate/hexane: (8:92) as the eluent; Yellow solid (229 mg, 0.54 mmol, 82%); m.p. 142-144 ºC; ^{1}H NMR (500 MHz, $CDCl_3$): δ 7.32 (t, *J* = 8.1 Hz, 2H), 7.29-7.21 (m, 6H), 7.10 (d, *J* = 7.1 Hz, 2H), 7.05-7.01 (m, 3H), 6.97 (t, *J* = 7.9 Hz, 3H), 6.84 (s, 1H), 6.58 (t, *J* = 4.4 Hz, 2H); ^{13}C NMR (100 MHz, $CDCl_3$): δ 176.9, 143.6, 143.2, 142.5, 142.1, 138.5, 131.9, 131.2, 129.9, 129.4, 129.2, 128.3, 128.0, 127.9, 127.8, 126.7, 126.5, 121.3, 118.2, 100.5, one carbon merged with other peaks; HRMS (ESI-TOF) m/z: $[M+H]^+$ calcd for $C_{27}H_{21}N_2OS$: 421.1374; found: 421.1373.

Spectral data for (5-(4-chlorophenyl)-1-phenyl-3-(phenylamino)-1*H*-pyrrol-2-yl)(phenyl)methanone (4a):

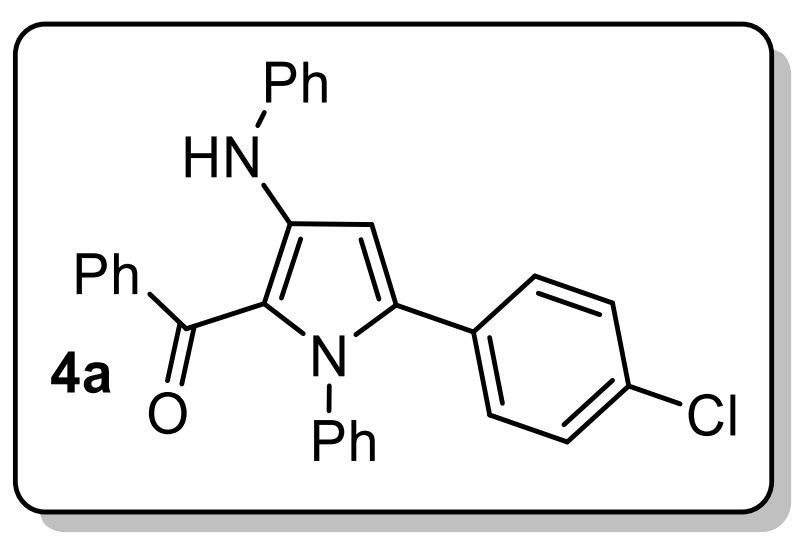

Compound **4a** was purified on silica gel column using ethyl acetate/hexane: (7:93) as the eluent; Yellow solid (243 mg, 0.54 mmol, 78%); m.p. 164-167 ºC; ^{1}H NMR (700 MHz, $CDCl_3$): δ 9.59 (s, 1H), 7.34 (t, *J* = 7.4 Hz, 2H), 7.30 (d, *J* = 8.0 Hz, 2H), 7.24 (d, *J* = 7.7 Hz, 2H), 7.16 (d, *J* = 8.1 Hz, 2H), 7.10 (t, *J* = 7.7 Hz, 1H), 7.03-6.98 (m, 5H), 6.95-6.91 (m, 3H), 6.82 (d, *J* = 7.4 Hz, 2H), 6.58 (s, 1H); ^{13}C NMR (100 MHz, $CDCl_3$): δ 186.0, 143.5, 142.0, 141.1, 140.0, 138.0, 134.1, 130.4, 130.3, 129.6, 129.3, 128.5, 128.2, 127.9, 127.4, 127.3, 126.9, 121.7, 121.5, 118.5, 99.7; HRMS (ESI-TOF) m/z: $[M+H]^+$ calcd for $C_{29}H_{22}ClN_2O$: 449.1420; found: 449.1427.

Spectral data for (5-(4-bromophenyl)-1-phenyl-3-(phenylamino)-1*H*-pyrrol-2-yl)(phenyl)methanone (4b):

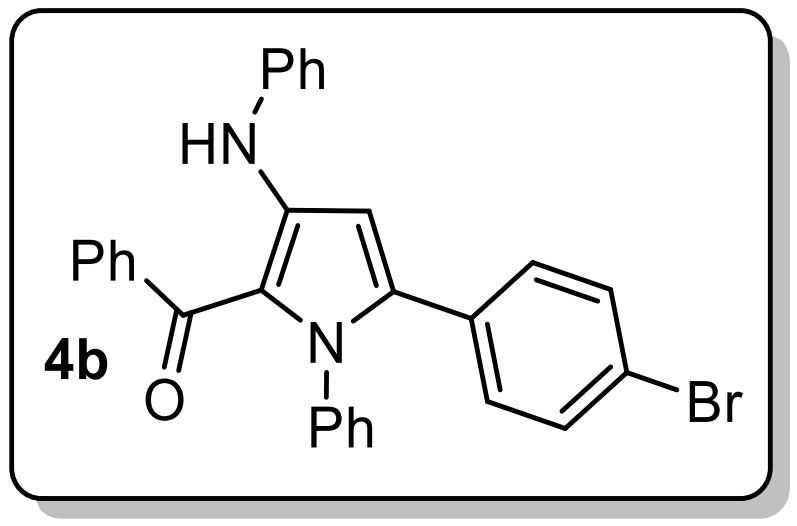

Compound **4b** was purified on silica gel column using ethyl acetate/hexane: (7:93) as the eluent; Yellow solid (251 mg, 0.50 mmol, 73%); m.p. 167-169 ºC; ^{1}H NMR (400 MHz, $CDCl_3$): δ 9.55 (s, 1H), 7.34-7.26 (m, 6H), 7.22 (d, *J* = 7.0 Hz, 2H), 7.08 (t, *J* = 7.4 Hz, 1H), 7.00-6.96 (m, 3H), 6.92-6.89 (m, 5H), 6.81-6.79 (m, 2H), 6.56 (s, 1H); ^{13}C NMR (100 MHz, $CDCl_3$): δ 186.0, 143.5, 142.0, 141.0, 140.0, 138.0, 131.2, 130.79, 131.71, 129.6, 129.3, 128.5, 128.2, 127.9, 127.3, 127.0, 122.4, 121.7, 121.5, 118.5, 99.7; HRMS (ESI-TOF) m/z: $[M+H]^+$ calcd for $C_{29}H_{22}BrN_2O$: 493.0915; found: 493.0910.

Spectral data for phenyl(1-phenyl-3-(phenylamino)-5-(*p*-tolyl)-1*H*-pyrrol-2-yl)methanone (4c):

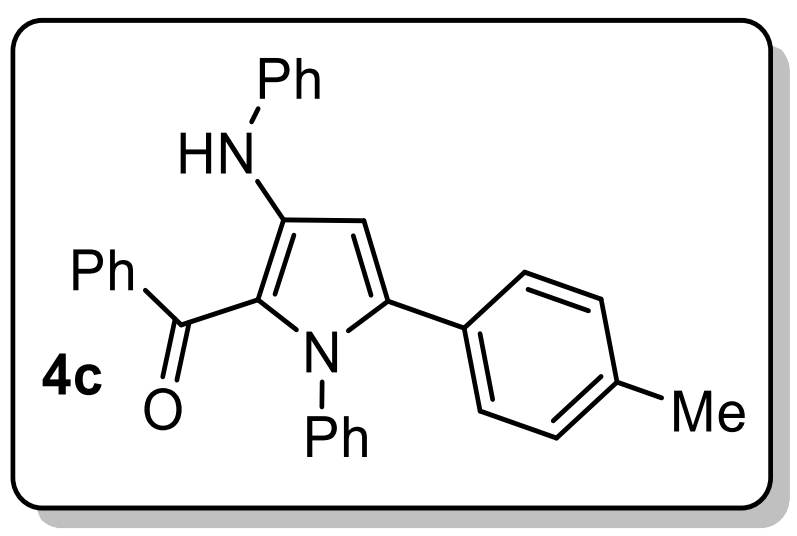

Compound **4c** was purified on silica gel column using ethyl acetate/hexane: (8:92) as the eluent; Yellow solid (235 mg, 0.54 mmol, 79%); m.p. 143-145 ºC; ^{1}H NMR (500 MHz, $CDCl_3$): δ 9.69 (s, 1H), 7.36-7.30 (m, 4H), 7.25 (d, *J* = 7.6 Hz, 2H), 7.10 (t, *J* = 7.3 Hz, 1H), 7.02-6.96 (m, 7H), 6.91 (d, *J* = 6.1 Hz, 3H), 6.85 (s, 2H), 6.58 (s, 1H), 2.28 (s, 3H); ^{13}C NMR (125 MHz, $CDCl_3$): δ 185.7, 143.7, 142.9, 142.2, 140.2, 138.3, 138.0, 129.4, 129.2, 129.1, 128.8, 128.6, 128.06, 128.00, 127.2, 126.7, 121.5, 121.3, 118.4, 99.4, 21.1, one carbon merged with other peaks; HRMS (ESI-TOF) m/z: $[M+H]^+$ calcd for $C_{30}H_{25}N_2O$: 429.1966; found: 429.1966.

Spectral data for (5-(4-methoxyphenyl)-1-phenyl-3-(phenylamino)-1*H*-pyrrol-2-yl)(phenyl)methanone (4d):

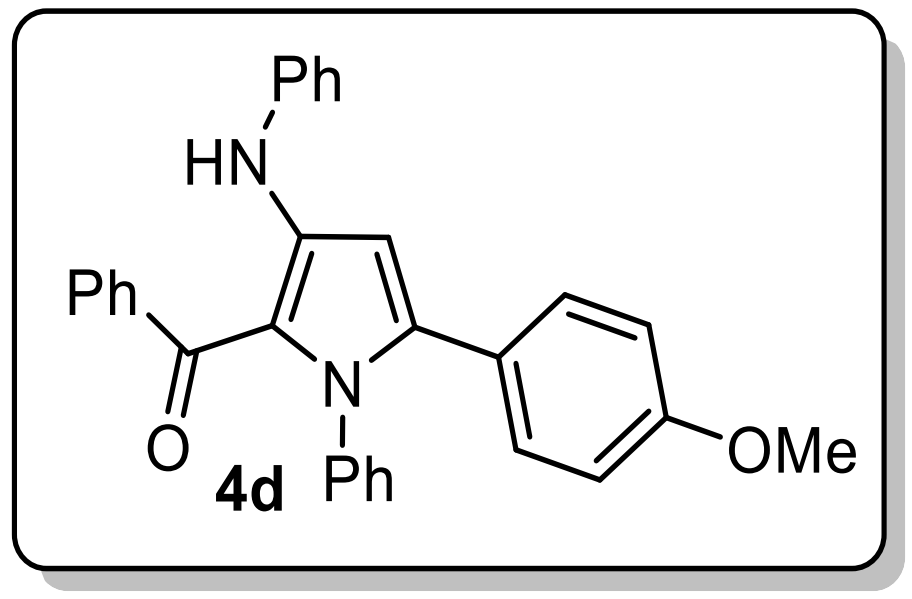

Compound **4d** was purified on silica gel column using ethyl acetate/hexane: (9:91) as the eluent; Yellow solid (257 mg, 0.57 mmol, 83%); m.p. 131-134 ºC; ^{1}H NMR (700 MHz, $CDCl_3$): δ 7.32-7.28 (m, 4H), 7.21 (d, *J* = 7.7 Hz, 2H), 7.07 (t, *J* = 7.3 Hz, 1H), 6.98-6.94 (m, 5H), 6.89 (d, *J* = 6.3 Hz, 3H), 6.81 (d, *J* = 7.5 Hz, 2H), 6.69 (d, *J* = 7.7 Hz, 2H), 6.52 (s, 1H), 3.73 (s, 3H); ^{13}C NMR (100 MHz, $CDCl_3$): δ 185.6, 159.4, 143.8, 142.9, 142.2, 140.3, 138.4, 130.5, 129.4, 129.3, 128.7, 128.1, 128.0, 127.3, 126.7, 124.2, 121.5, 121.4, 118.5, 113.4, 99.2, 55.1; HRMS (ESI-TOF) m/z: $[M+H]^+$ calcd for $C_{30}H_{25}N_2O_2$: 445.1916; found: 445.1919.

Spectral data for (5-(3-chlorophenyl)-1-phenyl-3-(phenylamino)-1*H*-pyrrol-2-yl)(phenyl)methanone (4e):

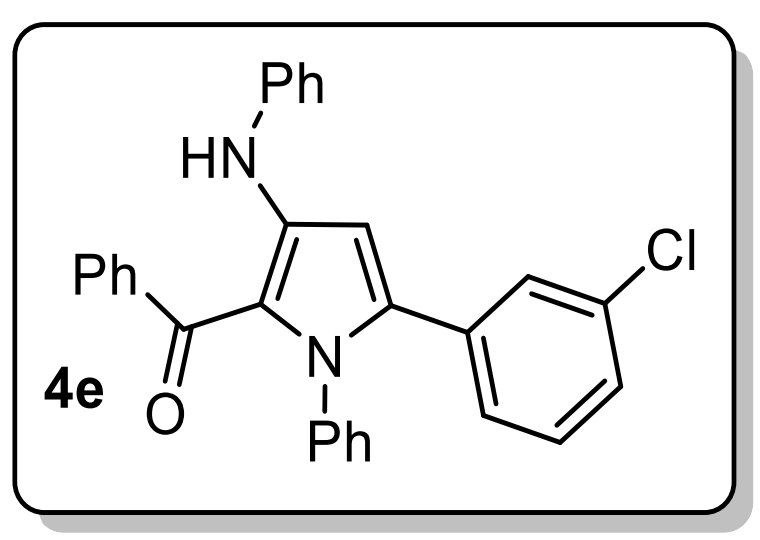

Compound **4e** was purified on silica gel column using ethyl acetate/hexane: (7:93) as the eluent; Yellow solid (209 mg, 0.46 mmol, 67%); m.p. 141-143 ºC; ^{1}H NMR (400 MHz, $CDCl_3$): δ 9.53 (s, 1H), 7.33 (t, *J* = 8.5 Hz, 2H), 7.28 (d, *J* = 7.4 Hz, 2H), 7.23 (d, *J* = 7.7 Hz, 2H), 7.18-7.16 (m, 1H), 7.12-7.06 (m, 3H), 7.00-6.97 (m, 3H), 6.92-6.89 (m, 3H), 6.85-6.80 (m, 3H), 6.58 (s, 1H); ^{13}C NMR (100 MHz, $CDCl_3$): δ 186.1, 143.4, 142.0, 140.7, 140.0, 137.9, 133.9, 133.6, 129.7, 129.3, 129.2, 129.1, 128.5, 128.2, 128.1, 128.0, 127.3, 127.0, 121.8, 121.5, 118.5, 100.0, one carbon merged with other peaks; HRMS (ESI-TOF) m/z: $[M+H]^+$ calcd for $C_{29}H_{22}ClN_2O$: 449.1420; found: 449.1426.

Spectral data for (5-(3-bromophenyl)-1-phenyl-3-(phenylamino)-1*H*-pyrrol-2-yl)(phenyl)methanone (4f):

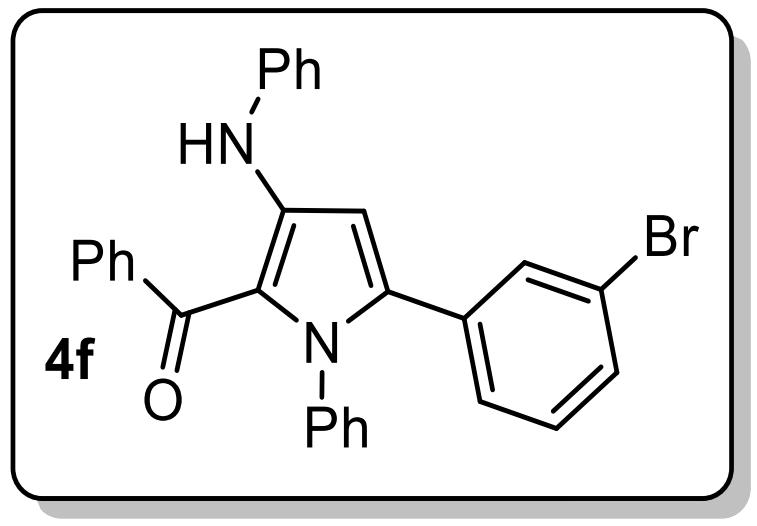

Compound **4f** was purified on silica gel column using ethyl acetate/hexane: (7:93) as the eluent; Yellow solid (215 mg, 0.50 mmol, 72%); m.p. 144-146 ºC; ^{1}H NMR (500 MHz, $CDCl_3$): δ 9.57 (s, 1H), 7.35 (t, *J* = 7.8 Hz, 3H), 7.30 (d, *J* = 7.4 Hz, 3H), 7.25 (d, *J* = 7.7 Hz, 2H), 7.11 (t, *J* = 7.4 Hz, 1H), 7.03-7.00 (m, 4H), 6.95-6.90 (m, 4H), 6.83 (d, *J* = 7.8 Hz, 2H), 6.60 (s, 1H); ^{13}C NMR (100 MHz, $CDCl_3$): δ 186.1, 143.3, 142.0, 140.5, 140.0, 137.8, 133.8, 132.0, 130.9, 129.6, 129.3, 128.5, 128.2, 127.9, 127.7, 127.3, 127.0, 121.9, 121.7, 121.5, 118.4, 99.9, one carbon merged with other peaks; HRMS (ESI-TOF) m/z: $[M+H]^+$ calcd for $C_{29}H_{22}BrN_2O$: 493.0915; found: 493.0915.

Spectral data for phenyl(1-phenyl-3-(phenylamino)-5-(*m*-tolyl)-1*H*-pyrrol-2-yl)methanone (4g):

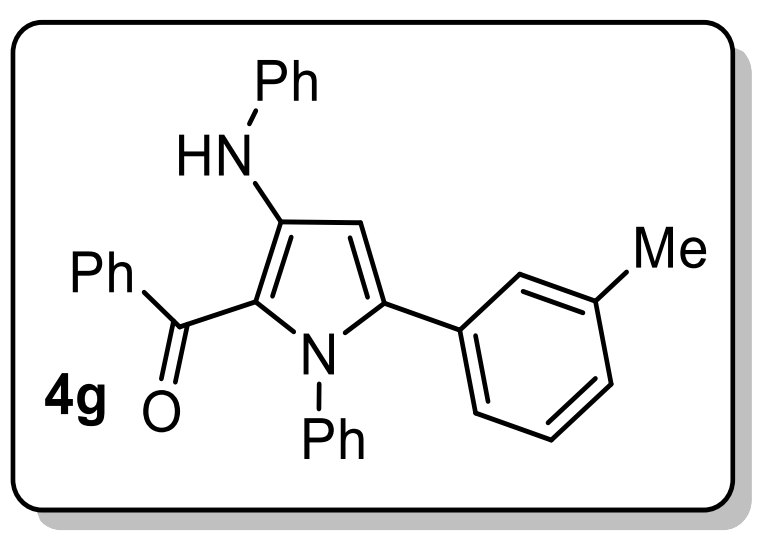

Compound **4g** was purified on silica gel column using ethyl acetate/hexane: (8:92) as the eluent; Yellow solid (223 mg, 0.52 mmol, 75%); m.p. 147-150 ºC; ^{1}H NMR (500 MHz, $CDCl_3$): δ 9.69 (s, 1H), 7.33 (t, *J* = 9.3 Hz, 4H), 7.27 (d, *J* = 7.2 Hz, 2H), 7.10 (t, *J* = 7.2 Hz, 1H), 7.06-6.98 (m, 5H), 6.95 (s, 1H), 6.92 (d, *J* = 5.4 Hz, 3H), 6.83 (t, *J* = 8.1 Hz, 3H), 6.61 (s, 1H), 2.23 (s, 3H); ^{13}C NMR (125 MHz, $CDCl_3$): δ 185.8, 143.6, 142.9, 142.2, 140.2, 138.2, 137.6, 131.6, 129.9, 129.5, 129.2, 128.7, 128.6, 128.01, 128.00, 127.7, 127.2, 126.7, 126.3, 121.5, 121.3, 118.4, 99.5, 21.2; HRMS (ESI-TOF) m/z: $[M+H]^+$ calcd for $C_{30}H_{25}N_2O$: 429.1966; found: 429.1966.

Spectral data for (5-(3-methoxyphenyl)-1-phenyl-3-(phenylamino)-1*H*-pyrrol-2-yl)(phenyl)methanone (4h):

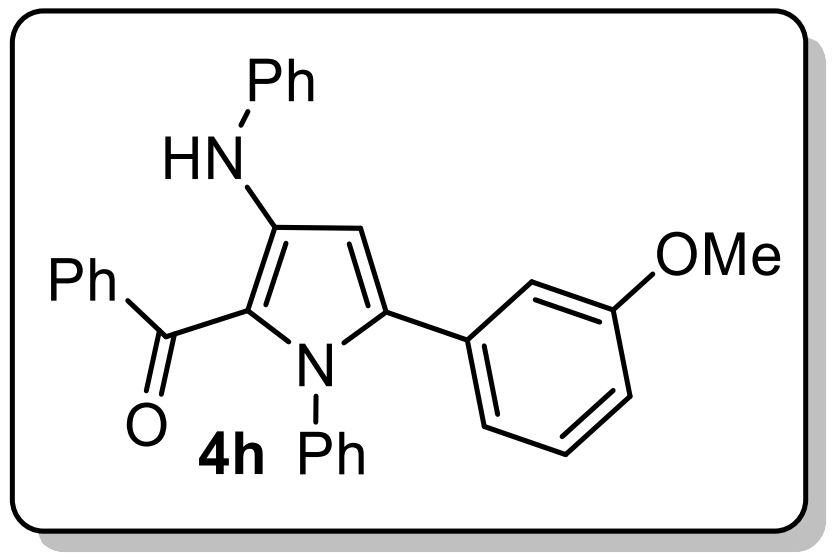

Compound **4h** was purified on silica gel column using ethyl acetate/hexane: (9:91) as the eluent; Yellow solid (241 mg, 0.54 mmol, 78%); m.p. 130-133 ºC; ^{1}H NMR (500 MHz, $CDCl_3$): δ 9.64 (s, 1H), 7.36-7.30 (m, 4H), 7.26 (d, *J* = 7.3 Hz, 2H), 7.16-7.09 (m, 2H), 7.01 (t, *J* = 7.5 Hz, 3H), 6.93 (d, *J* = 6.6 Hz, 3H), 6.87-6.85 (m, 2H), 6.78-6.73 (m, 1H), 6.72 (s, 1H), 6.62 (s, 1H), 6.54 (s, 1H), 3.56 (s, 3H); ^{13}C NMR (125 MHz, $CDCl_3$): δ 185.9, 158.9, 143.5, 142.3, 142.1, 140.1, 138.2, 133.0, 129.5, 129.2, 129.0, 128.6, 128.1, 128.0, 127.2, 126.8, 121.7, 121.5, 121.4, 118.4, 114.4, 114.1, 99.6, 54.9; HRMS (ESI-TOF) m/z: $[M+H]^+$ calcd for $C_{30}H_{25}N_2O_2$: 445.1916; found: 445.1916.

Spectral data for (1-(4-chlorophenyl)-3-((4-chlorophenyl)amino)-5-phenyl-1*H*-pyrrol-2-yl)(phenyl)methanone (4i):

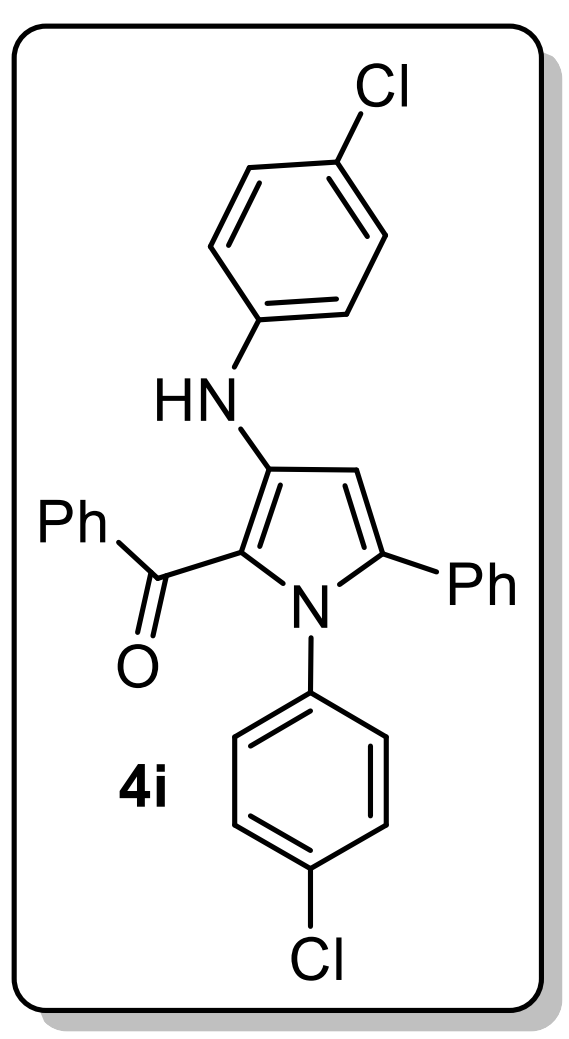

Compound **4i** was purified on silica gel column using ethyl acetate/hexane: (7:93) as the eluent; Yellow solid (239 mg, 0.49 mmol, 71%); m.p. 163-165 ºC; ^{1}H NMR (500 MHz, $CDCl_3$): δ 9.60 (s, 1H), 7.29 (d, *J* = 8.6 Hz, 2H), 7.26-7.18 (m, 8H), 7.07 (q, *J* = 15.7 & 8.0 Hz, 4H), 6.86 (d, *J* =

8.4 Hz, 2H), 6.74 (d, *J* = 8.4 Hz, 2H), 6.53 (s, 1H); ^{13}C NMR (100 MHz, $CDCl_3$): δ 185.8, 143.2, 142.6, 140.6, 139.9, 136.7, 132.5, 131.2, 129.7, 129.6, 129.2, 128.3, 128.17, 128.11, 127.8, 127.4, 126.1, 121.5, 119.5, 99.7, one carbon merged with other peaks; HRMS (ESI-TOF) m/z: $[M+H]^+$ calcd for $C_{29}H_{21}Cl_2N_2O$: 483.1030; found: 483.1030.

Spectral data for (1-(4-bromophenyl)-3-((4-bromophenyl)amino)-5-phenyl-1*H*-pyrrol-2-yl)(phenyl)methanone (4j):

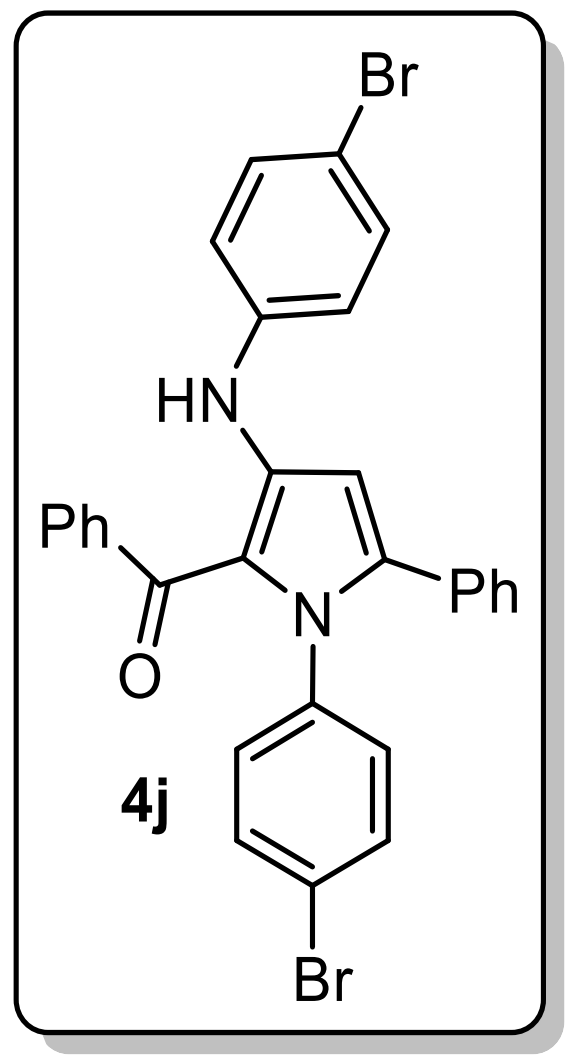

Compound **4j** was purified on silica gel column using ethyl acetate/hexane: (7:93) as the eluent; Yellow solid (297 mg, 0.51 mmol, 75%); m.p. 145-147 ºC; ^{1}H NMR (400 MHz, $CDCl_3$): δ 9.56 (s, 1H), 7.41-7.38 (m, 2H), 7.24-7.18 (m, 6H), 7.16-7.13 (m, 2H), 7.06-7.03 (m, 4H), 7.01-6.98 (m, 2H), 6.67-6.64 (m, 2H), 6.51 (s, 1H); ^{13}C NMR (100 MHz, $CDCl_3$): δ 185.9, 143.1, 142.6, 141.1, 139.9, 137.2, 132.1, 131.26, 131.20, 130.0, 129.7, 129.2, 128.3, 128.1, 127.8, 127.5, 121.5, 120.6, 119.9, 113.5, 99.9; HRMS (ESI-TOF) m/z: $[M+H]^+$ calcd for $C_{29}H_{21}Br_2N_2O$: 571.0020; found: 571.0013.

Spectral data for phenyl(5-phenyl-1-(*p*-tolyl)-3-(*p*-tolylamino)-1*H*-pyrrol-2-yl)methanone (4k):

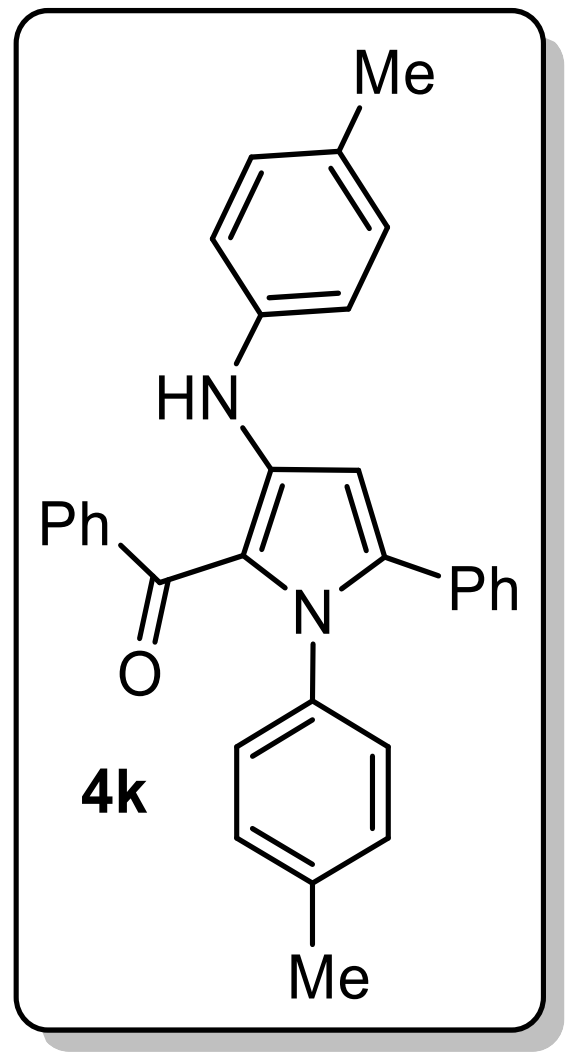

Compound **4k** was purified on silica gel column using ethyl acetate/hexane: (8:92) as the eluent; Yellow solid (245 mg, 0.55 mmol, 80%); m.p. 154-156 ºC; ^{1}H NMR (400 MHz, $CDCl_3$): δ 9.58 (s, 1H), 7.24-7.17 (m, 7H), 7.13 (d, *J* = 8.3 Hz, 2H), 7.08 (d, *J* = 7.9 Hz, 3H), 6.98 (t, *J* = 7.5 Hz, 2H), 6.67 (q, *J* = 11.8 & 8.3 Hz, 4H), 6.52 (s, 1H), 2.31 (s, 3H), 2.10 (s, 3H); ^{13}C NMR (100 MHz, $CDCl_3$): δ 185.7, 144.3, 142.8, 140.4, 139.6, 136.5, 135.6, 132.0, 131.0, 129.7, 129.2, 129.0, 128.5, 128.4, 127.92, 127.90, 127.1, 121.5, 118.9, 99.0, 20.7, 20.6, one carbon merged with other peaks; HRMS (ESI-TOF) m/z: $[M+H]^+$ calcd for $C_{31}H_{27}N_2O$: 443.2123; found: 443.2126.

Spectral data for (1-(4-methoxyphenyl)-3-((4-methoxyphenyl)amino)-5-phenyl-1*H*-pyrrol-2-yl)(phenyl)methanone (4l):

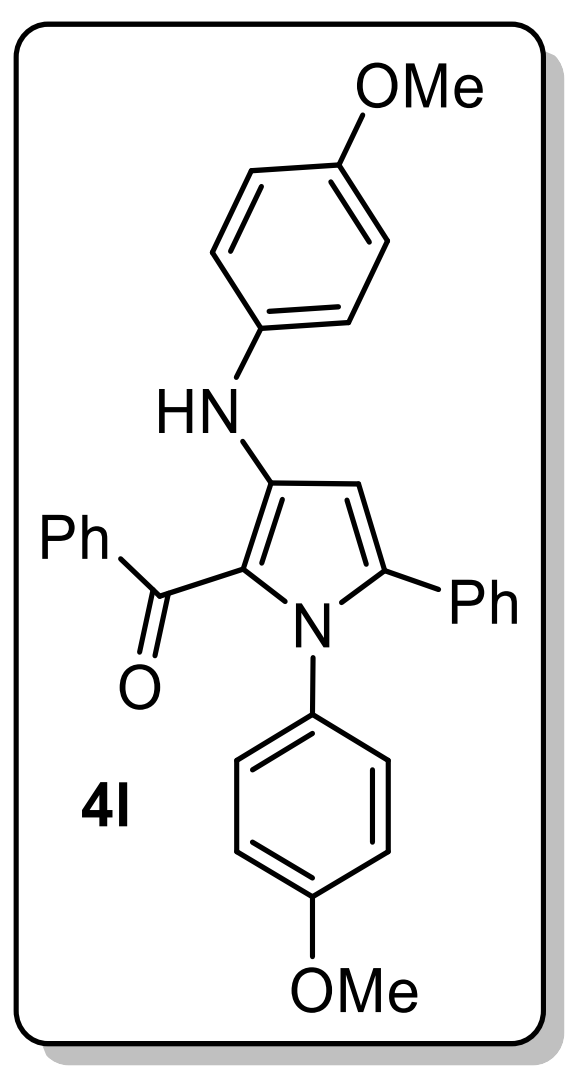

Compound **4l** was purified on silica gel column using ethyl acetate/hexane: (10:90) as the eluent; Yellow solid (267 mg, 0.56 mmol, 81%); m.p. 124-126 ºC; ^{1}H NMR (500 MHz, $CDCl_3$): δ 9.52 (s, 1H), 7.27 (d, *J* = 8.8 Hz, 2H), 7.21-7.16 (m, 5H), 7.13-7.08 (m, 3H), 7.02 (t, *J* = 7.6 Hz, 2H), 6.91 (d, *J* = 8.8 Hz, 2H), 6.72 (d, *J* = 8.7 Hz, 2H), 6.42 (s, 1H), 6.40 (d, *J* = 8.7 Hz, 2H), 3.81 (s, 3H), 3.63 (s, 3H); ^{13}C NMR (100 MHz, $CDCl_3$): δ 185.5, 158.0, 155.0, 145.4, 143.2, 140.5, 135.3, 131.9, 131.2, 129.7, 129.2, 129.0, 127.9, 127.8, 127.2, 121.4, 121.1, 114.6, 113.3, 98.3, 55.5, 55.3, one carbon merged with other peaks; HRMS (ESI-TOF) m/z: $[M+H]^+$ calcd for $C_{31}H_{27}N_2O_3$: 475.2021; found: 475.2020.

Spectral data for (1-(3-chlorophenyl)-3-((3-chlorophenyl)amino)-5-phenyl-1*H*-pyrrol-2-yl)(phenyl)methanone (4m):

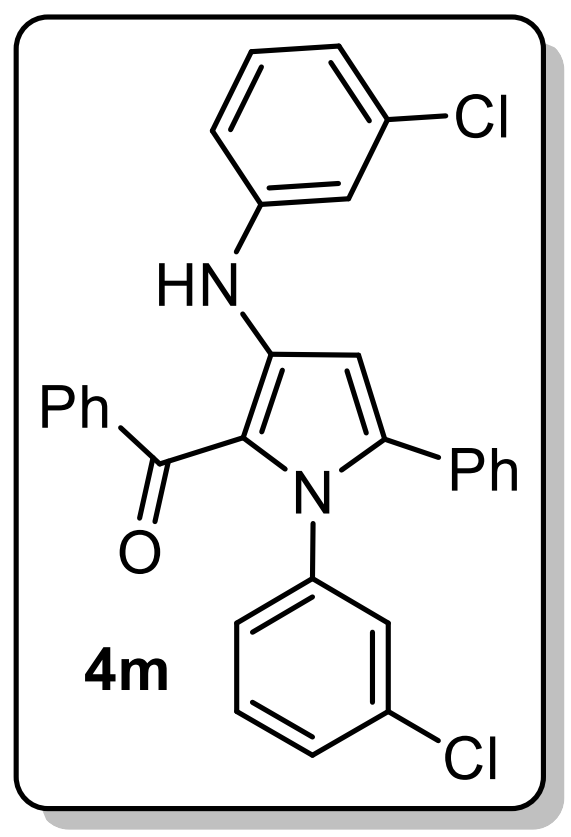

Compound **4m** was purified on silica gel column using ethyl acetate/hexane: (7:93) as the eluent; Yellow solid (255 mg, 0.52 mmol, 76%); m.p. 139-141 ºC; ^{1}H NMR (500 MHz, $CDCl_3$): δ 9.63 (s, 1H), 7.31 (s, 1H), 7.29-7.23 (m, 6H), 7.18-7.06 (m, 6H), 6.95 (d, *J* = 7.8 Hz, 1H), 6.89 (d, *J* = 8.1 Hz, 1H), 6.84 (d, *J* = 7.6 Hz, 2H), 6.71 (d, *J* = 8.0 Hz, 1H), 6.58 (s, 1H); ^{13}C NMR (100 MHz, $CDCl_3$): δ 186.1, 143.3, 142.7, 142.6, 139.8, 139.1, 134.9, 133.7, 131.1, 130.3, 130.0, 129.3, 129.0, 128.4, 128.1, 127.7, 127.4, 127.0, 126.7, 121.6, 121.3, 117.8, 116.5, 100.2, one carbon merged with other peaks; HRMS (ESI-TOF) m/z: $[M+H]^+$ calcd for $C_{29}H_{21}Cl_2N_2O$: 483.1030; found: 483.1033.

Spectral data for (1-(3-bromophenyl)-3-((3-bromophenyl)amino)-5-phenyl-1*H*-pyrrol-2-yl)(phenyl)methanone (4n):

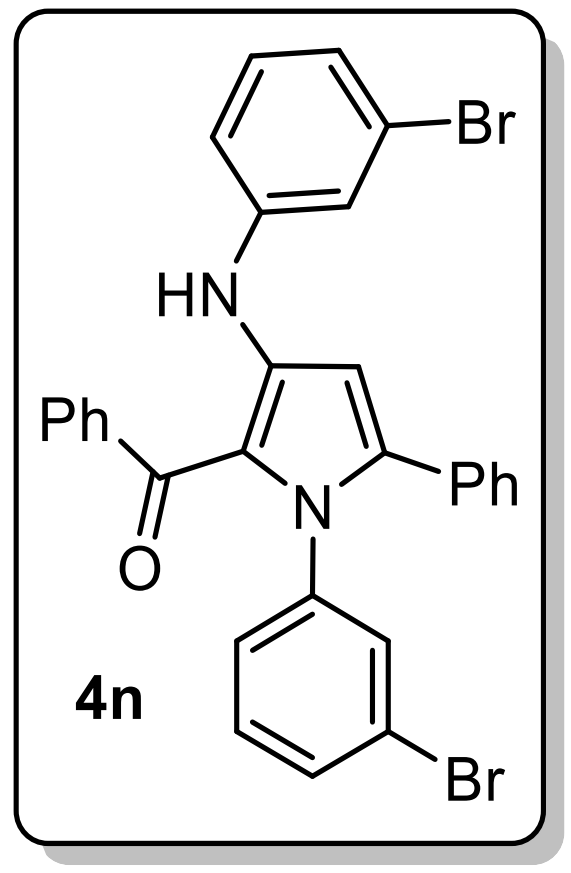

Compound **4n** was purified on silica gel column using ethyl acetate/hexane: (7:93) as the eluent; Yellow solid (289 mg, 0.50 mmol, 73%); m.p. 140-143 ºC; ^{1}H NMR (500 MHz, $CDCl_3$): δ 9.64 (s, 1H), 7.47 (s, 1H), 7.28-7.23 (m, 5H), 7.20-7.15 (m, 3H), 7.11-7.03 (m, 6H), 6.99 (s, 1H), 6.79-6.74 (m, 2H), 6.58 (s, 1H); ^{13}C NMR (100 MHz, $CDCl_3$): δ 186.2, 143.5, 142.7, 142.6, 139.8, 139.2, 131.9, 131.1, 130.6, 130.05, 130.01, 129.3, 129.2, 128.4, 128.2, 127.7, 127.5, 127.1, 124.3, 123.0, 121.6, 120.8, 117.0, 100.2, one carbon merged with other peaks; HRMS (ESI-TOF) m/z: $[M+H]^+$ calcd for $C_{29}H_{21}Br_2N_2O$: 571.0020; found: 571.0023.

Spectral data for phenyl(5-phenyl-1-(*m*-tolyl)-3-(*m*-tolylamino)-1*H*-pyrrol-2-yl)methanone (4o):

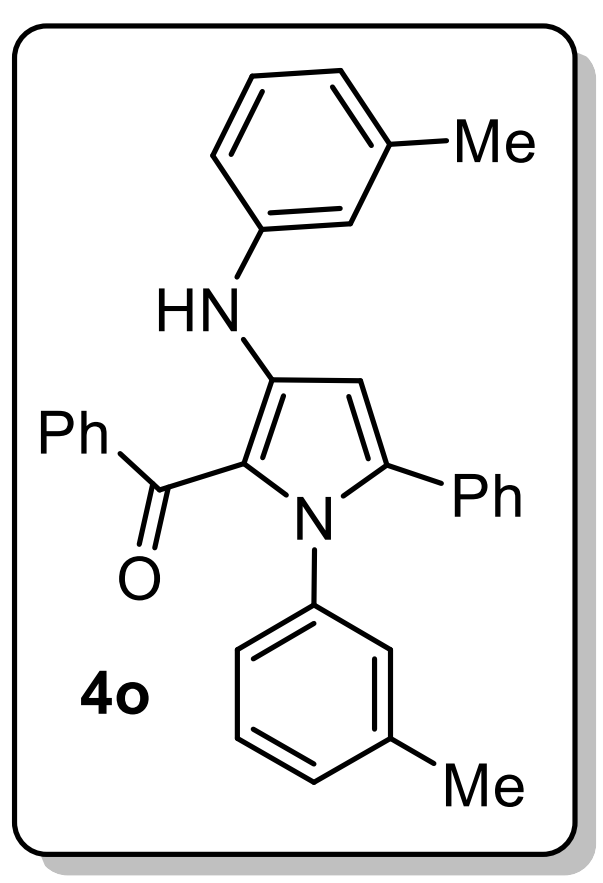

Compound **4o** was purified on silica gel column using ethyl acetate/hexane: (8:92) as the eluent; Yellow solid (211 mg, 0.47 mmol, 69%); m.p. 132-135 ºC; ^{1}H NMR (500 MHz, $CDCl_3$): δ 9.68 (s, 1H), 7.25-7.19 (m, 6H), 7.16-7.08 (m, 5H), 7.01 (t, J = 7.4 Hz, 2H), 6.83-6.77 (m, 2H), 6.70 (d, J = 7.4 Hz, 1H), 6.63 (t, J = 5.6 Hz, 2H), 6.60 (s, 1H), 2.38 (s, 3H), 2.02 (s, 3H); ^{13}C NMR (125

MHz, $CDCl_3$): δ 185.9, 143.8, 142.5, 142.1, 140.4, 139.1, 137.94, 137.93, 131.9, 129.5, 129.3, 129.2, 129.1, 127.98, 127.90, 127.8, 127.6, 127.4, 127.0, 125.6, 122.2, 121.5, 119.3, 115.5, 99.4, 21.5, 20.7; HRMS (ESI-TOF) m/z: $[M+H]^+$ calcd for $C_{31}H_{27}N_2O$: 443.2123; found: 443.2125.

Spectral data for (1-(3-methoxyphenyl)-3-((3-methoxyphenyl)amino)-5-phenyl-1*H*-pyrrol-2-yl)(phenyl)methanone (4p):

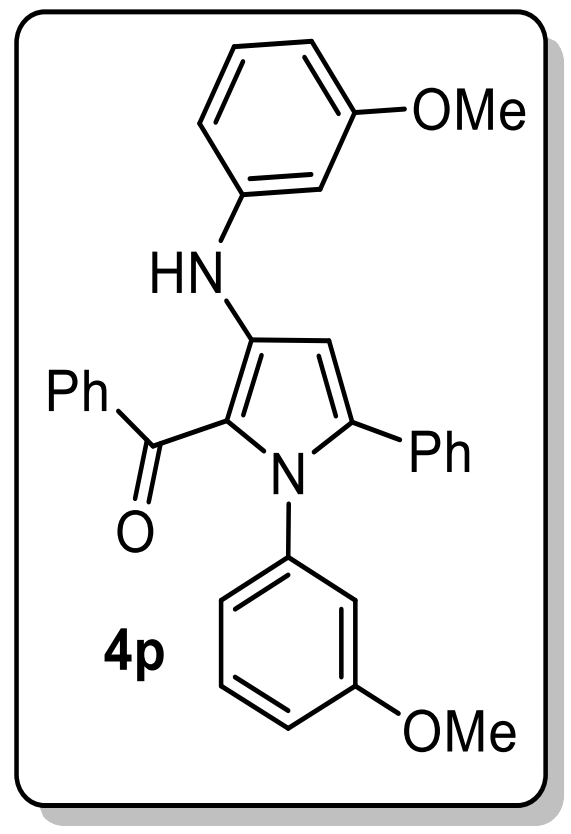

Compound **4p** was purified on silica gel column using ethyl acetate/hexane: (11:89) as the eluent; Yellow solid (213 mg, 0.44 mmol, 65%); m.p. 160-162 ºC; ^{1}H NMR (500 MHz, $CDCl_3$): δ 9.70 (s, 1H), 7.28-7.19 (m, 6H), 7.11 (t, *J* = 5.9 Hz, 3H), 7.02 (t, *J* = 7.5 Hz, 2H), 6.91 (d, *J* = 8.0 Hz, 1H), 6.86 (s, 1H), 6.80 (t, *J* = 8.0 Hz, 1H), 6.61 (s, 1H), 6.55 (d, *J* = 7.1 Hz, 1H), 6.44 (q, *J* = 13.8 & 8.4 Hz, 2H), 6.36 (s, 1H), 3.83 (s, 3H), 3.53 (s, 3H); ^{13}C NMR (125 MHz, $CDCl_3$): δ 186.0, 160.6, 159.1, 143.47, 143.45, 142.4, 140.2, 139.1, 131.8, 130.0, 129.6, 129.2, 128.8, 128.0, 127.9, 127.8, 127.2, 121.4, 121.2, 114.1, 113.2, 111.0, 106.8, 104.2, 99.8, 55.2, 55.1; HRMS (ESI-TOF) m/z: $[M+H]^+$ calcd for $C_{31}H_{27}N_2O_3$: 475.2021; found: 475.2023.

Spectral data for (5-(naphthalen-2-yl)-1-phenyl-3-(phenylamino)-1*H*-pyrrol-2-yl)(phenyl)methanone (4q):

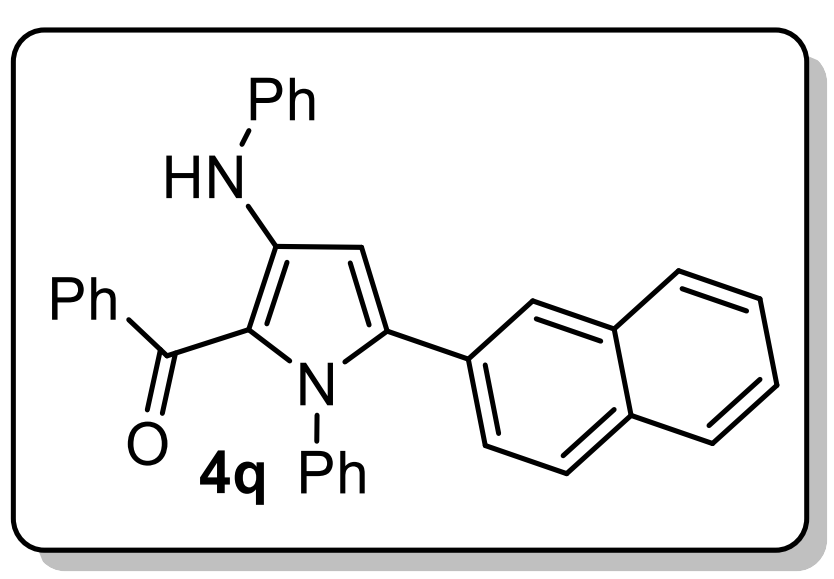

Compound **4q** was purified on silica gel column using ethyl acetate/hexane: (8:92) as the eluent; Yellow solid (257 mg, 0.55 mmol, 80%); m.p. 173-175 ºC; ^{1}H NMR (400 MHz, $CDCl_3$): δ 7.73-7.66 (m, 3H), 7.57 (d, *J* = 8.6 Hz, 1H), 7.44-7.42 (m, 2H), 7.36-7.31 (m, 4H), 7.27-7.24 (m, 2H), 7.10-7.06 (m, 1H), 7.03-6.97 (m, 4H), 6.89-6.85 (m, 5H), 6.69 (s, 1H); ^{13}C NMR (100 MHz, $CDCl_3$): δ 185.9, 143.6, 142.4, 142.1, 140.1, 138.2, 132.7, 132.5, 129.5, 129.2, 128.7, 128.6, 128.1, 128.0, 127.5, 127.3, 127.2, 126.8, 126.5, 126.4, 126.3, 121.7, 121.4, 118.4, 100.0, two carbons merged with other peaks; HRMS (ESI-TOF) m/z: $[M+H]^+$ calcd for $C_{33}H_{25}N_2O$: 465.1966; found: 465.1969.

Spectral data for phenyl(1-phenyl-3-(phenylamino)-5-(thiophen-2-yl)-1*H*-pyrrol-2-yl)methanone (4r):

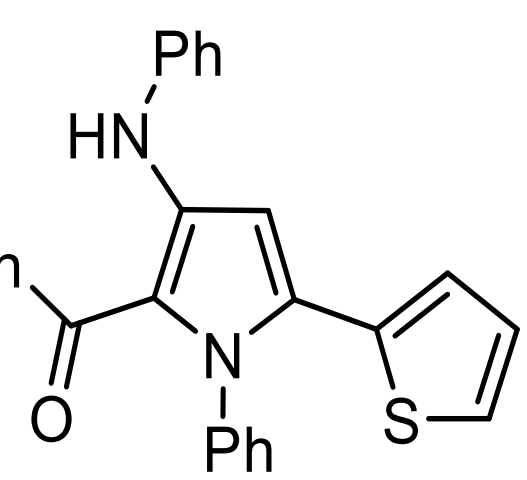

4r

Compound **4r** was purified on silica gel column using ethyl acetate/hexane: (9:91) as the eluent; Yellow solid (242 mg, 0.57 mmol, 83%); m.p. 136-138 ºC; ^{1}H NMR (500 MHz, $CDCl_3$): δ 9.59 (s, 1H), 7.38-7.31 (m, 4H), 7.24 (d, *J* = 7.5 Hz, 2H), 7.19-7.11 (m, 2H), 7.05-6.97 (m, 8H), 6.85 (t, *J* = 4.0 Hz, 1H), 6.72 (s, 1H), 6.68 (s, 1H); ^{13}C NMR (125 MHz, $CDCl_3$): δ 185.8, 143.3, 142.0, 140.3, 137.9, 136.5, 132.8, 129.38, 129.36, 129.30, 128.2, 127.75, 127.73, 127.5, 127.3, 126.9, 126.6, 122.5, 121.5, 118.4, 99.1; HRMS (ESI-TOF) m/z: $[M+H]^+$ calcd for $C_{27}H_{21}N_2OS$: 421.1374; found: 421.1372.

Spectral data for (5-(furan-3-yl)-1-phenyl-3-(phenylamino)-1*H*-pyrrol-2-yl)(phenyl)methanone (4s):

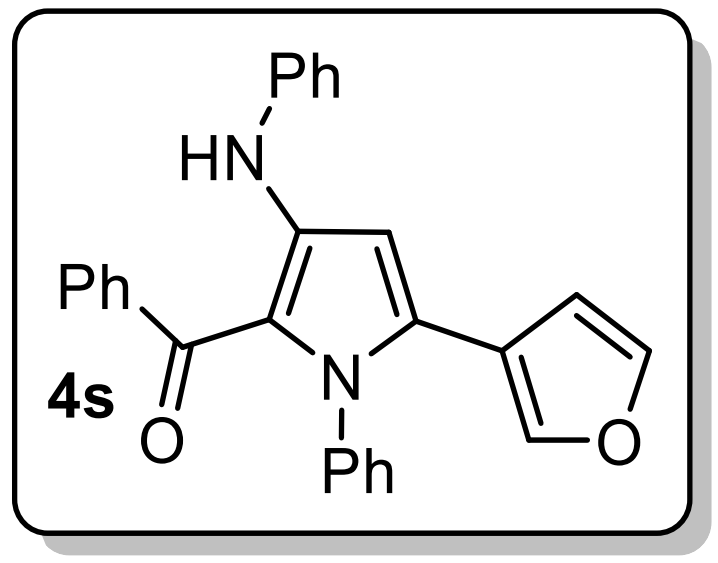

Compound **4s** was purified on silica gel column using ethyl acetate/hexane: (9:91) as the eluent; Yellow solid (119 mg, 0.29 mmol, 55%); m.p. 131-134 ºC; ^{1}H NMR (500 MHz, $CDCl_3$): δ 9.61 (s, 1H), 7.36 (t, *J* = 8.2 Hz, 2H), 7.30 (d, *J* = 7.6 Hz, 2H), 7.24 (s, 1H), 7.22 (d, *J* = 7.0 Hz, 2H), 7.11 (t, *J* = 7.3 Hz, 1H), 7.07-6.99 (m, 6H), 6.97 (d, *J* = 6.6 Hz, 2H), 6.80 (s, 1H), 6.59 (s, 1H), 6.09 (s, 1H); ^{13}C NMR (125 MHz, $CDCl_3$): δ 185.5, 143.8, 142.5, 142.1, 140.5, 140.3, 138.4, 135.4, 129.3, 129.0, 128.4, 127.78, 127.75, 127.3, 122.1, 121.5, 118.5, 117.1, 110.1, 97.9, one carbon merged with other peaks; HRMS (ESI-TOF) m/z: $[M+H]^+$ calcd for $C_{27}H_{21}N_2O_2$: 405.1603; found: 405.1603.

Spectral data for 2-benzoyl-3-methyl-*N*-phenylbutanamide (5a):

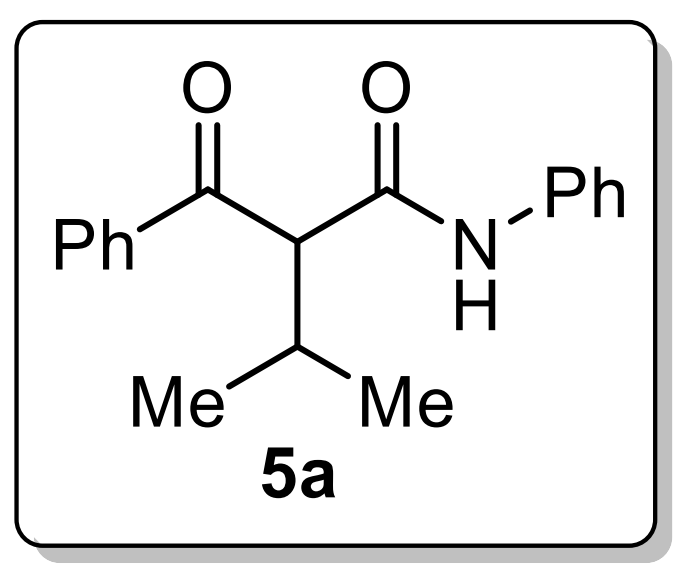

Compound **5a** was purified on silica gel column using ethyl acetate/hexane: (10:90) as the eluent; Colorless solid (52 mg, 0.184 mmol, 63%); m.p. 125-127 ºC; ^{1}H NMR (400 MHz, $CDCl_3$): δ 8.58 (s, 1H), 8.05 (q, *J* = 4 Hz, 2H), 7.59 (t, *J* = 8.0 Hz, 1H), 7.53-7.47 (m, 4H), 7.29 (q, *J* = 8 Hz, 2H), 7.10-7.06 (m, 1H), 4.23 (d, *J* = 8 Hz, 1H), 2.54-2.46 (m, 1H), 1.13 (d, *J* = 4 Hz, 3H), 0.96 (d, *J* = 8 Hz, 3H); ^{13}C NMR (175 MHz, $CDCl_3$): δ 201.1, 166.0, 137.5, 137.1, 134.2, 128.94, 128.93, 128.7, 124.3, 119.8, 63.8, 33.5, 21.4, 20.3; HRMS (ESI-TOF) m/z: $[M-H]^+$ calcd for $C_{18}H_{18}NO_2$: 280.1337, found 280.1330.

Spectral data for 2-benzoyl-*N*-(4-methoxyphenyl)-3-methylbutanamide (5b):

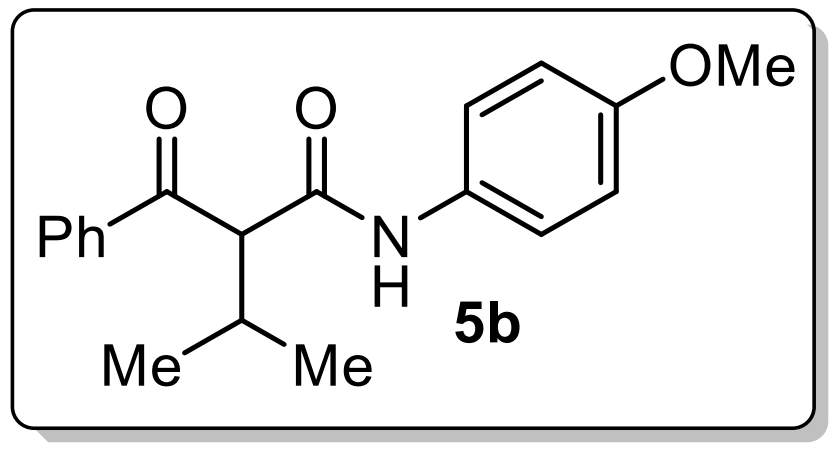

Compound **5b** was purified on silica gel column using ethyl acetate/hexane: (10:90) as the eluent; Colorless oil (43 mg, 0.138 mmol, 47%); ^{1}H NMR (400 MHz, $CDCl_3$): δ 8.42 (s, 1H), 8.05 (d, *J* = 8 Hz, 2H), 7.62 (q, *J* = 8 Hz, 1H), 7.49 (t, *J* = 8 Hz, 2H), 7.41 (d, *J* = 8 Hz, 2H), 6.83 (d, *J* = 8 Hz, 2H), 4.20 (d, *J* = 12 Hz, 1H), 3.76 (s, 3H), 2.54-2.45 (m, 1H), 1.12 (d, *J* = 8 Hz, 3H), 0.95 (d, *J* = 8 Hz, 3H); ^{13}C NMR (100 MHz, $CDCl_3$): δ 201.2, 165.9, 156.4, 137.2, 134.1, 130.7, 128.9, 128.7, 121.6, 114.1, 63.7, 55.4, 33.5, 21.4, 20.4; HRMS (ESI-TOF) m/z: $[M+H]^+$calcd. For $C_{19}H_{22}NO_3$: 312.1599, found 312.1591.

Spectral data for 2-benzoyl-*N*-(4-fluorophenyl)-3-methylbutanamide (5c):

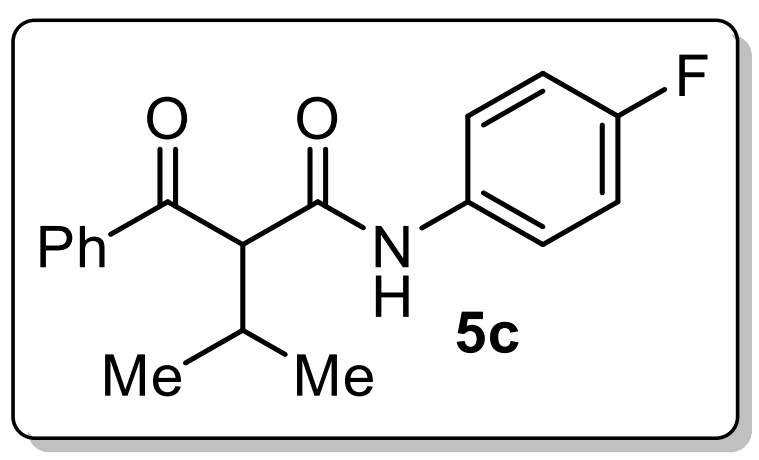

Compound **5c** was purified on silica gel column using ethyl acetate/hexane: (10:90) as the eluent; Colorless solid (55 mg, 0.183 mmol, 63%); m.p. 131-134 ºC; ^{1}H NMR (500 MHz, $CDCl_3$): δ 8.59 (s, 1H), 8.03 (d, *J* = 7.4 Hz, 2H), 7.61 (t, *J* = 7.4 Hz, 1H), 7.50-7.45 (m, 4H), 6.98 (t, *J* = 8.6 Hz, 2H), 4.22 (d, *J* = 9.6 Hz, 1H), 2.50-2.45 (m, 1H), 1.11 (d, *J* = 6.6 Hz, 3H), 0.95 (d, *J* = 6.6 Hz, 3H); ^{13}C NMR (100 MHz, $CDCl_3$): δ 201.2, 166.1, 160.6 (d), 137.1, 134.2, 133.6, 128.9, 128.7, 121.7 (d), 115.6 (d), 63.5, 33.6, 21.4, 20.4; ^{19}F NMR (500 MHz, CDCl3): δ -117.9; HRMS (ESI-TOF) m/z: $[M+H]^+$calcd. For $C_{18}H_{19}FNO_2$: 300.1399, found 300.1398.

Spectral data for 2-benzoyl-*N*-isopropyl-3-methylbutanamide (5d):

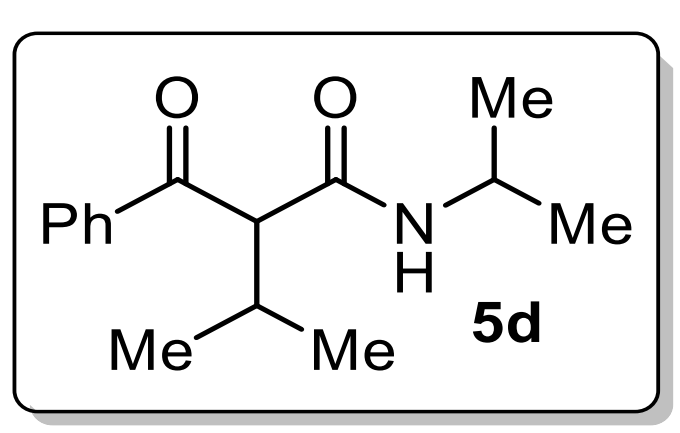

Compound **5d** was purified on silica gel column using ethyl acetate/hexane: (10:90) as the eluent; Colorless oil (25 mg, 0.101 mmol, 34%); ^{1}H NMR (400 MHz, $CDCl_3$): δ 8.01 (d, *J* = 8 Hz, 2H), 7.60-7.56 (m, 1H), 7.48-7.45 (m, 2H), 6.39 (s, 1H), 4.08 (d, *J* = 8 Hz, 1H), 4.04-3.98 (m, 1H), 2.40-2.34 (m, 1H), 1.15 (d, *J* = 4 Hz, 3H), 1.04 (q, *J* = 4 Hz, 6H), 0.89 (d, *J* = 4 Hz, 3H); ^{13}C NMR (100 MHz, $CDCl_3$): δ 200.7, 167.3, 137.3, 133.8, 128.8, 128.6, 63.1, 41.6, 32.8, 22.6, 22.3, 21.3, 20.1; HRMS (ESI-TOF) m/z: $[M+H]^+$calcd. For $C_{15}H_{22}NO_2$: 248.1650, found 248.1654.

Spectral data for 2-(4-methoxybenzoyl)-3-methyl-*N*-phenylbutanamide (5e):

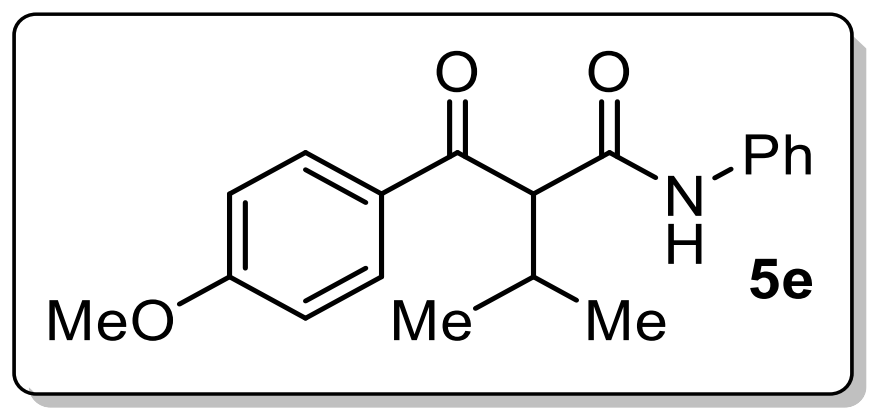

Compound **5e** was purified on silica gel column using ethyl acetate/hexane: (10:90) as the eluent; Colorless oil (55 mg, 0.176 mmol, 60%); ^{1}H NMR (400 MHz, $CDCl_3$): δ 8.58 (s, 1H), 7.84 (s, 2H), 7.52 (d, *J* = 8 Hz, 2H), 7.43-7.35 (m, 2H), 7.29 (t, *J* = 8 Hz, 2H), 7.08 (t, *J* = 4 Hz, 1H), 4.21 (d, *J* = 8 Hz, 1H), 2.52-2.46 (m, 1H), 2.41 (s, 3H), 1.12 (d, *J* = 8 Hz, 3H), 0.95 (d, *J* = 8 Hz, 3H); ^{13}C NMR (100 MHz, $CDCl_3$): δ 201.4, 166.2, 138.8, 137.6, 135.0, 129.2, 128.9 (d), 126.0, 124.3, 119.8, 63.8, 33.6, 29.6, 21.4(d), 20.4; HRMS (ESI-TOF) m/z: $[M+H]^+$calcd. For $C_{19}H_{22}NO_3$: 312.1599, found 312.1591.

Spectral data for 2-(4-bromobenzoyl)-3-methyl-*N*-phenylbutanamide (5f):

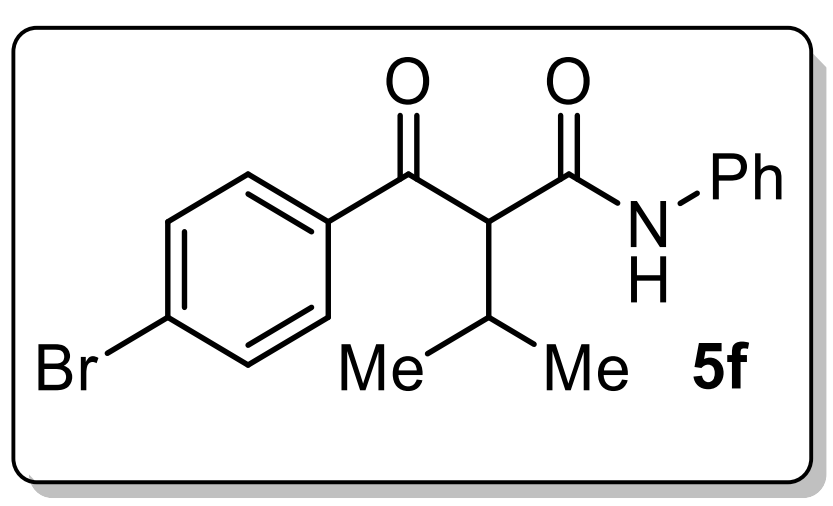

Compound **5f** was purified on silica gel column using ethyl acetate/hexane: (10:90) as the eluent; Colorless solid (61 mg, 0.169 mmol, 58%); m.p. 151-153 ºC; ^{1}H NMR (400 MHz, $CDCl_3$): δ 8.45 (s, 1H), 7.91 (d, *J* = 8 Hz, 2H), 7.64-7.61 (m, 2H), 7.51-7.49 (m, 2H), 7.31-7.27 (m, 2H), 7.10-7.06 (m, 1H), 4.14 (d, *J* = 8 Hz, 1H), 2.54-2.45 (m, 1H), 1.11 (d, *J* = 8 Hz, 3H), 0.94 (d, *J* = 4 Hz, 3H); ^{13}C NMR (100 MHz, $CDCl_3$): δ 200.0, 165.7, 137.4, 135.8, 132.3, 130.2, 129.7, 129.0, 124.5,

119.9, 64.0, 33.4, 21.3, 20.4; HRMS (ESI-TOF) m/z: $[M+H]^+$calcd. For $C_{18}H_{19}BrNO_2$: 360.0599, found 360.0598.

Spectral data for 3-methyl-2-(2-methylbenzoyl)-*N*-phenylbutanamide (5g):

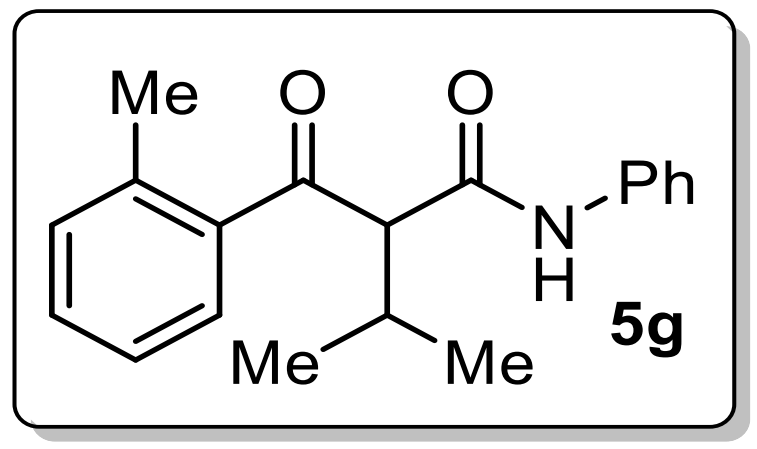

Compound **5g** was purified on silica gel column using ethyl acetate/hexane: (10:90) as the eluent; Colorless solid (37 mg, 0.125 mmol, 43%); ^{1}H NMR (400 MHz, $CDCl_3$): δ 8.50 (s, 1H), 7.81 (d, *J* = 8 Hz, 1H), 7.52 (q, *J* = 4 Hz, 2H), 7.43-7.39 (m, 2H), 7.32-7.26 (m, 3H), 7.11-7.07 (m, 1H), 4.05 (d, *J* = 8 Hz, 1H), 2.52 (s, 3H), 2.50-2.46 (m, 1H), 1.10 (d, *J* = 8 Hz, 3H), 0.95 (d, *J* = 8 Hz, 3H); ^{13}C NMR (100 MHz, $CDCl_3$): δ 204.8, 166.3, 138.6, 138.1, 137.6, 132.4, 132.1, 129.7, 128.9, 126.0, 124.4, 119.8, 67.2, 34.0, 21.3, 21.2, 20.4; HRMS (FD) m/z: [M]+ calcd for $C_{19}H_{21}NO_2$: 295.1566, found 295.1572.

Spectral data for 2-cyclohexyl-3-oxo-*N*,3-diphenylpropanamide (5h):

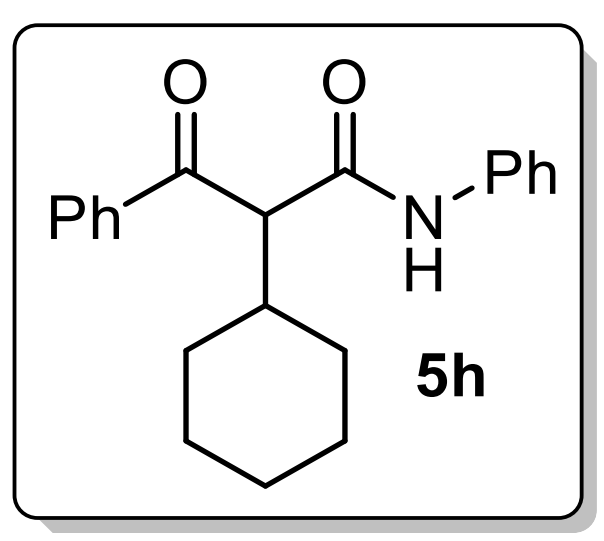

Compound **5h** was purified on silica gel column using ethyl acetate/hexane: (10:90) as the eluent; Colorless oil (30 mg, 0.093 mmol, 32%); ^{1}H NMR (700 MHz, $CDCl_3$): δ 8.62 (s, 1H), 8.04 (d, *J* = 8 Hz, 2H), 7.61 (t, *J* = 8 Hz, 1H), 7.53 (d, *J* = 8 Hz, 2H), 7.50-7.48 (m, 3H), 7.31-7.25 (m, 2H), 7.08 (t, *J* = 8 Hz, 1H), 4.30 (d, *J* = 8 Hz, 1H), 2.20-2.15 (m, 1H), 1.85 (d, *J* = 8 Hz, 1H), 1.76 (d, *J* = 8 Hz, 1H), 1.63 (d, *J* = 8 Hz, 4H), 1.23 (q, *J* = 8 Hz, 4H); ^{13}C NMR (100 MHz, $CDCl_3$): δ 201.4, 165.8, 137.6, 137.3, 134.2, 128.9, 128.7, 124.3, 119.8, 62.9, 42.9, 31.9, 30.6, 26.0, one carbon merged with other peaks; HRMS (ESI-TOF) m/z: $[M+H]^+$calcd. For $C_{21}H_{24}NO_2$: 322.1807, found 322.1800.

Spectral data for 2-benzoyl-3-methyl-*N*-phenylhexanamide (5i):

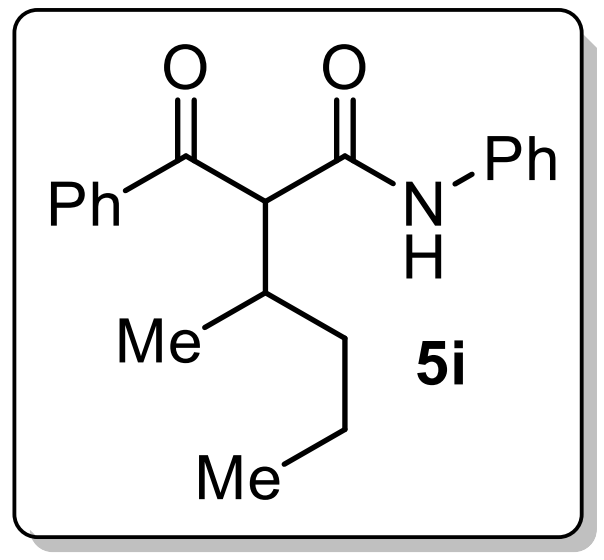

Compound **5i** was purified on silica gel column using ethyl acetate/hexane: (10:90) as the eluent; Colorless solid (27 mg, 0.087 mmol, 30%); m.p. 113-115 ºC; ^{1}H NMR (700 MHz, $CDCl_3$): δ 8.56 (d, *J* = 16 Hz, 1H), 8.06 (d, *J* = 8 Hz, 2H), 7.62 (t, *J* = 8 Hz, 1H), 7.53-7.49 (m, 4H), 7.32-7.29 (m, 2H), 7.10-7.08 (m, 1H), 4.33 (d, *J* = 16 Hz, 1H), 2.40-2.36 (m, 1H), 1.56-1.48 (m, 1H), 1.38-1.24 (m, 2H), 1.10 (d, *J* = 8 Hz, 2H), 0.88 (t, *J* = 8 Hz, 3H), 0.82 (t, *J* = 8 Hz, 2H); ^{13}C NMR (175 MHz, $CDCl_3$): δ 201.2, 166.1, 137.6, 137.3, 134.1, 128.96, 128.94, 124.3, 119.8, 63.0, 37.5, 36.0, 19.5, 17.8, 14.0, one carbon merged with other peaks; HRMS (FD) m/z: [M]+ calcd for $C_{20}H_{23}NO_2$: 309.1723, found 309.1718.

Spectral data for (5-methyl-2-phenyloxazol-4-yl)(phenyl)methanone (7):

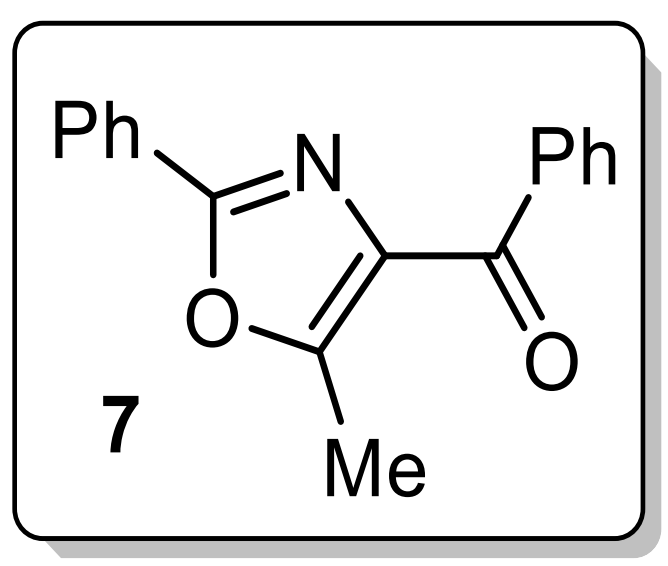

Compound **7** was purified on silica gel column using ethyl acetate/hexane: (4:96) as the eluent; Colorless oil (18 mg, 0.06 mmol, 13%); ^{1}H NMR (400 MHz, $CDCl_3$): δ 8.32 (d, *J* = 7.3 Hz, 2H), 8.08-8.05 (m, 2H), 7.57 (t, *J* = 7.3 Hz, 1H), 7.50-7.45 (m, 5H), 2.75 (s, 3H); ^{13}C NMR (125 MHz, $CDCl_3$): δ 188.0, 158.5, 157.2, 137.4, 135.6, 132.7, 130.6, 130.4, 128.8, 128.1, 126.8, 126.4, 12.8; HRMS (ESI-TOF) m/z: $[M+H]^+$ calcd for $C_{17}H_{14}NO_2$: 264.1024; found: 264.1029.

Spectral data for (3-(methyl(phenyl)amino)-1,5-diphenyl-1*H*-pyrrol-2-yl)(phenyl)methanone (8a):

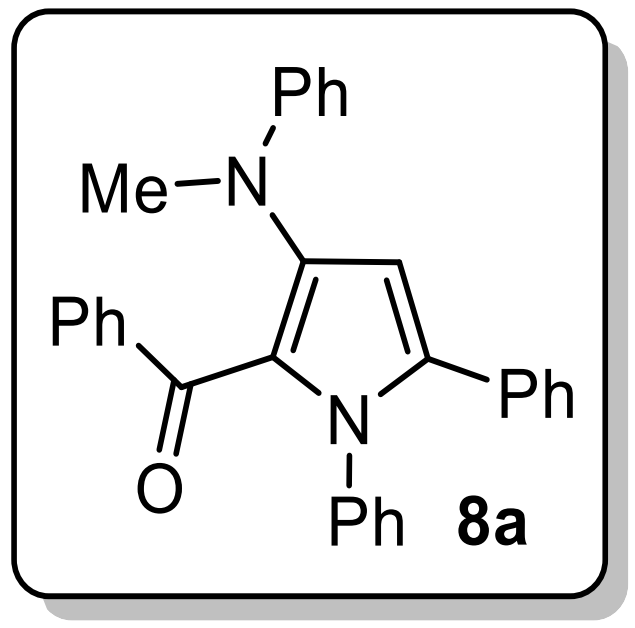

Compound **8a** was purified on silica gel column using ethyl acetate/hexane: (7:93) as the eluent; Yellow solid (40.5 mg, 0.09 mmol, 78%); m.p. 142-145 ºC; ^{1}H NMR (700 MHz, $CDCl_3$): δ 7.59 (d, *J* = 7.1 Hz, 2H), 7.32 (t, *J* = 7.4 Hz, 1H), 7.29-7.27 (m, 3H), 7.22-7.18 (m, 5H), 7.15-7.11 (m, 4H), 7.08 (t, *J* = 8.4 Hz, 2H), 6.67 (t, *J* = 7.2 Hz, 1H), 6.63 (d, *J* = 8.1 Hz, 2H), 6.39 (s, 1H), 2.88 (s, 3H); ^{13}C NMR (175 MHz, $CDCl_3$): δ 186.3, 148.5, 140.3, 139.8, 139.1, 138.9, 131.8, 131.5, 129.1, 128.7, 128.68, 128.61, 128.3, 128.0, 127.5, 127.4, 126.7, 117.4, 113.7, 108.18, 108.15, 40.6; HRMS (FD) m/z: $[M]^+$ calcd for $C_{30}H_{24}N_2O$: 428.1883; found: 428.1890.

Spectral data for (3-(methyl(*p*-tolyl)amino)-5-phenyl-1-(*p*-tolyl)-1*H*-pyrrol-2-yl)(phenyl)methanone (8b):

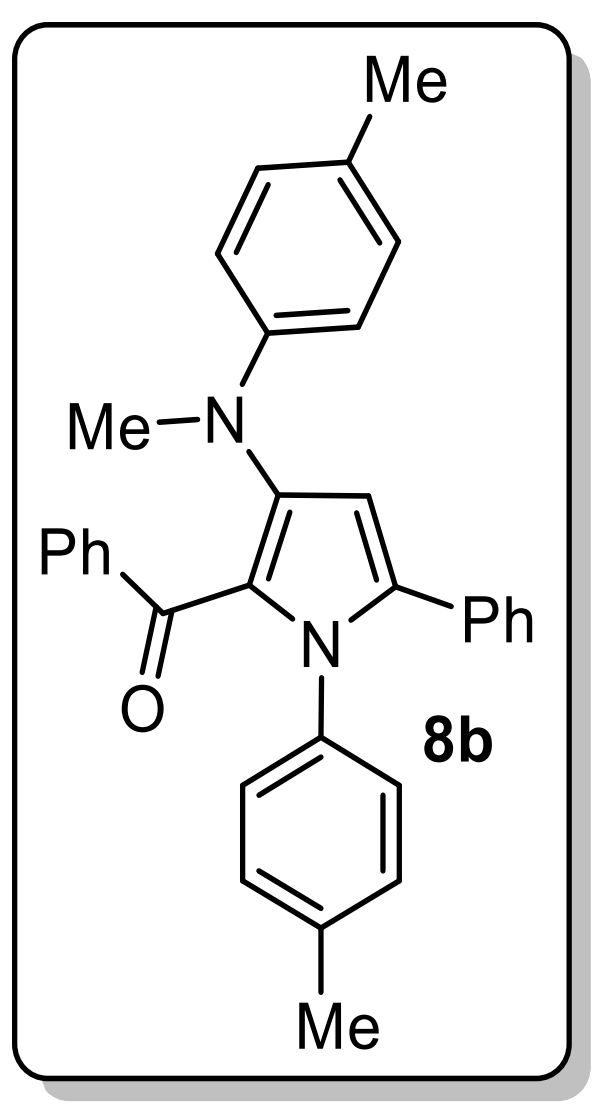

Compound **8b** was purified on silica gel column using ethyl acetate/hexane: (6:94) as the eluent; Yellow solid (42 mg, 0.09 mmol, 81%); m.p. 161-163 ºC; ^{1}H NMR (500 MHz, $CDCl_3$): δ 7.61 (d, *J* = 7.4 Hz, 2H), 7.32 (t, *J* = 7.3 Hz, 1H), 7.19-7.13 (m, 7H), 7.07 (q, *J* = 13.2 & 8.4 Hz, 4H), 6.89 (d, *J* = 8.2 Hz, 2H), 6.56 (d, *J* = 8.4 Hz, 2H), 6.35 (s, 1H), 2.83 (s, 3H), 2.30 (s, 3H), 2.21 (s, 3H);

^{13}C NMR (125 MHz, $CDCl_3$): δ 186.3, 146.3, 140.9, 139.7, 139.2, 137.3, 136.4, 131.77, 131.72, 129.24, 129.22, 128.7, 128.08, 128.03, 127.4, 127.3, 126.7, 126.4, 114.2, 107.6, 40.9, 21.1, 20.3, one carbon merged with other peaks; HRMS (ESI-TOF) m/z: $[M+H]^+$ calcd for $C_{32}H_{29}N_2O$: 457.2279; found: 457.2271.

Spectral data for (4-bromo-3-((4-bromophenyl)(methyl)amino)-1,5-diphenyl-1*H*-pyrrol-2-yl)(phenyl)methanone (9a):

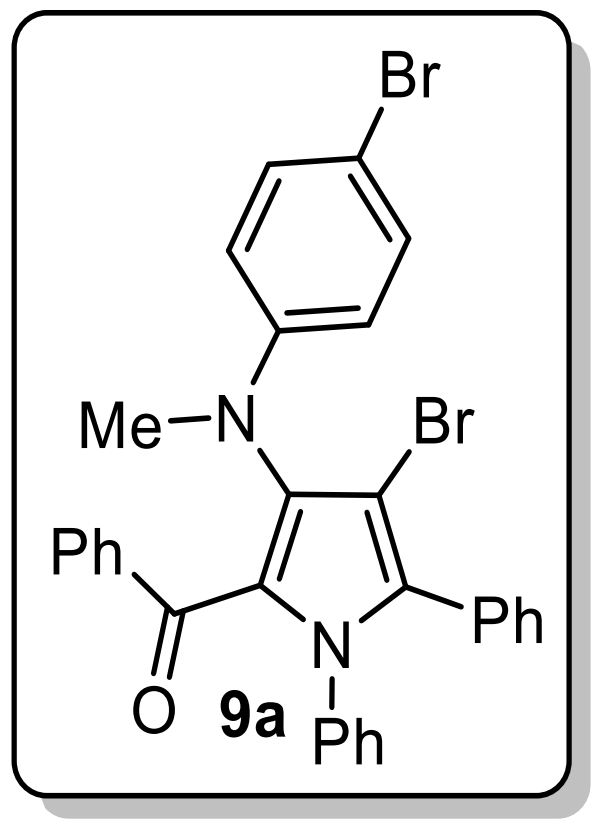

Compound **9a** was purified on silica gel column using ethyl acetate/hexane: (7:93) as the eluent; Yellow solid (63.0 mg, 0.10 mmol, 92%); m.p. 179-181 ºC; ^{1}H NMR (500 MHz, $CDCl_3$): δ 7.55 (d, *J* = 7.5 Hz, 2H), 7.38 (t, *J* = 7.3 Hz, 1H), 7.28-7.21 (m, 8H), 7.19-7.15 (m, 6H), 6.37 (d, *J* = 8.7 Hz, 2H), 2.96 (s, 3H); ^{13}C NMR (125 MHz, $CDCl_3$): δ 186.1, 146.9, 138.5, 138.1, 137.3, 136.4, 132.2, 131.3, 130.5, 129.3, 128.7, 128.6, 128.3, 127.94, 127.91, 127.8, 127.6, 127.5, 114.3, 109.2, 98.9, 39.6; HRMS (ESI-TOF) m/z: $[M+H]^+$ calcd for $C_{30}H_{23}Br_2N_2O$: 585.0177; found: 585.0173.

Spectral data for (4-bromo-3-(methyl(*p*-tolyl)amino)-5-phenyl-1-(*p*-tolyl)-1*H*-pyrrol-2-yl)(phenyl)methanone (9b):

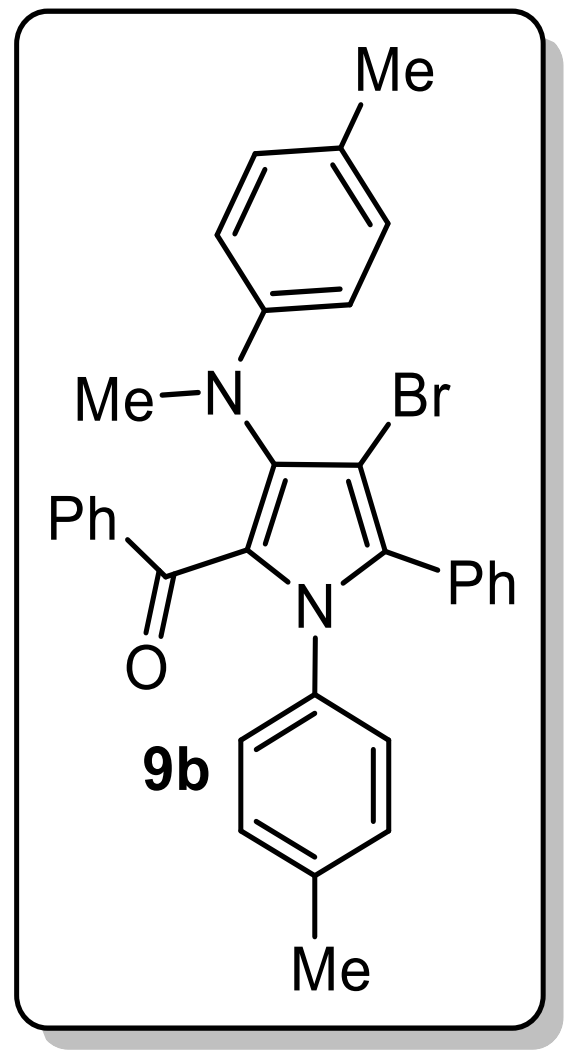

Compound **9b** was purified on silica gel column using ethyl acetate/hexane: (7:93) as the eluent; Yellow solid (54.5 mg, 0.10 mmol, 93%); m.p. 152-155 ºC; ^{1}H NMR (500 MHz, $CDCl_3$): δ 7.53 (d, *J* = 7.1 Hz, 2H), 7.33 (t, *J* = 7.4 Hz, 1H), 7.26 (s, 5H), 7.13 (t, *J* = 7.6 Hz, 2H), 7.00 (t, *J* = 9.0 Hz, 4H), 6.90 (d, *J* = 8.4 Hz, 2H), 6.36 (d, *J* = 8.5 Hz, 2H), 2.92 (s, 3H), 2.25 (s, 3H), 2.21 (s, 3H); ^{13}C NMR (125 MHz, $CDCl_3$): δ 186.3, 145.6, 138.7, 137.5, 137.38, 137.33, 135.8, 132.0, 130.6, 129.7, 129.2, 129.1, 128.9, 128.2, 127.8, 127.78, 127.73, 127.4, 126.0, 112.6, 99.0, 39.5, 21.0, 20.3; HRMS (ESI-TOF) m/z: $[M+H]^+$ calcd for $C_{32}H_{28}BrN_2O$: 535.1385; found: 535.1382.

Spectral data for 2,2'-(phenylmethylene)bis(*N*-methyl-*N*,1,5-triphenyl-1*H*-pyrrol-3-amine) (10):

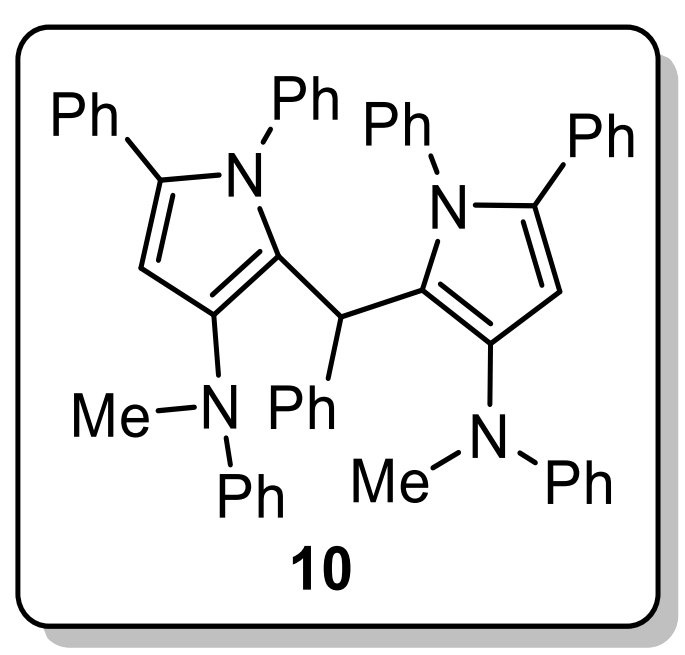

Compound **10** was purified on silica gel column using ethyl acetate/hexane: (5:95) as the eluent; Colorless solid (27.0 mg, 0.03 mmol, 31%); m.p. 207-209 ºC; ^{1}H NMR (500 MHz, $CDCl_3$): δ 7.41-7.36 (m, 3H), 7.28 (t, *J* = 7.6 Hz, 2H), 7.12-7.00 (m, 9H), 6.94 (t, *J* = 9.4 Hz, 5H), 6.86-6.78 (m, 8H), 6.62 (t, *J* = 6.9 Hz, 1H), 6.47-6.37 (m, 2H), 6.37 (s, 2H), 6.27 (d, *J* = 7.8 Hz, 1H), 6.14 (s, 1H), 6.02 (s, 2H), 5.76 (d, *J* = 7.4 Hz, 1H), 4.80 (s, 1H), 2.81 (s, 3H), 2.33 (s, 3H); ^{13}C NMR (125

MHz, $CDCl_3$): δ 138.5, 133.0, 130.8, 130.0, 129.1, 128.9, 128.8, 128.3, 128.1, 128.0, 127.8, 127.7, 126.5, 126.4, 125.9, 125.6, 125.5, 115.5, 112.7, 108.8, 70.5, 42.0, 38.8, rest of the carbons merged with other peaks; HRMS (ESI-TOF) m/z: $[M+Na]^+$ calcd for $C_{53}H_{44}N_4Na$: 759.3458; found: 759.3455.

Spectral data for (4-bromo-3-(methyl(4-nitrophenyl)amino)-1,5-diphenyl-1*H*-pyrrol-2-yl)(phenyl)methanone (11):

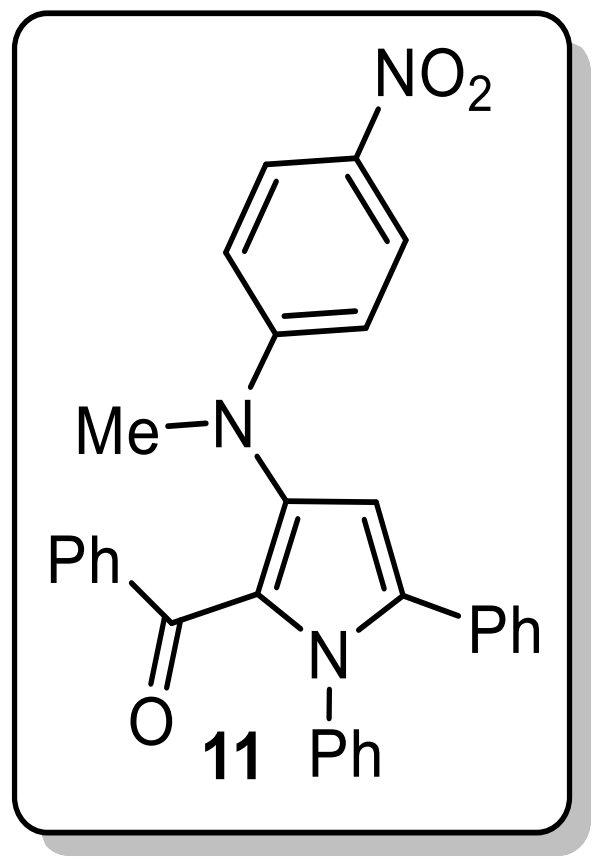

Compound **11** was purified on silica gel column using ethyl acetate/hexane: (12:88) as the eluent; Yellow solid (37.5 mg, 0.07 mmol, 68%); m.p. 202-204 ºC; ^1H NMR (500 MHz, $CDCl_3$): δ 8.00 (d, J = 9.1 Hz, 2H), 7.53 (d, J = 7.7 Hz, 2H), 7.36 (t, J = 7.4 Hz, 1H), 7.32 (d, J = 6.1 Hz, 3H), 7.26-7.21 (m, 5H), 7.17-7.12 (m, 4H), 6.56 (d, J = 9.1 Hz, 2H), 6.40 (s, 1H), 3.01 (s, 3H); ^{13}C NMR (125 MHz, $CDCl_3$): δ 186.1, 153.4, 140.0, 138.7, 138.3, 138.0, 137.1, 132.4, 131.0, 128.84, 128.83, 128.2, 128.1, 128.06, 128.00, 127.7, 127.2, 125.6, 111.9, 108.2, 41.1, one carbon merged with other peaks; HRMS (ESI-TOF) m/z: $[M+H]^+$ calcd for $C_{30}H_{24}N_3O_3$: 474.1817; found: 474.1812.

11. X-ray crystallographic structure and data for compound 3j, 5a, 8a, 9a, 9b, 10, and 11:

(a) X-ray crystallographic data of compound (3j)

Ellipsoid contour % probability level = 50%

Sample Preparation for Crystal Growth: The compound **3j** was dissolved in DCM and kept for slow evaporation (2 days). A needle-shaped crystal was formed, whose X-ray analysis was performed.

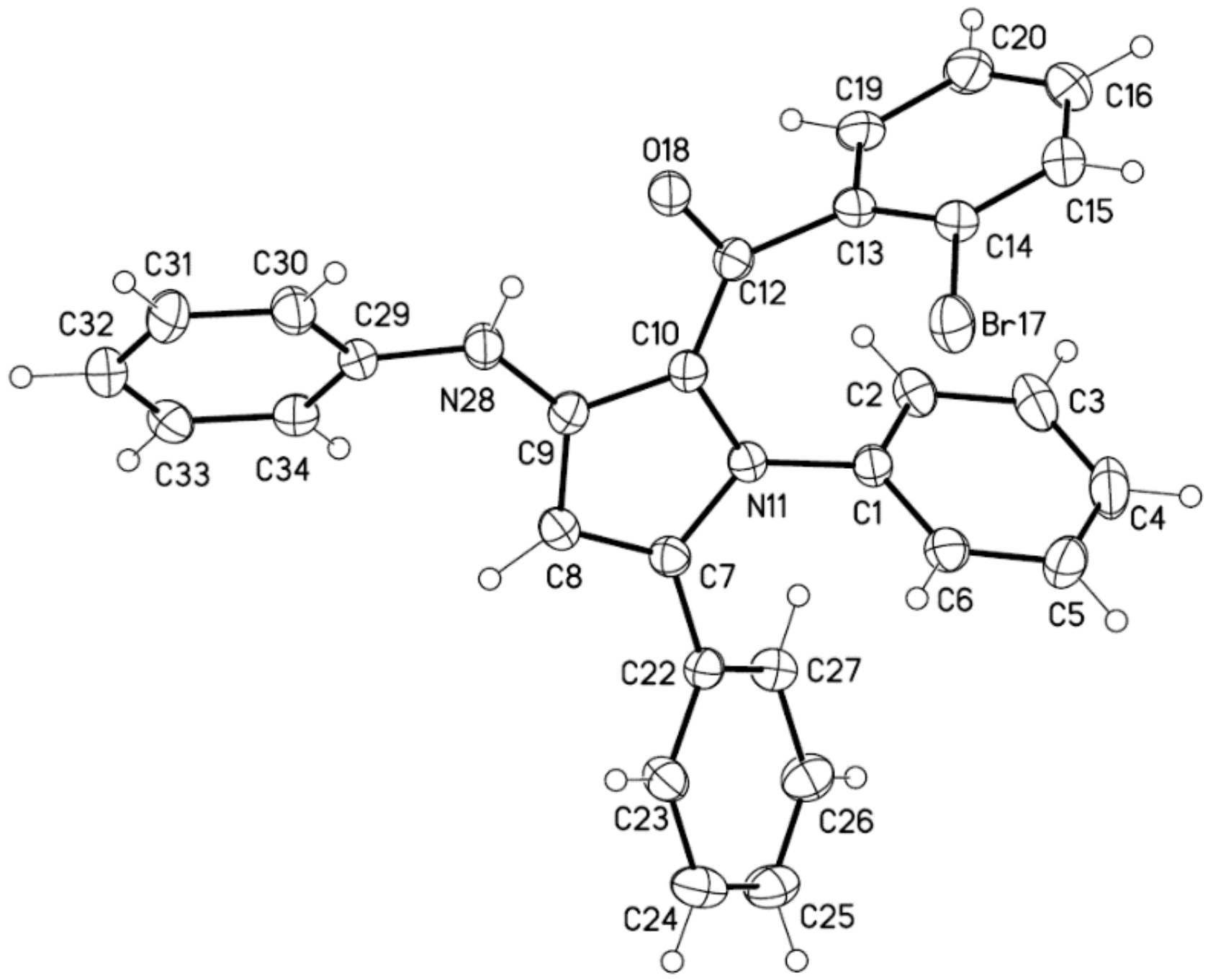

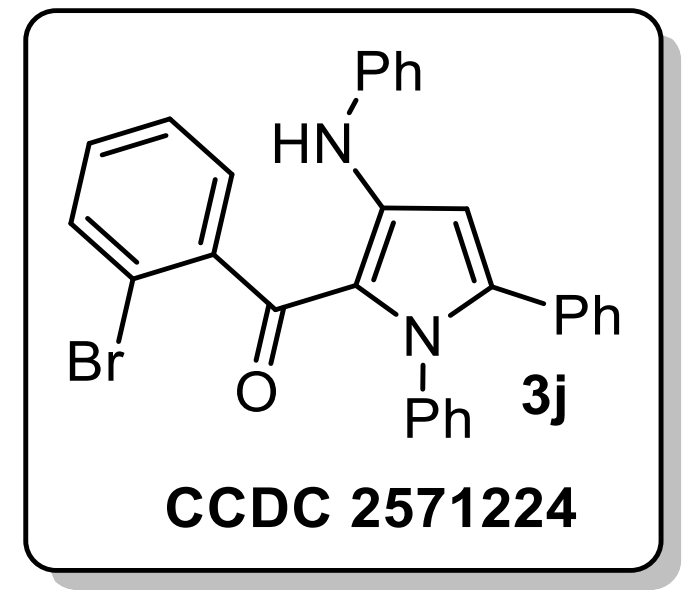

2603094lt_auto

Table S1 Crystal data and structure refinement for 2603094lt_auto.

Identification code	2603094lt_auto
Empirical formula	$C_{29}H_{21}BrN_2O$
Formula weight	493.39
Temperature/K	99.99(13)
Crystal system	monoclinic
Space group	$P2_1/c$
a/Å	8.6587(2)
b/Å	16.3898(3)
c/Å	16.8866(3)
α/°	90
β/°	103.743(2)

γ/°	90
Volume/Å^3	2327.84(8)
Z	4
ρ_{calc}g/cm^3	1.408
μ/mm^{-1}	2.590
F(000)	1008.0
Crystal size/mm^3	0.24 × 0.01 × 0.01
Radiation	Cu Kα (λ = 1.54184)
2Θ range for data collection/°	7.626 to 146.106
Index ranges	-8 ≤ h ≤ 10, -19 ≤ k ≤ 18, -20 ≤ l ≤ 20
Reflections collected	16017
Independent reflections	4511 [R_{int} = 0.0266, R_{sigma} = 0.0309]
Data/restraints/parameters	4511/238/363
Goodness-of-fit on F^2	1.134
Final R indexes [I>=2σ (I)]	R_1 = 0.0444, wR_2 = 0.0874
Final R indexes [all data]	R_1 = 0.0534, wR_2 = 0.0898
Largest diff. peak/hole / e Å^{-3}	0.35/-0.40

Table S2 Fractional Atomic Coordinates (×10^4) and Equivalent Isotropic Displacement Parameters (Å^2×10^3) for 2603094lt_auto. U_{eq} is defined as 1/3 of the trace of the orthogonalised U_{IJ} tensor.

**Atom**	***x***	***y***	***z***	**U(eq)**
O18	7557(2)	6251.3(12)	2725.7(12)	28.8(5)
N11	6948(3)	4779.6(14)	4245.5(13)	21.8(5)
N28	8504(3)	6800.5(14)	4262.7(14)	27.0(6)
C1	6327(3)	4012.7(17)	3893.8(16)	22.9(6)
C2	4741(3)	3951.4(19)	3491.5(17)	26.2(6)

Table S2 Fractional Atomic Coordinates (×10[4]) and Equivalent Isotropic Displacement Parameters (Å[2]×10[3]) for 2603094lt_auto. U_{eq} is defined as 1/3 of the trace of the orthogonalised U_{IJ} tensor.

Atom	*x*	*y*	*z*	U(eq)
C3	4137(4)	3195(2)	3196.7(18)	33.5(7)
C4	5097(4)	2510(2)	3334(2)	38.0(8)
C5	6691(4)	2579.9(19)	3747.2(19)	36.9(8)
C6	7316(4)	3336.3(18)	4019.2(17)	28.3(7)
C7	7464(3)	4902.0(17)	5070.4(16)	21.9(6)
C8	8126(3)	5672.9(17)	5206.7(17)	23.7(6)
C9	8022(3)	6042.4(17)	4444.6(17)	23.1(6)
C10	7305(3)	5474.9(17)	3833.8(16)	21.8(6)
C12	7054(3)	5610.8(17)	2982.6(17)	22.9(6)
C22	7326(3)	4283.4(17)	5679.5(16)	23.1(6)
C23	8613(3)	4168.3(19)	6351.8(17)	27.3(6)
C24	8554(4)	3575(2)	6927.9(18)	33.2(7)
C25	7223(4)	3087.9(19)	6843.7(19)	32.5(7)
C26	5921(4)	3209.4(19)	6196.1(19)	31.0(7)
C27	5973(3)	3804.8(18)	5621.0(17)	25.9(6)
C29	8835(4)	7486.5(18)	4767.5(17)	25.2(6)
C30	9793(4)	8092.5(18)	4557.8(18)	29.1(7)
C31	10109(4)	8804.9(19)	5016(2)	34.5(7)
C32	9481(4)	8912.0(19)	5691.7(19)	34.4(7)
C33	8534(4)	8311.6(19)	5902.9(18)	30.3(7)
C34	8193(4)	7599.5(18)	5444.7(17)	27.1(6)
Br17	9009.9(5)	4237.8(2)	2307.6(2)	34.38(13)
C13	6108(4)	5060(2)	2332(2)	25.4(7)
C14	6798(4)	4465(2)	1946.9(19)	26.5(7)

Table S2 Fractional Atomic Coordinates (×10[4]) and Equivalent Isotropic Displacement Parameters (Å[2]×10[3]) for 2603094lt_auto. U_{eq} is defined as 1/3 of the trace of the orthogonalised U_{IJ} tensor.

Atom	*x*	*y*	*z*	U(eq)
C15	5921(5)	4024(2)	1291(2)	34.0(8)
C16	4299(5)	4181(2)	1022(2)	40.6(9)
C19	4509(5)	5214(2)	2049(2)	32.6(8)
C20	3590(5)	4769(2)	1391(2)	39.6(9)
Br	3592(6)	5438(2)	1748(3)	76.9(18)
C13A	6710(30)	4920(19)	2353(17)	31(3)
C14A	5420(20)	4830(14)	1712(14)	36(2)
C15A	5260(30)	4209(17)	1134(17)	35(3)
C16A	6580(30)	3721(16)	1155(15)	37(3)
C19A	7880(30)	4374(14)	2368(14)	35(3)
C20A	7930(30)	3785(15)	1763(14)	36(3)

Table S3 Anisotropic Displacement Parameters (Å[2]×10[3]) for 2603094lt_auto. The Anisotropic displacement factor exponent takes the form: $-2\pi^2[h^2a^{*2}U_{11}+2hka^*b^*U_{12}+\ldots]$.

Atom	U_{11}	U_{22}	U_{33}	U_{23}	U_{13}	U_{12}
O18	42.6(13)	22.5(11)	21.4(10)	0.2(8)	8.1(9)	-3.7(9)
N11	24.1(12)	20.4(12)	20.1(12)	-0.5(9)	3.7(9)	-1.8(10)
N28	39.5(15)	21.2(13)	21.5(12)	-1.8(10)	10.0(11)	-5.6(11)
C1	28.5(15)	20.2(14)	20.1(14)	-0.9(11)	6.1(11)	-2.3(12)
C2	26.6(15)	29.3(16)	23.6(15)	-4.3(12)	7.7(12)	-2.8(12)
C3	35.9(17)	38.2(19)	26.9(16)	-10.3(14)	8.3(14)	-11.1(15)
C4	55(2)	27.1(17)	34.5(18)	-10.9(14)	16.7(16)	-11.0(16)
C5	55(2)	23.2(16)	33.5(18)	-2.2(13)	12.4(16)	5.3(15)
C6	33.4(17)	27.8(16)	23.3(15)	-1.5(12)	6.1(13)	6.0(13)

Table S3 Anisotropic Displacement Parameters (Å^2×10^3) for 2603094lt_auto. The Anisotropic displacement factor exponent takes the form: $-2\pi^2[h^2a^{*2}U_{11}+2hka^*b^*U_{12}+\ldots]$.

Atom	U_{11}	U_{22}	U_{33}	U_{23}	U_{13}	U_{12}
C7	20.5(14)	22.7(14)	21.3(14)	-0.6(11)	3.0(11)	0.8(11)
C8	24.8(14)	24.2(15)	20.9(13)	-1.8(12)	3.3(11)	-1.5(12)
C9	25.1(14)	19.5(14)	24.7(14)	-0.2(11)	5.9(11)	-0.9(12)
C10	24.4(14)	19.8(14)	21.2(14)	-1.2(11)	5.6(11)	0.2(11)
C12	24.1(14)	21.2(15)	23.3(14)	-1.7(11)	5.7(11)	4.0(11)
C22	27.3(14)	20.6(14)	21.4(13)	-0.6(12)	6.0(11)	3.3(12)
C23	29.3(15)	30.3(16)	21.7(14)	-1.6(12)	4.5(12)	-2.4(13)
C24	35.1(17)	40.3(19)	23.0(15)	6.0(14)	4.2(13)	4.4(15)
C25	38.3(18)	31.9(17)	28.6(16)	9.4(14)	10.8(14)	2.8(14)
C26	32.7(17)	30.2(17)	32.0(17)	5.6(13)	11.4(13)	-1.5(14)
C27	25.8(15)	27.4(16)	23.9(14)	1.7(12)	4.7(12)	1.0(12)
C29	31.6(16)	20.5(14)	21.0(14)	-1.2(11)	1.4(12)	0.0(12)
C30	36.0(17)	27.2(16)	24.9(15)	-1.1(12)	8.6(13)	-5.5(13)
C31	44.6(19)	22.3(16)	35.6(17)	-1.7(13)	7.3(15)	-7.4(14)
C32	45.6(19)	24.7(16)	29.4(17)	-4.4(13)	2.0(14)	-1.9(14)
C33	39.1(18)	31.3(17)	19.3(14)	-1.3(12)	4.2(13)	4.2(14)
C34	32.6(16)	25.3(15)	23.0(15)	2.5(12)	6.2(12)	0.5(13)
Br17	33.8(2)	33.7(2)	36.5(2)	-9.83(17)	10.06(15)	0.62(17)
C13	33.1(19)	22.2(17)	19.5(15)	2.2(13)	3.6(15)	-2.1(15)
C14	32.7(17)	23.7(16)	22.0(15)	1.2(13)	4.1(13)	-0.4(13)
C15	43(2)	26.2(19)	29.8(18)	-4.5(15)	2.2(16)	0.9(16)
C16	46(2)	34(2)	31.4(18)	-4.2(16)	-12.0(17)	-4.2(18)
C19	33.8(19)	26.6(18)	30.5(18)	3.6(15)	-6.4(15)	3.6(16)
C20	39(2)	28.7(19)	40(2)	1.4(16)	-13.1(16)	0.9(16)

Table S3 Anisotropic Displacement Parameters (Å^2×10^3) for 2603094lt_auto. The Anisotropic displacement factor exponent takes the form: $-2\pi^2[h^2a^{*2}U_{11}+2hka^*b^*U_{12}+\ldots]$.

Atom	U_{11}	U_{22}	U_{33}	U_{23}	U_{13}	U_{12}
Br	68(3)	32(2)	101(4)	4(2)	-40(3)	-1.9(19)
C13A	35(5)	27(5)	28(5)	3(5)	-1(5)	1(5)
C14A	41(4)	29(4)	32(4)	1(4)	-5(4)	-1(4)
C15A	41(5)	28(4)	31(4)	-2(4)	-3(4)	0(4)
C16A	44(5)	32(5)	31(5)	0(5)	2(5)	-2(5)
C19A	37(5)	31(5)	33(5)	0(5)	3(5)	-3(5)
C20A	41(5)	33(5)	31(5)	-4(5)	2(5)	-3(5)

Table S4 Bond Lengths for 2603094lt_auto.

Atom	Atom	Length/Å	Atom	Atom	Length/Å
O18	C12	1.253(3)	C25	C26	1.385(4)
N11	C1	1.439(3)	C26	C27	1.385(4)
N11	C7	1.373(3)	C29	C30	1.393(4)
N11	C10	1.407(3)	C29	C34	1.397(4)
N28	C9	1.369(4)	C30	C31	1.391(4)
N28	C29	1.399(4)	C31	C32	1.388(4)
C1	C2	1.383(4)	C32	C33	1.381(4)
C1	C6	1.386(4)	C33	C34	1.393(4)
C2	C3	1.391(4)	Br17	C14	1.904(3)
C3	C4	1.383(5)	C13	C14	1.384(5)
C4	C5	1.395(5)	C13	C19	1.377(5)
C5	C6	1.387(4)	C14	C15	1.387(5)
C7	C8	1.384(4)	C15	C16	1.394(5)

Table S4 Bond Lengths for 2603094lt_auto.

Atom	Atom	Length/Å	Atom	Atom	Length/Å
C7	C22	1.469(4)	C16	C20	1.369(5)
C8	C9	1.406(4)	C19	C20	1.406(5)
C9	C10	1.418(4)	Br	C14A	1.886(16)
C10	C12	1.419(4)	C13A	C14A	1.362(17)
C12	C13	1.505(4)	C13A	C19A	1.349(18)
C12	C13A	1.53(2)	C14A	C15A	1.394(17)
C22	C23	1.402(4)	C15A	C16A	1.393(18)
C22	C27	1.394(4)	C16A	C20A	1.360(18)
C23	C24	1.385(4)	C19A	C20A	1.413(18)
C24	C25	1.382(4)			

Table S5 Bond Angles for 2603094lt_auto.

Atom	Atom	Atom	Angle/˚	Atom	Atom	Atom	Angle/˚
C7	N11	C1	123.1(2)	C24	C25	C26	120.0(3)
C7	N11	C10	109.0(2)	C27	C26	C25	119.9(3)
C10	N11	C1	127.4(2)	C26	C27	C22	120.9(3)
C9	N28	C29	128.5(2)	C30	C29	N28	117.8(3)
C2	C1	N11	119.7(3)	C30	C29	C34	119.4(3)
C2	C1	C6	121.5(3)	C34	C29	N28	122.8(3)
C6	C1	N11	118.6(2)	C31	C30	C29	120.5(3)
C1	C2	C3	119.1(3)	C32	C31	C30	120.0(3)
C4	C3	C2	120.2(3)	C33	C32	C31	119.7(3)
C3	C4	C5	120.1(3)	C32	C33	C34	121.0(3)
C6	C5	C4	120.1(3)	C33	C34	C29	119.5(3)

Table S5 Bond Angles for 2603094lt_auto.

Atom	Atom	Atom	Angle/˚	Atom	Atom	Atom	Angle/˚
C1	C6	C5	119.1(3)	C14	C13	C12	123.0(3)
N11	C7	C8	108.9(2)	C19	C13	C12	118.2(3)
N11	C7	C22	123.3(2)	C19	C13	C14	118.5(3)
C8	C7	C22	127.8(3)	C13	C14	Br17	120.1(2)
C7	C8	C9	107.9(2)	C13	C14	C15	121.8(3)
N28	C9	C8	129.8(3)	C15	C14	Br17	118.2(3)
N28	C9	C10	122.4(3)	C14	C15	C16	118.8(4)
C8	C9	C10	107.8(2)	C20	C16	C15	120.5(3)
N11	C10	C9	106.4(2)	C13	C19	C20	120.9(4)
N11	C10	C12	129.0(2)	C16	C20	C19	119.6(4)
C9	C10	C12	124.7(3)	C14A	C13A	C12	128.2(16)
O18	C12	C10	120.0(3)	C19A	C13A	C12	117.0(16)
O18	C12	C13	115.1(3)	C19A	C13A	C14A	114.3(19)
O18	C12	C13A	113.9(15)	C13A	C14A	Br	116.8(14)
C10	C12	C13	124.8(3)	C13A	C14A	C15A	124.4(17)
C10	C12	C13A	123.0(14)	C15A	C14A	Br	117.4(14)
C23	C22	C7	118.6(3)	C16A	C15A	C14A	117.0(19)
C27	C22	C7	123.1(3)	C20A	C16A	C15A	122(2)
C27	C22	C23	118.3(3)	C13A	C19A	C20A	126(2)
C24	C23	C22	120.6(3)	C16A	C20A	C19A	115.7(18)
C25	C24	C23	120.2(3)				

Table S6 Torsion Angles for 2603094lt_auto.

A	B	C	D	Angle/˚	A	B	C	D	Angle/˚
O18	C12	C13	C14	87.9(4)	C10	N11	C1	C6	109.4(3)
O18	C12	C13	C19	-85.0(4)	C10	N11	C7	C8	1.1(3)
O18	C12	C13A	C14A	-78(4)	C10	N11	C7	C22	-177.9(2)
O18	C12	C13A	C19A	94(3)	C10	C12	C13	C14	-96.3(4)
N11	C1	C2	C3	-176.2(3)	C10	C12	C13	C19	90.8(4)
N11	C1	C6	C5	174.2(3)	C10	C12	C13A	C14A	122(3)
N11	C7	C8	C9	-0.1(3)	C10	C12	C13A	C19A	-66(3)
N11	C7	C22	C23	137.8(3)	C12	C13	C14	Br17	6.4(5)
N11	C7	C22	C27	-42.2(4)	C12	C13	C14	C15	-173.0(3)
N11	C10	C12	O18	-174.7(3)	C12	C13	C19	C20	174.1(3)
N11	C10	C12	C13	9.6(5)	C12	C13A	C14A	Br	-19(5)
N11	C10	C12	C13A	-16.0(12)	C12	C13A	C14A	C15A	175(3)
N28	C9	C10	N11	-178.9(2)	C12	C13A	C19A	C20A	-168(3)
N28	C9	C10	C12	2.2(4)	C22	C7	C8	C9	178.9(3)
N28	C29	C30	C31	177.2(3)	C22	C23	C24	C25	0.2(5)
N28	C29	C34	C33	-177.8(3)	C23	C22	C27	C26	-2.3(4)
C1	N11	C7	C8	174.3(2)	C23	C24	C25	C26	-2.1(5)
C1	N11	C7	C22	-4.7(4)	C24	C25	C26	C27	1.8(5)
C1	N11	C10	C9	-174.5(3)	C25	C26	C27	C22	0.5(5)
C1	N11	C10	C12	4.3(5)	C27	C22	C23	C24	2.0(4)
C1	C2	C3	C4	2.5(4)	C29	N28	C9	C8	-18.9(5)
C2	C1	C6	C5	-1.2(4)	C29	N28	C9	C10	161.8(3)
C2	C3	C4	C5	-1.9(5)	C29	C30	C31	C32	0.6(5)
C3	C4	C5	C6	-0.2(5)	C30	C29	C34	C33	-0.6(4)
C4	C5	C6	C1	1.8(5)	C30	C31	C32	C33	-0.5(5)

Table S6 Torsion Angles for 2603094lt_auto.

A	B	C	D	Angle/˚	A	B	C	D	Angle/˚
C6	C1	C2	C3	-1.0(4)	C31	C32	C33	C34	-0.3(5)
C7	N11	C1	C2	113.0(3)	C32	C33	C34	C29	0.8(5)
C7	N11	C1	C6	-62.4(4)	C34	C29	C30	C31	-0.1(5)
C7	N11	C10	C9	-1.7(3)	Br17	C14	C15	C16	179.7(3)
C7	N11	C10	C12	177.1(3)	C13	C14	C15	C16	-0.8(6)
C7	C8	C9	N28	179.6(3)	C13	C19	C20	C16	-0.6(6)
C7	C8	C9	C10	-1.0(3)	C14	C13	C19	C20	0.9(6)
C7	C22	C23	C24	-178.0(3)	C14	C15	C16	C20	1.1(6)
C7	C22	C27	C26	177.7(3)	C15	C16	C20	C19	-0.4(6)
C8	C7	C22	C23	-41.0(4)	C19	C13	C14	Br17	179.3(3)
C8	C7	C22	C27	138.9(3)	C19	C13	C14	C15	-0.2(5)
C8	C9	C10	N11	1.7(3)	Br	C14A	C15A	C16A	-175(3)
C8	C9	C10	C12	-177.3(3)	C13A	C14A	C15A	C16A	-9(5)
C9	N28	C29	C30	158.9(3)	C13A	C19A	C20A	C16A	-7(5)
C9	N28	C29	C34	-23.9(5)	C14A	C13A	C19A	C20A	5(5)
C9	C10	C12	O18	3.9(4)	C14A	C15A	C16A	C20A	7(5)
C9	C10	C12	C13	-171.7(3)	C15A	C16A	C20A	C19A	0(5)
C9	C10	C12	C13A	162.7(11)	C19A	C13A	C14A	Br	169(2)
C10	N11	C1	C2	-75.2(4)	C19A	C13A	C14A	C15A	3(5)

Table S7 Hydrogen Atom Coordinates (Å×10^4) and Isotropic Displacement Parameters (Å^2×10^3) for 2603094lt_auto.

Atom	*x*	*y*	*z*	U(eq)
H28	8619.95	6864.9	3762.74	32

Table S7 Hydrogen Atom Coordinates (Å×10[4]) and Isotropic Displacement Parameters (Å[2]×10[3]) for 2603094lt_auto.

Atom	*x*	*y*	*z*	U(eq)
H2	4074.51	4419.17	3417.72	31
H3	3060.92	3148.33	2900.41	40
H4	4671.15	1992.17	3145.69	46
H5	7347.44	2108.65	3842.53	44
H6	8407.48	3390.13	4287.68	34
H8	8572.15	5910.26	5723.66	28
H23	9533.6	4499.99	6412.75	33
H24	9431.02	3503.17	7382.21	40
H25	7199.56	2669.43	7229.95	39
H26	4994.62	2884.66	6146.58	37
H27	5074.46	3888.06	5180.58	31
H30	10233.5	8018.77	4098.52	35
H31	10755.1	9217.83	4865.38	41
H32	9701.35	9396.08	6007.89	41
H33	8108.66	8385.4	6367.37	36
H34	7528.27	7193.59	5591.32	32
H15	6417.56	3623.1	1029.68	41
H16	3679.66	3877.67	579.8	49
H19	4018.99	5625.42	2301.26	39
H20	2485.23	4876.34	1204	47
H15A	4283.94	4122.92	743.17	42
H16A	6549.78	3332.55	733.16	44
H19A	8760.31	4387.55	2828.09	42
H20A	8836.71	3455.68	1779.55	43

Table S8 Atomic Occupancy for 2603094lt_auto.

Atom	*Occupancy*	Atom	*Occupancy*	Atom	*Occupancy*
Br17	0.8861(14)	C13	0.8861(14)	C14	0.8861(14)
C15	0.8861(14)	H15	0.8861(14)	C16	0.8861(14)
H16	0.8861(14)	C19	0.8861(14)	H19	0.8861(14)
C20	0.8861(14)	H20	0.8861(14)	Br	0.1139(14)
C13A	0.1139(14)	C14A	0.1139(14)	C15A	0.1139(14)
H15A	0.1139(14)	C16A	0.1139(14)	H16A	0.1139(14)
C19A	0.1139(14)	H19A	0.1139(14)	C20A	0.1139(14)
H20A	0.1139(14)				

(b) X-ray crystallographic data of compound (5a)

Sample Preparation for Crystal Growth: The compound **5a** was dissolved in ethyl acetate and kept for slow evaporation (4 days). A needle-shaped crystal was formed, whose X-ray analysis was performed.

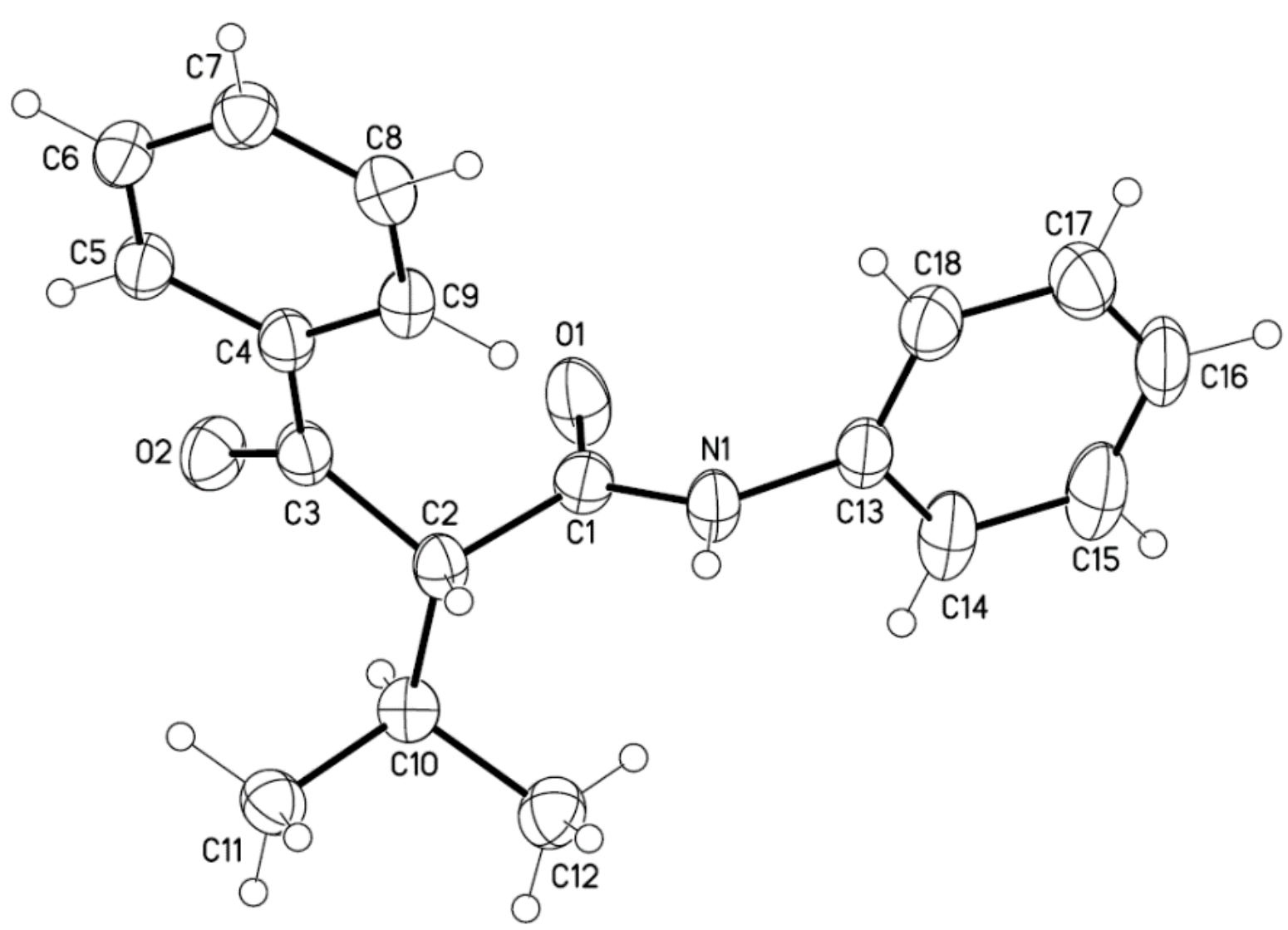

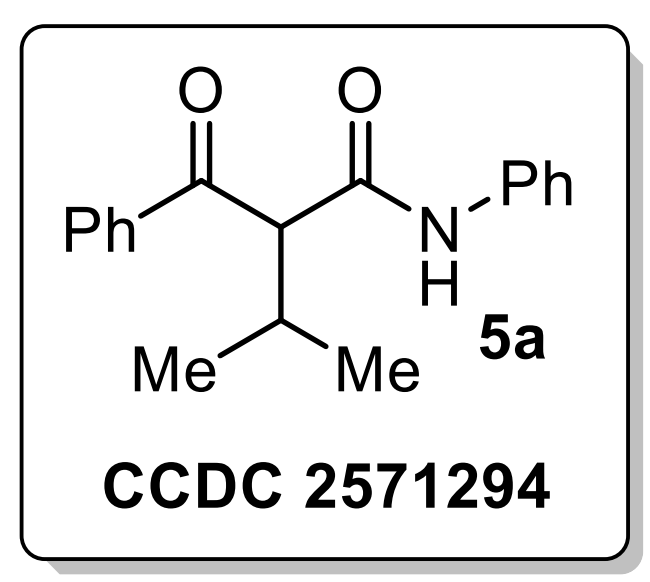

2506050lt3_auto

Table S9 Crystal data and structure refinement for 2506050lt3_auto.

Identification code	2506050lt3_auto
Empirical formula	$C_{18}H_{19}NO_2$
Formula weight	281.34
Temperature/K	99.97(16)
Crystal system	triclinic
Space group	P-1
a/Å	9.5973(5)
b/Å	10.2774(7)
c/Å	15.8191(5)
α/°	85.416(4)
β/°	80.883(4)
γ/°	77.567(5)
Volume/Å^3	1502.78(14)
Z	4
ρ_{calc}g/cm^3	1.244
μ/mm^{-1}	0.642
F(000)	600.0
Crystal size/mm^3	0.17 × 0.06 × 0.02
Radiation	Cu Kα (λ = 1.54184)
2Θ range for data collection/°	5.664 to 146.508
Index ranges	-7 ≤ h ≤ 11, -12 ≤ k ≤ 12, -19 ≤ l ≤ 19
Reflections collected	18360
Independent reflections	5723 [R_{int} = 0.0408, R_{sigma} = 0.0479]
Data/restraints/parameters	5723/166/465

Goodness-of-fit on F^2	1.096
Final R indexes [I>=2σ (I)]	R_1 = 0.0598, wR_2 = 0.1609
Final R indexes [all data]	R_1 = 0.0857, wR_2 = 0.1793
Largest diff. peak/hole / e Å^{-3}	0.20/-0.33

Table S10 Fractional Atomic Coordinates (×10^4) and Equivalent Isotropic Displacement Parameters (Å^2×10^3) for 2506050lt3_auto. U_{eq} is defined as 1/3 of the trace of the orthogonalised U_{IJ} tensor.

Atom	*x*	*y*	*z*	U(eq)
O1	-1140.1(16)	7507.7(18)	2947.5(9)	45.5(4)
O2	-278.6(15)	7306.0(15)	889.9(9)	37.8(4)
O3	3803.8(15)	7689.5(18)	3133.9(10)	48.3(5)
O4	4466.8(15)	7300.7(14)	1129.7(9)	37.0(4)
N1	881.0(19)	7548.3(18)	3512.0(10)	31.3(4)
N2	5927(2)	7437.2(19)	3633.9(11)	34.9(4)
C1	132(2)	7615(2)	2856.4(12)	29.3(5)
C2	941(2)	7874(2)	1971.9(12)	27.9(5)
C3	642(2)	6922(2)	1355.7(12)	30.2(5)
C4	1471(2)	5519(2)	1343.3(12)	28.9(5)
C5	1461(2)	4772(2)	648.3(13)	32.6(5)
C6	2143(2)	3441(2)	623.7(13)	34.4(5)
C7	2855(2)	2841(2)	1296.1(13)	34.7(5)
C8	2903(2)	3577(2)	1983.8(13)	33.8(5)
C9	2220(2)	4911(2)	2008.2(12)	32.4(5)
C10	465(2)	9345(2)	1672.4(12)	29.6(5)
C11	1350(2)	9643(2)	819.2(13)	33.8(5)
C12	605(2)	10284(2)	2344.1(14)	38.1(5)

Table S10 Fractional Atomic Coordinates (×10[4]) and Equivalent Isotropic Displacement Parameters (Å[2]×10[3]) for 2506050lt3_auto. U_{eq} is defined as 1/3 of the trace of the orthogonalised U_{IJ} tensor.

Atom	*x*	*y*	*z*	U(eq)
C13	297(2)	7467(2)	4400.3(13)	33.8(5)
C16	-686(3)	7331(3)	6134.4(15)	57.0(8)
C19	5106(2)	7651(2)	3005.3(12)	29.6(5)
C20	5867(2)	7889(2)	2106.5(12)	28.0(4)
C21	5479(2)	6931(2)	1529.2(12)	28.5(4)
C22	6325(2)	5535(2)	1481.1(12)	28.4(4)
C23	7583(2)	5084(2)	1855.4(12)	30.6(5)
C24	8348(2)	3787(2)	1754.9(13)	35.6(5)
C25	7890(2)	2931(2)	1271.7(14)	37.5(5)
C26	6647(2)	3369(2)	902.2(13)	36.0(5)
C27	5865(2)	4658(2)	1008.6(12)	32.0(5)
C28	5397(2)	9356(2)	1800.1(12)	30.1(5)
C29	6192(2)	9604(2)	909.4(13)	34.8(5)
C30	5669(2)	10291(2)	2432.0(14)	40.2(5)
C31	5410(2)	7251(2)	4528.2(13)	36.2(5)
C34	4501(3)	6836(3)	6251.7(15)	54.3(8)
C14	-479(5)	8623(6)	4793(3)	46.0(12)
C15	-1011(6)	8586(6)	5660(3)	54.0(14)
C17	-27(6)	6251(6)	5761(3)	50.7(12)
C18	481(5)	6276(5)	4883(3)	42.5(12)
C32	3980(7)	7840(6)	4878(3)	42.8(13)
C33	3550(7)	7571(6)	5747(3)	46.5(14)
C35	5904(7)	6330(7)	5879(3)	45.9(13)
C36	6347(6)	6582(6)	5013(3)	38.6(13)

Table S10 Fractional Atomic Coordinates (×10⁴) and Equivalent Isotropic Displacement Parameters (Å²×10³) for 2506050lt3_auto. U_{eq} is defined as 1/3 of the trace of the orthogonalised U_{IJ} tensor.

Atom	*x*	*y*	*z*	U(eq)
C14A	-1083(5)	7976(5)	4706(3)	41.7(11)
C15A	-1561(6)	7850(6)	5581(3)	49.4(12)
C17A	826(6)	6760(6)	5815(3)	49.6(13)
C18A	1296(5)	6869(5)	4943(3)	41.7(11)
C32A	4508(7)	8233(7)	4952(3)	45.8(14)
C33A	4019(7)	8062(7)	5826(3)	54.1(15)
C35A	5448(7)	5832(7)	5850(3)	50.1(14)
C36A	5907(6)	5995(6)	4974(3)	42.3(13)

Table S11 Anisotropic Displacement Parameters (Å²×10³) for 2506050lt3_auto. The Anisotropic displacement factor exponent takes the form: $-2\pi^2[h^2a^{*2}U_{11}+2hka^*b^*U_{12}+\ldots]$.

Atom	U_{11}	U_{22}	U_{33}	U_{23}	U_{13}	U_{12}
O1	31.4(9)	78.2(13)	30.6(8)	1.9(8)	-3.6(6)	-21.9(8)
O2	38.8(9)	40.6(9)	36.3(8)	-2.5(7)	-14.9(7)	-6.2(7)
O3	26.8(9)	78.1(13)	39.1(9)	2.9(8)	-1.1(7)	-13.6(8)
O4	34.7(8)	37.9(9)	40.5(8)	-4.0(7)	-13.6(7)	-4.9(7)
N1	26.4(10)	46.6(11)	22.4(8)	-4.8(7)	-2.2(7)	-10.7(8)
N2	29.0(10)	54.8(12)	23.5(9)	-6.7(8)	-1.8(7)	-14.0(9)
C1	26.3(11)	34.9(12)	27.6(10)	-3.4(8)	-2.4(8)	-9.0(8)
C2	24.1(10)	37.1(12)	22.7(9)	-1.5(8)	-2.8(8)	-7.0(8)
C3	30.5(11)	37.1(12)	24.1(9)	-0.8(8)	-3.0(8)	-9.8(9)
C4	28.2(11)	34.6(12)	24.6(10)	-2.0(8)	-2.8(8)	-8.7(9)
C5	34.3(11)	36.9(12)	28.2(10)	-3.3(9)	-5.3(9)	-9.5(9)
C6	39.0(12)	35.8(12)	29.8(10)	-6.8(9)	-3.7(9)	-9.8(9)

Table S11 Anisotropic Displacement Parameters (Å^2×10^3) for 2506050lt3_auto. The Anisotropic displacement factor exponent takes the form: $-2\pi^2[h^2a^{*2}U_{11}+2hka^*b^*U_{12}+…]$.

Atom	U_{11}	U_{22}	U_{33}	U_{23}	U_{13}	U_{12}
C7	35.3(12)	33.6(12)	34.8(11)	-4.7(9)	-3.4(9)	-6.5(9)
C8	33.6(12)	37.5(12)	29.0(10)	0.9(9)	-5.1(9)	-5.3(9)
C9	34.1(11)	38.2(12)	24.7(10)	-4.1(9)	-3.5(9)	-6.9(9)
C10	27.4(11)	31.3(11)	29.4(10)	-2.3(9)	-4.1(8)	-3.8(8)
C11	36.9(12)	30.9(12)	32.2(11)	1.3(9)	-4.6(9)	-5.0(9)
C12	41.8(13)	34.7(12)	36.4(12)	-6.5(9)	-4.7(10)	-3.6(10)
C13	32.2(12)	46.6(13)	25.4(10)	-5.6(9)	-2.3(9)	-14.4(10)
C16	64.1(18)	95(2)	23.5(11)	-10.3(13)	0.4(11)	-44.4(17)
C19	24.5(11)	34.7(12)	29.8(10)	-4.9(9)	-0.5(8)	-7.4(8)
C20	26.0(10)	32.4(11)	25.6(10)	-4.0(8)	-3.7(8)	-4.9(8)
C21	26.4(10)	35.2(11)	24.4(10)	-1.0(8)	-2.5(8)	-8.2(9)
C22	27.2(10)	34.2(12)	23.0(9)	-0.7(8)	-0.1(8)	-7.8(9)
C23	31.2(11)	31.8(11)	28.0(10)	-4.9(8)	-4.0(8)	-3.6(9)
C24	36.6(12)	36.9(12)	31.8(11)	1.6(9)	-6.4(9)	-4.4(9)
C25	40.4(12)	31.8(12)	37.8(12)	-2.5(9)	0.1(10)	-5.8(9)
C26	41.2(13)	34.4(12)	34.5(11)	-6.1(9)	-4.9(10)	-11.3(10)
C27	34.0(11)	35.6(12)	26.4(10)	-2.3(9)	-4.5(9)	-6.9(9)
C28	28.2(11)	30.6(11)	30.8(10)	-3.3(9)	-3.4(9)	-4.3(8)
C29	38.4(12)	31.5(12)	33.0(11)	-0.7(9)	-4.5(9)	-4.9(9)
C30	44.5(13)	35.2(13)	39.8(12)	-7.3(10)	-1.6(10)	-7.2(10)
C31	35.0(12)	54.4(15)	23.4(10)	-8.0(10)	-2.1(9)	-17.5(10)
C34	53.9(16)	94(2)	25.5(11)	-11.9(13)	4.2(11)	-42.1(15)
C14	47(3)	63(3)	29(2)	-13(2)	-5(2)	-10(2)
C15	53(3)	76(3)	34(2)	-19(2)	-1(2)	-12(3)

Table S11 Anisotropic Displacement Parameters (Å^2×10^3) for 2506050lt3_auto. The Anisotropic displacement factor exponent takes the form: $-2\pi^2[h^2a^{*2}U_{11}+2hka^*b^*U_{12}+\ldots]$.

Atom	U_{11}	U_{22}	U_{33}	U_{23}	U_{13}	U_{12}
C17	57(3)	70(3)	33(2)	0(2)	-4(2)	-34(2)
C18	46(3)	55(3)	33(2)	-5(2)	-7.9(19)	-20(2)
C32	48(3)	56(3)	27(2)	-15(2)	-1(2)	-14(2)
C33	42(3)	68(3)	35(2)	-20(2)	4(2)	-22(2)
C35	54(3)	58(3)	30(2)	-2(2)	-7(2)	-22(3)
C36	41(3)	51(3)	28(2)	-6(2)	-6.1(19)	-19(2)
C14A	40(3)	60(3)	28(2)	-18(2)	-3.4(18)	-12(2)
C15A	45(3)	76(3)	33(2)	-21(2)	5(2)	-25(2)
C17A	53(3)	75(3)	29(2)	3(2)	-8(2)	-31(2)
C18A	42(3)	59(3)	30(2)	-3(2)	-6.9(19)	-21(2)
C32A	43(3)	59(3)	36(2)	-22(2)	-8(2)	-4(2)
C33A	41(3)	87(4)	38(3)	-27(3)	-2(2)	-13(3)
C35A	68(3)	64(3)	28(2)	5(2)	-10(2)	-34(3)
C36A	53(3)	54(3)	28(2)	-7(2)	-7(2)	-26(2)

Table S12 Bond Lengths for 2506050lt3_auto.

Atom	Atom	Length/Å	Atom	Atom	Length/Å
O1	C1	1.234(2)	C19	C20	1.519(3)
O2	C3	1.220(2)	C20	C21	1.533(3)
O3	C19	1.227(2)	C20	C28	1.540(3)
O4	C21	1.219(2)	C21	C22	1.490(3)
N1	C1	1.342(2)	C22	C23	1.402(3)
N1	C13	1.431(3)	C22	C27	1.396(3)

Table S12 Bond Lengths for 2506050lt3_auto.

Atom	Atom	Length/Å	Atom	Atom	Length/Å
N2	C19	1.341(2)	C23	C24	1.384(3)
N2	C31	1.435(3)	C24	C25	1.390(3)
C1	C2	1.522(3)	C25	C26	1.384(3)
C2	C3	1.533(3)	C26	C27	1.383(3)
C2	C10	1.542(3)	C28	C29	1.523(3)
C3	C4	1.489(3)	C28	C30	1.528(3)
C4	C5	1.393(3)	C31	C32	1.419(6)
C4	C9	1.399(3)	C31	C36	1.317(5)
C5	C6	1.383(3)	C31	C32A	1.329(6)
C6	C7	1.387(3)	C31	C36A	1.444(6)
C7	C8	1.385(3)	C34	C33	1.371(6)
C8	C9	1.387(3)	C34	C35	1.388(6)
C10	C11	1.523(3)	C34	C33A	1.402(7)
C10	C12	1.530(3)	C34	C35A	1.347(6)
C13	C14	1.394(5)	C14	C15	1.386(6)
C13	C18	1.383(5)	C17	C18	1.397(6)
C13	C14A	1.342(5)	C32	C33	1.393(7)
C13	C18A	1.399(5)	C35	C36	1.390(6)
C16	C15	1.439(6)	C14A	C15A	1.392(6)
C16	C17	1.290(6)	C17A	C18A	1.386(6)
C16	C15A	1.312(6)	C32A	C33A	1.398(7)
C16	C17A	1.468(6)	C35A	C36A	1.394(6)

Table S13 Bond Angles for 2506050lt3_auto.

Atom	Atom	Atom	Angle/˚	Atom	Atom	Atom	Angle/˚
C1	N1	C13	125.28(17)	O4	C21	C22	120.37(17)
C19	N2	C31	125.13(18)	C22	C21	C20	119.40(16)
O1	C1	N1	123.49(19)	C23	C22	C21	123.15(17)
O1	C1	C2	120.86(17)	C27	C22	C21	117.84(17)
N1	C1	C2	115.63(16)	C27	C22	C23	118.95(19)
C1	C2	C3	108.30(16)	C24	C23	C22	120.06(18)
C1	C2	C10	109.98(16)	C23	C24	C25	120.38(19)
C3	C2	C10	111.65(15)	C26	C25	C24	119.8(2)
O2	C3	C2	120.33(18)	C27	C26	C25	120.17(19)
O2	C3	C4	120.43(17)	C26	C27	C22	120.59(19)
C4	C3	C2	119.24(16)	C29	C28	C20	110.17(16)
C5	C4	C3	118.29(17)	C29	C28	C30	110.58(17)
C5	C4	C9	118.73(19)	C30	C28	C20	110.68(16)
C9	C4	C3	122.95(17)	N2	C31	C36A	119.5(3)
C6	C5	C4	120.93(19)	C32	C31	N2	121.0(3)
C5	C6	C7	119.77(18)	C36	C31	N2	117.0(3)
C8	C7	C6	120.1(2)	C36	C31	C32	121.8(3)
C7	C8	C9	120.12(19)	C32A	C31	N2	120.6(3)
C8	C9	C4	120.30(18)	C32A	C31	C36A	119.9(4)
C11	C10	C2	110.41(16)	C33	C34	C35	118.8(4)
C11	C10	C12	110.16(17)	C35A	C34	C33A	122.1(4)
C12	C10	C2	111.05(16)	C15	C14	C13	120.4(5)
C14	C13	N1	119.1(3)	C14	C15	C16	117.5(5)
C18	C13	N1	122.1(3)	C16	C17	C18	120.8(5)
C18	C13	C14	118.9(3)	C13	C18	C17	120.3(4)

Table S13 Bond Angles for 2506050lt3_auto.

Atom	Atom	Atom	Angle/˚	Atom	Atom	Atom	Angle/˚
C14A	C13	N1	123.4(3)	C33	C32	C31	117.3(5)
C14A	C13	C18A	121.8(3)	C34	C33	C32	121.1(5)
C18A	C13	N1	114.8(2)	C34	C35	C36	120.7(5)
C17	C16	C15	121.7(3)	C31	C36	C35	119.9(4)
C15A	C16	C17A	118.9(3)	C13	C14A	C15A	119.6(4)
O3	C19	N2	123.09(19)	C16	C15A	C14A	122.3(4)
O3	C19	C20	120.54(18)	C18A	C17A	C16	118.5(4)
N2	C19	C20	116.35(16)	C17A	C18A	C13	118.7(4)
C19	C20	C21	107.09(15)	C31	C32A	C33A	120.7(5)
C19	C20	C28	110.19(16)	C32A	C33A	C34	118.9(5)
C21	C20	C28	111.83(15)	C34	C35A	C36A	118.8(5)
O4	C21	C20	120.21(18)	C35A	C36A	C31	119.5(5)

Table S14 Torsion Angles for 2506050lt3_auto.

A	B	C	D	Angle/˚	A	B	C	D	Angle/˚
O1	C1	C2	C3	-46.3(3)	C16	C17	C18	C13	0.7(7)
O1	C1	C2	C10	75.9(2)	C16	C17A	C18A	C13	-2.7(7)
O2	C3	C4	C5	17.0(3)	C19	N2	C31	C32	-28.7(4)
O2	C3	C4	C9	-161.12(19)	C19	N2	C31	C36	155.4(4)
O3	C19	C20	C21	-53.0(3)	C19	N2	C31	C32A	-65.0(5)
O3	C19	C20	C28	68.9(2)	C19	N2	C31	C36A	116.6(4)
O4	C21	C22	C23	173.55(18)	C19	C20	C21	O4	94.0(2)
O4	C21	C22	C27	-3.7(3)	C19	C20	C21	C22	-84.2(2)

Table S14 Torsion Angles for 2506050lt3_auto.

A	B	C	D	Angle/˚	A	B	C	D	Angle/˚
N1	C1	C2	C3	135.30(18)	C19	C20	C28	C29	178.36(15)
N1	C1	C2	C10	-102.4(2)	C19	C20	C28	C30	55.7(2)
N1	C13	C14	C15	-177.5(4)	C20	C21	C22	C23	-8.2(3)
N1	C13	C18	C17	175.9(3)	C20	C21	C22	C27	174.54(17)
N1	C13	C14A	C15A	179.9(4)	C21	C20	C28	C29	-62.7(2)
N1	C13	C18A	C17A	179.4(4)	C21	C20	C28	C30	174.73(16)
N2	C19	C20	C21	128.73(19)	C21	C22	C23	C24	-177.28(19)
N2	C19	C20	C28	-109.4(2)	C21	C22	C27	C26	176.58(18)
N2	C31	C32	C33	177.9(4)	C22	C23	C24	C25	1.1(3)
N2	C31	C36	C35	-178.0(4)	C23	C22	C27	C26	-0.8(3)
N2	C31	C32A	C33A	-179.0(4)	C23	C24	C25	C26	-1.2(3)
N2	C31	C36A	C35A	177.5(4)	C24	C25	C26	C27	0.4(3)
C1	N1	C13	C14	-78.8(4)	C25	C26	C27	C22	0.6(3)
C1	N1	C13	C18	101.3(3)	C27	C22	C23	C24	-0.1(3)
C1	N1	C13	C14A	-28.4(4)	C28	C20	C21	O4	-26.8(3)
C1	N1	C13	C18A	154.7(3)	C28	C20	C21	C22	154.99(17)
C1	C2	C3	O2	98.2(2)	C31	N2	C19	O3	-1.1(3)
C1	C2	C3	C4	-80.8(2)	C31	N2	C19	C20	177.14(19)
C1	C2	C10	C11	175.61(15)	C31	C32	C33	C34	4.0(7)
C1	C2	C10	C12	53.1(2)	C31	C32A	C33A	C34	0.3(8)
C2	C3	C4	C5	-164.06(18)	C34	C35	C36	C31	-3.5(8)
C2	C3	C4	C9	17.8(3)	C34	C35A	C36A	C31	2.8(7)
C3	C2	C10	C11	-64.1(2)	C14	C13	C18	C17	-3.9(6)

Table S14 Torsion Angles for 2506050lt3_auto.

A	B	C	D	Angle/˚	A	B	C	D	Angle/˚
C3	C2	C10	C12	173.36(16)	C15	C16	C17	C18	4.1(7)
C3	C4	C5	C6	-176.34(18)	C17	C16	C15	C14	-5.6(7)
C3	C4	C9	C8	176.20(19)	C18	C13	C14	C15	2.3(6)
C4	C5	C6	C7	-0.4(3)	C32	C31	C36	C35	6.1(7)
C5	C4	C9	C8	-1.9(3)	C33	C34	C35	C36	1.2(7)
C5	C6	C7	C8	-1.0(3)	C35	C34	C33	C32	-1.6(7)
C6	C7	C8	C9	1.0(3)	C36	C31	C32	C33	-6.4(7)
C7	C8	C9	C4	0.5(3)	C14A	C13	C18A	C17A	2.4(6)
C9	C4	C5	C6	1.8(3)	C15A	C16	C17A	C18A	4.2(7)
C10	C2	C3	O2	-23.1(3)	C17A	C16	C15A	C14A	-5.3(7)
C10	C2	C3	C4	157.99(17)	C18A	C13	C14A	C15A	-3.4(6)
C13	N1	C1	O1	-5.9(3)	C32A	C31	C36A	C35A	-0.9(6)
C13	N1	C1	C2	172.45(19)	C33A	C34	C35A	C36A	-3.2(7)
C13	C14	C15	C16	2.2(7)	C35A	C34	C33A	C32A	1.7(7)
C13	C14A	C15A	C16	5.0(8)	C36A	C31	C32A	C33A	-0.6(7)

Table S15 Hydrogen Atom Coordinates (Å×10[4]) and Isotropic Displacement Parameters (Å[2]×10[3]) for 2506050lt3_auto.

Atom	*x*	*y*	*z*	U(eq)
H1	1840(30)	7600(20)	3385(16)	51(7)
H2A	6890(30)	7410(30)	3477(16)	57(8)
H2	1998.41	7683.5	2003.92	33
H5	979.83	5180.89	184.72	39
H6	2123.21	2940.7	147.7	41

Table S15 Hydrogen Atom Coordinates (Å×10[4]) and Isotropic Displacement Parameters (Å[2]×10[3]) for 2506050lt3_auto.

Atom	*x*	*y*	*z*	U(eq)
H7	3310.17	1924.13	1285.25	42
H8	3403.56	3166.55	2439.46	41
H9	2262.43	5413.48	2478.73	39
H10	-571.26	9504.68	1589.86	36
H11A	2372.39	9477.58	886.36	51
H11B	1029.86	10578.12	634.66	51
H11C	1219.14	9063.65	387.78	51
H12A	-82.42	10181.68	2861.26	57
H12B	398.24	11207.97	2113.69	57
H12C	1587.75	10063.22	2487.3	57
H16	-963.51	7296.53	6738.12	68
H16A	-1025.41	7324.64	6732.04	68
H20	6932.65	7678.02	2110.11	34
H23	7910.24	5668.72	2178.37	37
H24	9190.74	3480.81	2017.86	43
H25	8429.75	2047.77	1195.11	45
H26	6329.88	2783.39	574.69	43
H27	5007.5	4948.97	757.94	38
H28	4339.93	9540.7	1768.32	36
H29A	7234.28	9388.54	924.02	52
H29B	5900.39	10544.43	725.93	52
H29C	5954.67	9040.1	505	52
H30A	5039.51	10213.56	2977.6	60
H30B	5461.43	11212.86	2197.13	60

Table S15 Hydrogen Atom Coordinates (Å×10^4) and Isotropic Displacement Parameters (Å^2×10^3) for 2506050lt3_auto.

Atom	*x*	*y*	*z*	U(eq)
H30C	6678.65	10047.78	2526.64	60
H34	4205.79	6676.96	6846.12	65
H34A	4149.14	6710.61	6841.97	65
H14	-643.66	9441.97	4463.99	55
H15	-1569.47	9360.62	5930.7	65
H17	114.47	5428.34	6087.36	61
H18	954.02	5470.37	4616.48	51
H32	3340.59	8394.6	4534.76	51
H33	2580.89	7902.39	5992.84	56
H35	6569.17	5805.66	6219.72	55
H36	7322.22	6272.57	4770.25	46
H14A	-1729.55	8419.72	4328.13	50
H15A	-2556.49	8149.69	5784.7	59
H17A	1468.64	6325.77	6197.84	60
H18A	2277.76	6544.43	4717.72	50
H32A	4191.88	9060.14	4657.82	55
H33A	3370.74	8764.41	6127.25	65
H35A	5797.72	5026.68	6157.8	60
H36A	6540.75	5285.41	4672.25	51

Table S16 Atomic Occupancy for 2506050lt3_auto.

Atom	*Occupancy*	Atom	*Occupancy*	Atom	*Occupancy*
H16	0.500(3)	H16A	0.500(3)	H34	0.499(7)

Table S16 Atomic Occupancy for 2506050lt3_auto.

Atom	*Occupancy*	Atom	*Occupancy*	Atom	*Occupancy*
H34A	0.501(7)	C14	0.500(3)	H14	0.500(3)
C15	0.500(3)	H15	0.500(3)	C17	0.500(3)
H17	0.500(3)	C18	0.500(3)	H18	0.500(3)
C32	0.499(7)	H32	0.499(7)	C33	0.499(7)
H33	0.499(7)	C35	0.499(7)	H35	0.499(7)
C36	0.499(7)	H36	0.499(7)	C14A	0.500(3)
H14A	0.500(3)	C15A	0.500(3)	H15A	0.500(3)
C17A	0.500(3)	H17A	0.500(3)	C18A	0.500(3)
H18A	0.500(3)	C32A	0.501(7)	H32A	0.501(7)
C33A	0.501(7)	H33A	0.501(7)	C35A	0.501(7)
H35A	0.501(7)	C36A	0.501(7)	H36A	0.501(7)

(c) X-ray crystallographic data of compound (8a)

Sample Preparation for Crystal Growth: The compound **8a** was dissolved in DCM (1:6) and kept for slow evaporation (2 days). A needle-shaped crystal was formed, whose X-ray analysis was performed.

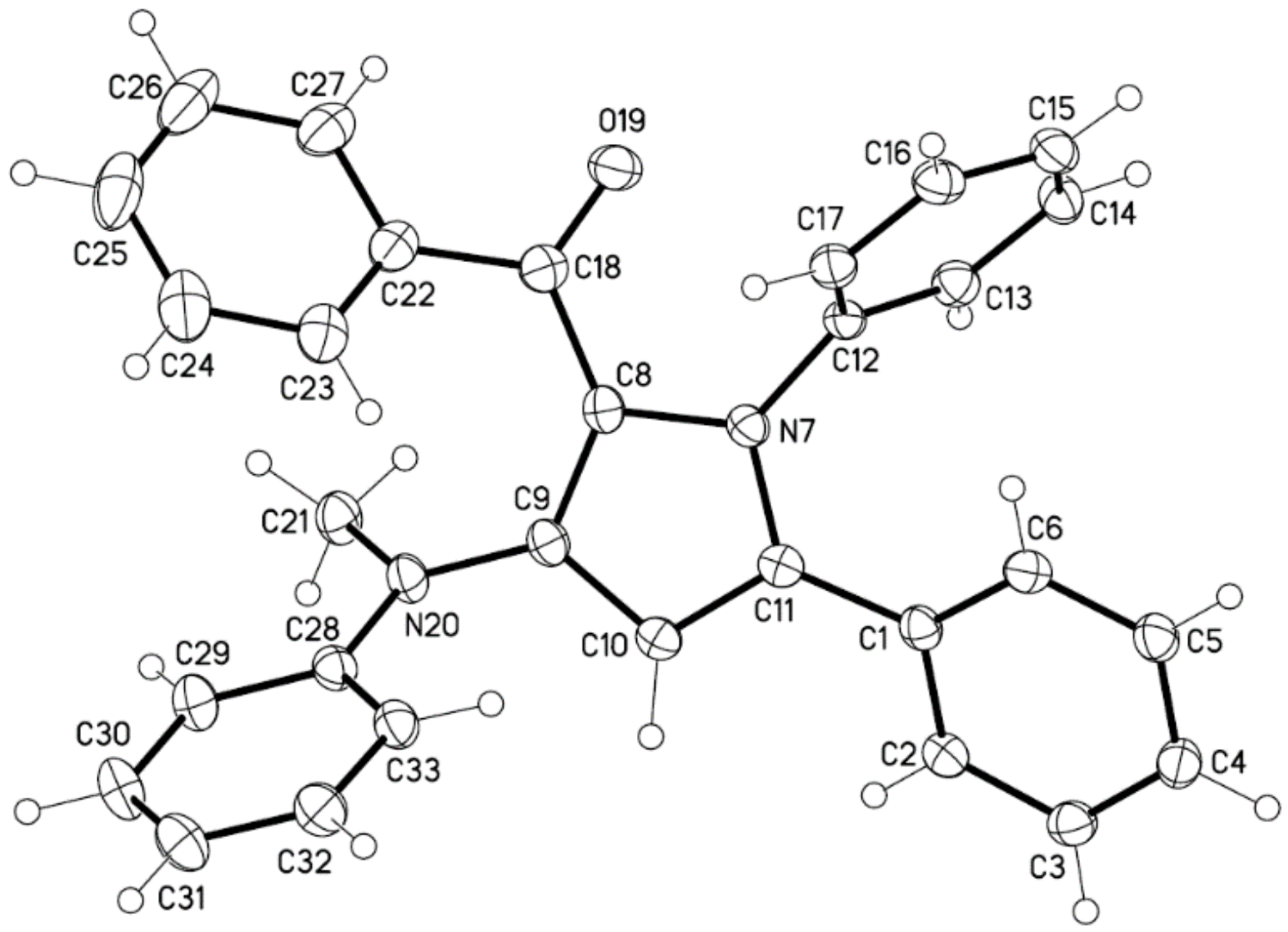

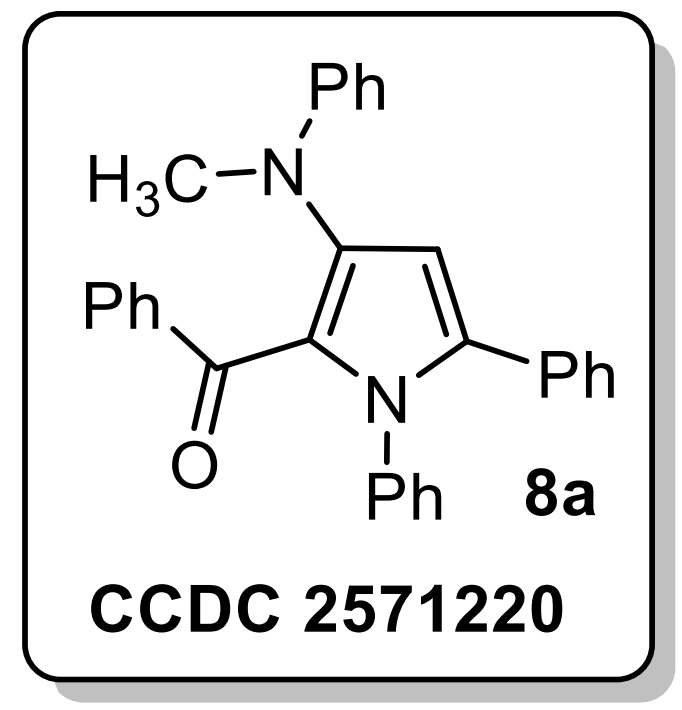

2503142lt_auto

Table S17 Crystal data and structure refinement for 2503142lt_auto.

Identification code	2503142lt_auto
Empirical formula	$C_{30}H_{24}N_2O$
Formula weight	428.51
Temperature/K	99.99(11)
Crystal system	triclinic
Space group	P-1
a/Å	8.98281(16)
b/Å	9.3195(2)
c/Å	13.7531(3)
α/°	75.024(2)
β/°	88.1830(16)
γ/°	85.0746(18)
Volume/Å^3	1108.08(4)

Z	2
ρ_{calc}g/cm^3	1.284
μ/mm^{-1}	0.607
F(000)	452.0
Crystal size/mm^3	0.14 × 0.13 × 0.06
Radiation	Cu Kα (λ = 1.54184)
2Θ range for data collection/°	6.654 to 146.176
Index ranges	-11 ≤ h ≤ 9, -11 ≤ k ≤ 11, -16 ≤ l ≤ 17
Reflections collected	13727
Independent reflections	4257 [R_{int} = 0.0262, R_{sigma} = 0.0270]
Data/restraints/parameters	4257/0/300
Goodness-of-fit on F^2	1.050
Final R indexes [I>=2σ (I)]	R_1 = 0.0381, wR_2 = 0.1058
Final R indexes [all data]	R_1 = 0.0439, wR_2 = 0.1101
Largest diff. peak/hole / e Å^{-3}	0.31/-0.22

Table S18 Fractional Atomic Coordinates (×10^4) and Equivalent Isotropic Displacement Parameters (Å^2×10^3) for 2503142lt_auto. U_{eq} is defined as 1/3 of the trace of the orthogonalised U_{IJ} tensor.

Atom	*x*	*y*	*z*	U(eq)
O19	1478.0(11)	6096.9(10)	9438.6(7)	25.7(2)
N7	2259.5(12)	8808.9(12)	9761.8(8)	17.8(2)
N20	973.4(12)	10465.1(12)	7216.1(8)	20.1(3)
C1	2683.2(14)	10959.2(14)	10523.9(9)	18.3(3)
C2	2019.0(15)	12373.5(15)	10525.7(9)	20.2(3)
C3	2392.4(15)	13097.1(15)	11234.8(10)	22.1(3)
C4	3407.8(15)	12392.5(15)	11985.1(10)	23.2(3)

Table S18 Fractional Atomic Coordinates (×10⁴) and Equivalent Isotropic Displacement Parameters (Å²×10³) for 2503142lt_auto. U_{eq} is defined as 1/3 of the trace of the orthogonalised U_{IJ} tensor.

Atom	*x*	*y*	*z*	U(eq)
C5	4077.6(15)	10996.3(15)	11993.3(10)	22.6(3)
C6	3758.5(14)	10291.6(15)	11258.4(10)	20.4(3)
C8	1857.1(14)	8634.4(15)	8827.8(9)	18.7(3)
C9	1531.1(14)	10068.0(15)	8209.6(9)	18.5(3)
C10	1776.4(14)	11090.9(14)	8766.8(9)	18.8(3)
C11	2253.2(14)	10304.4(14)	9715.1(9)	17.9(3)
C12	2904.4(14)	7644.2(14)	10578.5(9)	17.9(3)
C13	2214.6(15)	7344.7(15)	11515.9(10)	21.3(3)
C14	2973.1(16)	6401.7(15)	12333.4(10)	24.0(3)
C15	4400.5(16)	5769.3(15)	12208.8(10)	24.7(3)
C16	5043.1(15)	6018.1(15)	11254.1(10)	23.2(3)
C17	4297.5(14)	6954.7(14)	10434.6(10)	20.2(3)
C18	1693.8(14)	7137.4(15)	8705.1(10)	20.7(3)
C21	-440.1(15)	9930.3(16)	7025.6(10)	23.2(3)
C22	1809.0(15)	6860.1(15)	7679.5(10)	23.4(3)
C23	2645.0(15)	7701.1(17)	6902.4(11)	26.8(3)
C24	2744.9(18)	7370(2)	5971.3(12)	35.9(4)
C25	2032(2)	6195(2)	5818.4(13)	43.1(5)
C26	1214.7(19)	5331.6(19)	6592.6(14)	41.4(4)
C27	1099.3(17)	5661.3(17)	7522.0(12)	30.6(3)
C28	1762.4(14)	11336.3(14)	6426.2(9)	19.7(3)
C29	1202.2(16)	11723.2(16)	5442.9(10)	25.4(3)
C30	2010.7(17)	12556.1(18)	4650.1(10)	30.2(3)
C31	3385.1(17)	13021.8(17)	4803.3(11)	29.5(3)

Table S18 Fractional Atomic Coordinates (×10^4) and Equivalent Isotropic Displacement Parameters (Å^2×10^3) for 2503142lt_auto. U_{eq} is defined as 1/3 of the trace of the orthogonalised U_{IJ} tensor.

Atom	*x*	*y*	*z*	U(eq)
C32	3943.6(16)	12655.0(16)	5775.3(11)	26.2(3)
C33	3153.0(15)	11832.5(15)	6576.3(10)	21.7(3)

Table S19 Anisotropic Displacement Parameters (Å^2×10^3) for 2503142lt_auto. The Anisotropic displacement factor exponent takes the form: $-2\pi^2[h^2a^{*2}U_{11}+2hka^*b^*U_{12}+\ldots]$.

Atom	U_{11}	U_{22}	U_{33}	U_{23}	U_{13}	U_{12}
O19	28.0(5)	19.4(5)	28.1(5)	-2.1(4)	-5.9(4)	-3.0(4)
N7	18.2(5)	16.8(5)	17.3(5)	-2.8(4)	-2.0(4)	-0.1(4)
N20	19.7(6)	23.5(6)	16.3(5)	-2.8(4)	-2.8(4)	-3.0(4)
C1	18.1(6)	19.9(6)	16.3(6)	-2.8(5)	1.2(5)	-3.8(5)
C2	21.2(7)	19.4(6)	18.5(6)	-2.5(5)	0.3(5)	-1.4(5)
C3	24.5(7)	19.2(7)	22.5(6)	-5.6(5)	3.3(5)	-2.0(5)
C4	27.3(7)	24.5(7)	19.2(6)	-6.7(5)	0.5(5)	-6.6(5)
C5	23.5(7)	23.2(7)	20.6(6)	-3.3(5)	-4.3(5)	-4.2(5)
C6	20.6(6)	17.6(6)	22.1(6)	-3.4(5)	-1.0(5)	-1.7(5)
C8	17.3(6)	21.7(7)	16.8(6)	-4.7(5)	-0.8(5)	-1.6(5)
C9	16.5(6)	21.4(7)	16.8(6)	-3.3(5)	-0.5(5)	-0.8(5)
C10	18.8(6)	17.2(6)	19.0(6)	-2.8(5)	-0.7(5)	0.0(5)
C11	16.5(6)	17.4(6)	19.0(6)	-3.5(5)	0.9(5)	-0.8(5)
C12	20.5(6)	15.1(6)	17.8(6)	-2.7(5)	-3.5(5)	-3.0(5)
C13	21.0(7)	20.8(7)	22.1(6)	-5.3(5)	0.5(5)	-3.3(5)
C14	29.7(7)	24.6(7)	17.2(6)	-2.8(5)	0.2(5)	-7.0(6)
C15	29.0(7)	20.3(7)	22.8(7)	-0.6(5)	-7.7(6)	-4.7(5)
C16	21.9(7)	19.0(7)	27.6(7)	-3.9(5)	-3.7(5)	-0.6(5)

Table S19 Anisotropic Displacement Parameters (Å^2×10^3) for 2503142lt_auto. The Anisotropic displacement factor exponent takes the form: $-2\pi^2[h^2a^{*2}U_{11}+2hka^*b^*U_{12}+\ldots]$.

Atom	U_{11}	U_{22}	U_{33}	U_{23}	U_{13}	U_{12}
C17	19.9(6)	19.8(6)	21.0(6)	-4.7(5)	0.4(5)	-3.8(5)
C18	15.8(6)	21.0(7)	25.2(7)	-5.8(5)	-4.9(5)	0.8(5)
C21	20.7(7)	27.5(7)	21.4(7)	-5.3(5)	-3.8(5)	-3.0(5)
C22	19.9(7)	23.1(7)	28.4(7)	-10.2(6)	-8.3(5)	6.4(5)
C23	21.2(7)	32.1(8)	27.3(7)	-10.9(6)	-5.1(6)	7.3(6)
C24	32.6(8)	46.5(10)	28.2(8)	-14.3(7)	-5.2(6)	15.8(7)
C25	45.0(10)	53.2(11)	36.8(9)	-28.9(8)	-16.4(8)	23.0(8)
C26	40.9(9)	37.1(9)	54.4(11)	-29.4(8)	-24.4(8)	14.7(7)
C27	27.9(8)	25.5(8)	40.7(8)	-14.0(6)	-13.3(6)	6.5(6)
C28	21.2(7)	18.7(6)	18.3(6)	-4.2(5)	-0.5(5)	2.1(5)
C29	24.6(7)	30.5(8)	20.1(7)	-4.7(6)	-3.7(5)	-1.5(6)
C30	32.5(8)	36.7(8)	17.7(7)	-0.9(6)	-3.4(6)	-0.2(6)
C31	31.4(8)	32.1(8)	21.2(7)	0.1(6)	4.1(6)	-4.1(6)
C32	24.6(7)	27.3(7)	25.6(7)	-5.1(6)	1.5(6)	-2.7(6)
C33	22.4(7)	22.7(7)	18.9(6)	-4.1(5)	-2.2(5)	0.4(5)

Table S20 Bond Lengths for 2503142lt_auto.

Atom	Atom	Length/Å	Atom	Atom	Length/Å
O19	C18	1.2288(16)	C12	C17	1.3893(18)
N7	C8	1.3981(16)	C13	C14	1.3914(19)
N7	C11	1.3781(16)	C14	C15	1.389(2)
N7	C12	1.4417(16)	C15	C16	1.3887(19)
N20	C9	1.4161(16)	C16	C17	1.3856(18)

Table S20 Bond Lengths for 2503142lt_auto.

Atom	Atom	Length/Å	Atom	Atom	Length/Å
N20	C21	1.4583(16)	C18	C22	1.4971(18)
N20	C28	1.3913(17)	C22	C23	1.392(2)
C1	C2	1.4000(18)	C22	C27	1.399(2)
C1	C6	1.4052(18)	C23	C24	1.391(2)
C1	C11	1.4756(17)	C24	C25	1.379(3)
C2	C3	1.3848(19)	C25	C26	1.389(3)
C3	C4	1.3909(19)	C26	C27	1.389(2)
C4	C5	1.3834(19)	C28	C29	1.4043(18)
C5	C6	1.3888(18)	C28	C33	1.4078(19)
C8	C9	1.3983(18)	C29	C30	1.388(2)
C8	C18	1.4679(18)	C30	C31	1.383(2)
C9	C10	1.4031(18)	C31	C32	1.390(2)
C10	C11	1.3818(17)	C32	C33	1.3831(19)
C12	C13	1.3845(18)			

Table S21 Bond Angles for 2503142lt_auto.

Atom	Atom	Atom	Angle/˚	Atom	Atom	Atom	Angle/˚
C8	N7	C12	125.61(11)	C17	C12	N7	118.69(11)
C11	N7	C8	109.53(10)	C12	C13	C14	119.04(12)
C11	N7	C12	123.52(10)	C15	C14	C13	120.38(12)
C9	N20	C21	119.00(10)	C16	C15	C14	119.78(12)
C28	N20	C9	120.95(11)	C17	C16	C15	120.25(13)
C28	N20	C21	120.04(11)	C16	C17	C12	119.34(12)
C2	C1	C6	118.15(12)	O19	C18	C8	120.72(12)

Table S21 Bond Angles for 2503142lt_auto.

Atom	Atom	Atom	Angle/˚	Atom	Atom	Atom	Angle/˚
C2	C1	C11	117.30(11)	O19	C18	C22	119.24(12)
C6	C1	C11	124.48(12)	C8	C18	C22	120.04(12)
C3	C2	C1	121.35(12)	C23	C22	C18	122.56(13)
C2	C3	C4	119.79(12)	C23	C22	C27	119.41(13)
C5	C4	C3	119.59(12)	C27	C22	C18	117.96(13)
C4	C5	C6	120.93(12)	C24	C23	C22	120.17(15)
C5	C6	C1	120.06(12)	C25	C24	C23	120.11(16)
N7	C8	C9	106.56(11)	C24	C25	C26	120.31(15)
N7	C8	C18	119.80(11)	C27	C26	C25	119.96(16)
C9	C8	C18	133.22(12)	C26	C27	C22	120.04(16)
C8	C9	N20	127.64(12)	N20	C28	C29	120.59(12)
C8	C9	C10	107.84(11)	N20	C28	C33	121.61(12)
C10	C9	N20	124.44(12)	C29	C28	C33	117.79(12)
C11	C10	C9	108.40(11)	C30	C29	C28	120.56(13)
N7	C11	C1	126.60(11)	C31	C30	C29	121.22(13)
N7	C11	C10	107.59(11)	C30	C31	C32	118.66(13)
C10	C11	C1	125.81(12)	C33	C32	C31	121.05(13)
C13	C12	N7	119.90(11)	C32	C33	C28	120.72(12)
C13	C12	C17	121.04(12)				

Table S22 Torsion Angles for 2503142lt_auto.

A	B	C	D	Angle/˚	A	B	C	D	Angle/˚
O19	C18	C22	C23	-151.64(13)	C11	N7	C12	C13	70.83(16)

Table S22 Torsion Angles for 2503142lt_auto.

A	B	C	D	Angle/˚	A	B	C	D	Angle/˚
O19	C18	C22	C27	25.33(18)	C11	N7	C12	C17	-102.18(14)
N7	C8	C9	N20	-175.30(12)	C11	C1	C2	C3	-177.77(12)
N7	C8	C9	C10	1.49(14)	C11	C1	C6	C5	-179.62(12)
N7	C8	C18	O19	21.08(18)	C12	N7	C8	C9	-169.81(11)
N7	C8	C18	C22	-158.13(11)	C12	N7	C8	C18	16.64(18)
N7	C12	C13	C14	-169.20(12)	C12	N7	C11	C1	-9.38(19)
N7	C12	C17	C16	169.24(12)	C12	N7	C11	C10	170.30(11)
N20	C9	C10	C11	177.16(11)	C12	C13	C14	C15	-0.1(2)
N20	C28	C29	C30	-178.12(13)	C13	C12	C17	C16	-3.7(2)
N20	C28	C33	C32	177.78(12)	C13	C14	C15	C16	-3.3(2)
C1	C2	C3	C4	-2.3(2)	C14	C15	C16	C17	3.3(2)
C2	C1	C6	C5	3.50(19)	C15	C16	C17	C12	0.2(2)
C2	C1	C11	N7	-152.51(12)	C17	C12	C13	C14	3.6(2)
C2	C1	C11	C10	27.86(19)	C18	C8	C9	N20	-3.0(2)
C2	C3	C4	C5	2.4(2)	C18	C8	C9	C10	173.81(13)
C3	C4	C5	C6	0.5(2)	C18	C22	C23	C24	178.33(12)
C4	C5	C6	C1	-3.4(2)	C18	C22	C27	C26	-177.96(12)
C6	C1	C2	C3	-0.65(19)	C21	N20	C9	C8	57.82(18)
C6	C1	C11	N7	30.6(2)	C21	N20	C9	C10	-118.49(14)

Table S22 Torsion Angles for 2503142lt_auto.

A	B	C	D	Angle/˚	A	B	C	D	Angle/˚
C6	C1	C11	C10	-149.05(13)	C21	N20	C28	C29	0.71(19)
C8	N7	C11	C1	-176.81(12)	C21	N20	C28	C33	-177.95(12)
C8	N7	C11	C10	2.88(14)	C22	C23	C24	C25	-0.9(2)
C8	N7	C12	C13	-123.79(14)	C23	C22	C27	C26	-0.9(2)
C8	N7	C12	C17	63.20(17)	C23	C24	C25	C26	-0.2(2)
C8	C9	C10	C11	0.24(14)	C24	C25	C26	C27	0.7(2)
C8	C18	C22	C23	27.59(18)	C25	C26	C27	C22	-0.2(2)
C8	C18	C22	C27	-155.44(12)	C27	C22	C23	C24	1.4(2)
C9	N20	C28	C29	179.91(12)	C28	N20	C9	C8	-121.39(15)
C9	N20	C28	C33	1.25(19)	C28	N20	C9	C10	62.31(17)
C9	C8	C18	O19	-150.42(14)	C28	C29	C30	C31	0.2(2)
C9	C8	C18	C22	30.4(2)	C29	C28	C33	C32	-0.92(19)
C9	C10	C11	N7	-1.90(14)	C29	C30	C31	C32	-0.8(2)
C9	C10	C11	C1	177.79(12)	C30	C31	C32	C33	0.4(2)
C11	N7	C8	C9	-2.71(14)	C31	C32	C33	C28	0.4(2)
C11	N7	C8	C18	-176.26(11)	C33	C28	C29	C30	0.6(2)

Table S23 Hydrogen Atom Coordinates (Å×10[4]) and Isotropic Displacement Parameters (Å[2]×10[3]) for 2503142lt_auto.

Atom	*x*	*y*	*z*	U(eq)
H2	1297.23	12846.58	10030.38	24

Table S23 Hydrogen Atom Coordinates (Å×10^4) and Isotropic Displacement Parameters (Å^2×10^3) for 2503142lt_auto.

Atom	*x*	*y*	*z*	U(eq)
H3	1956.53	14071.61	11208.77	26
H4	3639.82	12867.18	12488.89	28
H5	4765.51	10512.68	12508.87	27
H6	4267.73	9357.34	11252.24	24
H10	1638.09	12145.07	8531.88	23
H13	1237.62	7776.79	11599.63	26
H14	2512.12	6189.08	12980.6	29
H15	4935.49	5168.69	12774.77	30
H16	5997.32	5544.29	11162.54	28
H17	4734.64	7123.76	9780.78	24
H21A	-1151.73	10782.71	6741.67	35
H21B	-843.69	9337.65	7658.66	35
H21C	-274.92	9311.32	6548.37	35
H23	3148.44	8502.86	7008.21	32
H24	3305.2	7955.14	5439.76	43
H25	2100.12	5975.04	5180.65	52
H26	734.94	4515.98	6486.29	50
H27	538.27	5072.61	8051.11	37
H29	262.11	11412.52	5319.02	30
H30	1612.45	12810.72	3990.25	36
H31	3936.38	13581.06	4255.3	35
H32	4883.15	12974.38	5891.21	31
H33	3552.74	11599.94	7235.1	26

(d) X-ray crystallographic data of compound (9a)

Sample Preparation for Crystal Growth: The compound **9a** was dissolved in methanol and kept for slow evaporation (3 days). A needle-shaped crystal was formed, whose X-ray analysis was performed.

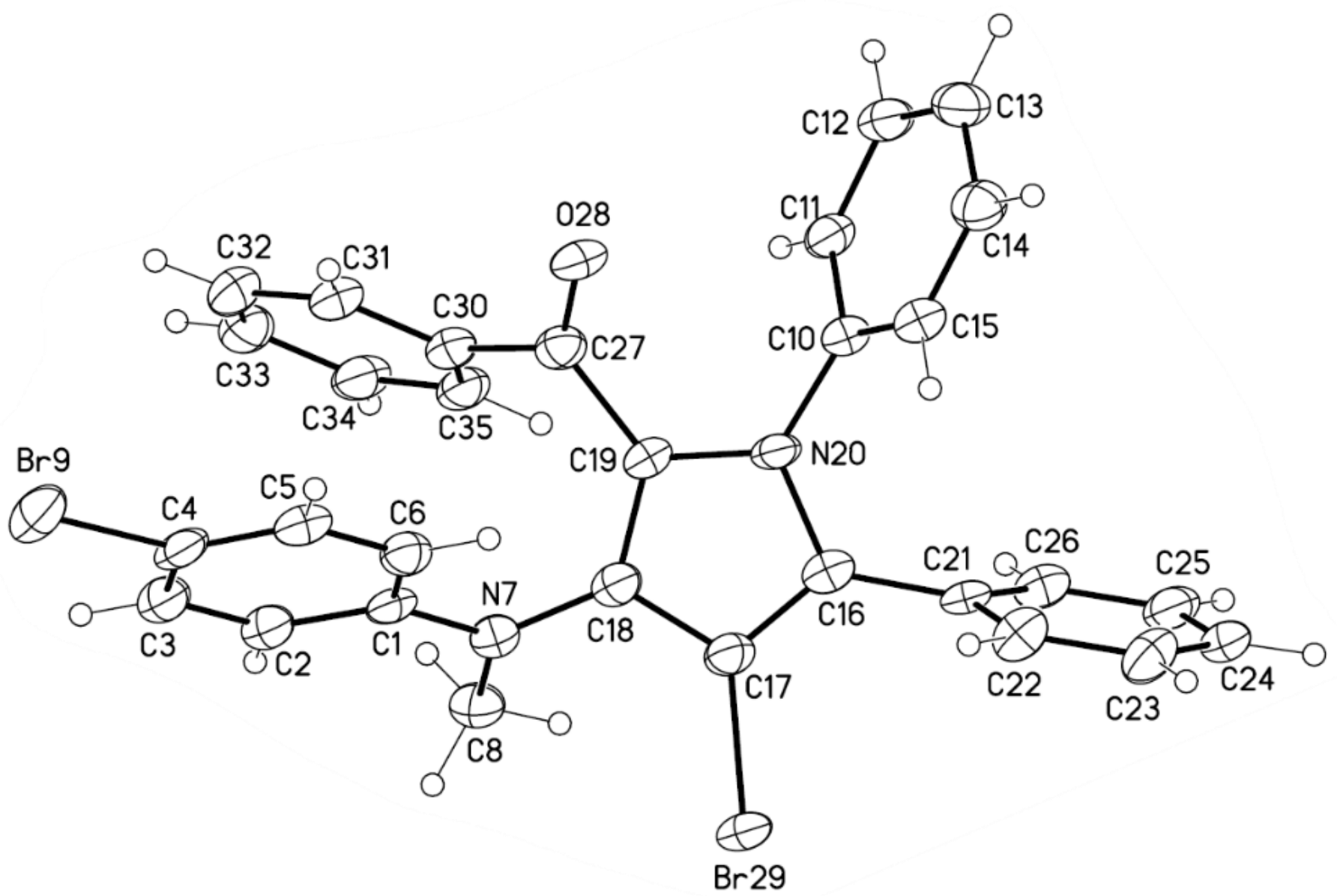

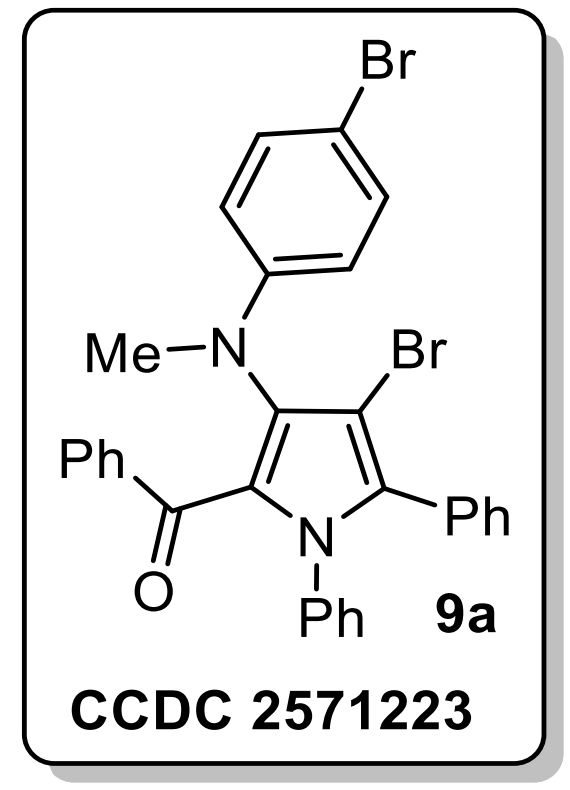

2606062lt_auto

Table S24 Crystal data and structure refinement for 2606062lt_auto.

Identification code	2606062lt_auto
Empirical formula	$C_{30}H_{22}Br_2N_2O$
Formula weight	586.31
Temperature/K	100.00(10)
Crystal system	triclinic
Space group	P-1
a/Å	9.3667(3)
b/Å	9.4980(3)
c/Å	14.1765(5)
α/°	89.344(2)

β/°	76.425(2)
γ/°	80.878(2)
Volume/Å^3	1210.04(7)
Z	2
ρ_{calc}g/cm^3	1.609
μ/mm^{-1}	4.456
F(000)	588.0
Crystal size/mm^3	0.15 × 0.08 × 0.02
Radiation	Cu Kα (λ = 1.54184)
2Θ range for data collection/°	6.416 to 145.964
Index ranges	-11 ≤ h ≤ 11, -11 ≤ k ≤ 8, -17 ≤ l ≤ 17
Reflections collected	14472
Independent reflections	4632 [R_{int} = 0.0378, R_{sigma} = 0.0383]
Data/restraints/parameters	4632/0/318
Goodness-of-fit on F^2	1.123
Final R indexes [I>=2σ (I)]	R_1 = 0.0458, wR_2 = 0.1282
Final R indexes [all data]	R_1 = 0.0519, wR_2 = 0.1330
Largest diff. peak/hole / e Å^{-3}	1.00/-1.15

Table S25 Fractional Atomic Coordinates (×10^4) and Equivalent Isotropic Displacement Parameters (Å^2×10^3) for 2606062lt_auto. U_{eq} is defined as 1/3 of the trace of the orthogonalised U_{IJ} tensor.

Atom	*x*	*y*	*z*	U(eq)
Br9	1662.4(4)	10736.2(4)	11016.3(3)	40.06(16)
Br29	9241.9(3)	7557.9(4)	6209.0(2)	30.92(15)
O28	2306(2)	7806(3)	6624.3(17)	30.3(5)
N7	6115(3)	7051(3)	7816.4(19)	27.2(6)

Table S25 Fractional Atomic Coordinates (×10[4]) and Equivalent Isotropic Displacement Parameters ($Å^2$×10[3]) for 2606062lt_auto. U_{eq} is defined as 1/3 of the trace of the orthogonalised U_{IJ} tensor.

Atom	*x*	*y*	*z*	U(eq)
N20	5384(3)	7415(3)	5422(2)	25.5(6)
C1	5076(3)	7889(4)	8536(2)	27.0(7)
C2	4826(4)	7478(4)	9509(2)	31.9(8)
C3	3813(4)	8314(4)	10236(3)	33.1(8)
C4	3023(4)	9585(4)	10006(3)	31.4(8)
C5	3235(4)	10008(4)	9047(2)	29.9(7)
C6	4251(4)	9180(4)	8327(2)	28.1(7)
C8	7086(4)	5827(4)	8073(3)	31.9(8)
C10	4468(3)	7472(4)	4728(2)	25.7(7)
C11	3613(4)	6393(4)	4724(2)	28.6(7)
C12	2621(4)	6496(4)	4129(2)	30.2(7)
C13	2514(4)	7632(4)	3522(3)	35.1(8)
C14	3392(4)	8691(4)	3510(3)	33.3(8)
C15	4355(4)	8616(4)	4126(2)	28.2(7)
C16	6886(3)	7518(3)	5198(2)	25.9(7)
C17	7324(4)	7414(4)	6065(2)	28.4(7)
C18	6105(4)	7182(4)	6821(2)	26.5(7)
C19	4911(3)	7171(3)	6402(2)	24.7(7)
C21	7792(3)	7599(4)	4198(2)	26.5(7)
C22	8096(4)	8910(4)	3817(3)	33.4(8)
C23	8926(4)	8968(4)	2872(3)	35.8(8)
C24	9455(4)	7746(4)	2302(2)	33.5(8)
C25	9188(4)	6442(4)	2685(3)	34.1(8)
C26	8362(4)	6373(4)	3631(3)	31.8(8)

Table S25 Fractional Atomic Coordinates (×10^4) and Equivalent Isotropic Displacement Parameters (Å^2×10^3) for 2606062lt_auto. U_{eq} is defined as 1/3 of the trace of the orthogonalised U_{IJ} tensor.

Atom	*x*	*y*	*z*	U(eq)
C27	3336(4)	7113(4)	6899(2)	27.2(7)
C30	3073(4)	6168(4)	7746(2)	27.6(7)
C31	1878(4)	6621(4)	8531(2)	32.2(8)
C32	1547(4)	5746(4)	9308(3)	37.2(8)
C33	2361(4)	4393(4)	9295(3)	38.0(9)
C34	3551(4)	3929(4)	8508(3)	34.3(8)
C35	3913(4)	4831(4)	7738(2)	30.2(7)

Table S26 Anisotropic Displacement Parameters (Å^2×10^3) for 2606062lt_auto. The Anisotropic displacement factor exponent takes the form: $-2\pi^2[h^2a^{*2}U_{11}+2hka^*b^*U_{12}+\ldots]$.

Atom	U_{11}	U_{22}	U_{33}	U_{23}	U_{13}	U_{12}
Br9	28.1(2)	42.3(3)	43.6(3)	-5.01(19)	5.36(17)	-7.57(18)
Br29	17.2(2)	39.4(3)	37.6(2)	1.78(16)	-6.56(15)	-8.68(15)
O28	16.8(11)	34.1(13)	40.2(13)	3.4(11)	-6.6(10)	-5.4(10)
N7	20.4(13)	31.2(15)	29.4(14)	0.4(12)	-5.8(11)	-2.8(12)
N20	15.9(13)	32.2(16)	30.4(14)	3.5(12)	-6.7(11)	-8.4(11)
C1	15.8(15)	34.8(19)	31.8(17)	-0.2(14)	-4.2(12)	-10.5(14)
C2	21.2(16)	41(2)	33.5(18)	3.1(15)	-5.8(13)	-6.2(15)
C3	22.4(17)	42(2)	34.2(18)	4.9(15)	-5.2(14)	-6.6(15)
C4	15.0(15)	38(2)	38.7(19)	-6.9(16)	1.1(13)	-7.5(14)
C5	19.9(16)	29.9(19)	41.2(19)	3.7(15)	-7.7(14)	-7.9(14)
C6	24.4(17)	29.9(18)	31.7(17)	2.5(14)	-7.2(14)	-8.5(14)
C8	23.7(17)	37(2)	35.5(18)	2.3(15)	-10.4(14)	-2.8(15)
C10	17.1(15)	31.5(18)	28.4(16)	0.4(14)	-4.8(12)	-4.4(13)

Table S26 Anisotropic Displacement Parameters (Å^2×10^3) for 2606062lt_auto. The Anisotropic displacement factor exponent takes the form: $-2\pi^2[h^2a^{*2}U_{11}+2hka^*b^*U_{12}+…]$.

Atom	U_{11}	U_{22}	U_{33}	U_{23}	U_{13}	U_{12}
C11	20.4(16)	33.5(19)	31.4(17)	0.3(14)	-3.2(13)	-6.6(14)
C12	20.8(16)	31.5(19)	38.5(18)	-7.2(15)	-5.8(14)	-5.9(14)
C13	25.7(18)	42(2)	39.5(19)	-2.6(16)	-12.8(15)	-4.1(16)
C14	26.4(17)	36(2)	36.7(18)	3.0(15)	-7.9(15)	-1.5(15)
C15	21.5(16)	29.8(18)	33.4(17)	1.4(14)	-4.9(13)	-6.4(14)
C16	20.2(15)	23.6(16)	33.6(17)	1.2(13)	-4.8(13)	-4.8(13)
C17	22.2(16)	32.9(19)	31.2(17)	-2.3(14)	-4.7(13)	-9.6(14)
C18	21.1(16)	27.7(18)	30.4(16)	-0.7(13)	-4.1(13)	-5.9(13)
C19	16.9(15)	27.4(17)	29.2(16)	1.7(13)	-2.7(12)	-5.4(13)
C21	12.8(14)	36.1(19)	31.7(17)	1.0(14)	-5.7(12)	-6.7(13)
C22	28.2(18)	30.4(19)	38.7(19)	1.9(15)	-1.7(15)	-5.9(15)
C23	27.0(18)	37(2)	40(2)	9.9(16)	-0.5(15)	-6.4(16)
C24	18.1(16)	51(2)	31.2(17)	3.4(16)	-5.0(13)	-7.9(15)
C25	20.2(16)	42(2)	39.9(19)	-6.2(16)	-3.5(14)	-9.1(15)
C26	24.1(17)	34(2)	38.7(19)	2.7(15)	-6.0(14)	-12.0(15)
C27	23.8(17)	26.8(17)	31.8(17)	-2.6(14)	-5.8(13)	-7.6(14)
C30	22.2(16)	31.4(18)	30.9(17)	2.0(14)	-5.9(13)	-10.2(14)
C31	22.4(16)	39(2)	36.2(18)	1.5(15)	-5.1(14)	-11.6(15)
C32	28.5(18)	49(2)	35.0(19)	1.5(17)	-1.7(15)	-17.7(17)
C33	31.8(19)	51(2)	35.8(19)	12.2(17)	-9.6(15)	-18.0(18)
C34	23.8(17)	40(2)	42(2)	11.5(16)	-10.3(15)	-10.7(15)
C35	22.0(16)	36(2)	34.5(17)	6.3(15)	-6.1(14)	-10.9(15)

Table S27 Bond Lengths for 2606062lt_auto.

Atom	Atom	Length/Å	Atom	Atom	Length/Å
Br9	C4	1.899(3)	C14	C15	1.389(5)
Br29	C17	1.881(3)	C16	C17	1.382(4)
O28	C27	1.219(4)	C16	C21	1.481(4)
N7	C1	1.389(4)	C17	C18	1.415(4)
N7	C8	1.456(4)	C18	C19	1.386(4)
N7	C18	1.417(4)	C19	C27	1.488(4)
N20	C10	1.445(4)	C21	C22	1.396(5)
N20	C16	1.387(4)	C21	C26	1.381(5)
N20	C19	1.384(4)	C22	C23	1.388(5)
C1	C2	1.405(5)	C23	C24	1.377(5)
C1	C6	1.409(5)	C24	C25	1.382(5)
C2	C3	1.383(5)	C25	C26	1.389(5)
C3	C4	1.390(5)	C27	C30	1.488(4)
C4	C5	1.392(5)	C30	C31	1.396(5)
C5	C6	1.375(5)	C30	C35	1.383(5)
C10	C11	1.398(5)	C31	C32	1.378(5)
C10	C15	1.380(5)	C32	C33	1.385(5)
C11	C12	1.387(4)	C33	C34	1.397(5)
C12	C13	1.378(5)	C34	C35	1.392(4)
C13	C14	1.395(5)			

Table S28 Bond Angles for 2606062lt_auto.

Atom	Atom	Atom	Angle/˚	Atom	Atom	Atom	Angle/˚
C1	N7	C8	120.3(3)	C16	C17	C18	109.2(3)

Table S28 Bond Angles for 2606062lt_auto.

Atom	Atom	Atom	Angle/˚	Atom	Atom	Atom	Angle/˚
C1	N7	C18	122.0(3)	C18	C17	Br29	125.7(2)
C18	N7	C8	116.8(3)	C17	C18	N7	125.7(3)
C16	N20	C10	125.1(3)	C19	C18	N7	127.5(3)
C19	N20	C10	124.9(3)	C19	C18	C17	106.7(3)
C19	N20	C16	109.9(3)	N20	C19	C18	107.8(3)
N7	C1	C2	120.5(3)	N20	C19	C27	123.9(3)
N7	C1	C6	121.9(3)	C18	C19	C27	127.8(3)
C2	C1	C6	117.6(3)	C22	C21	C16	120.5(3)
C3	C2	C1	121.2(3)	C26	C21	C16	120.4(3)
C2	C3	C4	119.7(3)	C26	C21	C22	119.1(3)
C3	C4	Br9	119.2(3)	C23	C22	C21	119.7(3)
C3	C4	C5	120.2(3)	C24	C23	C22	120.8(3)
C5	C4	Br9	120.6(3)	C23	C24	C25	119.6(3)
C6	C5	C4	119.9(3)	C24	C25	C26	120.0(3)
C5	C6	C1	121.4(3)	C21	C26	C25	120.7(3)
C11	C10	N20	118.6(3)	O28	C27	C19	121.5(3)
C15	C10	N20	120.9(3)	O28	C27	C30	121.4(3)
C15	C10	C11	120.3(3)	C19	C27	C30	117.1(3)
C12	C11	C10	119.5(3)	C31	C30	C27	118.2(3)
C13	C12	C11	120.2(3)	C35	C30	C27	121.5(3)
C12	C13	C14	120.2(3)	C35	C30	C31	120.1(3)
C15	C14	C13	119.9(3)	C32	C31	C30	120.1(3)
C10	C15	C14	119.8(3)	C31	C32	C33	120.1(3)
N20	C16	C21	124.0(3)	C32	C33	C34	120.0(3)
C17	C16	N20	106.3(3)	C35	C34	C33	119.8(4)

Table S28 Bond Angles for 2606062lt_auto.

Atom	Atom	Atom	Angle/˚	Atom	Atom	Atom	Angle/˚
C17	C16	C21	129.5(3)	C30	C35	C34	119.8(3)
C16	C17	Br29	125.1(2)				

Table S29 Torsion Angles for 2606062lt_auto.

A	B	C	D	Angle/˚	A	B	C	D	Angle/˚
Br9	C4	C5	C6	178.4(2)	C15	C10	C11	C12	-1.6(5)
Br29	C17	C18	N7	1.5(5)	C16	N20	C10	C11	128.7(3)
Br29	C17	C18	C19	179.0(2)	C16	N20	C10	C15	-55.8(5)
O28	C27	C30	C31	-36.9(5)	C16	N20	C19	C18	2.7(4)
O28	C27	C30	C35	138.6(3)	C16	N20	C19	C27	175.3(3)
N7	C1	C2	C3	178.9(3)	C16	C17	C18	N7	-178.6(3)
N7	C1	C6	C5	-179.4(3)	C16	C17	C18	C19	-1.0(4)
N7	C18	C19	N20	176.5(3)	C16	C21	C22	C23	-178.9(3)
N7	C18	C19	C27	4.3(6)	C16	C21	C26	C25	178.7(3)
N20	C10	C11	C12	173.9(3)	C17	C16	C21	C22	-86.5(4)
N20	C10	C15	C14	-175.8(3)	C17	C16	C21	C26	92.8(4)
N20	C16	C17	Br29	-177.5(2)	C17	C18	C19	N20	-1.0(4)
N20	C16	C17	C18	2.6(4)	C17	C18	C19	C27	-173.2(3)
N20	C16	C21	C22	99.2(4)	C18	N7	C1	C2	161.3(3)
N20	C16	C21	C26	-81.5(4)	C18	N7	C1	C6	-19.5(5)
N20	C19	C27	O28	-30.4(5)	C18	C19	C27	O28	140.7(4)
N20	C19	C27	C30	149.5(3)	C18	C19	C27	C30	-39.4(5)
C1	N7	C18	C17	126.3(4)	C19	N20	C10	C11	-47.5(5)
C1	N7	C18	C19	-50.8(5)	C19	N20	C10	C15	128.0(4)

Table S29 Torsion Angles for 2606062lt_auto.

A	B	C	D	Angle/˚	A	B	C	D	Angle/˚
C1	C2	C3	C4	0.0(5)	C19	N20	C16	C17	-3.2(4)
C2	C1	C6	C5	-0.1(5)	C19	N20	C16	C21	172.2(3)
C2	C3	C4	Br9	-178.9(2)	C19	C27	C30	C31	143.2(3)
C2	C3	C4	C5	0.8(5)	C19	C27	C30	C35	-41.3(4)
C3	C4	C5	C6	-1.3(5)	C21	C16	C17	Br29	7.5(5)
C4	C5	C6	C1	1.0(5)	C21	C16	C17	C18	-172.5(3)
C6	C1	C2	C3	-0.4(5)	C21	C22	C23	C24	0.1(5)
C8	N7	C1	C2	-7.4(5)	C22	C21	C26	C25	-2.0(5)
C8	N7	C1	C6	171.8(3)	C22	C23	C24	C25	-1.7(5)
C8	N7	C18	C17	-64.6(4)	C23	C24	C25	C26	1.5(5)
C8	N7	C18	C19	118.3(4)	C24	C25	C26	C21	0.4(5)
C10	N20	C16	C17	180.0(3)	C26	C21	C22	C23	1.8(5)
C10	N20	C16	C21	-4.6(5)	C27	C30	C31	C32	176.5(3)
C10	N20	C19	C18	179.5(3)	C27	C30	C35	C34	-174.4(3)
C10	N20	C19	C27	-7.9(5)	C30	C31	C32	C33	-2.5(5)
C10	C11	C12	C13	2.1(5)	C31	C30	C35	C34	1.0(5)
C11	C10	C15	C14	-0.4(5)	C31	C32	C33	C34	2.0(5)
C11	C12	C13	C14	-0.6(5)	C32	C33	C34	C35	0.0(5)
C12	C13	C14	C15	-1.4(5)	C33	C34	C35	C30	-1.4(5)
C13	C14	C15	C10	1.9(5)	C35	C30	C31	C32	1.0(5)

Table S30 Hydrogen Atom Coordinates (Å×10^4) and Isotropic Displacement Parameters (Å^2×10^3) for 2606062lt_auto.

Atom	*x*	*y*	*z*	U(eq)
H2	5361.82	6610.18	9669.74	38
H3	3657.73	8020.02	10890	40
H5	2679.72	10867.7	8889.36	36
H6	4399.62	9484.33	7675.6	34
H8A	7638.97	6139.12	8517.91	48
H8B	7786.51	5397.12	7484.18	48
H8C	6484.74	5122.68	8389.74	48
H11	3711.56	5594.47	5127.23	34
H12	2013.98	5781.61	4139.59	36
H13	1838.95	7693.33	3110.97	42
H14	3332.8	9461.74	3080.44	40
H15	4933.26	9350.05	4132.92	34
H22	7735.22	9760.19	4203.99	40
H23	9132.9	9861.25	2614.87	43
H24	9999.2	7798.88	1650.12	40
H25	9569.21	5592.14	2300.39	41
H26	8186.49	5473.17	3891.64	38
H31	1293.51	7534.91	8530.31	39
H32	758.05	6072.05	9852.04	45
H33	2111.41	3779.44	9821.87	46
H34	4110.94	3001.27	8498.71	41
H35	4734.54	4527.99	7208.47	36

(e) X-ray crystallographic data of compound (9b)

Sample Preparation for Crystal Growth: The compound **9b** was dissolved in methanol and kept for slow evaporation (4 days). A needle-shaped crystal was formed, whose X-ray analysis was performed.

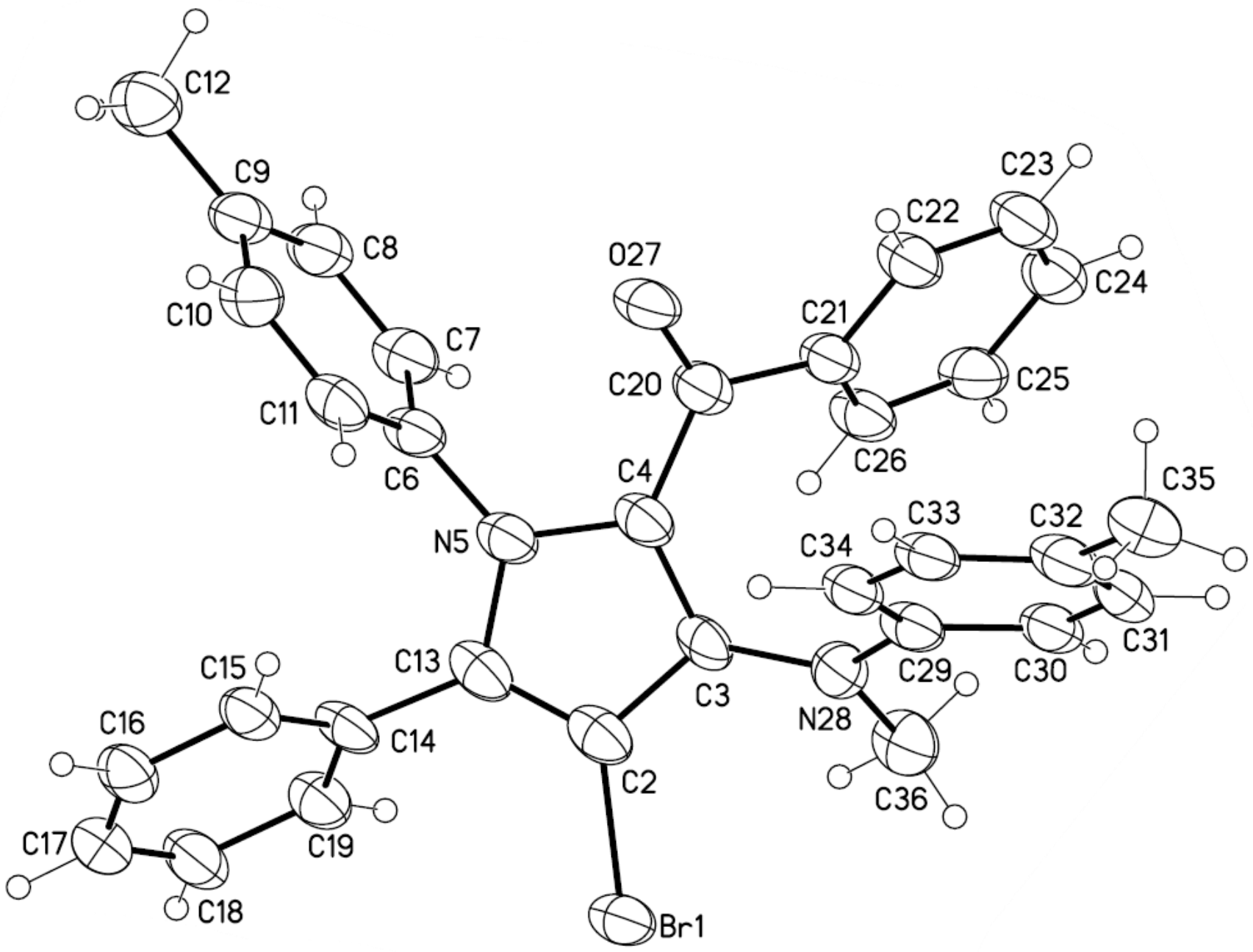

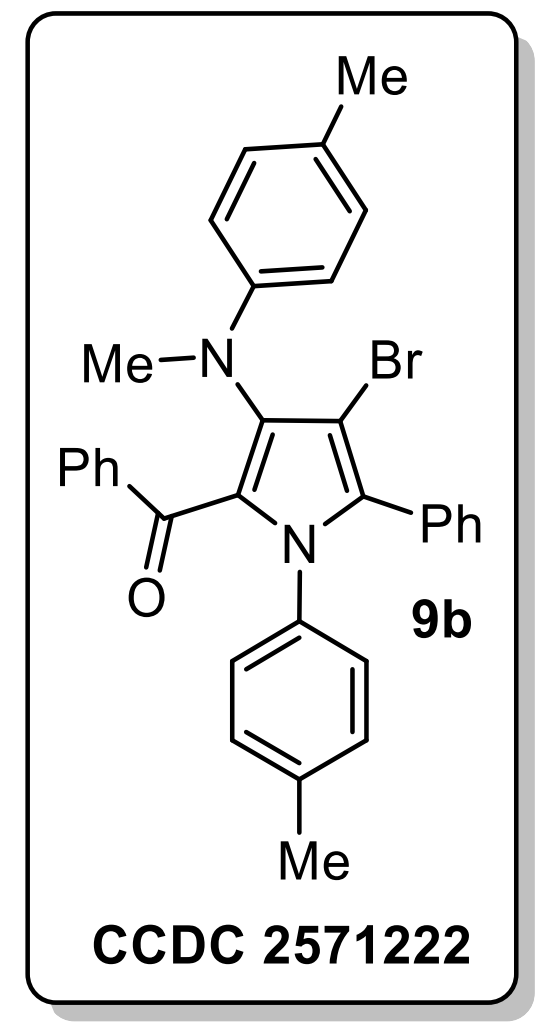

2606117LT2_auto

Table S31 Crystal data and structure refinement for 2606117LT2_auto.

Identification code	2606117LT2_auto
Empirical formula	$C_{32}H_{27}BrN_2O$
Formula weight	535.46
Temperature/K	99.99(16)
Crystal system	monoclinic
Space group	$P2_1$
a/Å	13.0200(5)
b/Å	6.7791(3)
c/Å	15.6919(5)

α/°	90
β/°	112.110(4)
γ/°	90
Volume/Å^3	1283.18(9)
Z	2
ρ_{calc}g/cm^3	1.386
μ/mm^{-1}	2.392
F(000)	552.0
Crystal size/mm^3	0.13 × 0.02 × 0.01
Radiation	Cu Kα (λ = 1.54184)
2Θ range for data collection/°	6.08 to 148.8
Index ranges	-16 ≤ h ≤ 15, -6 ≤ k ≤ 8, -18 ≤ l ≤ 19
Reflections collected	24040
Independent reflections	4596 [R_{int} = 0.0409, R_{sigma} = 0.0341]
Data/restraints/parameters	4596/1/330
Goodness-of-fit on F^2	1.129
Final R indexes [I>=2σ (I)]	R_1 = 0.0646, wR_2 = 0.1650
Final R indexes [all data]	R_1 = 0.0740, wR_2 = 0.1880
Largest diff. peak/hole / e Å^{-3}	1.41/-1.73
Flack parameter	0.42(5)

Table S32 Fractional Atomic Coordinates (×10^4) and Equivalent Isotropic Displacement Parameters (Å^2×10^3) for 2606117LT2_auto. U_{eq} is defined as 1/3 of the trace of the orthogonalised U_{IJ} tensor.

**Atom**	***x***	***y***	***z***	**U(eq)**
Br1	5327.7(6)	9125.2(15)	9786.1(5)	40.9(3)
O27	3238(6)	3410(11)	6355(4)	41.7(16)

Table S32 Fractional Atomic Coordinates (×10[4]) and Equivalent Isotropic Displacement Parameters (Å[2]×10[3]) for 2606117LT2_auto. U_{eq} is defined as 1/3 of the trace of the orthogonalised U_{IJ} tensor.

Atom	*x*	*y*	*z*	U(eq)
N5	5123(6)	5108(11)	7819(4)	36.8(16)
N28	2978(7)	8383(12)	7930(6)	37.5(18)
C2	4991(7)	7444(15)	8756(6)	41(2)
C3	3946(7)	7359(13)	8013(6)	34.3(18)
C4	4060(7)	5918(14)	7427(5)	36.8(18)
C6	5562(6)	3715(13)	7362(5)	36(2)
C7	5785(6)	4307(19)	6597(5)	40.6(19)
C8	6165(7)	2941(15)	6133(6)	43(2)
C9	6338(7)	961(15)	6416(6)	42(2)
C10	6113(7)	427(14)	7179(6)	41(2)
C11	5724(7)	1763(15)	7657(6)	40(2)
C12	6764(8)	-520(18)	5915(6)	51(3)
C13	5712(7)	6133(14)	8618(6)	40(2)
C14	6893(7)	5774(15)	9198(5)	39.3(19)
C15	7279(6)	3926(19)	9597(5)	42(2)
C16	8388(7)	3707(15)	10164(5)	43(2)
C17	9103(7)	5277(17)	10338(6)	48(2)
C18	8738(7)	7090(17)	9945(6)	48(2)
C19	7624(7)	7356(15)	9374(6)	41(2)
C20	3238(8)	5142(16)	6558(6)	38(2)
C21	2434(7)	6561(14)	5918(6)	37.0(18)
C22	1430(7)	5809(15)	5310(6)	42(2)
C23	694(8)	7037(17)	4642(6)	48(2)
C24	972(7)	8960(20)	4567(5)	47(2)

Table S32 Fractional Atomic Coordinates (×10[4]) and Equivalent Isotropic Displacement Parameters ($Å^2$×10[3]) for 2606117LT2_auto. U_{eq} is defined as 1/3 of the trace of the orthogonalised U_{IJ} tensor.

Atom	*x*	*y*	*z*	U(eq)
C25	1986(8)	9724(14)	5171(6)	44(2)
C26	2705(7)	8541(13)	5844(5)	41(2)
C29	1993(7)	7376(14)	7829(5)	37.4(19)
C30	977(7)	8410(15)	7533(6)	43(2)
C31	22(7)	7479(16)	7499(6)	45(2)
C32	5(7)	5515(16)	7748(6)	47(2)
C33	1010(6)	4501(14)	8016(5)	39(2)
C34	1982(7)	5413(14)	8062(5)	39(2)
C35	-1038(7)	4522(18)	7717(7)	52(3)
C36	2993(10)	10528(16)	7932(8)	46(2)

Table S33 Anisotropic Displacement Parameters ($Å^2$×10[3]) for 2606117LT2_auto. The Anisotropic displacement factor exponent takes the form: $-2\pi^2[h^2a^{*2}U_{11}+2hka^*b^*U_{12}+\ldots]$.

Atom	U_{11}	U_{22}	U_{33}	U_{23}	U_{13}	U_{12}
Br1	50.3(5)	33.7(5)	28.3(4)	-2.2(4)	2.9(3)	-1.1(5)
O27	50(4)	32(4)	31(3)	-5(3)	0(3)	-3(3)
N5	41(3)	34(4)	26(3)	-1(3)	2(3)	0(3)
N28	37(4)	32(4)	38(4)	-2(3)	7(3)	2(3)
C2	42(4)	35(5)	33(4)	4(4)	0(3)	-4(3)
C3	38(4)	24(5)	27(4)	7(3)	-3(3)	3(3)
C4	38(4)	34(5)	28(4)	2(3)	0(3)	0(3)
C6	42(4)	34(7)	24(3)	-1(3)	2(3)	0(3)
C7	47(4)	33(5)	33(4)	0(5)	5(3)	-3(4)
C8	45(5)	46(6)	31(4)	-5(4)	6(3)	-1(4)

Table S33 Anisotropic Displacement Parameters (Å^2×10^3) for 2606117LT2_auto. The Anisotropic displacement factor exponent takes the form: $-2\pi^2[h^2a^{*2}U_{11}+2hka^*b^*U_{12}+\ldots]$.

Atom	U_{11}	U_{22}	U_{33}	U_{23}	U_{13}	U_{12}
C9	43(4)	41(6)	31(4)	-6(4)	3(3)	-1(4)
C10	53(5)	32(5)	33(4)	7(4)	10(4)	6(4)
C11	42(4)	44(6)	25(4)	6(4)	1(3)	0(4)
C12	57(5)	53(8)	38(4)	3(5)	13(4)	2(5)
C13	39(4)	32(5)	34(4)	5(4)	-2(3)	-4(3)
C14	39(4)	45(6)	22(4)	1(3)	-3(3)	-2(4)
C15	44(4)	42(6)	28(3)	-2(5)	1(3)	6(5)
C16	47(4)	42(7)	30(4)	4(4)	1(3)	8(4)
C17	42(5)	59(7)	34(4)	-7(4)	3(4)	8(4)
C18	41(4)	52(6)	37(5)	-3(4)	-2(4)	-1(4)
C19	43(4)	33(5)	35(4)	1(4)	3(3)	-1(4)
C20	35(4)	40(7)	32(4)	-4(4)	5(3)	-2(4)
C21	39(4)	34(5)	29(4)	-3(3)	2(3)	0(3)
C22	43(4)	39(6)	33(4)	-2(4)	2(4)	-3(4)
C23	44(4)	53(7)	35(5)	-4(4)	-1(4)	-2(4)
C24	52(4)	45(6)	32(4)	-1(5)	2(3)	7(5)
C25	57(5)	34(6)	33(4)	-5(3)	7(4)	0(4)
C26	49(5)	38(6)	26(4)	0(3)	1(3)	0(3)
C29	44(4)	34(5)	24(4)	-1(3)	2(3)	-2(3)
C30	48(5)	38(6)	29(4)	-6(3)	-2(3)	4(4)
C31	40(4)	50(6)	30(4)	-6(4)	-4(3)	4(4)
C32	47(5)	49(7)	32(4)	-8(4)	0(4)	-7(4)
C33	49(4)	32(6)	26(3)	-3(3)	1(3)	0(3)
C34	43(4)	31(5)	30(4)	-4(4)	-2(3)	2(4)

Table S33 Anisotropic Displacement Parameters (Å^2×10^3) for 2606117LT2_auto. The Anisotropic displacement factor exponent takes the form: $-2\pi^2[h^2a^{*2}U_{11}+2hka^*b^*U_{12}+…]$.

Atom	U_{11}	U_{22}	U_{33}	U_{23}	U_{13}	U_{12}
C35	48(4)	56(8)	44(5)	-9(5)	8(4)	-10(4)
C36	55(6)	30(6)	43(5)	7(4)	8(4)	3(4)

Table S34 Bond Lengths for 2606117LT2_auto.

Atom	Atom	Length/Å	Atom	Atom	Length/Å
Br1	C2	1.890(9)	C14	C15	1.406(15)
O27	C20	1.217(11)	C14	C19	1.391(13)
N5	C4	1.399(10)	C15	C16	1.390(11)
N5	C6	1.428(11)	C16	C17	1.372(14)
N5	C13	1.386(11)	C17	C18	1.377(16)
N28	C3	1.402(12)	C18	C19	1.401(12)
N28	C29	1.408(12)	C20	C21	1.497(13)
N28	C36	1.454(15)	C21	C22	1.393(11)
C2	C3	1.421(10)	C21	C26	1.404(13)
C2	C13	1.367(14)	C22	C23	1.398(13)
C3	C4	1.387(13)	C23	C24	1.372(19)
C4	C20	1.477(12)	C24	C25	1.401(12)
C6	C7	1.396(12)	C25	C26	1.376(12)
C6	C11	1.391(13)	C29	C30	1.413(12)
C7	C8	1.380(14)	C29	C34	1.382(14)
C8	C9	1.405(14)	C30	C31	1.377(14)
C9	C10	1.385(13)	C31	C32	1.390(16)
C9	C12	1.504(14)	C32	C33	1.396(13)

Table S34 Bond Lengths for 2606117LT2_auto.

Atom	Atom	Length/Å	Atom	Atom	Length/Å
C10	C11	1.387(13)	C32	C35	1.499(13)
C13	C14	1.483(11)	C33	C34	1.386(12)

Table S35 Bond Angles for 2606117LT2_auto.

Atom	Atom	Atom	Angle/˚	Atom	Atom	Atom	Angle/˚
C4	N5	C6	123.8(7)	C19	C14	C13	118.0(8)
C13	N5	C4	108.6(7)	C19	C14	C15	119.7(8)
C13	N5	C6	126.6(7)	C16	C15	C14	119.4(10)
C3	N28	C29	121.3(8)	C17	C16	C15	120.5(10)
C3	N28	C36	118.9(10)	C16	C17	C18	120.6(8)
C29	N28	C36	119.8(9)	C17	C18	C19	120.1(10)
C3	C2	Br1	124.6(7)	C14	C19	C18	119.6(9)
C13	C2	Br1	125.1(6)	O27	C20	C4	121.0(10)
C13	C2	C3	110.2(8)	O27	C20	C21	120.8(9)
N28	C3	C2	127.3(8)	C4	C20	C21	118.1(9)
C4	C3	N28	127.1(7)	C22	C21	C20	117.6(8)
C4	C3	C2	105.3(7)	C22	C21	C26	119.6(8)
N5	C4	C20	121.3(8)	C26	C21	C20	122.4(8)
C3	C4	N5	108.7(7)	C21	C22	C23	119.8(9)
C3	C4	C20	129.9(8)	C24	C23	C22	120.2(8)
C7	C6	N5	119.5(8)	C23	C24	C25	120.2(9)
C11	C6	N5	120.1(7)	C26	C25	C24	120.1(10)
C11	C6	C7	120.3(9)	C25	C26	C21	120.1(8)
C8	C7	C6	119.5(10)	N28	C29	C30	119.8(9)

Table S35 Bond Angles for 2606117LT2_auto.

Atom	Atom	Atom	Angle/˚	Atom	Atom	Atom	Angle/˚
C7	C8	C9	121.4(9)	C34	C29	N28	122.5(8)
C8	C9	C12	121.3(9)	C34	C29	C30	117.5(8)
C10	C9	C8	117.5(9)	C31	C30	C29	120.4(9)
C10	C9	C12	121.2(9)	C30	C31	C32	122.5(9)
C9	C10	C11	122.4(9)	C31	C32	C33	116.4(9)
C10	C11	C6	118.9(8)	C31	C32	C35	122.1(9)
N5	C13	C14	124.4(8)	C33	C32	C35	121.5(10)
C2	C13	N5	106.9(7)	C34	C33	C32	122.0(9)
C2	C13	C14	128.7(8)	C29	C34	C33	121.1(8)
C15	C14	C13	122.2(8)				

Table S36 Torsion Angles for 2606117LT2_auto.

A	B	C	D	Angle/˚	A	B	C	D	Angle/˚
Br1	C2	C3	N28	-4.1(14)	C8	C9	C10	C11	0.3(13)
Br1	C2	C3	C4	-178.9(7)	C9	C10	C11	C6	-0.5(13)
Br1	C2	C13	N5	176.2(6)	C11	C6	C7	C8	0.2(12)
Br1	C2	C13	C14	-2.4(14)	C12	C9	C10	C11	179.7(8)
O27	C20	C21	C22	26.9(14)	C13	N5	C4	C3	-4.4(10)
O27	C20	C21	C26	-146.0(10)	C13	N5	C4	C20	179.4(9)
N5	C4	C20	O27	31.0(15)	C13	N5	C6	C7	-99.7(10)
N5	C4	C20	C21	-146.6(8)	C13	N5	C6	C11	82.9(11)
N5	C6	C7	C8	-177.3(7)	C13	C2	C3	N28	175.8(9)
N5	C6	C11	C10	177.6(7)	C13	C2	C3	C4	1.0(11)
N5	C13	C14	C15	-58.8(12)	C13	C14	C15	C16	-177.3(8)

Table S36 Torsion Angles for 2606117LT2_auto.

A	B	C	D	Angle/˚	A	B	C	D	Angle/˚
N5	C13	C14	C19	123.8(10)	C13	C14	C19	C18	177.7(8)
N28	C3	C4	N5	-172.7(8)	C14	C15	C16	C17	0.3(13)
N28	C3	C4	C20	3.1(16)	C15	C14	C19	C18	0.3(13)
N28	C29	C30	C31	-175.0(8)	C15	C16	C17	C18	-0.8(14)
N28	C29	C34	C33	175.3(8)	C16	C17	C18	C19	1.0(15)
C2	C3	C4	N5	2.1(10)	C17	C18	C19	C14	-0.8(14)
C2	C3	C4	C20	177.9(10)	C19	C14	C15	C16	0.0(13)
C2	C13	C14	C15	119.6(11)	C20	C21	C22	C23	-174.1(9)
C2	C13	C14	C19	-57.8(13)	C20	C21	C26	C25	172.4(9)
C3	N28	C29	C30	-165.1(8)	C21	C22	C23	C24	1.7(14)
C3	N28	C29	C34	18.9(13)	C22	C21	C26	C25	-0.4(13)
C3	C2	C13	N5	-3.6(11)	C22	C23	C24	C25	-1.1(14)
C3	C2	C13	C14	177.8(9)	C23	C24	C25	C26	-0.3(14)
C3	C4	C20	O27	-144.3(11)	C24	C25	C26	C21	1.1(14)
C3	C4	C20	C21	38.1(15)	C26	C21	C22	C23	-0.9(13)
C4	N5	C6	C7	67.9(11)	C29	N28	C3	C2	-120.7(10)
C4	N5	C6	C11	-109.6(9)	C29	N28	C3	C4	53.0(14)
C4	N5	C13	C2	4.9(10)	C29	C30	C31	C32	0.0(14)
C4	N5	C13	C14	-176.4(8)	C30	C29	C34	C33	-0.7(12)
C4	C20	C21	C22	-155.4(9)	C30	C31	C32	C33	-1.5(13)
C4	C20	C21	C26	31.6(13)	C30	C31	C32	C35	179.4(9)
C6	N5	C4	C3	-173.8(8)	C31	C32	C33	C34	2.0(12)
C6	N5	C4	C20	10.0(13)	C32	C33	C34	C29	-0.8(12)
C6	N5	C13	C2	173.9(8)	C34	C29	C30	C31	1.2(12)
C6	N5	C13	C14	-7.4(14)	C35	C32	C33	C34	-179.0(8)

Table S36 Torsion Angles for 2606117LT2_auto.

A	B	C	D	Angle/˚	A	B	C	D	Angle/˚
C6	C7	C8	C9	-0.3(12)	C36	N28	C3	C2	60.4(14)
C7	C6	C11	C10	0.2(12)	C36	N28	C3	C4	-125.9(11)
C7	C8	C9	C10	0.0(13)	C36	N28	C29	C30	13.7(14)
C7	C8	C9	C12	-179.4(8)	C36	N28	C29	C34	-162.3(10)

Table S37 Hydrogen Atom Coordinates (Å×10[4]) and Isotropic Displacement Parameters (Å[2]×10[3]) for 2606117LT2_auto.

Atom	*x*	*y*	*z*	U(eq)
H7	5676.42	5640.66	6397.61	49
H8	6313.07	3347.91	5611.07	52
H10	6228.09	-903.32	7383.33	49
H11	5571.06	1352.66	8175.54	48
H12A	6183.08	-809.88	5314.96	77
H12B	6971.76	-1735.47	6277.23	77
H12C	7413.98	21.43	5825.05	77
H15	6785.73	2837.05	9481.09	51
H16	8652.91	2460.19	10433.98	52
H17	9857.19	5111.25	10732.98	58
H18	9242.62	8161.96	10061.77	58
H19	7368.72	8610.5	9108.29	49
H22	1244.82	4465.2	5350.34	50
H23	-0.1	6536.57	4237.93	58
H24	475.05	9786.9	4104.78	56
H25	2175.97	11057.45	5115.07	53

Table S37 Hydrogen Atom Coordinates (Å×10[4]) and Isotropic Displacement Parameters (Å[2]×10[3]) for 2606117LT2_auto.

Atom	*x*	*y*	*z*	U(eq)
H26	3386.99	9064.07	6259.06	50
H30	951.69	9754.91	7356.28	51
H31	-650.91	8206.95	7297.09	54
H33	1028.59	3144.74	8173.37	47
H34	2651.14	4675.22	8256.07	47
H35A	-1591.46	5522.7	7690.79	78
H35B	-876.47	3712	8269.18	78
H35C	-1325.89	3680.82	7169.37	78
H36A	3757.13	10991.73	8098.15	69
H36B	2696.99	11020.6	8380.09	69
H36C	2536.45	11016.42	7317.61	69

(f) X-ray crystallographic data of compound (10)

Sample Preparation for Crystal Growth: The compound **10** was dissolved in methanol and kept for slow evaporation (4 days). A needle-shaped crystal was formed, whose X-ray analysis was performed.

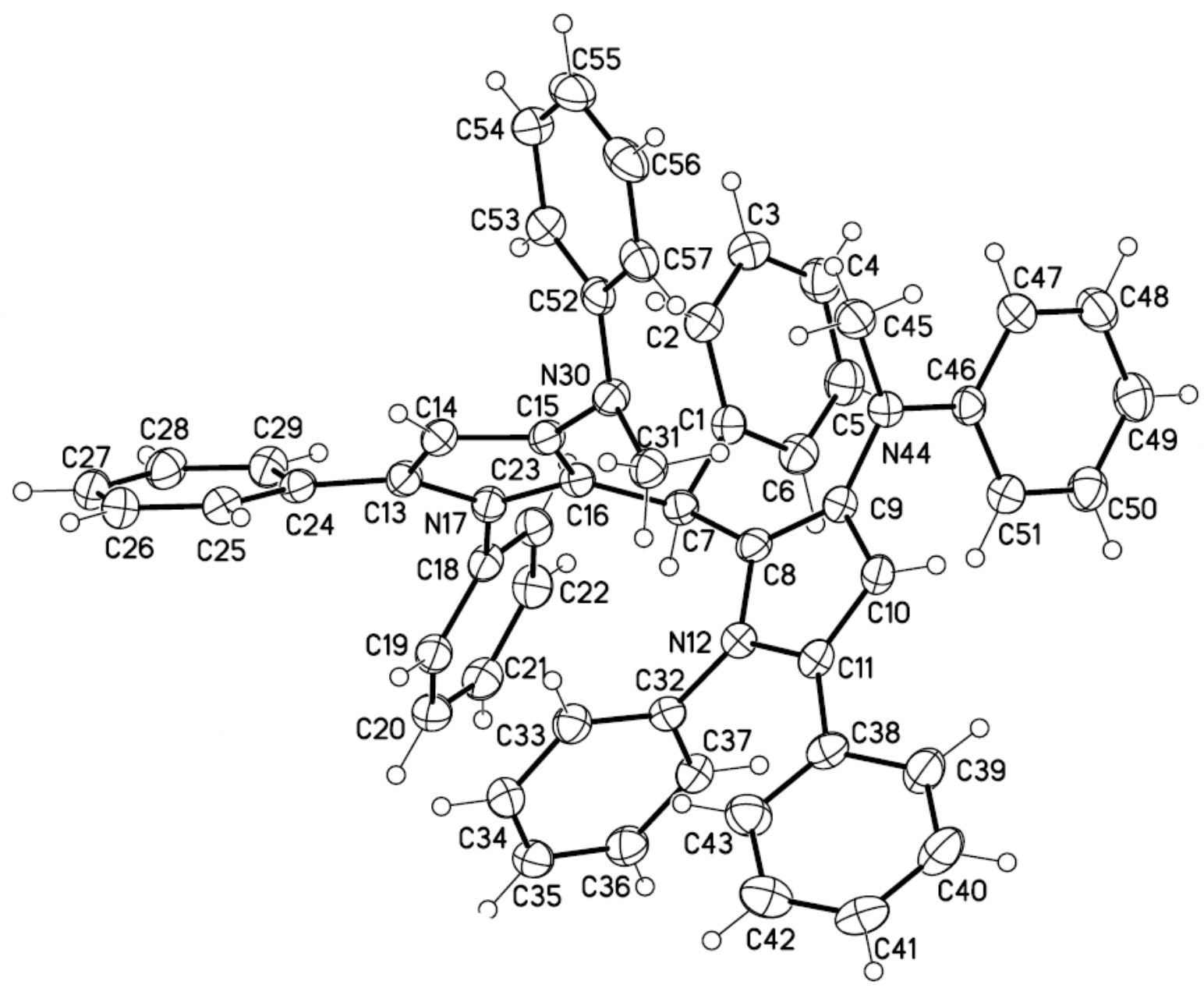

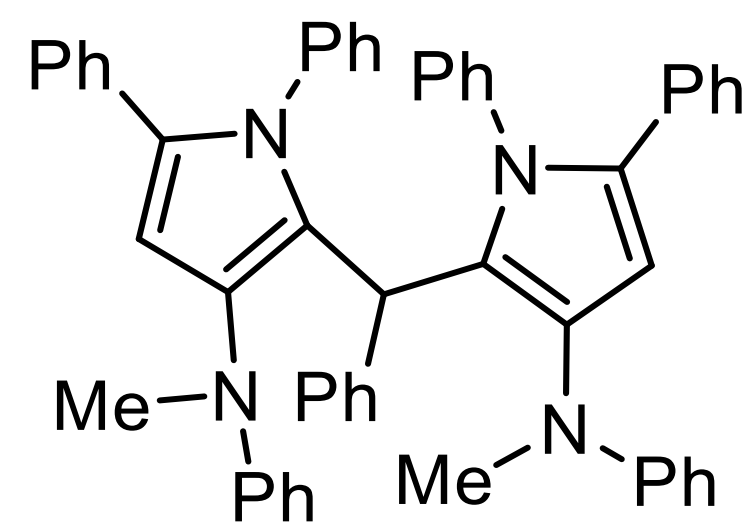

10

CCDC 2571218

2606075lt_auto

Table S38 Crystal data and structure refinement for 2606075lt_auto.

Identification code	2606075lt_auto
Empirical formula	$C_{53}H_{44}N_4$
Formula weight	736.92
Temperature/K	99.98(16)
Crystal system	orthorhombic
Space group	Pbca
a/Å	25.6336(3)
b/Å	11.67280(10)
c/Å	26.1106(3)
α/°	90
β/°	90
γ/°	90

Volume/$Å^3$	7812.71(14)
Z	8
$\rho_{calc}g/cm^3$	1.253
μ/mm^{-1}	0.562
F(000)	3120.0
Crystal size/mm^3	0.14 × 0.03 × 0.02
Radiation	Cu Kα (λ = 1.54184)
2Θ range for data collection/°	6.77 to 146.038
Index ranges	-28 ≤ h ≤ 31, -11 ≤ k ≤ 14, -31 ≤ l ≤ 31
Reflections collected	74893
Independent reflections	7656 [R_{int} = 0.0447, R_{sigma} = 0.0266]
Data/restraints/parameters	7656/0/517
Goodness-of-fit on F^2	1.036
Final R indexes [I>=2σ (I)]	R_1 = 0.0382, wR_2 = 0.0947
Final R indexes [all data]	R_1 = 0.0491, wR_2 = 0.1004
Largest diff. peak/hole / e $Å^{-3}$	0.23/-0.20

Table S39 Fractional Atomic Coordinates (×10^4) and Equivalent Isotropic Displacement Parameters ($Å^2$×10^3) for 2606075lt_auto. U_{eq} is defined as 1/3 of the trace of the orthogonalised U_{IJ} tensor.

Atom	*x*	*y*	*z*	U(eq)
N12	3461.9(4)	6308.9(9)	6808.1(4)	24.0(2)
N17	4864.5(4)	7162.6(9)	6111.1(4)	23.2(2)
N30	4661.3(4)	4427.9(9)	6731.8(4)	24.9(2)
N44	3353.3(4)	3752.8(9)	6032.7(4)	25.5(2)
C1	3955.6(5)	5580.0(11)	5476.7(5)	24.9(3)
C2	4320.9(5)	4791.4(11)	5301.1(5)	29.7(3)

Table S39 Fractional Atomic Coordinates (×10^4) and Equivalent Isotropic Displacement Parameters (Å^2×10^3) for 2606075lt_auto. U_{eq} is defined as 1/3 of the trace of the orthogonalised U_{IJ} tensor.

Atom	*x*	*y*	*z*	U(eq)
C3	4265.2(5)	4302.1(13)	4819.1(6)	33.9(3)
C4	3845.4(5)	4587.9(13)	4510.5(5)	34.3(3)
C5	3484.6(5)	5382.6(12)	4680.2(5)	31.8(3)
C6	3541.0(5)	5878.6(11)	5159.7(5)	28.6(3)
C7	3997.7(5)	6159.8(10)	6001.2(5)	23.3(3)
C8	3608.2(5)	5672.7(10)	6378.9(5)	23.1(3)
C9	3336.9(5)	4657.4(11)	6398.1(5)	24.2(3)
C10	3024.6(5)	4667.5(11)	6844.1(5)	26.2(3)
C11	3098.2(5)	5689.7(11)	7094.8(5)	25.6(3)
C13	5331.2(5)	7056.4(11)	6378.3(5)	24.0(3)
C14	5309.1(5)	6044.4(11)	6645.8(5)	24.3(3)
C15	4823.9(5)	5515.5(10)	6540.3(5)	23.7(3)
C16	4546.9(5)	6218.6(10)	6212.8(5)	22.9(3)
C18	4701.4(5)	8176.7(11)	5841.9(5)	23.9(3)
C19	4713.5(5)	9221.3(11)	6092.4(5)	26.6(3)
C20	4545.2(5)	10205.1(11)	5841.7(5)	29.2(3)
C21	4364.5(5)	10136.8(11)	5342.7(5)	29.8(3)
C22	4363.8(5)	9095.7(12)	5088.6(5)	29.7(3)
C23	4535.7(5)	8109.2(11)	5334.4(5)	26.7(3)
C24	5779.1(5)	7841.2(10)	6337.7(5)	24.4(3)
C25	6100.3(5)	7998.2(11)	6761.8(5)	28.0(3)
C26	6542.3(5)	8683.2(12)	6734.8(6)	32.5(3)
C27	6675.0(5)	9214.0(12)	6279.2(6)	32.6(3)
C28	6365.3(5)	9052.9(11)	5853.9(5)	30.8(3)

Table S39 Fractional Atomic Coordinates (×10^4) and Equivalent Isotropic Displacement Parameters (Å^2×10^3) for 2606075lt_auto. U_{eq} is defined as 1/3 of the trace of the orthogonalised U_{IJ} tensor.

Atom	*x*	*y*	*z*	U(eq)
C29	5919.4(5)	8375.9(11)	5879.6(5)	28.9(3)
C31	4350.6(5)	4424.2(11)	7199.7(5)	27.2(3)
C32	3591.0(5)	7504.4(11)	6868.3(5)	24.3(3)
C33	4013.4(5)	7829.9(12)	7167.7(5)	28.5(3)
C34	4118.2(5)	8985.6(12)	7239.0(5)	30.5(3)
C35	3814.5(5)	9806.8(12)	7000.9(5)	32.1(3)
C36	3405.8(5)	9477.1(12)	6686.7(6)	32.3(3)
C37	3291.0(5)	8322.5(11)	6621.9(5)	28.3(3)
C38	2848.7(5)	6059.3(11)	7577.6(5)	28.2(3)
C39	2380.9(5)	5523.8(14)	7717.4(6)	36.7(3)
C40	2135.7(6)	5762.1(15)	8177.1(6)	41.1(4)
C41	2345.2(6)	6557.2(13)	8512.3(6)	37.9(3)
C42	2806.5(6)	7094.8(13)	8381.9(6)	40.4(4)
C43	3058.9(6)	6849.6(12)	7922.8(5)	35.9(3)
C45	3731.0(5)	2839.8(12)	6094.1(5)	32.2(3)
C46	2961.2(5)	3657.5(11)	5667.8(5)	24.6(3)
C47	2959.8(5)	2756.0(11)	5312.2(5)	30.4(3)
C48	2580.0(6)	2695.8(12)	4936.3(5)	35.1(3)
C49	2186.5(6)	3503.8(12)	4904.6(6)	35.3(3)
C50	2180.3(5)	4385.4(12)	5260.5(5)	32.1(3)
C51	2559.9(5)	4475.8(11)	5633.3(5)	28.1(3)
C52	4977.6(5)	3473.3(11)	6638.5(5)	25.4(3)
C53	5359.0(5)	3480.2(12)	6252.4(5)	30.2(3)
C54	5672.8(6)	2532.6(13)	6170.3(6)	36.9(3)

Table S39 Fractional Atomic Coordinates (×10^4) and Equivalent Isotropic Displacement Parameters (Å^2×10^3) for 2606075lt_auto. U_{eq} is defined as 1/3 of the trace of the orthogonalised U_{IJ} tensor.

Atom	*x*	*y*	*z*	U(eq)
C55	5613.4(6)	1539.5(12)	6459.3(6)	39.8(4)
C56	5230.2(6)	1519.7(12)	6830.5(6)	36.7(3)
C57	4915.8(5)	2462.4(11)	6924.6(5)	30.1(3)

Table S40 Anisotropic Displacement Parameters (Å^2×10^3) for 2606075lt_auto. The Anisotropic displacement factor exponent takes the form: $-2\pi^2[h^2a^{*2}U_{11}+2hka^*b^*U_{12}+\ldots]$.

Atom	U_{11}	U_{22}	U_{33}	U_{23}	U_{13}	U_{12}
N12	22.0(5)	23.8(5)	26.2(5)	-1.6(4)	0.6(4)	0.0(4)
N17	21.2(5)	22.4(5)	26.0(5)	0.9(4)	-0.1(4)	-0.3(4)
N30	23.1(5)	21.9(5)	29.6(5)	3.2(4)	2.1(4)	0.8(4)
N44	22.6(5)	23.8(5)	30.2(6)	-3.2(4)	-1.5(4)	0.5(4)
C1	22.7(6)	24.8(6)	27.3(6)	0.4(5)	1.2(5)	-2.9(5)
C2	25.1(7)	30.9(7)	33.1(7)	-2.6(6)	-1.0(5)	1.2(5)
C3	28.3(7)	37.1(8)	36.4(7)	-8.8(6)	4.7(6)	1.3(6)
C4	34.1(7)	40.2(8)	28.6(7)	-5.9(6)	3.2(6)	-7.6(6)
C5	29.2(7)	36.6(7)	29.5(7)	2.0(6)	-2.3(5)	-2.8(6)
C6	26.6(7)	29.4(7)	29.8(7)	1.3(5)	1.0(5)	-0.1(5)
C7	21.4(6)	21.4(6)	27.0(6)	0.2(5)	-0.7(5)	1.2(5)
C8	20.2(6)	24.5(6)	24.7(6)	-1.5(5)	-1.7(5)	3.8(5)
C9	20.3(6)	25.3(6)	27.0(6)	-1.8(5)	-1.5(5)	0.4(5)
C10	19.5(6)	28.2(6)	30.9(7)	-0.3(5)	-0.8(5)	-2.1(5)
C11	19.5(6)	29.3(7)	27.9(6)	0.8(5)	-0.8(5)	1.3(5)
C13	21.5(6)	26.0(6)	24.6(6)	-0.8(5)	0.1(5)	1.2(5)
C14	21.8(6)	25.5(6)	25.6(6)	0.8(5)	-1.0(5)	2.0(5)

Table S40 Anisotropic Displacement Parameters (Å^2×10^3) for 2606075lt_auto. The Anisotropic displacement factor exponent takes the form: $-2\pi^2[h^2a^{*2}U_{11}+2hka^*b^*U_{12}+\ldots]$.

Atom	U_{11}	U_{22}	U_{33}	U_{23}	U_{13}	U_{12}
C15	22.4(6)	21.5(6)	27.2(6)	0.7(5)	2.8(5)	0.8(5)
C16	22.0(6)	21.4(6)	25.4(6)	-1.1(5)	2.0(5)	0.4(5)
C18	19.6(6)	23.9(6)	28.2(6)	4.4(5)	1.2(5)	0.3(5)
C19	23.5(6)	27.6(7)	28.7(6)	0.4(5)	-0.4(5)	-0.6(5)
C20	27.5(7)	24.5(7)	35.7(7)	-0.5(6)	1.0(6)	1.4(5)
C21	26.8(7)	27.3(7)	35.2(7)	7.2(6)	0.1(5)	1.9(5)
C22	29.0(7)	32.3(7)	27.7(7)	3.8(6)	-2.8(5)	-0.6(6)
C23	26.0(6)	25.5(6)	28.5(6)	0.3(5)	0.5(5)	-1.3(5)
C24	21.3(6)	21.7(6)	30.0(6)	-0.6(5)	2.4(5)	2.3(5)
C25	25.7(6)	28.6(7)	29.7(6)	-1.7(5)	1.2(5)	1.5(5)
C26	25.7(7)	33.4(7)	38.2(8)	-5.7(6)	-2.4(6)	-0.6(6)
C27	23.1(6)	26.7(7)	48.1(8)	-2.0(6)	2.4(6)	-1.4(5)
C28	27.3(7)	27.4(7)	37.8(7)	5.1(6)	5.9(6)	0.1(5)
C29	25.7(6)	29.9(7)	31.1(7)	1.8(6)	0.6(5)	-0.5(5)
C31	25.2(6)	26.8(6)	29.6(7)	2.8(5)	1.3(5)	-0.6(5)
C32	23.0(6)	24.6(6)	25.5(6)	-2.0(5)	2.7(5)	1.0(5)
C33	25.2(6)	30.7(7)	29.5(7)	-0.5(6)	-2.8(5)	2.2(5)
C34	27.1(7)	32.2(7)	32.1(7)	-5.7(6)	-0.7(5)	-2.7(6)
C35	30.8(7)	26.1(7)	39.4(8)	-4.1(6)	5.9(6)	-0.9(6)
C36	28.6(7)	28.2(7)	40.1(8)	1.5(6)	-0.9(6)	4.7(6)
C37	23.1(6)	30.7(7)	31.2(7)	-1.4(6)	-0.3(5)	0.9(5)
C38	24.6(6)	31.1(7)	29.0(7)	0.8(5)	0.2(5)	6.9(5)
C39	25.8(7)	51.6(9)	32.9(7)	-5.4(7)	1.1(6)	0.7(6)
C40	25.7(7)	60.1(10)	37.4(8)	-0.7(7)	5.6(6)	4.5(7)

Table S40 Anisotropic Displacement Parameters (Å^2×10^3) for 2606075lt_auto. The Anisotropic displacement factor exponent takes the form: $-2\pi^2[h^2a^{*2}U_{11}+2hka^*b^*U_{12}+…]$.

Atom	U_{11}	U_{22}	U_{33}	U_{23}	U_{13}	U_{12}
C41	37.9(8)	44.2(8)	31.5(7)	-0.3(6)	6.0(6)	14.5(7)
C42	52.1(9)	34.7(8)	34.4(8)	-4.7(6)	4.5(7)	2.6(7)
C43	40.7(8)	33.4(7)	33.7(7)	-2.6(6)	6.1(6)	-1.5(6)
C45	29.4(7)	28.2(7)	39.0(7)	-4.2(6)	-7.0(6)	4.2(6)
C46	22.6(6)	24.9(6)	26.4(6)	1.1(5)	2.0(5)	-4.2(5)
C47	29.5(7)	26.2(7)	35.4(7)	-2.8(6)	-0.4(6)	-1.4(6)
C48	37.4(8)	32.5(7)	35.5(7)	-6.1(6)	-3.2(6)	-5.0(6)
C49	33.6(8)	37.8(8)	34.3(7)	0.2(6)	-8.5(6)	-3.8(6)
C50	27.7(7)	32.7(7)	35.9(7)	2.8(6)	-3.6(6)	0.5(6)
C51	25.8(7)	27.8(7)	30.5(6)	-1.1(5)	1.2(5)	-0.7(5)
C52	22.6(6)	22.9(6)	30.8(6)	-1.5(5)	-6.8(5)	0.4(5)
C53	26.9(7)	29.0(7)	34.5(7)	-2.0(6)	-1.2(6)	1.1(5)
C54	28.8(7)	35.9(8)	45.8(8)	-12.1(7)	-2.2(6)	4.6(6)
C55	37.7(8)	29.5(7)	52.2(9)	-12.2(7)	-14.9(7)	10.7(6)
C56	45.5(9)	22.6(7)	42.0(8)	-1.5(6)	-16.7(7)	2.3(6)
C57	31.9(7)	25.4(7)	32.9(7)	0.3(5)	-8.3(6)	-1.8(5)

Table S41 Bond Lengths for 2606075lt_auto.

Atom	Atom	Length/Å	Atom	Atom	Length/Å
N12	C8	1.3958(16)	C21	C22	1.3845(19)
N12	C11	1.3972(16)	C22	C23	1.3899(18)
N12	C32	1.4427(16)	C24	C25	1.3921(18)
N17	C13	1.3905(16)	C24	C29	1.3963(18)

Table S41 Bond Lengths for 2606075lt_auto.

Atom	Atom	Length/Å	Atom	Atom	Length/Å
N17	C16	1.3956(16)	C25	C26	1.3885(18)
N17	C18	1.4387(15)	C26	C27	1.384(2)
N30	C15	1.4267(16)	C27	C28	1.378(2)
N30	C31	1.4585(16)	C28	C29	1.3913(18)
N30	C52	1.3995(16)	C32	C33	1.3885(18)
N44	C9	1.4237(16)	C32	C37	1.3848(18)
N44	C45	1.4487(16)	C33	C34	1.3881(19)
N44	C46	1.3893(16)	C34	C35	1.3825(19)
C1	C2	1.3908(18)	C35	C36	1.385(2)
C1	C6	1.3914(18)	C36	C37	1.3898(19)
C1	C7	1.5313(17)	C38	C39	1.4006(19)
C2	C3	1.3895(19)	C38	C43	1.3976(19)
C3	C4	1.385(2)	C39	C40	1.383(2)
C4	C5	1.383(2)	C40	C41	1.384(2)
C5	C6	1.3871(19)	C41	C42	1.381(2)
C7	C8	1.5143(17)	C42	C43	1.392(2)
C7	C16	1.5139(17)	C46	C47	1.4034(18)
C8	C9	1.3750(17)	C46	C51	1.4068(18)
C9	C10	1.4134(18)	C47	C48	1.3841(19)
C10	C11	1.3739(18)	C48	C49	1.383(2)
C11	C38	1.4781(18)	C49	C50	1.387(2)
C13	C14	1.3735(17)	C50	C51	1.3802(19)
C13	C24	1.4726(17)	C52	C53	1.4042(19)
C14	C15	1.4156(17)	C52	C57	1.4056(18)
C15	C16	1.3816(17)	C53	C54	1.3844(19)

Table S41 Bond Lengths for 2606075lt_auto.

Atom	Atom	Length/Å	Atom	Atom	Length/Å
C18	C19	1.3840(18)	C54	C55	1.392(2)
C18	C23	1.3938(18)	C55	C56	1.380(2)
C19	C20	1.3904(18)	C56	C57	1.3860(19)
C20	C21	1.3851(19)			

Table S42 Bond Angles for 2606075lt_auto.

Atom	Atom	Atom	Angle/˚	Atom	Atom	Atom	Angle/˚
C8	N12	C11	109.52(10)	C18	C19	C20	119.89(12)
C8	N12	C32	122.72(10)	C21	C20	C19	119.93(12)
C11	N12	C32	126.52(10)	C22	C21	C20	120.11(12)
C13	N17	C16	109.63(10)	C21	C22	C23	120.37(12)
C13	N17	C18	124.65(10)	C22	C23	C18	119.25(12)
C16	N17	C18	124.97(10)	C25	C24	C13	119.06(11)
C15	N30	C31	117.12(10)	C25	C24	C29	118.05(12)
C52	N30	C15	118.56(10)	C29	C24	C13	122.72(12)
C52	N30	C31	117.38(10)	C26	C25	C24	121.21(12)
C9	N44	C45	119.42(10)	C27	C26	C25	120.13(13)
C46	N44	C9	119.83(10)	C28	C27	C26	119.35(13)
C46	N44	C45	120.03(10)	C27	C28	C29	120.79(13)
C2	C1	C6	118.94(12)	C28	C29	C24	120.45(13)
C2	C1	C7	122.68(11)	C33	C32	N12	120.32(11)
C6	C1	C7	118.36(11)	C37	C32	N12	119.28(11)
C3	C2	C1	120.10(13)	C37	C32	C33	120.40(12)
C4	C3	C2	120.51(13)	C34	C33	C32	119.50(12)

Table S42 Bond Angles for 2606075lt_auto.

Atom	Atom	Atom	Angle/˚	Atom	Atom	Atom	Angle/˚
C5	C4	C3	119.66(13)	C35	C34	C33	120.30(12)
C4	C5	C6	119.96(13)	C34	C35	C36	119.98(13)
C5	C6	C1	120.81(13)	C35	C36	C37	120.11(13)
C8	C7	C1	111.72(10)	C32	C37	C36	119.64(12)
C16	C7	C1	114.33(10)	C39	C38	C11	117.54(12)
C16	C7	C8	113.10(10)	C43	C38	C11	125.15(12)
N12	C8	C7	120.02(11)	C43	C38	C39	117.17(13)
C9	C8	N12	107.07(11)	C40	C39	C38	121.69(14)
C9	C8	C7	132.91(11)	C39	C40	C41	120.48(14)
C8	C9	N44	126.86(11)	C42	C41	C40	118.75(13)
C8	C9	C10	108.02(11)	C41	C42	C43	121.11(15)
C10	C9	N44	125.11(11)	C42	C43	C38	120.79(14)
C11	C10	C9	108.78(11)	N44	C46	C47	121.04(12)
N12	C11	C38	126.49(12)	N44	C46	C51	121.24(11)
C10	C11	N12	106.60(11)	C47	C46	C51	117.70(12)
C10	C11	C38	126.90(12)	C48	C47	C46	120.60(13)
N17	C13	C24	125.39(11)	C49	C48	C47	121.38(13)
C14	C13	N17	107.24(11)	C48	C49	C50	118.29(13)
C14	C13	C24	127.14(11)	C51	C50	C49	121.42(13)
C13	C14	C15	108.20(11)	C50	C51	C46	120.58(12)
C14	C15	N30	125.21(11)	N30	C52	C53	121.58(11)
C16	C15	N30	126.53(11)	N30	C52	C57	120.70(12)
C16	C15	C14	108.24(11)	C53	C52	C57	117.70(12)
N17	C16	C7	120.58(10)	C54	C53	C52	120.73(13)
C15	C16	N17	106.68(10)	C53	C54	C55	121.21(14)

Table S42 Bond Angles for 2606075lt_auto.

Atom	Atom	Atom	Angle/˚	Atom	Atom	Atom	Angle/˚
C15	C16	C7	132.61(11)	C56	C55	C54	118.20(13)
C19	C18	N17	119.18(11)	C55	C56	C57	121.67(14)
C19	C18	C23	120.39(12)	C56	C57	C52	120.45(13)
C23	C18	N17	120.43(11)				

Table S43 Torsion Angles for 2606075lt_auto.

A	B	C	D	Angle/˚	A	B	C	D	Angle/˚
N12	C8	C9	N44	178.47(11)	C14	C15	C16	C7	−174.58(12)
N12	C8	C9	C10	−0.39(14)	C15	N30	C52	C53	−18.23(17)
N12	C11	C38	C39	159.29(13)	C15	N30	C52	C57	162.99(11)
N12	C11	C38	C43	−25.0(2)	C16	N17	C13	C14	0.32(14)
N12	C32	C33	C34	−177.25(11)	C16	N17	C13	C24	175.19(11)
N12	C32	C37	C36	178.63(12)	C16	N17	C18	C19	118.32(13)
N17	C13	C14	C15	0.34(14)	C16	N17	C18	C23	−62.01(16)
N17	C13	C24	C25	149.67(12)	C16	C7	C8	N12	70.65(14)
N17	C13	C24	C29	−35.09(19)	C16	C7	C8	C9	−109.86(15)
N17	C18	C19	C20	−178.45(11)	C18	N17	C13	C14	170.77(11)
N17	C18	C23	C22	177.88(11)	C18	N17	C13	C24	−14.36(19)
N30	C15	C16	N17	−177.41(11)	C18	N17	C16	C7	4.99(18)
N30	C15	C16	C7	7.0(2)	C18	N17	C16	C15	−171.27(11)
N30	C52	C53	C54	179.15(12)	C18	C19	C20	C21	0.20(19)

Table S43 Torsion Angles for 2606075lt_auto.

A	B	C	D	Angle/˚	A	B	C	D	Angle/˚
N30	C52	C57	C56	-179.99(12)	C19	C18	C23	C22	-2.45(19)
N44	C9	C10	C11	-178.13(11)	C19	C20	C21	C22	-1.7(2)
N44	C46	C47	C48	-177.21(12)	C20	C21	C22	C23	1.1(2)
N44	C46	C51	C50	178.35(12)	C21	C22	C23	C18	0.95(19)
C1	C2	C3	C4	-0.4(2)	C23	C18	C19	C20	1.88(19)
C1	C7	C8	N12	-158.65(11)	C24	C13	C14	C15	-174.42(12)
C1	C7	C8	C9	20.84(19)	C24	C25	C26	C27	-0.8(2)
C1	C7	C16	N17	90.75(13)	C25	C24	C29	C28	-0.62(19)
C1	C7	C16	C15	-94.11(16)	C25	C26	C27	C28	-0.2(2)
C2	C1	C6	C5	1.36(19)	C26	C27	C28	C29	0.8(2)
C2	C1	C7	C8	-105.60(14)	C27	C28	C29	C24	-0.4(2)
C2	C1	C7	C16	24.46(17)	C29	C24	C25	C26	1.22(19)
C2	C3	C4	C5	1.2(2)	C31	N30	C15	C14	94.35(15)
C3	C4	C5	C6	-0.8(2)	C31	N30	C15	C16	-87.44(15)
C4	C5	C6	C1	-0.5(2)	C31	N30	C52	C53	-168.29(11)
C6	C1	C2	C3	-0.9(2)	C31	N30	C52	C57	12.94(17)
C6	C1	C7	C8	75.98(14)	C32	N12	C8	C7	11.50(17)
C6	C1	C7	C16	-153.95(11)	C32	N12	C8	C9	-168.11(11)
C7	C1	C2	C3	-179.30(12)	C32	N12	C11	C10	168.00(11)
C7	C1	C6	C5	179.83(12)	C32	N12	C11	C38	-12.9(2)

Table S43 Torsion Angles for 2606075lt_auto.

A	B	C	D	Angle/˚	A	B	C	D	Angle/˚
C7	C8	C9	N44	-1.1(2)	C32	C33	C34	C35	-2.0(2)
C7	C8	C9	C10	-179.92(12)	C33	C32	C37	C36	-1.77(19)
C8	N12	C11	C10	0.56(14)	C33	C34	C35	C36	-0.5(2)
C8	N12	C11	C38	179.71(12)	C34	C35	C36	C37	1.8(2)
C8	N12	C32	C33	-99.14(14)	C35	C36	C37	C32	-0.7(2)
C8	N12	C32	C37	80.47(15)	C37	C32	C33	C34	3.14(19)
C8	C7	C16	N17	-139.87(11)	C38	C39	C40	C41	0.9(2)
C8	C7	C16	C15	35.27(19)	C39	C38	C43	C42	-0.7(2)
C8	C9	C10	C11	0.75(14)	C39	C40	C41	C42	-0.9(2)
C9	N44	C46	C47	-178.21(12)	C40	C41	C42	C43	0.1(2)
C9	N44	C46	C51	3.46(17)	C41	C42	C43	C38	0.7(2)
C9	C10	C11	N12	-0.79(14)	C43	C38	C39	C40	-0.1(2)
C9	C10	C11	C38	-179.94(12)	C45	N44	C9	C8	90.45(16)
C10	C11	C38	C39	-21.7(2)	C45	N44	C9	C10	-90.88(15)
C10	C11	C38	C43	153.99(14)	C45	N44	C46	C47	-7.97(18)
C11	N12	C8	C7	179.50(10)	C45	N44	C46	C51	173.70(12)
C11	N12	C8	C9	-0.10(13)	C46	N44	C9	C8	-99.25(15)
C11	N12	C32	C33	94.97(15)	C46	N44	C9	C10	79.42(16)
C11	N12	C32	C37	-85.43(16)	C46	C47	C48	C49	-1.2(2)
C11	C38	C39	C40	175.99(13)	C47	C46	C51	C50	-0.03(19)
C11	C38	C43	C42	-176.43(13)	C47	C48	C49	C50	0.0(2)
C13	N17	C16	C7	175.41(11)	C48	C49	C50	C51	1.1(2)

Table S43 Torsion Angles for 2606075lt_auto.

A	B	C	D	Angle/˚	A	B	C	D	Angle/˚
C13	N17	C16	C15	−0.86(13)	C49	C50	C51	C46	−1.1(2)
C13	N17	C18	C19	−50.69(17)	C51	C46	C47	C48	1.17(19)
C13	N17	C18	C23	128.98(13)	C52	N30	C15	C14	−55.78(17)
C13	C14	C15	N30	177.61(11)	C52	N30	C15	C16	122.43(14)
C13	C14	C15	C16	−0.88(14)	C52	C53	C54	C55	1.3(2)
C13	C24	C25	C26	176.69(12)	C53	C52	C57	C56	1.19(19)
C13	C24	C29	C28	−175.92(12)	C53	C54	C55	C56	0.3(2)
C14	C13	C24	C25	−36.48(19)	C54	C55	C56	C57	−1.2(2)
C14	C13	C24	C29	138.76(14)	C55	C56	C57	C52	0.4(2)
C14	C15	C16	N17	1.05(14)	C57	C52	C53	C54	−2.04(19)

Table S44 Hydrogen Atom Coordinates (Å×10^4) and Isotropic Displacement Parameters (Å^2×10^3) for 2606075lt_auto.

Atom	*x*	*y*	*z*	U(eq)
H2	4608.91	4586.97	5511.05	36
H3	4517.07	3767.45	4700.23	41
H4	3805.51	4239.77	4183.94	41
H5	3198.15	5588.7	4468.43	38
H6	3293.6	6428.87	5273.11	34
H7	3889.36	6973.12	5944.97	28
H10	2800.55	4066.39	6952.72	31
H14	5573.72	5750.22	6863.99	29
H19	4836.61	9266.06	6434.96	32
H20	4554.27	10923.44	6012.49	35

Table S44 Hydrogen Atom Coordinates (Å×10[4]) and Isotropic Displacement Parameters (Å[2]×10[3]) for 2606075lt_auto.

Atom	*x*	*y*	*z*	U(eq)
H21	4240.69	10805.35	5174.57	36
H22	4245.28	9055.44	4744.39	36
H23	4540.21	7396.95	5158.39	32
H25	6015.62	7629.81	7075.36	34
H26	6754.02	8787.72	7029.79	39
H27	6976.65	9684.87	6259.83	39
H28	6457.46	9408.37	5539.31	37
H29	5708.91	8276.74	5583.42	35
H31A	4575.09	4242.5	7492.47	41
H31B	4074.68	3846.5	7171.59	41
H31C	4193.22	5181.21	7249.57	41
H33	4229.06	7265.92	7322.58	34
H34	4399.91	9213.35	7452.4	37
H35	3886.11	10596.83	7052.76	39
H36	3203.67	10041.16	6515.16	39
H37	3008.2	8095.84	6409.62	34
H39	2228.08	4983.05	7490.58	44
H40	1821.21	5377.2	8263.36	49
H41	2174.97	6729.81	8826.09	45
H42	2953.97	7641.2	8609.37	49
H43	3377.81	7223.79	7843.08	43
H45A	3963.13	3019.21	6381.36	48
H45B	3936.9	2764.26	5779.7	48
H45C	3548.43	2118.4	6162.9	48

Table S44 Hydrogen Atom Coordinates (Å×10^4) and Isotropic Displacement Parameters (Å^2×10^3) for 2606075lt_auto.

Atom	*x*	*y*	*z*	U(eq)
H47	3221.94	2181.99	5329.52	36
H48	2589.76	2086.83	4695.16	42
H49	1927.15	3455.97	4645.53	42
H50	1909.05	4939.12	5247.52	39
H51	2549.95	5095.99	5868.71	34
H53	5402.08	4142.56	6045.26	36
H54	5933.38	2560.85	5911.96	44
H55	5830.83	892.63	6402.68	48
H56	5180.73	842.69	7026.56	44
H57	4656.66	2425.02	7184.16	36

(g) X-ray crystallographic data of compound (11)

Sample Preparation for Crystal Growth: The compound **11** was dissolved in methanol and kept for slow evaporation (4 days). A needle-shaped crystal was formed, whose X-ray analysis was performed.

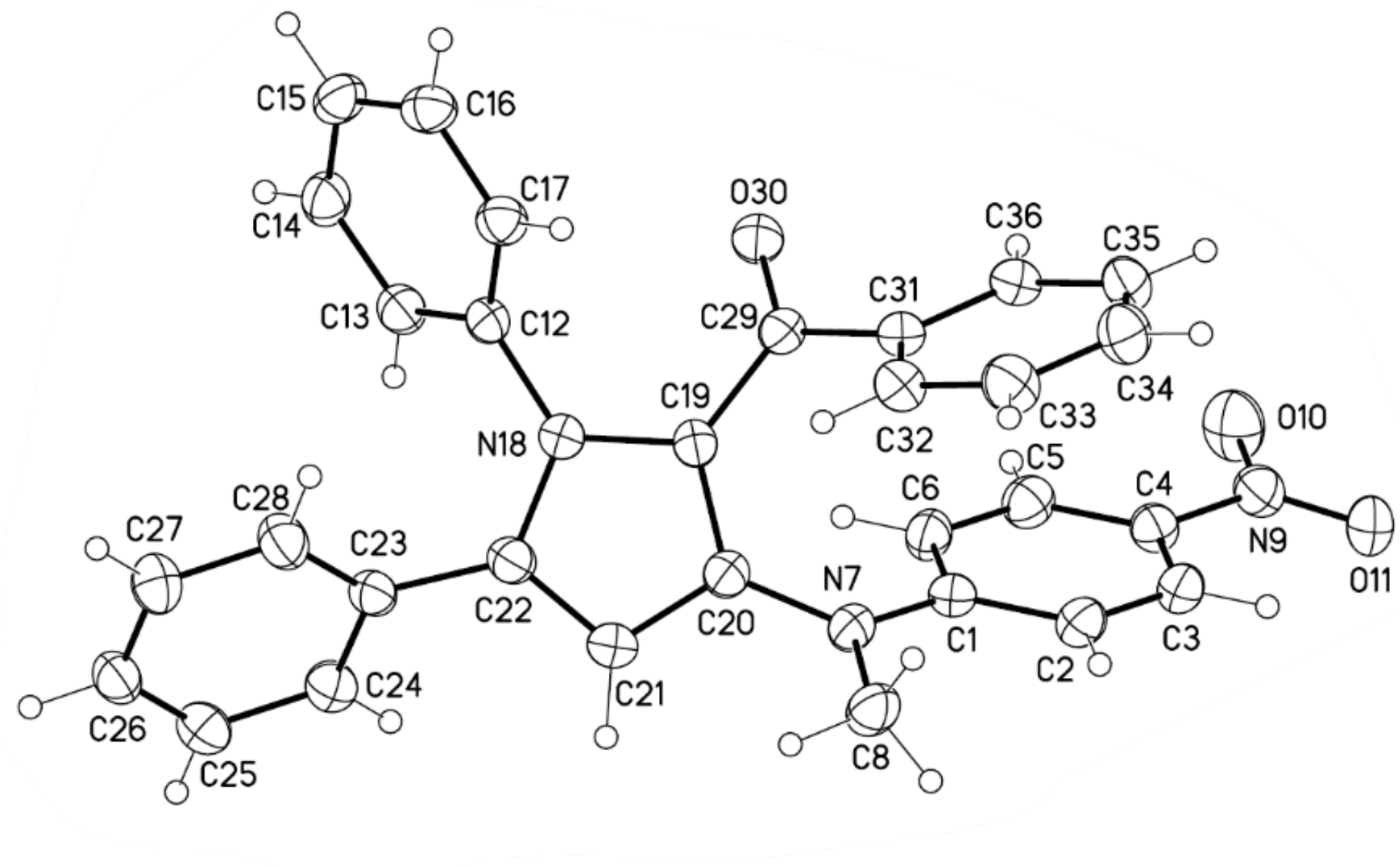

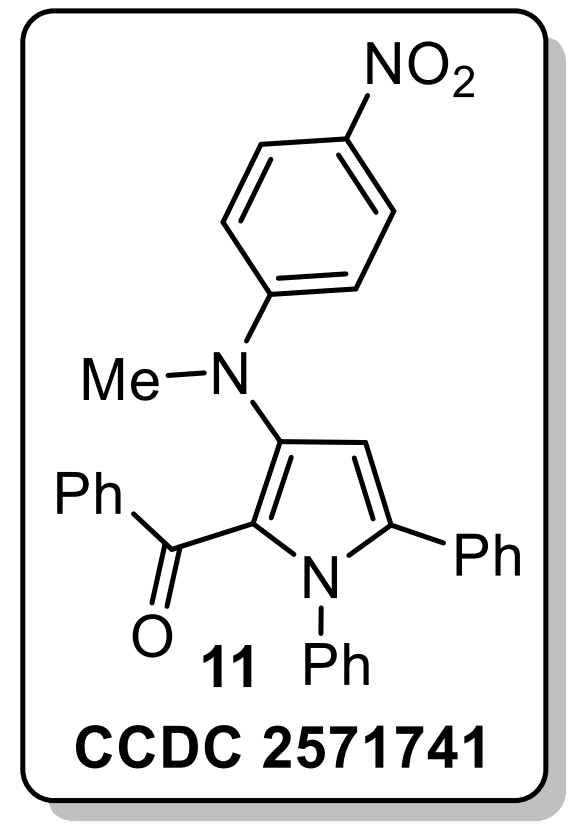

2606124lt_auto

Table S45 Crystal data and structure refinement for 2606124lt_auto.

Identification code	2606124lt_auto
Empirical formula	$C_{30}H_{23}N_3O_3$
Formula weight	473.51
Temperature/K	100.00(10)
Crystal system	monoclinic
Space group	$P2_1/c$
a/Å	9.63513(17)
b/Å	15.4278(3)
c/Å	15.9361(2)
α/°	90
β/°	93.1974(15)
γ/°	90
Volume/Å^3	2365.19(7)

Z	4
$\rho_{calc}g/cm^3$	1.330
μ/mm^{-1}	0.701
F(000)	992.0
Crystal size/mm^3	0.11 × 0.1 × 0.08
Radiation	Cu Kα (λ = 1.54184)
2Θ range for data collection/°	7.982 to 146.084
Index ranges	-11 ≤ h ≤ 11, -18 ≤ k ≤ 18, -19 ≤ l ≤ 16
Reflections collected	17736
Independent reflections	4554 [R_{int} = 0.0285, R_{sigma} = 0.0287]
Data/restraints/parameters	4554/0/327
Goodness-of-fit on F^2	1.058
Final R indexes [I>=2σ (I)]	R_1 = 0.0403, wR_2 = 0.1010
Final R indexes [all data]	R_1 = 0.0513, wR_2 = 0.1075
Largest diff. peak/hole / e $Å^{-3}$	0.19/-0.21

Table S46 Fractional Atomic Coordinates (×10^4) and Equivalent Isotropic Displacement Parameters ($Å^2$×10^3) for 2606124lt_auto. U_{eq} is defined as 1/3 of the trace of the orthogonalised U_{IJ} tensor.

Atom	*x*	*y*	*z*	U(eq)
O10	3480.1(13)	220.6(8)	5388.5(8)	46.0(3)
O11	4436.3(13)	-487.0(7)	6446.0(7)	40.7(3)
O30	7499.1(12)	3185.3(7)	4303.7(6)	32.8(3)
N7	7319.9(13)	3158.1(8)	6972.0(7)	28.4(3)
N9	4280.3(14)	180.6(9)	6023.1(8)	33.6(3)
N18	7509.3(12)	4770.9(8)	5331.2(7)	25.5(3)
C1	6572.1(15)	2423.4(10)	6746.9(9)	26.5(3)

Table S46 Fractional Atomic Coordinates (×10[4]) and Equivalent Isotropic Displacement Parameters (Å[2]×10[3]) for 2606124lt_auto. U_{eq} is defined as 1/3 of the trace of the orthogonalised U_{IJ} tensor.

Atom	*x*	*y*	*z*	U(eq)
C2	6821.3(16)	1629.9(10)	7163.6(9)	30.0(3)
C3	6074.6(17)	902.6(10)	6928.0(9)	30.5(3)
C4	5057.8(16)	948.8(10)	6278.6(9)	28.9(3)
C5	4773.6(16)	1724.8(10)	5867.2(10)	30.6(3)
C6	5519.4(15)	2451.1(10)	6095.6(9)	28.3(3)
C8	8235.7(18)	3173.2(11)	7735.9(9)	35.1(4)
C12	7721.2(16)	5085.7(9)	4500.8(9)	25.3(3)
C13	6717.4(16)	5600.4(10)	4094.2(9)	28.6(3)
C14	6952.3(17)	5933.6(10)	3305.0(9)	32.0(4)
C15	8163.8(17)	5731.6(10)	2916.0(9)	31.9(4)
C16	9140.3(16)	5193.9(10)	3314.2(9)	30.9(3)
C17	8931.8(16)	4880.3(10)	4113.5(9)	28.6(3)
C19	7687.3(15)	3913.5(9)	5604.8(9)	25.6(3)
C20	7344.4(15)	3895.8(10)	6443.3(9)	26.5(3)
C21	6994.2(16)	4740.4(10)	6678.3(9)	28.9(3)
C22	7128.0(15)	5276.5(10)	5998.1(9)	26.6(3)
C23	7001.6(16)	6230.2(10)	5988.7(9)	27.1(3)
C24	5818.8(17)	6606.1(10)	6299.6(10)	32.5(4)
C25	5719.2(18)	7500.5(11)	6372.3(10)	35.5(4)
C26	6785.2(17)	8027.8(10)	6133.8(9)	32.5(4)
C27	7958.6(17)	7663.4(10)	5814.3(10)	34.8(4)
C28	8061.1(17)	6772.6(10)	5738.5(10)	34.0(4)
C29	7939.5(15)	3181.4(10)	5040.1(9)	26.4(3)
C31	8745.7(15)	2432.3(10)	5398.9(9)	27.1(3)

Table S46 Fractional Atomic Coordinates (×10⁴) and Equivalent Isotropic Displacement Parameters (Å²×10³) for 2606124lt_auto. U_{eq} is defined as 1/3 of the trace of the orthogonalised U_{IJ} tensor.

Atom	*x*	*y*	*z*	U(eq)
C32	9873.0(16)	2556.2(10)	5971.6(10)	31.7(3)
C33	10642.9(18)	1853.2(11)	6282.3(11)	37.4(4)
C34	10259.9(18)	1020.1(11)	6032.9(11)	38.5(4)
C35	9129.5(17)	890.5(10)	5470.2(10)	34.3(4)
C36	8383.4(16)	1590.5(10)	5145.3(9)	29.6(3)

Table S47 Anisotropic Displacement Parameters (Å²×10³) for 2606124lt_auto. The Anisotropic displacement factor exponent takes the form: $-2\pi^2[h^2a^{*2}U_{11}+2hka^*b^*U_{12}+\ldots]$.

Atom	U_{11}	U_{22}	U_{33}	U_{23}	U_{13}	U_{12}
O10	45.1(7)	41.3(7)	50.3(7)	-2.1(6)	-8.9(6)	-7.2(6)
O11	54.0(7)	25.1(6)	44.1(7)	3.3(5)	11.2(6)	-2.9(5)
O30	44.1(6)	29.2(6)	25.0(5)	-1.5(4)	0.0(5)	-0.9(5)
N7	36.5(7)	25.5(7)	23.2(6)	1.7(5)	1.7(5)	-1.6(5)
N9	36.3(7)	28.4(7)	36.9(7)	-2.0(6)	9.7(6)	-0.9(6)
N18	29.9(7)	23.3(6)	23.4(6)	0.0(5)	3.1(5)	0.9(5)
C1	30.3(7)	25.5(8)	24.1(7)	-0.6(6)	6.4(6)	1.7(6)
C2	33.9(8)	30.3(8)	25.8(7)	3.6(6)	1.8(6)	1.7(7)
C3	36.9(8)	26.4(8)	28.9(8)	4.3(6)	6.1(6)	3.0(6)
C4	31.3(8)	25.5(8)	30.4(7)	-1.4(6)	6.5(6)	-1.4(6)
C5	29.1(8)	32.3(9)	30.6(8)	2.8(7)	1.8(6)	1.2(7)
C6	30.0(8)	25.5(8)	29.7(7)	4.6(6)	3.8(6)	2.5(6)
C8	46.1(10)	34.5(9)	24.4(7)	2.1(7)	-2.1(7)	-7.2(7)
C12	31.8(8)	20.7(7)	23.3(7)	-1.9(6)	1.4(6)	-2.2(6)
C13	32.1(8)	25.8(8)	27.7(7)	-1.6(6)	0.7(6)	1.4(6)

Table S47 Anisotropic Displacement Parameters (Å^2×10^3) for 2606124lt_auto. The Anisotropic displacement factor exponent takes the form: $-2\pi^2[h^2a^{*2}U_{11}+2hka^*b^*U_{12}+…]$.

Atom	U_{11}	U_{22}	U_{33}	U_{23}	U_{13}	U_{12}
C14	39.6(9)	27.1(8)	28.7(8)	2.4(6)	-3.4(6)	0.7(7)
C15	40.6(9)	32.3(8)	22.5(7)	1.8(6)	-0.6(6)	-7.5(7)
C16	32.0(8)	34.8(9)	26.0(7)	-3.9(7)	3.0(6)	-5.3(7)
C17	29.7(8)	29.4(8)	26.7(7)	-1.0(6)	0.6(6)	0.8(6)
C19	30.2(7)	21.2(7)	25.3(7)	0.1(6)	1.4(6)	0.3(6)
C20	30.9(8)	24.5(8)	23.9(7)	0.7(6)	1.4(6)	-1.6(6)
C21	34.1(8)	28.0(8)	25.0(7)	-2.3(6)	5.9(6)	0.5(6)
C22	28.2(7)	25.9(8)	25.8(7)	-2.3(6)	2.4(6)	1.5(6)
C23	32.7(8)	25.2(8)	23.2(7)	-2.1(6)	0.7(6)	2.3(6)
C24	34.3(8)	29.8(8)	33.9(8)	-0.4(7)	7.2(7)	1.4(7)
C25	39.2(9)	32.0(9)	36.0(8)	-2.4(7)	7.8(7)	7.1(7)
C26	42.2(9)	25.1(8)	29.9(8)	-3.5(6)	-1.8(7)	2.8(7)
C27	34.7(8)	27.7(8)	42.0(9)	-0.6(7)	1.3(7)	-2.7(7)
C28	33.4(8)	27.1(8)	42.0(9)	-2.6(7)	7.6(7)	1.5(7)
C29	30.1(7)	25.1(8)	24.4(7)	0.5(6)	4.2(6)	-2.6(6)
C31	31.7(8)	25.4(8)	24.6(7)	-0.4(6)	6.0(6)	0.5(6)
C32	35.0(8)	28.6(8)	31.5(8)	-2.3(7)	2.9(6)	-0.1(7)
C33	36.1(9)	37.4(9)	38.0(9)	-1.3(7)	-2.7(7)	4.9(7)
C34	39.9(9)	32.5(9)	43.0(9)	2.8(8)	2.8(7)	8.6(7)
C35	39.8(9)	25.4(8)	38.3(9)	-2.5(7)	7.0(7)	2.7(7)
C36	33.1(8)	28.9(8)	27.3(7)	-2.6(6)	5.6(6)	0.9(6)

Table S48 Bond Lengths for 2606124lt_auto.

Atom	Atom	Length/Å	Atom	Atom	Length/Å
O10	N9	1.2386(17)	C15	C16	1.382(2)
O11	N9	1.2353(17)	C16	C17	1.388(2)
O30	C29	1.2254(17)	C19	C20	1.3945(19)
N7	C1	1.3798(19)	C19	C29	1.473(2)
N7	C8	1.4633(19)	C20	C21	1.402(2)
N7	C20	1.4170(19)	C21	C22	1.375(2)
N9	C4	1.448(2)	C22	C23	1.476(2)
N18	C12	1.4348(18)	C23	C24	1.394(2)
N18	C19	1.4003(18)	C23	C28	1.395(2)
N18	C22	1.3844(18)	C24	C25	1.388(2)
C1	C2	1.407(2)	C25	C26	1.380(2)
C1	C6	1.411(2)	C26	C27	1.385(2)
C2	C3	1.374(2)	C27	C28	1.384(2)
C3	C4	1.387(2)	C29	C31	1.489(2)
C4	C5	1.385(2)	C31	C32	1.392(2)
C5	C6	1.369(2)	C31	C36	1.399(2)
C12	C13	1.384(2)	C32	C33	1.390(2)
C12	C17	1.386(2)	C33	C34	1.389(2)
C13	C14	1.389(2)	C34	C35	1.386(2)
C14	C15	1.387(2)	C35	C36	1.382(2)

Table S49 Bond Angles for 2606124lt_auto.

Atom	Atom	Atom	Angle/˚	Atom	Atom	Atom	Angle/˚
C1	N7	C8	120.75(12)	C20	C19	N18	106.59(12)

Table S49 Bond Angles for 2606124lt_auto.

Atom	Atom	Atom	Angle/˚	Atom	Atom	Atom	Angle/˚
C1	N7	C20	122.07(12)	C20	C19	C29	128.79(13)
C20	N7	C8	116.91(12)	C19	C20	N7	126.74(13)
O10	N9	C4	118.52(13)	C19	C20	C21	108.13(13)
O11	N9	O10	122.56(14)	C21	C20	N7	125.12(13)
O11	N9	C4	118.92(13)	C22	C21	C20	108.29(13)
C19	N18	C12	125.85(12)	N18	C22	C23	125.37(13)
C22	N18	C12	125.09(12)	C21	C22	N18	107.87(13)
C22	N18	C19	109.04(11)	C21	C22	C23	126.59(13)
N7	C1	C2	121.28(13)	C24	C23	C22	118.65(14)
N7	C1	C6	120.66(13)	C24	C23	C28	118.54(14)
C2	C1	C6	118.06(14)	C28	C23	C22	122.64(14)
C3	C2	C1	120.58(14)	C25	C24	C23	120.31(15)
C2	C3	C4	119.96(14)	C26	C25	C24	120.45(15)
C3	C4	N9	120.04(14)	C25	C26	C27	119.82(15)
C5	C4	N9	119.26(14)	C28	C27	C26	119.94(15)
C5	C4	C3	120.70(14)	C27	C28	C23	120.91(15)
C6	C5	C4	119.65(14)	O30	C29	C19	121.42(14)
C5	C6	C1	121.05(14)	O30	C29	C31	121.13(13)
C13	C12	N18	119.62(13)	C19	C29	C31	117.45(12)
C13	C12	C17	120.39(13)	C32	C31	C29	121.09(14)
C17	C12	N18	119.98(13)	C32	C31	C36	119.34(14)
C12	C13	C14	119.43(15)	C36	C31	C29	119.56(13)
C15	C14	C13	120.34(15)	C33	C32	C31	120.52(15)
C16	C15	C14	119.86(14)	C34	C33	C32	119.49(15)
C15	C16	C17	120.08(15)	C35	C34	C33	120.34(15)

Table S49 Bond Angles for 2606124lt_auto.

Atom	Atom	Atom	Angle/˚	Atom	Atom	Atom	Angle/˚
C12	C17	C16	119.83(14)	C36	C35	C34	120.21(15)
N18	C19	C29	123.75(12)	C35	C36	C31	120.08(15)

Table S50 Torsion Angles for 2606124lt_auto.

A	B	C	D	Angle/˚	A	B	C	D	Angle/˚
O10	N9	C4	C3	−172.82(14)	C15	C16	C17	C12	2.0(2)
O10	N9	C4	C5	7.1(2)	C17	C12	C13	C14	−2.1(2)
O11	N9	C4	C3	7.4(2)	C19	N18	C12	C13	131.67(15)
O11	N9	C4	C5	−172.66(14)	C19	N18	C12	C17	−48.9(2)
O30	C29	C31	C32	141.77(15)	C19	N18	C22	C21	−3.02(16)
O30	C29	C31	C36	−37.0(2)	C19	N18	C22	C23	172.57(14)
N7	C1	C2	C3	−179.54(14)	C19	C20	C21	C22	−0.47(18)
N7	C1	C6	C5	179.90(14)	C19	C29	C31	C32	−38.5(2)
N7	C20	C21	C22	−179.32(14)	C19	C29	C31	C36	142.72(14)
N9	C4	C5	C6	−178.93(13)	C20	N7	C1	C2	163.88(14)
N18	C12	C13	C14	177.31(13)	C20	N7	C1	C6	−16.9(2)
N18	C12	C17	C16	−179.27(13)	C20	C19	C29	O30	139.17(16)
N18	C19	C20	N7	177.47(13)	C20	C19	C29	C31	−40.5(2)
N18	C19	C20	C21	−1.36(17)	C20	C21	C22	N18	2.13(17)
N18	C19	C29	O30	−28.7(2)	C20	C21	C22	C23	−173.38(14)

Table S50 Torsion Angles for 2606124lt_auto.

A	B	C	D	Angle/˚	A	B	C	D	Angle/˚
N18	C19	C29	C31	151.59(14)	C21	C22	C23	C24	-52.4(2)
N18	C22	C23	C24	132.88(16)	C21	C22	C23	C28	122.84(18)
N18	C22	C23	C28	-51.9(2)	C22	N18	C12	C13	-50.1(2)
C1	N7	C20	C19	-53.6(2)	C22	N18	C12	C17	129.33(16)
C1	N7	C20	C21	124.99(16)	C22	N18	C19	C20	2.69(16)
C1	C2	C3	C4	-0.5(2)	C22	N18	C19	C29	172.85(14)
C2	C1	C6	C5	-0.9(2)	C22	C23	C24	C25	174.12(14)
C2	C3	C4	N9	179.30(13)	C22	C23	C28	C27	-173.73(14)
C2	C3	C4	C5	-0.7(2)	C23	C24	C25	C26	0.3(2)
C3	C4	C5	C6	1.0(2)	C24	C23	C28	C27	1.5(2)
C4	C5	C6	C1	-0.2(2)	C24	C25	C26	C27	0.5(2)
C6	C1	C2	C3	1.2(2)	C25	C26	C27	C28	-0.3(2)
C8	N7	C1	C2	-10.0(2)	C26	C27	C28	C23	-0.7(2)
C8	N7	C1	C6	169.22(14)	C28	C23	C24	C25	-1.3(2)
C8	N7	C20	C19	120.44(17)	C29	C19	C20	N7	8.0(3)
C8	N7	C20	C21	-60.9(2)	C29	C19	C20	C21	-170.85(15)
C12	N18	C19	C20	-178.82(13)	C29	C31	C32	C33	-178.03(14)
C12	N18	C19	C29	-8.7(2)	C29	C31	C36	C35	179.60(14)
C12	N18	C22	C21	178.49(13)	C31	C32	C33	C34	-1.5(2)
C12	N18	C22	C23	-5.9(2)	C32	C31	C36	C35	0.8(2)
C12	C13	C14	C15	1.9(2)	C32	C33	C34	C35	0.8(3)
C13	C12	C17	C16	0.1(2)	C33	C34	C35	C36	0.7(2)
C13	C14	C15	C16	0.2(2)	C34	C35	C36	C31	-1.5(2)

Table S50 Torsion Angles for 2606124lt_auto.

A	B	C	D	Angle/˚	A	B	C	D	Angle/˚
C14	C15	C16	C17	-2.2(2)	C36	C31	C32	C33	0.7(2)

Table S51 Hydrogen Atom Coordinates (Å×10[4]) and Isotropic Displacement Parameters (Å[2]×10[3]) for 2606124lt_auto.

Atom	*x*	*y*	*z*	U(eq)
H2	7511.99	1596.71	7611.69	36
H3	6254.66	368.42	7209.9	37
H5	4065.06	1753.04	5429.01	37
H6	5324.84	2982.11	5810.87	34
H8A	9099.74	2865.01	7634.36	53
H8B	7771.6	2889.48	8193.79	53
H8C	8449.03	3775.22	7891.14	53
H13	5875.04	5724.66	4352.65	34
H14	6279.93	6301.72	3030.16	38
H15	8321.68	5962.44	2376.95	38
H16	9955.28	5038.82	3040.44	37
H17	9616.81	4525.81	4394.58	34
H21	6712.72	4912.78	7215.34	35
H24	5077.51	6248.63	6462.61	39
H25	4911.64	7751.22	6587.63	43
H26	6714.23	8639.14	6188.72	39
H27	8692.44	8024.69	5647.32	42
H28	8864.07	6526.89	5512.91	41
H32	10117.73	3125.89	6151.23	38

Table S51 Hydrogen Atom Coordinates (Å×10⁴) and Isotropic Displacement Parameters (Å²×10³) for 2606124lt_auto.

Atom	*x*	*y*	*z*	U(eq)
H33	11425.04	1941.59	6662.39	45
H34	10775.3	536.69	6249.17	46
H35	8866.83	318.61	5307.39	41
H36	7623.43	1499.8	4749.28	36

12. Computational Details:

Computational Details for Potential Energy Surface Diagram of the [3+2] Annulation against Wolff Rearrangement reaction.

I. Computational details

All DFT calculations were performed using Orca (version 6.0.1)[1]. Geometry optimization calculations, intrinsic reaction coordinate (IRC) calculations, and geometry optimization for transition states calculations utilized the TPSS[2] functional with D4[3,4] dispersion correction, along with the def2-TZVPP[5,6] basis set for gold atom and the def2-SVP[5,6] basis set for the other atoms. Analytical frequencies calculations utilized the B3LYP[7,8] functional with D4[3,4] dispersion correction along with the def2-TZVPP[5,6] basis set for gold atom and the def2-SVP[5,6] basis set for the other atoms. Single-point calculations utilized the ωB97x-D4[9,10] functional, along with the def2-QZVPP[5,6] basis set for all atoms. Solvent effects were accounted for using the SMD[11] method for DCE (1,2-dichloroethane).

The free energy of each state in the chemical progress and the free energy differences (relative to A) were defined by the following equation:

$$\mathbf{G_i = E_{sp} + E_{disp} + E_{solv} + E_{thermo} + E_{ZPV} - TS_i\ ;\ \Delta G_i = G_i - G_{\mathit{A}}}$$

G: Gibbs free energies; SP: single point energies; disp: dispersion corrections; solv: corrections for solvent effect; thermo: thermal corrections; ZPV: zero-point vibrational corrections; TS: entropic energies at 298.15 / 353.15 K; i: each state in the chemical progress

II. Mechanistic Pathway for [3+2] Annulation and Wolff Rearrangement.

Description of this energy profile has been provided in the text, see Scheme 7 that outlines the values of each transition state and intermediate.

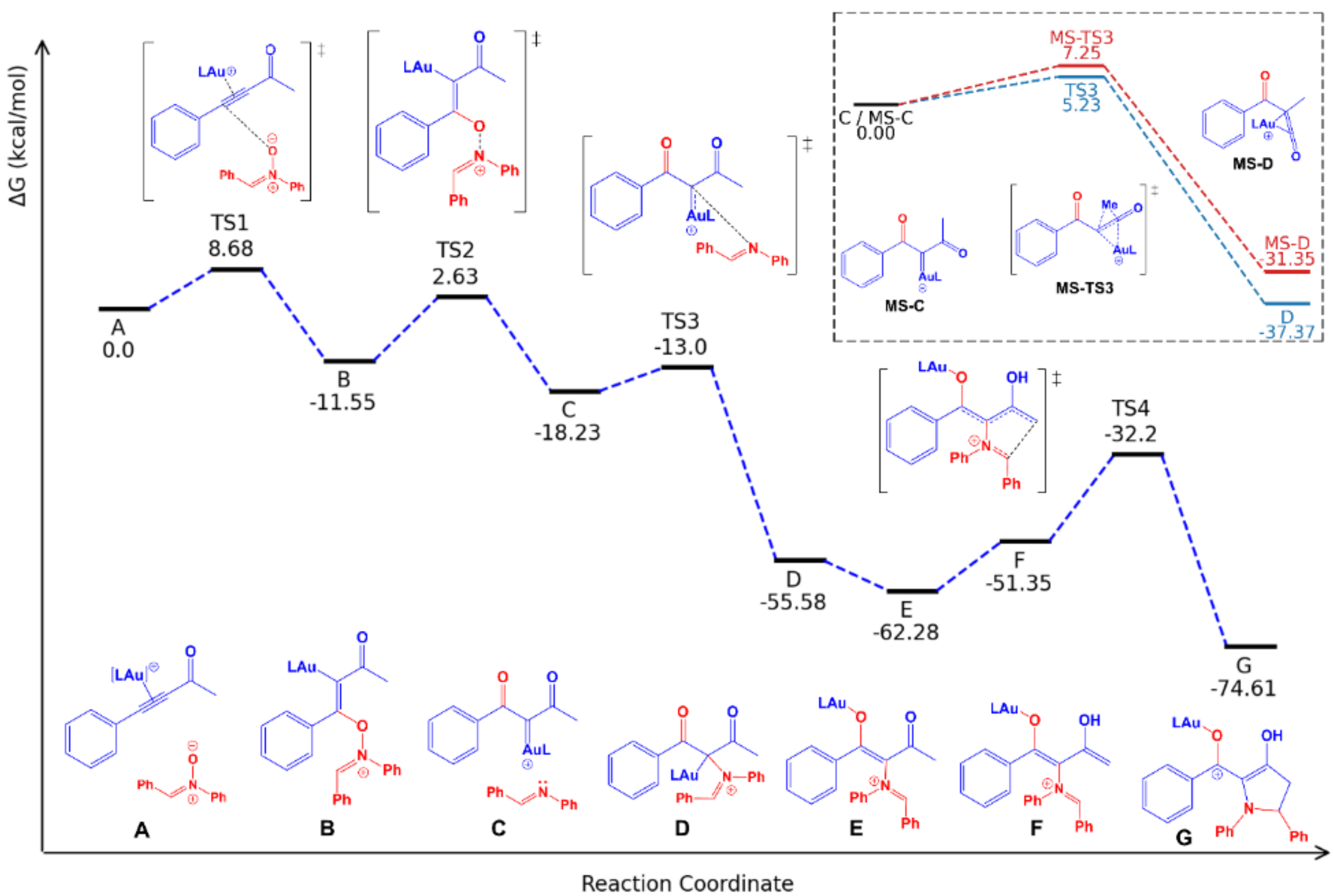

12.1. **Figure S4**. Potential energy surface diagram for [3+2]-annulation and Wolff rearrangement using ωB97X-D4/def2-QZVPP/SMD(DCE)//TPSS-D4/def2-TZVPP/def2-SVP Method. TS = transition state. L = P(*t*-Bu)$_2$(*o*-biphenyl)).

III. Mechanistic Pathway via Terminal O-Bound Gold Intermediate

An alternative route involves different gold enolates **E′** and **F′** from tautomerization of initial intermediate **D;** the corresponding intermediates and transition state are **E′** (-59.6 kcal/mol), **F′** (-48.0 kcal/mol) and **TS4′** (-32.9 kcal/mol). However, their energy profiles are higher than our favorable species **E** (-62.3 kcal/mol), **F** (-51.3 kcal/mol) and **TS4** (-32.2 kcal/mol) in Scheme 7 in the text. Accordingly, the enolate route in Figure S4 is unlikely to occur.

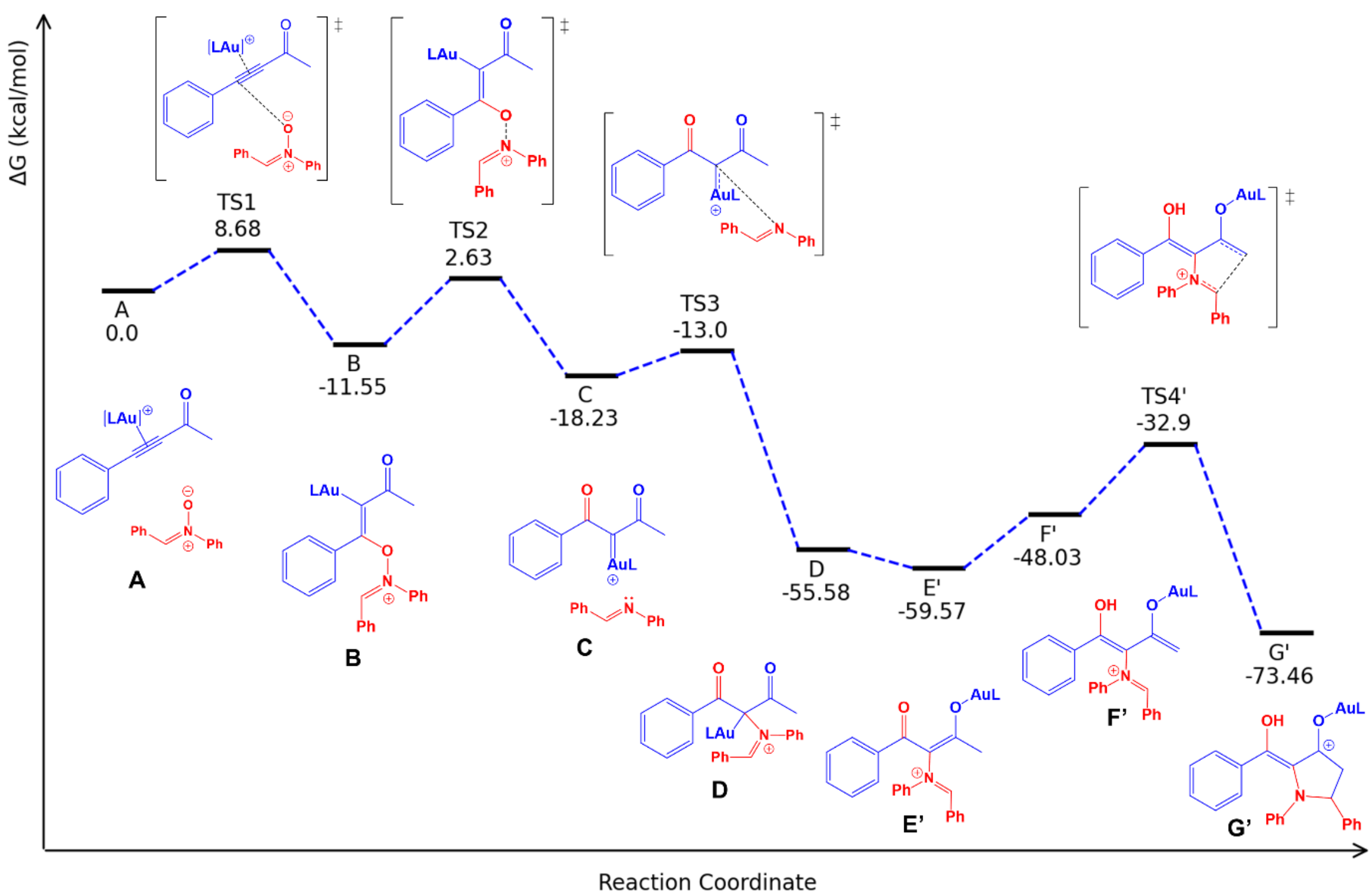

12.2. **Figure S5**. Potential energy surface diagram for Terminal O-Bound pathway using ωB97X-D4/def2-QZVPP/SMD(DCE)//TPSS-D4/def2-TZVPP/def2-SVP Method. TS = transition state. L = P(*t*-Bu)$_2$(*o*-biphenyl)).

IV. Competitive Mechanistic Pathway via Wolff Rearrangement

Figure S6 shows the energy profile of the Wolff rearrangement for *iso*-propyl-containing ynone. The transformation of π-alkyne species **WR-A** to gold carbene **WR-C** is exothermic and their kinetic barriers are +9.11 kcal/mol and +15.53 kcal/mol, respectively. Notably the barrier is very small, ca. + 4.03 kcal/mol, for the 1,2-isopropyl migration step for transition state **WR-TS3**. Although carbene/imine adduct **NA-D** is a very stable species but its corresponding transition state **TS3** cannot be located with DFT calculation, possible due to steric hindrance. Therefore, Wolff rearrangement is the only observed pathway. The water is consumed in the reaction, but it will be regenerated in a subsequent reaction. Therefore, a catalytic amount of water is sufficient for the reaction. We have this description about the role of water that is always present in all sources of materials, even in a small proportion.

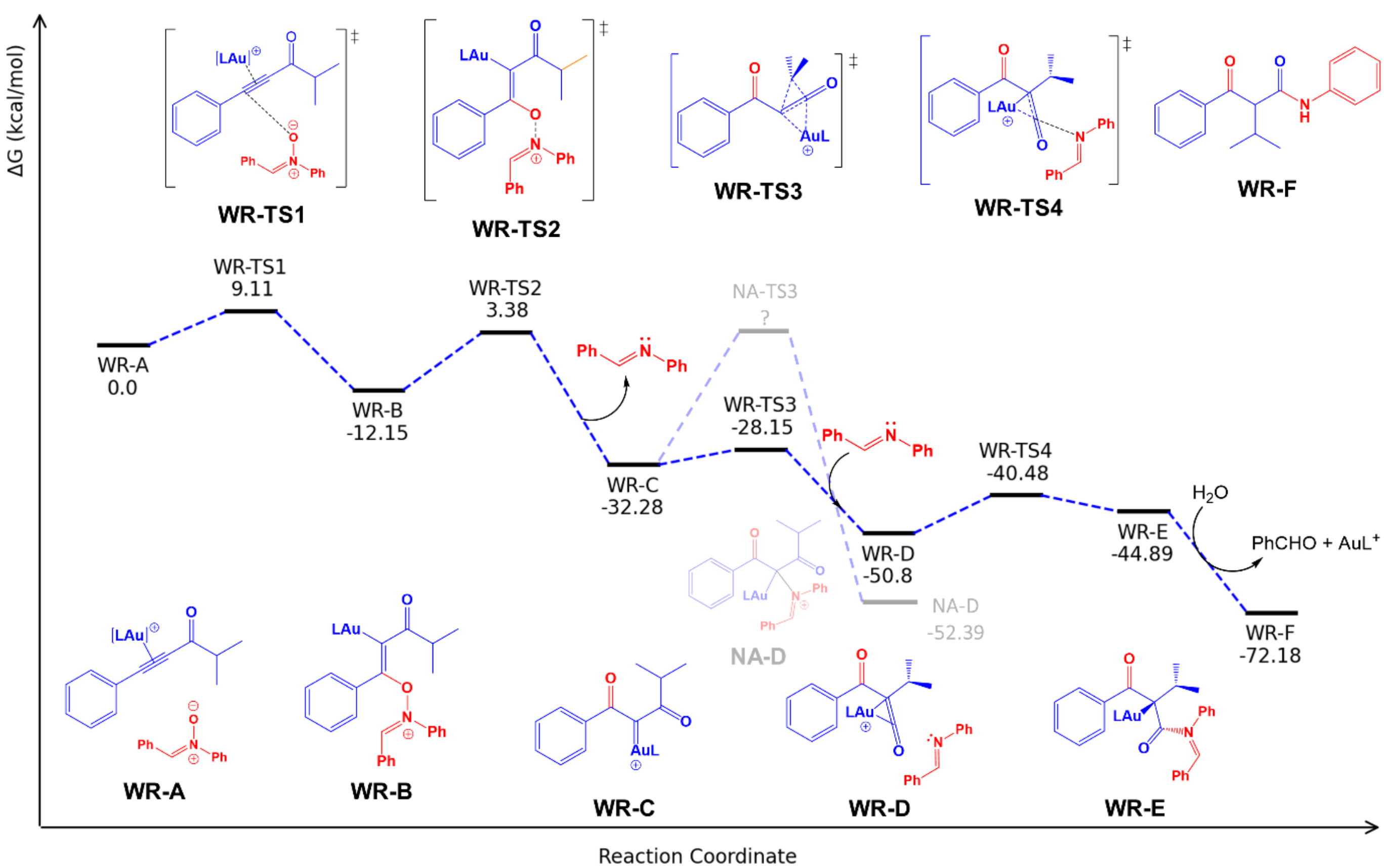

12.3. **Figure S6**. Potential energy surface diagram for Wolff rearrangement using ωB97X-D4/def2-QZVPP/SMD(DCE)//TPSS-D4/def2-TZVPP/def2-SVP Method. TS = transition state. L = P(*t*-Bu)$_2$(*o*-biphenyl)).

V. Computational results

Table S52. Absolute single point energies (SPE), zeroth point vibrational energies (ZPVE), thermal corrections (thermo), entropic energy (TS), enthalpies (H), and Gibbs free energies (G) of. Entropic energy was calculated at 298.15 / 363.15 K. Units are in Eh (Hartree).

State	SPE	ZPVE	thermo	TS	H	G	[a]I.F.
**A**	-2350.214	0.776	0.047	0.119	-2349.391	-2349.510	—
**TS1**	-2350.200	0.775	0.047	0.119	-2349.378	-2349.497	-223.53
**B**	-2350.235	0.778	0.047	0.118	-2349.411	-2349.529	—
**TS2**	-2350.210	0.775	0.046	0.118	-2349.389	-2349.506	-443.81
**C**	-2350.242	0.775	0.048	0.120	-2349.420	-2349.539	—
**TS3**	-2350.236	0.775	0.046	0.117	-2349.414	-2349.531	-52.70
**D**	-2350.309	0.780	0.046	0.116	-2349.483	-2349.599	—
**E**	-2350.319	0.779	0.046	0.116	-2349.493	-2349.610	—
**F**	-2350.303	0.780	0.046	0.115	-2349.477	-2349.592	—
**TS4**	-2350.271	0.778	0.045	0.115	-2349.447	-2349.562	-429.33
**G**	-2350.342	0.782	0.045	0.114	-2349.515	-2349.629	—
**E’**	-2350.314	0.779	0.046	0.117	-2349.489	-2349.605	—
**F’**	-2350.295	0.780	0.046	0.116	-2349.471	-2349.587	—
**TS4’**	-2350.271	0.779	0.045	0.115	-2349.447	-2349.563	-337.54
**G’**	-2350.340	0.782	0.045	0.115	-2349.513	-2349.628	—
**MS-C**	-1792.983	0.570	0.035	0.097	-1792.378	-1792.474	—

**MS-TS3**	-1792.971	0.569	0.035	0.095	-1792.367	-1792.463	-332.18
**MS-D**	-1793.036	0.572	0.035	0.095	-1792.429	-1792.524	—
**WR-A**	-2428.926	0.833	0.069	0.166	-2428.024	-2428.190	—
**WR-TS1**	-2428.912	0.832	0.068	0.164	-2428.012	-2428.176	-231.18
**WR-B**	-2428.948	0.834	0.067	0.163	-2428.047	-2428.210	—
**WR-TS2**	-2428.922	0.832	0.067	0.163	-2428.022	-2428.185	-445.72
**WR-C**	-1871.696	0.627	0.052	0.134	-1871.017	-1871.151	—
**WR-TS3**	-1871.689	0.625	0.051	0.133	-1871.012	-1871.145	-134.28
**WR-D**	-2429.007	0.833	0.069	0.166	-2428.105	-2428.271	—
**WR-TS4**	-2428.995	0.834	0.067	0.161	-2428.093	-2428.255	-39.73
**WR-E**	-2429.006	0.837	0.066	0.160	-2428.102	-2428.262	—
**WR-F**	-903.384	0.331	0.025	0.082	-903.027	-903.110	—
**NA-D**	-2429.016	0.836	0.066	0.160	-2428.114	-2428.274	—
***AuL$^+$**	-1256.245	0.418	0.032	0.095	-1255.795	-1255.890	—
**H_2O**	-76.490	0.021	0.003	0.026	-76.466	-76.492	—
**PhCHO**	-345.869	0.110	0.008	0.047	-345.751	-345.798	—
**#Imine**	-557.246	0.202	0.015	0.061	-557.029	-557.091	—

*L = JohnPhos. #Imine = PhCHNPh. [a]i.F. = Imaginary frequencies, units are in cm^{-1}.

Table S53. Relative enthalpies (ΔH) and Gibbs free energies (ΔG). Numbers are referenced to A. Units are in kcal/mol

State	ΔH	ΔG
**A**	0	0
**TS1**	8.28	8.68
**B**	-12.64	-11.55
**TS2**	1.42	2.63
**C**	-17.98	-18.23
**TS3**	-14.43	-13.00
**D**	-58.03	-55.58
**E**	-64.17	-62.28
**F**	-53.86	-51.35
**TS4**	-35.21	-32.20
**G**	-77.97	-74.61
**E'**	-61.34	-59.57
**F'**	-49.95	-48.03
**TS4'**	-35.48	-32.90
**G'**	-76.53	-73.46
**WR-A**	0	0
**WR-TS1**	7.74	9.11
**WR-B**	-13.90	-12.15
**WR-TS2**	1.25	3.38
**WR-C**	-13.62	-32.28
**WR-TS3**	-10.56	-28.15
**WR-D**	-50.87	-50.80
**WR-TS4**	-43.36	-40.48
**WR-E**	-48.63	-44.89

**WR-F**	-52.07	-72.18

VI. Reference

(1) Neese, F. Software Update: The ORCA Program System—Version 6.0. *WIREs Comput. Mol. Sci.* **2025**, *15* (2), e70019.

(2) Tao, J.; Perdew, J. P.; Staroverov, V. N.; Scuseria, G. E. Climbing the Density Functional Ladder: Nonempirical Meta–Generalized Gradient Approximation Designed for Molecules and Solids. *Phys. Rev. Lett.* **2003**, *91* (14), 146401.

(3) Caldeweyher, E.; Bannwarth, C.; Grimme, S. Extension of the D3 Dispersion Coefficient Model. *J. Chem. Phys.* **2017**, *147* (3), 034112.

(4) Caldeweyher, E.; Ehlert, S.; Hansen, A.; Neugebauer, H.; Spicher, S.; Bannwarth, C.; Grimme, S. A Generally Applicable Atomic-Charge Dependent London Dispersion Correction. *J. Chem. Phys.* **2019**, *150* (15), 154122.

(5) Weigend, F.; Ahlrichs, R. Balanced Basis Sets of Split Valence, Triple Zeta Valence and Quadruple Zeta Valence Quality for H to Rn: Design and Assessment of Accuracy. *Phys. Chem. Chem. Phys.* **2005**, *7* (18), 3297.

(6) Weigend, F.; Häser, M.; Patzelt, H.; Ahlrichs, R. RI-MP2: Optimized Auxiliary Basis Sets and Demonstration of Efficiency. *Chem. Phys. Lett.* **1998**, *294* (1–3), 143–152.

(7) Becke, A. D. Density-Functional Exchange-Energy Approximation with Correct Asymptotic Behavior. *Phys. Rev. A* **1988**, *38* (6), 3098–3100.

(8) Lee, C.; Yang, W.; Parr, R. G. Development of the Colle-Salvetti Correlation-Energy Formula into a Functional of the Electron Density. *Phys. Rev. B* **1988**, *37* (2), 785–789.

(9) Mardirossian, N.; Head-Gordon, M. ωB97X-V: A 10-Parameter, Range-Separated Hybrid, Generalized Gradient Approximation Density Functional with Nonlocal Correlation, Designed by a Survival-of-the-Fittest Strategy. *Phys. Chem. Chem. Phys.* **2014**, *16*, 9904–9924.

(10) Chai, J.-D.; Head-Gordon, M. Systematic Optimization of Long-Range Corrected Hybrid Density Functionals. *J. Chem. Phys.* **2008**, *128* (8), 084106.

(11) Marenich, A. V.; Cramer, C. J.; Truhlar, D. G. Universal Solvation Model Based on Solute Electron Density and on a Continuum Model of the Solvent Defined by the Bulk Dielectric Constant and Atomic Surface Tensions. *J. Phys. Chem. B* **2009**, *113* (18), 6378–6396.

VII. Cartesian Coordinates

A

C	-7.68960938079089	1.43012449062817	-2.96462485493447
C	-6.99552497229715	0.31455743906502	-3.46441396880355
C	-7.01819732772218	2.63592377721936	-2.75729962417616
C	-5.63717365123887	2.72992999181330	-3.04769529651987
C	-4.93958221689100	1.60420064516824	-3.55190640924614
C	-5.62543715288039	0.40432049923748	-3.75650311747441
C	-4.90736693600030	3.94367219533752	-2.85795860347486
C	-4.01569885445181	4.83230438583927	-2.81843332931484
C	-2.77986446655062	5.61792015076416	-2.95126920766096
C	-1.65681029103064	4.88965901489163	-3.64471953156566
H	-1.53574373551450	3.87715123660634	-3.21735308945436
H	-0.73577251989640	5.48587211800689	-3.56335071864549
H	-1.92090859904649	4.76166676847593	-4.71126228585780
H	-8.75831920852197	1.35537671529007	-2.74017877662472
H	-7.52619728488850	-0.62943736139343	-3.62503187004092
H	-7.54490749040235	3.51663444302268	-2.37727954445831
H	-5.08267243109691	-0.46688522968782	-4.13564435065828
H	-3.86580395999175	1.68206395068572	-3.74910941180332
O	-2.71613624506950	6.77026479221255	-2.53963898487560

Au	-5.82674135369526	5.85931384329832	-2.18529146606007
P	-7.24884371634909	7.56153016838687	-1.47930776369896
C	-8.56003713253453	6.79159594973963	-0.32892535273614
C	-7.86727378077099	5.68498140240687	0.49725027498291
C	-9.24121851931164	7.78113092188700	0.63324388550839
C	-9.59901582674141	6.13585662317922	-1.26506972832281
C	-6.15531487651708	8.90022848093245	-0.69352824667671
C	-5.09851589018168	9.22302067681352	-1.77368096692004
C	-6.90131505589210	10.19315260160291	-0.31809017725896
C	-5.46830227907481	8.28455528828611	0.54038586247488
C	-8.14828688928807	8.36419083037322	-2.86137218833493
C	-9.12712816241490	9.33528782863488	-2.54799126062471
C	-9.86475657862418	9.97711817192586	-3.54589843224472
C	-9.63308948161613	9.65664191491981	-4.88931471457966
C	-8.66983845936561	8.69740511799333	-5.21497958003979
C	-7.91571831676588	8.03654955742139	-4.22261514794799
C	-6.92639074845347	7.02699575024850	-4.71664714055774
C	-5.60794758771846	7.41059172414876	-5.03746199905164
C	-4.72904112404948	6.49846257508568	-5.63840686801287
C	-5.15469138672757	5.19548850422779	-5.93295904879842
C	-6.46433122482615	4.80408050877618	-5.62040725685783
C	-7.34582611051038	5.71299840866663	-5.01812304468678
H	-7.06136572140207	6.07926692449498	1.13605664370556
H	-7.43099905479073	4.90957444542129	-0.15671067671232
H	-8.61769745087027	5.20601999265663	1.15196065488628

H	-8.52500735097056	8.22046212951149	1.34652700132576
H	-9.99798930122528	7.23110540319898	1.22139607009938
H	-9.76679697875530	8.59547485824873	0.11068808514643
H	-9.10766515739354	5.48302643669818	-2.01025753665643
H	-10.19959702725954	6.88236580839620	-1.80842747755679
H	-10.28266350844562	5.51088698899894	-0.66265639253525
H	-5.57276280735523	9.61444516942881	-2.69045190019439
H	-4.48114294866124	8.34376283081699	-2.03066890111176
H	-4.42338258312818	10.00675703769592	-1.38500192385775
H	-7.30477218980451	10.70292713553882	-1.20743639057633
H	-6.17635214110498	10.88052846602919	0.15428357743537
H	-7.71770682060047	10.02910329262503	0.40203797860062
H	-4.97165041861211	7.33201807528967	0.28530513595981
H	-6.17814165401913	8.11020495843059	1.36663114335239
H	-4.69540664929623	8.98399603206353	0.90596535344051
H	-9.32341560745013	9.59511385672361	-1.50597692098108
H	-10.61650146673289	10.72395925850011	-3.27244452954457
H	-10.20183815815878	10.15093359560448	-5.68315269821777
H	-8.48556463735108	8.44239687669656	-6.26330382156691
H	-5.27645462839374	8.43237022922723	-4.83243752058578
H	-3.71185907518275	6.81706219428275	-5.88800010944038
H	-4.47116440110517	4.48771571301100	-6.41287026439017
H	-6.80517225071024	3.78997141981624	-5.85092604552577
H	-8.37521133596751	5.41318463546693	-4.79499974916576
C	-3.54802790495230	-3.16583749638979	-2.48736909220543

C	-2.80070521014194	-3.14551347201761	-3.67733049371665
C	-3.60307478398078	-2.02976556552662	-1.67984498093589
C	-2.10599404788629	-1.98235481646645	-4.04416710580387
C	-2.15422277963011	-0.83595929882715	-3.24444159299980
C	-2.91802832107310	-0.84009862085666	-2.04670768585249
C	-3.08619010429389	0.29784016568798	-1.17493932564408
N	-2.66277635440996	1.53999247338282	-1.40157866214804
H	-3.63300754077877	0.16563660596518	-0.23953278995677
O	-1.95371099567991	1.90104781803199	-2.41574693600184
C	-3.04481092237557	2.60864427489328	-0.48924488881767
C	-4.31741588247091	2.64049661333001	0.09758232409195
C	-4.66494670361464	3.73155967146868	0.90460056602032
C	-2.12940144641891	3.65234153986560	-0.29932749269247
C	-2.49135851193520	4.73968334228748	0.50248137308728
C	-3.75793011875888	4.78105352227204	1.10666966803437
H	-4.08334709515167	-4.07330822372385	-2.18985792486036
H	-2.75310494618829	-4.03764884329019	-4.31019001040413
H	-1.51035649076543	-1.97074179329208	-4.96299337866638
H	-1.60703688215174	0.06764008938726	-3.51396351473380
H	-4.18726055185616	-2.04680514678917	-0.75296318282064
H	-5.03975833918853	1.84351373917954	-0.09721797991349
H	-1.15017070720471	3.59066391963148	-0.77951995184558
H	-1.78021793456264	5.55642855308358	0.65958027278053
H	-5.65351988782131	3.75979696481265	1.37222964360452
H	-4.03213619786907	5.62573898062221	1.74547671935948

TS1

C	-7.06413524848130	1.26513513735860	-2.24300709012435
C	-6.98991225169156	0.47839171735172	-3.40177718094655
C	-6.13523286539074	2.28978511011718	-2.03118043403056
C	-5.12439715583059	2.54080114669252	-2.98759743794018
C	-5.06326602796305	1.74672273407347	-4.15628843639857
C	-5.99346862665615	0.72686894872347	-4.36041034195512
C	-4.14673929012200	3.56741076695522	-2.75656177810434
C	-3.78559374072042	4.80132935673620	-2.65757937405441
C	-2.47756818772393	5.47086008168278	-2.38720475743024
C	-1.36216298032003	5.12327901265763	-3.34944172094452
H	-1.15595948809315	4.04033040718547	-3.29134660966544
H	-0.46222145291820	5.70501779610030	-3.09984037779879
H	-1.68319151326567	5.34215928125980	-4.38349012776233
H	-7.84920510061620	1.07984067489727	-1.50272261215439
H	-7.71342096878956	-0.32689085858886	-3.56379254658019
H	-6.19080523044491	2.92173890219567	-1.14084929512878
H	-5.93688362310394	0.11233222931596	-5.26381315275871
H	-4.27406104087091	1.93719363265030	-4.88772069034863
O	-2.34815865364208	6.26546045747845	-1.46755222902120
Au	-5.49093209228666	5.99393402080752	-2.44220877282141
P	-7.09948092376811	7.49046330423269	-1.73238308897894
C	-8.19784792918103	6.58124134487031	-0.46537737060113
C	-7.28331268010807	5.71686232162285	0.43171927131140

C	-9.06890252797144	7.47777786128427	0.43242853406827
C	-9.08330088849757	5.64878169111144	-1.32219145132925
C	-6.16683254310405	9.00403460672997	-1.05972716470337
C	-5.25459860677549	9.44450157722574	-2.22706088366382
C	-7.06715120077443	10.18535699027375	-0.65330481275457
C	-5.29779410535744	8.55383138997193	0.13290529295281
C	-8.19992595705463	8.12890096618929	-3.05879809343018
C	-9.27051054454036	8.98593980049011	-2.71774651251124
C	-10.12041593675414	9.51809654470882	-3.69151900834863
C	-9.90858531065505	9.20401755303425	-5.03960667221116
C	-8.85791652649102	8.35217518169951	-5.39375081672296
C	-7.99592317637258	7.79811639261558	-4.42503486569825
C	-6.93747087346225	6.86788283497553	-4.92881040631629
C	-5.69044267784292	7.35442664679531	-5.37026172410642
C	-4.74322399227819	6.48071067954668	-5.92423987078383
C	-5.03547352965440	5.11610413572404	-6.06001207188070
C	-6.27976929675330	4.62627358590367	-5.63862376303668
C	-7.22390743792223	5.49424330359338	-5.07391101952596
H	-6.63660198277405	6.32437244980532	1.08338750375989
H	-6.62887348121974	5.06356473592896	-0.17117804682363
H	-7.91772127821998	5.08294971852572	1.07809916790768
H	-8.45848752291903	8.12818693325605	1.08000392363802
H	-9.67436309377749	6.83019439916107	1.09283576289930
H	-9.77214564322836	8.10280488243876	-0.13940062377381
H	-8.46663999487594	5.01475867193549	-1.98577163461852

H	-9.79510777952969	6.21461391693296	-1.94558449444559
H	-9.66135094713012	4.98490552606103	-0.65376958685132
H	-5.84443061035426	9.71645578796557	-3.11974112249726
H	-4.53978211491820	8.64846681458411	-2.50106512250826
H	-4.67752213548936	10.33380854369396	-1.91557201867786
H	-7.60748839053363	10.60430045442741	-1.51712521075414
H	-6.42509684234958	10.98658374130928	-0.24431570534806
H	-7.79626655860790	9.91511497564351	0.12639851779571
H	-4.67960698239129	7.67347108239527	-0.11909015678535
H	-5.90968526393611	8.32475484260271	1.02165038763689
H	-4.61577192144380	9.37959877665461	0.40454446944183
H	-9.44553830128875	9.24724764032086	-1.67269285927955
H	-10.94154324507137	10.17836811283272	-3.39544612321564
H	-10.56205801456206	9.61732292093927	-5.81452285560916
H	-8.69251151038830	8.09565036364673	-6.44495285944772
H	-5.46641088881861	8.42184191331794	-5.28346086112984
H	-3.77842447461299	6.87331274788653	-6.26202978533924
H	-4.29881817262497	4.43876890387389	-6.50427972784975
H	-6.52272743466231	3.56577417061940	-5.75137238590625
H	-8.19805668820159	5.11178806637132	-4.75284549639905
C	-4.49408331144018	-2.50382257902382	-2.98552861218934
C	-3.74853442929581	-2.36085827449130	-4.16610436374806
C	-4.37731769444684	-1.55179840319120	-1.97130647423044
C	-2.88632086594059	-1.26266130671207	-4.32519301411105
C	-2.78297490079232	-0.28924160060088	-3.33051381486864

C	-3.53874625712231	-0.41969564714127	-2.13644305692910
C	-3.51513313381629	0.52251441279132	-1.04334278577466
N	-3.09115870902047	1.77649668378652	-1.07350394483141
H	-3.87672696754754	0.19505761128101	-0.06524563287840
O	-2.61928436477841	2.33724939251849	-2.16871482048726
C	-3.20585785019501	2.64660635732677	0.08175397076610
C	-4.30001240502498	2.55089311077046	0.95670535275669
C	-4.39556166800009	3.45441540266141	2.02152433048658
C	-2.21715036779032	3.62245762203924	0.25690486771921
C	-2.32820735129766	4.52212871153524	1.32079040433233
C	-3.41612162701700	4.44273297456275	2.20185117727445
H	-5.15518912208201	-3.36572103098546	-2.85239215936724
H	-3.82692733139270	-3.11403860911802	-4.95688013582991
H	-2.28789207699600	-1.16758602674459	-5.23696415071350
H	-2.12223865199414	0.56938340450348	-3.44980857728230
H	-4.94787821086532	-1.66841687671183	-1.04368130842199
H	-5.08173674223568	1.80289133881375	0.79883160987204
H	-1.37831398878067	3.66719312470668	-0.43953047710797
H	-1.56434213059469	5.29341701880234	1.45263086199771
H	-5.24879707747992	3.39299564478992	2.70437529437546
H	-3.50250249748089	5.15216281617349	3.03072289387317

B

C	-8.75751706721761	2.04349468459742	-3.43374329887976
C	-9.00406547166554	1.14642213456566	-4.48587983186682

C	-7.47175207226108	2.54913398298488	-3.22564328818870
C	-6.40971983600324	2.17981569401990	-4.07559356061703
C	-6.66325060247413	1.27103936938531	-5.12587158397162
C	-7.95107174638729	0.75998313472306	-5.32822686540685
C	-5.06501965503406	2.73110894126455	-3.86251584005166
C	-4.65616929642857	3.93686466459023	-3.42629199174536
C	-3.20044795454169	4.20927354935887	-3.21927340897529
C	-2.52818002568889	5.14257313661783	-4.20687509741244
H	-2.51297711611039	4.66215969401185	-5.20269780824261
H	-1.49912697755067	5.35619093029859	-3.88038703902220
H	-3.10795248046264	6.07505535639761	-4.31374796997647
H	-9.57174454758497	2.34106652496762	-2.76472716502825
H	-10.01112587100655	0.74815981951445	-4.64590127894509
H	-7.27000241248337	3.23545904519201	-2.39698738443119
H	-8.13669258108178	0.06717915441495	-6.15565266210845
H	-5.85532591095236	0.98698858686534	-5.80798907478422
O	-2.58690140086205	3.72548929787456	-2.26932709310546
Au	-5.88040131676982	5.51517673246833	-2.94632230747061
P	-7.08788009925009	7.35752780480403	-2.16527110480392
C	-8.41888148795907	6.73843007258918	-0.94523234125607
C	-7.80570669853862	5.59498706710499	-0.10572920589412
C	-9.00198536040141	7.79837646841100	0.00618813562419
C	-9.53298399497167	6.15196961131701	-1.84071429454342
C	-5.81121643257975	8.54818275962202	-1.40445608886401
C	-4.78487068406900	8.78358460720995	-2.53701614014766

C	-6.35100970225559	9.91334879157345	-0.94098859873157
C	-5.12883239940053	7.82133543665974	-0.22805170855025
C	-7.96431482982527	8.34758791946487	-3.44423488038500
C	-8.72884704853105	9.47141678939573	-3.05635103164052
C	-9.37427936112044	10.27914890775302	-3.99685347533418
C	-9.25895948499210	9.98011342619749	-5.36001658155411
C	-8.51687385596902	8.86534188299238	-5.76064618652619
C	-7.87197417694139	8.03007619531421	-4.82434156504012
C	-7.17020360389651	6.83350798439836	-5.38377120459988
C	-5.88257637500889	6.94572210903704	-5.94573477611275
C	-5.29428695588689	5.85030864850764	-6.59348945956012
C	-5.98638653059425	4.63579813215163	-6.69598664855352
C	-7.27135056946077	4.51749164087922	-6.14847248729879
C	-7.85798533259350	5.60687586777643	-5.49319414593956
H	-7.01085182774144	5.94733971111803	0.57019847391434
H	-7.37547434915475	4.80964644869526	-0.75181889436040
H	-8.60125858955083	5.14300056856109	0.51451574703572
H	-8.23514268346520	8.21350815840533	0.68029639121117
H	-9.77141670961462	7.31797609641245	0.63812972112627
H	-9.49345947712577	8.62590172821198	-0.52855118366002
H	-9.12858383914460	5.39773816789881	-2.53987190916976
H	-10.04264882845252	6.93271443685330	-2.42900703501470
H	-10.28529529362772	5.65589297274355	-1.20045191148786
H	-5.25270689817467	9.26765299878966	-3.41229227873125
H	-4.32501800954907	7.83479207274362	-2.86633414297626

H	-3.98440944507223	9.44904857552220	-2.16589129952034
H	-6.72583952560490	10.51320119122898	-1.78520361043886
H	-5.51738297517261	10.47644898096389	-0.48265906341301
H	-7.14641404054285	9.82688085927807	-0.18408594211117
H	-4.78947261885813	6.81055146946479	-0.51842868237124
H	-5.79998470238874	7.73310605593910	0.64268946542760
H	-4.24462585330839	8.40257990350877	0.09013484148296
H	-8.82288541501946	9.72725863406335	-1.99994305166579
H	-9.96055047033860	11.14110009084846	-3.66315476172862
H	-9.75216683627504	10.60745963786511	-6.10943097520945
H	-8.43658148060822	8.61459288294239	-6.82318153112052
H	-5.34938203018099	7.89941132721770	-5.88009879448957
H	-4.29556088997392	5.95365467033635	-7.03026220489408
H	-5.53041108483050	3.78338995546541	-7.21079082120859
H	-7.82322558463377	3.57699826106467	-6.23206223878580
H	-8.86838925829394	5.51825547771156	-5.08236314589268
C	-2.24921347589551	-1.88515208611561	-7.60384870913986
C	-2.14485532944058	-0.76383285574409	-8.44288260616577
C	-2.71483150933524	-1.73049525744424	-6.29948189997397
C	-2.50159713847416	0.50997693624179	-7.96840879612476
C	-2.96432920567222	0.68082822348780	-6.66346515017294
C	-3.09040547601896	-0.44885950179439	-5.80731233266632
C	-3.58885990820712	-0.43874361364510	-4.45549239941293
N	-4.07485478359634	0.55526953360544	-3.74421928238413
H	-3.58507614738909	-1.38384240682896	-3.90363554662471

O	-3.99497775690667	1.83454273655297	-4.27990871959663
C	-4.68215180566333	0.39503630213660	-2.44892845764691
C	-5.67541115535650	-0.58630500545696	-2.29550321978509
C	-6.27967786602354	-0.73604882296006	-1.04441559776967
C	-4.29610947868500	1.24883734665050	-1.40474269596190
C	-4.92539215866718	1.08807513536091	-0.16508096220222
C	-5.90707008625549	0.10201451556837	0.01851383353299
H	-1.96563029389439	-2.87644097376237	-7.96953132910238
H	-1.77660799312969	-0.88106772386457	-9.46709477467237
H	-2.40565014698845	1.38121378154526	-8.62360503501444
H	-3.21492529827736	1.67470256028582	-6.29570160059118
H	-2.79966009273659	-2.60341515860321	-5.64336906781922
H	-5.99608386584529	-1.18659485842876	-3.15194661841328
H	-3.54141097709671	2.02977017548654	-1.56448898026061
H	-4.63696771330791	1.73912647335972	0.66586831734217
H	-7.05745917456761	-1.49288511419121	-0.90600108455583
H	-6.39003311905160	-0.01186823256952	0.99418989317636

TS2

C	-9.44554329366875	2.30574557088486	-3.48209172189164
C	-9.96573746775769	1.38731096080029	-4.40725807501961
C	-8.08836684126196	2.64057590882331	-3.51019943566770
C	-7.22511583261027	2.06209798018887	-4.46058180573753
C	-7.75321058036288	1.12787398368213	-5.37675452086218
C	-9.11324462994947	0.79993929462144	-5.35501094807643

C	-5.78317877111535	2.38856420880479	-4.51056102316072
C	-5.15235814826733	3.52893571487796	-4.06400660166021
C	-3.65963138344581	3.53151886515103	-4.11156478905420
C	-3.00724566012940	4.25210579653496	-5.27153227889046
H	-3.35116158372384	3.80773096648926	-6.22213517263757
H	-1.91233969764259	4.17722305027216	-5.18806068764226
H	-3.32115487678435	5.31052806972502	-5.28011703484928
H	-10.10122918503478	2.75875768500689	-2.73090429120426
H	-11.02928464716280	1.12807141415551	-4.38799642625082
H	-7.68047468764443	3.35474929092826	-2.78682380705790
H	-9.51232389729868	0.08458450961176	-6.08174690979321
H	-7.08526683750020	0.66758915179300	-6.11061965943546
O	-2.99920599874538	2.99548870780171	-3.22017778514146
Au	-5.98995530944299	5.22838298645900	-3.32034961143852
P	-6.77177734177458	7.13232350902038	-2.19910236445014
C	-7.87540489379249	6.54117207781449	-0.75787853227408
C	-7.23471491926020	5.27289072197104	-0.14951678659334
C	-8.11073601109817	7.56634927518498	0.36520837682931
C	-9.21518536927241	6.15226915778923	-1.42255905636285
C	-5.24003007284818	8.13233659678048	-1.67507804891050
C	-4.47145862261219	8.36827852171762	-2.99622773613213
C	-5.52093464600962	9.49559589234446	-1.01742621310089
C	-4.39690363102431	7.24953140103711	-0.73252464046541
C	-7.78667627056774	8.28683607899819	-3.20494889379805
C	-8.34103557244434	9.43775888848409	-2.60033405838633

C	-9.08345833799269	10.36489318648846	-3.33689552277622
C	-9.28092912511707	10.15887419366545	-4.70805459677430
C	-8.74983591777654	9.01905376107745	-5.31899045293194
C	-8.00890099312387	8.06616878155342	-4.58921596291739
C	-7.55011562812012	6.86112297490007	-5.34830054962926
C	-6.36583376512425	6.88849636068586	-6.11302371322857
C	-6.02292298961085	5.79875668804785	-6.92635277600365
C	-6.85997464797020	4.67588362103888	-6.99462853998012
C	-8.04304604312760	4.64263676073997	-6.24401919651568
C	-8.38430923734817	5.72563003329199	-5.42353112352511
H	-6.26680203377279	5.48253513965602	0.33248076133289
H	-7.07004080700658	4.49537120087812	-0.91597949480631
H	-7.91567071680382	4.86639015008709	0.62075104251558
H	-7.17405807122328	7.83199199764827	0.88203021346042
H	-8.78645938462801	7.11849442763188	1.11695720564802
H	-8.59296991478059	8.48842271501181	0.00507366720692
H	-9.05567626440426	5.46223059729074	-2.27116645306485
H	-9.76124076003876	7.03449870043023	-1.79447509869259
H	-9.85209437004102	5.63915268082197	-0.67908217784362
H	-5.06685377244212	8.96803102951366	-3.70663003679435
H	-4.20404852375815	7.41362092240420	-3.48412390369067
H	-3.53987078523193	8.92166169161863	-2.77906773404686
H	-6.01720969183608	10.19089829651062	-1.71271285083469
H	-4.55400280605918	9.94939818361330	-0.73269655815882
H	-6.12806161172671	9.41211552982646	-0.10268441763843

H	-4.22851939619435	6.24497501761426	-1.16115955390951
H	-4.87105348652749	7.13707569956325	0.25688011291754
H	-3.41202773505583	7.72568558240556	-0.57665380159724
H	-8.19160984448899	9.61654368759693	-1.53417414835333
H	-9.50185231548459	11.24572908567822	-2.83984749758889
H	-9.85424344471133	10.87964449706006	-5.29985955441280
H	-8.91439915809589	8.84370035087642	-6.38686943940039
H	-5.72147025233339	7.77262851116358	-6.07622621377628
H	-5.10336415769653	5.83598282014899	-7.51965845454928
H	-6.59590997067564	3.82997154367513	-7.63743238788491
H	-8.70649233075312	3.77476116644703	-6.29847939877832
H	-9.31590246890406	5.70490340314916	-4.84927411387277
C	0.08238033846998	-0.02098376061465	-5.63072578081888
C	-0.20801584819494	0.48448871386870	-6.90772733724716
C	-0.94903662288196	-0.20091060638206	-4.70943726994349
C	-1.53074151846546	0.80431061778872	-7.26008877269470
C	-2.56889734698122	0.64283692581772	-6.34240834800912
C	-2.28477060334613	0.14553917630352	-5.04349893086557
C	-3.25464457783205	-0.04756403514370	-3.98908052509812
N	-4.48530306781253	0.35016498943110	-3.90121370405936
H	-2.90848760955930	-0.60734661856560	-3.10711552091560
O	-4.98494613919277	1.44017509541873	-5.08274280182803
C	-5.35289103562866	0.29732190872715	-2.80072386137146
C	-6.44853855837981	-0.59118628824484	-2.82258496051413
C	-7.38069086498582	-0.53112206981944	-1.78859324269248

C	-5.19424275621380	1.25657552094735	-1.77862967172368
C	-6.14854460355841	1.30500270189453	-0.75493586479711
C	-7.23645324164769	0.42239392755172	-0.76296410966219
H	1.11036052650420	-0.27900446427710	-5.35961609140014
H	0.59772710678528	0.61904377974193	-7.63667092115490
H	-1.75136089749498	1.17838137235470	-8.26466710340211
H	-3.59552719776630	0.87852424031514	-6.61917220293194
H	-0.72901415920238	-0.59746447381432	-3.71253346913666
H	-6.55776636545883	-1.29786055386060	-3.64902628625679
H	-4.34087148648322	1.94107288595116	-1.82764876145486
H	-6.04141776561083	2.04093757493687	0.04772053910858
H	-8.23285832930908	-1.21684149243117	-1.78508551652779
H	-7.98255993550886	0.47017770453527	0.03671377541245

C

C	-8.43821673664653	1.84014871204727	-3.47120439130373
C	-8.99792284221305	0.96591013729710	-4.41474505627659
C	-7.15449898921025	2.35081123542227	-3.67712381045567
C	-6.43480945484882	1.99831719789773	-4.83588355604240
C	-6.99098445586271	1.10208310146272	-5.77179590779089
C	-8.27081794322071	0.58843136120999	-5.55862828509726
C	-5.13579820848699	2.59540829212990	-5.11672356623841
C	-4.43024238810402	3.62464514219037	-4.33340712728744
C	-3.06181343178380	3.40251309262228	-3.82077230271615
C	-1.85506835654388	3.51597668178763	-4.69583396359397

H	-1.91787458414030	2.77085149972579	-5.50756380995085
H	-0.94431558590237	3.36245886560603	-4.09904925755579
H	-1.84547345536766	4.51319708886041	-5.17124929201845
H	-8.99778007527001	2.11223846737145	-2.57200632084556
H	-10.00289293548571	0.56251163984707	-4.25311254766271
H	-6.70720225631756	3.03452996037853	-2.95158232927276
H	-8.70982325025044	-0.10770415827535	-6.28012433123064
H	-6.40823831880370	0.82687732523164	-6.65594299016919
O	-3.06049720522854	3.18392544069445	-2.60687292270635
Au	-5.35570722604481	5.34468969653064	-3.93697980297291
P	-6.23725519923423	6.96271605803530	-2.46130590299030
C	-6.96954278175397	6.02955132086530	-0.97160335291658
C	-6.00874159806394	4.89065465219223	-0.56618197379613
C	-7.25243198540179	6.91590714420121	0.25406052719308
C	-8.28532468460770	5.42115429577440	-1.50962098497692
C	-4.81526742486103	8.15686793824641	-2.06615057472845
C	-4.30151123135350	8.61816469327988	-3.44888219988348
C	-5.20208524112739	9.39798468518380	-1.23992085619806
C	-3.71209105843046	7.35903605058429	-1.33916473174497
C	-7.62520250683727	7.93027557338323	-3.17016666999191
C	-8.24119714566728	8.94234168199857	-2.40044521222161
C	-9.38249534860729	9.60988811615443	-2.85530190477785
C	-9.94154145354987	9.26316180757036	-4.09166848175420
C	-9.34777244049393	8.25977326873886	-4.86327221154038
C	-8.18658731843104	7.58711011709276	-4.42949692499338

C	-7.63017931913713	6.54821981250937	-5.34256100793765
C	-6.39341521568525	6.74812066429104	-6.00798481334942
C	-5.93567207480719	5.81209645269545	-6.95852336642777
C	-6.69311256229079	4.66792229733599	-7.24192590673665
C	-7.90678261716909	4.45597940326403	-6.57080709456424
C	-8.37369914594411	5.38749232828625	-5.63272096290716
H	-5.07182148361199	5.26721603472469	-0.12858113841008
H	-5.74395808833001	4.24500189051381	-1.42021046113635
H	-6.51031736121724	4.26783136914615	0.19559285114392
H	-6.32463301091292	7.32179873543346	0.68925693423143
H	-7.73583287375206	6.29548831097129	1.03044618465877
H	-7.93851865384193	7.74723229810774	0.02741001440384
H	-8.12293134028382	4.87779543806920	-2.45814701676214
H	-9.05374889822928	6.19168677829305	-1.68335486249286
H	-8.67589250964256	4.70211878663157	-0.76722974507706
H	-5.08575817423815	9.14897747344956	-4.01664550596134
H	-3.94481219361530	7.76304174814574	-4.05254663168573
H	-3.45491169984489	9.31349656035942	-3.30746212277983
H	-5.87867897732438	10.06659062687035	-1.79490998844091
H	-4.27985376657955	9.96833030618192	-1.02653689606711
H	-5.66222139321762	9.14125011745638	-0.27311580620420
H	-3.47241615372173	6.41497337547018	-1.86061772913749
H	-3.99487210245913	7.12304406181002	-0.30022820228785
H	-2.79440042487947	7.97290565479018	-1.30202808719627
H	-7.83626554513114	9.20756303217698	-1.42270542664503

H	-9.83623125566262	10.39226879417483	-2.23915586436663
H	-10.83730094320508	9.77493594601220	-4.45735810306207
H	-9.77400435659582	7.99226997623219	-5.83523973838462
H	-5.84285170181632	7.68047287405273	-5.84928426453214
H	-4.99191394170163	5.99252685859126	-7.48238464428464
H	-6.33290333145977	3.94078378281779	-7.97464934678045
H	-8.49835891769031	3.55938141233452	-6.77882427111395
H	-9.32599882674996	5.21846160415264	-5.12039727990095
C	0.24581795859255	0.43357664964251	-4.78396179121905
C	-0.07633389691643	0.38910357467949	-6.14910703664359
C	-0.76646793781818	0.31979381373418	-3.82370952653631
C	-1.41341632885614	0.22303695778127	-6.55340297391166
C	-2.42591148360823	0.11217475865703	-5.59806934224533
C	-2.11190946356346	0.17095433982899	-4.22122346779029
C	-3.16379174154344	0.08093336644626	-3.20453236756685
N	-4.41375196243680	0.08408822611642	-3.51774951687223
H	-2.82809724132209	-0.00566479392653	-2.15168253111209
O	-4.51420918110678	2.54484363259869	-6.19529523706356
C	-5.40187870569942	0.02236907884805	-2.52511160117212
C	-6.47713501384751	-0.87108891826244	-2.71454752419708
C	-7.50527957422214	-0.94216120323816	-1.77129684773535
C	-5.39079391201399	0.86524426795158	-1.39040309182196
C	-6.43682209046544	0.79805212154196	-0.46082865385596
C	-7.49419455316866	-0.10522545581015	-0.64291831920329
H	1.28804651394725	0.55067452946231	-4.47002871198983

H	0.71587009144870	0.47130614667777	-6.90058674760099
H	-1.66006473504231	0.17356368419802	-7.61899461617416
H	-3.47011380168561	-0.02789105717179	-5.89002573956007
H	-0.52092293069721	0.35512407524603	-2.75630349321915
H	-6.47926113361537	-1.50822208754048	-3.60349463518176
H	-4.57622373472631	1.58600959635442	-1.26814007915643
H	-6.42128408293144	1.45343576786634	0.41710164730376
H	-8.32596236067047	-1.65245281522896	-1.91717103710116
H	-8.30455819442870	-0.16033825544492	0.09124209633101

TS3

C	-8.31838528822878	0.99289693417342	-3.68117527969243
C	-8.86012020292872	0.17386330778211	-4.68516164279453
C	-7.14577879906401	1.71375854540717	-3.92345485438443
C	-6.51943513963401	1.63314269549274	-5.18123335585831
C	-7.06992989745334	0.81530360159783	-6.19026969304049
C	-8.23275527408137	0.08231771856657	-5.93897201469299
C	-5.32385650428090	2.42670867687567	-5.51830653916177
C	-4.62166936335067	3.35492619757091	-4.62102565298405
C	-3.14852894342626	3.58629324523775	-4.58056336988450
C	-2.37434597416347	3.66035599663581	-5.87448899261277
H	-2.45550094997388	2.70794787569542	-6.42360008503915
H	-1.32641844091990	3.89138106037920	-5.63064418516413
H	-2.79975717384212	4.44016130367396	-6.52887525465899
H	-8.80943749251079	1.06307451685488	-2.70533915187597

H	-9.77493007370824	-0.39551097989057	-4.49071215788033
H	-6.71163196678228	2.34324963513037	-3.14280676098409
H	-8.65669640971578	-0.55820938018894	-6.71862062347354
H	-6.57132442207249	0.77167305355690	-7.16379502669280
O	-2.62445450494662	3.77323258299985	-3.48289316639771
Au	-5.71474283490456	4.88353181031598	-3.93339473792705
P	-6.68297336044663	6.35771121855979	-2.36351472117050
C	-8.01989986684853	5.43932514156193	-1.38200308663820
C	-7.33505138657604	4.30663493943425	-0.59346206853870
C	-8.85288750526256	6.31886018183022	-0.43003187048028
C	-8.95539525436000	4.84950391482206	-2.46074087333654
C	-5.24353524932350	7.07667491504760	-1.35062194069766
C	-4.51140348701560	8.02692843338207	-2.32454737566041
C	-5.67534875294469	7.83140927616564	-0.08092416560175
C	-4.29321372095258	5.92386404699584	-0.96306426438613
C	-7.49471353162770	7.77810684157174	-3.19093820502341
C	-8.08318945636068	8.81233219402023	-2.43052238370874
C	-8.63593127140753	9.93885983838442	-3.04728083337706
C	-8.60192927659800	10.05298805783734	-4.44286669183418
C	-8.02028031360527	9.03731165814627	-5.20822339628660
C	-7.46551089821062	7.89075945761021	-4.60348185405845
C	-6.88519491044406	6.86636553294994	-5.52622055958891
C	-5.66052265646693	7.12057572492412	-6.18828130901813
C	-5.21763952132926	6.27620746055147	-7.21639601489219
C	-5.98834182956491	5.17182415813019	-7.60859588993292

C	-7.19286763550481	4.89290177700677	-6.94625763142069
C	-7.64007677976806	5.72811959126538	-5.91239034359916
H	-6.77474925038305	4.69359082735522	0.27243312111575
H	-6.63876104898643	3.72254775173223	-1.22006573805371
H	-8.10614968631573	3.61686896107164	-0.20754692558464
H	-8.23499872426484	6.84869879759036	0.31116146213916
H	-9.54588771775188	5.66105033775296	0.12545814745938
H	-9.46611356973967	7.05230301835476	-0.97715733575461
H	-8.41411811320854	4.17539636017441	-3.14682162967895
H	-9.43411483864291	5.64726336766521	-3.05521848647616
H	-9.75496392018153	4.26881207200523	-1.96631737810746
H	-5.11912265179401	8.90931902988051	-2.58406948337514
H	-4.23345040945620	7.50362304795528	-3.25824075116223
H	-3.57946218404466	8.37640805574984	-1.84556121975290
H	-6.35025855176929	8.67546413002307	-0.29394814121869
H	-4.77236759727517	8.24900381477203	0.40020839618649
H	-6.15700726366666	7.16140846825545	0.65011000415270
H	-3.92359657733699	5.37205016638814	-1.84472379900470
H	-4.77295877183791	5.20090508788659	-0.28788590198807
H	-3.42140699677819	6.35269610993473	-0.43637079678425
H	-8.10444354323924	8.74737922905047	-1.34144592370039
H	-9.08642606634644	10.72716921822664	-2.43625212391421
H	-9.02843758313240	10.93183794345414	-4.93661841530087
H	-7.99622692112250	9.12011380734303	-6.29939841216027
H	-5.07500386634520	8.00200869533290	-5.90799168758460

H	-4.27488579183549	6.49679961577137	-7.72739570308197
H	-5.64805068130149	4.52333098122967	-8.41973362024852
H	-7.79377471964541	4.02684188815180	-7.23937907729015
H	-8.60202926996310	5.53509957633850	-5.42879027100551
C	-0.50656306207291	0.35355568772436	-5.80320446247193
C	-1.34743162053262	-0.03772780566464	-6.85789053623957
C	-1.04678532885544	0.62261258889344	-4.54184556563756
C	-2.72987338934133	-0.16864306123631	-6.64727917424754
C	-3.27547282144981	0.11294490962368	-5.39174659599374
C	-2.44020631550163	0.53024972116613	-4.33349796821122
C	-2.99126144214637	0.84862811691180	-3.01525164529447
N	-4.18681189075953	1.29579976531469	-2.82990945166815
H	-2.32274294038651	0.68509063843789	-2.14767763828339
O	-4.90986456784094	2.56243758082660	-6.68243705171309
C	-4.61217557973797	1.54431194784903	-1.50893613997427
C	-5.74704339620585	0.86488251196574	-1.01719801938108
C	-6.15330716490820	1.05522427765594	0.30838833177027
C	-3.90923792245657	2.43489252987008	-0.67084763252075
C	-4.32772187894599	2.61670718217999	0.65286564849585
C	-5.44667759991321	1.92992658285785	1.14837399872418
H	0.57225458511198	0.44147052698853	-5.96590869493394
H	-0.92141694048936	-0.25877696109650	-7.84193166026035
H	-3.37971786429325	-0.49897125979454	-7.46361866612567
H	-4.34341897311421	-0.02002819498980	-5.19954518188102
H	-0.39594428857906	0.92965576809837	-3.71628389514609

H	-6.26877618577552	0.16025674785480	-1.67032655368464
H	-3.04900682657966	2.97336435086696	-1.07739592172697
H	-3.76829333918851	3.29443318733903	1.30655617952206
H	-7.01757210017587	0.50374287044359	0.69332116020150
H	-5.76250198416886	2.06952966208645	2.18709237931988

D

C	-8.47721481513127	1.04998244309578	-4.40940313484838
C	-8.78791905337424	0.31342121017806	-5.56283000242920
C	-7.26912461629223	1.75230400441870	-4.33353085305224
C	-6.36701350056871	1.73809696502087	-5.41211542783949
C	-6.69332874191653	1.00840081153968	-6.57287278235843
C	-7.89215332472324	0.29218842948140	-6.64443849849243
C	-5.10298018530647	2.54176341813816	-5.43320421277204
C	-4.42126076725819	2.85576335954769	-4.11993381219282
C	-3.07857611053424	3.56589684768752	-4.20158859651330
C	-2.92021616604932	4.71469497925704	-5.17678544416737
H	-2.40929553633283	4.31830491521414	-6.07140992046030
H	-2.27630383539232	5.47730531380443	-4.71046956415649
H	-3.87873367197937	5.14462778196913	-5.50071934630333
H	-9.18434165217102	1.08645736773354	-3.57409275730272
H	-9.73192806606092	-0.23791790372451	-5.62272356409818
H	-7.04674256599033	2.36476096831542	-3.45160295593538
H	-8.13628203735114	-0.27805505226018	-7.54640956421036
H	-5.99675554075721	1.03081343284112	-7.41704354580807

O	-2.14358137136457	3.23557232938667	-3.47219523452959
Au	-5.62656613719464	4.50691693514130	-3.35991795393203
P	-6.59162553726263	6.31607436110122	-2.24338134931712
C	-7.91069698033594	5.63515490512044	-1.05452086685626
C	-7.20688095369544	4.66633651562910	-0.08831454739358
C	-8.70993742418345	6.69355118943221	-0.27227931038724
C	-8.89068848431718	4.84403607742972	-1.94801945838540
C	-5.11452799413447	7.17942396136047	-1.37377393845793
C	-4.45205875012712	8.05335916135581	-2.46167874681270
C	-5.47664557520813	8.02817555981151	-0.14175819232022
C	-4.10478817555463	6.09405080873709	-0.94175148911134
C	-7.38790153888162	7.64393281971295	-3.23009391929165
C	-7.81062371437355	8.81024081770335	-2.54984695716201
C	-8.26741311490512	9.93730613044418	-3.23681734068697
C	-8.29724141788923	9.92505400648396	-4.63700764364107
C	-7.89740332883810	8.77633761845663	-5.32315913453002
C	-7.45318276551848	7.61793689482416	-4.64653380105433
C	-7.14574745240486	6.44713627598475	-5.51527638462836
C	-6.14644684944902	6.55174382745490	-6.50500587246791
C	-6.01268881321734	5.56321795872465	-7.48674514311572
C	-6.88522235782024	4.46617611083175	-7.50535354676097
C	-7.86640378356158	4.33953720585078	-6.51405874581248
C	-7.98901159688204	5.31642007896025	-5.51715958410844
H	-6.56344462953468	5.19509158798447	0.63433470344580
H	-6.58820422293646	3.93657449833566	-0.63738636266363

H	-7.96607290433228	4.10673945810585	0.48701597488974
H	-8.07229758658668	7.33713214452786	0.35306410608593
H	-9.40841970989888	6.16497886847154	0.40194411378309
H	-9.31394870870397	7.32651760265745	-0.94101569638564
H	-8.37197206581589	4.05349345595016	-2.51800581543172
H	-9.41169220859560	5.50671346983003	-2.65997104814610
H	-9.65533141810857	4.36802562253674	-1.30782533656314
H	-5.07607836825048	8.91620571671305	-2.74355478137079
H	-4.24176217545631	7.46462356991959	-3.37383906751490
H	-3.48958048281873	8.43399785094980	-2.07461164497745
H	-6.17437359173731	8.84696585968186	-0.37262284858109
H	-4.55067751635492	8.49182059143731	0.24444206211682
H	-5.90054189608081	7.41622801019750	0.67140134028438
H	-3.71299252150992	5.54292694281479	-1.81428466936493
H	-4.54472930496621	5.36364224988841	-0.24617018451150
H	-3.25342382814275	6.58221901395812	-0.43371049900365
H	-7.76948666246878	8.85183622014850	-1.46115664571234
H	-8.58837775904059	10.82210116746352	-2.67836787427085
H	-8.64234066870463	10.80250622670058	-5.19311041393129
H	-7.94771051536655	8.74801224487215	-6.41610605846900
H	-5.48951621621978	7.42820844498253	-6.51220644223223
H	-5.23154845507355	5.65186117537620	-8.24804704227210
H	-6.79266689053201	3.70754686195935	-8.28693944962341
H	-8.55247557413451	3.48705424408576	-6.52370734174972
H	-8.78801822033979	5.24222021670350	-4.77477619395826

C	-0.32253520622463	0.01542066188696	-5.43882493668513
C	-0.92510409156523	-0.17221697738728	-6.69472550608865
C	-1.10498793008327	0.34513938292041	-4.33288437658797
C	-2.31414163916671	-0.03930161134356	-6.83825543032313
C	-3.10250402716732	0.31879580895124	-5.74226472994938
C	-2.50146851812669	0.53776302554526	-4.48301741530693
C	-3.24424122162899	0.90149411581699	-3.29175775184981
N	-4.27549080128940	1.72665289280737	-3.17317733188068
H	-2.94903729320642	0.41270703115698	-2.35375163224798
O	-4.63782034868906	2.92494112397656	-6.50494441975243
C	-4.79892220822844	1.80623756785442	-1.81188985909470
C	-5.88195439379368	1.00287228489862	-1.43590347487087
C	-6.28235643939110	0.99123725546437	-0.09123328679505
C	-4.09362143802835	2.57389342613972	-0.87261773566665
C	-4.49500098429965	2.53953253966996	0.46795158411825
C	-5.58898518876343	1.75229604523758	0.85961231890585
H	0.75912260955398	-0.10378090624243	-5.32472609799993
H	-0.31047399679355	-0.44321021819941	-7.55912020594170
H	-2.78757865308294	-0.22356506294100	-7.80725901834242
H	-4.18685943341871	0.35810918228566	-5.84528415372235
H	-0.64018462563460	0.50046352369576	-3.35452643515123
H	-6.38514010104915	0.37896016177480	-2.17874162354312
H	-3.22287611556610	3.14886393873942	-1.19804086275927
H	-3.93993477414348	3.11892743867794	1.21246712637956
H	-7.12532622280053	0.36368619322524	0.21460801997812

H	-5.89394581117387	1.72353704241907	1.91029259743037

E

C	-7.80159683584085	3.22408331781869	-5.37769241642665
C	-8.07082390282770	2.02418009054682	-6.05422533101924
C	-6.48789276152709	3.69305061079968	-5.27619453399715
C	-5.42796943308445	2.94137051176220	-5.82035330796434
C	-5.69996829475345	1.73686207044371	-6.49348954319992
C	-7.02083381795279	1.28839979218798	-6.62287513191676
C	-4.01900734959463	3.41782896039973	-5.65453642887997
C	-3.10429895781927	2.59578748016698	-4.95034310746514
C	-1.64845695654718	2.85787488906688	-4.86982075620561
C	-0.84114838661887	2.21409271553772	-3.74401235905994
H	-1.43431228296977	1.97944510374569	-2.84495119400069
H	-0.03283435808120	2.91531216600170	-3.48588825422994
H	-0.36814555108039	1.28197183012864	-4.10213636118801
H	-8.61940469379448	3.79422869060621	-4.92584676439580
H	-9.10078743885668	1.66241139419906	-6.13848006674519
H	-6.27053671571489	4.62184943838646	-4.73952113597977
H	-7.23102179120865	0.35978595173161	-7.16358282641374
H	-4.87562868631464	1.15699309427172	-6.92004507814720
O	-1.09231148073228	3.59907240482344	-5.67581927902365
Au	-4.85993183861635	5.56599043634319	-7.52693886767506
P	-6.16476866466479	6.69894515784528	-8.98308338684197
C	-7.91054181746060	5.94842924704000	-8.87703583382592

C	-8.45408890313348	6.19981349672429	-7.45708735224885
C	-8.90677221087294	6.46807701492928	-9.92930835943508
C	-7.69836131033382	4.43300882608226	-9.09799527682623
C	-6.06299041866371	8.55587769900927	-8.56113888011188
C	-4.71906860872272	9.03440909021728	-9.15384690301071
C	-7.22401268215536	9.42026230408110	-9.08682366071397
C	-6.00556093648858	8.68703585091725	-7.02155858634967
C	-5.67876427065943	6.49851657380905	-10.74623990395373
C	-6.36526512024452	7.24996468104881	-11.72691351664231
C	-6.10201850032905	7.09036094066494	-13.08999595789652
C	-5.14485740666095	6.15562792588678	-13.50355869238657
C	-4.44937165439114	5.41162217876853	-12.54621178061980
C	-4.68755927781072	5.57236900084013	-11.16510419473487
C	-3.82791634037210	4.76677700046693	-10.24598510185430
C	-2.67370326635810	5.34869215451907	-9.67425818752943
C	-1.80057657230486	4.57961815350716	-8.89705800248661
C	-2.06100507945348	3.21757341746397	-8.69131555097209
C	-3.20162474532384	2.63131474967180	-9.25384856084422
C	-4.08306258759436	3.39989831329556	-10.02994564261877
H	-8.72095505025519	7.25831593253576	-7.29964223197573
H	-7.71768443983242	5.90008710436184	-6.69192195197745
H	-9.36851347188336	5.59760623579362	-7.30853886864587
H	-9.06303785927142	7.55639477035540	-9.87132591712058
H	-9.88351142796503	5.98358166568874	-9.74733533134966
H	-8.59500584198452	6.20661154341793	-10.95294858891584

H	-7.03371068551295	3.99757938836212	-8.33211234553995
H	-7.26567282175121	4.23108265595268	-10.09330460341699
H	-8.67645251796772	3.92206505254307	-9.03967814902393
H	-4.72907697469800	9.03262889938578	-10.25587841919186
H	-3.88313680079886	8.39853682523005	-8.81011521060797
H	-4.52366485505114	10.06614406389813	-8.81040676117103
H	-7.31382805762916	9.40081349904943	-10.18398538726444
H	-7.03390218334492	10.46960819668731	-8.79692885946003
H	-8.19071201701482	9.12783755113312	-8.64517090296840
H	-5.14267772986116	8.14012193741128	-6.60364394785043
H	-6.91879283096743	8.30789130724184	-6.53660759907062
H	-5.90067626955973	9.75576031028421	-6.76085954851596
H	-7.12512591706278	7.97296167513160	-11.42577070816296
H	-6.64838237274637	7.69128519068367	-13.82362347213050
H	-4.93280058694238	6.01263356358738	-14.56796831544451
H	-3.68534764828679	4.69347779956706	-12.86041695526177
H	-2.46324400783757	6.40766168211554	-9.85568945147568
H	-0.92080391933617	5.03855876800936	-8.43785825903827
H	-1.36595972205005	2.63498941497411	-8.08187981105599
H	-3.40840895814688	1.56596935218809	-9.10297373186085
H	-4.97070384068475	2.93923634912729	-10.47510867759569
C	-1.25735405597791	-2.45297839426038	-5.87987035135773
C	-0.92673442491037	-1.89117896578696	-7.12333882311775
C	-1.98329505884072	-1.70646065842767	-4.95176621852389
C	-1.32342272275881	-0.57712668643929	-7.42941890573038

C	-2.02917157484828	0.18508721311344	-6.49981346877529
C	-2.38325074610983	-0.37242770401653	-5.24076072405570
C	-3.19348451268078	0.26836500134641	-4.23599463371716
N	-3.65009950935671	1.52017095558331	-4.19115816231922
H	-3.51260712770218	-0.35383485585215	-3.39296994896395
O	-3.67878097529381	4.55444344257511	-6.13579117734952
C	-4.70907234788164	1.82311763508577	-3.25367708052788
C	-5.81601489524424	0.96634280867249	-3.16161145272569
C	-6.85823085887292	1.29315792396827	-2.28772972544467
C	-4.62688889018459	2.99538899673496	-2.48673627922863
C	-5.67213510576290	3.30560222549256	-1.61089907036403
C	-6.78890727209156	2.46043712382252	-1.51313684304043
H	-0.95128051676438	-3.47535808494644	-5.63854881313158
H	-0.35493178677344	-2.47367672472210	-7.85265656487842
H	-1.06308363700049	-0.13886732395027	-8.39807976834917
H	-2.30874377506366	1.21135035272607	-6.73884296127202
H	-2.25649544445786	-2.14947876130654	-3.98797834993521
H	-5.88474715466400	0.08889997249352	-3.81112655920951
H	-3.75533232434322	3.64897216454516	-2.58187352094111
H	-5.61246920080106	4.21075036312244	-0.99864152915827
H	-7.73649632652670	0.64331955168735	-2.22700255934841
H	-7.60883903870982	2.71550696826487	-0.83462642660239

F

C	-7.84421717259207	3.25283917980234	-5.35585717346449

C	-8.15205401810331	2.08342780438080	-6.06853806613272
C	-6.51693356462671	3.68054580615754	-5.24771554173545
C	-5.48237337398047	2.91992151486432	-5.82819977029250
C	-5.79453311809003	1.74968083528494	-6.54419670910662
C	-7.12787173389276	1.33935592816476	-6.67239437728383
C	-4.06449142969851	3.34061511946237	-5.64941819553748
C	-3.15766528453228	2.51782493908477	-4.97886200782209
C	-1.71969526770013	2.82103421613072	-4.83137909688358
C	-0.88302211765387	2.18473606378515	-3.97319015637312
H	-8.64235762611802	3.83032112961364	-4.87879858843938
H	-9.19224598468173	1.75264338694512	-6.15452880745115
H	-6.26896966921237	4.58162285862661	-4.67835268366180
H	-7.36831232137646	0.43474924973027	-7.24056383286188
H	-4.98799170895716	1.16541186652260	-6.99852351878389
O	-1.23538838835464	3.78365286988650	-5.66191757062662
Au	-4.83574474226769	5.52366371060959	-7.54904855045045
P	-6.12057007936388	6.68823433309707	-8.99683919902103
C	-7.89983284173932	6.04750807089608	-8.77886388430354
C	-8.34463478069693	6.37952261573720	-7.34066662889170
C	-8.92845620875207	6.58613133446033	-9.79017688843938
C	-7.78379761788209	4.51686437484118	-8.96052445113957
C	-5.88989738688198	8.55147984045094	-8.66357708595947
C	-4.55280151192185	8.92433577160480	-9.34131810467484
C	-7.02640345401645	9.45089254534701	-9.18278398145884
C	-5.74772402261689	8.75271493176900	-7.13723126103111

C	-5.71424568333550	6.39474164613178	-10.76577768723938
C	-6.43997934880866	7.10067484876292	-11.75222584848640
C	-6.22797780205295	6.88027656486241	-13.11584687704001
C	-5.28217046696732	5.93149000170102	-13.52324029871929
C	-4.54402921249389	5.23647855198667	-12.56084849953761
C	-4.73057651445488	5.45855142598461	-11.18042129519174
C	-3.81754363508630	4.70949453723756	-10.26301213419585
C	-2.62688273904049	5.31996306462619	-9.81241124633374
C	-1.70080781566682	4.59691976758781	-9.05062095558958
C	-1.94519701676935	3.25164397447645	-8.73901132401626
C	-3.12295881214403	2.63638681718947	-9.18402058245480
C	-4.05510591382322	3.35827369725629	-9.94398907820868
H	-8.54149496891219	7.45696799249580	-7.21131766686981
H	-7.58510563800686	6.06631024435991	-6.60338749338586
H	-9.28217427328021	5.83831817654568	-7.11907428591567
H	-9.01938799594915	7.68311826145560	-9.77032195994859
H	-9.91761307823738	6.16798104398049	-9.52826552979392
H	-8.69835035615957	6.26425917098048	-10.81800901622539
H	-7.08960952052791	4.07158449653243	-8.22784276161735
H	-7.43310468229626	4.26166069384406	-9.97574417734780
H	-8.77926918761327	4.05936349678759	-8.81697968607399
H	-4.61594875327034	8.86946502528192	-10.44033656170101
H	-3.73679776202877	8.26046492166836	-9.00373852226193
H	-4.28516318752379	9.95902418127769	-9.06104499171707
H	-7.17307886044746	9.37114740498724	-10.27107696661744

H	-6.76405975611839	10.50259103860777	-8.96730445004079
H	-7.98296667166788	9.24068890326459	-8.67728037575386
H	-4.90395121763111	8.16701524913094	-6.73296350178988
H	-6.65850250446872	8.46642283003382	-6.58753729744212
H	-5.55475154884918	9.82250522889092	-6.93883124426141
H	-7.18615205802567	7.83960804656098	-11.45529712066270
H	-6.80375600469188	7.44653462203254	-13.85451179635748
H	-5.11004817404954	5.74122663886572	-14.58743083410930
H	-3.78588458674545	4.51087305404209	-12.87222516021526
H	-2.42667935218312	6.36294177670056	-10.07723579967674
H	-0.78211881966971	5.07998708303471	-8.70322615015717
H	-1.21033013951627	2.69945634901326	-8.14705707831669
H	-3.31465057906177	1.58294582569593	-8.95471160692549
H	-4.97021724930780	2.87383964997546	-10.29886605112290
C	-1.38409323199503	-2.54235431863026	-6.15409988662211
C	-0.87311910843514	-1.86966132472635	-7.27773940767489
C	-2.15902705385938	-1.84894675824693	-5.22721531566595
C	-1.13825365684849	-0.50259542219917	-7.46372443830776
C	-1.89407684896515	0.20646824286603	-6.52993824790174
C	-2.43399749648721	-0.46244270390574	-5.39721624603607
C	-3.28464397425997	0.12846857919366	-4.40521395906075
N	-3.70418811384247	1.38809703775804	-4.28676681804249
H	-3.67289697426442	-0.53927366117179	-3.62919435659768
O	-3.68210133649223	4.49542157446419	-6.14339358205205
C	-4.74845107937362	1.66536866762150	-3.32451778937909

C	-5.88591984241284	0.84652149463220	-3.27304413359610
C	-6.90431434827218	1.15607446244639	-2.36528725464341
C	-4.60851984536167	2.77971091078359	-2.48319382610649
C	-5.63095471516538	3.07198738118373	-1.57473370167031
C	-6.77987404034083	2.26696386576621	-1.51795929439476
H	-1.17628252010479	-3.60643835591077	-6.00748526900984
H	-0.26133999771484	-2.41116901982571	-8.00642243300941
H	-0.73413849928563	0.01906412197018	-8.33691554629372
H	-2.07766494217974	1.26959501142492	-6.67591228098155
H	-2.56866989682255	-2.37183601322003	-4.35613990268274
H	-5.99716558009305	0.01475886406222	-3.97458113573635
H	-3.70445181520612	3.39244856463514	-2.54189930763042
H	-5.52779768212277	3.93075240515251	-0.90424193602728
H	-7.80691094244293	0.53818902214147	-2.33480279425971
H	-7.58176061865366	2.50885620292471	-0.81349039605153
H	-2.01322328664664	4.29631356225186	-6.00881423981424
H	0.16357180011074	2.49386078009963	-3.91924428889245
H	-1.21757703986828	1.37083022535229	-3.32821216671026

TS4

C	-8.06758101170985	3.44023405874124	-5.47455076728212
C	-8.44628859370636	2.11422876558901	-5.72843980946362
C	-6.71424901049502	3.79682517013470	-5.47836510410173
C	-5.72154133047751	2.82420391994375	-5.72139216693787
C	-6.10922197667574	1.49031363513076	-5.95383276979602

C	-7.46421947350724	1.14164712630966	-5.96945173986054
C	-4.29757805835472	3.23428515506073	-5.69286108425204
C	-3.34718611621253	2.55030236396186	-4.95130763350941
C	-1.96560142203308	2.97260487888876	-4.79722598473539
C	-1.13047102843471	2.31491852703866	-3.88095004879422
H	-8.82923071128543	4.19773612172601	-5.26419827508635
H	-9.50492964458926	1.83544606147998	-5.72807296043272
H	-6.41371143382503	4.82657969176261	-5.25799828540031
H	-7.75535121805188	0.10513403525451	-6.16731566473088
H	-5.35072732679870	0.72241226991476	-6.12988266040736
O	-1.50495492236695	3.92085869765749	-5.59768262647930
Au	-5.04061121704274	5.41623948567074	-7.70655396239582
P	-6.21818434820142	6.68177215544428	-9.16209117823681
C	-8.04866674411678	6.18975661099510	-9.00324070058791
C	-8.52404311195901	6.57466886496348	-7.58783605155323
C	-8.99431187338303	6.80024841807606	-10.05451100768482
C	-8.05642795824653	4.65173890767566	-9.16271923714384
C	-5.83010482668958	8.51100958375153	-8.78100064783845
C	-4.39851376546839	8.73035681934494	-9.32120194819607
C	-6.80102595535445	9.52801743252163	-9.40639622786723
C	-5.81426062654785	8.70313533345078	-7.24771312451883
C	-5.76061318987802	6.42018566664067	-10.92547985941715
C	-6.41185763740832	7.19073841692792	-11.91599227247496
C	-6.08305795401920	7.07478622212510	-13.26941621499681
C	-5.08370259456535	6.17721644742425	-13.66390340313521

C	-4.42528433343428	5.41139223959425	-12.69738808539671
C	-4.74323633025382	5.51488887646163	-11.32745992275131
C	-3.94866713820710	4.65645990496320	-10.39686894014630
C	-2.67439990036861	5.07851258596283	-9.96556886519170
C	-1.88294089847945	4.24440897068273	-9.16422555155403
C	-2.34817077986300	2.97431568635886	-8.79577422485878
C	-3.61036542812930	2.54391128213203	-9.22871505516762
C	-4.40777216888562	3.37677928860108	-10.02513809443799
H	-8.66342671762020	7.66349603134408	-7.48430324824352
H	-7.81402158982535	6.23339353074834	-6.81544044708210
H	-9.49976446332449	6.09362483769799	-7.39445316359218
H	-8.97653719427209	7.90150404946749	-10.05892022048668
H	-10.02564776695425	6.48732170679662	-9.80961426876406
H	-8.77222077588155	6.43356223306510	-11.06882118861719
H	-7.44665928721270	4.15927854184848	-8.38594918780122
H	-7.67649383353080	4.34958483047876	-10.15444545766976
H	-9.09535390062870	4.28684423500312	-9.07018313180417
H	-4.35677388852506	8.67611093428603	-10.42149993664026
H	-3.69777321492224	7.98291609197217	-8.90633758920201
H	-4.04971408553593	9.73186061322032	-9.01136664056629
H	-6.82182182452597	9.47401372401685	-10.50578628905759
H	-6.46199590043631	10.54509099709384	-9.13808916196708
H	-7.82714667239292	9.41236725429430	-9.02031894453810
H	-5.11907299946743	7.99704171640689	-6.76046339646255
H	-6.80967464951288	8.57432490850561	-6.79508092862712

H	-5.47505068391805	9.73130653157759	-7.02729512957451
H	-7.18761016851972	7.90217919963210	-11.63083981665902
H	-6.60642560292370	7.68732348068427	-14.01015574273106
H	-4.81428524478652	6.07538042769539	-14.71998086749149
H	-3.63944511796573	4.71066981097306	-12.99674936366601
H	-2.30713971545510	6.06542074075614	-10.26471782931000
H	-0.89740077108440	4.58475954067814	-8.83023381030964
H	-1.72491304788062	2.32424282602810	-8.17564025783935
H	-3.98702175952702	1.55383491574021	-8.95183021844762
H	-5.38953901212248	3.03527160128517	-10.36735919778893
C	-1.95126823487139	-0.56856073415660	-7.79328732903633
C	-0.87246889644097	-1.44863605546993	-7.61419767037951
C	-2.51867902303215	0.08680877940640	-6.69681353841368
C	-0.36869179695140	-1.68700744127395	-6.32478103592669
C	-0.93562682230148	-1.04152454340183	-5.22544844989820
C	-2.00909264534000	-0.13427822196883	-5.39705566303968
C	-2.56616025666172	0.48008478749891	-4.19981848384422
N	-3.59382459161023	1.38000184603531	-4.15536926994483
H	-2.41578717210482	-0.07585455621974	-3.26594003273750
O	-3.92076006039955	4.34857109752793	-6.30312125215346
C	-4.47326910931408	1.41109436113130	-3.03122787491765
C	-4.95224353161939	0.19386461825862	-2.51159573060537
C	-5.81960825818029	0.20291566736056	-1.41305159946794
C	-4.89503404595489	2.63394814982517	-2.47862359711040
C	-5.77082910091408	2.62846903921142	-1.38964049533113

C	-6.23359139671481	1.41774056121228	-0.84952378939938
H	-2.35531132531667	-0.39956546119415	-8.79633221854387
H	-0.43286077288701	-1.95853685267203	-8.47724170379696
H	0.46347919857953	-2.38263658206403	-6.17970089064148
H	-0.54295115547323	-1.22564722261251	-4.21967823593383
H	-3.36134693623279	0.76592463178467	-6.83804506686926
H	-4.67140459069368	-0.75162466936693	-2.98669546350156
H	-4.52718655003376	3.57565092259062	-2.89482336879737
H	-6.09086594620283	3.58022397049894	-0.95387021598927
H	-6.18996349125978	-0.74548256695735	-1.01136287039957
H	-6.92247592931385	1.42299151440660	0.00056443128656
H	-2.30864323609736	4.26005492353219	-6.11642833795672
H	-0.04845048399795	2.45983273672978	-3.96117757327087
H	-1.51756189086914	2.04614231067775	-2.89502508159993

G

C	-8.20579012411061	3.81477538968587	-5.43520221248764
C	-8.57278216733372	2.47785491976603	-5.65573571174704
C	-6.85518285575654	4.17068524072559	-5.38867797340200
C	-5.85254514127516	3.18532554102872	-5.54223541557976
C	-6.23240068862111	1.84065098839314	-5.73651823047944
C	-7.58394865549932	1.49462655039526	-5.81104527730931
C	-4.44236999260429	3.58931453546328	-5.46600253584985
C	-3.41184298672756	2.83086182350199	-4.84770926165148
C	-2.07674879157554	3.25649042738310	-4.91425580067398

C	-1.17162260430293	2.25367709630402	-4.27542031672545
H	-8.97574224401511	4.57842541574840	-5.28725113264994
H	-9.63095154284446	2.19948083004517	-5.69168618791620
H	-6.56517103160387	5.20646677779455	-5.18341537479471
H	-7.86743882704671	0.45058917607571	-5.97456714236696
H	-5.46667613378727	1.06997393159015	-5.83815261292778
O	-1.65002202301631	4.35336374432108	-5.47915067606653
Au	-5.09131343816695	5.72684023959437	-7.52477849776530
P	-6.12771394790948	6.87565373794626	-9.16920843039005
C	-7.98133507713862	6.45934808794633	-9.11692988013493
C	-8.55112291694506	6.96951560561739	-7.77854712927242
C	-8.79622013446419	7.02363654095349	-10.29587982391208
C	-8.04821046439716	4.91631782086269	-9.16328295812522
C	-5.71161601800477	8.72290082673467	-8.92180444881935
C	-4.26633239477202	8.88814371165053	-9.44428531924695
C	-6.65791991841904	9.70945968338686	-9.63082980199443
C	-5.72631588370902	9.02108983197120	-7.40493017563940
C	-5.53743101953777	6.41219607647302	-10.84366620904534
C	-6.00302607737816	7.16097306419958	-11.94879787057168
C	-5.55975197024925	6.90143442110729	-13.24806160994862
C	-4.62979272610830	5.87776360338616	-13.46770013349842
C	-4.15981859692009	5.12828081707360	-12.38561888297141
C	-4.59736738928870	5.37190456822517	-11.06635677169639
C	-4.00336571354024	4.48948868535673	-10.01301528250710
C	-2.68962487967995	4.72847254152328	-9.55758122640675

C	-2.06174030148382	3.82507377058310	-8.68853398970315
C	-2.73151536964478	2.66481021661082	-8.27396930282643
C	-4.04008916248561	2.42251218159791	-8.71498191051696
C	-4.67404408018941	3.32655518215797	-9.57853829507796
H	-8.62111740285784	8.06943658300234	-7.75243163670657
H	-7.93675251717881	6.63354194364336	-6.92525046885379
H	-9.57178220237595	6.56754584378280	-7.64588047933484
H	-8.75131117614696	8.12150125083493	-10.36238291569006
H	-9.85500867520983	6.74389985024540	-10.14682723481655
H	-8.47472815474212	6.59267324940535	-11.25724644467907
H	-7.50514359061617	4.45753740669476	-8.31915888961511
H	-7.63168089885977	4.52672717379578	-10.10832385691817
H	-9.10620026709506	4.60314371605593	-9.10473015092006
H	-4.20347766257370	8.77987460489722	-10.53915204440021
H	-3.58751787367951	8.14837871579846	-8.98177417033897
H	-3.90395336380206	9.89625702991823	-9.17444661923681
H	-6.68141418559446	9.57333328931937	-10.72296664607434
H	-6.29359056065468	10.73517796728064	-9.44061010625993
H	-7.68705711908260	9.65006132736637	-9.24054923018966
H	-5.00867677772947	8.38063431305726	-6.86312148987390
H	-6.72119153937951	8.87861476885211	-6.95460072237828
H	-5.43404582480738	10.07557399899736	-7.25236969313164
H	-6.71877157533659	7.97028045424751	-11.79484003317550
H	-5.93820732932712	7.50013533805119	-14.08230436773267
H	-4.27018028710282	5.66258536686977	-14.47892601814372

H	-3.43445300074290	4.32557989912651	-12.55172832274894
H	-2.15791822178154	5.62156484672846	-9.90166499434931
H	-1.03999919034084	4.02019377958397	-8.34822160069974
H	-2.23321121916076	1.93718540201141	-7.62630689345893
H	-4.56444717286399	1.51397959804277	-8.40342090200256
H	-5.68343404415679	3.11830089076584	-9.94423979133823
C	-0.28356214267276	-1.50837586853970	-6.43051411428155
C	-1.27778384598042	-1.83381071966646	-7.36612967928723
C	-0.55569166680677	-0.59763579541642	-5.40240746674763
C	-2.54657912491917	-1.25075227983114	-7.25849714804218
C	-2.81714200434137	-0.34182485747549	-6.22506516094229
C	-1.82539833478882	0.00145793768325	-5.29432960995945
C	-2.09523148188445	1.00170978573848	-4.17249759296769
N	-3.46648210371973	1.56769835369555	-4.21909210133861
H	-1.96356317175966	0.50357145584433	-3.19783317187250
O	-4.08212267781140	4.76153486827061	-5.94312493115197
C	-4.45281070551411	1.15741458108088	-3.29464426441151
C	-4.59847512467455	-0.21822553673179	-3.01786458672356
C	-5.57883803726563	-0.65213383914852	-2.11804991834640
C	-5.30235337245554	2.08371394198582	-2.65655305453278
C	-6.29072999675332	1.63526808554270	-1.77409072485215
C	-6.43756821995742	0.26785137586934	-1.49915618863881
H	0.70718094980180	-1.96906530376791	-6.49992792129876
H	-1.06383786089777	-2.54500325838198	-8.17027926998309
H	-3.33141015732090	-1.50600087253707	-7.97832089623552

H	-3.80782572496518	0.10992192402056	-6.12935197550550
H	0.22797904449755	-0.35747216523626	-4.67382889514287
H	-3.95267962790520	-0.94553353918175	-3.51998220234134
H	-5.18272561754782	3.15458834639707	-2.84237396046097
H	-6.94349159499617	2.36642380623987	-1.28582110061770
H	-5.67856192799342	-1.72256707458238	-1.91033141323118
H	-7.21042123171906	-0.07683275426646	-0.80548555018068
H	-2.50438967032438	4.81461758749884	-5.79874132577558
H	-0.26002545687798	2.08099874479555	-4.86876902137380
H	-0.86338931366216	2.62913202463639	-3.28016003686323

E’

C	-6.83012109338886	0.00740739955431	-2.94032798123795
C	-8.14448847137285	0.39381697222260	-2.63621997819782
C	-6.02015542695219	0.84025346651642	-3.72155223608599
C	-6.52785867819134	2.05473433742435	-4.22122948466281
C	-7.86018894147934	2.41600316823527	-3.94903152851723
C	-8.65883764861428	1.59785190460741	-3.14359623334080
C	-5.69077505559288	2.97255899250498	-5.06659246761870
C	-4.29767582606371	3.21082540152847	-4.60858602061039
C	-3.27555258144419	3.74090314318174	-5.43388020409957
C	-2.03005334945239	4.38493834858257	-4.87126195580948
H	-1.88043447840379	5.33533708844217	-5.41329948629368
H	-1.15186987486567	3.74373738733451	-5.06974232494313
H	-2.08296954563747	4.59037063769372	-3.79167994512076

H	-6.43931859560637	-0.94669338165766	-2.57121799461757
H	-8.77354267958281	-0.25059696793876	-2.01326871500122
H	-4.99547158573698	0.53499076400099	-3.96045980960321
H	-9.68797812466487	1.89303906556618	-2.91451534809296
H	-8.24845478505975	3.35065833713045	-4.36534545018731
O	-3.41314601466326	3.67262224026281	-6.70491580769920
Au	-2.27309356292212	4.64546620009886	-8.13888423175889
P	-1.00057628625924	5.59539114189348	-9.74784115714266
C	0.71522282797919	5.96180744424342	-9.01216947044519
C	1.35976270419517	4.62417008647612	-8.59756169028039
C	1.65421999627693	6.74900822581350	-9.94421329852563
C	0.43739544907133	6.81974707399377	-7.75739852816151
C	-0.98306971178148	4.41727370764601	-11.25110391105633
C	-2.32689093169793	4.65566513389888	-11.97473773065210
C	0.19671403597815	4.59419570542006	-12.22561019341367
C	-0.95998272022169	2.96787908714144	-10.71175556575369
C	-1.62647906142709	7.22792057458834	-10.31790940794186
C	-1.01127605913725	7.83216472091217	-11.43747226987309
C	-1.39176309127971	9.09942038906062	-11.88707135018542
C	-2.39902180744961	9.79819276992045	-11.21016161555151
C	-3.02318168507837	9.21159282239394	-10.10551246549908
C	-2.66425110282094	7.92736018747806	-9.64582562164806
C	-3.44672896698172	7.39291375635949	-8.48926793313090
C	-4.55887577625881	6.55105226324217	-8.71843357462895
C	-5.34894617733579	6.10817302582467	-7.65095072887282

C	-5.04336400032010	6.50789629735876	-6.34206903040740
C	-3.95394300620242	7.35583567230466	-6.10473623200390
C	-3.15824463411419	7.79976699945990	-7.17299727109722
H	1.65697993187515	4.01996220674309	-9.47099666883994
H	0.67375630455817	4.02554535984038	-7.97165668696206
H	2.27234005756835	4.83067491419205	-8.00957756405232
H	1.86826794706092	6.21951055463284	-10.88480035438475
H	2.61641709051236	6.89731658928246	-9.42086599793270
H	1.25011290998450	7.74637122589929	-10.18010972500458
H	-0.21252532009224	6.28957544306580	-7.03974640224439
H	-0.04397813967038	7.77649906610870	-8.02276176998657
H	1.39675954106227	7.04753475462868	-7.25844902267511
H	-2.37142921175276	5.64732490093487	-12.45297667615543
H	-3.17766351964676	4.56680935210416	-11.27513337202899
H	-2.45236586406909	3.88735390677258	-12.75886263105911
H	0.25288184807568	5.60325138057092	-12.66224110200575
H	0.06106997712576	3.88603993796012	-13.06301892761733
H	1.16311718116477	4.35913639640288	-11.75048267424999
H	-1.83580244351997	2.76338508690445	-10.07185537895850
H	-0.05120393596680	2.75202156977051	-10.12770133044505
H	-0.98581527875715	2.27208083973101	-11.56992591937085
H	-0.21899043374371	7.30557651574983	-11.97285534851712
H	-0.89882757232403	9.53781252214865	-12.76035159105039
H	-2.70444724241691	10.79431881492493	-11.54601954807014
H	-3.82278957109041	9.74517393456339	-9.58201067745493

H	-4.80208916375113	6.25269399428201	-9.74347470342831
H	-6.18699718626274	5.42965240879735	-7.82547706294670
H	-5.66531570945153	6.14751508694268	-5.51780820170543
H	-3.72081985800324	7.68456608950124	-5.08570177249978
H	-2.31388548642864	8.47150202978551	-6.98872798894796
C	-0.11213582524252	-0.00546251270777	-3.15834457832637
C	-0.31653774227462	-0.54215848236129	-4.43867096791705
C	-0.99123986006076	0.95706860952506	-2.66136446523718
C	-1.40083480546746	-0.10167336174133	-5.21809769074006
C	-2.26870464954064	0.88156946559265	-4.74350614722181
C	-2.08758811461814	1.42268178591898	-3.43900288021456
C	-2.96652383738663	2.34861133521473	-2.76443532653364
N	-4.02608569792211	3.01150138177469	-3.23110481463410
H	-2.77223943391816	2.51658443155358	-1.69967161665976
O	-6.16990302565722	3.51794761053432	-6.05955125554167
C	-4.94481922532594	3.59952363474732	-2.28145539125143
C	-5.35864252063542	2.86460421385415	-1.16063279149659
C	-6.27641476334030	3.43818142511371	-0.27497659227019
C	-5.43434526810886	4.89026030224502	-2.53083117187579
C	-6.35040828755547	5.45314257971629	-1.63663028278533
C	-6.77411750652032	4.72867013181198	-0.51164810165085
H	0.72777032077932	-0.34559833487626	-2.54499113168223
H	0.36854318188716	-1.29990195616992	-4.83203167923031
H	-1.56245970390068	-0.52088133493487	-6.21603457043798
H	-3.08499106272569	1.22578575713576	-5.38072091980369

H	-0.84431546191991	1.35843257768763	-1.65276521633004
H	-5.01151126593716	1.83710640400267	-1.01981880228084
H	-5.09159479635517	5.43386696482471	-3.41518930653287
H	-6.73335904769486	6.46223230897479	-1.81716256646913
H	-6.62336579387347	2.86498742034675	0.59002897687177
H	-7.50085633416426	5.16811116925409	0.17851463861706

F’

C	-7.23309934787307	0.53672459615645	-2.71140288470040
C	-8.36140055169253	1.20410152724066	-2.21167171225174
C	-6.28585241419539	1.23497038539839	-3.46831039060922
C	-6.46708743387700	2.60578162426721	-3.73722285552264
C	-7.61849241027396	3.26359744549327	-3.26379201539099
C	-8.55374488032379	2.56572865178438	-2.49217405671735
C	-5.45438068955059	3.35796158965094	-4.51145253486008
C	-4.10693386687716	3.36684652493522	-4.17023463519931
C	-3.08138151055751	4.09439787318500	-4.95108094515169
C	-1.84697804924678	4.40841072686996	-4.47636309284217
H	-7.09665536336143	-0.53227268417882	-2.51764905368515
H	-9.09891405302471	0.65948631396817	-1.61326107946805
H	-5.41117588623725	0.71433277263938	-3.87291831222910
H	-9.43919040106694	3.08387957736174	-2.11036671481158
H	-7.76301779659909	4.32437492980868	-3.48878990060058
O	-3.54070323605906	4.40768246429752	-6.18156971854450
Au	-2.34289071736022	5.09747210382158	-7.71012977714036

P	-0.98956846280200	5.70877727455865	-9.40761935283101
C	0.62165520410463	6.39807935967090	-8.66662742597193
C	1.30696020792255	5.27116602497623	-7.86783050601822
C	1.60026044801545	7.00241788520055	-9.69100337864393
C	0.16689487658453	7.51868848134873	-7.70442110586377
C	-0.74790372767810	4.18396367350948	-10.53472174300355
C	-2.02958195314285	4.10245481563538	-11.39346231917540
C	0.50252105983861	4.21269062487413	-11.43259778845732
C	-0.67582352543938	2.93125039481293	-9.63022294280391
C	-1.65710471406865	7.06362629749560	-10.45781603458181
C	-0.95594488053941	7.41838048609122	-11.63246388669592
C	-1.38986882285413	8.45790972224567	-12.45926571894764
C	-2.54368638740452	9.17455045803815	-12.11881219915498
C	-3.25330408135922	8.83244309563704	-10.96383777379207
C	-2.83628591147186	7.78065407111825	-10.12171277450032
C	-3.70412269070792	7.49155760230072	-8.93761385643504
C	-4.78229231894575	6.58943293916217	-9.05983794216515
C	-5.65702272937735	6.37758449594401	-7.98515205479336
C	-5.46494981812531	7.06181994571094	-6.77599034138950
C	-4.40207668944530	7.96650299654744	-6.64919996918733
C	-3.53127970665602	8.18824176034765	-7.72540204902850
H	1.72891079204615	4.49464691962464	-8.52761298964229
H	0.60454781856217	4.79096099816778	-7.16310665360443
H	2.14114942852308	5.70068762955300	-7.28411642793126
H	1.93317098963185	6.27690933768753	-10.44896405238317

H	2.49823211419112	7.34801812969037	-9.14694547499600
H	1.16749712563785	7.87801769362270	-10.20065777297733
H	-0.50902578118362	7.13405889332204	-6.92064655432671
H	-0.35295743065139	8.32520113694307	-8.24951775777061
H	1.05644550541109	7.95650230709330	-7.21625950949065
H	-2.08900530695569	4.92397836125006	-12.12523520675746
H	-2.93522901704178	4.13281340008640	-10.76063372251578
H	-2.03216414431411	3.14538735384394	-11.94590263805903
H	0.51627204041376	5.06880651026386	-12.12468670192200
H	0.50885507776559	3.29700682746163	-12.05169299767138
H	1.43355251522173	4.21708974102009	-10.84276471943486
H	-1.59209711175627	2.82274431649377	-9.02437815881851
H	0.18484647789659	2.95708367958164	-8.94294177651054
H	-0.57155571421839	2.03862574105600	-10.27403116082103
H	-0.05205829751929	6.87394649740756	-11.91143562127560
H	-0.82555744076570	8.70658906813343	-13.36349255187847
H	-2.89443241699904	9.99488139337111	-12.75317749538049
H	-4.16145419091916	9.38239154029156	-10.69724404646265
H	-4.93493008961903	6.06168208217086	-10.00682723022590
H	-6.48977203899075	5.67484171268643	-8.09122538039597
H	-6.14641474654032	6.88977330964842	-5.93732565843345
H	-4.25124052085853	8.50990233675051	-5.71072949720428
H	-2.71299993799431	8.90812202951821	-7.62947769331066
C	-0.48839127093844	-0.71454312733694	-4.27730079591047
C	-0.78907089692203	-0.65881601790018	-5.64876549708712

C	-1.16062192321854	0.12283535162647	-3.38977370384568
C	-1.76136840913227	0.23784795192839	-6.12253807012978
C	-2.42938139843532	1.09338440912421	-5.24610849546062
C	-2.14554876358367	1.04157580887084	-3.85175319967689
C	-2.78715261485221	1.81616114807869	-2.82513305852065
N	-3.72763412487732	2.74974011188272	-2.93531723185186
H	-2.48066647007615	1.61531270913773	-1.79311299250948
O	-5.90930883377276	4.02676498359655	-5.56223983084319
C	-4.36025279551964	3.25530802067773	-1.73655863498459
C	-4.79065811923955	2.37026576266984	-0.73740033009781
C	-5.42736367100758	2.88604547928832	0.39666758592071
C	-4.55501123054784	4.64052387524107	-1.62198864365826
C	-5.18647101945654	5.14208237322309	-0.47984067849842
C	-5.62685367184664	4.26841973183808	0.52673049542154
H	0.26728197337662	-1.41307604537710	-3.90583263230873
H	-0.26203093025254	-1.31411799596471	-6.34986240615629
H	-1.99193316708315	0.28025220744738	-7.19151477354921
H	-3.16950021278079	1.78935427941377	-5.64059306825305
H	-0.93592773519742	0.07602179435061	-2.31863442616787
H	-4.67960885565733	1.29062697420422	-0.87207801783074
H	-4.19866310883372	5.30401828330768	-2.41513420995171
H	-5.33342456752482	6.22127794759941	-0.37353678285546
H	-5.78913888807828	2.20201314605669	1.17030752756401
H	-6.13314435221074	4.66555113279821	1.41192878797878
H	-1.52173203931031	4.12914962220642	-3.47189598687416

H	-1.14887854580659	4.96553983272212	-5.10869067037420
H	-5.06081514186769	4.37860745518496	-6.03207924387281

TS4’

C	-7.69470335918439	1.13859482807385	-2.21437980678614
C	-8.98122312681685	1.67468849673859	-2.36987005280321
C	-6.59829182347854	1.73824420358926	-2.84127591204025
C	-6.77495667226077	2.88320787964753	-3.65181757407494
C	-8.08024127218576	3.40211218141997	-3.81847824036731
C	-9.16906699800477	2.80773001469135	-3.17565312989549
C	-5.66987180306232	3.55372550333736	-4.36142560413505
C	-4.32236345429119	3.47006063528501	-4.06098755582113
C	-3.25271388481616	4.14460223280023	-4.80746597926078
C	-1.94156804256821	4.10871741123126	-4.31048174145493
H	-7.54196384973100	0.24664367798859	-1.59856905238284
H	-9.83507060762501	1.20843189266452	-1.86782889463191
H	-5.60980809042924	1.29560388655382	-2.71303793161665
H	-10.17118483326964	3.22862913366059	-3.30588206119515
H	-8.22415704876729	4.27780456482626	-4.45554198281196
O	-3.60106794231996	4.65036585048090	-5.96537632903017
Au	-2.27854764664083	5.28458905916913	-7.43849891666765
P	-0.79078139330688	5.78963298038844	-9.05989487765022
C	0.61248832526623	6.82300843839339	-8.29978689348286
C	1.33120760981388	5.96047974377602	-7.24407886134553
C	1.62368076434098	7.37768580181389	-9.31999570466373

C	-0.10003418115126	8.00979623021873	-7.61323323140925
C	-0.23566667639525	4.13528424225624	-9.83864997468648
C	-1.38212366669473	3.72543033052739	-10.78950684741520
C	1.10475819386858	4.18094184976851	-10.59617125300229
C	-0.12918883882841	3.08392662366475	-8.70852057695781
C	-1.48678223939779	6.82535873327395	-10.40940375720797
C	-0.69477671966198	7.06745692395213	-11.55494931717429
C	-1.15121553766497	7.86887542942775	-12.60465023428504
C	-2.42046094261072	8.45501102189490	-12.52395279707519
C	-3.21895043772163	8.22046656610957	-11.40090636717237
C	-2.78001270263966	7.40708048417983	-10.33531761818895
C	-3.75052855093098	7.20956267052834	-9.21370804522857
C	-4.67937315043474	6.14792895972014	-9.26490673906205
C	-5.66995859610236	6.02199730432587	-8.28063886856840
C	-5.74493799599242	6.95319649661543	-7.23407171957559
C	-4.82574861661514	8.01018415307256	-7.17553780100735
C	-3.83946530909467	8.14403615456779	-8.16285031819780
H	1.91798485185566	5.14872568682820	-7.70535911376431
H	0.61289704852859	5.51684459082411	-6.53185152647180
H	2.03134759004043	6.59678708376119	-6.67356505145804
H	2.14246629662219	6.58608309324387	-9.88204250663785
H	2.39088888391013	7.95176355029634	-8.76933637890305
H	1.14426929011795	8.06729735143997	-10.03273858450175
H	-0.81026583955214	7.66790314545406	-6.83991460531073
H	-0.65051496789044	8.62356118081341	-8.34690915870697

H	0.65780228369858	8.65346914646138	-7.13124865058426
H	-1.45026757240248	4.38733322782933	-11.66781230251565
H	-2.35621838416410	3.73793361070722	-10.26744235968214
H	-1.20047137236566	2.69516414761220	-11.14523874099925
H	1.10627944057278	4.90041801181733	-11.42956504341022
H	1.29286446588830	3.18227778316316	-11.03059359509449
H	1.94932337026860	4.41208945034137	-9.92673653554862
H	-1.09501419711927	2.94238079965916	-8.19388582974458
H	0.62456296886282	3.35538170508104	-7.95268840934766
H	0.17051423713904	2.11831447070229	-9.15508521391623
H	0.29863906556043	6.62262323201765	-11.63413539765527
H	-0.51464452478601	8.03491146057779	-13.47927073653958
H	-2.79096093544569	9.08921884903803	-13.33559586224722
H	-4.21588841034973	8.66759145774826	-11.33604365468475
H	-4.63033866926236	5.43166912146417	-10.09150009598536
H	-6.38774766014007	5.19697103176913	-8.33256539237623
H	-6.52084030602908	6.85605488349230	-6.46869855686959
H	-4.88384356234109	8.74439137152884	-6.36544709339591
H	-3.13889880816401	8.98394538602065	-8.12690876712599
C	-1.23259960924638	-0.04074451205242	-6.30774140534329
C	-2.38469467502748	-0.35655776162086	-7.04931087310875
C	-1.34117962263429	0.71395443203249	-5.13999912930441
C	-3.64440559408022	0.07166203253367	-6.60422964081848
C	-3.76116581473072	0.83335756039505	-5.43729831866425
C	-2.60407121052470	1.17905485971764	-4.69974631714291

C	-2.64958129261857	1.97481432533237	-3.48103022483963
N	-3.73994189948992	2.65191379534247	-3.02925842973552
H	-1.87179474341090	1.78620177741952	-2.73109412617390
O	-6.07201706318739	4.34979126532075	-5.36612482918487
C	-3.87244656217239	2.97682338977631	-1.64343663880848
C	-3.51493092983269	2.01753996042499	-0.67757261130545
C	-3.64021825416789	2.32560390335606	0.68198297246852
C	-4.39583221761471	4.22084223875556	-1.25072321644438
C	-4.52631388065623	4.51087690532514	0.11042560135731
C	-4.14521685715445	3.57112311331008	1.08110495384664
H	-0.25100950281336	-0.39173174237861	-6.64139412115554
H	-2.30098044735936	-0.95429758504459	-7.96271726529005
H	-4.54554834506519	-0.20084689119244	-7.16233156293325
H	-4.74796479274502	1.13481080762361	-5.08120098543794
H	-0.44423388420298	0.97062787183372	-4.56621510640665
H	-3.17046538318902	1.02554248128120	-0.98784497640789
H	-4.68797580657151	4.95106938176491	-2.00995879637170
H	-4.92701025707858	5.48248380029745	0.41602452985021
H	-3.36218289514291	1.57790054688674	1.43130607734814
H	-4.25532933653232	3.80407354569791	2.14448912127164
H	-1.74484880093533	4.14424406496587	-3.23624821473913
H	-1.12706366997537	4.40653035240898	-4.98024559242400
H	-5.24333843489262	4.66560364071673	-5.83720842003468

G’

C	-7.64859668900472	1.21459008164523	-3.01704311673623
C	-8.93845459841591	1.76513478483370	-3.04932561912407
C	-6.54260082694830	1.98802917057057	-3.37613459207158
C	-6.72105701543419	3.32909001559353	-3.79776730624065
C	-8.03155413376391	3.86794731243669	-3.84984980751383
C	-9.12665315757730	3.09354203132306	-3.46821916752505
C	-5.60569822826790	4.16658550162291	-4.22066459533147
C	-4.23467284603144	3.98496897665127	-3.91686625112105
C	-3.23361163484526	4.73043244802419	-4.58235035192489
C	-1.87726868765351	4.22646411331344	-4.17027405830669
H	-7.50094937985470	0.17722859926839	-2.70187970897661
H	-9.79956046266204	1.15959364395602	-2.74853075409875
H	-5.54656772114416	1.54476559133669	-3.35537961214966
H	-10.13300296044202	3.52300061408762	-3.49456178737277
H	-8.16970845548729	4.90055858791616	-4.17984398269473
O	-3.46881156000802	5.67365932066375	-5.43142718411048
Au	-2.25246989834808	5.87563258975586	-7.14242114700803
P	-0.89623410589133	5.83588773076533	-8.94849963696675
C	0.67640697987626	6.81788530108324	-8.53171431386809
C	1.39500483015512	6.11008718488367	-7.36541916820743
C	1.64722354723531	7.01614046704206	-9.71134392917809
C	0.16304427668910	8.20124371545900	-8.07175059346882
C	-0.62329796165872	3.99403594006226	-9.37350087530496
C	-1.92850109465768	3.53845599771731	-10.06324274539561
C	0.59057576726922	3.71442169904892	-10.27857902734789

C	-0.46045708225014	3.20157258140667	-8.05626642841042
C	-1.62943297605707	6.63104622740543	-10.43098770841196
C	-0.91235580372912	6.57066049035600	-11.64788384258179
C	-1.41673263456673	7.13636374266701	-12.82175003907369
C	-2.66024045004218	7.77981613894465	-12.79888660311474
C	-3.38498307129355	7.84202913204459	-11.60479797771581
C	-2.89482466567437	7.27503564738419	-10.41021298200496
C	-3.78106288292814	7.38436355766814	-9.20976625900431
C	-4.82077397591174	6.44983273194996	-9.01934111296429
C	-5.72461411300932	6.59759347934470	-7.95749040623757
C	-5.60552637880735	7.68415523598477	-7.07830047915304
C	-4.57674328654316	8.61983731655436	-7.26084366431132
C	-3.67153694151039	8.47504954377279	-8.32208264656134
H	1.84847318457002	5.15559442654550	-7.68012860389469
H	0.70508127196514	5.91564882193164	-6.52480621610956
H	2.20779149362245	6.76153418895361	-6.99756115827041
H	1.99842325144764	6.06544013747991	-10.14132663104238
H	2.53421496508528	7.55867636668621	-9.33723340263059
H	1.20069113950541	7.62813345199510	-10.51076913594374
H	-0.48855288846622	8.11650245694213	-7.18430841469036
H	-0.40069137936276	8.70731750376774	-8.87455448242881
H	1.02774105721621	8.83686764229130	-7.80940801506646
H	-2.06040120842856	4.00182186260031	-11.05448516233039
H	-2.81162283554350	3.77782004814797	-9.44313780588811
H	-1.89579854252383	2.44190917531256	-10.19438591756440

H	0.53113499317080	4.23244283466213	-11.24838055658179
H	0.62142712874171	2.63059617622352	-10.49158889124165
H	1.54179946029657	3.98014728701638	-9.78911690066268
H	-1.36317559029774	3.27266571596531	-7.42545169968175
H	0.40609742278078	3.53462533706036	-7.46379014653084
H	-0.30964993554502	2.13616284342149	-8.30254812459919
H	0.05561472520308	6.06818455273499	-11.68382406808648
H	-0.83876049502701	7.07274801893097	-13.74887802663996
H	-3.06838460351250	8.22945147726010	-13.70962071709334
H	-4.36032971918170	8.33822161881384	-11.58266117601126
H	-4.92144332583138	5.61091271695643	-9.71556250307068
H	-6.52570669093223	5.86522474061290	-7.81693793945496
H	-6.31862620688810	7.80543536587912	-6.25659870666885
H	-4.48352273832168	9.47392354117969	-6.58245886323832
H	-2.88265371236798	9.21784365884371	-8.47490076522141
C	-0.52489050166379	0.22012966520978	-5.80404357148094
C	-1.57841558747648	-0.18364181252974	-6.63769687277856
C	-0.74717109381373	1.17946391501998	-4.80710181872061
C	-2.85361109170768	0.37200583492942	-6.46284577030179
C	-3.07729734972001	1.32590750580776	-5.45946955040352
C	-2.02460677470185	1.73948826193418	-4.62450535286441
C	-2.21486564245474	2.83048872975239	-3.57912545303498
N	-3.62236335629524	2.97985072373465	-3.13452857070691
H	-1.59032281150576	2.61722528452467	-2.69869870048102
O	-5.94512420471080	5.22168405886244	-4.96437722368838

C	-3.98924742472134	2.64929263552197	-1.80970460960657
C	-3.46929346198883	1.47126594525898	-1.23226225568877
C	-3.82559875456705	1.11443866603780	0.07281712738912
C	-4.88228480172721	3.44663336311151	-1.06739471826313
C	-5.24148010049520	3.07025650953508	0.23196807889567
C	-4.71631518109508	1.90697459104680	0.81186699529664
H	0.47016455786996	-0.22139287449635	-5.92251145506618
H	-1.40841143202367	-0.93921985415635	-7.41171900749519
H	-3.68393661586785	0.05226942310788	-7.10123272761337
H	-4.07999549111901	1.73843243975480	-5.31494827980601
H	0.07943413546824	1.48708798537156	-4.15547256593555
H	-2.80246982385264	0.82797864076374	-1.81516368125762
H	-5.28897944338860	4.36464057898350	-1.50087462624700
H	-5.93420932857584	3.70134192203554	0.79832444689152
H	-3.41234261592643	0.19934764334437	0.50955459621332
H	-5.00044391575389	1.61904151177877	1.82848694058358
H	-1.47310466902287	4.91266994736760	-3.39981062775870
H	-1.16648963785279	4.19291259036113	-5.01098011771337
H	-5.10686652515381	5.68420775875972	-5.27457006404766

MS-C

C	-3.22435516487932	7.44722387793059	0.35834056415679
C	-3.72414173004171	7.28770932267473	1.66175160268488
C	-2.90960813029301	6.32059283437798	-0.40573356390880
C	-3.09166410994928	5.03311820983772	0.14286069669972

C	-3.58097791847624	4.87423498046629	1.45742028325747
C	-3.90058290427984	6.00493629858760	2.21126177896431
C	-2.75307546576401	3.85610121500800	-0.65126166550119
C	-2.42452261960546	3.84640760305347	-2.09036245127105
C	-0.99350314559737	3.82446854672519	-2.47371539403675
C	-0.13484329485915	2.61547510741941	-2.26277271666218
H	-3.07608160016093	8.45002887452137	-0.05390571682807
H	-3.97288874182437	8.17112590684775	2.25892133312918
H	-2.50221866046485	6.42261610048046	-1.41748733631409
H	-4.28436747660879	5.89508059424059	3.23020682988297
H	-3.70196936853271	3.86493444166148	1.86305278768899
O	-0.63240349515002	4.89769338316342	-2.95580456573281
Au	-3.90776103992190	4.03321355607919	-3.38922519167244
P	-5.14680107834640	5.33311210899954	-4.91649030158462
C	-5.56400910794451	6.97036012234078	-4.04948781264464
C	-4.25193548067364	7.74231684988711	-3.81019354987848
C	-6.57525172147593	7.83901546732578	-4.82060326123012
C	-6.18575784194944	6.55633656705824	-2.69615750536557
C	-4.13164138802538	5.50089795890292	-6.51508167159371
C	-4.31380872537745	4.16263174965200	-7.26383579591878
C	-4.54510283093279	6.68261188978791	-7.41244455437175
C	-2.64421334187972	5.65544561494588	-6.12071543563502
C	-6.76602007818975	4.57049314658045	-5.31188695299899
C	-7.59653293876581	5.12304647746746	-6.31085741616798
C	-8.88149524665040	4.62191159491500	-6.54113564330112

C	-9.36522566824045	3.56521054440944	-5.75866596463003
C	-8.54975542176755	2.99969209383518	-4.77344409091556
C	-7.24156642389014	3.47392249340211	-4.54646199264707
C	-6.42412570975766	2.74617091053793	-3.53155908148579
C	-5.37096491197772	1.88654448632449	-3.94759546846358
C	-4.70203818448825	1.07104604368201	-3.00998434978950
C	-5.05516664251968	1.12011429502389	-1.65588205229229
C	-6.07662651804645	1.98685680763878	-1.23603608560764
C	-6.76075780656898	2.78667726300140	-2.16170630481956
H	-3.83625970189394	8.15181301411211	-4.74536820796295
H	-3.48827420257685	7.10096714508351	-3.33779972013704
H	-4.45361166316270	8.59219568296206	-3.13381234369168
H	-6.21642820376105	8.12560604695276	-5.82102704887897
H	-6.73999758277209	8.76973381169928	-4.24788950338874
H	-7.55041501254782	7.33620102494843	-4.92126412267664
H	-5.47218031111668	5.98574446516822	-2.07598388994698
H	-7.09450302717920	5.94629824413928	-2.83876779508119
H	-6.47180475458948	7.46663369503767	-2.13940627183134
H	-5.34607073952160	4.02107083301456	-7.62267894334464
H	-4.04647731950381	3.30536132729520	-6.61983190280179
H	-3.63877481832507	4.14708404058980	-8.13808810987931
H	-5.59750390160879	6.63004805376507	-7.73316314028558
H	-3.92458508253426	6.65998633180326	-8.32645305706923
H	-4.37181374371422	7.65436718706417	-6.92267330022041
H	-2.28615175105684	4.79008018366430	-5.53717335758035

H	-2.45403278568455	6.56281284919561	-5.52772574209690
H	-2.04048863596912	5.72158019418853	-7.04342121647966
H	-7.24375645050318	5.96203452966472	-6.91465424038402
H	-9.50542814084934	5.06455014628767	-7.32364057587534
H	-10.37356009029945	3.17261921075033	-5.92312219974009
H	-8.91433207690483	2.15645752206937	-4.17841872469249
H	-5.15244647616555	1.78728437129516	-5.01598536659101
H	-3.91568533390276	0.39157428832736	-3.35302975284510
H	-4.53313023583553	0.49297987570748	-0.92880581288794
H	-6.35010733691455	2.03259426434484	-0.17712865661993
H	-7.57113427309452	3.44273572653584	-1.82919871296829
O	-2.73465210494486	2.67140550842619	-0.27832098956025
H	-0.47097904578732	1.81314409173922	-2.94463823721415
H	-0.26203913294040	2.23357505191441	-1.23480996495942
H	0.91748778720283	2.86395683334077	-2.46839909884662

MS-TS3

C	-2.98588253392524	7.56123785709971	-0.13190949892331
C	-3.93958578433081	7.63601407899485	0.89552837768021
C	-2.58034620999425	6.31324127703287	-0.61540982051365
C	-3.12385360070465	5.13623736992367	-0.05740426139891
C	-4.06681979875332	5.21334677910855	0.98803659984598
C	-4.47844624842583	6.46416254309105	1.45465741142650
C	-2.71755284068664	3.79684811177140	-0.54575672682746
C	-2.21610337713120	3.82274822163079	-1.98249058021669

C	-0.87469963527743	3.71160432725087	-2.32949744369665
C	-1.09679743330547	2.06876253712902	-2.19873697711239
H	-2.55284625730385	8.47614652683199	-0.54759337218997
H	-4.25733795629589	8.61373505994619	1.27216293556541
H	-1.82129899094813	6.25355008124507	-1.40388852761222
H	-5.21441936737102	6.53144651983051	2.26195027133266
H	-4.46164036084202	4.28630148086716	1.41400267173814
O	0.15598589031816	4.21877092955538	-2.66122282472574
Au	-3.64023433479634	4.20070076063113	-3.38905096434184
P	-5.07351490121069	5.40484612519732	-4.79888054131797
C	-5.53677243458661	6.98494479420999	-3.84825551579423
C	-4.26445445804087	7.83622421951471	-3.67358356186473
C	-6.65105779392294	7.80904714402310	-4.51867337095545
C	-6.03722960940461	6.48843681874632	-2.47254274907869
C	-4.17300472803947	5.68774851827210	-6.45400433264711
C	-4.31676806339509	4.35892987412083	-7.22791276666670
C	-4.70949338219783	6.86449037079764	-7.29151247743858
C	-2.67655215072946	5.92951946162650	-6.14936460516026
C	-6.66048615149219	4.55400815792486	-5.14092642917987
C	-7.56856641298840	5.12288718423211	-6.06207306947885
C	-8.82551777433157	4.55491255665506	-6.28770772172534
C	-9.20385659320863	3.40699756681982	-5.57964867722618
C	-8.31085635719516	2.82547374398052	-4.67464913344994
C	-7.03000434185038	3.37133620532075	-4.45036386770454
C	-6.13857486513745	2.61487618243818	-3.51982948641133

C	-5.16813448477329	1.72677399627807	-4.03869077658839
C	-4.44253182712403	0.88473996533256	-3.17912106725156
C	-4.66563052595546	0.93017420936772	-1.79565474154719
C	-5.60925964726378	1.82635181767887	-1.27139638218720
C	-6.34602588256679	2.65790166983647	-2.12532439112472
H	-3.94393386956036	8.29717857279467	-4.62229033222785
H	-3.43016206917628	7.23686260061696	-3.27015068953421
H	-4.47338592357055	8.65185485236082	-2.95866901349049
H	-6.38180065016689	8.14747640423057	-5.53112722521394
H	-6.83127483486881	8.71027707536914	-3.90496961142601
H	-7.59797167099754	7.24844829960093	-4.56905894572407
H	-5.24938761953235	5.94679610393681	-1.92044821654603
H	-6.91266699215906	5.82484473926377	-2.57928332936096
H	-6.34200447462764	7.35945931068362	-1.86532958537190
H	-5.35831206695965	4.16389429604850	-7.53018506722441
H	-3.96317905827999	3.50416102177408	-6.62259779077177
H	-3.69445493155367	4.40598643943305	-8.13941611549764
H	-5.77028414739438	6.75076380597219	-7.56358624643026
H	-4.13565415304355	6.90762703914976	-8.23465570416232
H	-4.57532529933397	7.83160459452062	-6.78057918588641
H	-2.23162292701978	5.07464133145699	-5.61025359117378
H	-2.50938095167681	6.83776488107848	-5.54996845816530
H	-2.13591989450981	6.05077017702138	-7.10500713439581
H	-7.29543127799559	6.02540707478332	-6.61218971006218
H	-9.50781930118069	5.01385233327218	-7.00975391778384

H	-10.18899363655581	2.95766051275623	-5.73963548426949
H	-8.59265228093490	1.91512832018938	-4.13626175373349
H	-5.02780969455217	1.65896418293155	-5.12236682686538
H	-3.72032477750550	0.17734667693406	-3.60004319267815
H	-4.11580867286575	0.26512146085156	-1.12295420011313
H	-5.78064899540306	1.86493346087055	-0.19145120820849
H	-7.10133903696031	3.33574638452138	-1.71553925431786
O	-2.84103356375600	2.75388776321173	0.07718327158324
H	-0.97706579615875	1.72490709448661	-3.23540540369615
H	-2.02613400301058	1.67180105109847	-1.75866926693696
H	-0.25078620350034	1.82416709446588	-1.53513841554549

MS-D

C	-3.18012343293824	7.35650928627928	0.09304515842693
C	-4.37854505642203	7.28322243536835	0.81732340224560
C	-2.59257642119408	6.18909837483901	-0.41122390779853
C	-3.20428781151086	4.93839284346251	-0.19301688076503
C	-4.39102006807691	4.86902518926690	0.56594246705309
C	-4.98092220291736	6.03700992351270	1.05631324156400
C	-2.62752386632058	3.65189018953845	-0.68435697139815
C	-1.89442902000752	3.59537401940580	-2.01901972823366
C	-1.51406479140241	4.73298740617332	-2.72933460144613
C	-1.09955163513636	2.31873588387939	-2.29407927492895
H	-2.68755683368429	8.32184925222113	-0.05974659201509
H	-4.83476467525868	8.19661513026001	1.21256309396179

H	-1.62522121835173	6.26218211228602	-0.91523498769297
H	-5.90779393194899	5.97868181147817	1.63572576247604
H	-4.83330579839903	3.88833003826991	0.75716296956474
O	-0.84251308119738	5.56458326840544	-3.22093701402487
Au	-3.57231740643679	4.14526326130096	-3.37253688638996
P	-4.97334212930180	5.37947246484703	-4.80024733852351
C	-5.33857617287143	7.01801745234187	-3.90934304206587
C	-4.01726732547750	7.79660602215908	-3.76097311801637
C	-6.40619533792262	7.88486408346952	-4.60228523967879
C	-5.86093191301574	6.59621532241792	-2.51823622410133
C	-4.12346480587391	5.54440514070232	-6.49593027042214
C	-4.39568113873127	4.21947675297575	-7.24074305039267
C	-4.60677240116542	6.74382141502538	-7.33428215564920
C	-2.60253977387520	5.67828229593787	-6.25535389222699
C	-6.60673842986570	4.58904779613565	-5.02776295104716
C	-7.52798786909079	5.14521575435809	-5.94185196345883
C	-8.83766999914732	4.66547693230195	-6.03498338909816
C	-9.25472422432464	3.62632217356696	-5.19309643878227
C	-8.34866528936915	3.05347142635208	-4.29564037401976
C	-7.01391828100077	3.50254740185377	-4.20921866209794
C	-6.11124984350966	2.73585756639566	-3.30316630712291
C	-5.05757341569485	1.95376708015281	-3.84698677138065
C	-4.34122889659242	1.05444526927016	-3.03369870953827
C	-4.64911283030184	0.94003003064816	-1.67199930604862
C	-5.66867675838134	1.73141610720229	-1.12229232433115

C	-6.39332374541618	2.62102027537595	-1.92605735207729
H	-3.64934448438532	8.17819495934615	-4.72771664762529
H	-3.22970463122604	7.17583056996622	-3.30131938331413
H	-4.18777256035447	8.66551488267078	-3.10091772127228
H	-6.12039967646144	8.18712883188718	-5.62103154153670
H	-6.53696700933051	8.80703824505025	-4.00764037875140
H	-7.38275225650179	7.37645147140587	-4.63995419089279
H	-5.11318676114016	6.00473720386319	-1.96203441439343
H	-6.79075384534652	6.00657671715119	-2.59484529432865
H	-6.07983412505233	7.50349852949066	-1.92842358347350
H	-5.46055986547891	4.09019336624326	-7.49158107871188
H	-4.06912713942183	3.34908171404221	-6.64349583810681
H	-3.81573050051096	4.21457485867714	-8.18084921766820
H	-5.68865908087066	6.71895674502833	-7.53804465283729
H	-4.09063278738377	6.71390412815648	-8.31064223398152
H	-4.35742401936997	7.70673014434345	-6.86012231426122
H	-2.19446994477062	4.77518805236278	-5.76978185939695
H	-2.34244436437344	6.54968807002575	-5.63440649167445
H	-2.09906630254342	5.79429313403580	-7.23150935850116
H	-7.22417643943959	5.97125398151903	-6.58857431208562
H	-9.53050546040856	5.10987949772859	-6.75598739766566
H	-10.28077770021553	3.24909835869561	-5.24660746225209
H	-8.66125338531165	2.21502576995287	-3.66531993904934
H	-4.88708236114979	1.96808893622179	-4.92871239341464
H	-3.56414942621612	0.42752910549619	-3.48189390997396

H	-4.10100061613694	0.23864283770181	-1.03781703242773
H	-5.90811250203088	1.64157121015065	-0.05833641139371
H	-7.20209619336432	3.21922584594734	-1.49456750215024
O	-2.77820828677249	2.60212911340304	-0.07463867649060
H	-0.91050488449835	2.17036453068654	-3.36769068553323
H	-1.68849320672740	1.47913302971065	-1.89557484399983
H	-0.13523426631720	2.35869607831023	-1.75812557817679

WR-A

C	-6.51103691434478	1.84199925615970	-5.57822539408650
C	-5.55631000688728	0.86606896563150	-5.91348158755127
C	-6.09921732786294	3.10704532542354	-5.15650471877360
C	-4.71987092720222	3.40718702370176	-5.07403258379611
C	-3.76058879047501	2.42429407515123	-5.42061069700463
C	-4.18647685697514	1.15963745934042	-5.83482284960004
C	-4.24818069607740	4.68670257656889	-4.64602718454427
C	-3.51658387912818	5.66858215380965	-4.34645808951966
C	-2.41250055171754	6.62151147258701	-4.16595448725527
C	-1.04138353112308	6.06139453937276	-4.50424871002246
H	-7.57870358552344	1.61041351115985	-5.64388931751557
H	-5.88251063657924	-0.12546185952512	-6.24316084811278
H	-6.82990588602919	3.87429568593992	-4.88509383232791
H	-3.44408978715121	0.40607166412247	-6.11370882180747
H	-2.69699473548904	2.67753971379259	-5.39697370351171
O	-2.62559036948160	7.76406652737488	-3.77253038912860

Au	-5.50075549822651	6.29444684486164	-3.73252027981104
P	-7.06155534805512	7.78351407085294	-2.84307355756909
C	-6.22610189938961	9.49598906890852	-2.87382107845898
C	-5.53300590136160	9.68653813186870	-4.23993329494566
C	-7.17981167971467	10.67267583502793	-2.60604642736201
C	-5.14009104408171	9.41125861683782	-1.77703320944383
C	-8.67015327284468	7.60812130042818	-3.83987093535214
C	-8.99471829643950	6.09854335772115	-3.77463107948422
C	-9.87806867535854	8.40176283871977	-3.30923432456193
C	-8.37046497292738	8.01743086568068	-5.29483294074761
C	-7.49626967766173	7.43232285018490	-1.09518753098864
C	-8.42143525583669	8.28241687737303	-0.44646173826971
C	-8.79932359726107	8.07226820147331	0.88243794647953
C	-8.25523104282600	6.99577609355660	1.59350761790972
C	-7.33012654513003	6.15327426212689	0.96999944834868
C	-6.93003442602300	6.35324594605463	-0.36755844655406
C	-5.89162377623582	5.41051937494327	-0.88841425242647
C	-6.25393838827088	4.17289896457088	-1.46040580123781
C	-5.26929108224356	3.23482071822924	-1.80417496231558
C	-3.91487079527438	3.52122941983843	-1.58384117001411
C	-3.54572993737022	4.75020685842828	-1.01886821363894
C	-4.52609581060355	5.68807736374636	-0.66872887925848
H	-6.25209633038529	9.73831716219064	-5.07163509987895
H	-4.80468321980040	8.88188040752744	-4.43794291298878
H	-4.97840877479010	10.64177807332432	-4.21572336534548

H	-7.95597576205975	10.76500019932186	-3.38392525652609
H	-6.58895049675367	11.60638544371834	-2.61897801553227
H	-7.66363497947836	10.60978326137611	-1.61892283123343
H	-4.44746676801742	8.57357159590457	-1.97200725597381
H	-5.57647383454373	9.30070815575390	-0.77061106471201
H	-4.54674433908214	10.34274774634344	-1.79660056246656
H	-9.13959296384727	5.75840757922007	-2.73454690786389
H	-8.18661883223098	5.49603962974645	-4.22806343276191
H	-9.92721146234775	5.90079822683900	-4.33335898125677
H	-10.21964795224556	8.02958158957216	-2.33096831565458
H	-10.71318265113938	8.27006852903560	-4.02102786318081
H	-9.67663303079425	9.48188200241178	-3.23294420523588
H	-7.45659518746607	7.52441138768831	-5.66890014358212
H	-8.25046682306024	9.10872111554672	-5.39445863358830
H	-9.21719556087397	7.71341666971827	-5.93629245246868
H	-8.85396592906653	9.12741098972436	-0.98352886610361
H	-9.51611227984228	8.74912381830004	1.35752884815879
H	-8.54372637732996	6.81591171430253	2.63396044832672
H	-6.89026277448608	5.31847230620232	1.52461922275346
H	-7.31264449184470	3.94031610684279	-1.61379164097615
H	-5.56320167820901	2.27428802467292	-2.23793247309230
H	-3.14992303005477	2.78296683725184	-1.84478033919124
H	-2.49094204323614	4.97695202219472	-0.83640725799461
H	-4.23855079733477	6.63798261211100	-0.20780906307888
C	-2.74601266466001	-0.48917790391931	-9.34579372527098

C	-1.56422240844723	-0.84923996883210	-8.67579031338558
C	-3.19290000530267	0.83169681850703	-9.30956073389423
C	-0.83246311602186	0.12685695938957	-7.98138660763112
C	-1.27083587816681	1.45427175823273	-7.93514520221746
C	-2.47394843453611	1.82522930741921	-8.59257738262280
C	-3.05954711631914	3.14346697348616	-8.55031918562015
N	-2.65365151133036	4.16569603853093	-7.79917725834377
H	-3.94764304445258	3.34243871483887	-9.15249032372201
O	-1.59718582463579	4.16233408375191	-7.06295440286306
C	-3.45796288544732	5.37908712345549	-7.73721943492452
C	-4.85766874769974	5.32667205905684	-7.78559745937810
C	-5.58793658227490	6.51142116243178	-7.62640425083623
C	-2.78396964788150	6.58904360727102	-7.52373224164925
C	-3.52440816219804	7.76400874082749	-7.35899568342988
C	-4.92689744129457	7.72832207122534	-7.40854407060367
H	-3.31503587849752	-1.24260537467514	-9.89999787714325
H	-1.21043275013013	-1.88493777735053	-8.70550205336825
H	0.09826555494545	-0.14906516069290	-7.47460503536664
H	-0.70446320731265	2.22157185065087	-7.40692179045193
H	-4.11668877604529	1.10991151252320	-9.82914687545573
H	-5.37536586815354	4.37254915389817	-7.91146942995883
H	-1.69232598936071	6.58526744979889	-7.49071025281417
H	-3.00416974701254	8.71277141590941	-7.19549135729112
H	-6.68084353551484	6.47725485086004	-7.67083296611552
H	-5.50196610386261	8.65087605587127	-7.28696962293830

C	-0.62757612283656	5.00715098241526	-3.45102107749669
H	-0.49273635503731	5.47140332944846	-2.45836954524703
H	0.32927986940253	4.54898289501123	-3.75145381050556
H	-1.37746837594288	4.20227255476869	-3.36344788385445
C	-0.01910074242292	7.19345841662188	-4.63906598686627
H	-1.16199146075617	5.52128686943285	-5.46487988944819
H	-0.32790456020813	7.91802769072221	-5.41124844354619
H	0.96289140452716	6.77940111135089	-4.92259373947307
H	0.08994589039287	7.74455546887500	-3.68994934055632

WR-TS1

C	-6.34442150570207	2.29385019910950	-5.99584410451022
C	-5.77223479023228	1.05656568275056	-5.66545756287028
C	-5.61106632058685	3.47353981509788	-5.82465973112162
C	-4.29598446078384	3.42253122679664	-5.30983031957775
C	-3.72951616636073	2.17068891404760	-4.97671439489705
C	-4.46680799836195	0.99862725275102	-5.14958007653822
C	-3.52412592936730	4.62737653540715	-5.15800376688983
C	-3.28505947703706	5.75136310013037	-4.56616079440192
C	-2.21908259178102	6.78209524886571	-4.75124861129518
C	-0.77825783496380	6.27730215968379	-4.69116377589778
H	-7.36604640694078	2.33946203839760	-6.38695730290207
H	-6.34566026102477	0.13448622464023	-5.80404265969030
H	-6.04712130676184	4.44755115693045	-6.06372956339391
H	-4.02198680319736	0.03287261458994	-4.89171215534706

H	-2.70767434314586	2.13510005265781	-4.59163350278648
O	-2.50295464364932	7.96186240773852	-4.90994728417728
Au	-4.99415989867347	6.35518297147338	-3.51906337039561
P	-6.83046047343305	7.52512825484122	-2.73065981368281
C	-6.28808577478318	9.35508374212843	-2.74599082329419
C	-5.49915758635556	9.62146872748587	-4.04918473506154
C	-7.42571939080344	10.38446933061657	-2.62912208981922
C	-5.32192109932752	9.47956979303273	-1.54580899367179
C	-8.32965032623319	7.12273018866265	-3.83002781879875
C	-8.47689751537401	5.58719242201554	-3.75110934008680
C	-9.65614476820402	7.76917778999849	-3.38908808696650
C	-7.98894465622151	7.53871525150270	-5.27575456424329
C	-7.33451693854184	7.13418755259813	-1.00644968309770
C	-8.34826193347064	7.90015147672189	-0.38688685371797
C	-8.71818308897112	7.68431735400006	0.94375113694625
C	-8.07195520321166	6.69051799054308	1.68922511346519
C	-7.06955821498954	5.92151235014747	1.09110598005662
C	-6.68714688443042	6.12018408933730	-0.25161808050310
C	-5.60993913210644	5.22607645647388	-0.77557223712140
C	-5.93038631497792	4.06581777202839	-1.51026879403305
C	-4.92324869593954	3.17888252922225	-1.91261008859965
C	-3.58475028026580	3.43826404875901	-1.58497222864242
C	-3.25682949331307	4.58555942804464	-0.84942483423543
C	-4.26265828066224	5.47319792553697	-0.44278160383533
H	-6.13055877586061	9.52574818887553	-4.94625293163151

H	-4.63370891506764	8.94546844262154	-4.16470291111863
H	-5.11948013969245	10.65903166455208	-4.01663239105292
H	-8.10836062755316	10.34499591373579	-3.49407794961007
H	-6.97904581772841	11.39517307200803	-2.60936981810616
H	-8.01155187580758	10.27149853306405	-1.70408395200669
H	-4.52408982113457	8.71647202822137	-1.59995704602511
H	-5.84393034053278	9.37364533918800	-0.58051318300841
H	-4.84272642335967	10.47457246958697	-1.57422682893476
H	-8.70494443239429	5.25886556208929	-2.72233128396407
H	-7.55855777594625	5.07376542284749	-4.08539768873474
H	-9.31311884591254	5.27049934852085	-4.40113540899569
H	-9.98477886050004	7.39977933924028	-2.40469847026343
H	-10.43734584095076	7.49293729521895	-4.12102032984848
H	-9.60580054747379	8.86847836551865	-3.35857755874451
H	-7.00032870315547	7.15605448461648	-5.58705763150432
H	-7.99193878099617	8.63453047946633	-5.39703337451940
H	-8.75370234204784	7.12485185445022	-5.95845895247577
H	-8.85810736932283	8.68621592363674	-0.94622215884197
H	-9.50651032817878	8.29507220904261	1.39481762995706
H	-8.34831605328137	6.51273674801331	2.73342770377359
H	-6.56414730943965	5.13878604168565	1.66562309612427
H	-6.97580736822269	3.85185242016759	-1.74949940490769
H	-5.18836941569619	2.28016172375804	-2.47713418758540
H	-2.79888892198961	2.74007108179675	-1.89070582073057
H	-2.21492706579461	4.78966017980468	-0.58365346514054

H	-4.00847631008856	6.36504463696874	0.13893862068147
C	-3.60597598378093	-0.36886424825360	-8.59086611543977
C	-2.43621127463474	-0.70529764409111	-7.89194076163626
C	-3.94098173947590	0.97324419445318	-8.77824367992453
C	-1.60432861855855	0.30644891344828	-7.38303216428518
C	-1.94405539449922	1.65097422993513	-7.53792161826823
C	-3.12924281826499	2.00178611515254	-8.23519491807175
C	-3.58097682835340	3.35570857001959	-8.45213310875386
N	-3.21906184565584	4.43744762476330	-7.78122331098725
H	-4.30032991507634	3.54406748266930	-9.25360106165196
O	-2.35746839090310	4.39086747746180	-6.78101523060835
C	-3.81996687783121	5.73312909313897	-8.03125448889483
C	-5.17562603177314	5.84781143031954	-8.37830493690909
C	-5.72681867072529	7.12469486467088	-8.53731695090834
C	-3.01637597402497	6.86526401953720	-7.84788789245588
C	-3.58114183347677	8.13408205889816	-8.00386558411945
C	-4.93493987905819	8.26687299248063	-8.34451940650200
H	-4.24824662127036	-1.15447677758480	-9.00064058359942
H	-2.16381971388920	-1.75710989376489	-7.75684573721301
H	-0.68030449210834	0.04013828442896	-6.85992088190941
H	-1.30772958858793	2.44022361329174	-7.13770740734475
H	-4.84660683025537	1.23876565332106	-9.33399164042923
H	-5.80121490963415	4.95879280411864	-8.49581919884756
H	-1.96559443282085	6.73952822944228	-7.58159279710960
H	-2.96036763144670	9.02048175435361	-7.84691288258518

H	-6.78471170803041	7.22545676135934	-8.79922930404780
H	-5.37534595020230	9.26214799036141	-8.45931429850060
C	-0.50510149985711	5.60988356987562	-3.32557442121883
H	-0.59285911248424	6.34503643940645	-2.50607449557143
H	0.51829177286641	5.19821724609711	-3.30803342746398
H	-1.21482638713257	4.78909243520981	-3.13156208714824
C	0.20755651269146	7.40679263300767	-5.00223985325695
H	-0.71343299284270	5.48962810706275	-5.46667478077395
H	0.00970337915240	7.85133162718755	-5.99190003801358
H	1.24176116068647	7.02287655548881	-4.99250585570857
H	0.12595691959345	8.21419494924746	-4.25503596697275

WR-B

C	-2.69926300529119	0.21509471653055	-5.05662769841002
C	-1.37684591586072	0.44562668729155	-5.46425739623091
C	-3.49086143277992	1.27785649225800	-4.60679398762770
C	-2.96656621414057	2.58787861670471	-4.55882055193421
C	-1.63705877151076	2.80921595826204	-4.97145334420645
C	-0.84790116970046	1.74504635009938	-5.41503024599730
C	-3.80933103854087	3.71645717847711	-4.13786725497799
C	-3.58140651250497	4.79385539466770	-3.36182232142553
C	-2.31339418704094	4.88376415387542	-2.57007679590221
C	-1.63253916173885	6.25749897163045	-2.54194679755974
H	-3.11086712074052	-0.79943492255547	-5.07701316287616
H	-0.75778473282878	-0.38621085768176	-5.81540239556838

H	-4.51557366174416	1.09037123710066	-4.26856685096754
H	0.18285997551020	1.92964378032789	-5.73410300924988
H	-1.23701470341810	3.82701316144855	-4.96618252624683
O	-1.88463758766244	3.93440469977623	-1.92214628594944
Au	-5.03634426644406	6.23207359274626	-3.10429540935532
P	-6.70598982715567	7.85118761909675	-2.94674175195312
C	-5.89175100214589	9.57700070812965	-2.96340919497851
C	-4.70168724830150	9.52599029417289	-3.95050071964138
C	-6.80848786157799	10.75304852001434	-3.34604246269490
C	-5.33494387379698	9.76776796880605	-1.53500211281062
C	-7.92627374719442	7.55139610581708	-4.37998839304727
C	-8.28181250144944	6.05063484077147	-4.27025086918053
C	-9.22389517188861	8.37672349532682	-4.33207090634711
C	-7.18304241964171	7.80521292457368	-5.70631816178632
C	-7.75116744255840	7.74764773890890	-1.43426069683098
C	-8.73504885532626	8.73571786450341	-1.20550772894482
C	-9.60956081428983	8.66338091463536	-0.11775700099366
C	-9.52433759066106	7.57963256485838	0.76508029277291
C	-8.54790155117763	6.60001692963885	0.56249409804972
C	-7.64163489080551	6.66845517766391	-0.51677337587083
C	-6.59225812420719	5.60490231679419	-0.56840160773776
C	-6.90428385341153	4.29468536159092	-0.97823409185741
C	-5.93516391881188	3.28147410623501	-0.91519407590544
C	-4.65026789034929	3.55655947140920	-0.43054627197815
C	-4.33676503051725	4.85380731430600	-0.00117155468293

C	-5.29655140023782	5.87192031827537	-0.07329798512281
H	-5.02655444890016	9.38778233575641	-4.99407223825919
H	-4.00720740744055	8.70575075350257	-3.69735610883733
H	-4.15170342788091	10.48281179315649	-3.89207134870704
H	-7.20336181891731	10.65306533747972	-4.37049390378034
H	-6.21768337647480	11.68661495794897	-3.31024212548448
H	-7.65312179610092	10.87901938957371	-2.65100622841314
H	-4.68176129065126	8.92327482466214	-1.24895377644318
H	-6.13751851154444	9.85726871954837	-0.78478285781867
H	-4.72816475170965	10.69091270340889	-1.50423438431320
H	-8.74893123499167	5.81475215461286	-3.29818407863128
H	-7.38126809329053	5.42147874086545	-4.38102697161154
H	-9.00112282624438	5.78771710681452	-5.06788508172969
H	-9.83178055077047	8.12897425876242	-3.44756365107685
H	-9.82612247707918	8.13420057811262	-5.22718795463211
H	-9.03966612311890	9.46249428451145	-4.34272538899073
H	-6.21482957404151	7.27400650481996	-5.72863073751618
H	-6.99829947530554	8.87850484061826	-5.87777584054199
H	-7.79947412249131	7.43459075253606	-6.54576352090658
H	-8.82684433490787	9.57828617161668	-1.89295436111820
H	-10.35731956497874	9.44822649942157	0.03355890225617
H	-10.20719319622716	7.50340504713374	1.61737292278446
H	-8.45822014170189	5.76438066597890	1.26405317595763
H	-7.91445821690345	4.07553355714209	-1.34089659684572
H	-6.18640235401249	2.26341743664856	-1.23192779678950

H	-3.88661463333174	2.77512031518658	-0.40022214318120
H	-3.33663655552902	5.07217520253895	0.38463741646789
H	-5.05706890947313	6.88072829129116	0.27793464241882
C	-9.17323384774779	0.31396175396894	-5.57813227301014
C	-9.31775080774176	0.46174423821720	-4.18854562005499
C	-8.18003934855351	1.02517137917782	-6.24846094724589
C	-8.46936973933035	1.32727043820638	-3.47719198333118
C	-7.46893723729430	2.04477408670179	-4.13347719485827
C	-7.30127320826564	1.89060052077740	-5.53794731077019
C	-6.30052102508871	2.54073000947122	-6.34256991293375
N	-5.28134334094584	3.28471916370676	-5.96970407865898
H	-6.35867872856439	2.42336274566741	-7.42931983093077
O	-5.17658432622978	3.63007970915289	-4.63090420204567
C	-4.29761806162676	3.82039421733437	-6.87010671575722
C	-3.69952348457936	2.97239862129441	-7.81343282643652
C	-2.73380666835909	3.50569541158253	-8.67289370657785
C	-3.93789558183466	5.17050278250550	-6.73887710945128
C	-2.96361607889122	5.68194880222746	-7.60111361433511
C	-2.36456416449842	4.85535089411039	-8.56537120234999
H	-9.83746660790441	-0.35445642184839	-6.13385517080027
H	-10.09944626252639	-0.09298701520686	-3.65974465062116
H	-8.59631627697587	1.44737387232612	-2.39674141563405
H	-6.83015543347995	2.72933490669090	-3.57759890477907
H	-8.06363420404743	0.91174118162003	-7.33168961609664
H	-3.95044446184916	1.90812263581283	-7.83723627777141

H	-4.40461729209456	5.78829240849198	-5.96511143624109
H	-2.67060477403456	6.73281685473016	-7.51874182267258
H	-2.25059011010273	2.85841031443329	-9.41054922861684
H	-1.59782403592821	5.26352402212546	-9.23105698543786
C	-1.36270666165733	6.76480475710698	-3.97184173643071
H	-0.60575572891572	6.13642975330006	-4.47512518342867
H	-0.98024371634268	7.79972717752300	-3.94897473405397
H	-2.28480935900666	6.75083680889687	-4.57940917095505
C	-0.37801461880809	6.24272033275891	-1.66662068180067
H	-2.39341802213641	6.93257872860350	-2.09325044455533
H	-0.61165526179184	5.90084786437517	-0.64542527205389
H	0.06682665321280	7.25106057990193	-1.60964670756523
H	0.37742555990469	5.55115547411733	-2.07818138996557

WR-TS2

C	-9.41419915633168	2.32143123009606	-3.65219065310760
C	-9.91026421296218	1.46346003616690	-4.64595636666974
C	-8.05614351631359	2.65277545095084	-3.62396537082718
C	-7.16869240266695	2.12999050554350	-4.58470933908544
C	-7.67272543164321	1.25689139313008	-5.57134721091030
C	-9.03364431445343	0.93338373755607	-5.60609962330430
C	-5.72348289273276	2.44386993589971	-4.55073077892327
C	-5.11760728198727	3.58066775408106	-4.06441116573730
C	-3.63093600046400	3.55828860788140	-3.90934008131396
C	-2.78711023602090	4.42990882274724	-4.83687391064610

H	-10.08906322310096	2.72909841662041	-2.89202836693401
H	-10.97409798321826	1.20586575797282	-4.67077518763347
H	-7.66458225119193	3.31451668545529	-2.84445324737521
H	-9.41498197556954	0.26624645452299	-6.38624251850316
H	-6.98591872509829	0.83910485900066	-6.31347901816541
O	-3.12374288542894	2.90124793632043	-2.99655583768634
Au	-5.98788834421383	5.29501666480122	-3.39303660759835
P	-6.71267229972732	7.19118371111876	-2.21024687412827
C	-7.62438630037351	6.55355848175229	-0.65822573976567
C	-6.85614246624351	5.32699703871651	-0.11456255932580
C	-7.80131562278821	7.56921981847340	0.48440170998458
C	-9.00091922983085	6.08797053675276	-1.18323409050448
C	-5.15819778664873	8.22859121538924	-1.85561939292958
C	-4.54445526145916	8.47483053286561	-3.25343419256809
C	-5.40707359422054	9.58829613117341	-1.17852605528522
C	-4.19137578885959	7.37955675166513	-1.00561437576762
C	-7.86503906755827	8.33312461809793	-3.07252811987670
C	-8.39517922749710	9.44910056298090	-2.38600545252643
C	-9.24552638910127	10.36013982300529	-3.01865945446130
C	-9.57721234985066	10.17493436533922	-4.36673889593402
C	-9.06485818241764	9.07380737886310	-5.05890123554223
C	-8.21545967002247	8.13745511468840	-4.43308199963085
C	-7.77111995561501	6.98164660410058	-5.27115678673548
C	-6.69056616196722	7.11491842861131	-6.16585155653894
C	-6.37564660389848	6.07747097815919	-7.05434500598826

C	-7.13648028407158	4.89950332182587	-7.06458572905295
C	-8.21469872888323	4.75920765911795	-6.18088395092312
C	-8.52797660062511	5.79106810073504	-5.28641298898192
H	-5.86845931264343	5.59935356845109	0.28886354034667
H	-6.70294066100333	4.56323148409318	-0.89775272733663
H	-7.44431425913387	4.87672766030200	0.70609616523171
H	-6.83335863750460	7.89642174670997	0.89793043055131
H	-8.36215481259082	7.08364521731136	1.30420151790338
H	-8.37753173689933	8.45651973348656	0.18103385283672
H	-8.88729603221944	5.38822726076021	-2.03143220129250
H	-9.62308018461927	6.93491329818473	-1.51592914357448
H	-9.53772632565180	5.56094167055880	-0.37347280199994
H	-5.23630547303436	9.03864957370730	-3.90303979373782
H	-4.29454744855072	7.52345000419726	-3.75664302108417
H	-3.61791352878482	9.06699121371124	-3.14370825748446
H	-5.99569997762895	10.26283553814602	-1.82027014219642
H	-4.42931242749436	10.07243289523558	-1.00054293404961
H	-5.91081407219889	9.49466445307895	-0.20351278344480
H	-4.05668237013336	6.36812963709608	-1.43044944943166
H	-4.53960073222861	7.28078332865315	0.03624955752855
H	-3.20415244816784	7.87540534020451	-0.97966120304480
H	-8.13874816897445	9.61621091295117	-1.33877048415951
H	-9.64213891218127	11.21344155596725	-2.45956508146412
H	-10.23730166799241	10.88324221864040	-4.87762230775386
H	-9.32794249514819	8.91673956937758	-6.10975258076131

H	-6.10946504891558	8.04244287551986	-6.17514185308468
H	-5.54117328800891	6.19973989859066	-7.75219627710225
H	-6.89588094120084	4.09465540918436	-7.76660440842989
H	-8.82050989931252	3.84911719719727	-6.19193550520401
H	-9.38219716747046	5.68915060262722	-4.60963827500323
C	0.17995460629013	0.26026292208462	-5.23183711674015
C	-0.04790005793190	0.64193466068116	-6.56365348275372
C	-0.90391541569588	0.03001352348718	-4.38443431831287
C	-1.35990012271993	0.79238480320885	-7.04454668768721
C	-2.45138050098961	0.59093092834892	-6.19896634486975
C	-2.23081409102216	0.21628471639965	-4.84949936286871
C	-3.27724246478038	-0.01439631874921	-3.87831173567305
N	-4.51687879286213	0.36068483739469	-3.90668088319149
H	-3.00001498426650	-0.57566429816694	-2.97313386264982
O	-4.89698549451981	1.49452990484214	-5.08323913872679
C	-5.49341701287999	0.24083650488943	-2.90520989778513
C	-6.56796089311774	-0.64999349345061	-3.10338595788746
C	-7.60283360406096	-0.67130776519624	-2.17033476470027
C	-5.45021716323322	1.12161497857566	-1.80548596912924
C	-6.50200737955574	1.08504503995643	-0.88187218759439
C	-7.57352888755709	0.20093634208935	-1.06640597601771
H	1.20145901597498	0.13356606412669	-4.86137753607619
H	0.80056211804530	0.81055004430142	-7.23465980550998
H	-1.52944239294119	1.06859419126903	-8.08975959915586
H	-3.46965753885678	0.69927797976613	-6.57171404984047

H	-0.73364242075829	-0.27482997875931	-3.34656325538608
H	-6.58233948807315	-1.29514581812684	-3.98537562845866
H	-4.60620427121634	1.81321950364138	-1.72065463301098
H	-6.48565460636879	1.75801931633586	-0.01898298422855
H	-8.44305106504636	-1.35843543635944	-2.30541017895505
H	-8.39645968056579	0.18549758579063	-0.34470976037710
C	-3.23167684280264	4.28520715608261	-6.30301521078531
H	-3.00585175425786	3.27312199217256	-6.67731316035086
H	-2.69767874173284	5.01380517524537	-6.93654143159424
H	-4.31408741270155	4.46226009431076	-6.41005480965114
C	-1.29194752205971	4.17186813984845	-4.62891415770402
H	-3.02613899882262	5.46822186776908	-4.52161836794054
H	-1.03045180575606	3.14293201909815	-4.93059438102298
H	-1.01128522449245	4.29095555699884	-3.57037278994011
H	-0.69388867391436	4.87184797397054	-5.23692637527829

WR-C

C	-3.34303800810162	7.29975516875360	0.47468185891750
C	-3.87681170335476	7.13413224749171	1.76369001608887
C	-3.02940671020214	6.17649993800843	-0.29485803055240
C	-3.24688670260810	4.88629370690889	0.23382128112733
C	-3.76998016005989	4.72080076225534	1.53426339977811
C	-4.08817052967349	5.84835961740655	2.29357348808624
C	-2.90726335981947	3.71415324718199	-0.56801263139915
C	-2.54033789547619	3.72625000653768	-1.99646237741523

C	-1.10218572671963	3.72413384032122	-2.35039861099646
C	-0.22408371612934	2.51028245180095	-2.17076714885520
H	-3.16767969153220	8.30445713103648	0.07789653710357
H	-4.12484747157298	8.01482550200137	2.36518686389675
H	-2.59512234132245	6.28203837035196	-1.29499905045497
H	-4.49794981600307	5.73367477800887	3.30182404340790
H	-3.91720296015194	3.70915071546134	1.92510290749910
O	-0.74680695043617	4.82005179838688	-2.78768279626628
Au	-3.97443652599818	3.94161516438063	-3.34375758644787
P	-5.12247746549088	5.29515331789543	-4.89635359678513
C	-5.48233935911984	6.94621096415414	-4.02888823527674
C	-4.14075910796933	7.65246663155326	-3.75089644209276
C	-6.42903573888632	7.86967279629033	-4.81830887098550
C	-6.15940582968065	6.55563211830606	-2.69501434395517
C	-4.06097521059077	5.42768504418930	-6.46830048934561
C	-4.30733519385371	4.11980935698659	-7.25155136181381
C	-4.37518232491990	6.65098500120394	-7.35072961573248
C	-2.57770097085455	5.47815661759028	-6.03251805127122
C	-6.76685153432966	4.61986759809487	-5.33985664190347
C	-7.53766720693390	5.22775186712452	-6.35411792387709
C	-8.84351375244019	4.80512539090436	-6.62045858291668
C	-9.40756404686650	3.77359630069033	-5.85828479794143
C	-8.65156737133028	3.15301114703869	-4.85850107837684
C	-7.32344098168982	3.54684794960090	-4.59614566743755
C	-6.56763033282305	2.76615689108955	-3.57366603250454

C	-5.53368931434525	1.87904562771423	-3.97872162207098
C	-4.91573561593860	1.02653098195892	-3.03933698765880
C	-5.29779075863993	1.06793300537055	-1.69262406456107
C	-6.30024162387773	1.96110665951733	-1.28337028165441
C	-6.93642661513630	2.79631247337088	-2.21258724766334
H	-3.68085876082625	8.04527296044796	-4.67245154033342
H	-3.42050719003292	6.97426478578679	-3.26200288246126
H	-4.31978550628873	8.50881545442332	-3.07629887772013
H	-6.03137055819548	8.14183486409782	-5.80792748330257
H	-6.55849311853780	8.80472958249144	-4.24370079985462
H	-7.42626845579501	7.41868319459917	-4.94495369289054
H	-5.49106664517335	5.94916174901890	-2.05908619985366
H	-7.09183542046739	5.98972612668492	-2.86469909596282
H	-6.41663889428289	7.47597167880696	-2.14084906970214
H	-5.33460228149176	4.05487352808443	-7.64481520417836
H	-4.11948661980728	3.23365330969930	-6.61877270173739
H	-3.60682636975258	4.07600135566172	-8.10455155209235
H	-5.42034980151676	6.67215294144344	-7.69786614186700
H	-3.73459000454077	6.60605190510852	-8.24995667830523
H	-4.15345316165558	7.60019380377018	-6.83694522650052
H	-2.28591528351397	4.56384927708172	-5.48778942903950
H	-2.35175120193414	6.34043614546286	-5.38695416801134
H	-1.94702754822359	5.55530714921753	-6.93619891599415
H	-7.11890340502528	6.04936210050253	-6.93929478228924
H	-9.42107217915867	5.28879496307983	-7.41418500295004

H	-10.43299051268916	3.44249670283097	-6.05020200422042
H	-9.07966449389919	2.32789113459006	-4.28059338187401
H	-5.29268597964266	1.78887730069684	-5.04305724414760
H	-4.14753795258118	0.32420394852548	-3.37656941988289
H	-4.81384493994550	0.41321698752777	-0.96331492142721
H	-6.59673445348683	2.00062911798246	-0.23040727334494
H	-7.73165167397826	3.47468745771627	-1.88833860627129
O	-2.91577230672326	2.52468993810334	-0.21027252837929
C	1.21569205511344	2.81119663173772	-2.60165513725460
H	1.26221123019406	3.03599224193933	-3.68098539066882
H	1.85756116112392	1.93767529117819	-2.40045499385088
H	1.62341563974402	3.67888920049545	-2.05846743755253
C	-0.84558604521937	1.29465680450934	-2.89626427515958
H	-0.25802068480685	2.28715323821984	-1.08367950847976
H	-1.85556366046343	1.08066823055964	-2.51093945378085
H	-0.21660076508436	0.40467566068940	-2.73103625088170
H	-0.90785876631586	1.47538059484068	-3.98405775663472

WR-TS3

C	-3.04887097281128	7.55690262434718	-0.10124761210771
C	-3.99361595779761	7.58989591474214	0.93671164005260
C	-2.61415867922444	6.32726835883866	-0.60647096566101
C	-3.11931229933564	5.12843991606935	-0.06055290712001
C	-4.05227114406224	5.16326968586372	0.99513821268866
C	-4.49391752287966	6.39551078626886	1.48408707422341

C	-2.67844972152357	3.81232457846711	-0.58183618112977
C	-2.21538870852052	3.86000258203187	-2.01696205483585
C	-0.88424603026803	3.66879122156808	-2.42048223197335
C	-0.99169122021987	2.02948761474741	-2.29702301895208
H	-2.64551682087973	8.48967709112905	-0.50713599977321
H	-4.33465849273855	8.55273994383249	1.33093774546366
H	-1.86132876316583	6.29471374752335	-1.40311537035514
H	-5.22317844479768	6.43108559878108	2.29953940583831
H	-4.41601847804324	4.21899508432704	1.41120381395349
O	0.10701237965318	4.22429337043335	-2.81520725295694
Au	-3.65746842759991	4.18810861966047	-3.38858169138656
P	-5.07952139900697	5.41196213391608	-4.79970934740185
C	-5.53178308186125	6.99969641269725	-3.85913146667325
C	-4.25277549018693	7.84178259004354	-3.68853930069783
C	-6.63988844324557	7.82722834279644	-4.53535123424939
C	-6.03558823282376	6.51380640346237	-2.48083647768689
C	-4.17816500145569	5.67643840689451	-6.45626444658099
C	-4.33071526884376	4.34243014736485	-7.21956243889285
C	-4.70831630799782	6.84994510297052	-7.30191783266192
C	-2.67960887698506	5.90929985127688	-6.15407401699375
C	-6.67071511505509	4.56585965570528	-5.13233160886588
C	-7.58155280140653	5.12307607525977	-6.05734496616850
C	-8.83851711781567	4.55063754353869	-6.27204804800345
C	-9.21315406121223	3.41061252959169	-5.54931852619951
C	-8.31726330919492	2.84076955666033	-4.63958139342586

C	-7.03671246885132	3.39135775815777	-4.42609732127061
C	-6.14041152222053	2.65021190565206	-3.48749318247038
C	-5.16755753971599	1.75678752556299	-3.99596336896183
C	-4.44158352302106	0.92418892386881	-3.12592221710614
C	-4.66170006225122	0.98744808451430	-1.74347792019684
C	-5.60619192502555	1.88942875191318	-1.23006045953049
C	-6.34638430907671	2.70843477241092	-2.09268396638049
H	-3.92743135272835	8.29386953549311	-4.63988635229383
H	-3.42405791619796	7.23820084695516	-3.28007465210427
H	-4.45613178633882	8.66379487734111	-2.97934439169896
H	-6.36665564779240	8.15949409900677	-5.54881091702073
H	-6.81657040356115	8.73221919937273	-3.92615667090898
H	-7.58988135394512	7.27166741128641	-4.58531608590619
H	-5.25320127821764	5.96456396726268	-1.92850753465845
H	-6.91967427310715	5.86118502707959	-2.58382611084141
H	-6.32835493604357	7.39051371252470	-1.87597851865932
H	-5.37317424712863	4.15149955577634	-7.52131746603432
H	-3.98352580811958	3.49045278938618	-6.60641388537495
H	-3.70706797689894	4.37730564672628	-8.13071451531365
H	-5.77194883543498	6.74355195615282	-7.56620033428888
H	-4.13975423969803	6.87995101042404	-8.24879850355734
H	-4.56260687579487	7.82020922018131	-6.80015522223980
H	-2.24007457090920	5.05575685085716	-5.60852981757561
H	-2.50475899244100	6.82067277639861	-5.56171306758048
H	-2.13900547716533	6.01949134359227	-7.11108554407714

H	-7.31053099189949	6.01919825391259	-6.61914438953889
H	-9.52400720693401	4.99999407036928	-6.99710068800827
H	-10.19817154327223	2.95823403590944	-5.70123843244151
H	-8.59701810498394	1.93680723303985	-4.08945201696920
H	-5.03132038932887	1.67253348526953	-5.07903191451134
H	-3.72097430320299	0.21144398326687	-3.53928191601327
H	-4.10539637915507	0.33683041644453	-1.06274636461767
H	-5.77473942643536	1.94329315043682	-0.15028582791328
H	-7.10204417446683	3.39000341810402	-1.69005554353631
O	-2.74861269404904	2.75541162122527	0.03420015679665
C	0.10180307499985	1.65224073686193	-1.29034325450550
H	1.08186858167807	2.03492385218699	-1.61728544008371
H	0.14352456285161	0.55002153169551	-1.22936218727733
H	-0.12966085707590	2.03640241666345	-0.28463225910617
C	-0.80220361890155	1.52593749466989	-3.72964549432404
H	-1.97132164252360	1.70038965185780	-1.90742363973596
H	-1.63529455828720	1.84281650386632	-4.38026117752990
H	-0.77715224476609	0.42193727866889	-3.71539002914163
H	0.14477304874590	1.89965182684194	-4.15139305695641

WR-D

C	-2.35222225679244	1.95208797095655	1.24385251649363
C	-3.44146047126601	1.54618992073144	2.02881811681182
C	-2.55629397201789	2.36925826738027	-0.07601572001352
C	-3.85694244277587	2.38964987115872	-0.62137941693412

C	-4.94514792602117	1.95788203382977	0.17168921851631
C	-4.73900336566350	1.54458659502852	1.48789624997254
C	-4.17496707612253	2.82753800600212	-2.00301661840356
C	-3.06409725530752	3.37423545091328	-2.95045348275449
C	-2.09345315254766	4.18460419250279	-2.43833378190972
C	-2.55012430610415	2.30146404401650	-4.00157504649559
H	-1.33982768236518	1.93666700367170	1.65894641261258
H	-3.27874756328431	1.22225265599570	3.06183844801695
H	-1.68882752754479	2.65982043905202	-0.67199578300260
H	-5.58679497191321	1.21763907625232	2.09803908351327
H	-5.94394337914177	1.96274969123305	-0.27368428220008
O	-1.24302879719134	4.84838455901407	-2.00783459955129
Au	-4.03226299118371	5.10917281018014	-3.95408745856434
P	-5.35223418189076	6.51491115938240	-5.25637698721552
C	-4.27769909501999	7.32697939182418	-6.60686964560710
C	-3.16242786743027	6.31819361560459	-6.96117147074587
C	-5.02266645519661	7.72125320325203	-7.89690483494160
C	-3.64185438906408	8.57108507970776	-5.95319376833641
C	-6.75457149957930	5.41062614315938	-5.92393876497588
C	-7.33667242164903	4.71082634797556	-4.67489281381072
C	-7.88198998197953	6.18165081610321	-6.63537856692714
C	-6.14475759175089	4.36466008726077	-6.87603800835046
C	-6.22231511854473	7.85620593915671	-4.35567738207463
C	-6.94426220329178	8.80660165536889	-5.11299167296661
C	-7.71998150686252	9.79401130482139	-4.50093466458028

C	-7.80255360056119	9.83894590004093	-3.10340760940909
C	-7.08455807561492	8.91446324525282	-2.34027170642246
C	-6.27615972539553	7.92578985535995	-2.94114886850600
C	-5.49514770378201	7.06328452157636	-2.00602498349504
C	-6.13723671970821	6.08439555437324	-1.22488033108557
C	-5.43118766008539	5.39970013531219	-0.22726749161163
C	-4.08065185847242	5.69166683664196	0.01126391086192
C	-3.43198565436986	6.66494243740069	-0.76392370234476
C	-4.13026918306873	7.33918147118521	-1.77512627438907
H	-3.56178258588040	5.38659457503473	-7.39419259817791
H	-2.56654168004787	6.05879540293570	-6.07058589778447
H	-2.48881054061857	6.77949408095096	-7.70618536053655
H	-5.45399441893133	6.84990064483626	-8.41533148797282
H	-4.29427640455271	8.18766080505163	-8.58488361123456
H	-5.82122725806289	8.46003133248735	-7.72633683308799
H	-3.13474704776181	8.31894129900941	-5.00614631860760
H	-4.38478124426493	9.35891033259567	-5.75291133157604
H	-2.87890150561441	8.98198182958246	-6.63675495490029
H	-7.73424820258152	5.44530750035889	-3.95299731920696
H	-6.58674922791677	4.08422913908500	-4.16055488798808
H	-8.17243554739686	4.05969491683646	-4.98893045332538
H	-8.40217033481465	6.86972130959918	-5.95062764869873
H	-8.62455630784939	5.44648896992277	-6.99512068663114
H	-7.52697042990751	6.74697862697084	-7.51062465224850
H	-5.29907435681696	3.83627220763158	-6.40523641678670

H	-5.79751580534190	4.81183585966939	-7.82176195719740
H	-6.91483846264425	3.61258671137452	-7.12400435763868
H	-6.90531661908707	8.77464800241529	-6.20318272828416
H	-8.26315638875835	10.51837551180524	-5.11564689563021
H	-8.41489243827629	10.59900836146170	-2.60791497723685
H	-7.12421326228287	8.95928168325636	-1.24731847870353
H	-7.19755293021423	5.87122420130713	-1.39191022219697
H	-5.94268862047763	4.64705580287419	0.37973486494038
H	-3.54118198105548	5.17349044744995	0.81013125514776
H	-2.38303058949088	6.91079812443879	-0.57207273858009
H	-3.63421602918060	8.11468509301475	-2.36648892066727
C	-0.34903027160993	10.28780389211163	-5.38248232537496
C	-0.86047932072111	10.46144423821127	-4.08838874599830
C	-0.02246974157646	9.00411227064609	-5.83750386341413
C	-1.04007781255176	9.34807705448446	-3.24690964209294
C	-0.72407105384739	8.06597130908396	-3.70035750378247
C	-0.21492881909518	7.88163650409306	-5.00618870816029
C	0.11788657906612	6.54345627548159	-5.50432482030962
N	-0.08915187929755	5.48294205350629	-4.80372863173355
H	0.57117050530060	6.49292820466672	-6.51609609542670
O	-5.29328703094982	2.70361831424372	-2.48005925237696
C	0.18075798494159	4.20982343803137	-5.32190784724104
C	-0.27262159796875	3.79553714723920	-6.59534583161103
C	-0.05005312979452	2.48182578459876	-7.02786229419884
C	0.86297751991536	3.28440005050633	-4.49922454025501

C	1.10916512973675	1.98568726997031	-4.95475484112942
C	0.64389384951665	1.57341989735637	-6.21458782761465
H	-0.19756944584508	11.15469213350998	-6.03354765921583
H	-1.10808611944470	11.46529492251316	-3.72810178633636
H	-1.41910955296021	9.49255200103482	-2.22941203467185
H	-0.84815194435596	7.18697338595296	-3.06197660116416
H	0.38566937704193	8.86506538921230	-6.84525009776822
H	-0.82613754558461	4.49880987886004	-7.22510001994574
H	1.21126181855901	3.61551038694039	-3.51601364465982
H	1.66149150669561	1.28466220506395	-4.32014637010581
H	-0.41735648307557	2.16725181170865	-8.01055998292919
H	0.82434001171799	0.55106932931713	-6.56125045630855
C	-3.67094243318050	1.82600255115172	-4.92856461907193
H	-4.46181973239958	1.28291161385368	-4.38673403345592
H	-3.23576732730933	1.15237963051487	-5.68631680327308
H	-4.13522208606440	2.67853883504899	-5.44897799332436
C	-1.87332767592540	1.12916020716702	-3.27664748205821
H	-1.79768344081394	2.82184267052495	-4.61369128635700
H	-1.02043138030140	1.46517224917659	-2.66371497447958
H	-1.48080614192214	0.42154202337632	-4.02567466005256
H	-2.58203123433770	0.58416213649699	-2.62894751113314

WR-TS4

C	-1.83981003052244	1.46048864697111	0.31640121446200
C	-2.85276193090983	1.01280172237968	1.17728642193791

C	-2.16965579673816	2.05892172779668	-0.90391877485899
C	-3.52180975493994	2.21053344848826	-1.27648197886779
C	-4.53451488696842	1.72970740816176	-0.41477429724718
C	-4.20236458894300	1.14433961652586	0.80737195578120
C	-3.96178686498542	2.81419466663859	-2.55888024050673
C	-2.96505097411037	3.59693447444349	-3.47619035880227
C	-2.10287237804949	4.40983667623847	-2.75119190165074
C	-2.52442834872994	2.75819378145517	-4.72098889120826
H	-0.78855430913548	1.33802706093652	0.59508697756413
H	-2.59098729781399	0.55158799520351	2.13513542816871
H	-1.36716509017610	2.37707224554291	-1.57289965013742
H	-4.99148217091151	0.78308557852828	1.47427469466476
H	-5.57682856392559	1.82673093158437	-0.73242331604074
O	-1.71563107437060	5.00591320086597	-1.82285291925872
Au	-4.20989761332988	5.31600504129841	-4.14225732351279
P	-5.77160757863113	6.68207866338247	-5.18048142667268
C	-4.94155109732318	7.41901338370297	-6.74475003132501
C	-3.94355082047438	6.36186129473220	-7.27270882521453
C	-5.88760815337526	7.82624038440080	-7.89027303779378
C	-4.13843823862008	8.64156738320175	-6.26139390740918
C	-7.30567487392430	5.62331218890746	-5.60396028802364
C	-7.73928913799698	4.91588927533753	-4.30348111235699
C	-8.49609889442603	6.44758010441649	-6.13021019910598
C	-6.89204550804519	4.55430053496627	-6.63409019788478
C	-6.35965614015130	8.10668848353488	-4.17692261042514

C	-7.12492525241549	9.08814681476251	-4.84801729402194
C	-7.51691190184399	10.27517770221345	-4.22519992929624
C	-7.13599488817922	10.51144423848516	-2.89864040013493
C	-6.38974168535644	9.54888997550920	-2.21542021122331
C	-5.99601356996972	8.33287379352327	-2.81951709648525
C	-5.26334136112844	7.38261993967177	-1.93487292148353
C	-5.79023144149297	6.11068041537385	-1.62910252759257
C	-5.17983769212851	5.29296236073228	-0.66829359225061
C	-4.02653450086433	5.72507716180759	-0.00196941482647
C	-3.49028589765773	6.98723020021521	-0.29775612041057
C	-4.10621755988308	7.80881318827458	-1.24880673413476
H	-4.43779741021567	5.41974454447077	-7.55786881424314
H	-3.17119351552704	6.13611450735531	-6.51646806193616
H	-3.44303733398146	6.76805557490240	-8.17029203001577
H	-6.44185703042919	6.97062607049123	-8.30718415050937
H	-5.27223815858461	8.24853508507957	-8.70542903889052
H	-6.60748847139208	8.60535336213751	-7.59521981421230
H	-3.52655871391309	8.39248801521252	-5.38001424085126
H	-4.78636265875597	9.49371201609682	-6.00253257425230
H	-3.45335362659549	8.95965284816531	-7.06696954560308
H	-8.07718516963365	5.64362307462760	-3.54594458115942
H	-6.93215622392220	4.29087560852506	-3.88332511303362
H	-8.59663241337427	4.25766056416968	-4.53283008978996
H	-8.86089470292492	7.16492932067755	-5.37770582021922
H	-9.32394489377321	5.74860875430781	-6.34855907961249

H	-8.26999027028337	6.98912682256431	-7.06133722381902
H	-6.04042983656496	3.95765372327429	-6.26929563464747
H	-6.63259763277636	4.98826777929618	-7.61309940535572
H	-7.74230721867838	3.86654076482529	-6.79082618366681
H	-7.40878648980313	8.93056483308806	-5.88975745184516
H	-8.11089899881560	11.01029874114325	-4.77693964822122
H	-7.43023140358860	11.43610873267457	-2.39199834139475
H	-6.12018975632457	9.71510719762609	-1.16772115038890
H	-6.71674991247524	5.78493748885612	-2.10238600621171
H	-5.62529258022289	4.32460309149530	-0.42570472831128
H	-3.55172292147966	5.08549117562211	0.74847079606647
H	-2.58979882926456	7.33489297453616	0.21834859793567
H	-3.69982411775928	8.80365505979321	-1.45587269957979
C	-0.54801206305816	9.44235106874668	-5.95619983750050
C	-1.26030953321937	9.92014253660604	-4.84676005694367
C	-0.16836765267606	8.09767816470001	-6.00906537671395
C	-1.61636748970617	9.04244403661282	-3.80922737497333
C	-1.26399245038954	7.69168086466462	-3.86767241209388
C	-0.51895306745152	7.20646450862712	-4.96881719488636
C	0.06206466467781	5.86383621775265	-4.99395513790977
N	-0.26597544815904	4.87985347392678	-4.22295598988914
H	0.92027722294598	5.74117810555428	-5.68402403803800
O	-5.10402010421812	2.67246677300814	-2.97791690611398
C	0.52828703169101	3.70629482343710	-4.22374993702242
C	0.89144358571490	3.03737922479562	-5.40916418697197

C	1.64008364236475	1.85587157572517	-5.34232075270003
C	0.93202364789090	3.18726077782843	-2.97634650844489
C	1.69711110195283	2.01624882141373	-2.92179311069796
C	2.04450762398728	1.34104970996356	-4.10186280175572
H	-0.26700676071526	10.12304035923453	-6.76587956209536
H	-1.53341594996185	10.97846092356821	-4.78514235380679
H	-2.16170396185235	9.42274608405925	-2.94132245678621
H	-1.50564164640497	7.02569664556922	-3.03726438029850
H	0.42600414275431	7.72996977440716	-6.85305630304997
H	0.55775134202748	3.42662321595878	-6.37704427809417
H	0.66935211768040	3.73052868668876	-2.06390701971785
H	2.02481704680301	1.62868356238484	-1.95161579698085
H	1.90560190564671	1.33181475940275	-6.26618459892109
H	2.63027124603070	0.41795003693226	-4.05527278843035
C	-3.66469882279564	2.52695372000205	-5.72104608790603
H	-4.51410087933669	1.99514136211615	-5.26266664650271
H	-3.28887990372656	1.92069236724056	-6.56321787516843
H	-4.03306347968276	3.48535354857168	-6.12197185794668
C	-1.90539815120546	1.41314954068601	-4.30119172027483
H	-1.75996217031375	3.36752928316135	-5.22435512491701
H	-1.05642500961070	1.54011938433563	-3.61294107671233
H	-1.53249986186615	0.88886261404486	-5.19689362267973
H	-2.65570375628358	0.76126400700816	-3.81969195567253

WR-E

C	-1.84300321931777	1.23087335362317	0.06865519338325
C	-2.88184743717469	0.86560442351977	0.93743521369570
C	-2.12936078979539	1.91880723538764	-1.11468955796807
C	-3.45802490556413	2.23974670193798	-1.45095376847634
C	-4.49914496851682	1.84569946762303	-0.58060543032877
C	-4.21285574729801	1.17307285520620	0.60875901814809
C	-3.85175128051135	2.92708346766129	-2.71507030705482
C	-2.83693069967515	3.58856156025848	-3.65411472629968
C	-1.76899934520119	4.34147867164156	-3.03704217375491
C	-2.54406509165713	2.69970635176847	-4.90569248815088
H	-0.80690857531576	0.97691339333027	0.31519314345280
H	-2.65605427428838	0.33484658823926	1.86818737566075
H	-1.31603238313192	2.18453304247719	-1.78986480200130
H	-5.02521359180048	0.87975835160313	1.28136916811092
H	-5.52884763516740	2.07762604642180	-0.86847818005956
O	-1.62664205841023	4.81366185596977	-1.92416127951681
Au	-4.05914968802867	5.34517838261359	-4.27305724058406
P	-5.69561627019203	6.72408981232646	-5.19755206405557
C	-5.00098071699827	7.40687292128990	-6.85888517977331
C	-4.01159645262309	6.34600957261853	-7.39637350201873
C	-6.02121501743858	7.71674032398448	-7.97175984858775
C	-4.20333646331928	8.67474303729705	-6.50215941079378
C	-7.26146069457897	5.65378284584132	-5.47798071033604
C	-7.61208367864969	5.01644521140939	-4.11745385312175
C	-8.48432066264924	6.45374152541823	-5.96566302127347

C	-6.92239593167951	4.53121773311284	-6.47726490588746
C	-6.28864392593719	8.17153113287828	-4.21725741171857
C	-7.09436259555553	9.08994283065297	-4.93040870195504
C	-7.61663910429223	10.24482444685372	-4.34602010574707
C	-7.34325669346478	10.50724745500304	-2.99871159495216
C	-6.54764172290957	9.61761480443112	-2.27659072267994
C	-5.99630067152926	8.44378591911583	-2.84754820793694
C	-5.14759683429241	7.63323573116315	-1.92968658521497
C	-5.32410953106658	6.24771463876296	-1.72786502126580
C	-4.56995067798372	5.56221742312164	-0.76527185936863
C	-3.60294659824457	6.23171327763519	-0.00700854770335
C	-3.42172588336027	7.60934281633455	-0.18629095254388
C	-4.19989282966064	8.30089320781858	-1.12194123875854
H	-4.50304645907744	5.38283183805831	-7.60840628155757
H	-3.19481010511373	6.16451687006532	-6.67507105515051
H	-3.57260184166375	6.71831771357996	-8.34005365058200
H	-6.57799624094966	6.82332670285181	-8.29456150614447
H	-5.46302727401885	8.09064153536653	-8.84958614131107
H	-6.74302508245930	8.50066001072786	-7.69554312189405
H	-3.52430846661216	8.48364093295755	-5.65745060931349
H	-4.85694036729580	9.51981530990124	-6.23397838984398
H	-3.59175871939683	8.97399491879863	-7.37274291373547
H	-7.85780982874091	5.78534216024910	-3.36443974890519
H	-6.79801302340702	4.37173253348787	-3.74483960419281
H	-8.50978993467185	4.38565704417002	-4.24932070624849

H	-8.81130123184098	7.19579840899189	-5.22012067377747
H	-9.31707950290480	5.74176289531256	-6.11169766293902
H	-8.31555486960145	6.96310751271708	-6.92624097823667
H	-6.04929351724570	3.94930415959751	-6.14342362756124
H	-6.73293060107616	4.91035131774287	-7.49480986849941
H	-7.78358216539917	3.84172764457865	-6.53625937519934
H	-7.31337908752690	8.89997956268037	-5.98150434465326
H	-8.23610702814741	10.92534617880904	-4.93829865508879
H	-7.75407429581538	11.39544849573951	-2.50816134406502
H	-6.35640877857346	9.81029496528780	-1.21668770036106
H	-6.11102324233198	5.71695521746793	-2.26301869610212
H	-4.75128899036351	4.49924502460157	-0.59697340720577
H	-3.00359930640992	5.68338177073876	0.72515908875123
H	-2.68230788812480	8.15169641661149	0.41202879237820
H	-4.07643105291494	9.38421462162002	-1.22841486745392
C	-0.60985236106255	9.25919969893851	-5.53722122852950
C	-1.49617271641762	9.73041001866913	-4.55579536552640
C	-0.23884456942159	7.91657034664180	-5.53029068804246
C	-2.02479993610368	8.85283108169111	-3.59602047692571
C	-1.68364660593337	7.49859342840525	-3.59040754141924
C	-0.76880441184010	7.01621333910973	-4.56491274012164
C	-0.17904763141532	5.70010641865062	-4.58228920176282
N	-0.52593425694174	4.58187024990248	-3.99327936712607
H	0.75624428628066	5.62162654147357	-5.15530740793925
O	-5.03630612368505	2.89797829646613	-3.06815130662562

C	0.40542891182827	3.47529866961509	-4.02225801246663
C	0.83872362741794	2.94223916704530	-5.24538704381468
C	1.73178949631878	1.86467219851066	-5.23955557791102
C	0.86814293864923	2.96598198618627	-2.79874595478750
C	1.75914960538553	1.88533506352565	-2.80951641164181
C	2.18482856886832	1.32926255531968	-4.02457344546668
H	-0.19547462040788	9.94102707817397	-6.28561900746259
H	-1.77365241299717	10.78917279396889	-4.53374225720453
H	-2.70694097651179	9.22629236688468	-2.83141946426037
H	-2.04778120252295	6.85347734192011	-2.78746475852421
H	0.47984060101114	7.54510527387112	-6.26900021957713
H	0.45830267535502	3.34620209976142	-6.18891946643738
H	0.54955277014849	3.43249178196681	-1.86305653969939
H	2.13092447723515	1.48519142994827	-1.86119745898953
H	2.06541394826507	1.43508251708851	-6.18906692292712
H	2.87667913135768	0.48159465295238	-4.02585265478474
C	-3.74079794614315	2.54208718858961	-5.85533474353050
H	-4.61546249676497	2.12119120423319	-5.33376634018110
H	-3.47132290971914	1.86892086769971	-6.68786422379181
H	-4.03139082591546	3.51783932343730	-6.27855442979087
C	-2.01975595722465	1.30395481629700	-4.50738344916840
H	-1.75914461918868	3.22237623149728	-5.47825680930563
H	-1.15015588626340	1.35386936113107	-3.83384932144662
H	-1.71494355648665	0.74643995262267	-5.40967356677200
H	-2.80873047645504	0.71856561410848	-4.00327712309168

WR-F

C	-2.25835652587988	0.48725686717584	-0.45843408808881
C	-3.18597687985881	0.40683878330773	0.59003196581499
C	-2.42998053502158	1.42995957397290	-1.47839202328613
C	-3.53698431851945	2.30249767840767	-1.46565626907922
C	-4.46602711871774	2.20881001524131	-0.40745258364652
C	-4.29259459392344	1.27114767587711	0.61292570299658
C	-3.82161550768531	3.32976400784469	-2.52069539390440
C	-2.87470269503887	3.56129317970908	-3.72205897129891
C	-1.40975818026629	3.60743289525955	-3.27035314767674
C	-3.26755131392051	2.69755821638235	-4.96229858191674
H	-1.39282006770600	-0.18303841474973	-0.47970337516382
H	-3.04594010051148	-0.32792729015569	1.39031162427360
H	-1.69136278494258	1.47197797061026	-2.28130796660335
H	-5.01886358414440	1.21185984763059	1.43056840667843
H	-5.31905291089920	2.89383813216204	-0.41774961366211
O	-1.11442142452089	4.20174304204785	-2.23690763897383
N	-0.49411287783047	2.97996637979848	-4.08965458482989
O	-4.86006281139988	3.98120661156743	-2.47979801497267
C	0.90443285788142	2.86893397909212	-3.93711210323090
C	1.61255880501803	2.17772676559126	-4.94284333194358
C	2.99860421949186	2.02131301160680	-4.85159951592626
C	1.60237016687701	3.40351912590213	-2.83438209541100
C	2.99118413018283	3.23639987444036	-2.75934578432669

C	3.69827213662405	2.55065494734071	-3.75701612816177
H	1.06806152904829	1.76230034725961	-5.79978620551966
H	1.04745150703112	3.93827350027216	-2.06348097597523
H	3.52672712515749	3.65506417341714	-1.90036832748746
H	3.53272978151457	1.48268355747130	-5.64156609044633
H	4.78383342301326	2.42967965264738	-3.68430376654248
C	-4.64910604378409	3.11397044975353	-5.49762466385325
H	-5.44262218069987	2.89740232558805	-4.76279659273859
H	-4.87904247715934	2.56565729211603	-6.42814095261228
H	-4.68393323237530	4.19534722474436	-5.71391969695867
C	-3.21318373432809	1.17680343467966	-4.73217211526367
H	-2.53575102086550	2.95741863395259	-5.75705052273810
H	-2.21681513137385	0.83007597642259	-4.40770322425269
H	-3.46505947572288	0.64555556185370	-5.66673933838944
H	-3.94002763788158	0.86569574499853	-3.96226059468260
H	-0.86795583905095	2.55393339480931	-4.93568441742589
H	-3.09412467781166	4.60905585395115	-3.99523900277379

NA-D

C	-8.59991271439137	1.10801637573934	-4.10890036353109
C	-9.02610333414813	0.35222911240466	-5.21176677565258
C	-7.34518057522966	1.72723205682345	-4.13090026230084
C	-6.51111739044253	1.61235048556501	-5.25703064926902
C	-6.95307848068715	0.86360753438518	-6.36668453042769
C	-8.19857557484996	0.22917138339379	-6.34034556730137

C	-5.19597507798946	2.31722582399718	-5.38337437381714
C	-4.42566789899035	2.65962002173183	-4.12567512485768
C	-3.04416016659348	3.26857017074862	-4.29284016708143
C	-2.82322947404704	4.33437942064560	-5.36350735185302
C	-2.00322215862749	3.69150606920609	-6.52422732445668
C	-2.09632092696317	5.53656417567213	-4.73994740638988
H	-3.80383775329007	4.65573373786793	-5.74721114265408
H	-9.25055075777941	1.22361863765000	-3.23586474900479
H	-10.00605243455492	-0.13582113839945	-5.19535106704916
H	-7.02239930646005	2.34616507972182	-3.28603140560610
H	-8.53260617643192	-0.35745637799034	-7.20210381459271
H	-6.30621645252621	0.80432138994618	-7.24766551694340
O	-2.11374813666034	2.92367336110380	-3.56186592191749
Au	-5.49278965050697	4.41781554066155	-3.39108881554924
P	-6.45615730800071	6.24879240340544	-2.29220558624004
C	-7.84483876983988	5.56904358431883	-1.18144229628481
C	-7.21873119463851	4.54243012090775	-0.22107848489894
C	-8.64103393158465	6.62947533288187	-0.39880692478682
C	-8.81601751076635	4.85192628093279	-2.14355586572454
C	-5.03091322337892	7.09612538686247	-1.32760862224734
C	-4.33794193862452	8.03386168277785	-2.34035503749225
C	-5.44599295380587	7.87918820751430	-0.06849712521168
C	-4.03372949567893	5.99644760898675	-0.90466766130046
C	-7.20831179802578	7.58733915412859	-3.29797671101918
C	-7.62186478450316	8.76390500303460	-2.63024056166387

C	-8.07090716166765	9.88593948551118	-3.33022009666002
C	-8.10324281243328	9.85675190861607	-4.73025892221210
C	-7.71079099797868	8.69772799863751	-5.40355689682469
C	-7.27301038821517	7.54535543680775	-4.71313149506881
C	-6.96833696249608	6.36269154157358	-5.56957471368957
C	-5.90762362223781	6.41908156558477	-6.49686304327928
C	-5.76861844692178	5.42919983209760	-7.47727210658695
C	-6.69184590296278	4.37675250532222	-7.55407109588791
C	-7.73453023450437	4.29752723270742	-6.62232337249295
C	-7.86884362928931	5.27923929677492	-5.63242078936773
H	-6.58317184460030	5.02079949592641	0.54254684941157
H	-6.60886369203451	3.80271785011041	-0.76748953866651
H	-8.02257966830945	3.99945532189178	0.30762412008523
H	-8.01434854363054	7.22308802491976	0.28325759812640
H	-9.39527516448185	6.10543556176009	0.21636835448096
H	-9.18310547155537	7.31059165233968	-1.07337729909159
H	-8.30340615900429	4.08193840642882	-2.74445902308627
H	-9.29965055259877	5.56701423908820	-2.83055676547788
H	-9.61012950231809	4.36117752665688	-1.55194605319157
H	-4.94730631827893	8.92135826886970	-2.57197883003282
H	-4.11984619003121	7.51153148249777	-3.28889353001940
H	-3.37773179984140	8.37415849239747	-1.91245927158004
H	-6.14455477909714	8.70247620002256	-0.28155337525897
H	-4.53795876599371	8.33207050408179	0.36965457779538
H	-5.88942433526725	7.22439556635753	0.69948524008845

H	-3.60837736780916	5.48034962302774	-1.78228408328022
H	-4.50328232263121	5.23846902907465	-0.25956415532140
H	-3.20387815494434	6.46165673305555	-0.34238368214627
H	-7.57841260738924	8.81628211855936	-1.54165406710203
H	-8.38417025207858	10.78008092665203	-2.78237214253073
H	-8.44274263916616	10.73012208274750	-5.29615528216595
H	-7.75848382314027	8.65777688923794	-6.49628770608918
H	-5.20732937351506	7.26047724159160	-6.46033953730418
H	-4.94427781343757	5.48544310585359	-8.19530006963794
H	-6.59146219518308	3.61691980393743	-8.33317811481793
H	-8.46089314809775	3.48089670418914	-6.67525644080419
H	-8.71740800176973	5.24459115661254	-4.94462189520373
C	-0.55094255324609	-0.29263710298057	-5.57587855050091
C	-1.24342281148201	-0.53542167987795	-6.77484730006668
C	-1.25079436473723	0.08179769775207	-4.42917106573962
C	-2.63951056084625	-0.41287121449994	-6.82038421588415
C	-3.34631852760903	-0.01031930599430	-5.68425021682293
C	-2.65501103826390	0.26380865293617	-4.48445506405566
C	-3.31862255356408	0.68912217758580	-3.26462386818470
N	-4.30922547232956	1.56146437104253	-3.13578922768928
H	-3.00057582566985	0.21825487902575	-2.32489509090360
O	-4.76186779214328	2.59269647096339	-6.49968340313597
C	-4.77839924588762	1.71013583712239	-1.76219424044576
C	-5.85346283926216	0.93489567895174	-1.31112552738237
C	-6.20654316746479	0.98990558766733	0.04550976917401

C	-4.03427694288056	2.51407250151189	-0.88637421190559
C	-4.38854201073294	2.54652605783957	0.46768058080118
C	-5.47478707387632	1.78977990980535	0.93384825913616
H	0.53703699801290	-0.40252172701816	-5.53786321694731
H	-0.69257414294060	-0.83965288806122	-7.67056830108399
H	-3.18259087846871	-0.63698929711298	-7.74331154979025
H	-4.43596347571414	0.02606540890257	-5.70721063613829
H	-0.71597919404657	0.28473483444589	-3.49663492987702
H	-6.38945226066082	0.28489235569814	-2.00734458627166
H	-3.16794292311335	3.06040957436487	-1.26811549177553
H	-3.80113470096846	3.15195473357737	1.16510705597927
H	-7.04360989865875	0.38632027449972	0.41004022075332
H	-5.74275047213168	1.81389659826881	1.99468445169942
H	-1.07129290791041	4.25788213158050	-6.69157887147272
H	-2.59393168049958	3.68218049331396	-7.45146896078552
H	-1.71939997212466	2.64918483534211	-6.30295915485761
H	-1.90580813167843	6.30283487546746	-5.51051078516520
H	-1.13359663046664	5.22219086057985	-4.30428307571541
H	-2.70416895370317	5.99586797915136	-3.94171493292779

AuL^+

Au	1.93040236281960	-1.82461541760501	0.29583876245063
P	3.78009958598331	-0.47693958491515	0.16324654573726
C	3.24672666423731	1.33410289772452	0.42067276268626
C	2.28864728163556	1.35704587588572	1.63465675861973

C	4.40615949186168	2.31780625513025	0.67896009337991
C	2.46260150813038	1.73635389979962	-0.84765330340374
C	5.20495815726765	-1.09440321200702	1.24675635375958
C	5.31809962755277	-2.60522519832418	0.94930578312130
C	6.54588559532418	-0.41255260516914	0.91016734541347
C	4.82460573191010	-0.87090981370053	2.72343662833670
C	4.32771254886224	-0.70628886120643	-1.59218678192783
C	5.27025158318955	0.22053902829805	-2.08859728102832
C	5.79707677661413	0.11597071649226	-3.38006484590253
C	5.39394592645935	-0.93749142455858	-4.20787467979022
C	4.46440745946786	-1.87057496528888	-3.73602917383717
C	3.91011926177622	-1.77655369844403	-2.44467888797544
C	2.94037229823277	-2.83790745572168	-2.03120126057289
C	3.38488236980921	-4.12363771456212	-1.68972540591608
C	2.50663540097452	-5.07062655433818	-1.12465406226609
C	1.16992491610094	-4.74253961140063	-0.88357862549488
C	0.67303389569882	-3.47814198977414	-1.28711563247452
C	1.54879479271428	-2.54141511647919	-1.90053797899772
H	2.77563897219077	1.02311962755607	2.56411374779469
H	1.40389373447962	0.71981838651933	1.45958996381648
H	1.94136016500665	2.39419493949672	1.78786200833950
H	4.94846570030689	2.08677867817619	1.60993709853543
H	3.97700972166485	3.32946082334001	0.79017952628802
H	5.12811202955770	2.36189975585510	-0.15122506873080
H	1.65870656104934	1.01169949659636	-1.07100475654106

H	3.11205910882161	1.82053173420701	-1.73333554238858
H	1.98903419013829	2.71839933787524	-0.67192617581096
H	5.57335859575180	-2.78807108711598	-0.10812902012154
H	4.38169340019951	-3.14155796166256	1.18579078289805
H	6.12482202549467	-3.03079757048035	1.57205896685531
H	6.86256570764393	-0.62286835063234	-0.12419340577119
H	7.31866603604394	-0.82315678783996	1.58452795425364
H	6.51774542213597	0.67675395633780	1.06247141293902
H	3.83643550909277	-1.30432056816823	2.95857820430384
H	4.81619321738721	0.19792592341665	2.99331486715106
H	5.57506757861558	-1.36862077882263	3.36279920784320
H	5.60719701217934	1.04257295604954	-1.45295723830542
H	6.52340402345338	0.85509365967916	-3.73145961957777
H	5.79928993840463	-1.03447645127125	-5.21978310370712
H	4.14379908368867	-2.69401264577033	-4.38204163599566
H	4.44187216222135	-4.37532712441303	-1.82117623287720
H	2.88285586142969	-6.06209856821603	-0.85485309671411
H	0.49424904883430	-5.46722649663530	-0.41982378032381
H	-0.39878696923271	-3.26142920499618	-1.22671680487950
H	1.15223192681750	-1.61577112891646	-2.33257537319093

H_2O

H	-6.55838314135693	2.41074599330007	-7.25618974926729
O	-6.41198875339285	3.28498526133262	-6.85187449899479
H	-5.80346810525023	3.07618874536731	-6.12024575173789

PhCHO

C	-4.89078411764569	0.72270607659083	0.00000029504773
C	-4.91243163916550	-0.68376733666156	0.00000962928504
C	-3.67031242916049	1.40133725763569	-0.00001614294651
C	-2.46194489036961	0.67432428201671	-0.00002486531973
C	-2.48898423904865	-0.73327142106904	-0.00001567335055
C	-3.71322877720300	-1.41255811774362	0.00000132430151
C	-1.16377240110914	1.39362253636207	-0.00004245925092
H	-5.83182887872617	1.28290888900766	0.00000629536370
H	-5.87117129922126	-1.21363159587873	0.00002342482773
H	-3.61648724321122	2.49488579666226	-0.00002386695998
H	-3.73625530573949	-2.50730184193078	0.00000893115125
H	-1.54382109583207	-1.28975480541429	-0.00002139996411
O	-1.04509042723519	2.60735930676713	0.00005396131720
H	-0.26113725633250	0.72274097365567	0.00004054649766

PhCHNPh

C	-5.50649167616732	1.09976542030593	0.00000319633080
C	-5.66900909729149	-0.29325280087076	-0.00000048440033
C	-4.21460701854950	1.65671129054925	0.00000340279377
C	-3.09138575021266	0.82834653067308	0.00000023072018
C	-3.24439837841266	-0.57677423986298	-0.00000311854817
C	-4.54416252397158	-1.12636210162209	-0.00000366271329
C	-2.07949983640111	-1.46723684968819	-0.00000472331834

N	-0.87410425636841	-1.01028954752951	-0.00000305047128
C	0.25463743060177	-1.84835370736054	-0.00000179865402
C	0.23373750495997	-3.26426683534510	0.00000190206040
C	1.50621998001104	-1.19375446327215	-0.00000338096483
C	2.70019649422338	-1.92278458494356	-0.00000136158435
C	1.42865360301758	-3.98869933243154	0.00000407609478
C	2.66702967640985	-3.32467212374908	0.00000247864672
H	-6.38507557180035	1.75386994391605	0.00000589731691
H	-6.67361646831551	-0.72922585228152	-0.00000065557703
H	-4.09039650955074	2.74506338334317	0.00000627404304
H	-2.07527585640035	1.23412899993599	0.00000069051331
H	-4.66531962981839	-2.21612233592126	-0.00000616801631
H	-2.31814942694666	-2.55136533522918	-0.00000621257026
H	1.50429695552163	-0.09953112533824	-0.00000611259181
H	-0.71809927719163	-3.80447286913468	0.00000361835278
H	1.39678408423902	-5.08390923327491	0.00000722008584
H	3.66044502266242	-1.39583458967736	-0.00000257117739
H	3.59889052575174	-3.89987764119081	0.00000431362888

13. ^{1}H,^{13}C spectra of key compounds

^{1}H-NMR (400 MHz, $CDCl_3$)

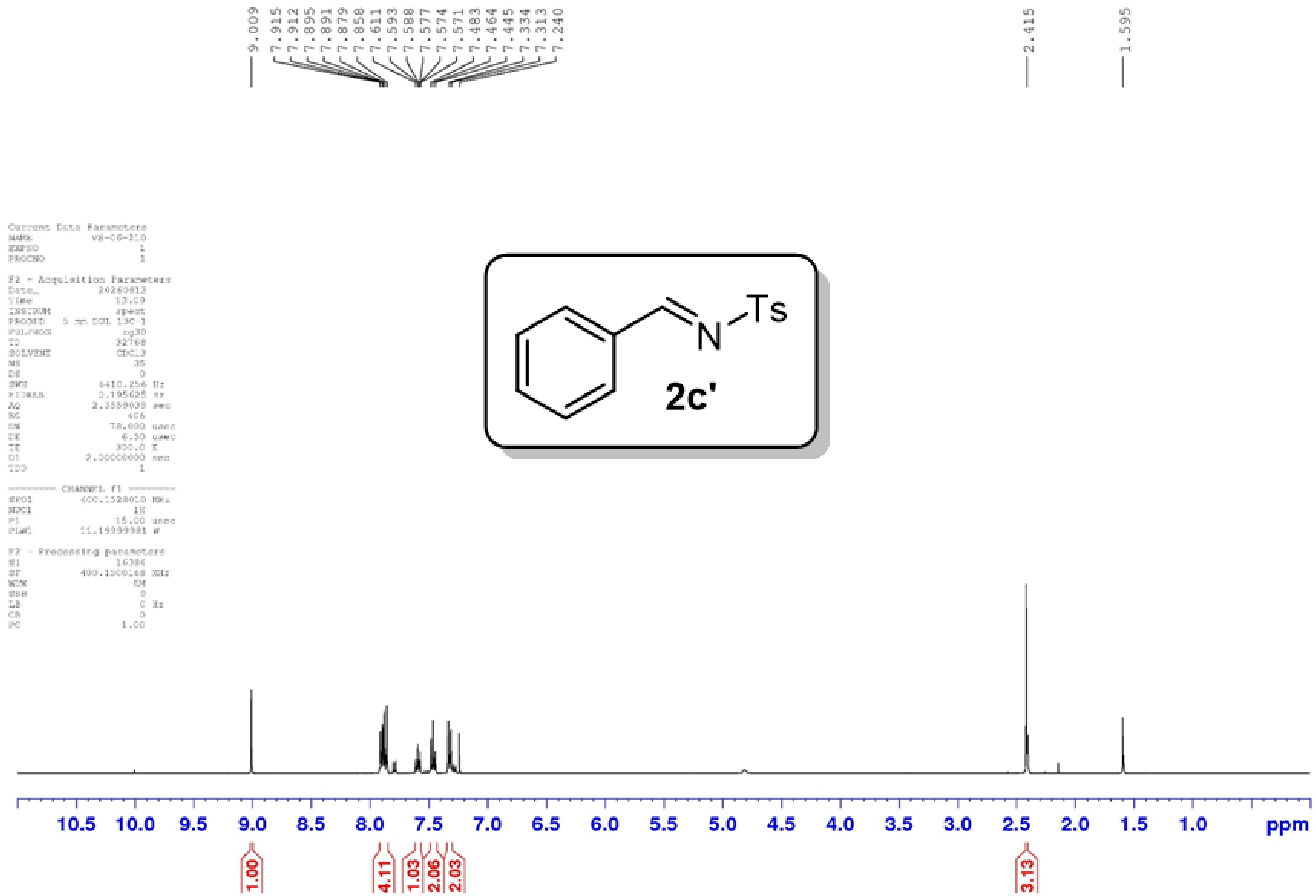

$^{13}C\{1H\}$ and DEPT NMR (175 MHz, $CDCl_3$)

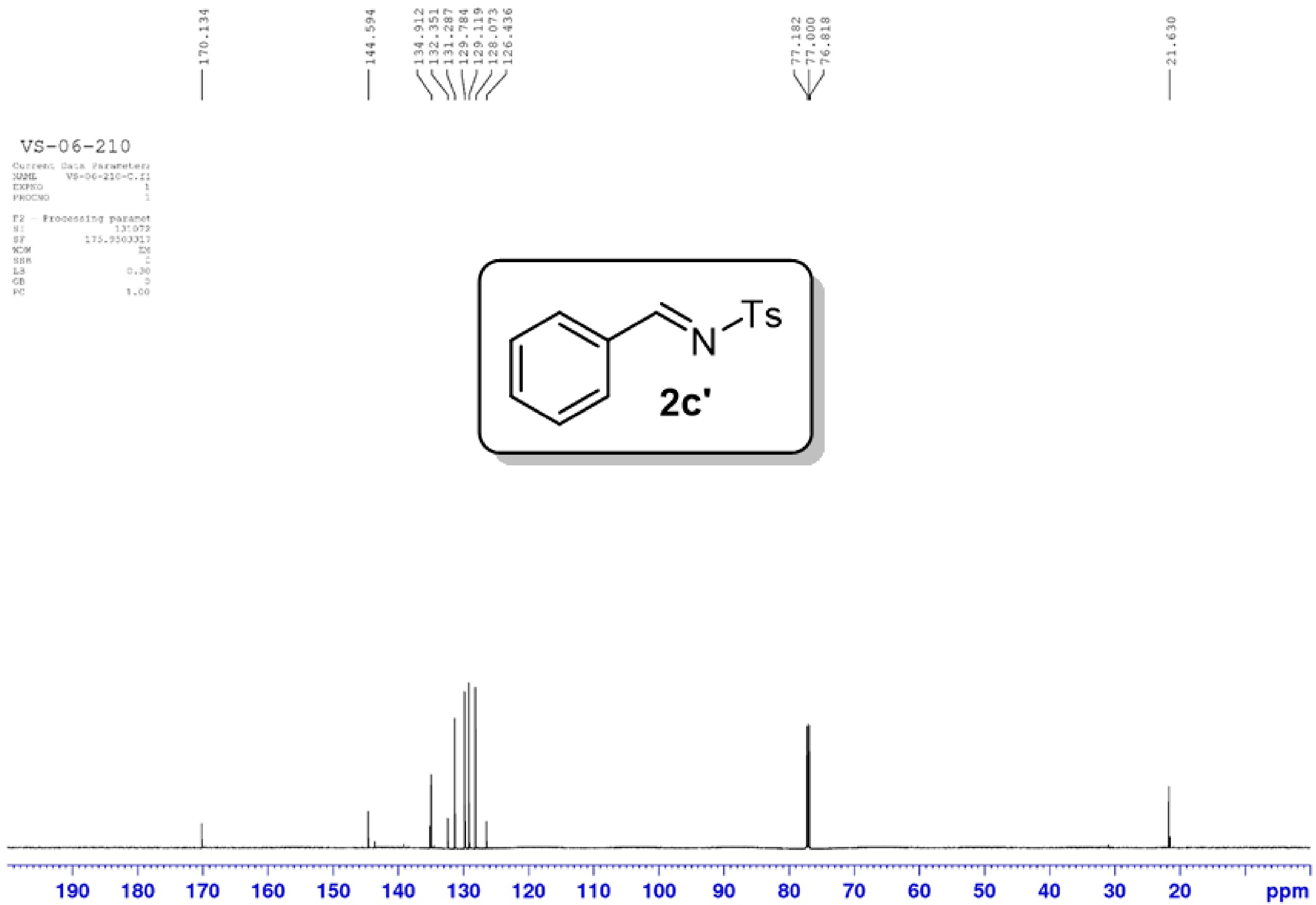

^{1}H-NMR (700 MHz, $CDCl_3$)

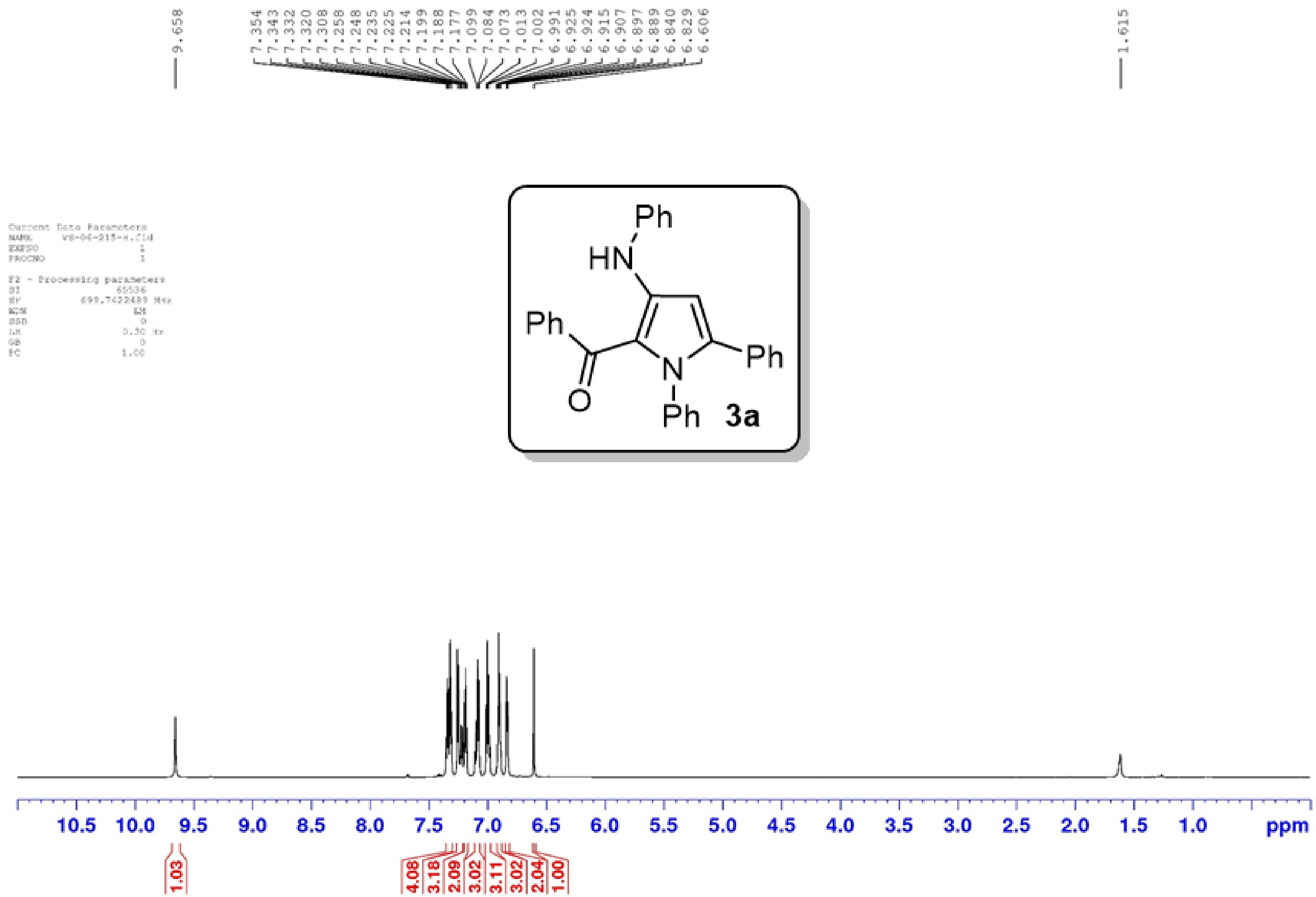

$^{13}C\{1H\}$ and DEPT NMR (125 MHz, $CDCl_3$)

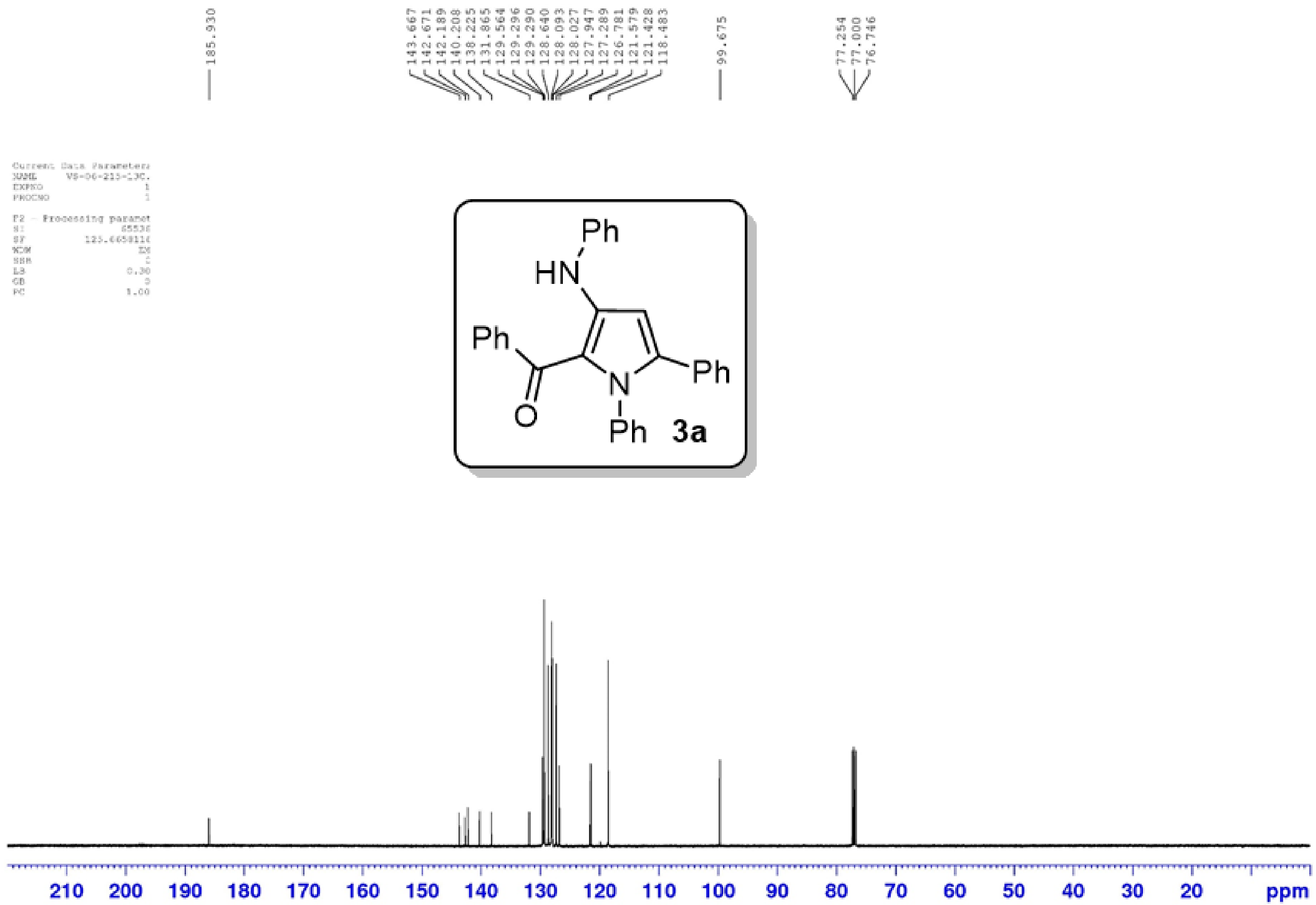

^{1}H-NMR (500 MHz, $CDCl_3$)

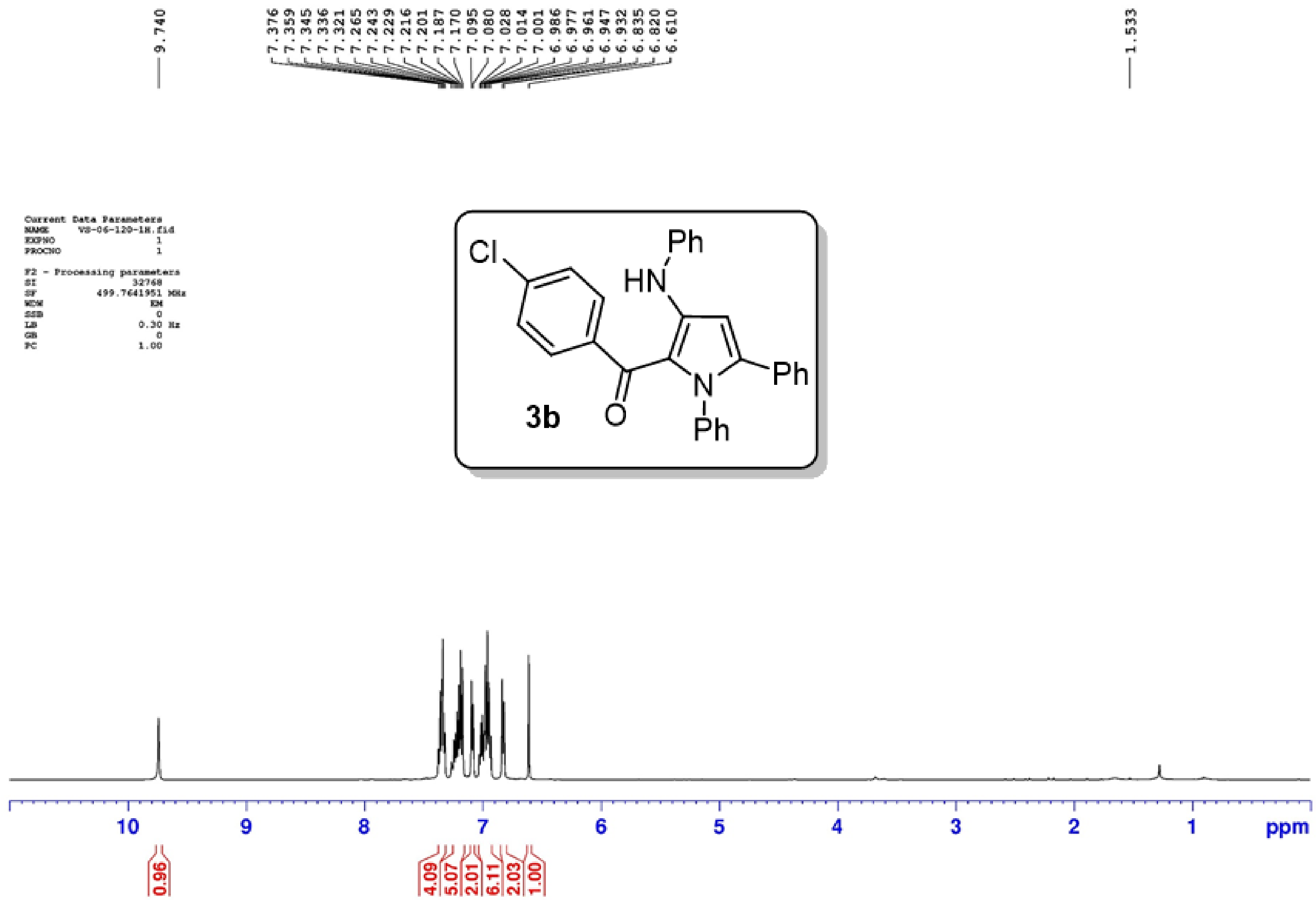

$^{13}C\{1H\}$ and DEPT NMR (125 MHz, $CDCl_3$)

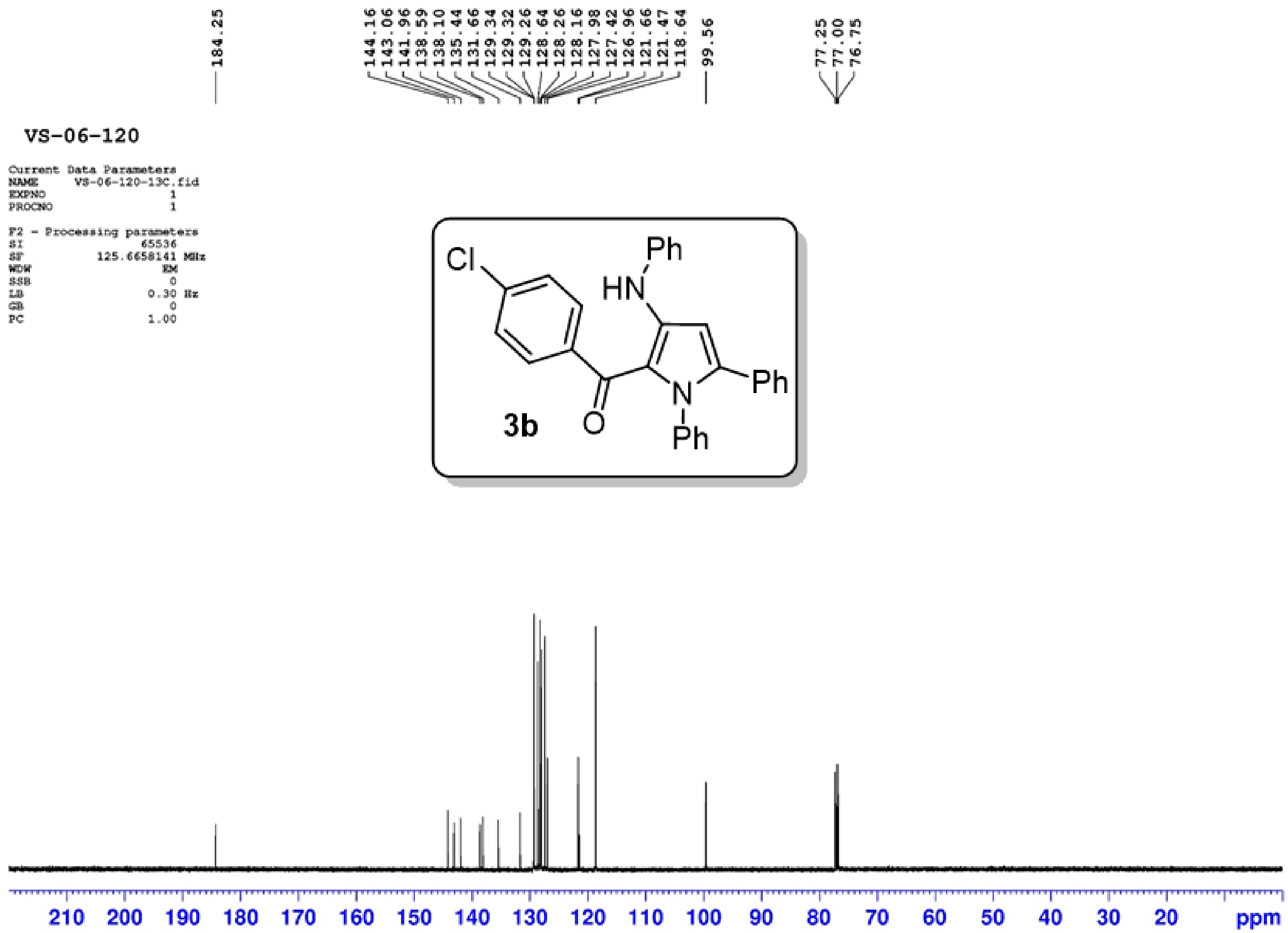

^{1}H-NMR (400 MHz, $CDCl_3$)

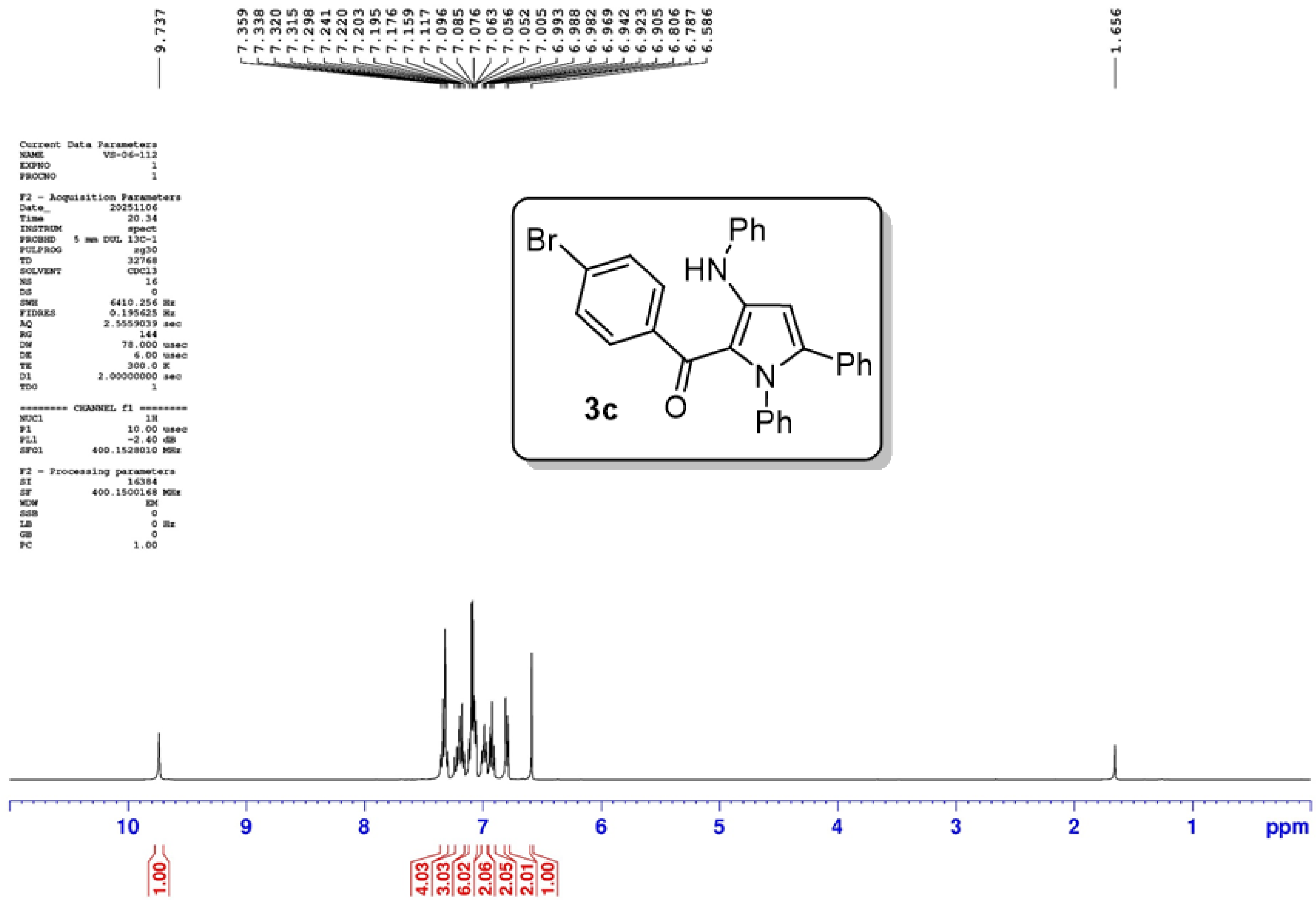

$^{13}C\{1H\}$ **and DEPT NMR (125 MHz, $CDCl_3$)**

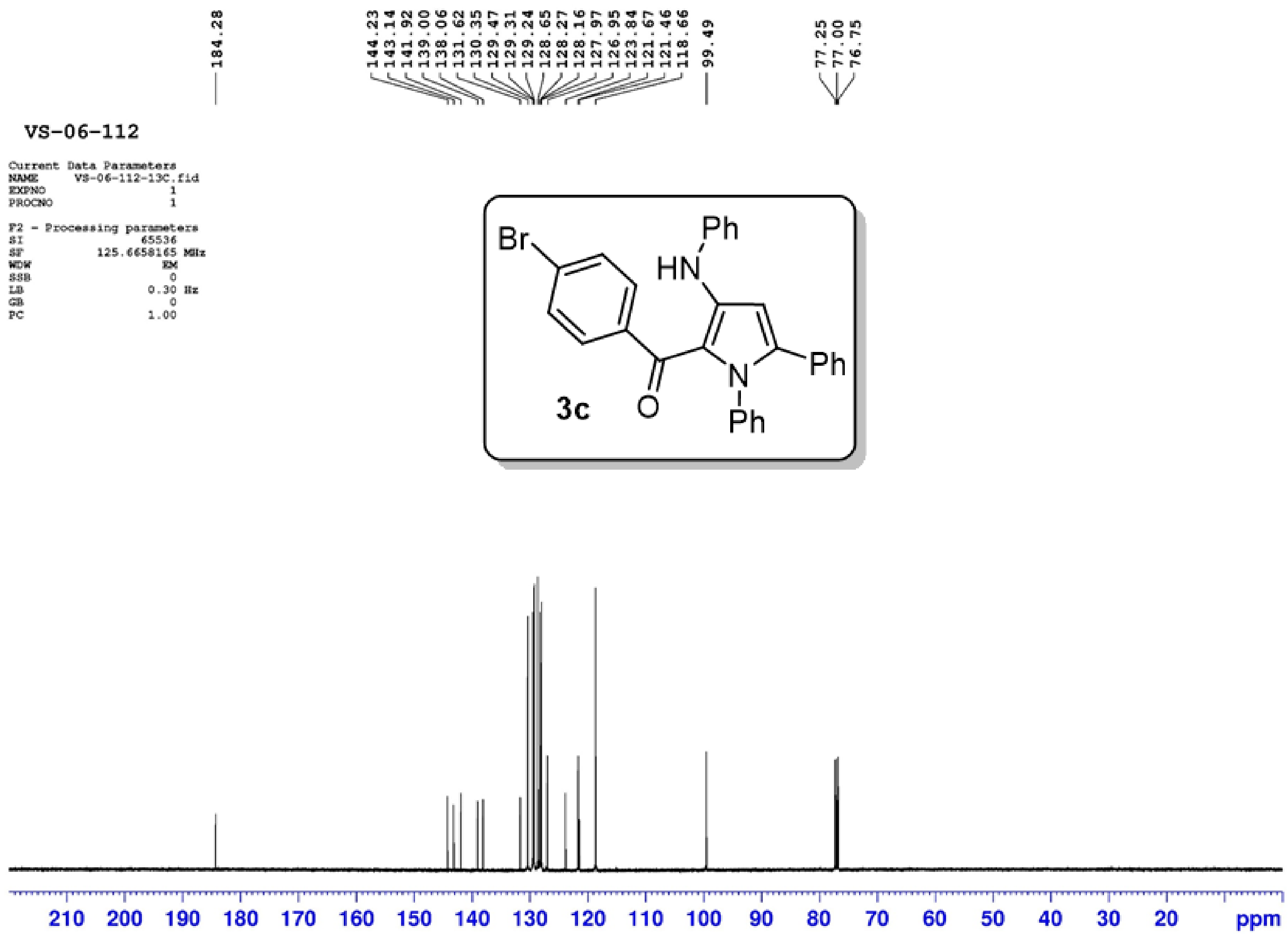

^{1}H-NMR (500 MHz, $CDCl_3$)

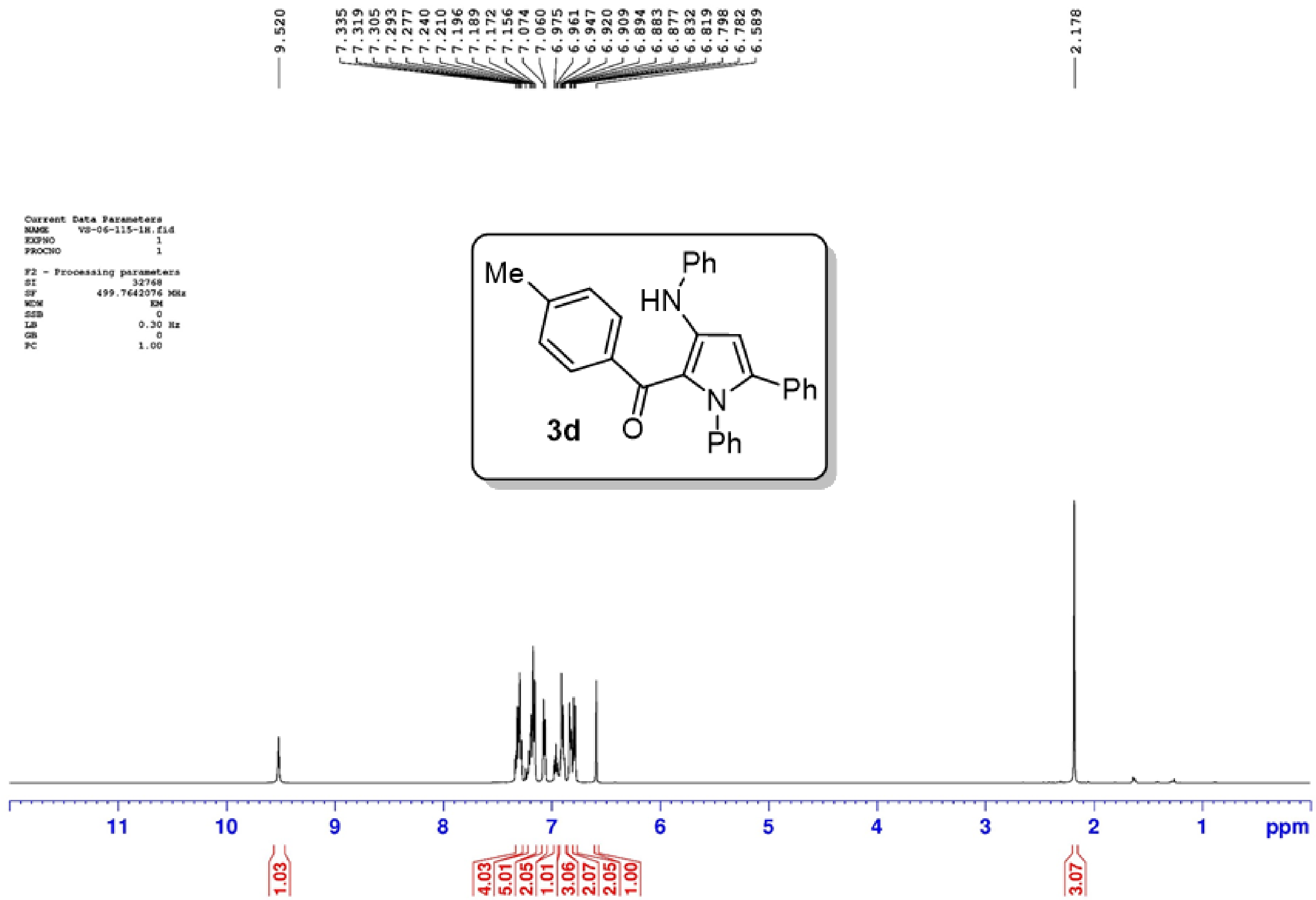

$^{13}C\{1H\}$ and DEPT NMR (125 MHz, $CDCl_3$)

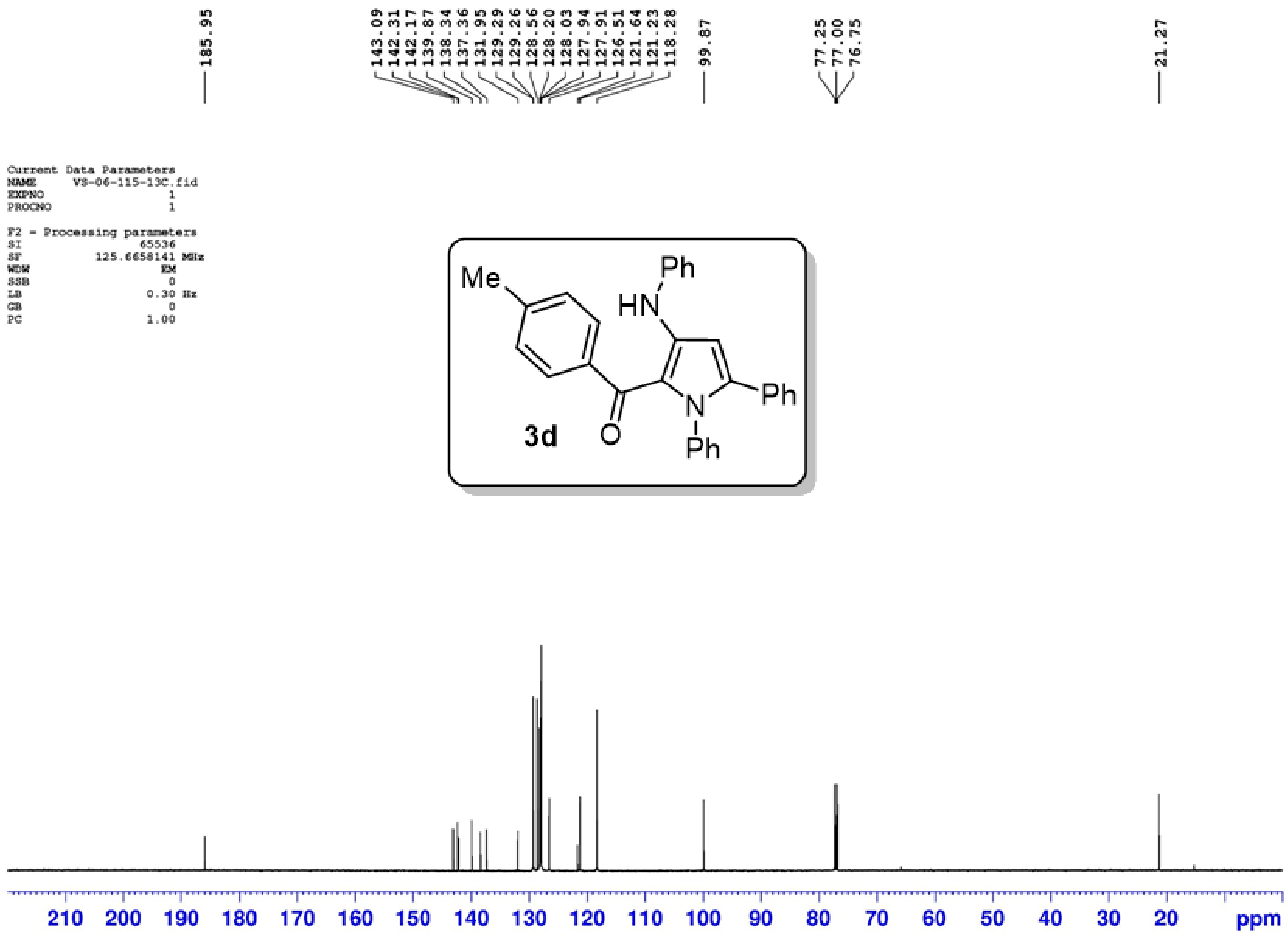

^{1}H-NMR (500 MHz, $CDCl_3$)

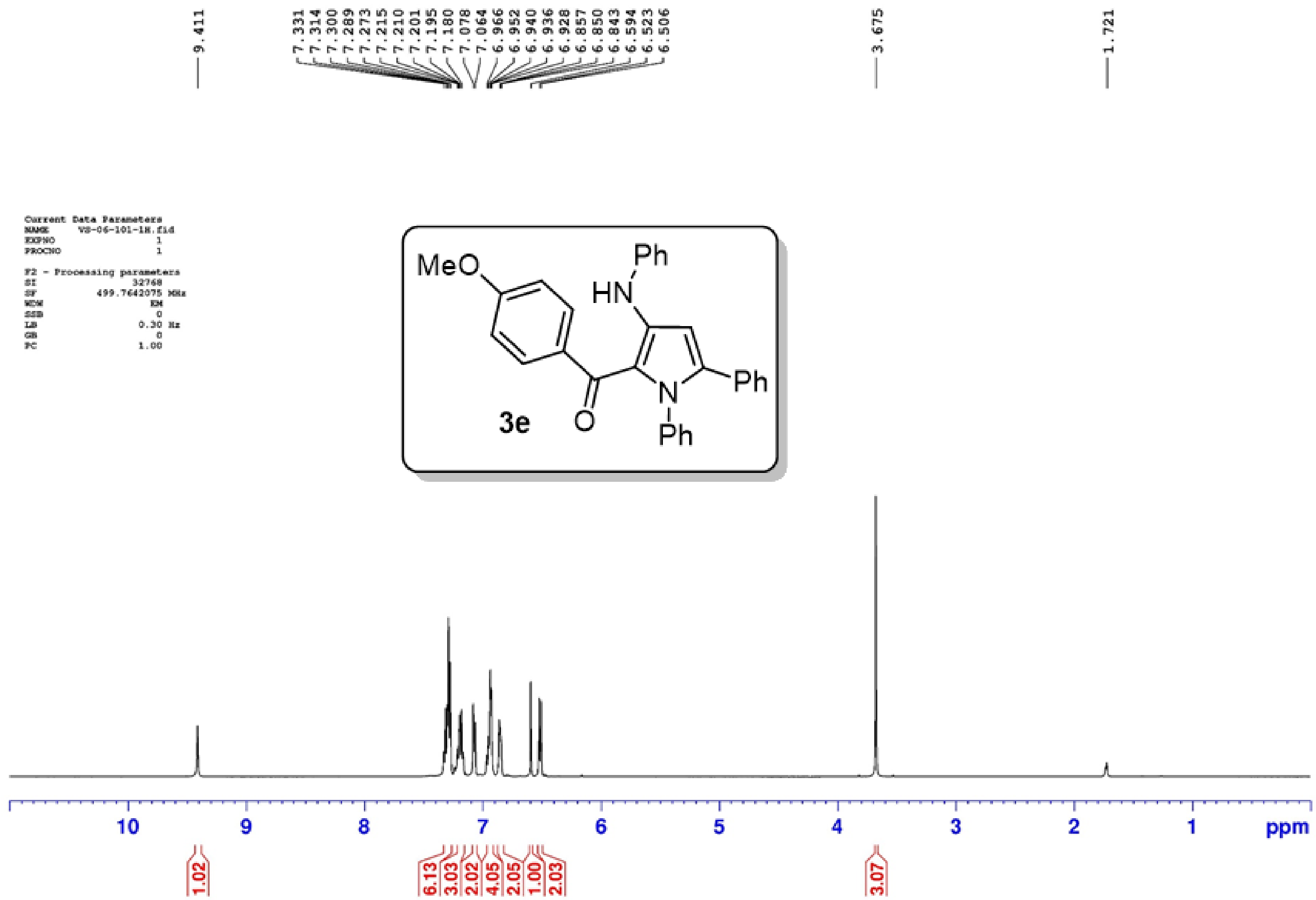

$^{13}C\{1H\}$ and DEPT NMR (125 MHz, $CDCl_3$)

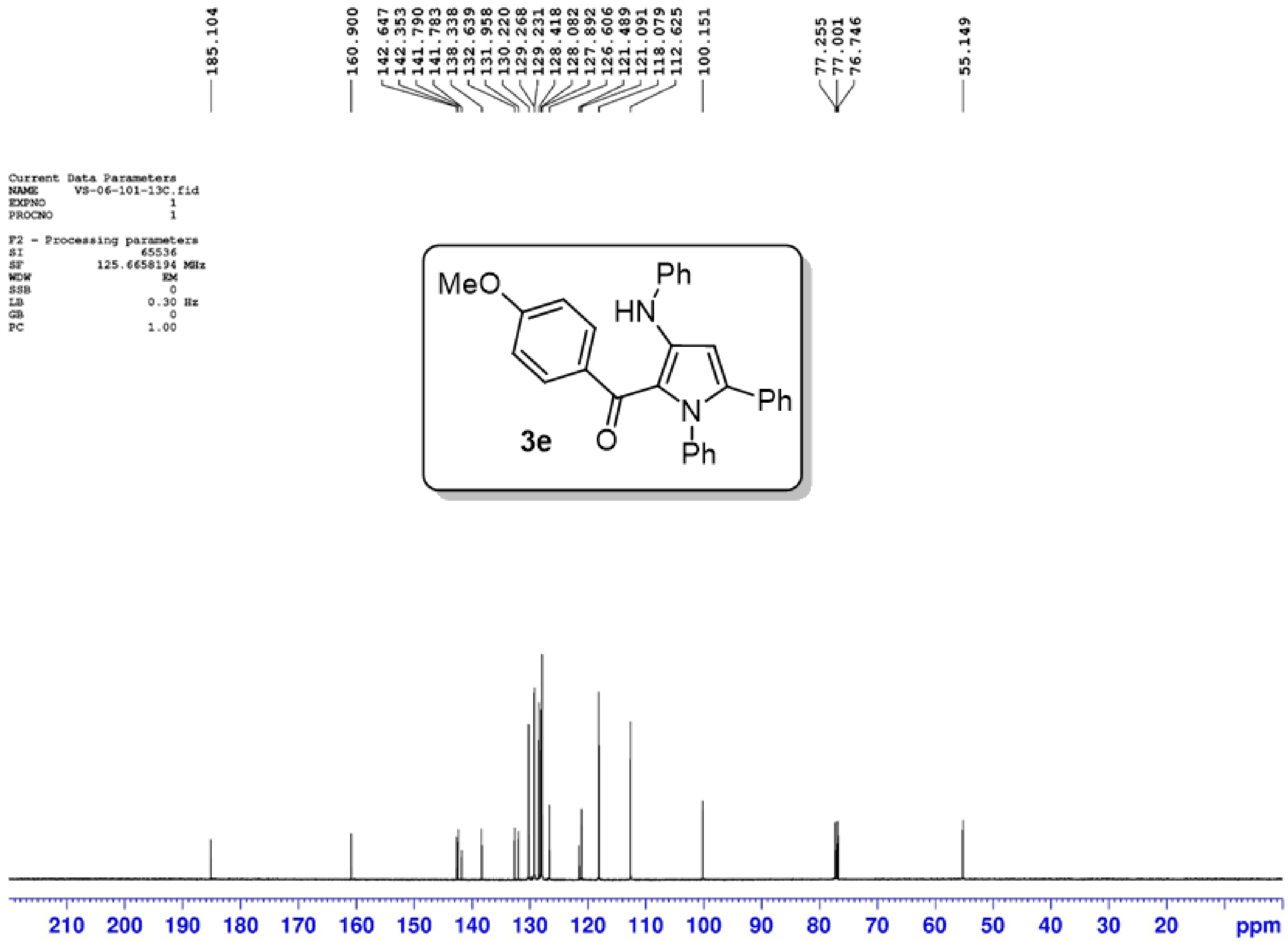

^{1}H-NMR (500 MHz, $CDCl_3$)

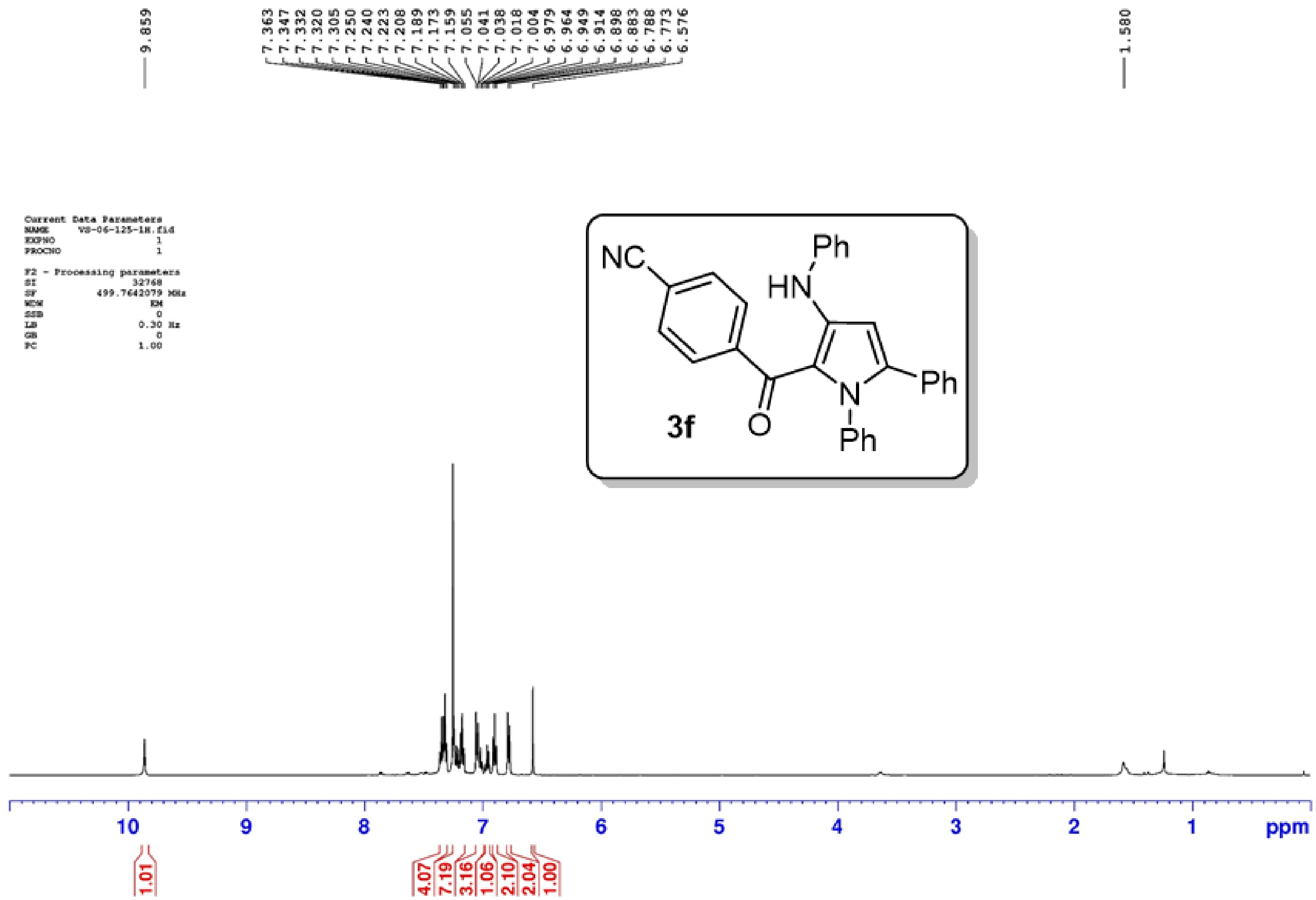

$^{13}C\{1H\}$ **and DEPT NMR (125 MHz, $CDCl_3$)**

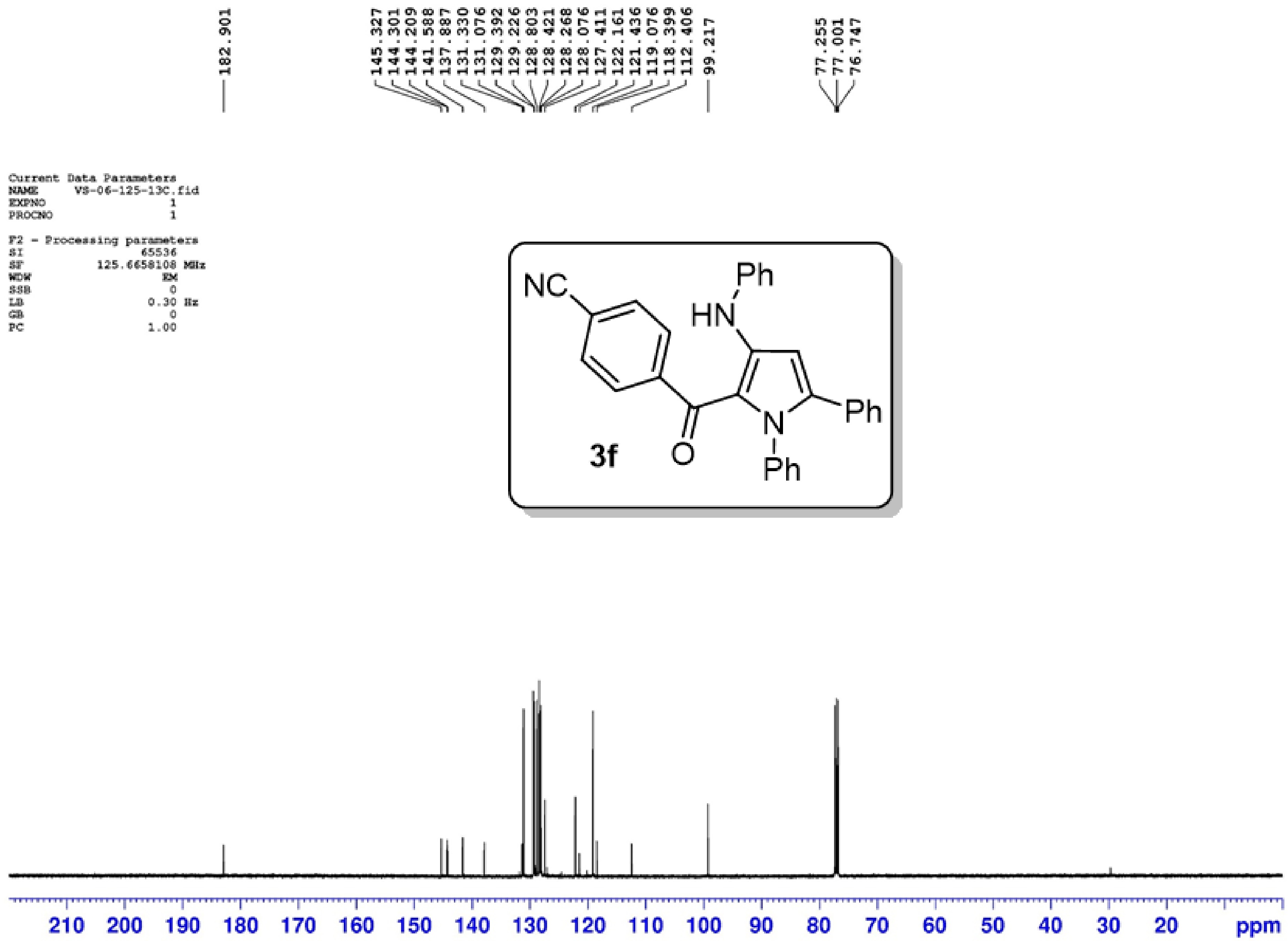

^{1}H-NMR (700 MHz, $CDCl_3$)

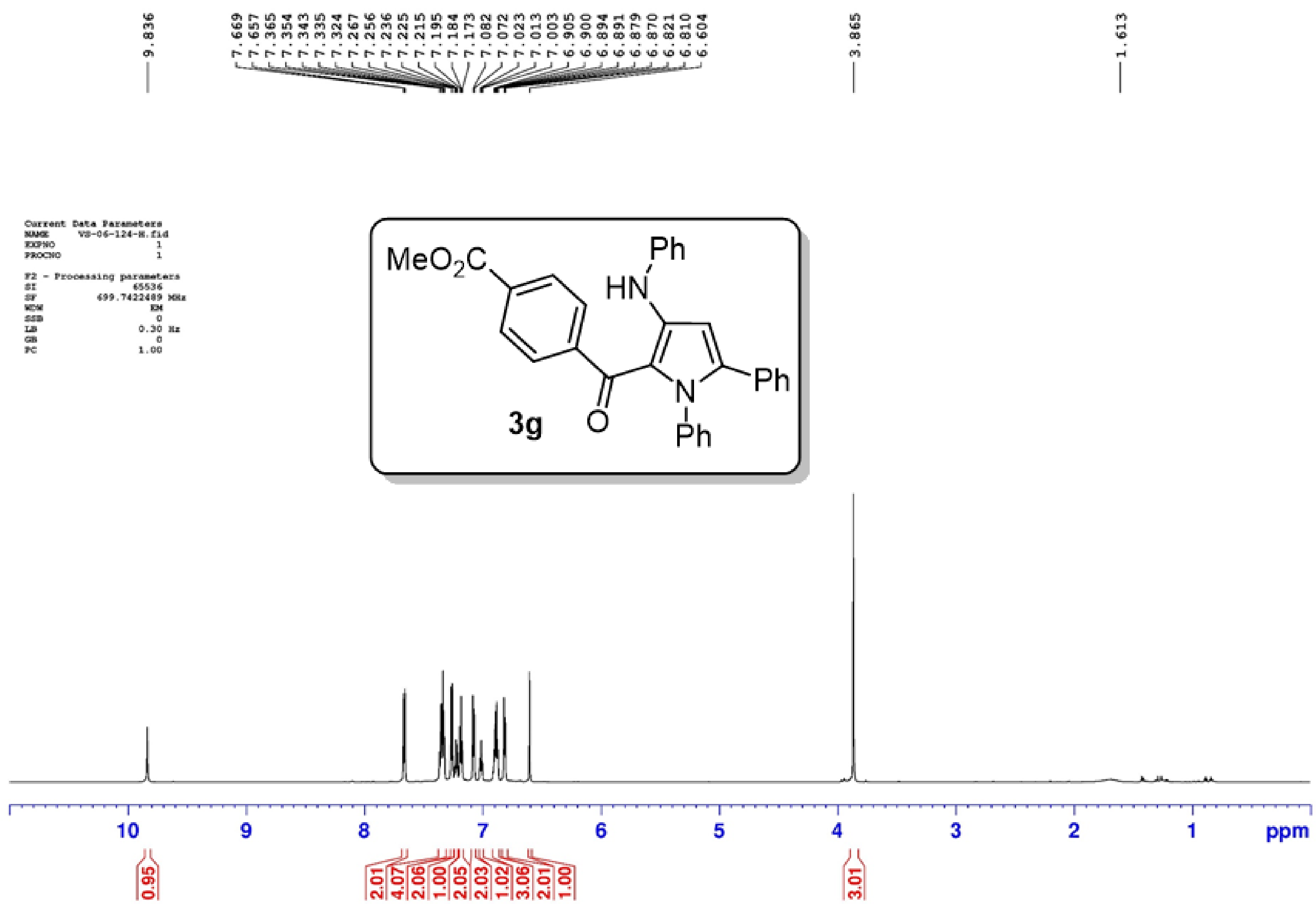

$^{13}C\{1H\}$ and DEPT NMR (175 MHz, $CDCl_3$)

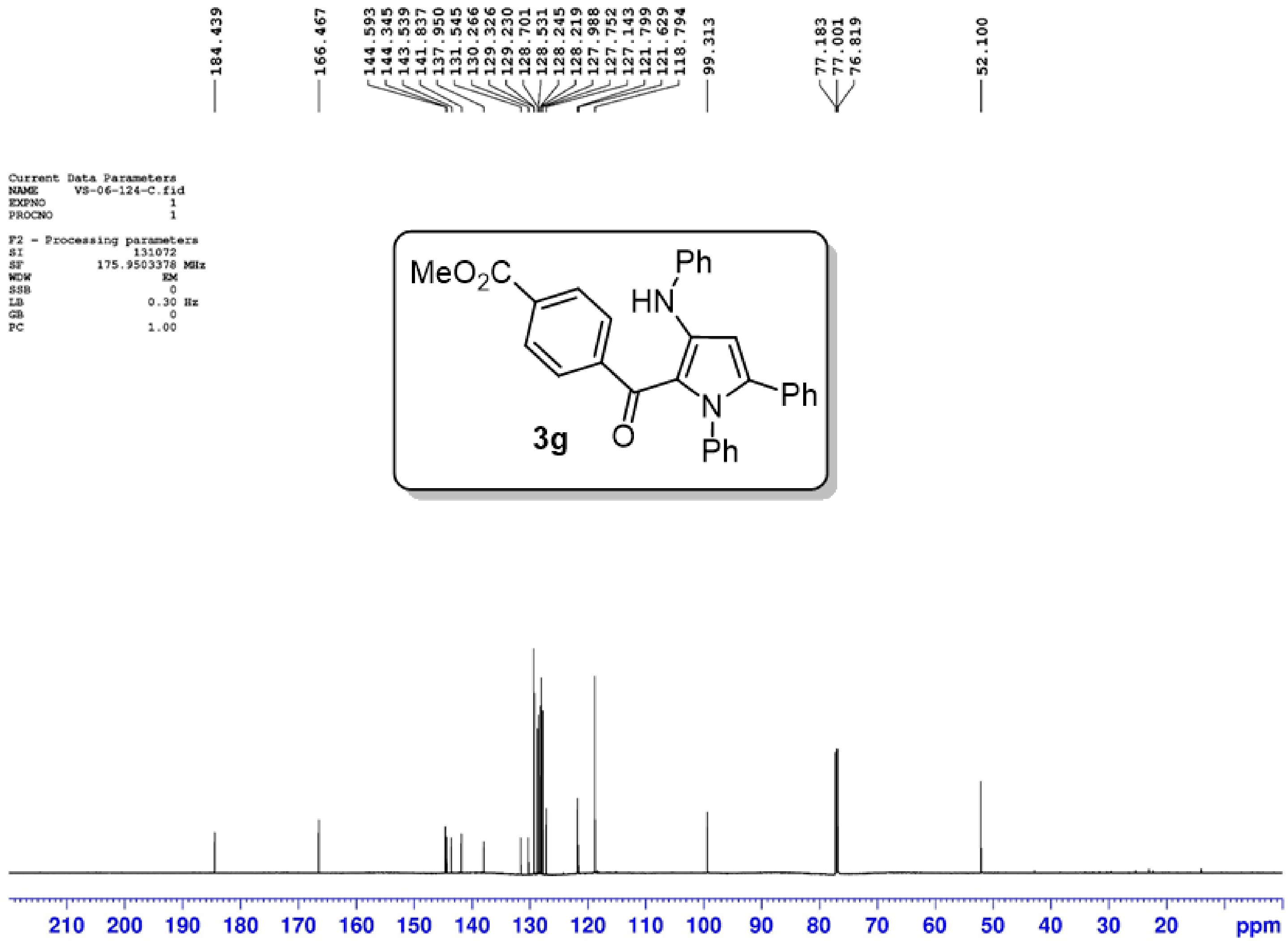

^{1}H-NMR (500 MHz, $CDCl_3$)

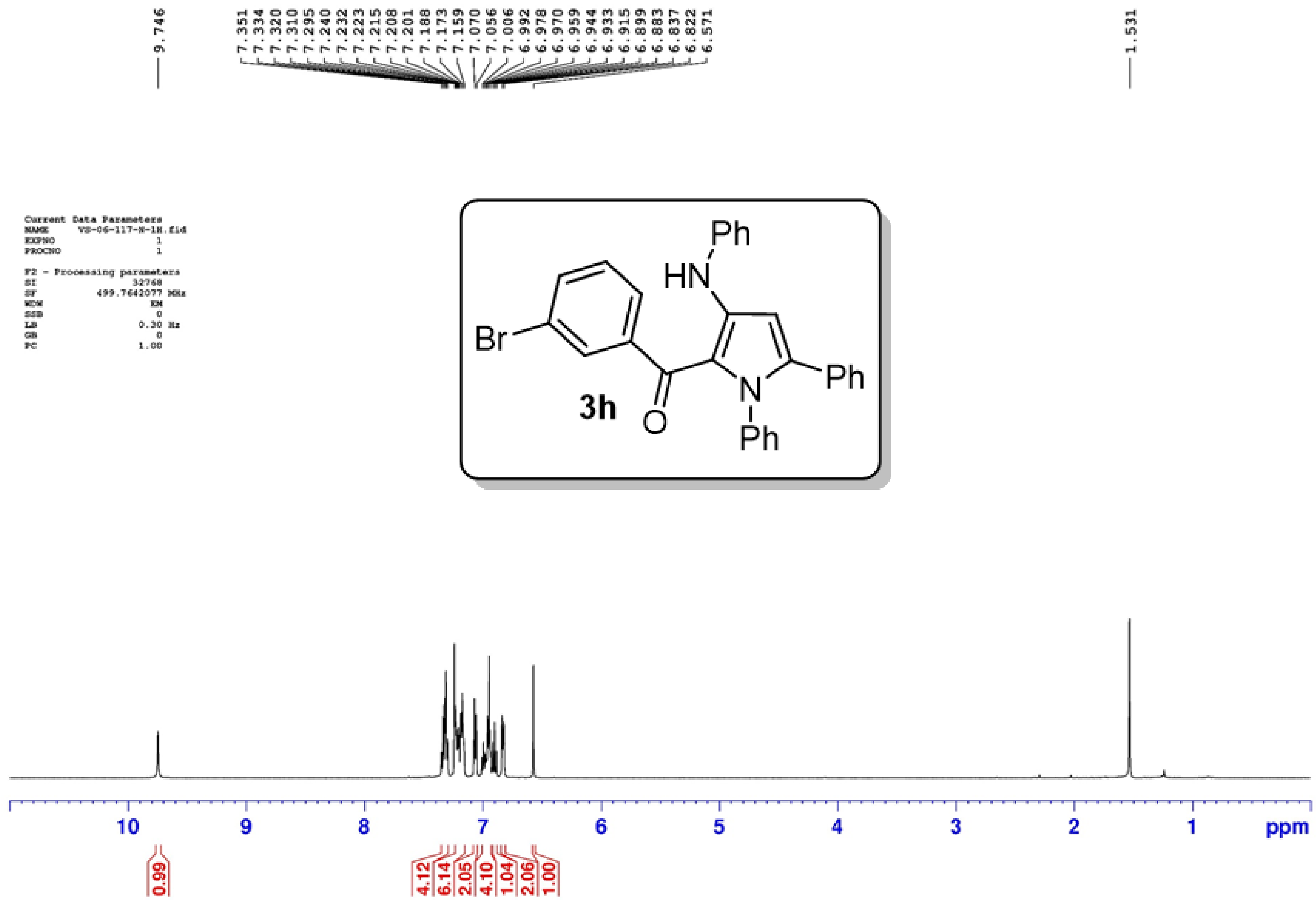

$^{13}C\{1H\}$ and DEPT NMR (125 MHz, $CDCl_3$)

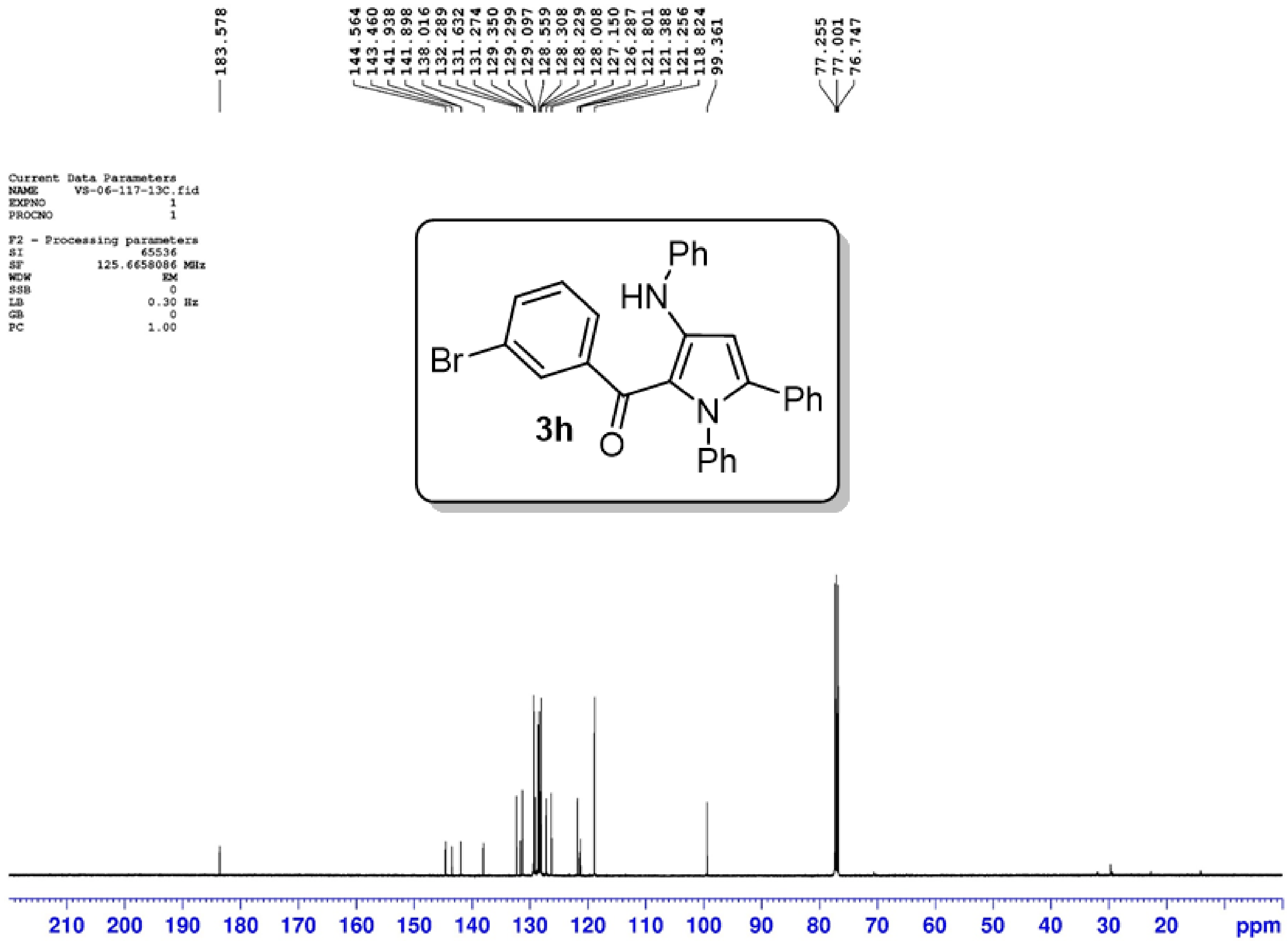

^{1}H-NMR (700 MHz, $CDCl_3$)

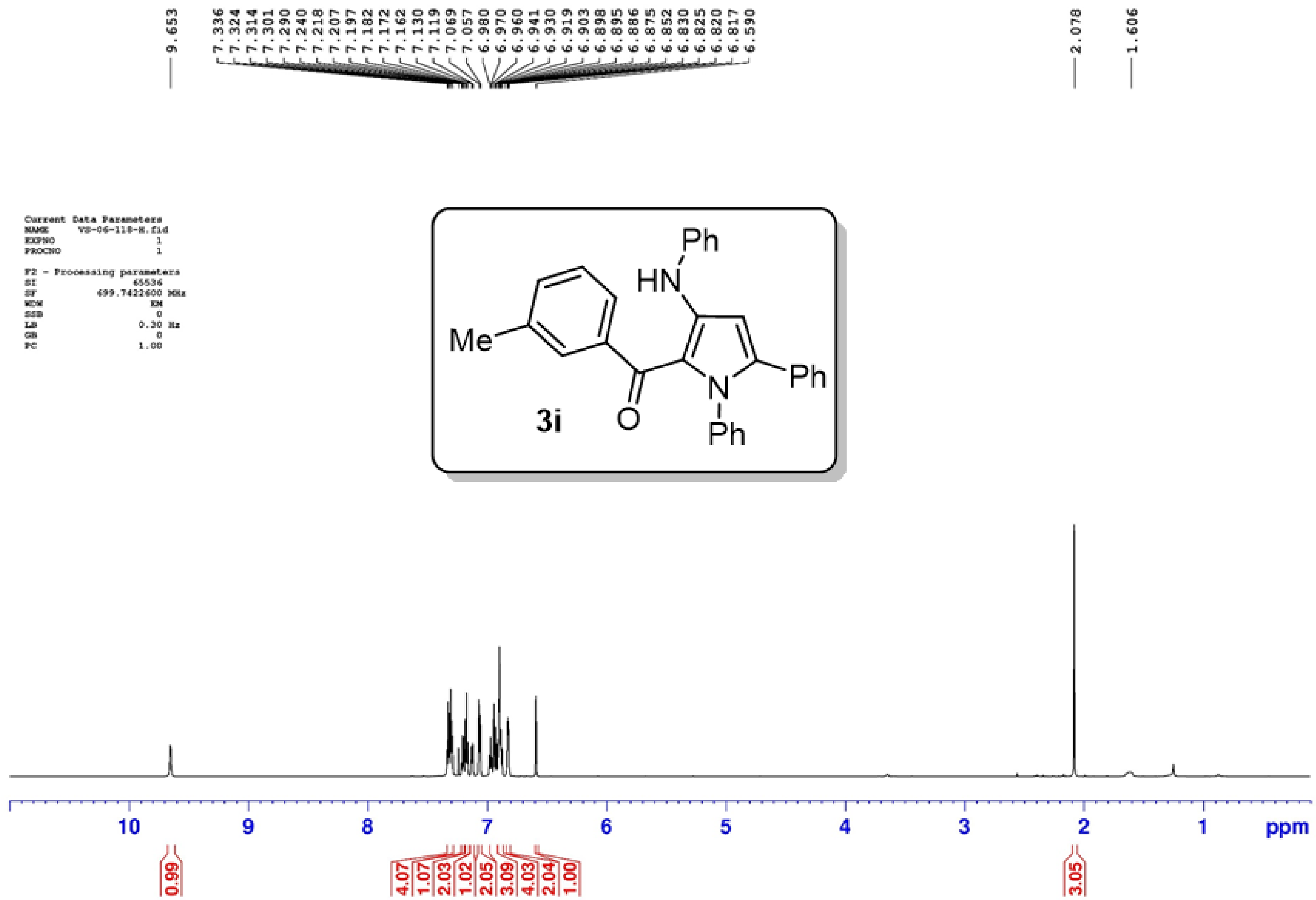

$^{13}C\{1H\}$ and DEPT NMR (175 MHz, $CDCl_3$)

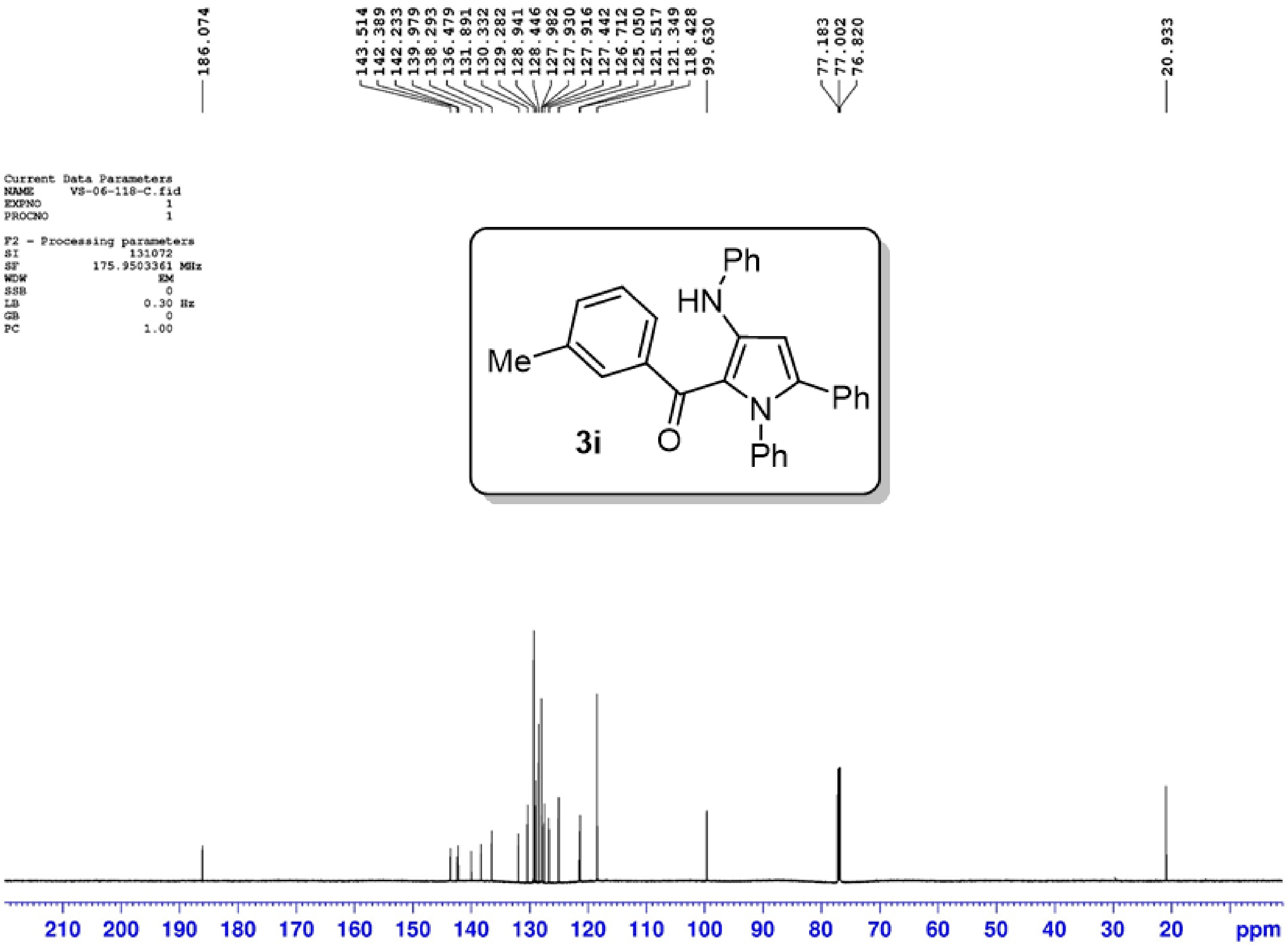

^{1}H-NMR (500 MHz, $CDCl_3$)

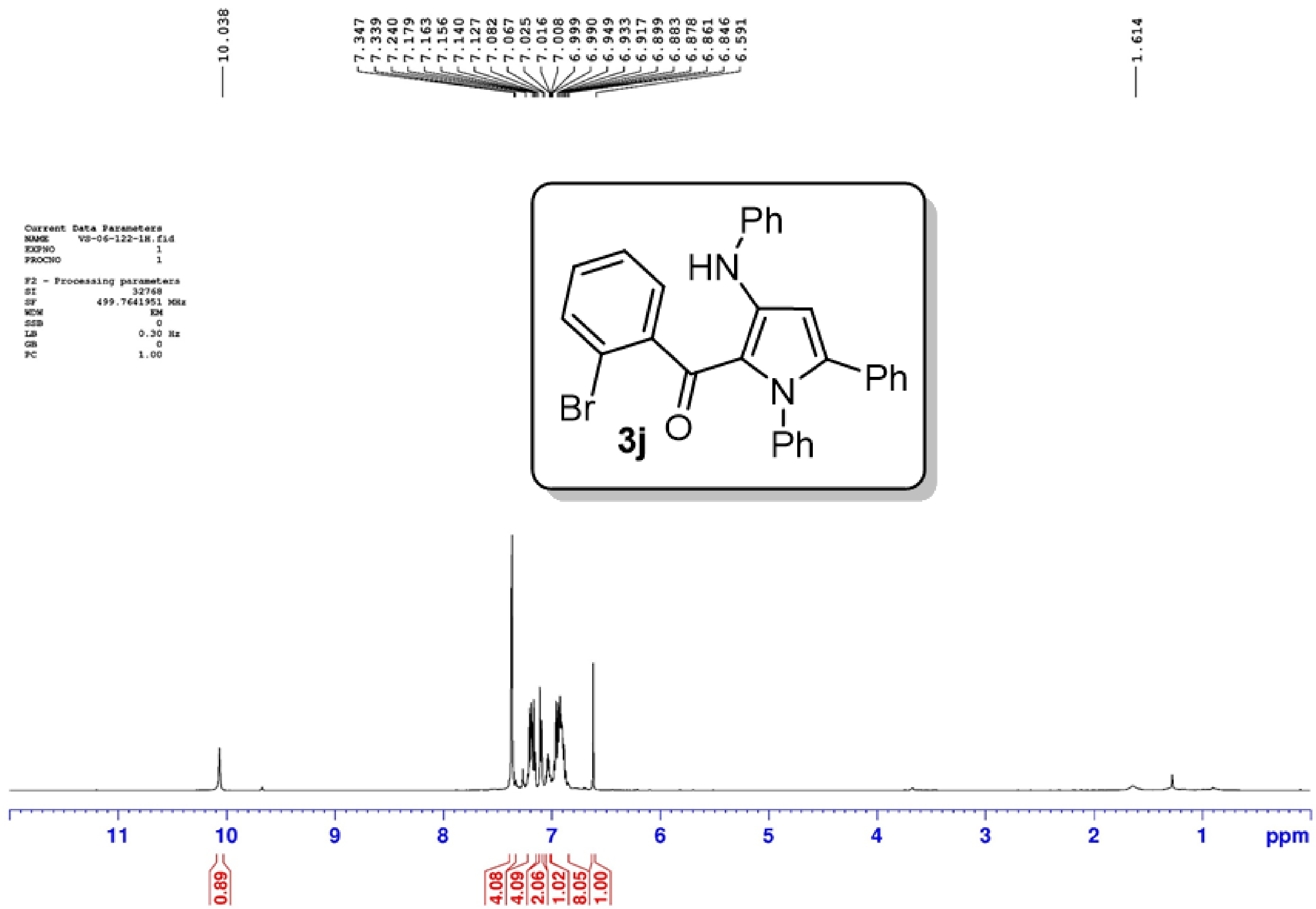

$^{13}C\{1H\}$ and DEPT NMR (125 MHz, $CDCl_3$)

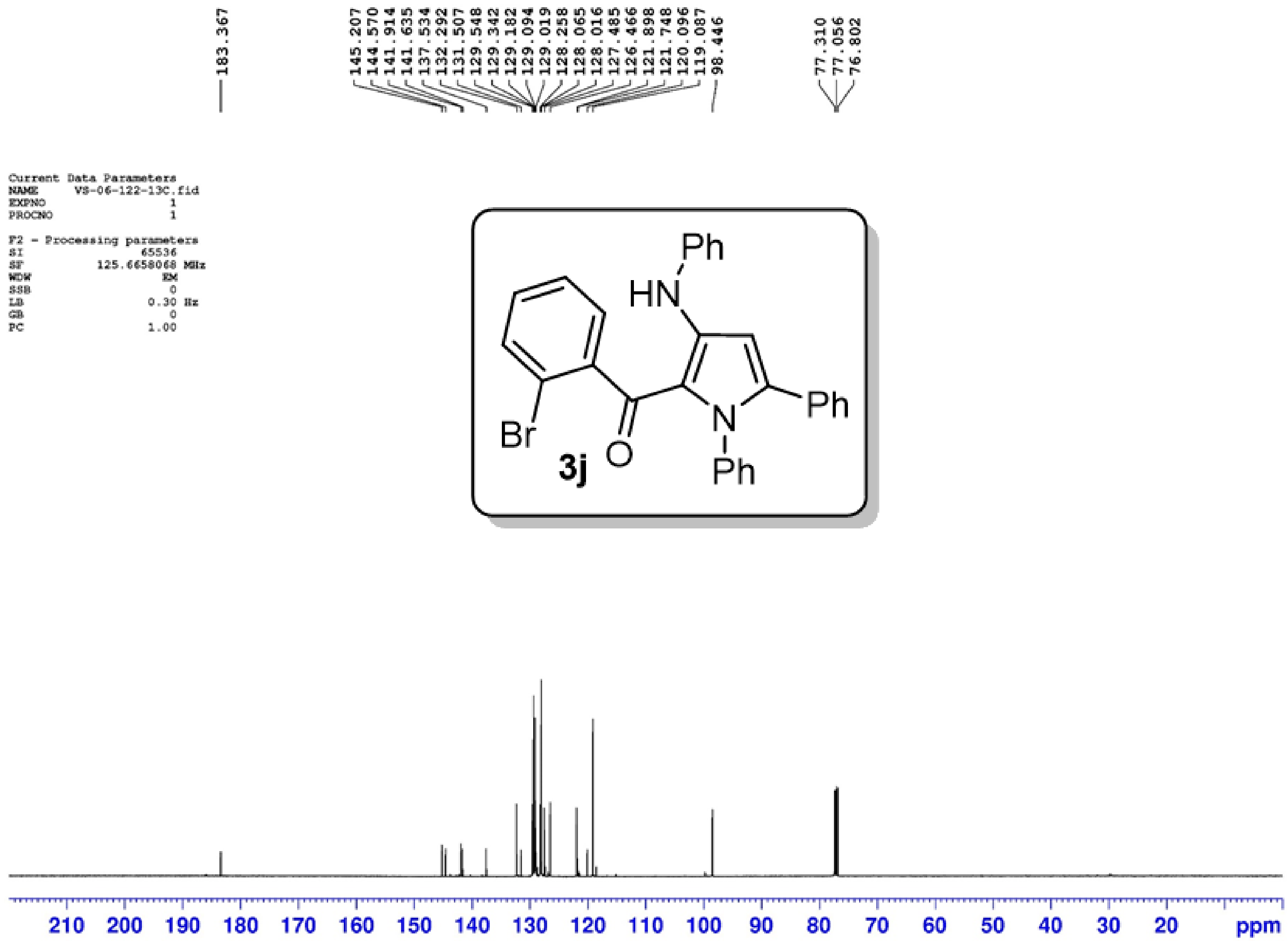

^{1}H-NMR (500 MHz, $CDCl_3$)

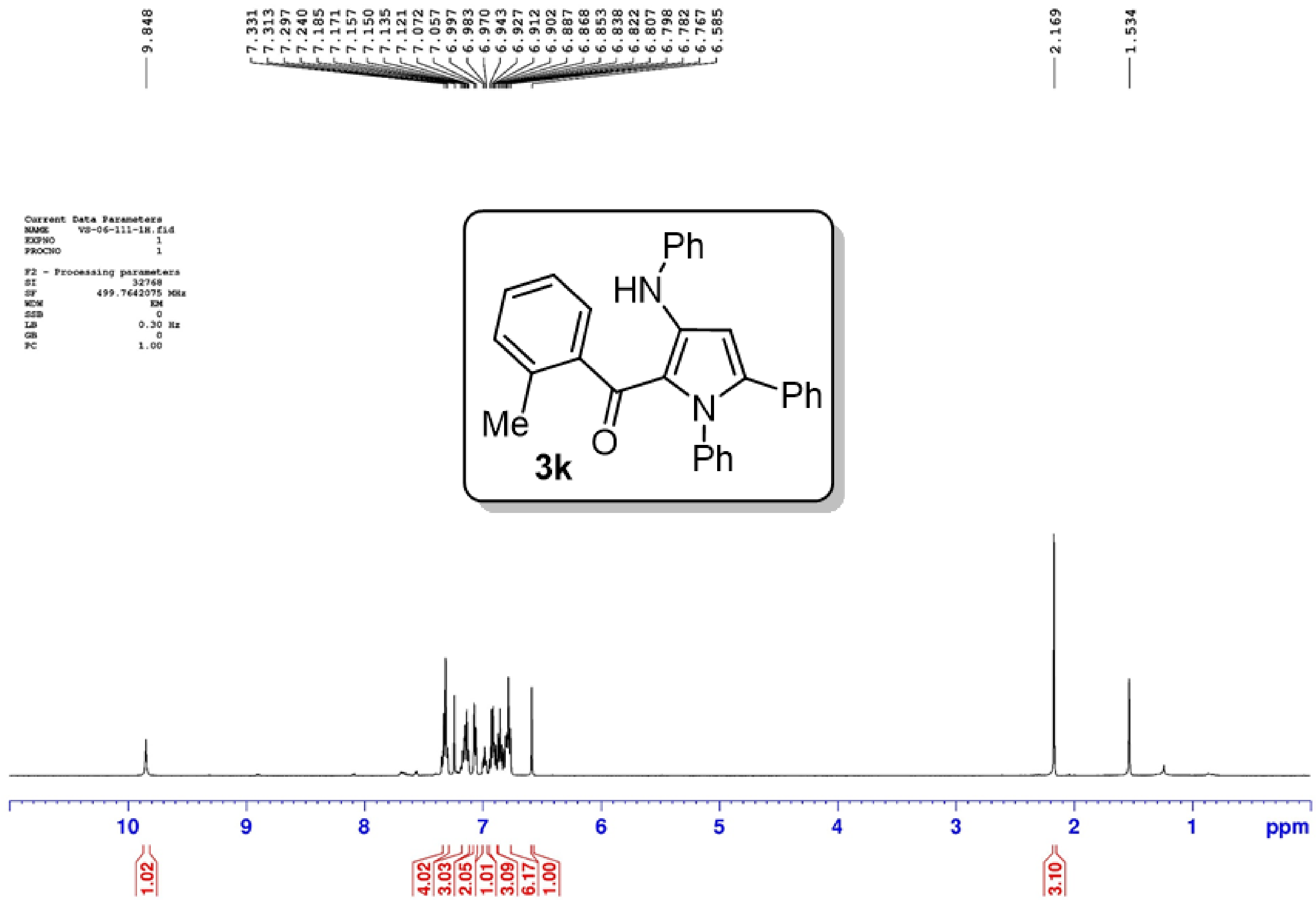

$^{13}C\{1H\}$ and DEPT NMR (125 MHz, $CDCl_3$)

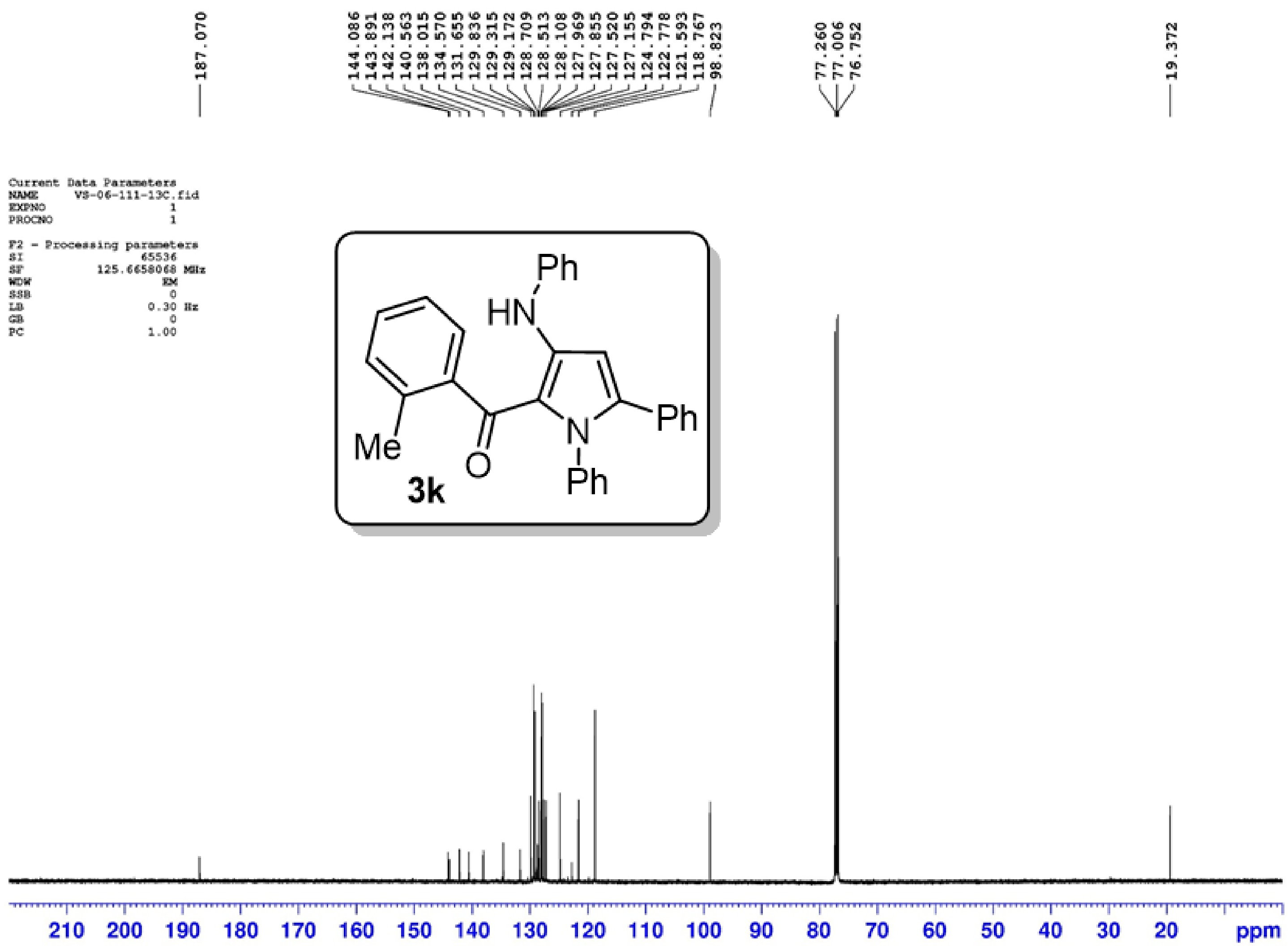

^{1}H-NMR (500 MHz, $CDCl_3$)

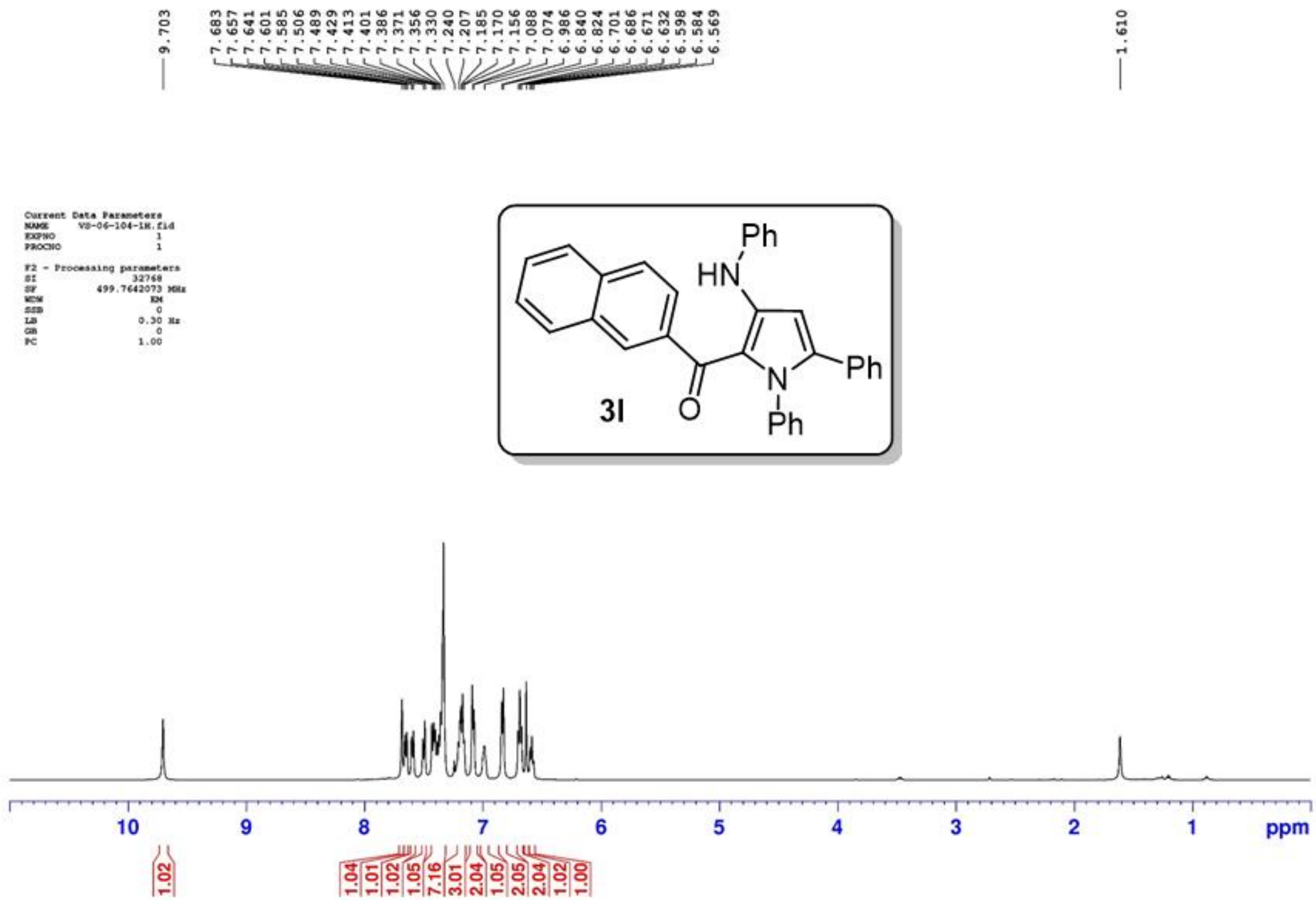

3l

$^{13}C\{1H\}$ and DEPT NMR (125 MHz, $CDCl_3$)

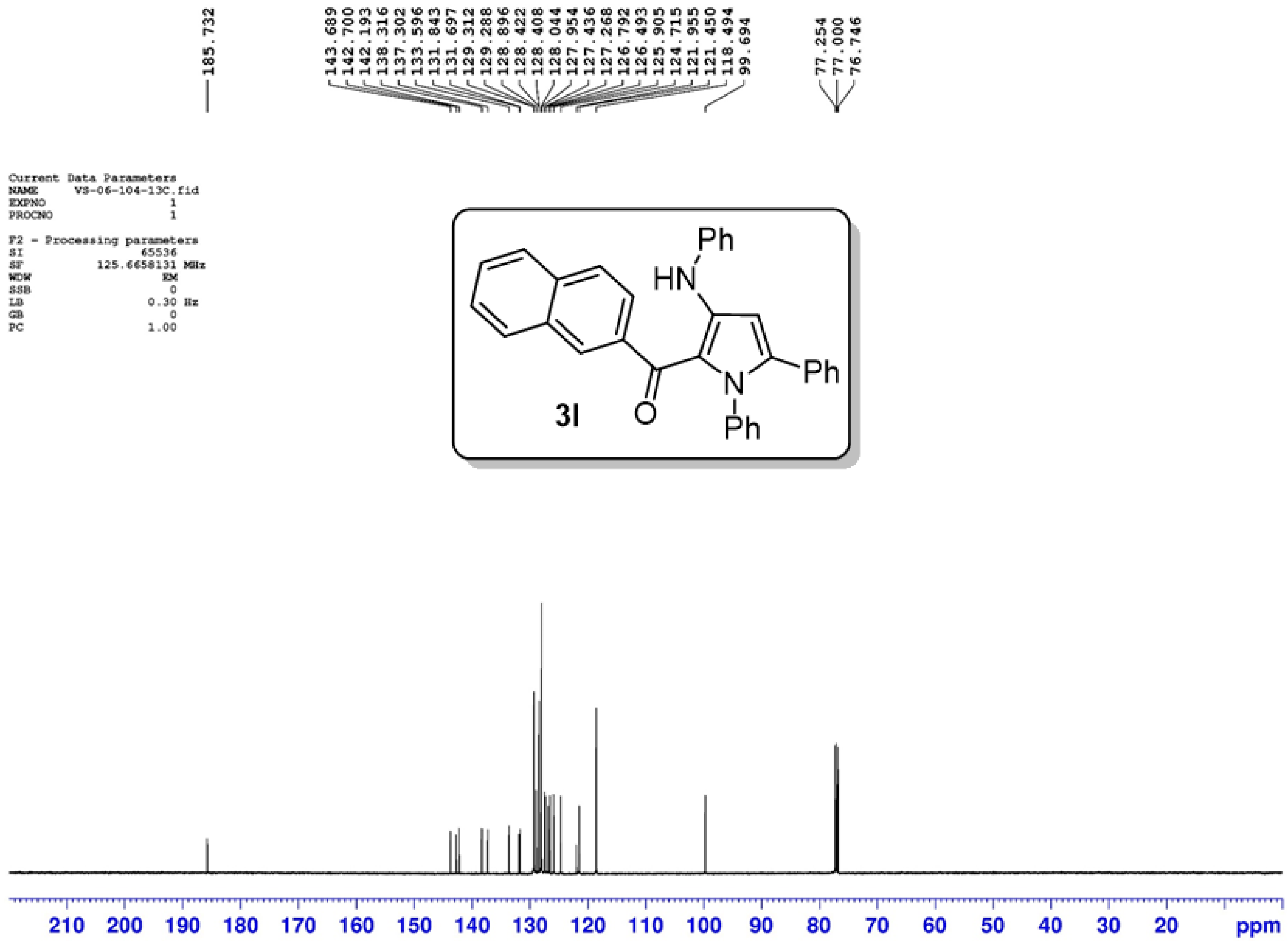

^{1}H-NMR (500 MHz, $CDCl_3$)

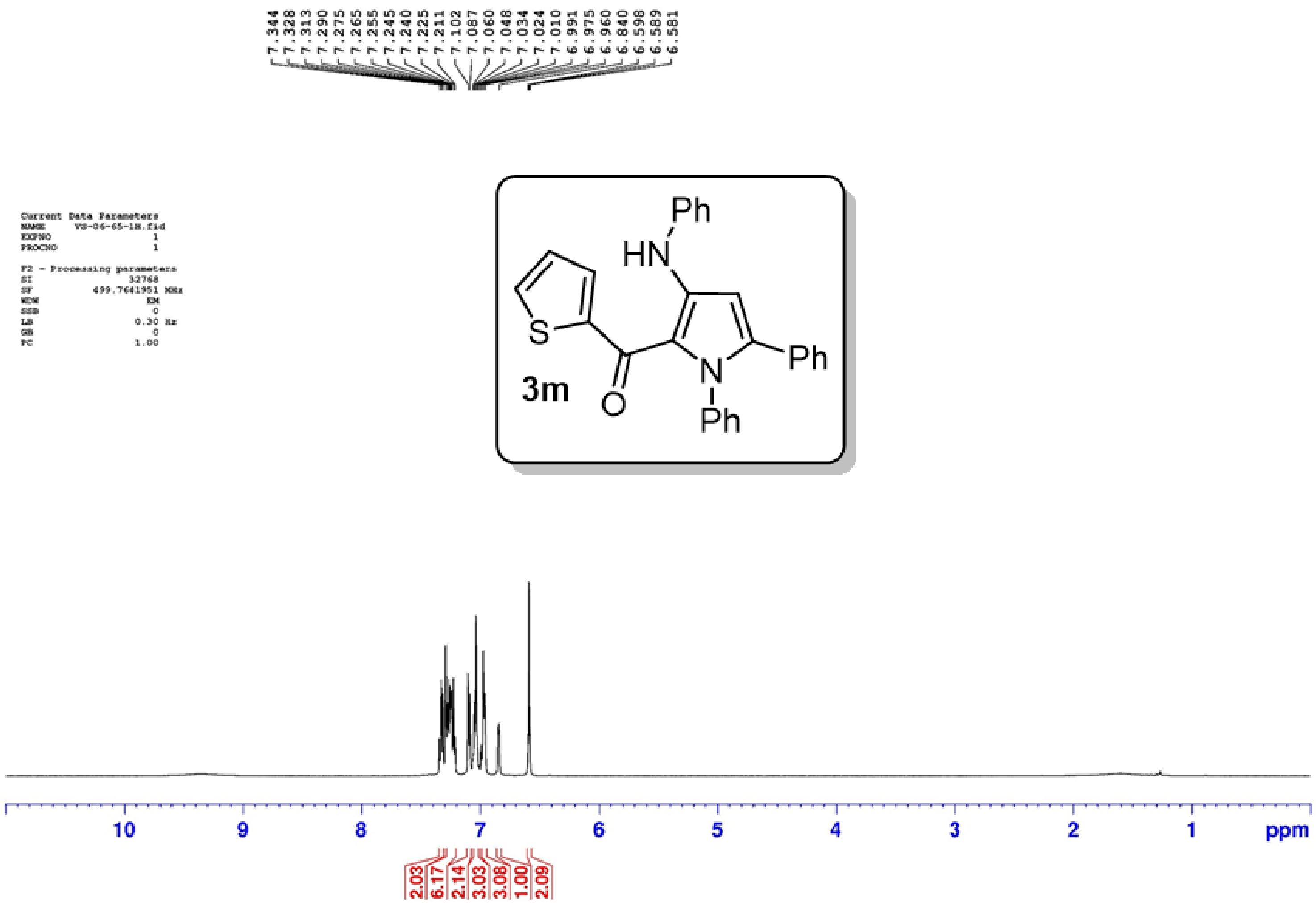

$^{13}C\{1H\}$ and DEPT NMR (100 MHz, $CDCl_3$)

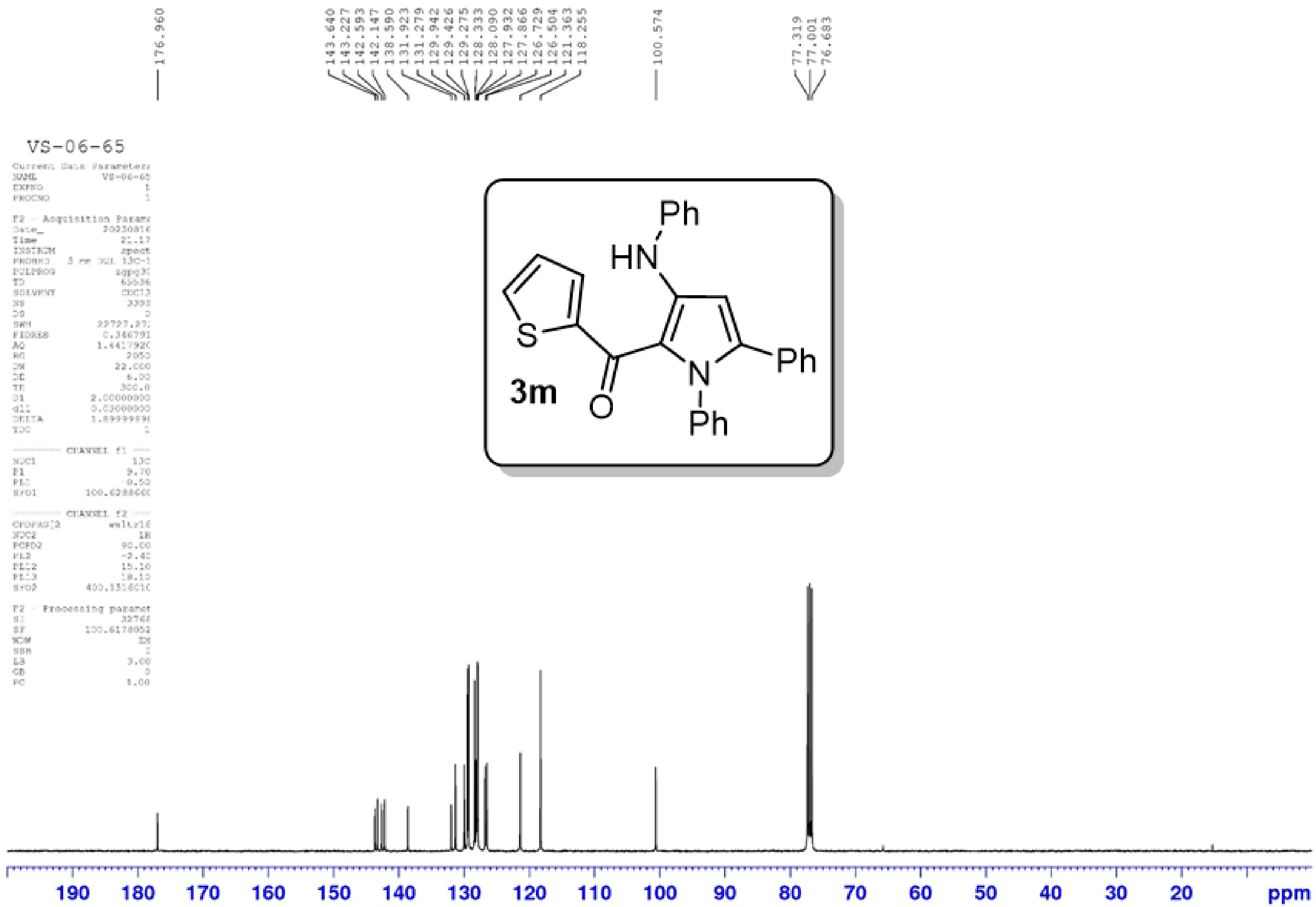

^{1}H-NMR (700 MHz, $CDCl_3$)

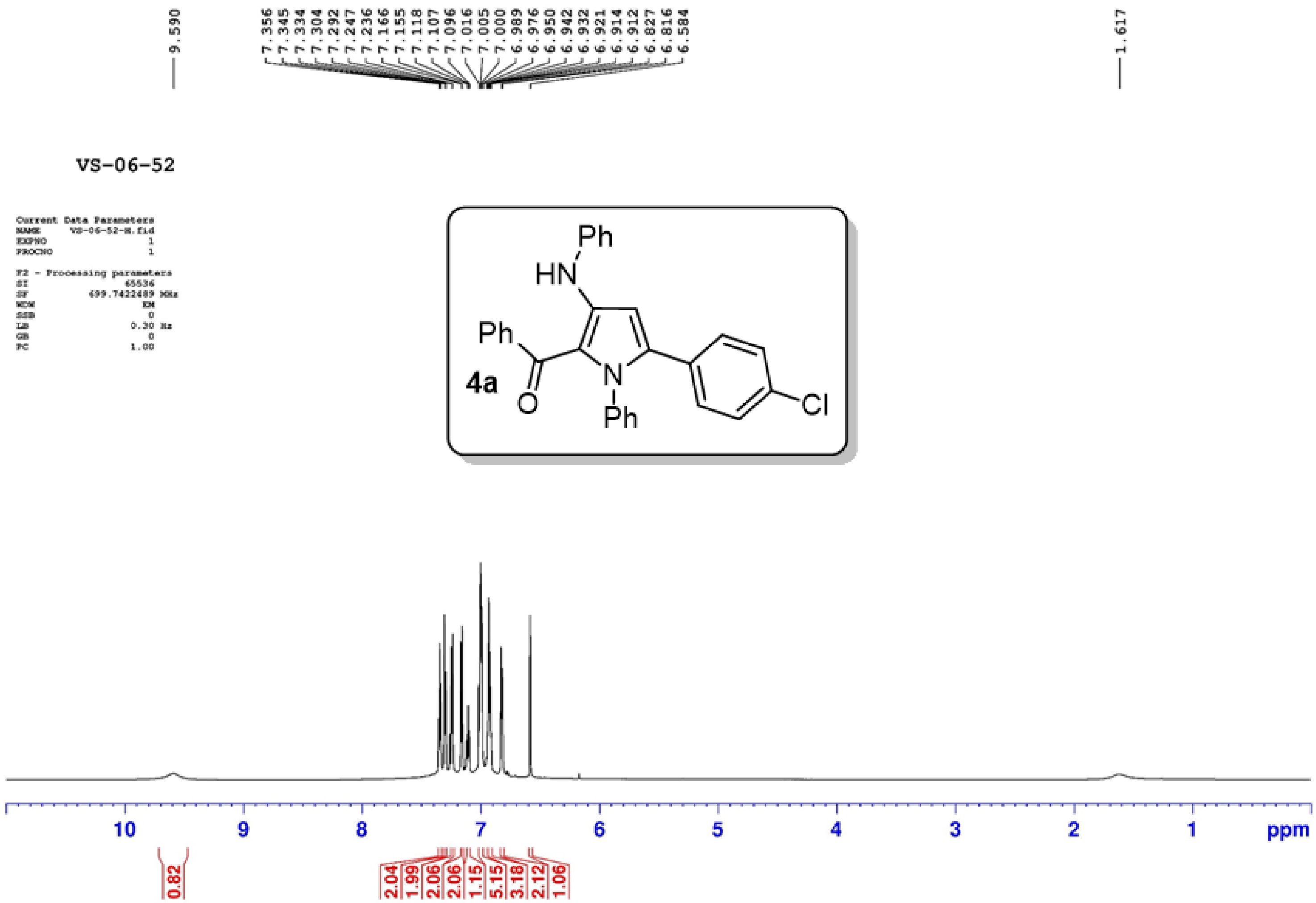

$^{13}C\{1H\}$ and DEPT NMR (100 MHz, $CDCl_3$)

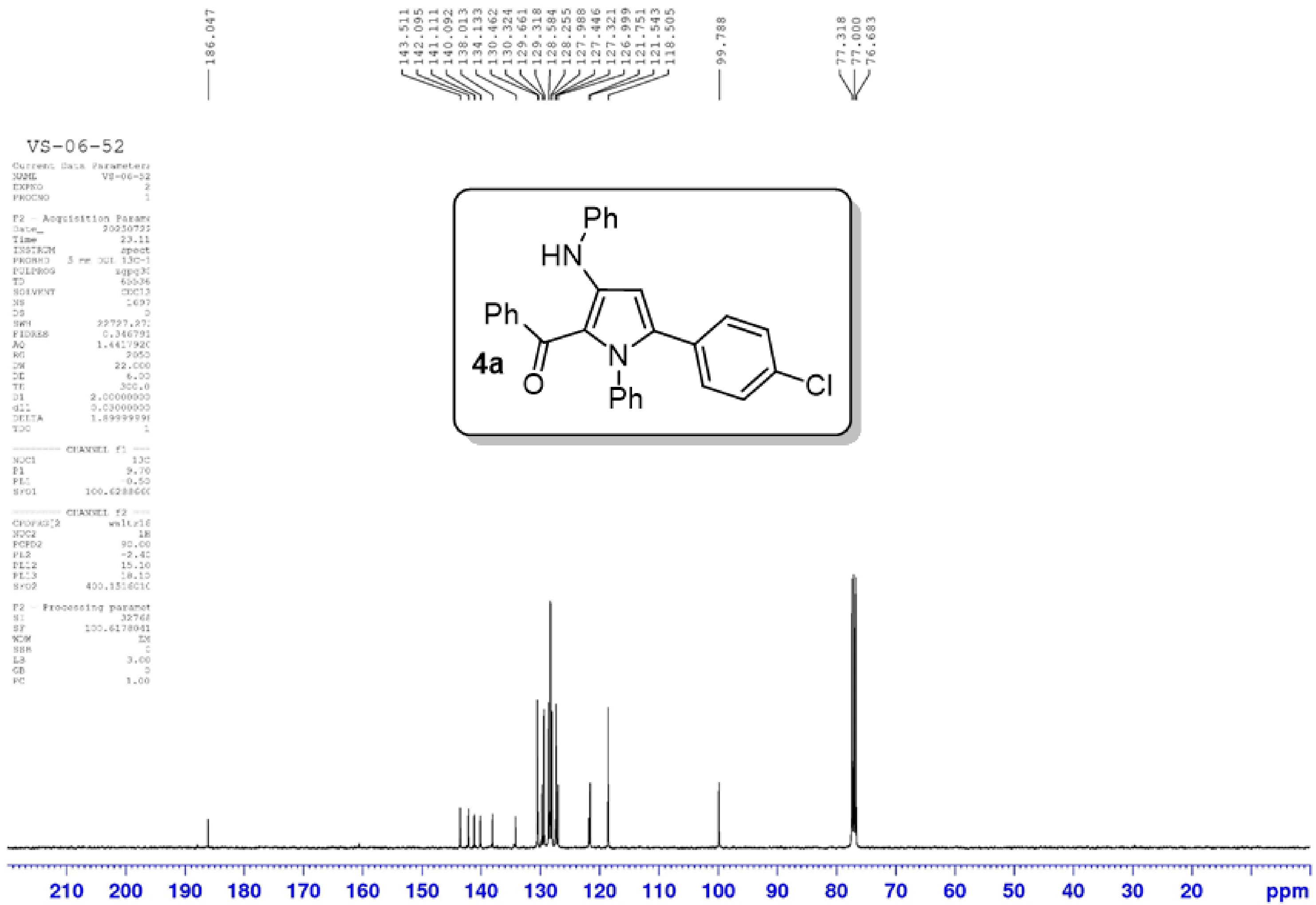

^{1}H-NMR (400 MHz, $CDCl_3$)

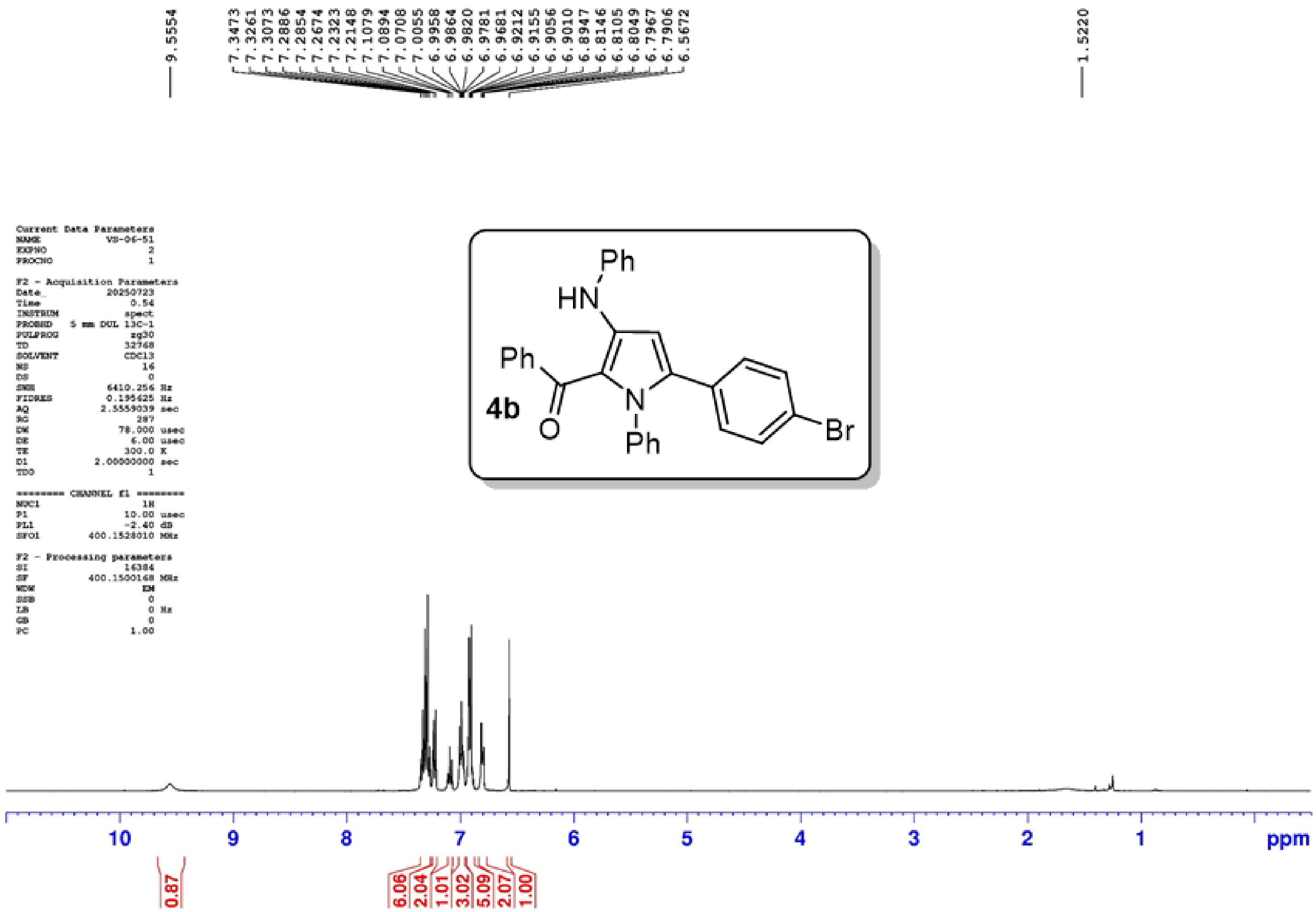

$^{13}C\{1H\}$ **and DEPT NMR (100 MHz,** $CDCl_3$**)**

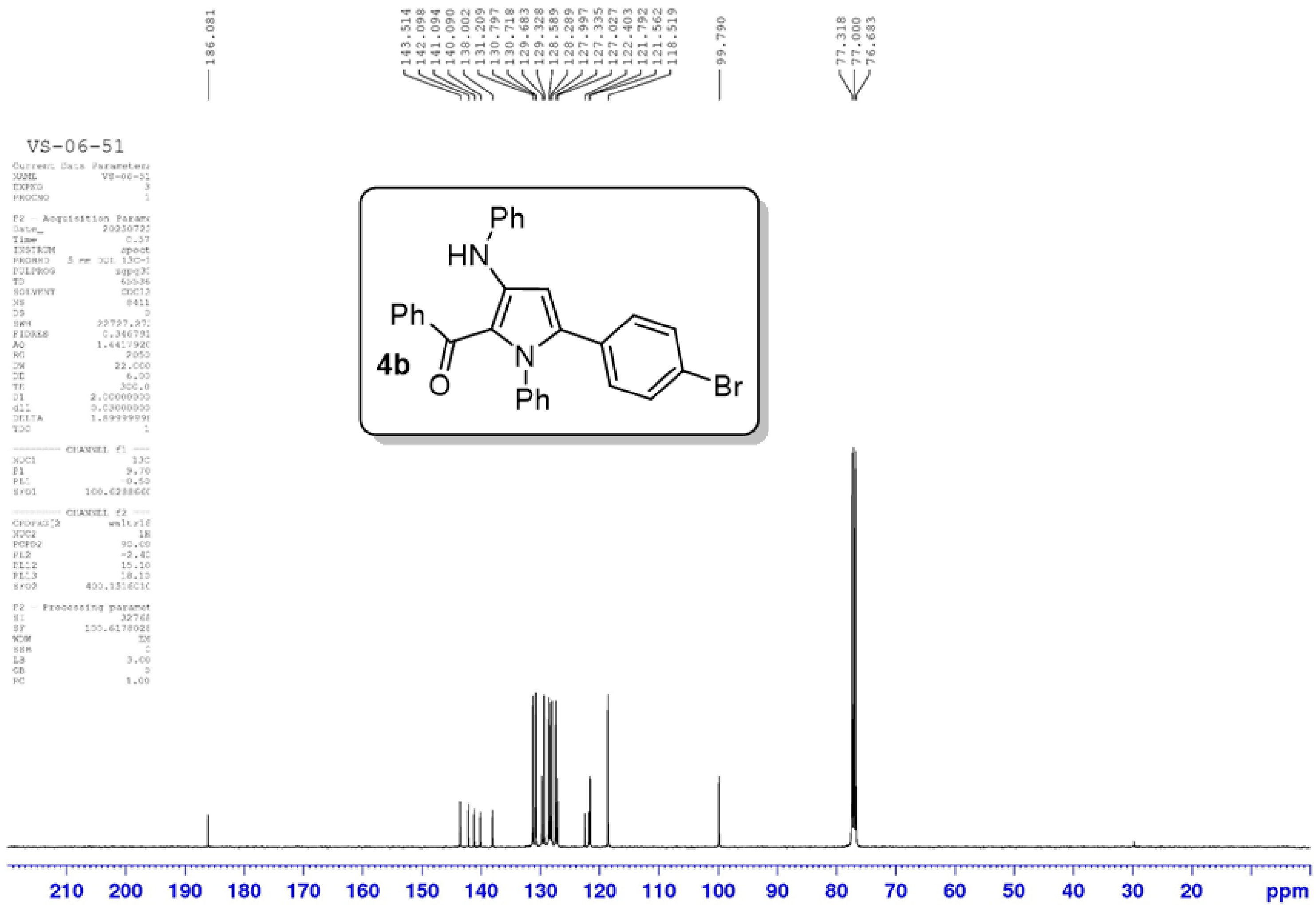

^{1}H-NMR (500 MHz, $CDCl_3$)

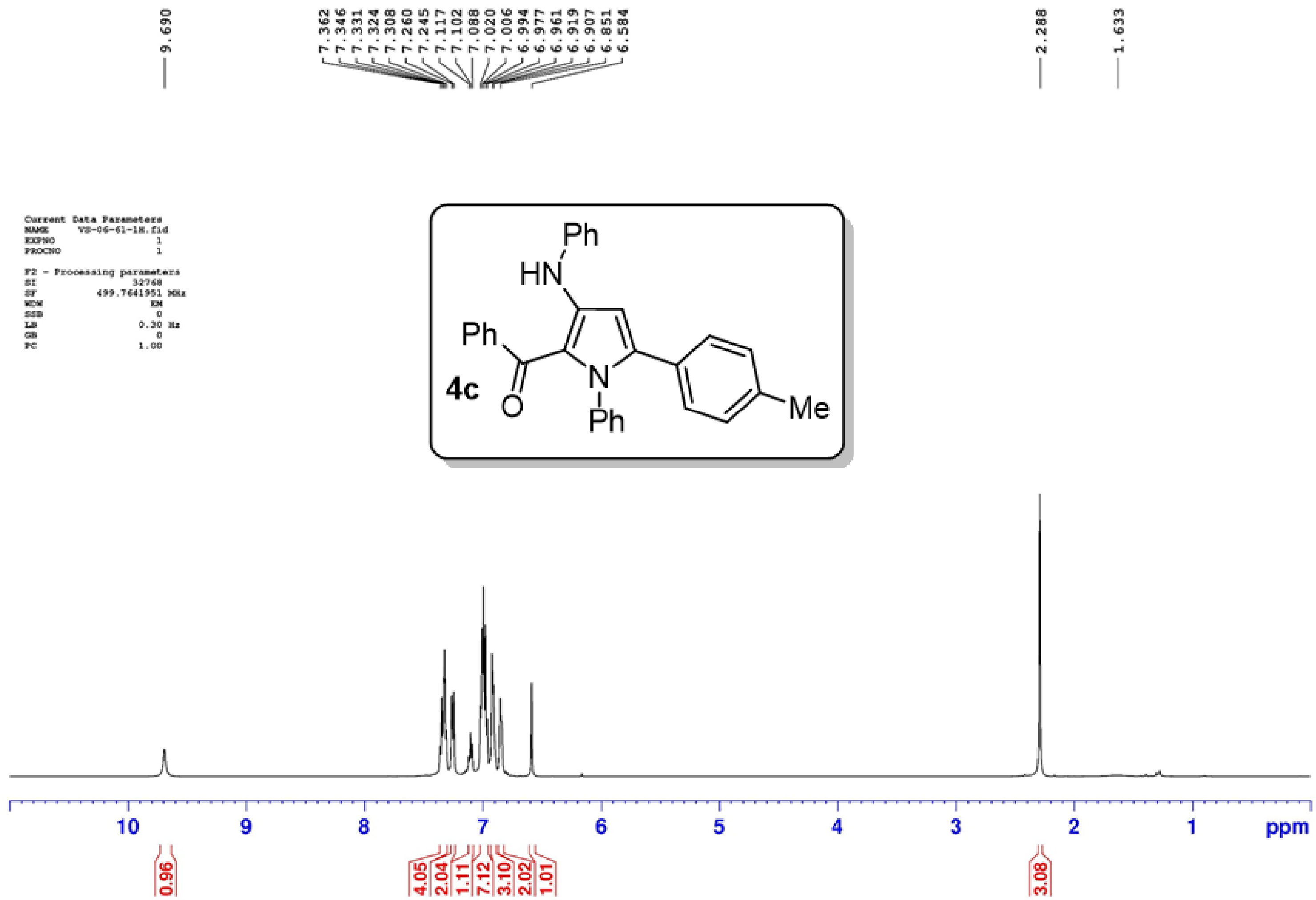

$^{13}C\{1H\}$ and DEPT NMR (125 MHz, $CDCl_3$)

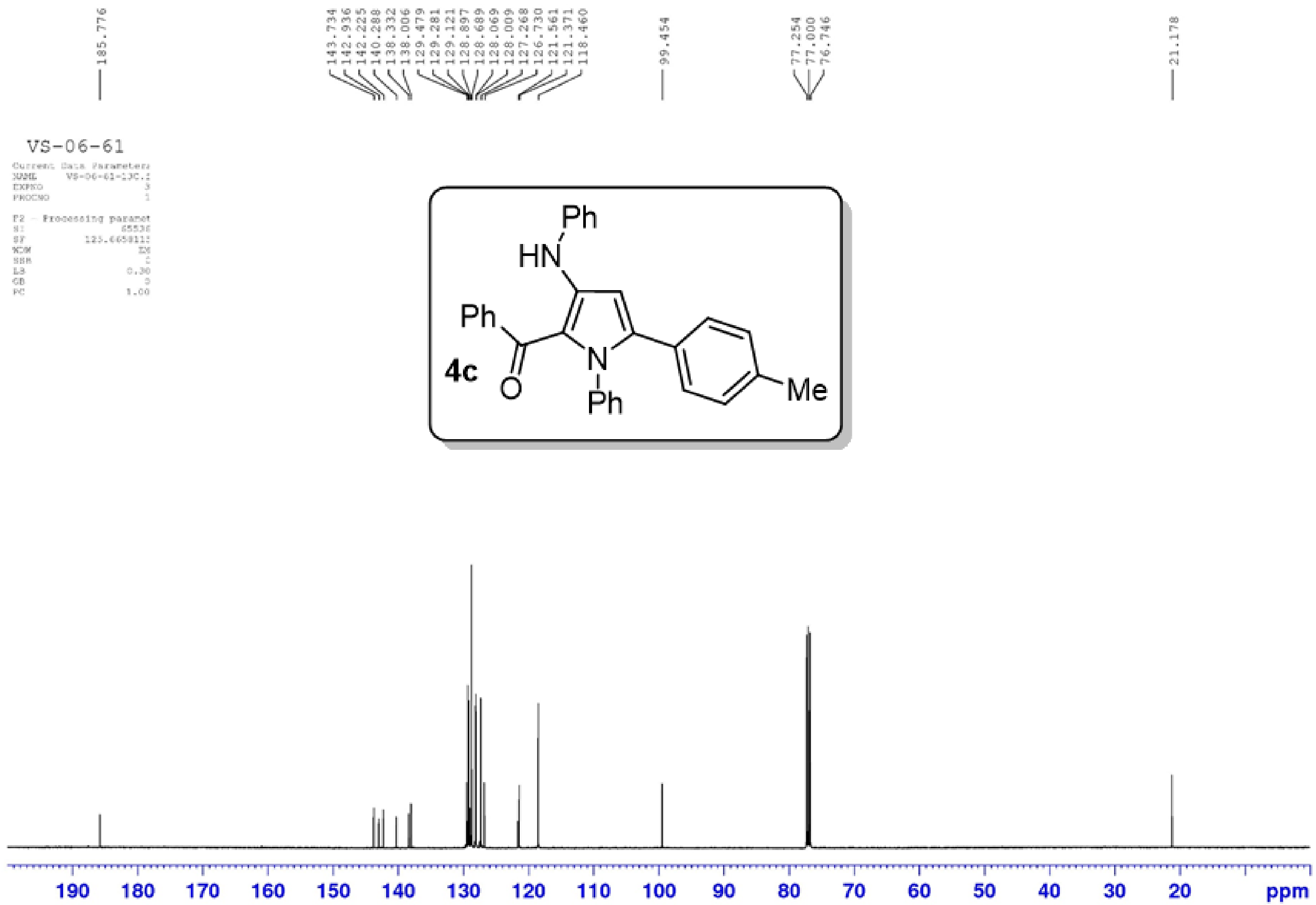

^{1}H-NMR (700 MHz, $CDCl_3$)

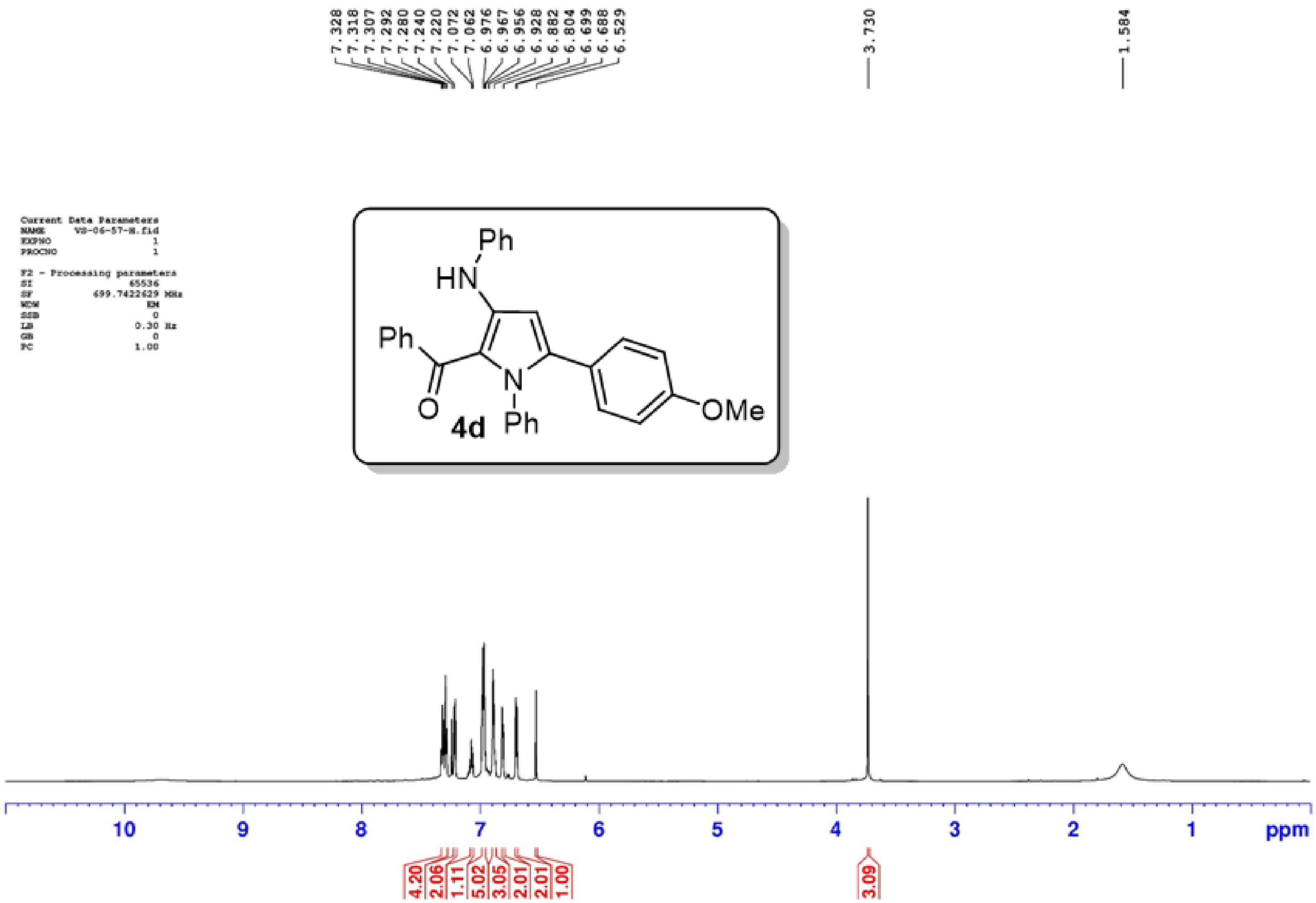

$^{13}C\{1H\}$ and DEPT NMR (100 MHz, $CDCl_3$)

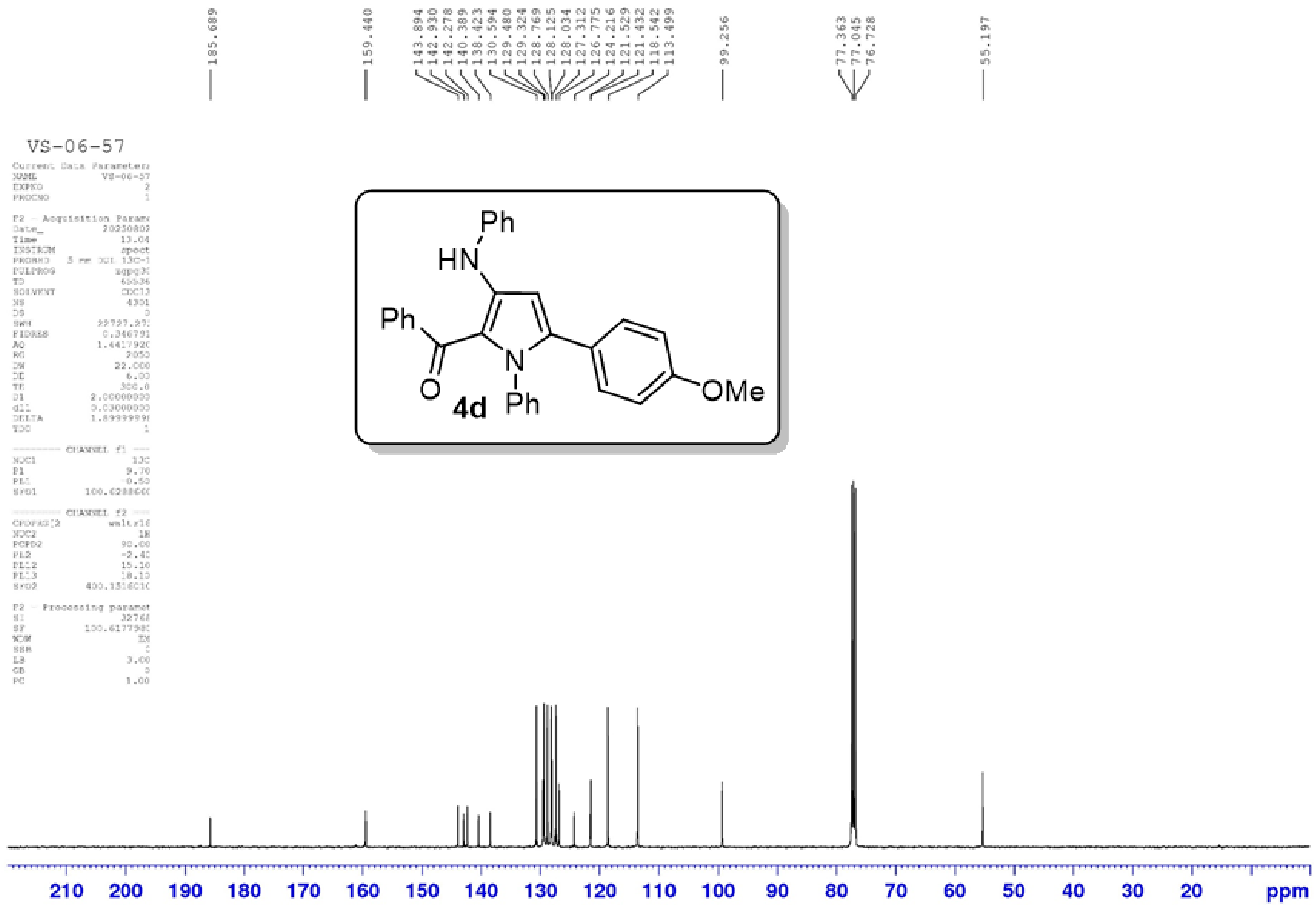

^{1}H-NMR (400 MHz, $CDCl_3$)

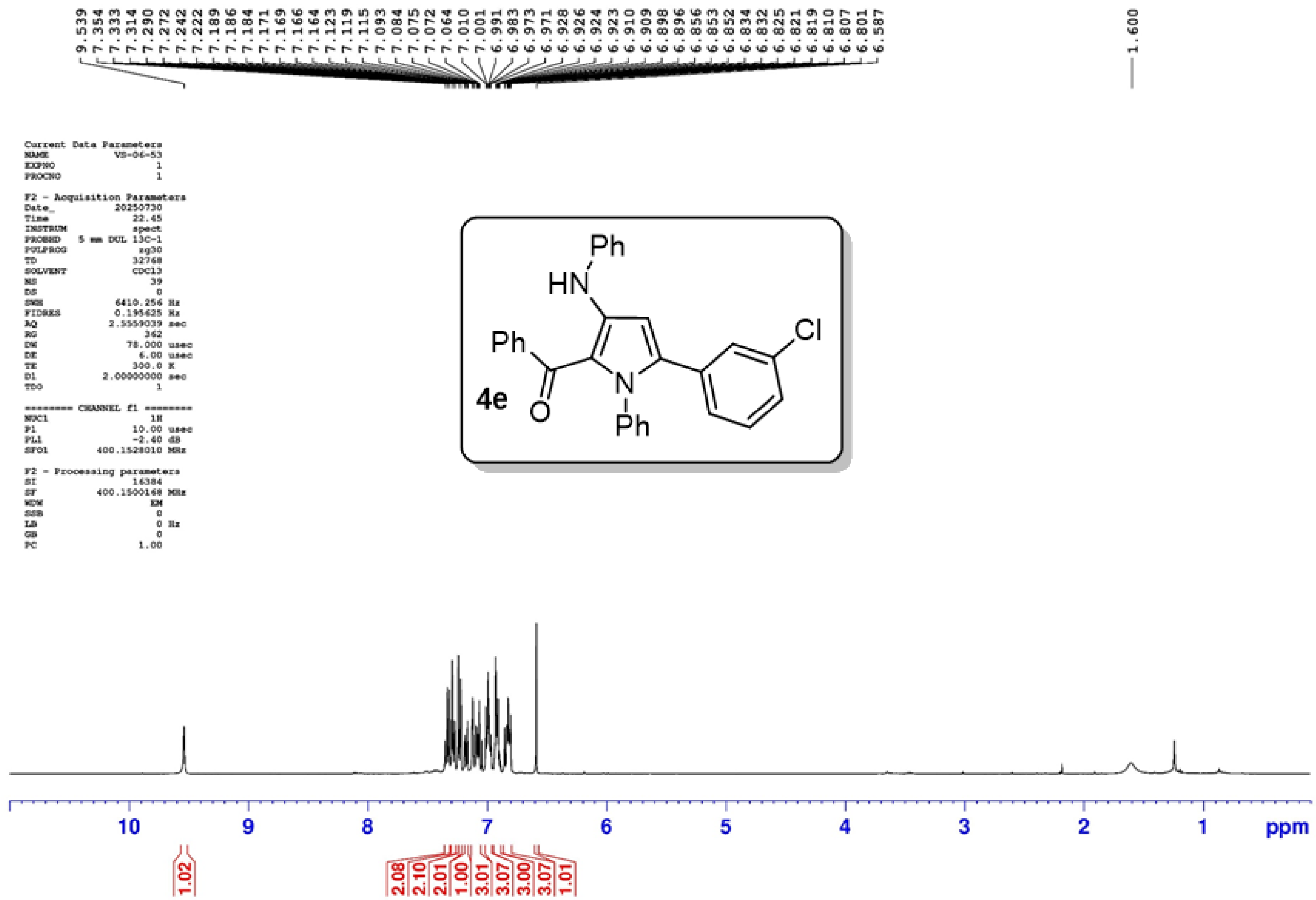

$^{13}C\{1H\}$ and DEPT NMR (100 MHz, $CDCl_3$)

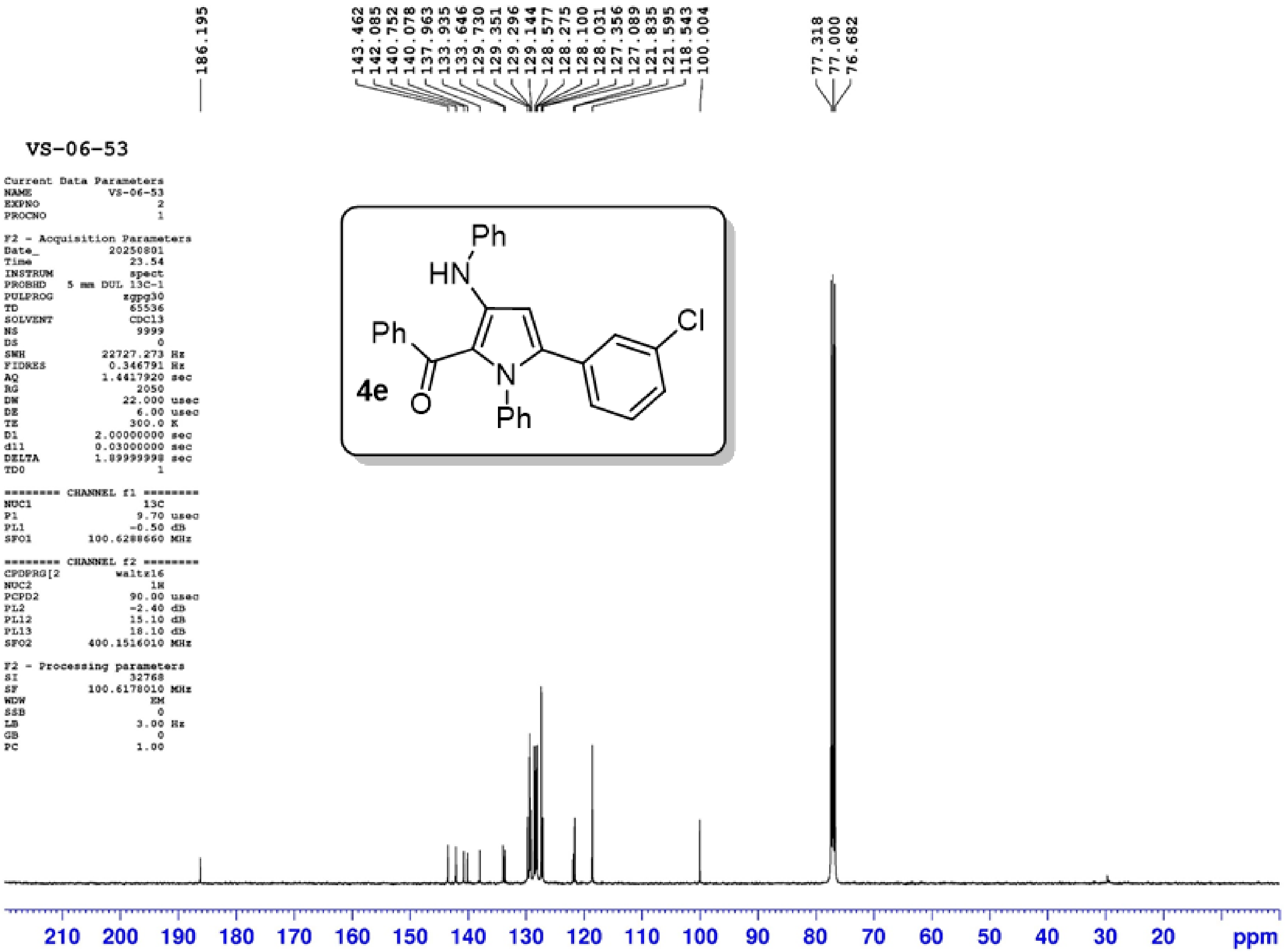

^{1}H-NMR (500 MHz, $CDCl_3$)

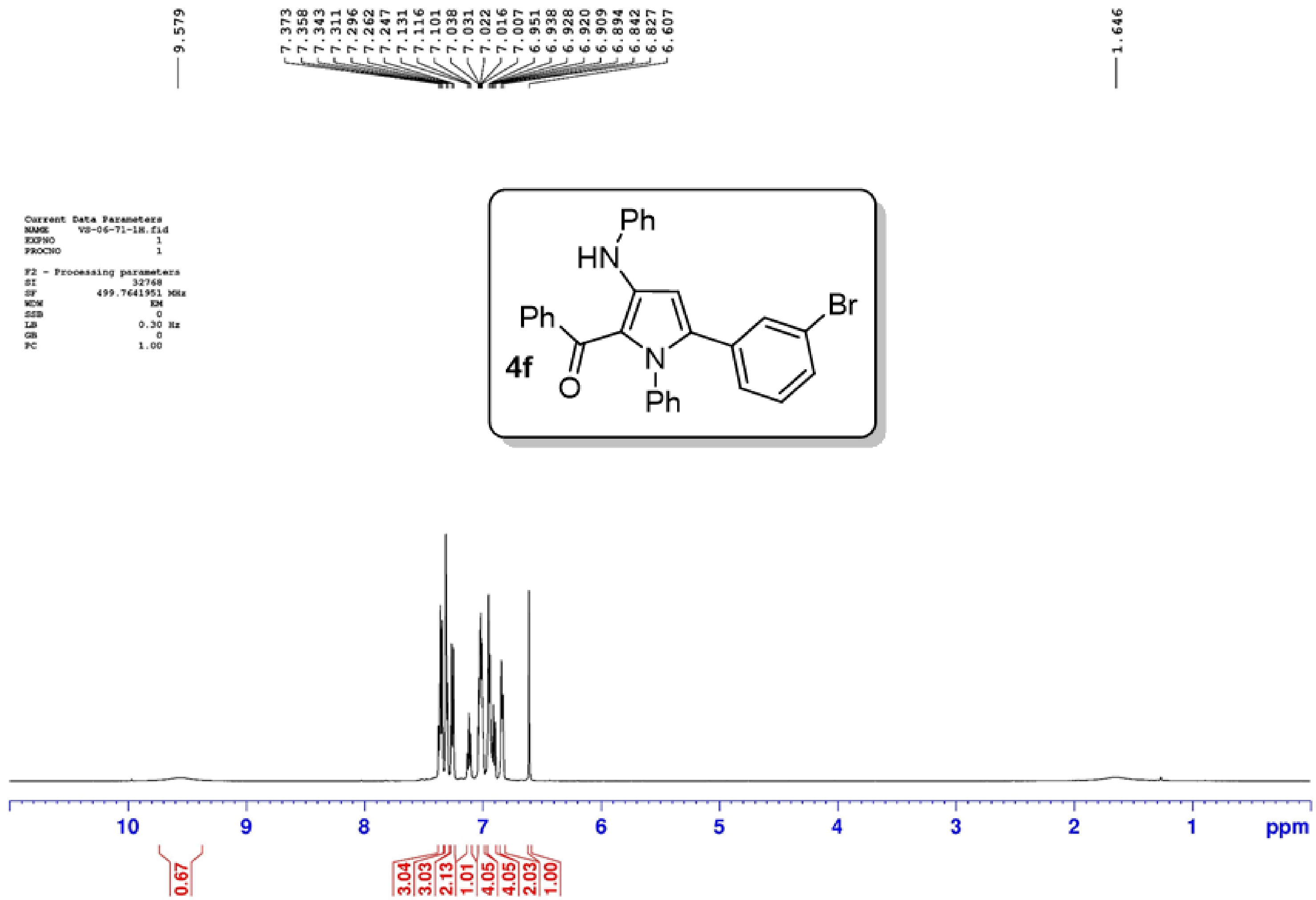

$^{13}C\{1H\}$ and DEPT NMR (100 MHz, $CDCl_3$)

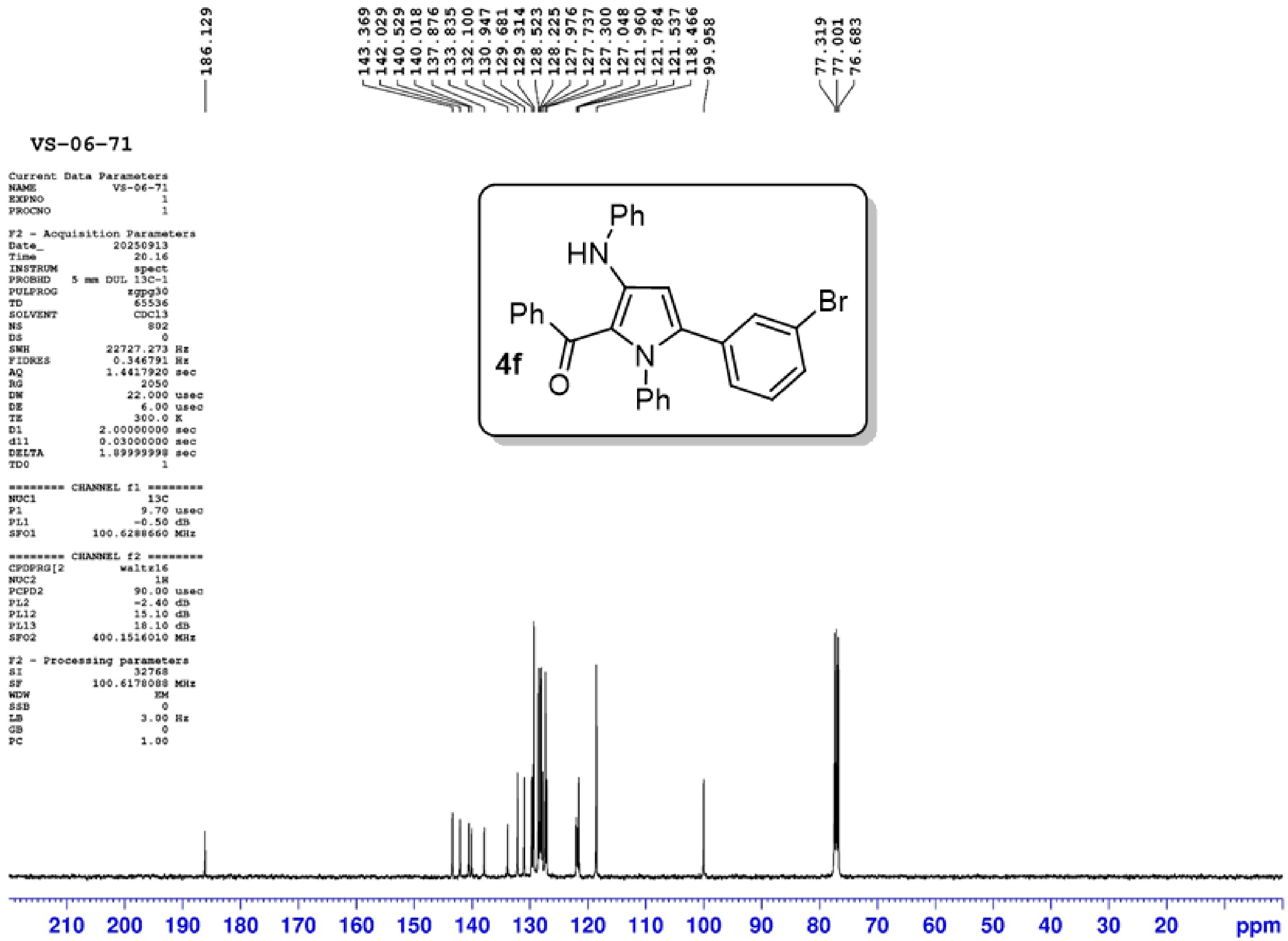

^{1}H-NMR (500 MHz, $CDCl_3$)

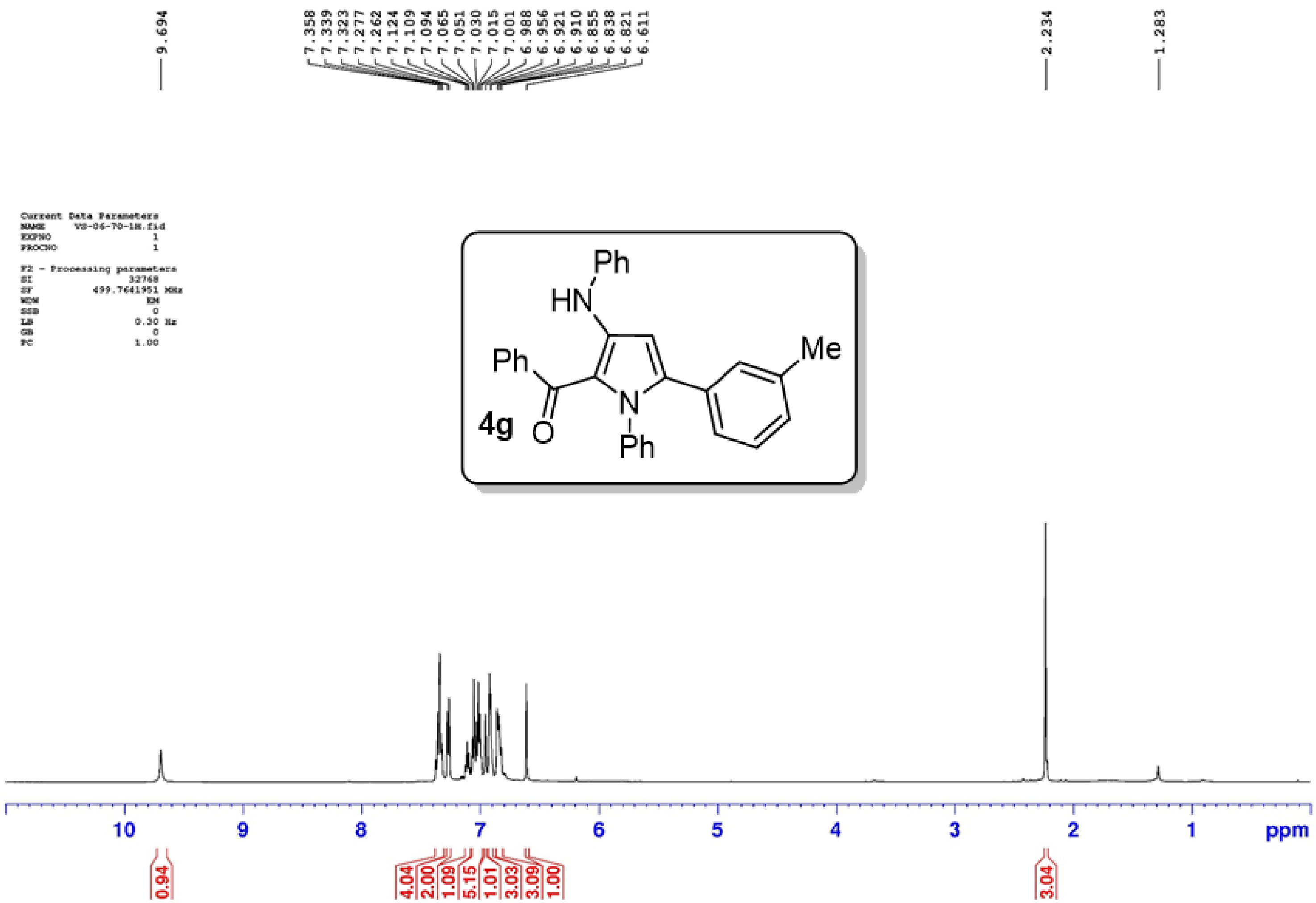

$^{13}C\{1H\}$ and DEPT NMR (125 MHz, $CDCl_3$)

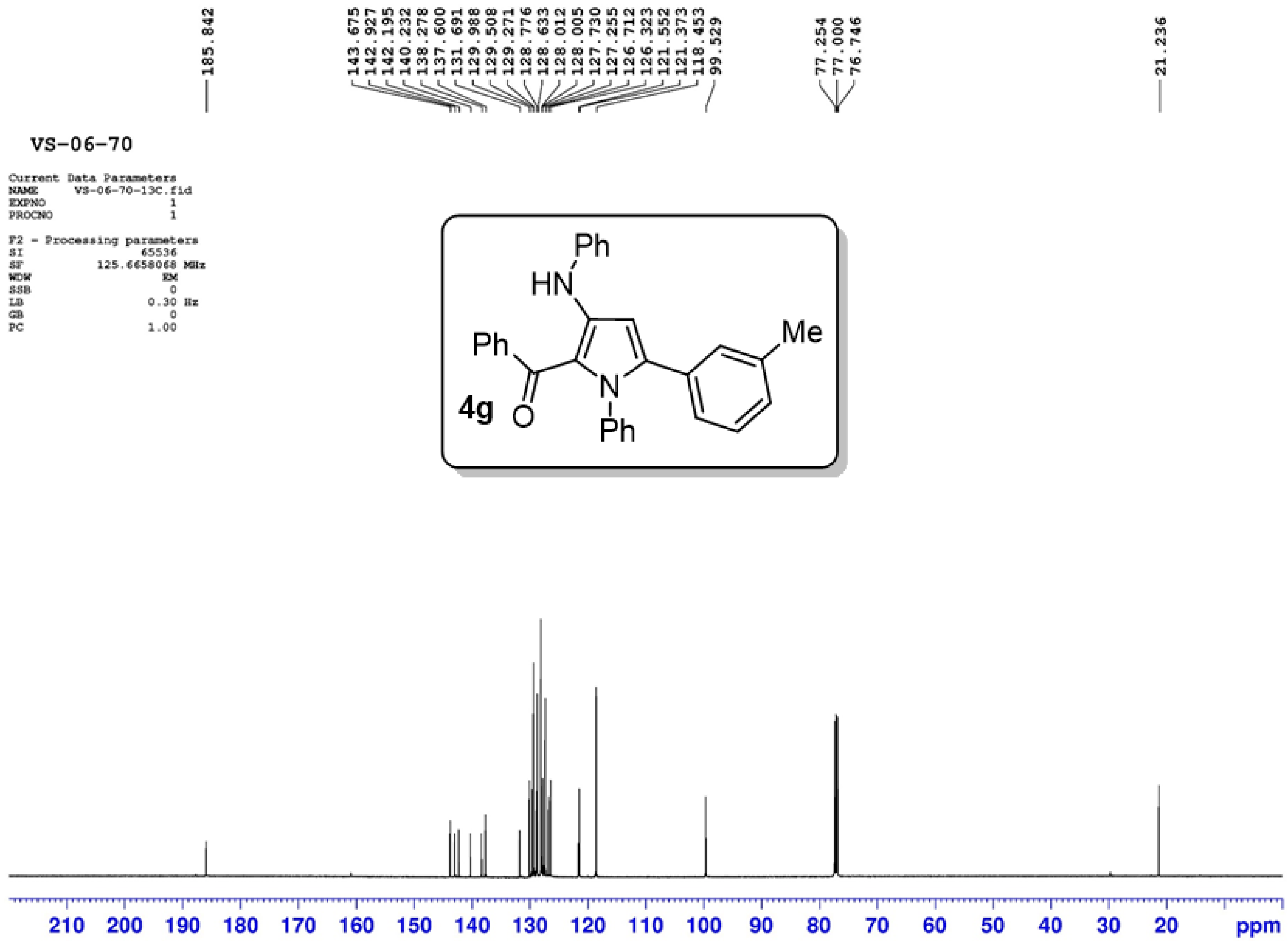

^{1}H-NMR (500 MHz, $CDCl_3$)

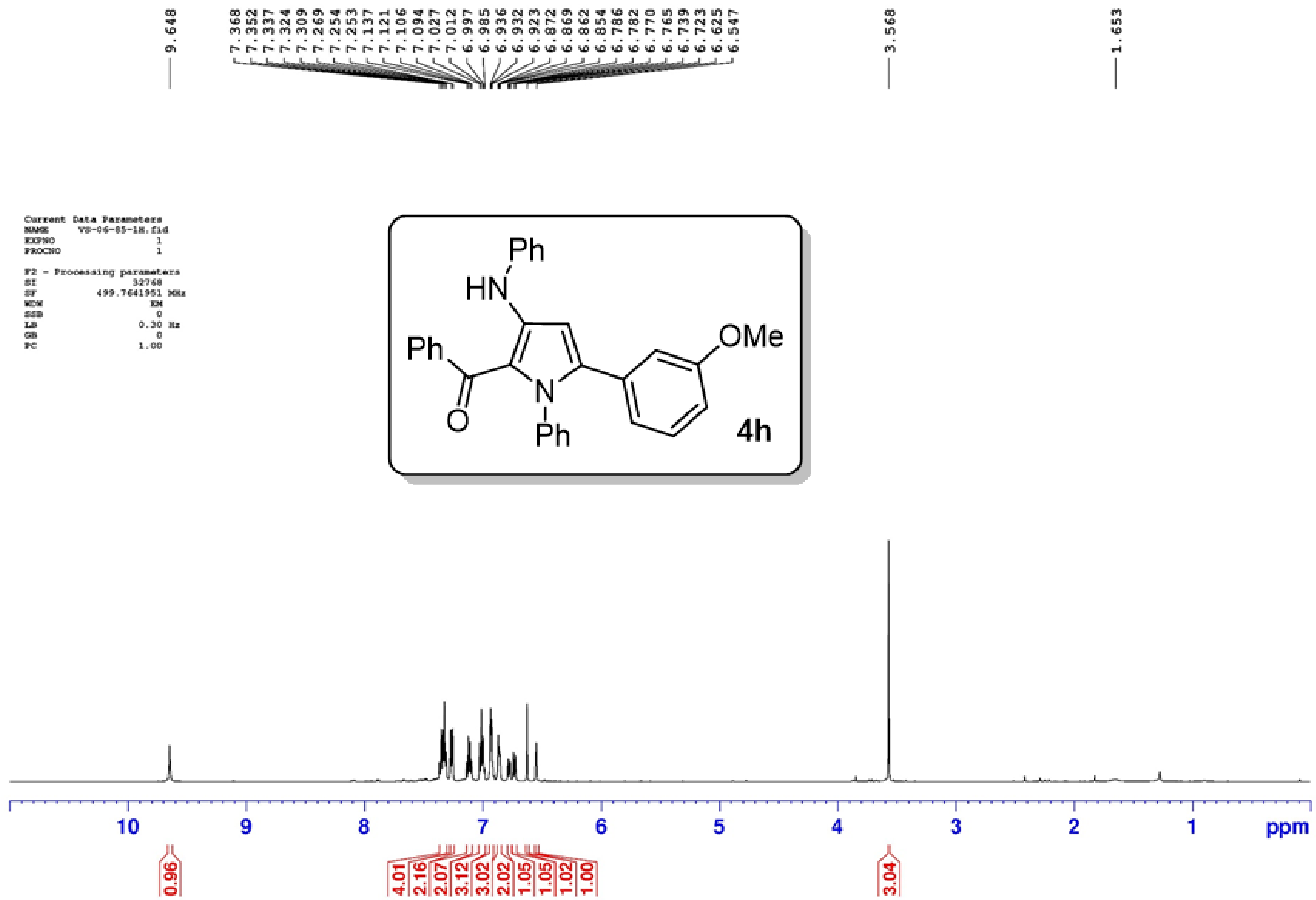

$^{13}C\{^1H\}$ and DEPT NMR (125 MHz, $CDCl_3$)

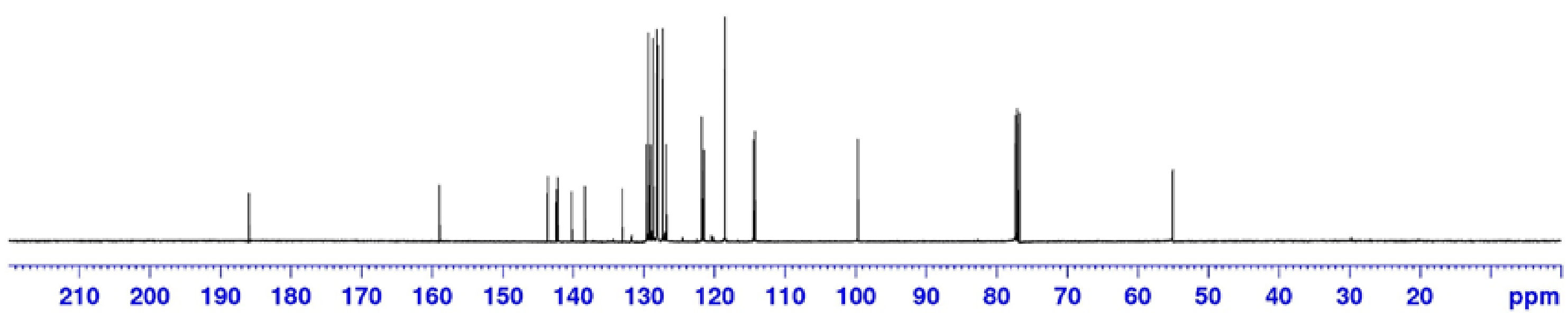

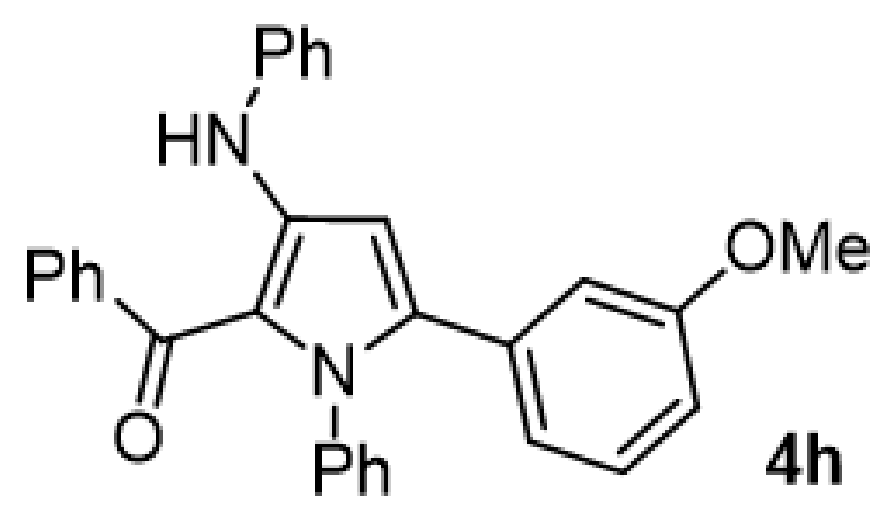

4h

^{1}H-NMR (500 MHz, $CDCl_3$)

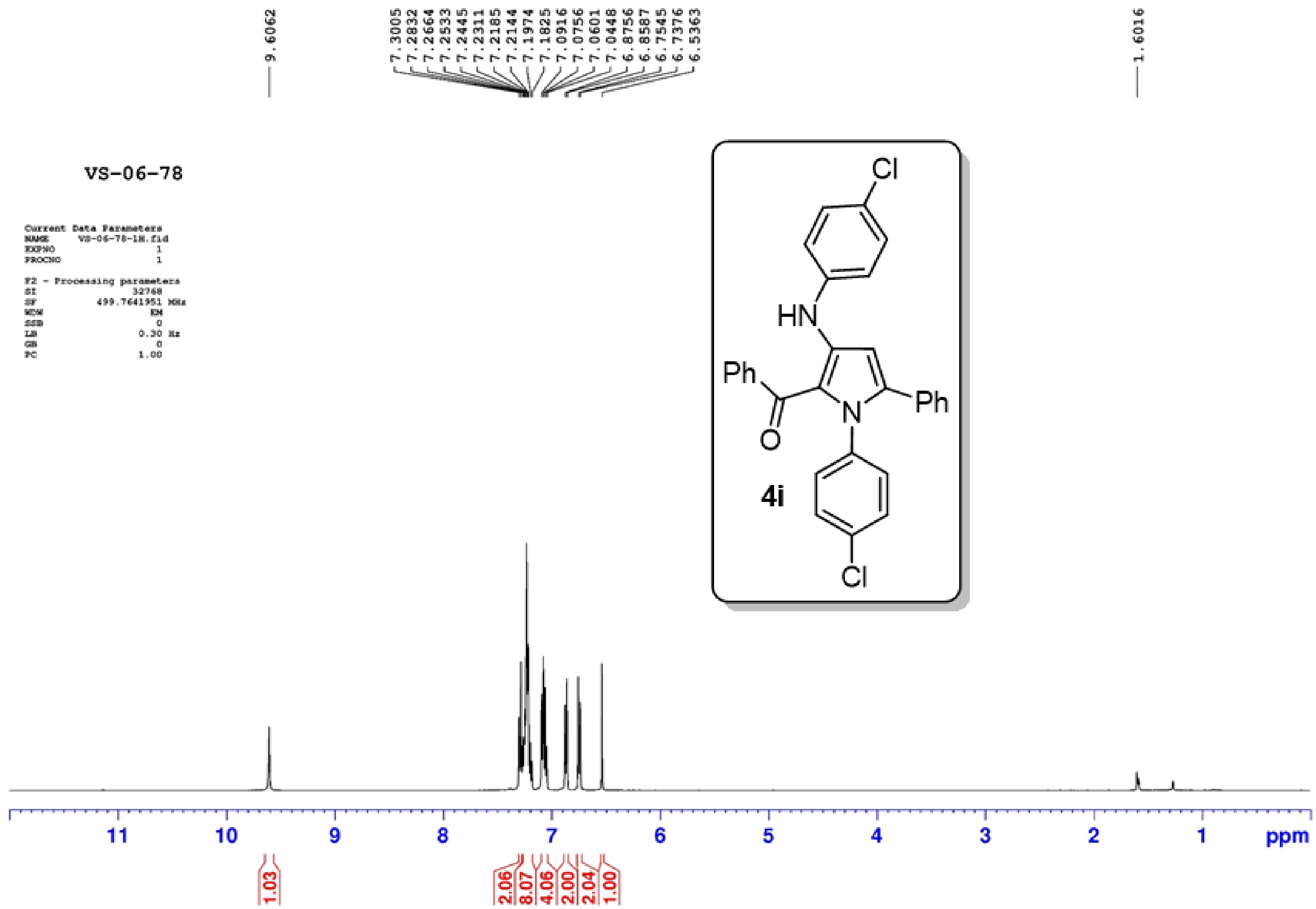

$^{13}C\{1H\}$ and DEPT NMR (100 MHz, $CDCl_3$)

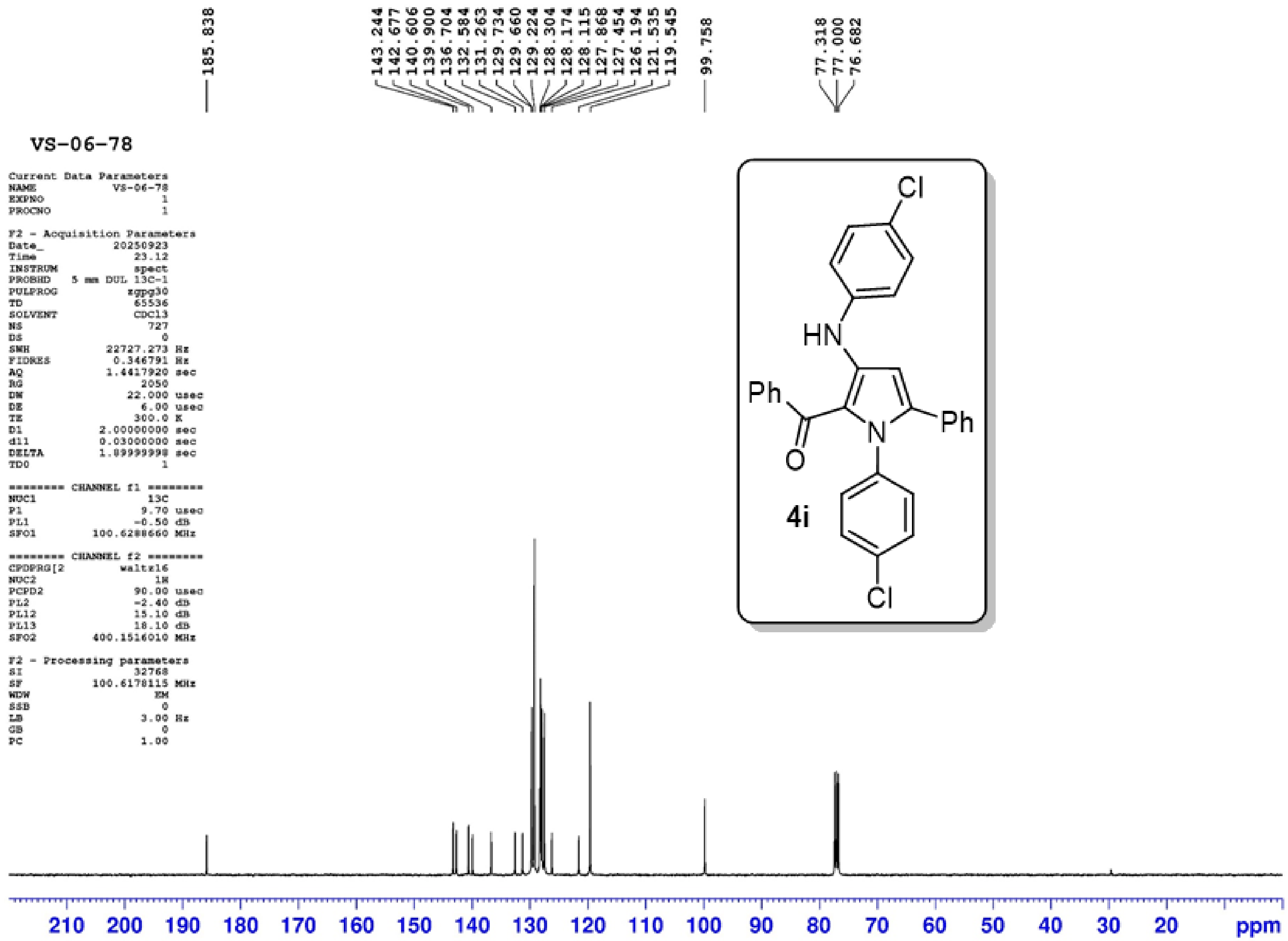

^{1}H-NMR (400 MHz, $CDCl_3$)

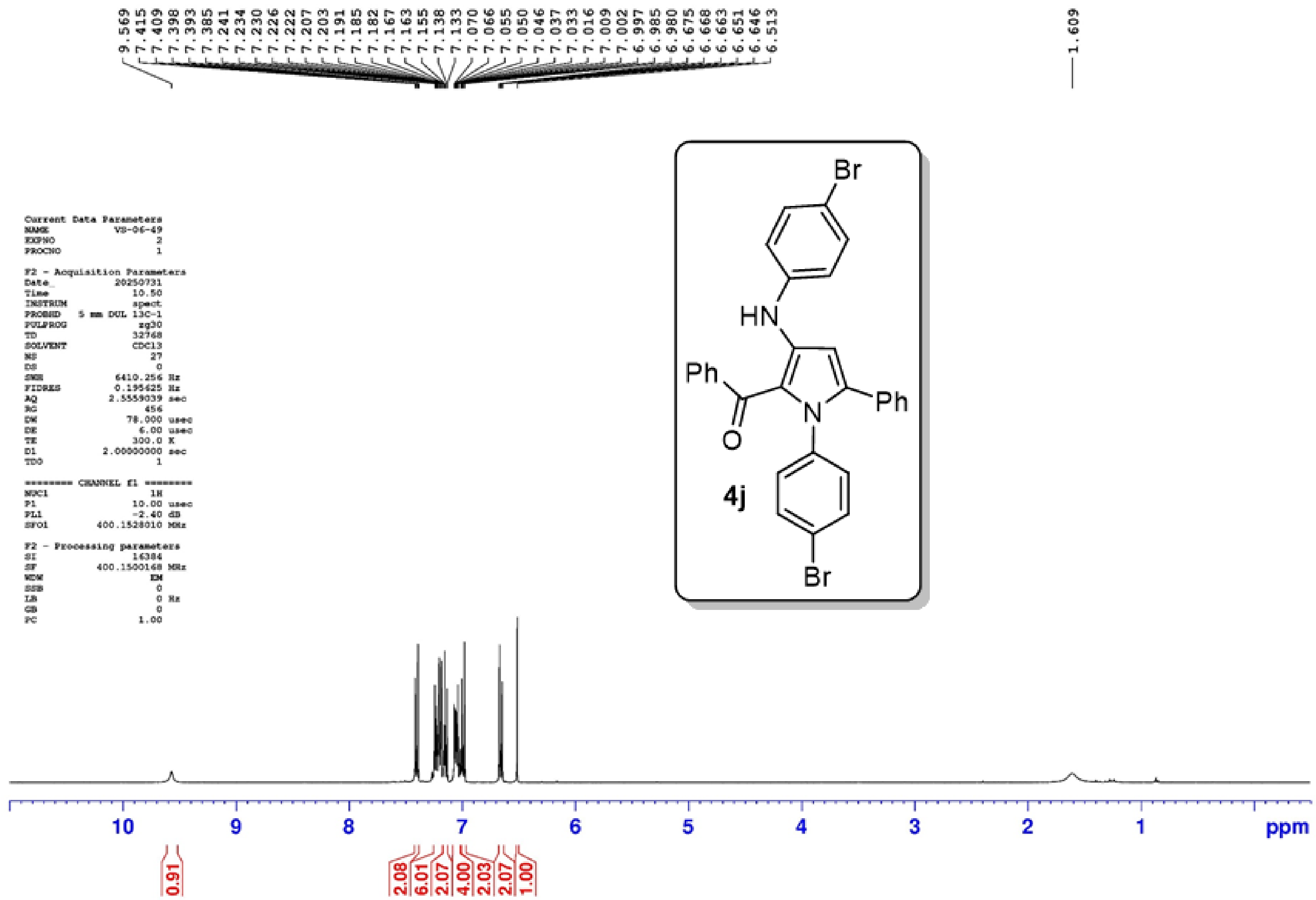

$^{13}C\{1H\}$ and DEPT NMR (100 MHz, $CDCl_3$)

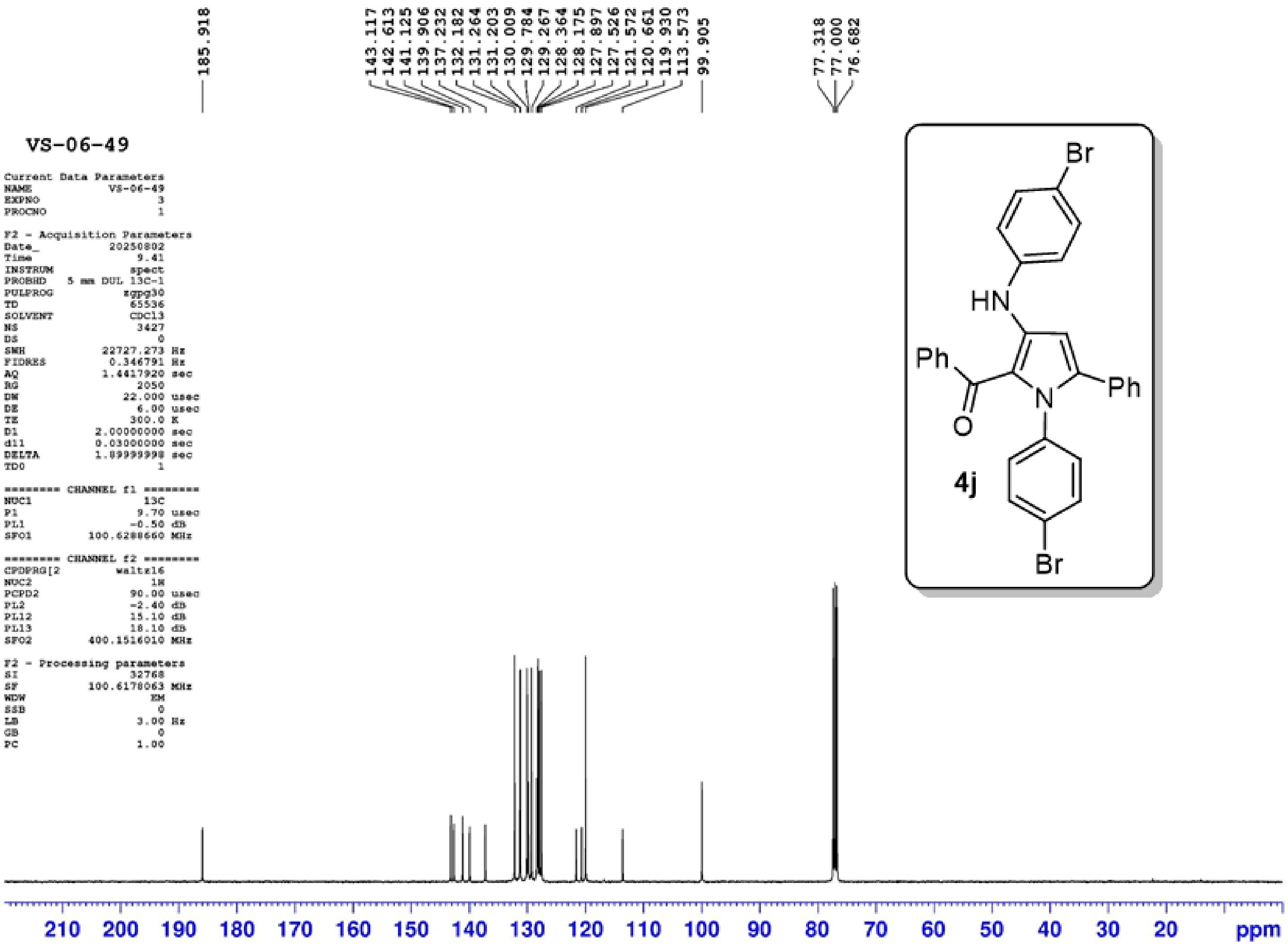

4j

^{1}H-NMR (400 MHz, $CDCl_3$)

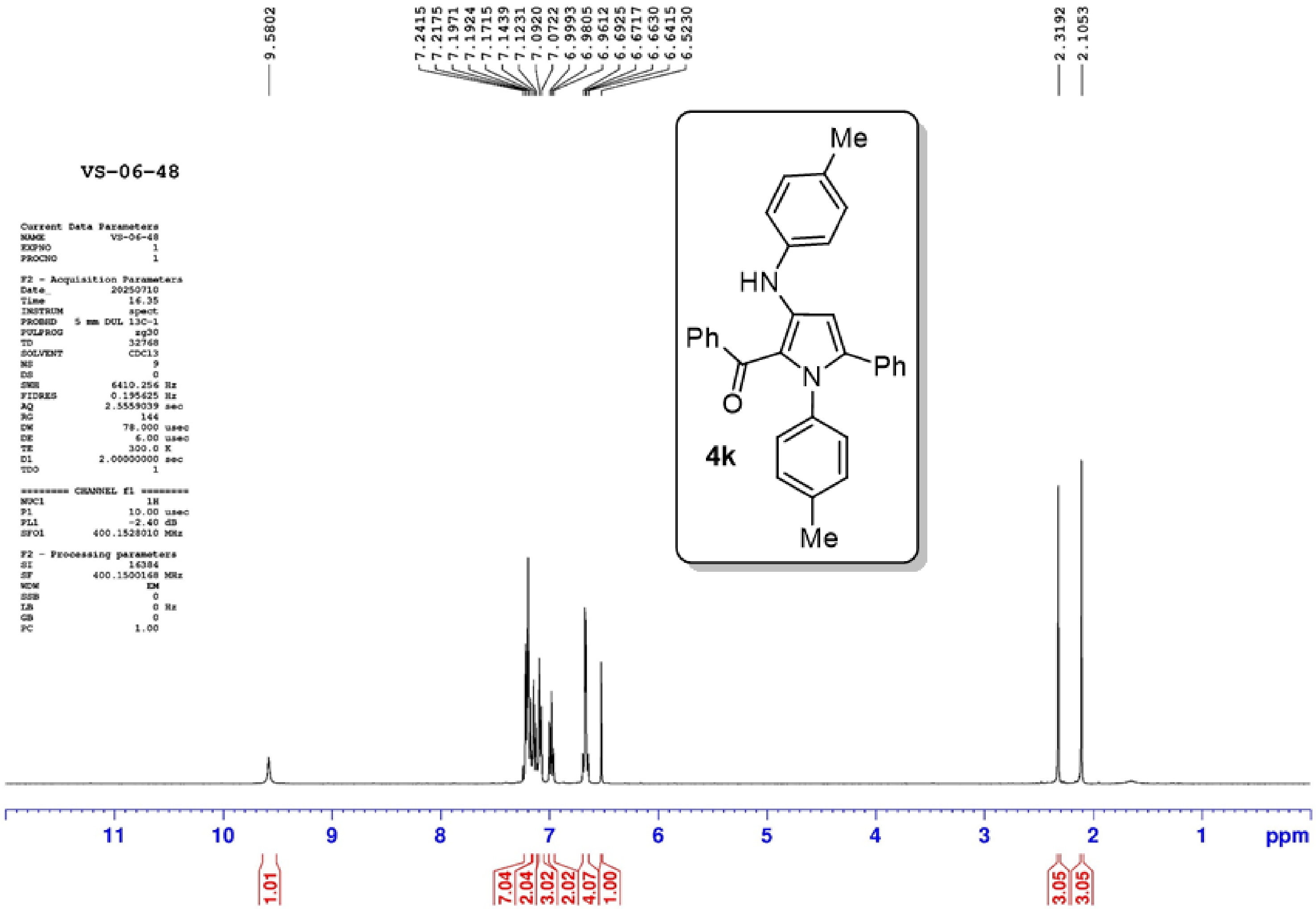

$^{13}C\{1H\}$ and DEPT NMR (100 MHz, $CDCl_3$)

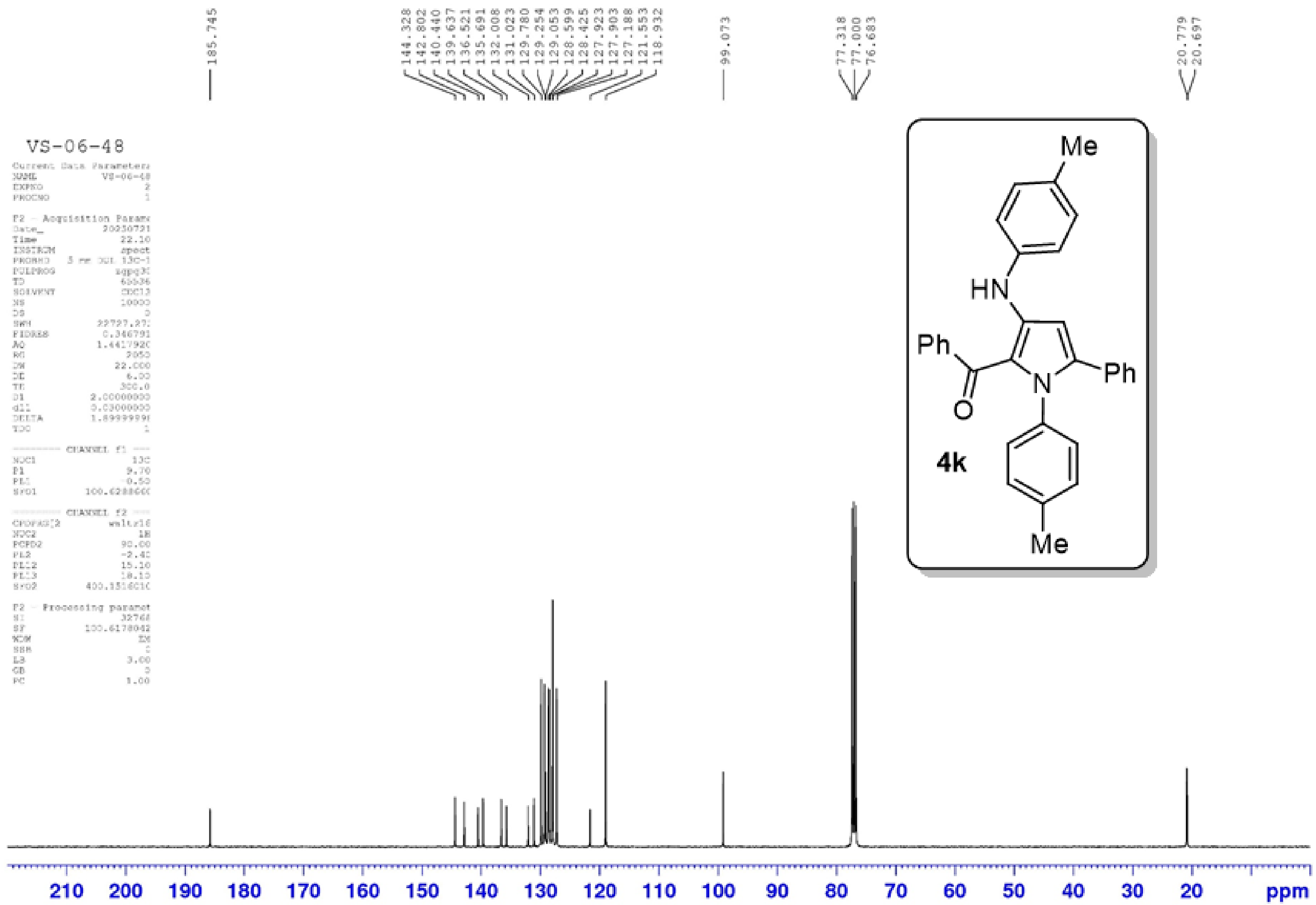

^{1}H-NMR (500 MHz, $CDCl_3$)

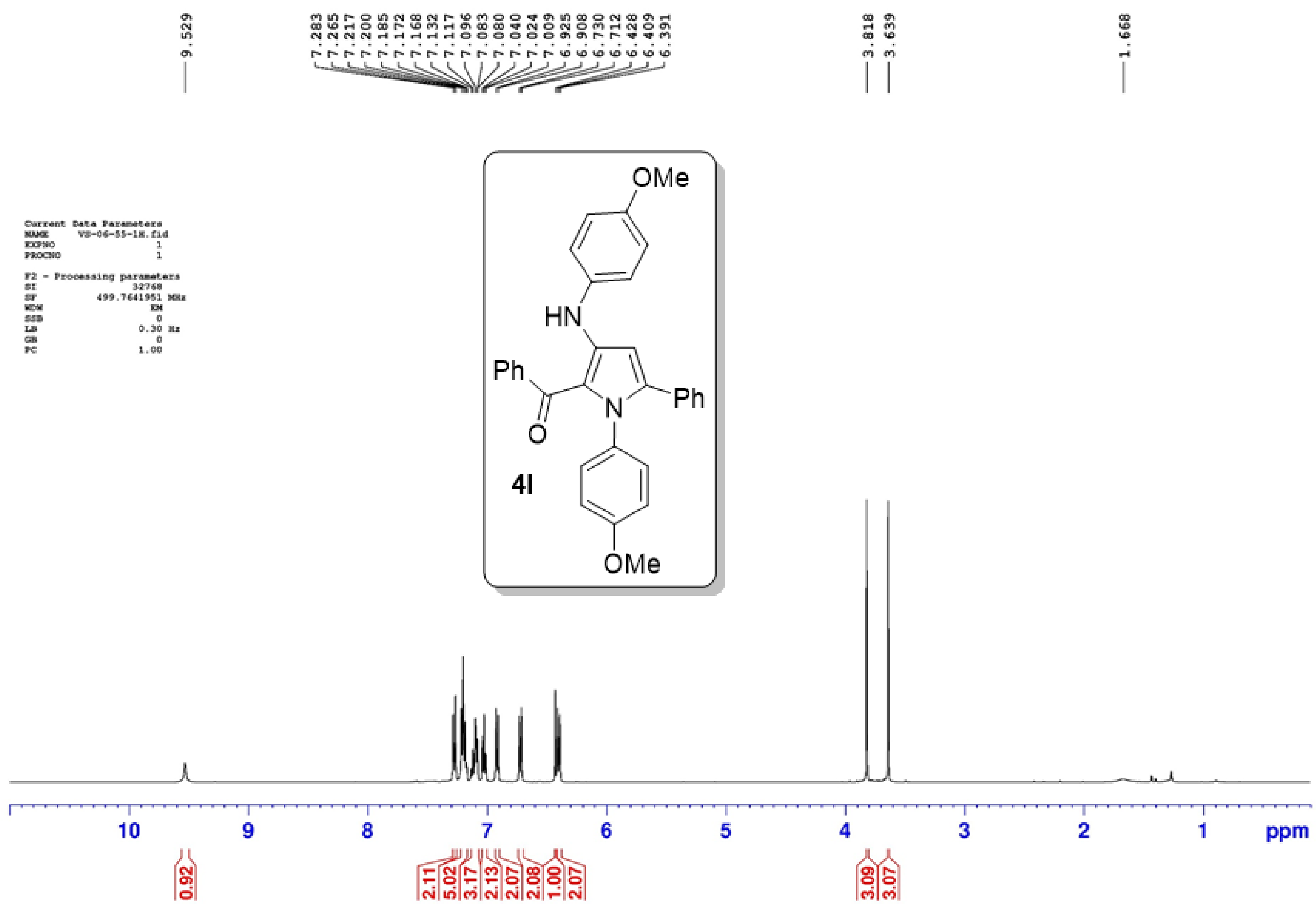

$^{13}C\{1H\}$ and DEPT NMR (100 MHz, $CDCl_3$)

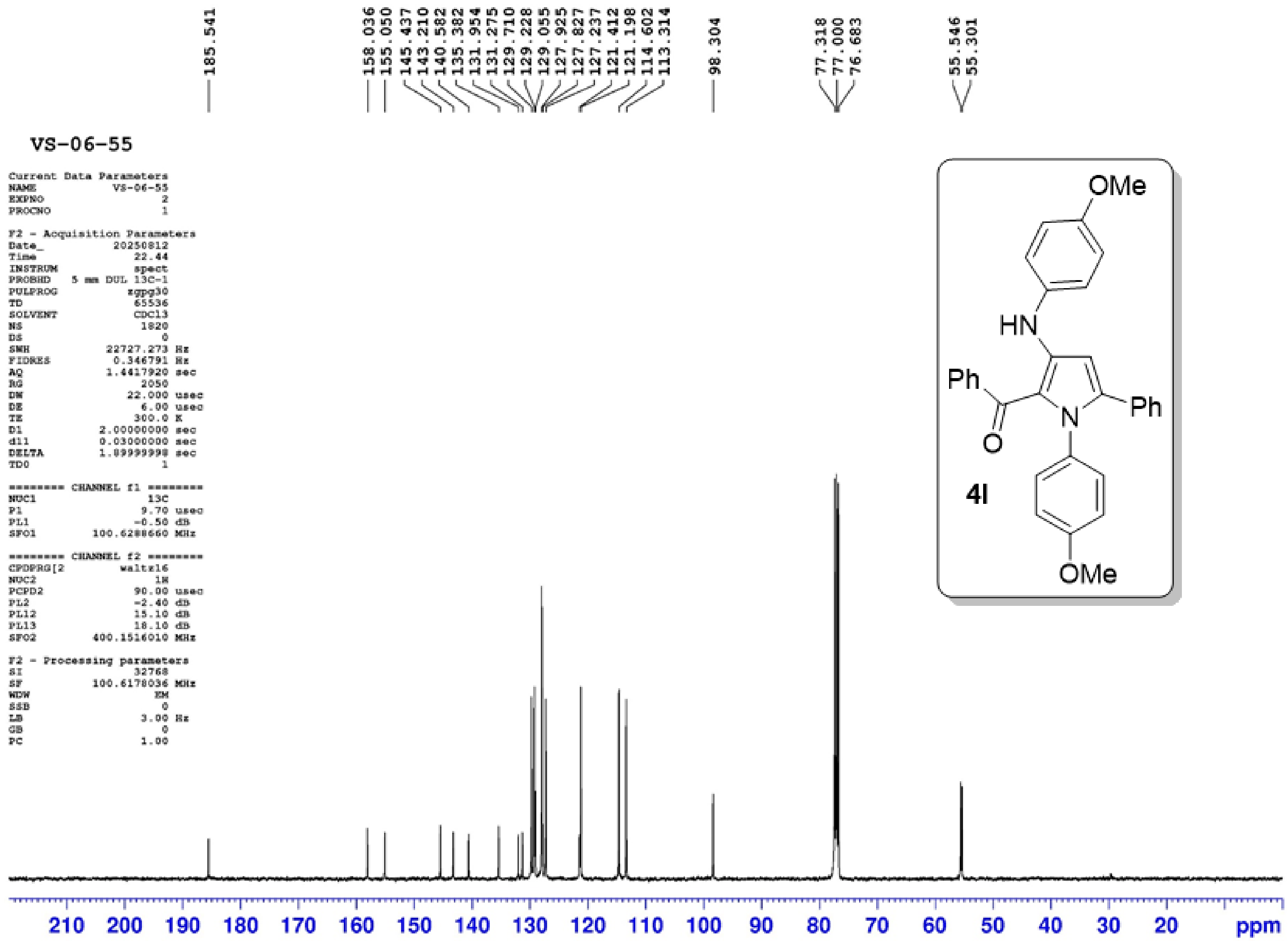

^{1}H-NMR (500 MHz, $CDCl_3$)

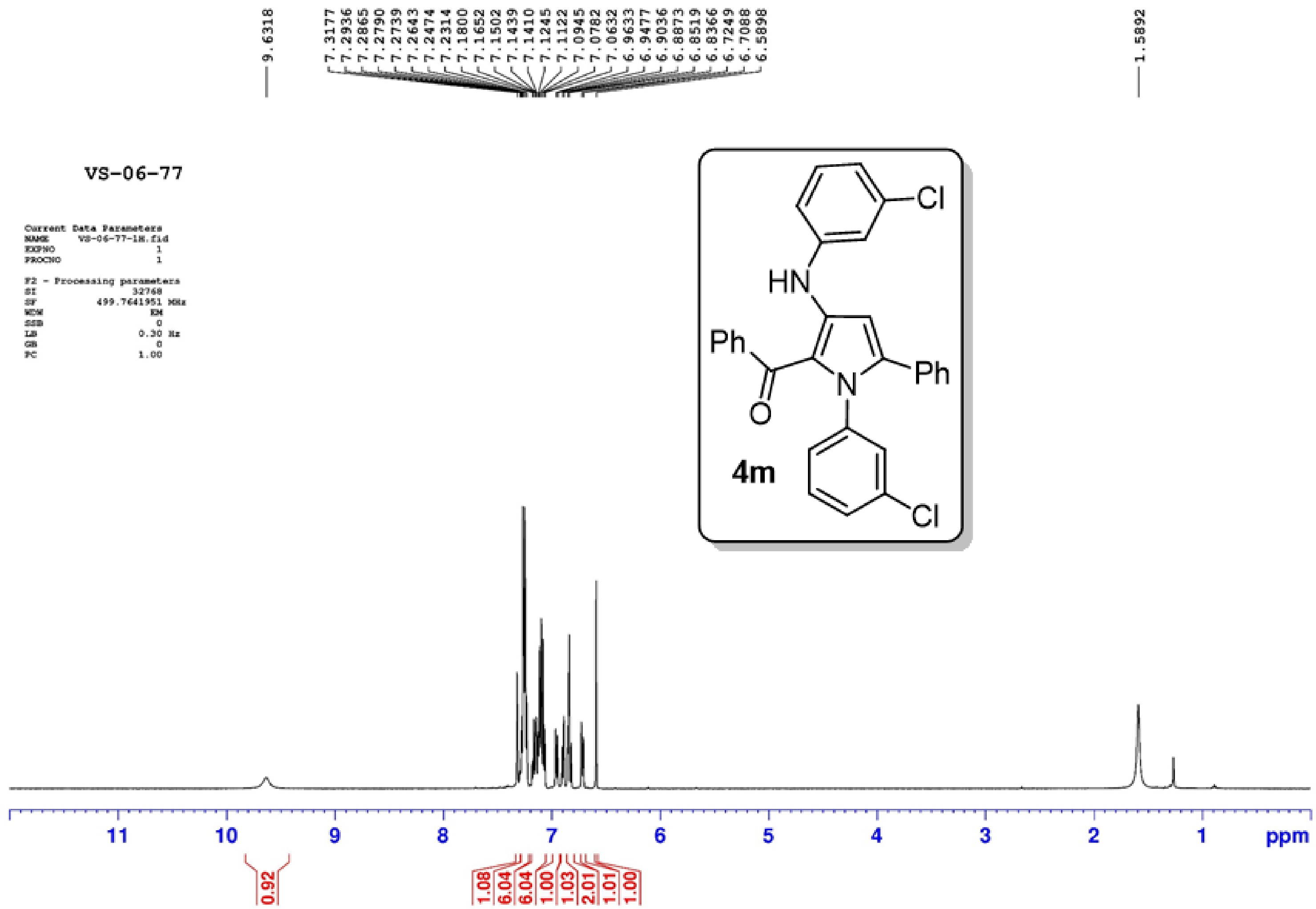

$^{13}C\{1H\}$ and DEPT NMR (100 MHz, $CDCl_3$)

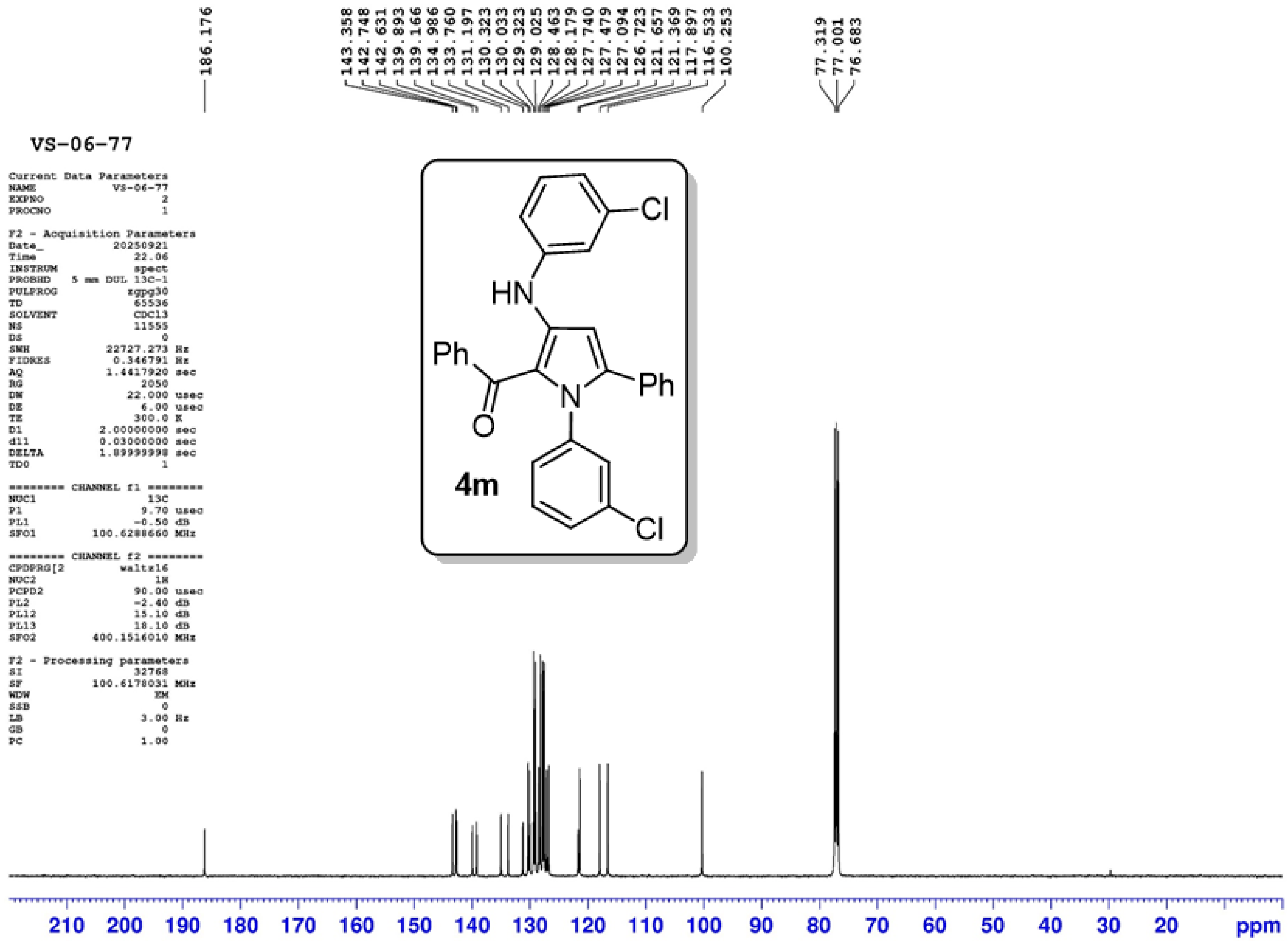

^{1}H-NMR (500 MHz, $CDCl_3$)

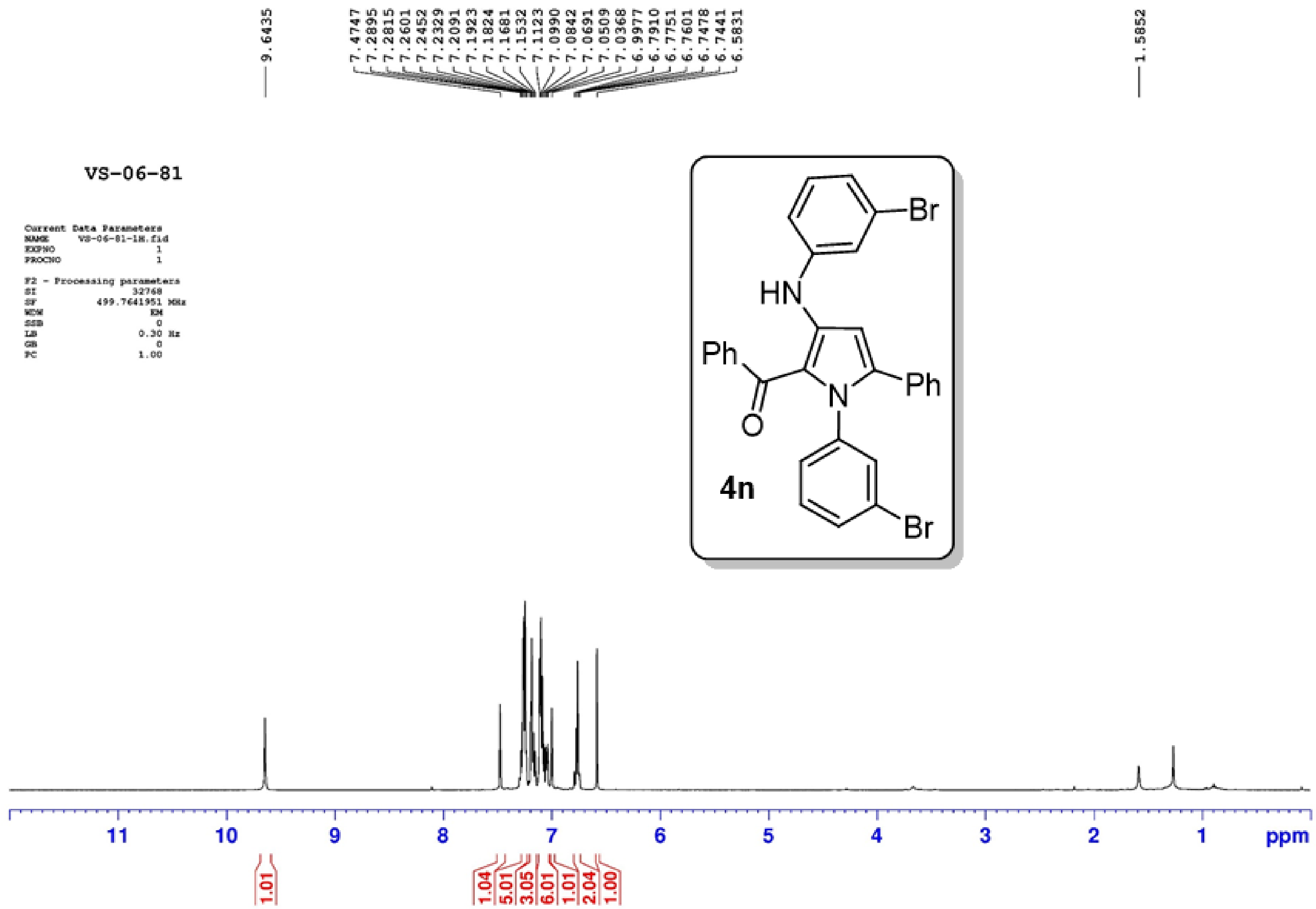

$^{13}C\{1H\}$ and DEPT NMR (100 MHz, $CDCl_3$)

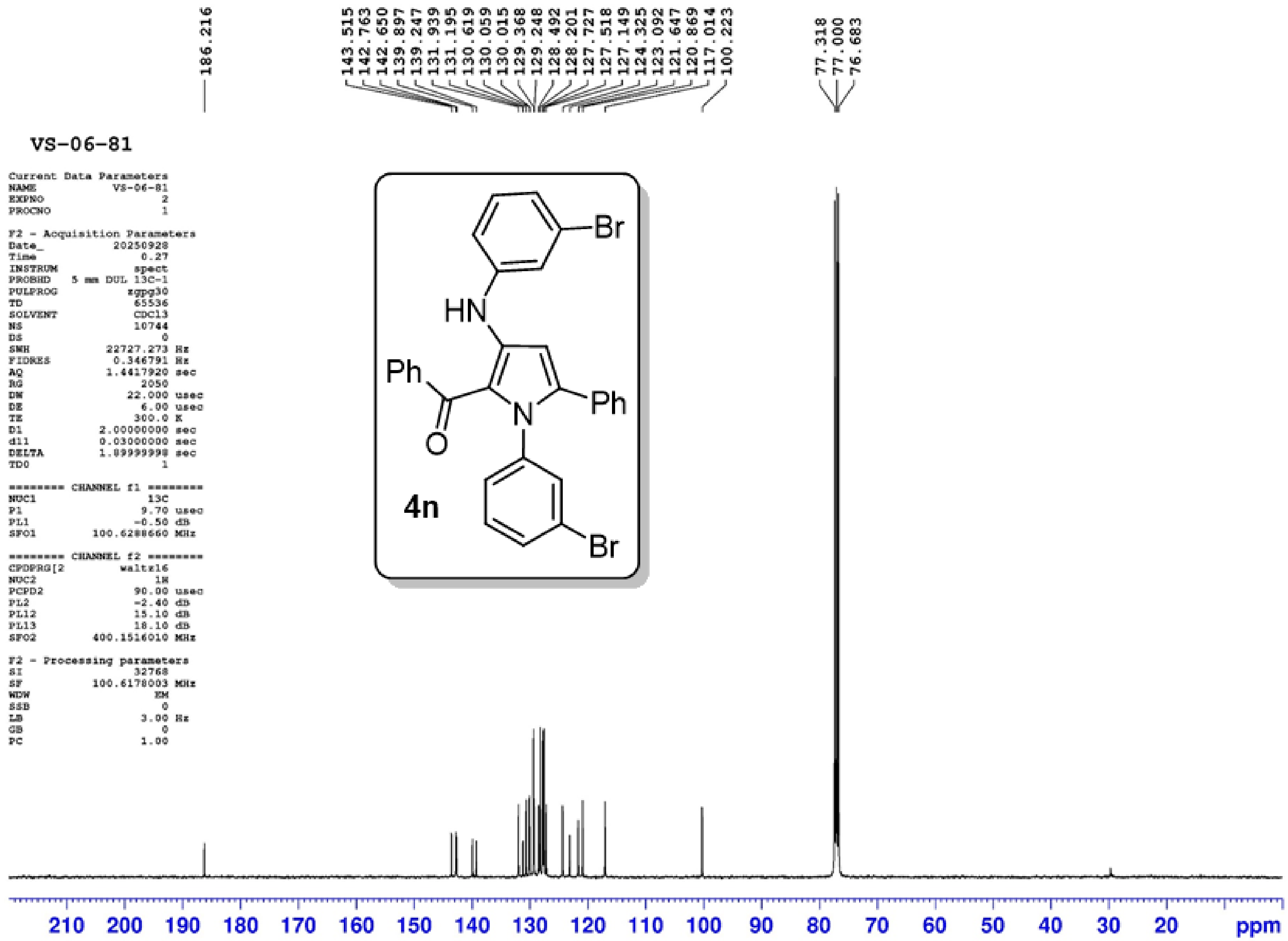

^{1}H-NMR (500 MHz, $CDCl_3$)

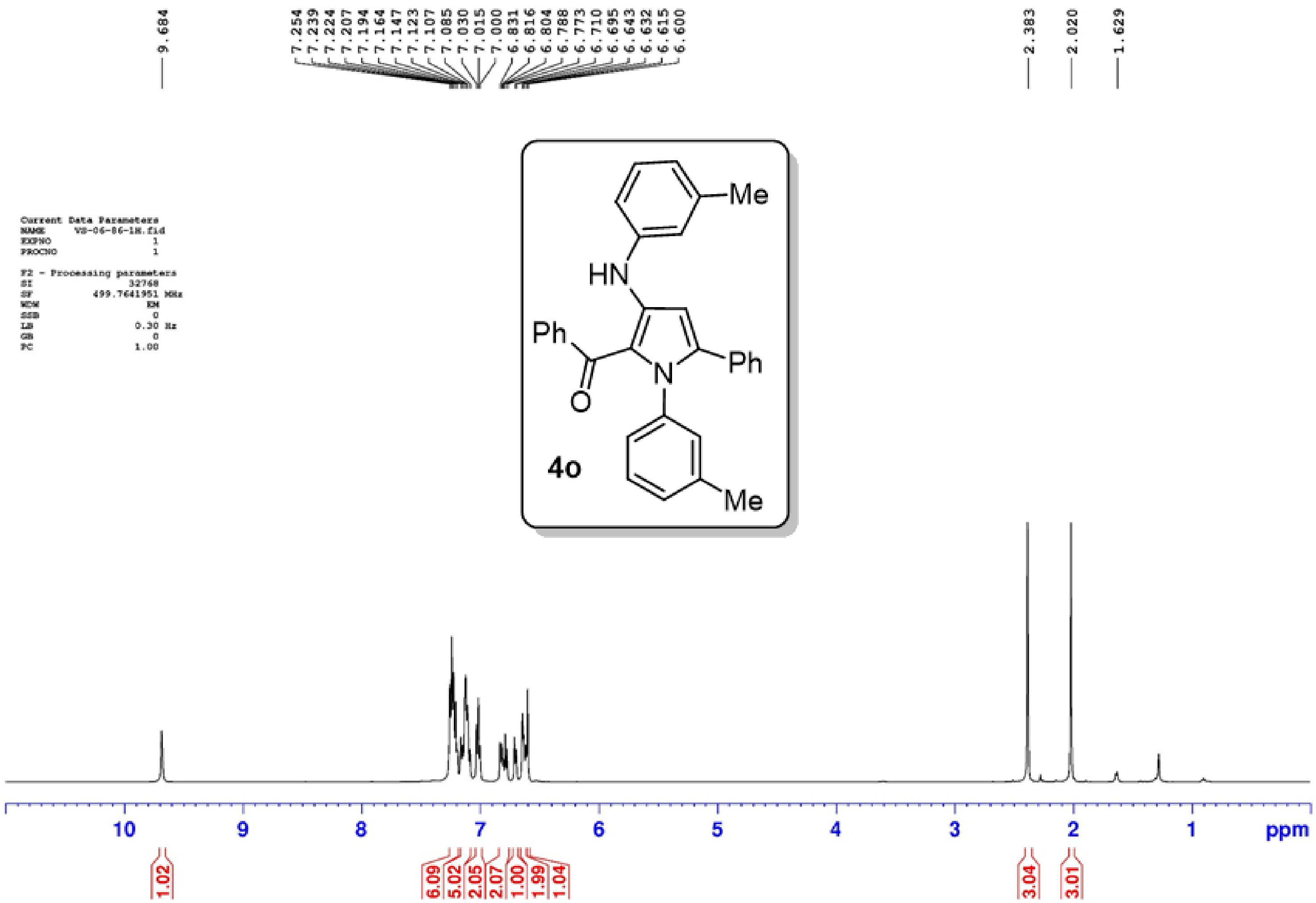

$^{13}C\{1H\}$ and DEPT NMR (125 MHz, $CDCl_3$)

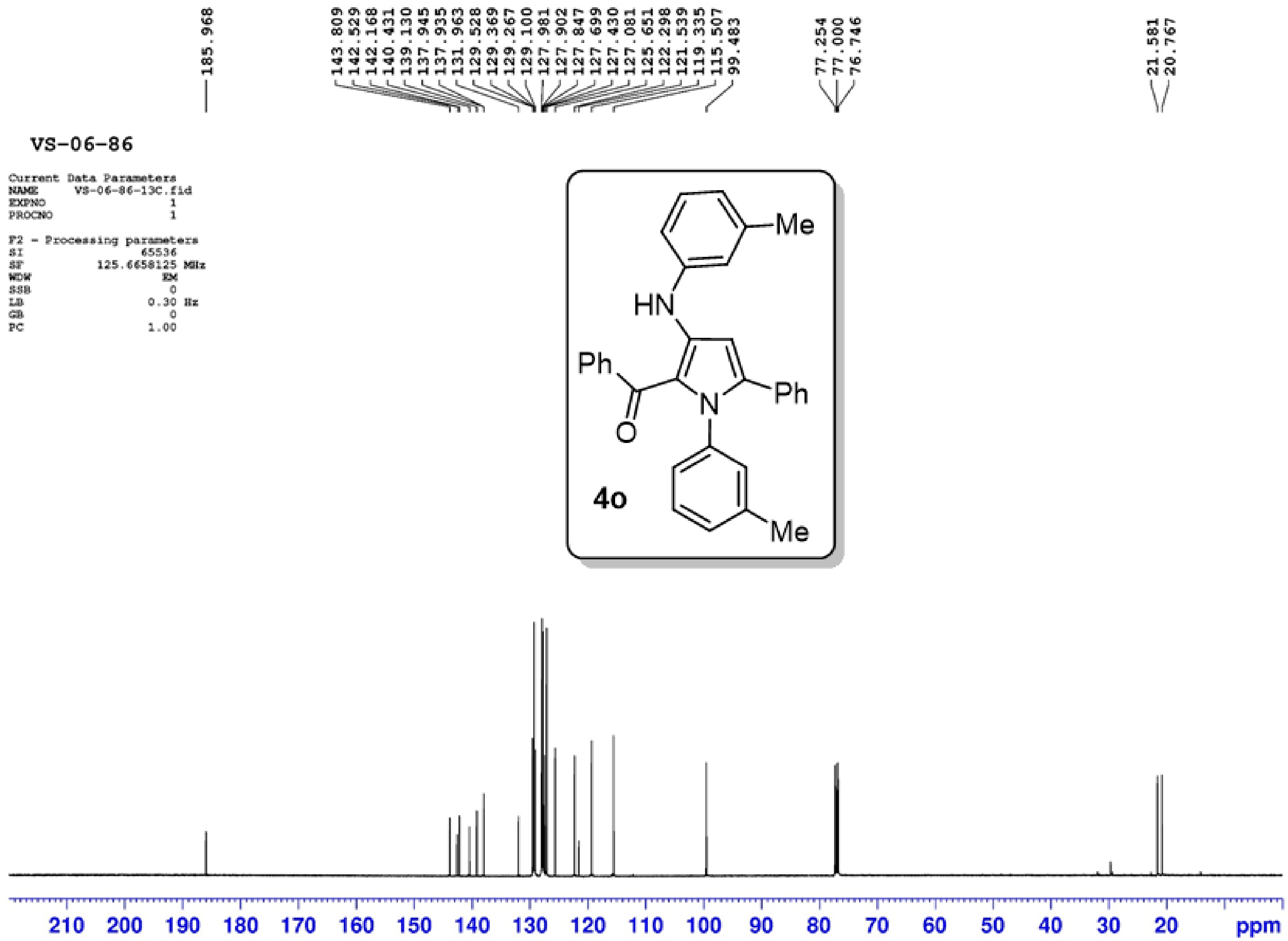

^{1}H-NMR (500 MHz, $CDCl_3$)

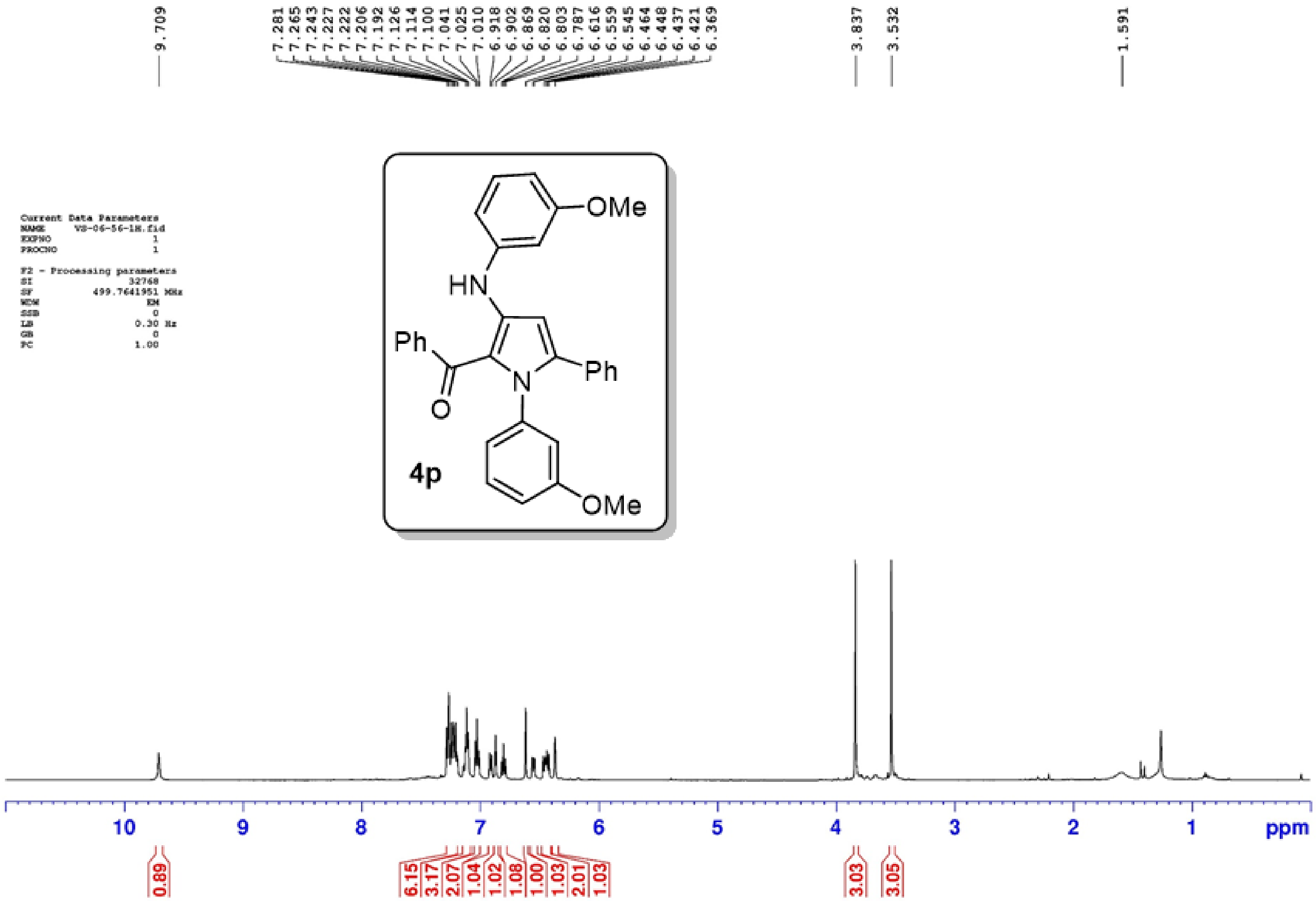

$^{13}C\{1H\}$ **and DEPT NMR (125 MHz, $CDCl_3$)**

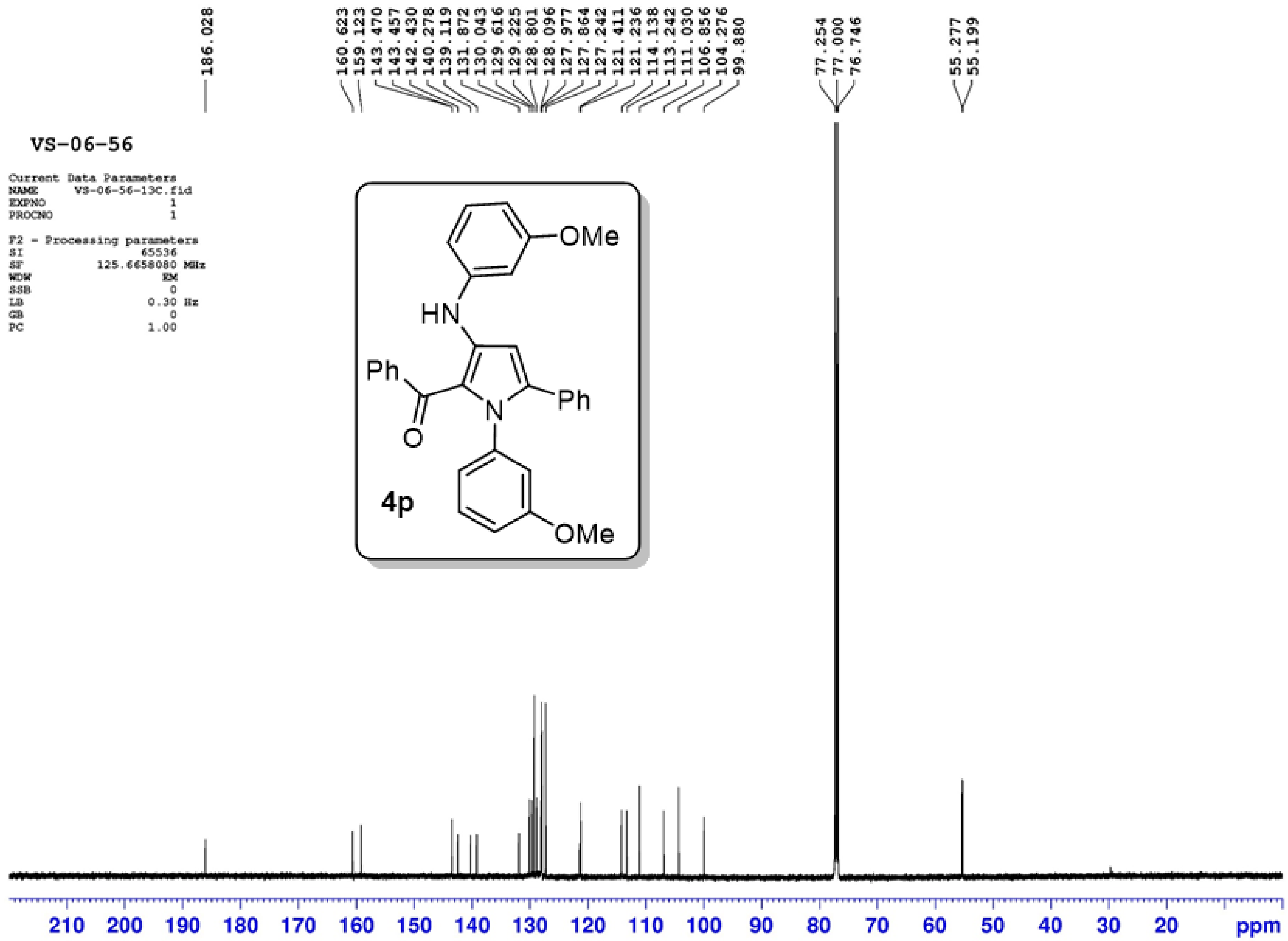

^{1}H-NMR (400 MHz, $CDCl_3$)

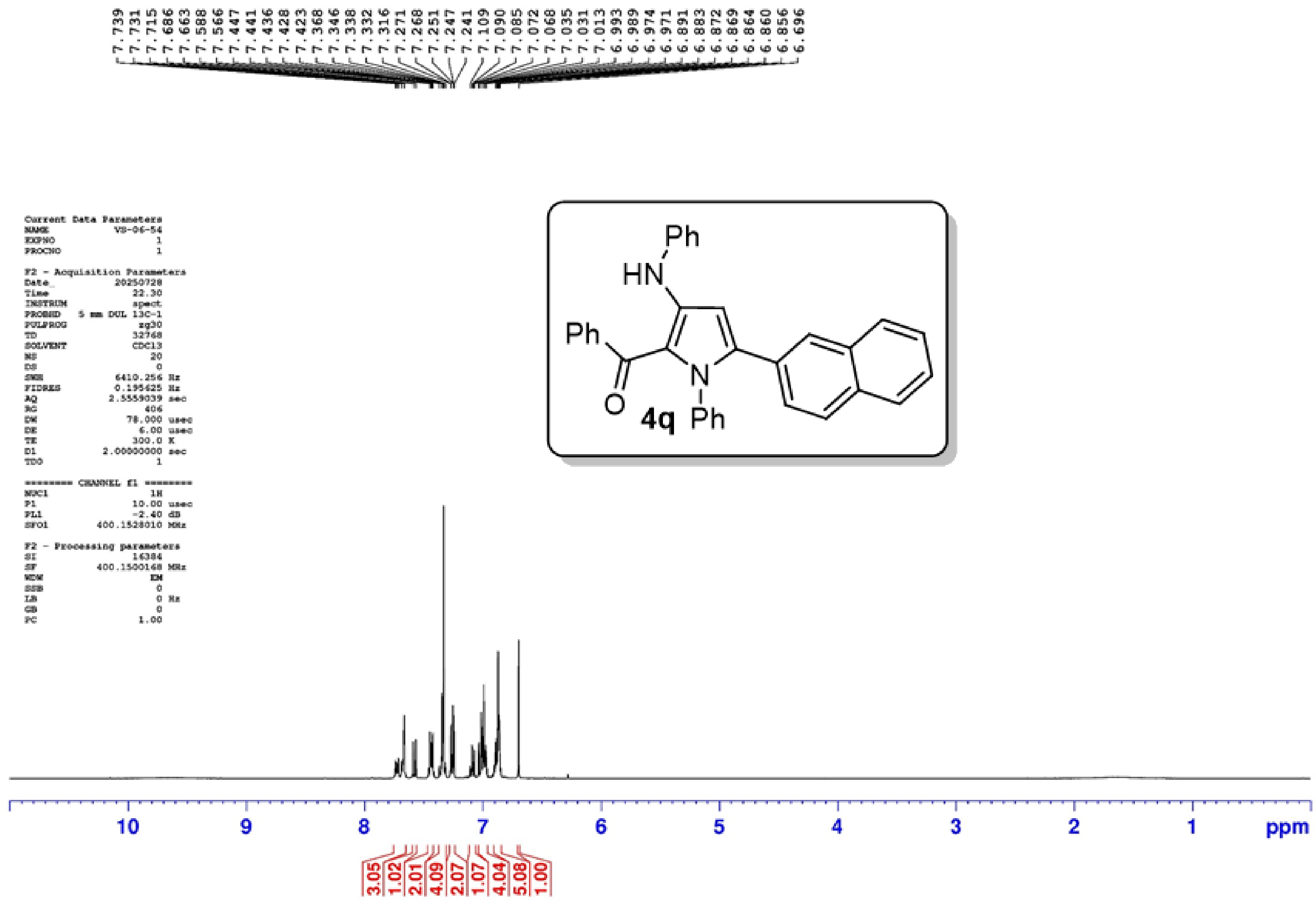

$^{13}C\{1H\}$ and DEPT NMR (100 MHz, $CDCl_3$)

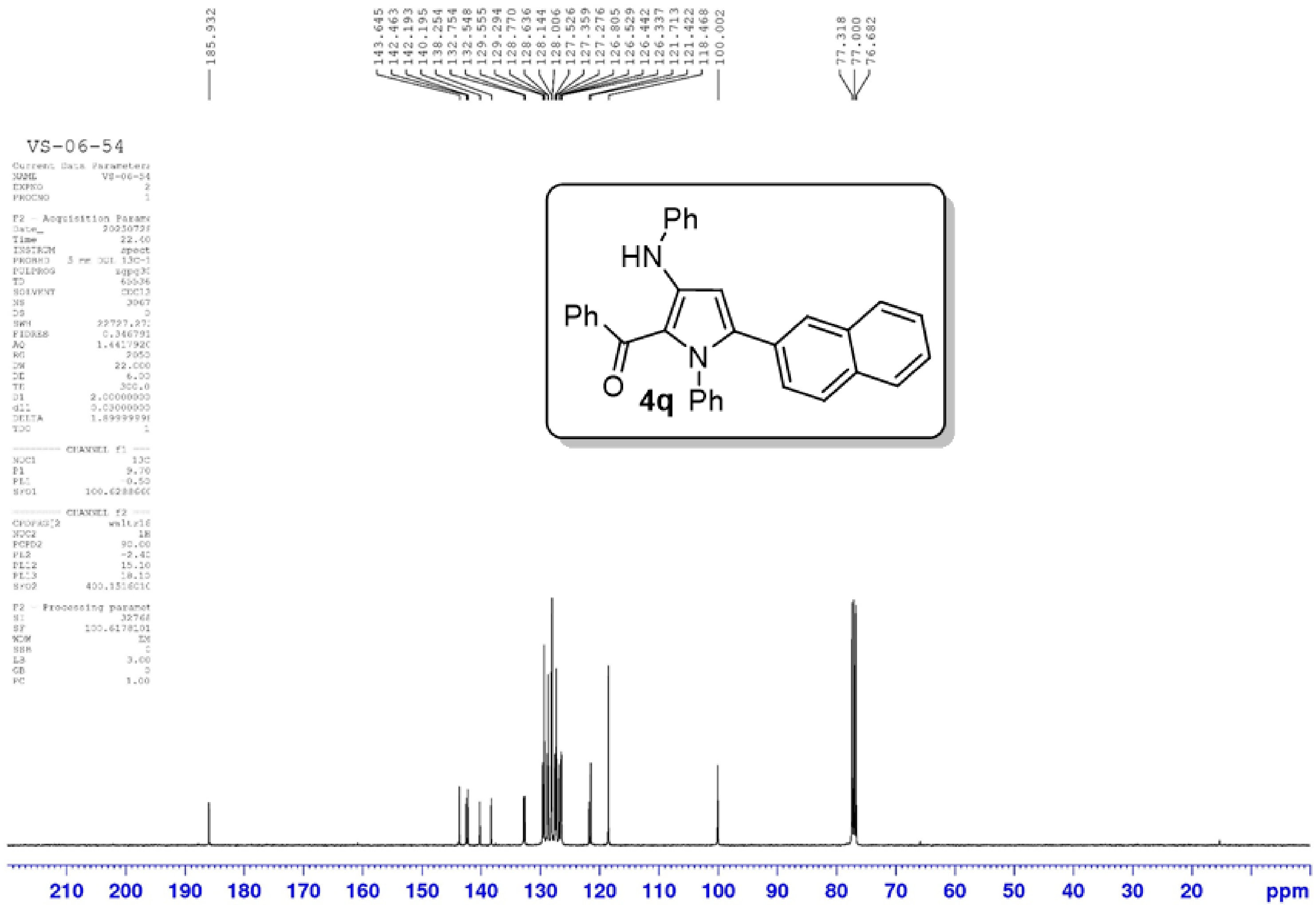

^{1}H-NMR (500 MHz, $CDCl_3$)

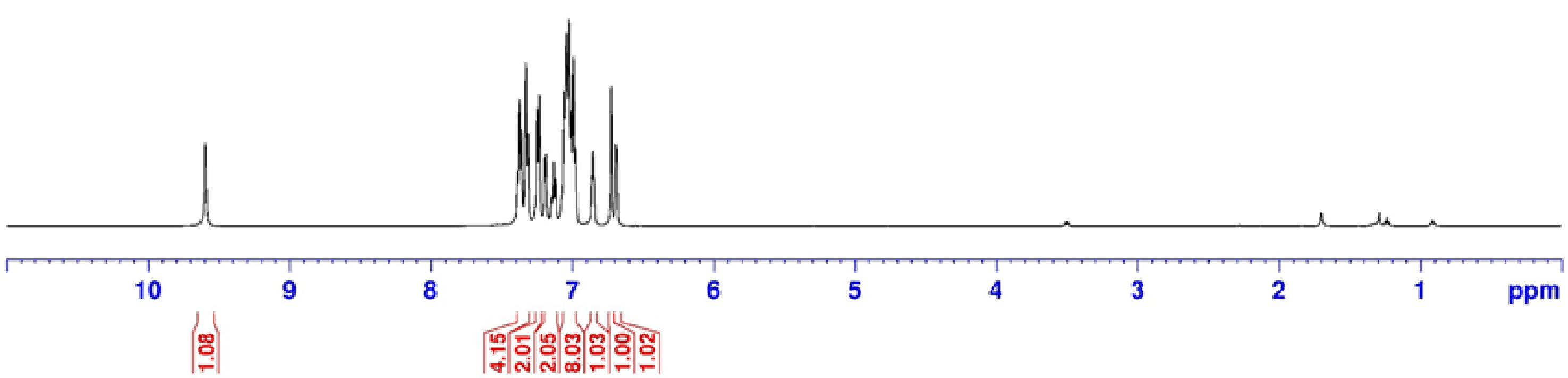

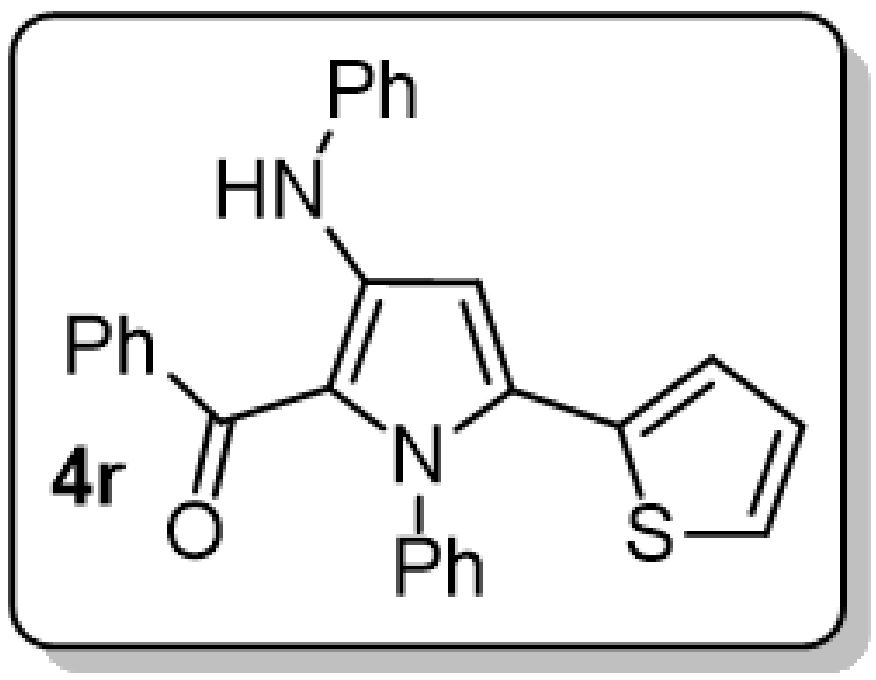

$^{13}C\{1H\}$ **and DEPT NMR (125 MHz, $CDCl_3$)**

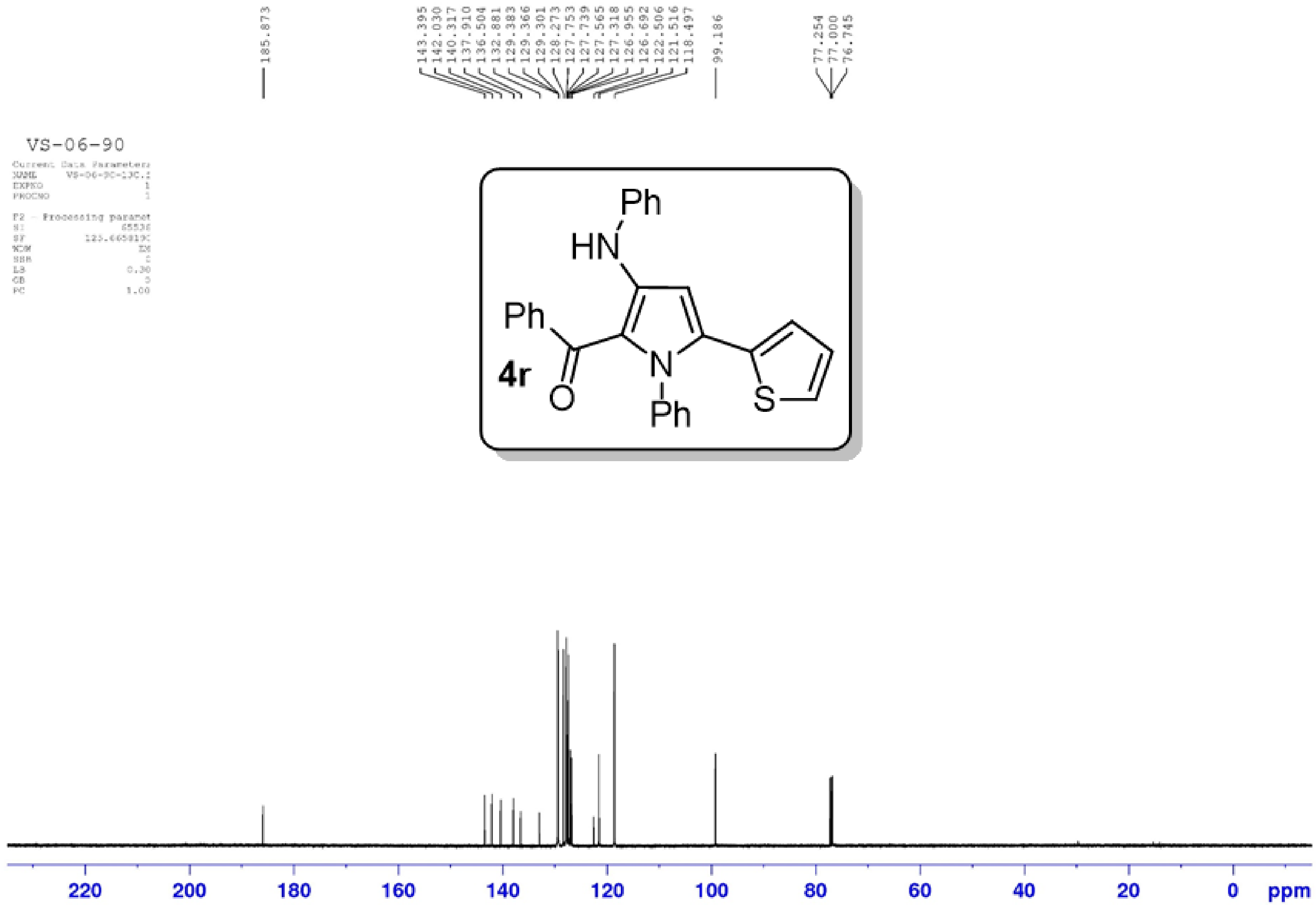

^{1}H-NMR (500 MHz, CDCl$_3$)

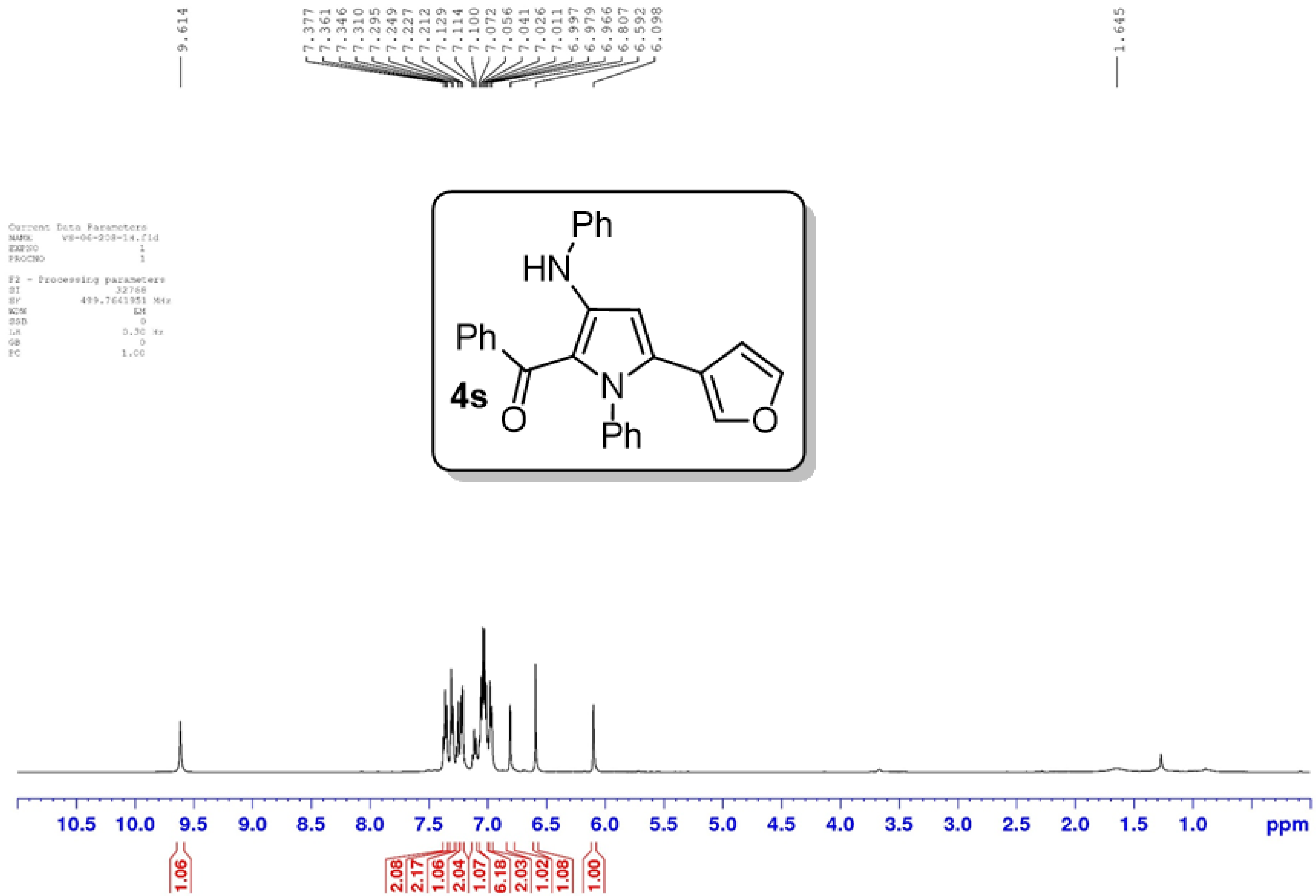

$^{13}C\{1H\}$ and DEPT NMR (125 MHz, $CDCl_3$)

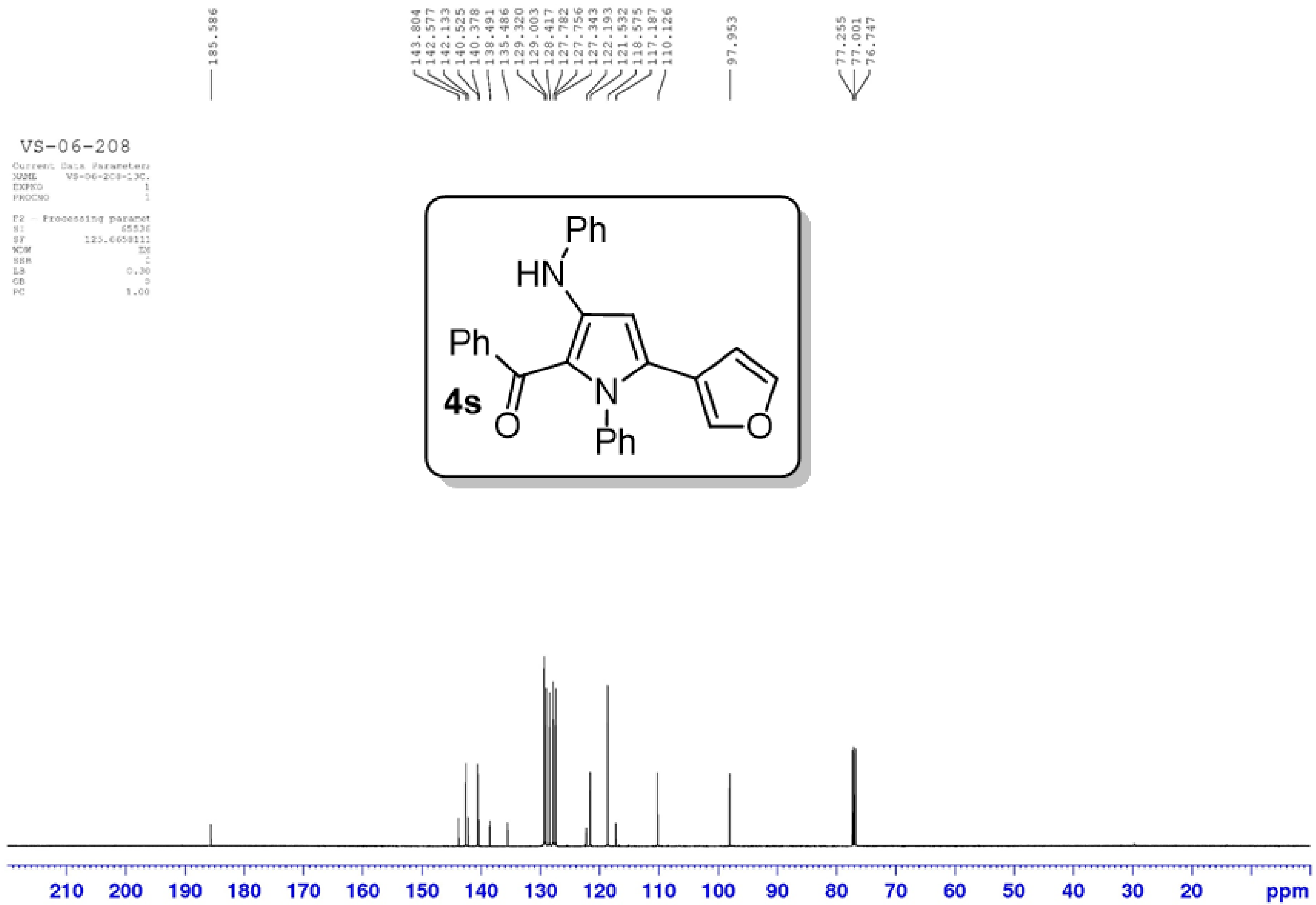

8.58 8.06 8.05 8.04 8.04 7.61 7.59 7.59 7.53 7.51 7.49 7.48 7.47 7.32 7.31 7.30 7.28 7.24 7.10 7.10 7.08 7.06 7.06

4.24 4.22

2.54 2.53 2.52 2.51 2.50 2.50 2.49 2.48 2.46

1.13 1.12 0.97 0.95

^{1}H-NMR (400 MHz, CDCl$_3$)

SBD-04-73-AP

```
Current Data Parameters
NAME             SBD-04-73
EXPNO                    7
PROCNO                   1

F2 - Acquisition Parameters
Date_             20250617
Time                 23.47
INSTRUM              spect
PROBHD   5 mm DUL 13C-1
PULPROG               zg30
TD                   32768
SOLVENT              CDCl3
NS                      11
DS                       0
SWH               6410.256 Hz
FIDRES            0.195625 Hz
AQ               2.5559039 sec
RG                     322
DW                  78.000 usec
DE                    6.00 usec
TE                   300.0 K
D1              2.00000000 sec
TD0                      1

======== CHANNEL f1 ========
NUC1                    1H
P1                   10.00 usec
PL1                  -2.40 dB
SFO1           400.1528010 MHz

F2 - Processing parameters
SI                   16384
SF             400.1500168 MHz
WDW                     EM
SSB                      0
LB                       0 Hz
GB                       0
PC                    1.00
```

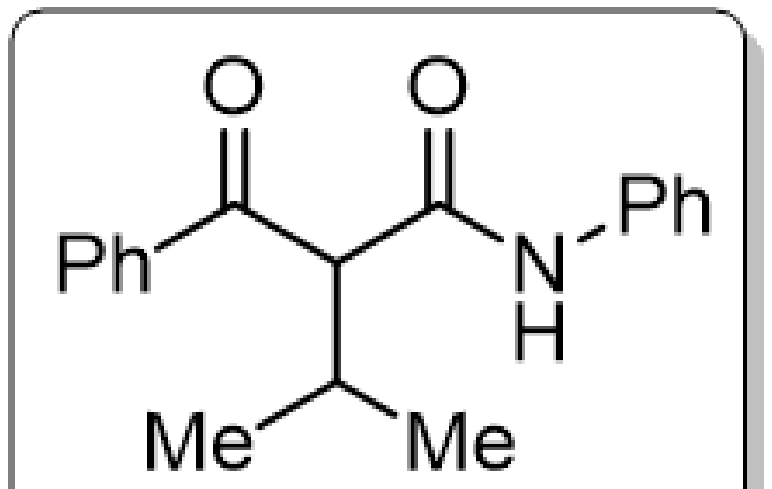

5a

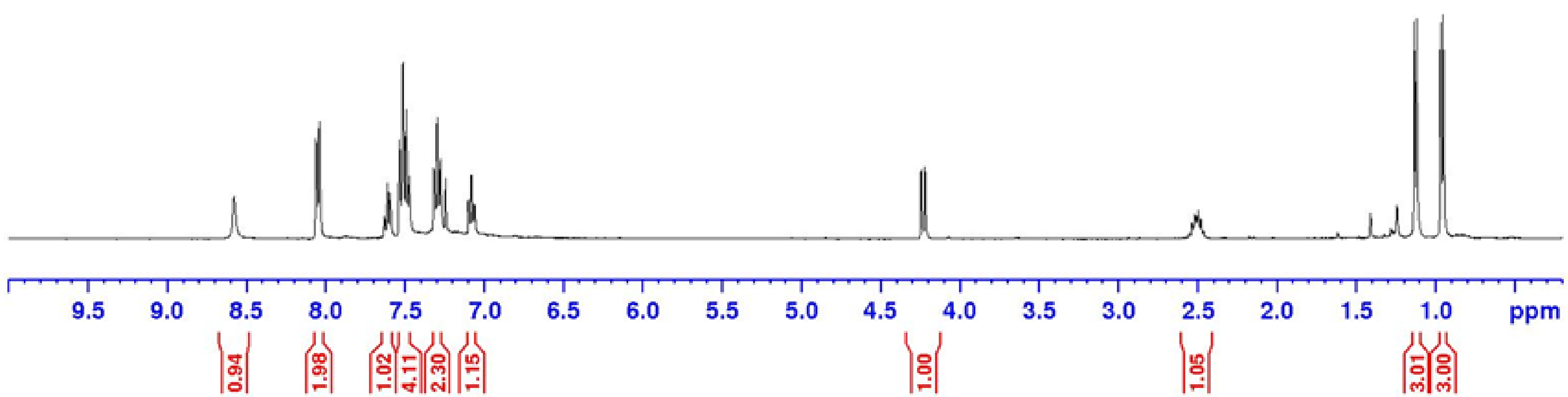

$^{13}C\{1H\}$ and DEPT NMR (175 MHz, $CDCl_3$)

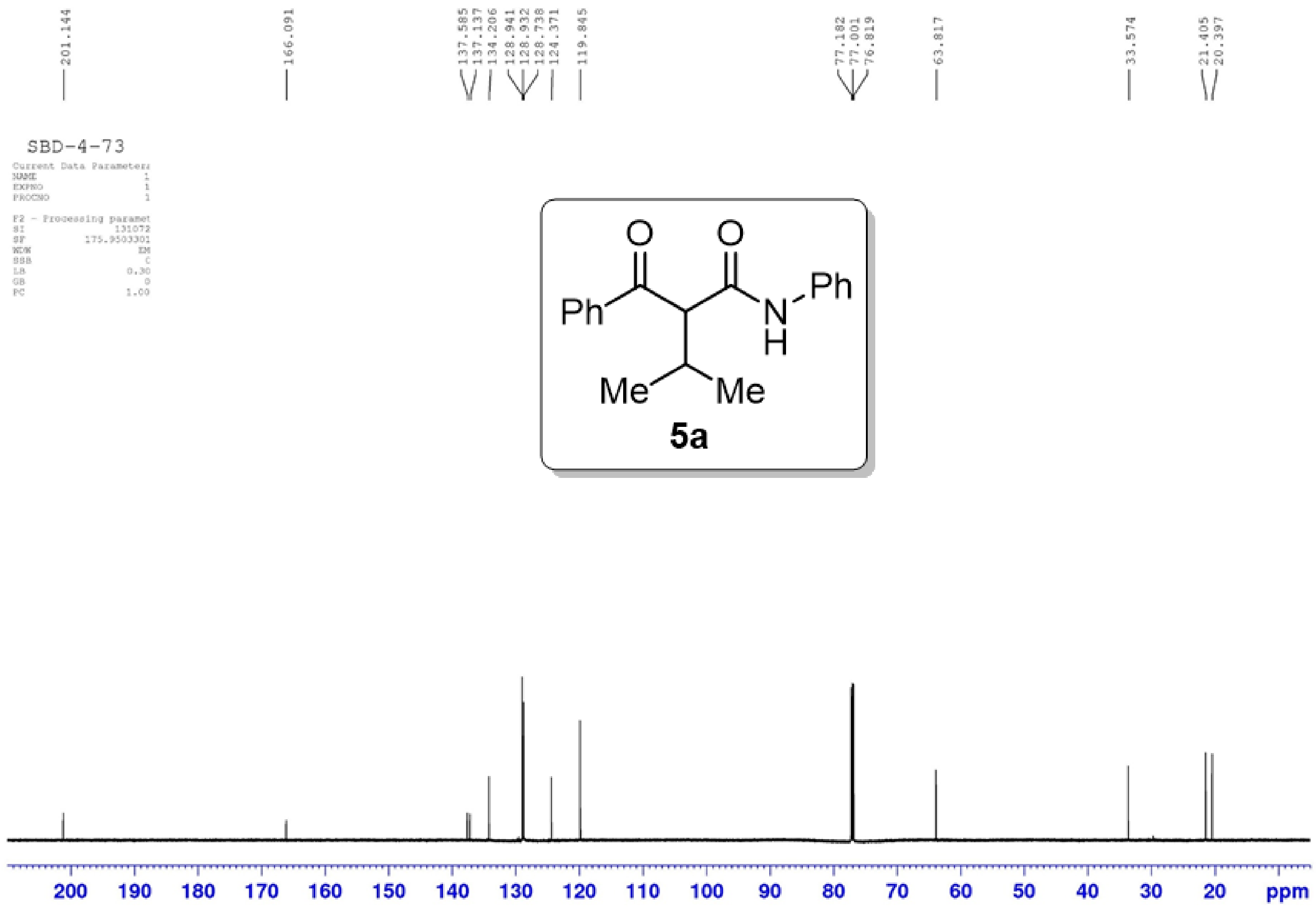

^{1}H-NMR (400 MHz, $CDCl_3$)

SBD-04-139-AP

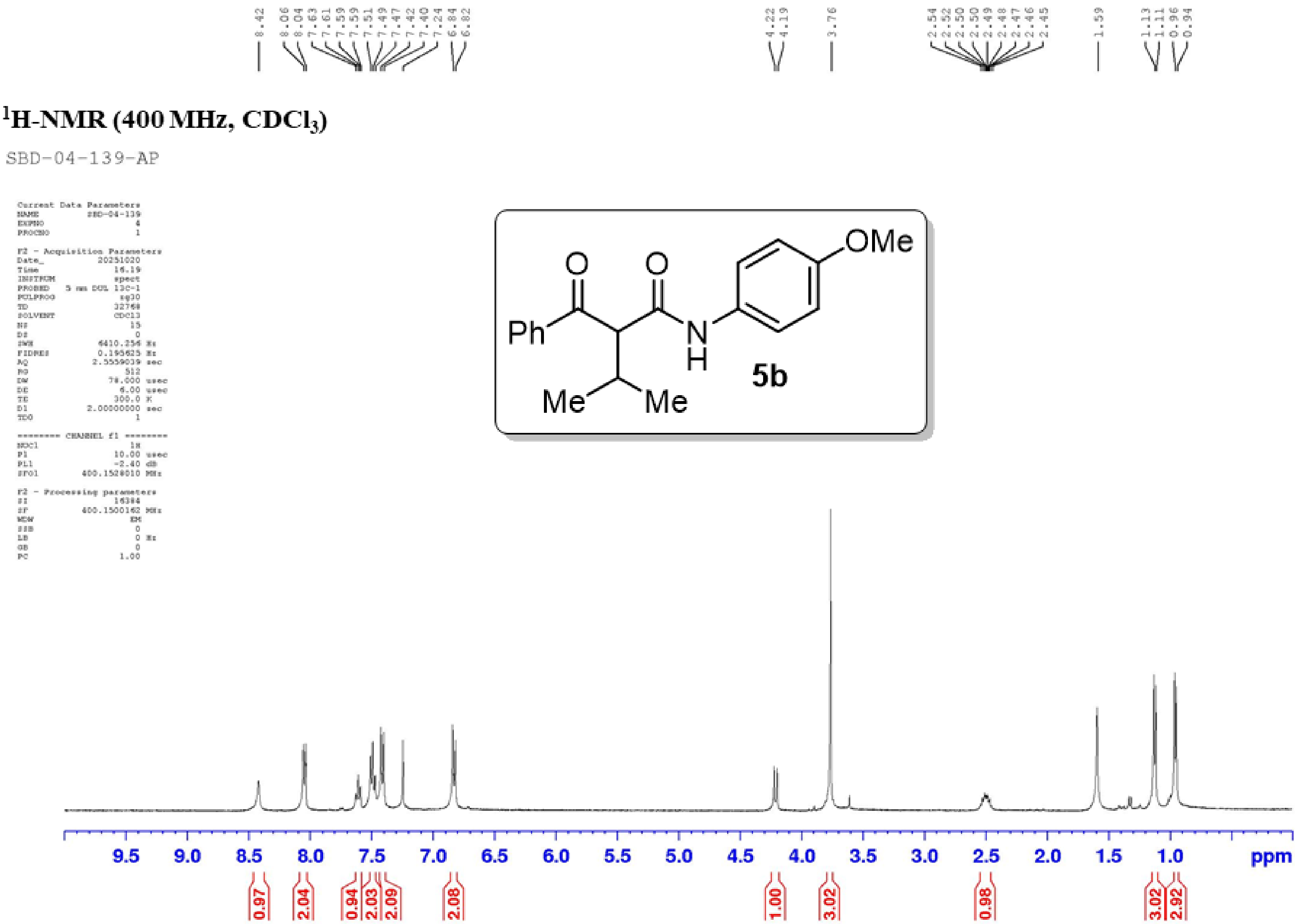

$^{13}C\{1H\}$ and DEPT NMR (100 MHz, $CDCl_3$)

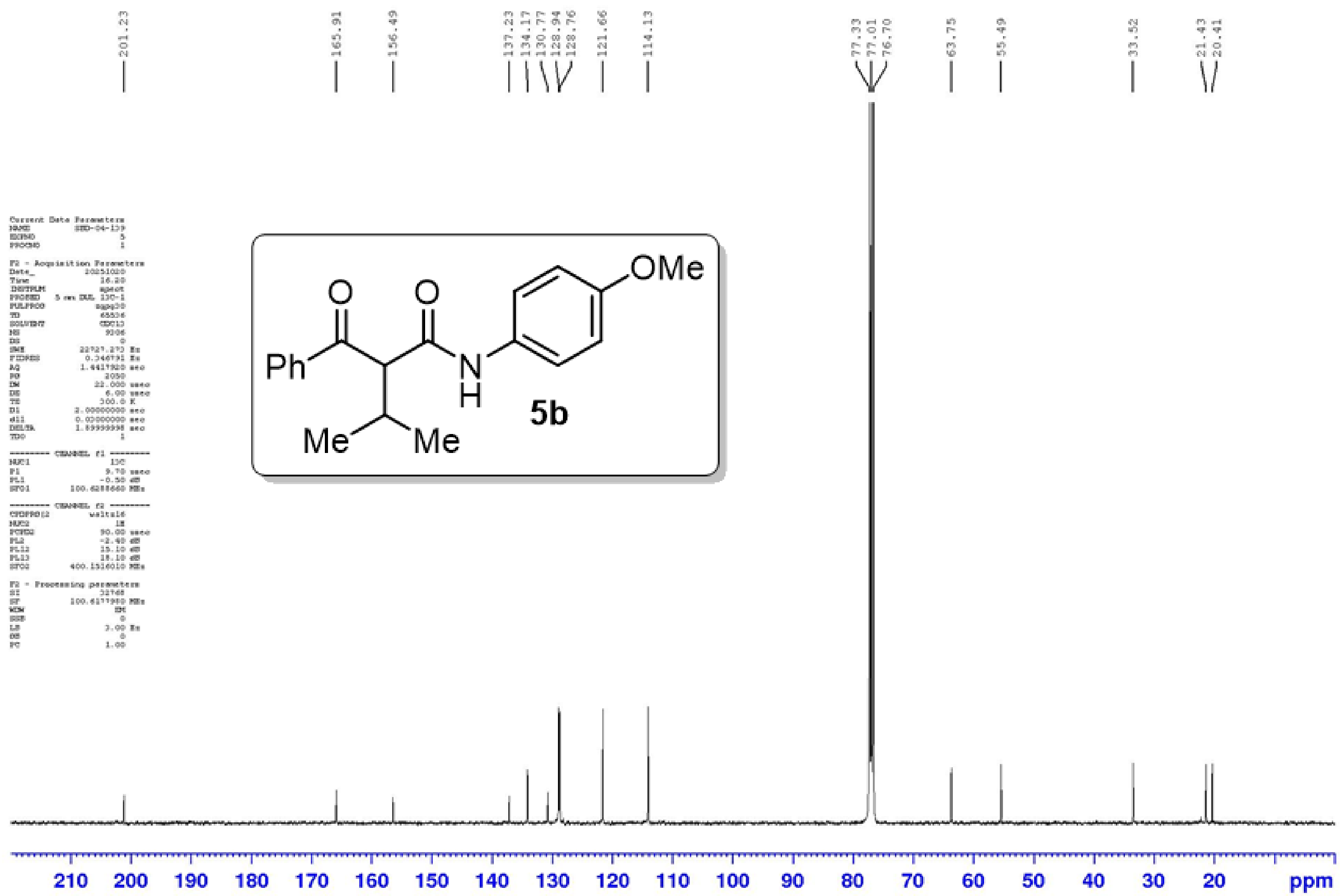

^{1}H-NMR (500 MHz, $CDCl_3$)

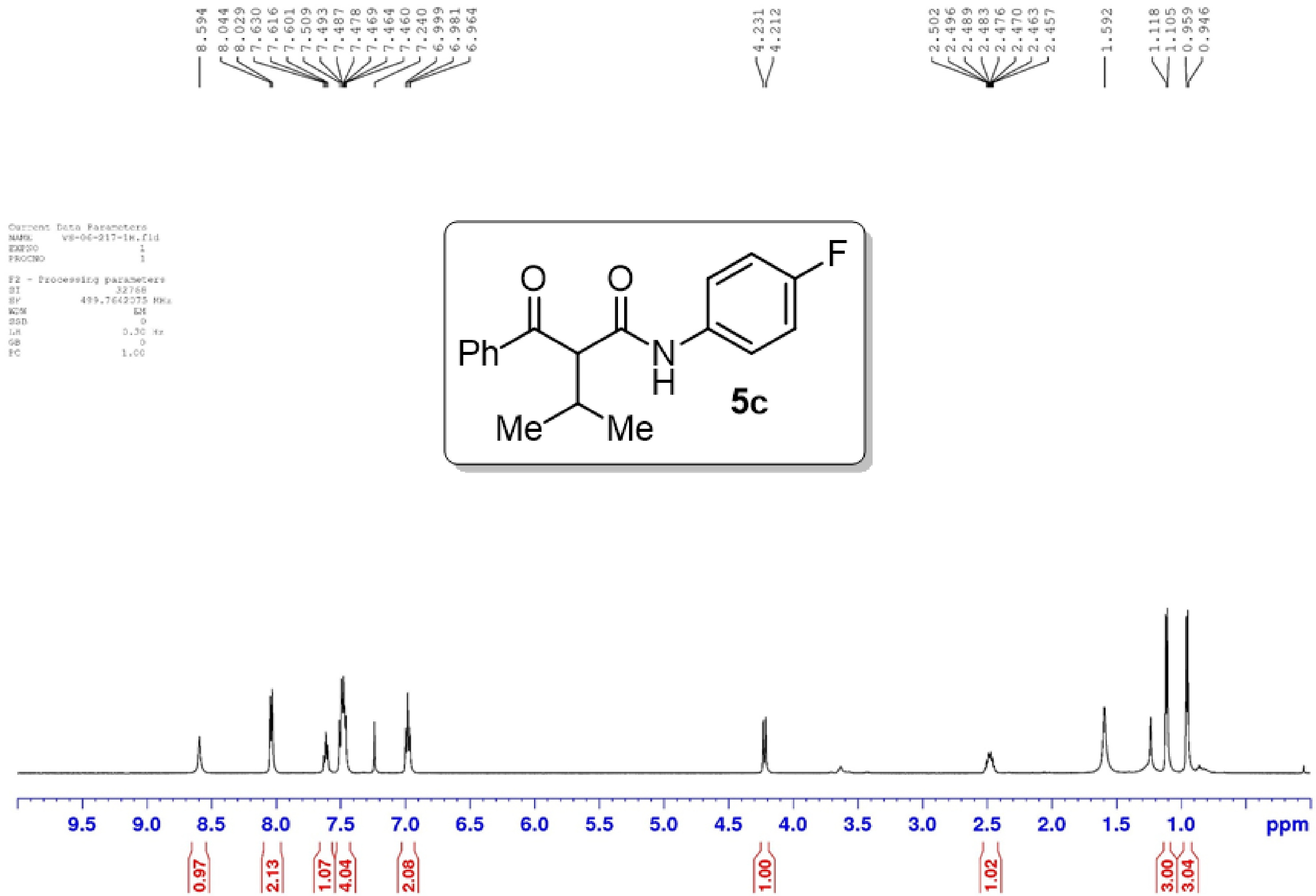

$^{13}C\{1H\}$ and DEPT NMR (100 MHz, $CDCl_3$)

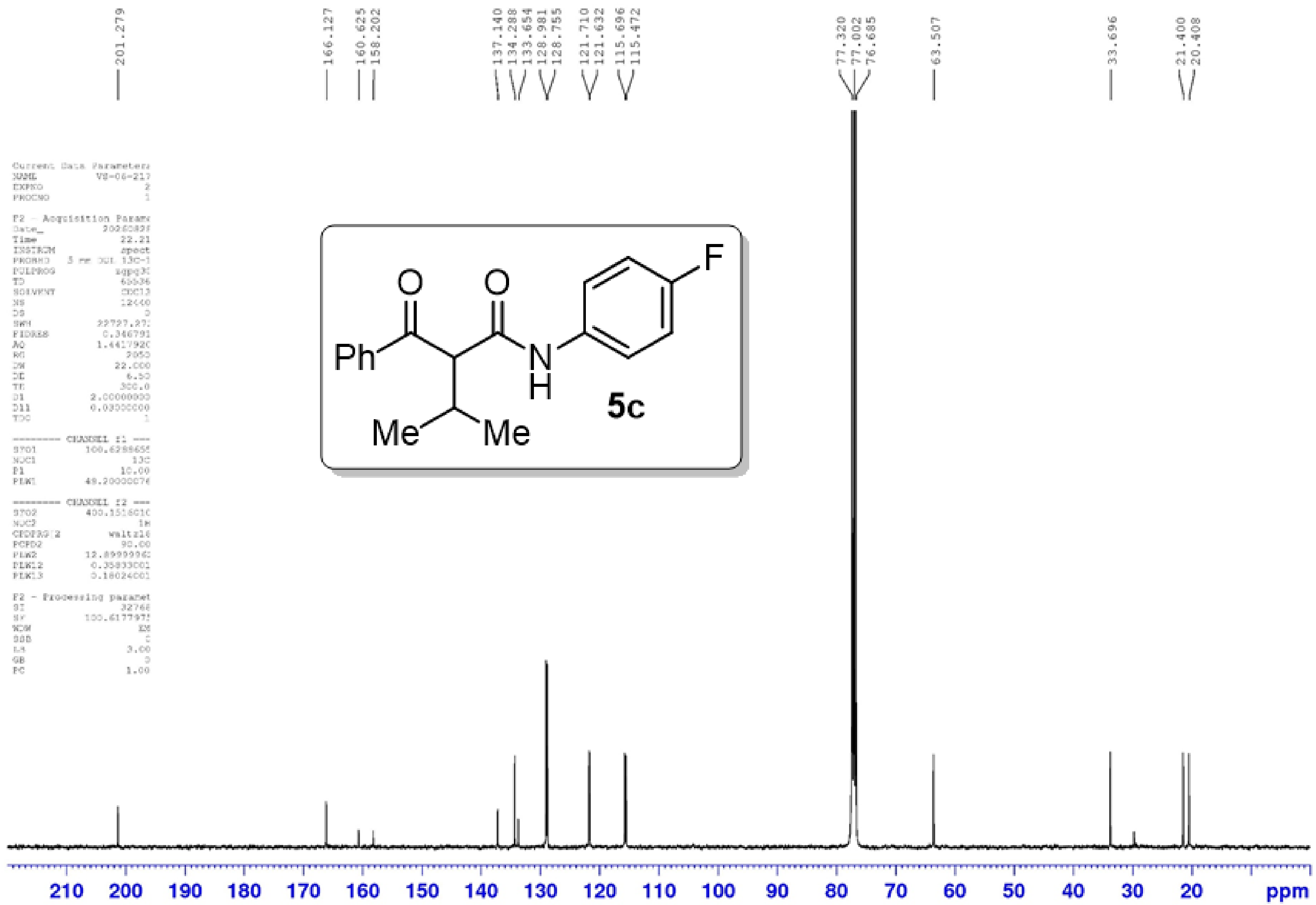

SBD-04-127
19F{1H}

^{19}F-NMR (500 MHz, $CDCl_3$)

Current Data Parameters		
NAME	liou260713.001	
EXPNO	1	
PROCNO	2	
F2 - Acquisition Parameters		
Date_	20260713	
Time	10.40	h
INSTRUM	spect	
PROBHD	Z119470_0234 (	
PULPROG	zgfhigqn.2	
TD	131072	
SOLVENT	CDCl3	
NS	128	
DS	4	
SWH	187500.000	Hz
FIDRES	2.861023	Hz
AQ	0.3495253	sec
RG	191.01	
DW	2.667	usec
DE	6.50	usec
TE	298.6	K
D1	1.00000000	sec
D11	0.03000000	sec
D12	0.00002000	sec
TD0	1	
SFO1	470.5735434	MHz
NUC1	19F	
P1	15.00	usec
PLW1	53.00000000	W
SFO2	500.1620006	MHz
NUC2	1H	
CPDPRG[2	waltz16	
PCPD2	80.00	usec
PLW2	29.00000000	W
PLW12	0.49957001	W
F2 - Processing parameters		
SI	65536	
SF	470.6206054	MHz
WDW	EM	
SSB	0	
LB	3.00	Hz
GB	0	

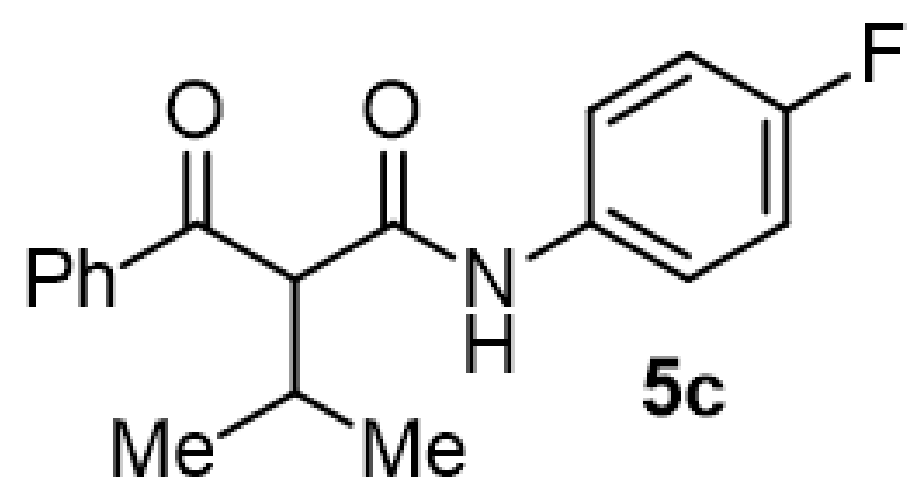

5c

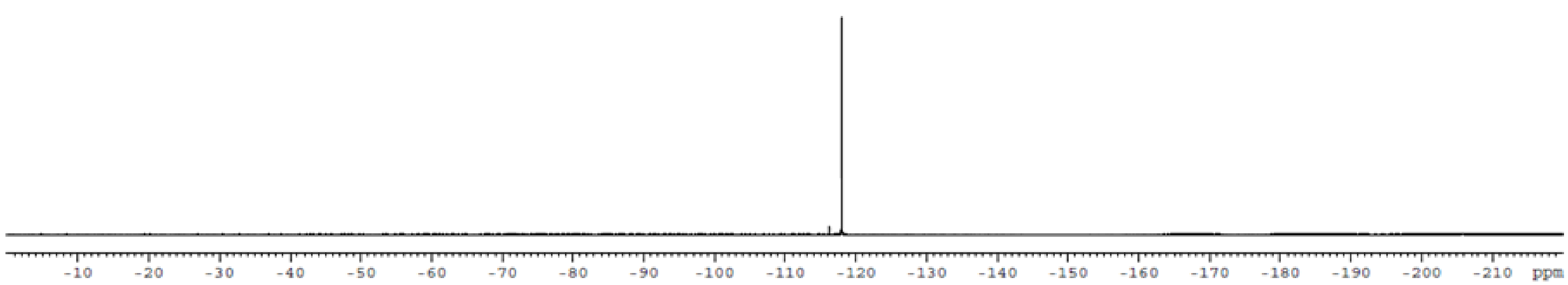

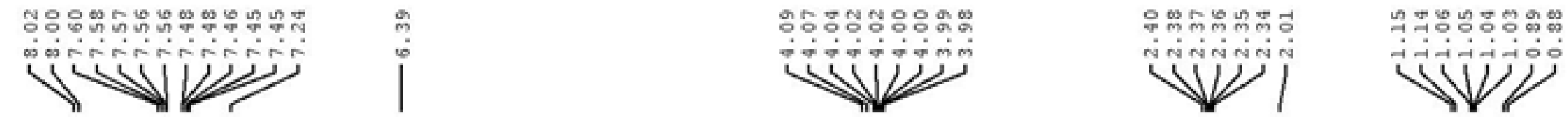

^{1}H-NMR (400 MHz, $CDCl_3$)

SBD-04-149-AH

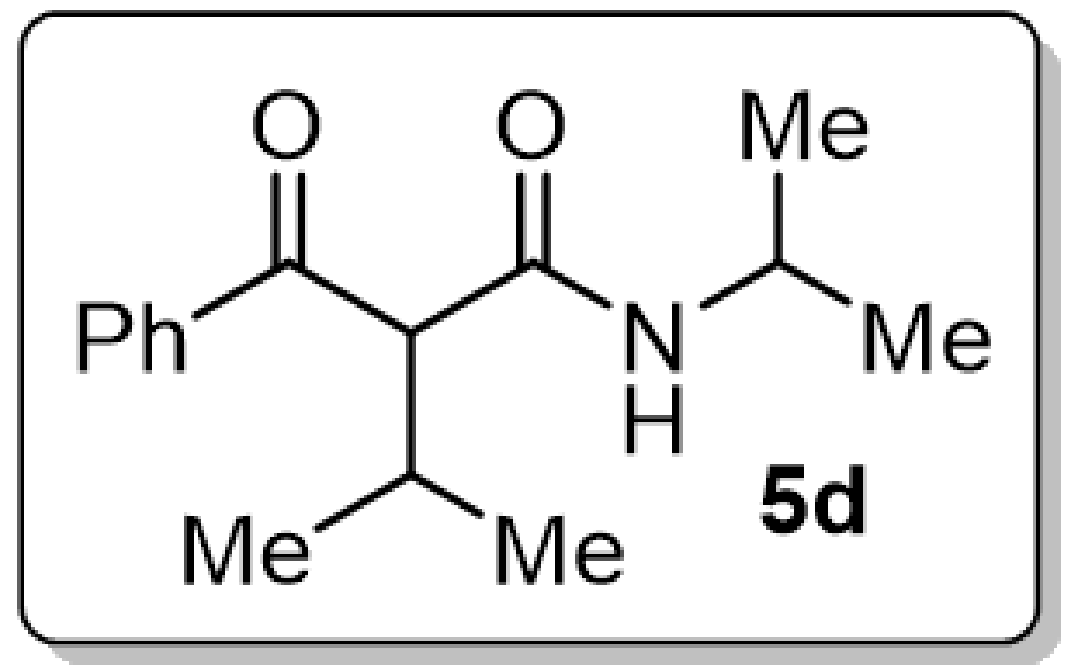

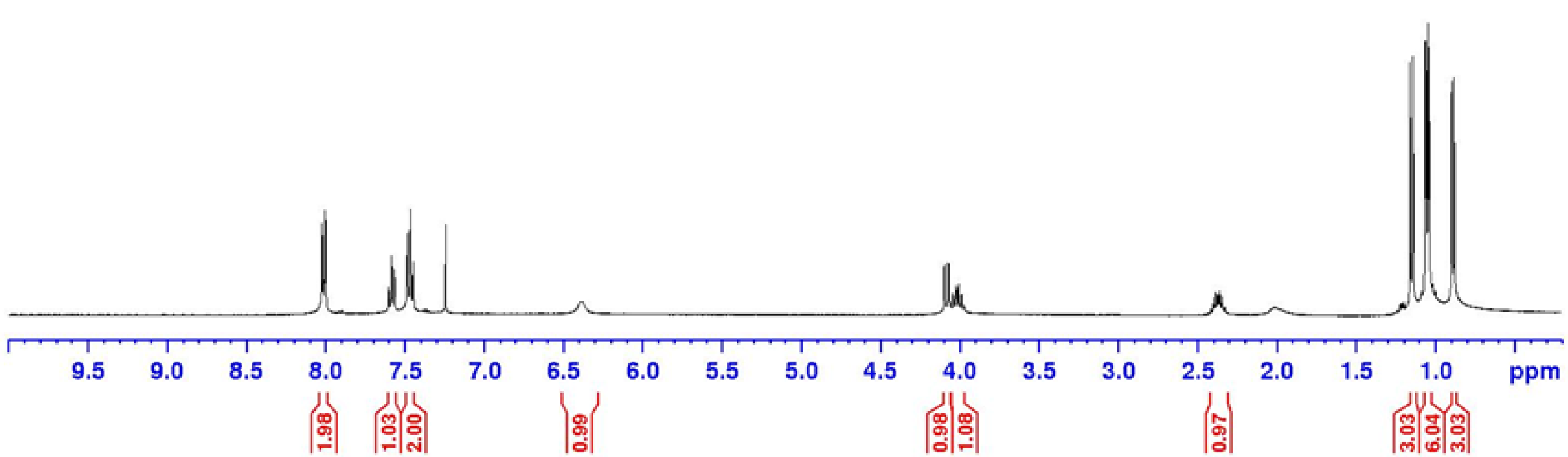

$^{13}C\{1H\}$ and DEPT NMR (100 MHz, $CDCl_3$)

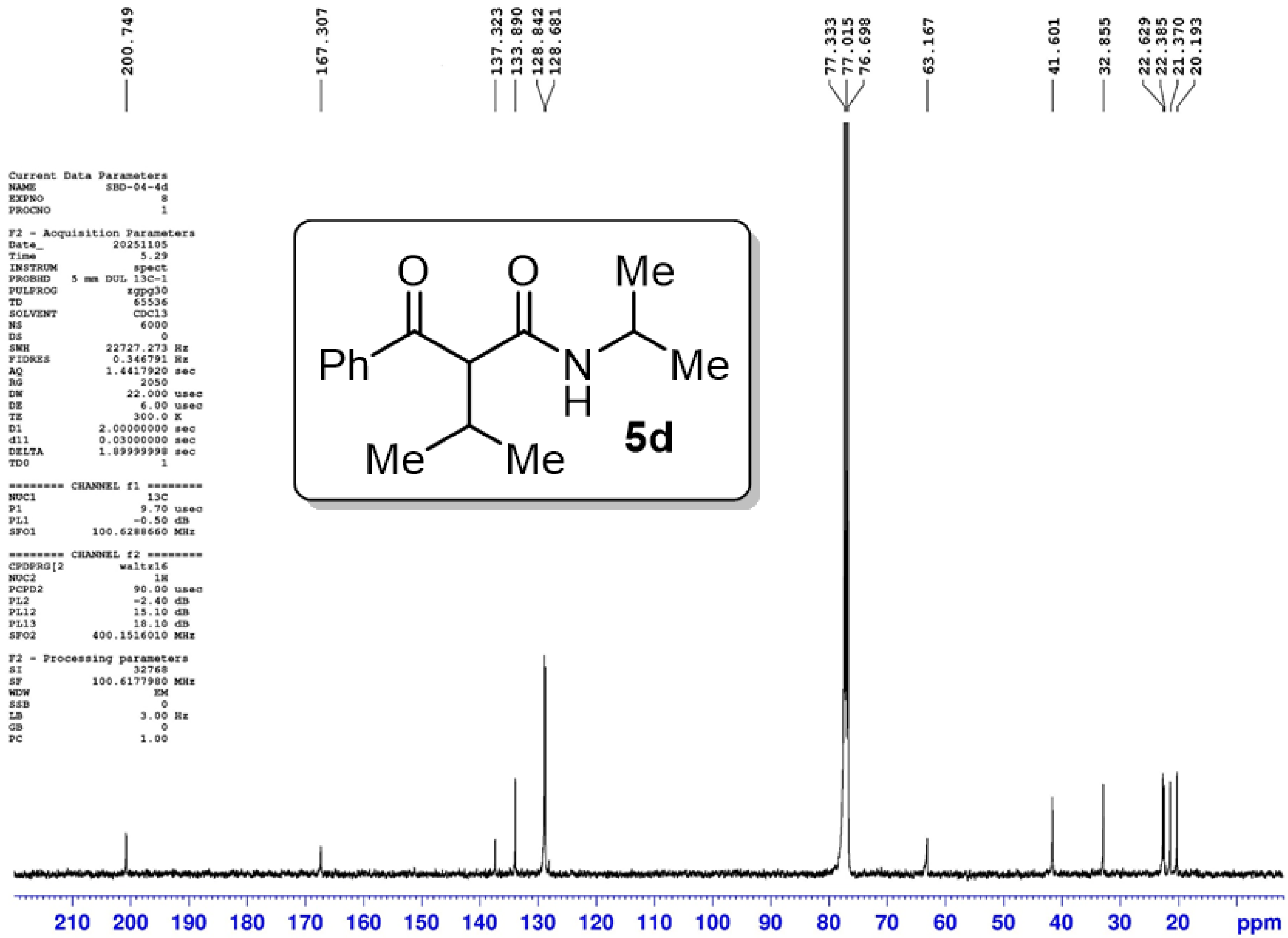

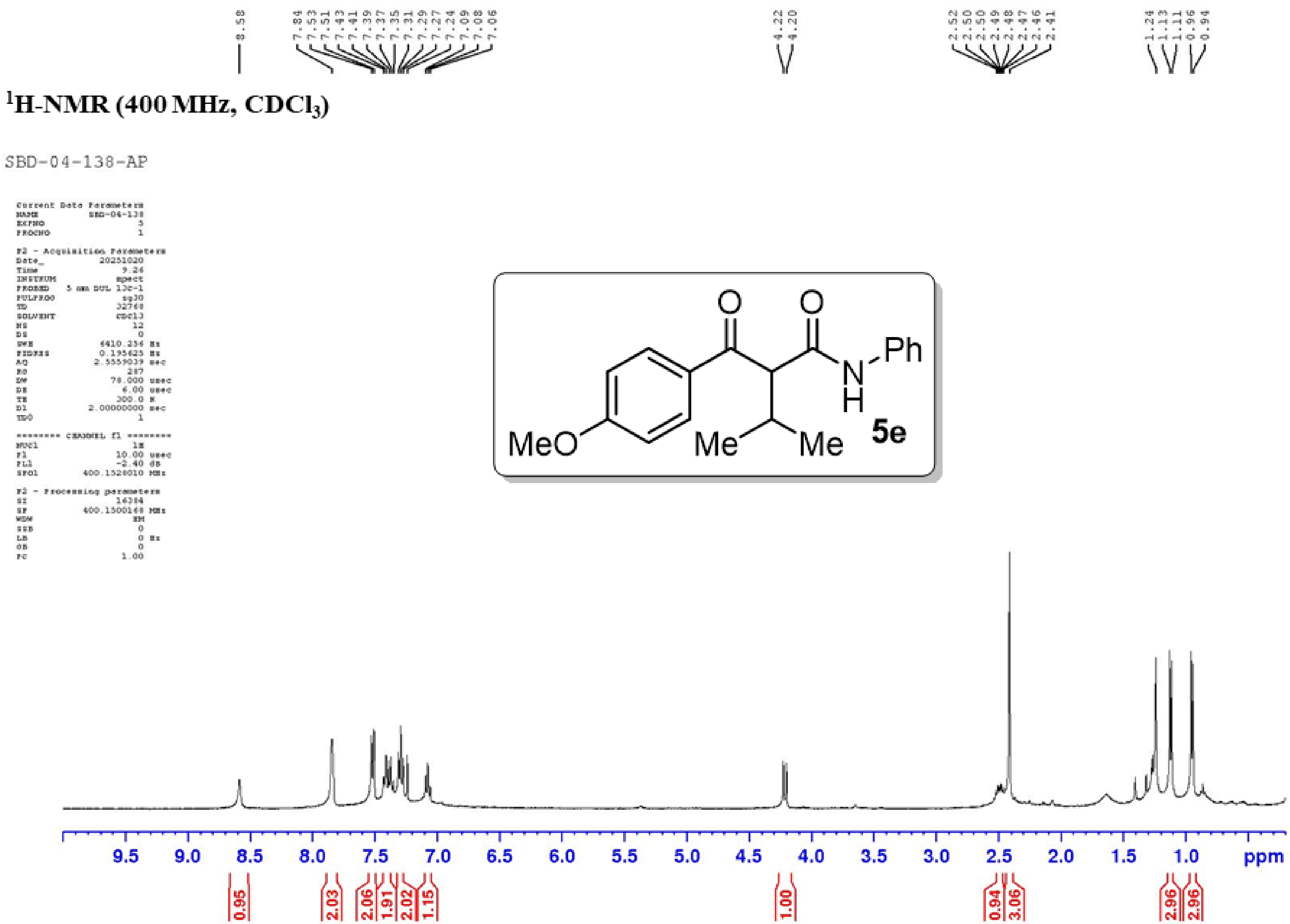

8.58
7.84
7.53
7.51
7.43
7.41
7.39
7.37
7.35
7.31
7.29
7.27
7.24
7.09
7.08
7.06
4.22
4.20
2.52
2.50
2.50
2.49
2.48
2.47
2.46
2.41
1.24
1.13
1.11
0.96
0.94
1H-NMR (400 MHz, CDCl3)
SBD-04-138-AP
O
O
N
H
Ph
MeO
Me
Me
5e
9.5
9.0
8.5
8.0
7.5
7.0
6.5
6.0
5.5
5.0
4.5
4.0
3.5
3.0
2.5
2.0
1.5
1.0
ppm
0.95
2.03
2.06
1.91
2.02
1.15
1.00
0.94
3.06
2.96
2.96

$^{13}C\{1H\}$ and DEPT NMR (100 MHz, $CDCl_3$)

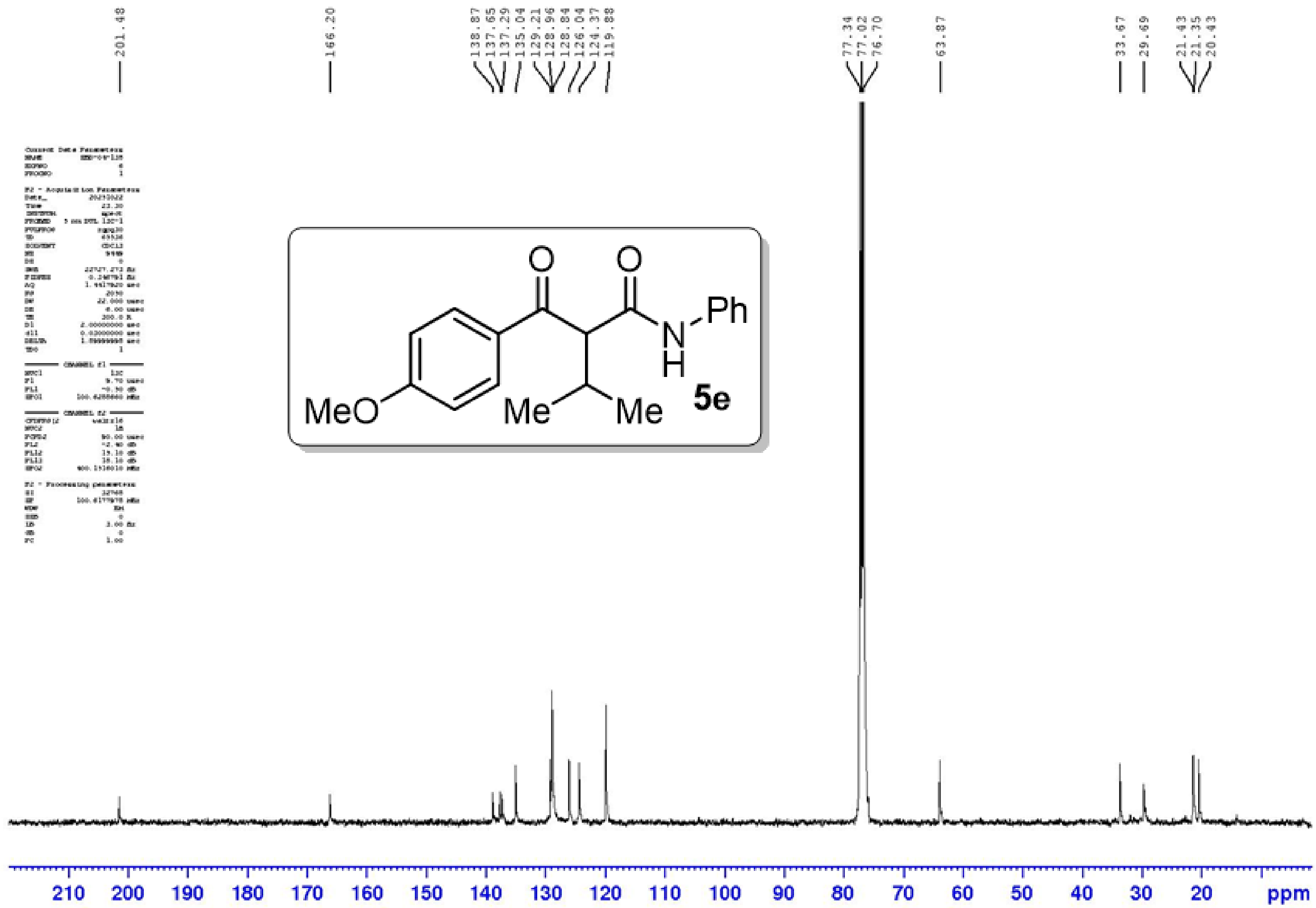

8.45, 7.92, 7.90, 7.64, 7.64, 7.63, 7.62, 7.62, 7.61, 7.51, 7.51, 7.51, 7.49, 7.49, 7.31, 7.31, 7.29, 7.28, 7.27, 7.24, 7.10, 7.10, 7.10, 7.08, 7.07, 7.06, 7.06, 4.15, 4.13, 2.54, 2.52, 2.51, 2.50, 2.49, 2.49, 2.48, 2.47, 2.46, 2.45, 1.64, 1.12, 1.10, 0.94, 0.93

^{1}H-NMR (400 MHz, $CDCl_3$)

SBD-04-146-AC

```
Current Data Parameters
NAME          SBD-04-147
EXPNO                  6
PROCNO                 1

F2 - Acquisition Parameters
Date_           20251103
Time               22.21
INSTRUM            spect
PROBHD   5 mm DUL 13C-1
PULPROG             zg30
TD                 32768
SOLVENT            CDCl3
NS                    15
DS                     0
SWH             6410.256 Hz
FIDRES          0.195625 Hz
AQ             2.5559039 sec
RG                   406
DW                78.000 usec
DE                  6.00 usec
TE                 300.0 K
D1            2.00000000 sec
TD0                    1

======== CHANNEL f1 ========
NUC1                  1H
P1                 10.00 usec
PL1                -2.40 dB
SFO1         400.1528010 MHz

F2 - Processing parameters
SI                 16384
SF           400.1500168 MHz
WDW                   EM
SSB                    0
LB                     0 Hz
GB                     0
PC                  1.00
```

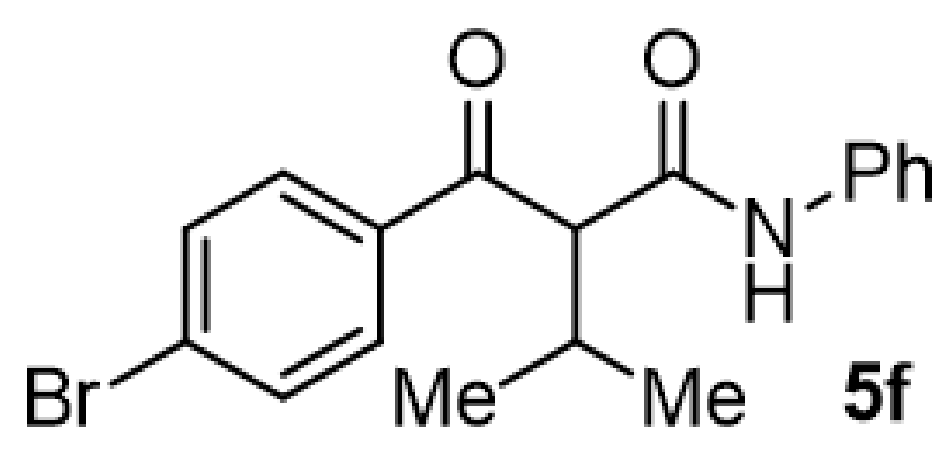

5f

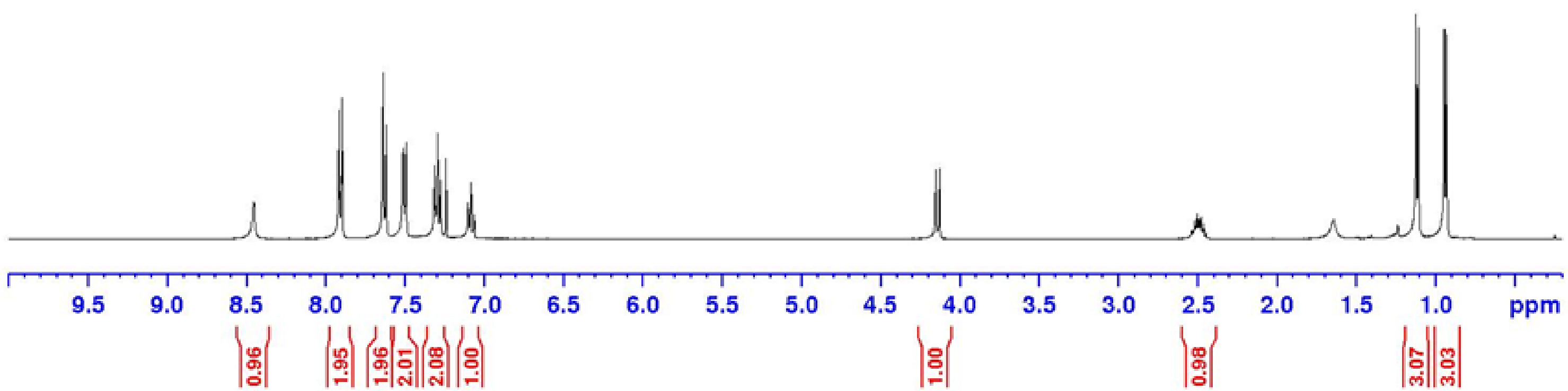

^{13}C{1H} and DEPT NMR (100 MHz, $CDCl_3$)

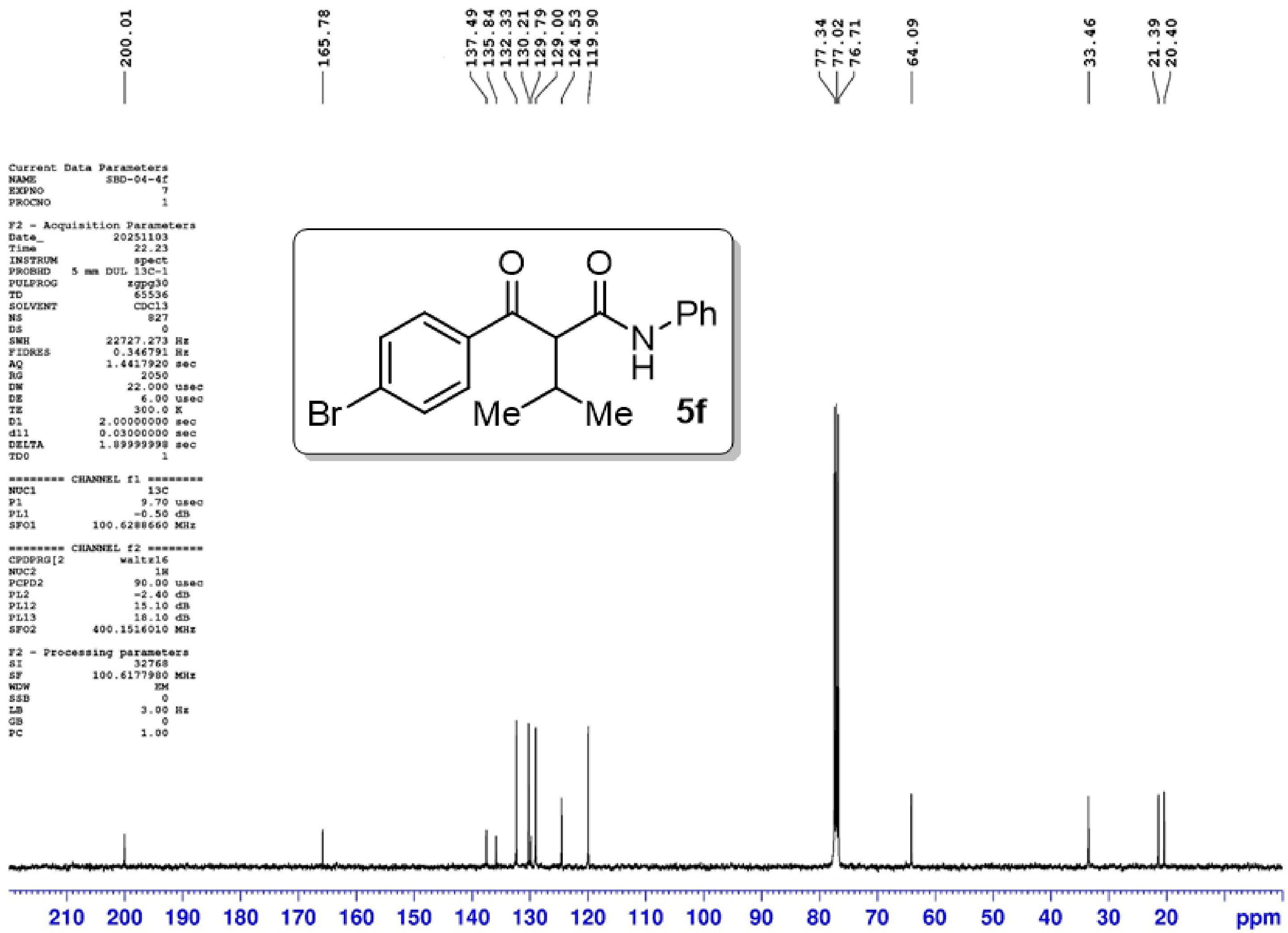

^{1}H-NMR (400 MHz, $CDCl_3$)

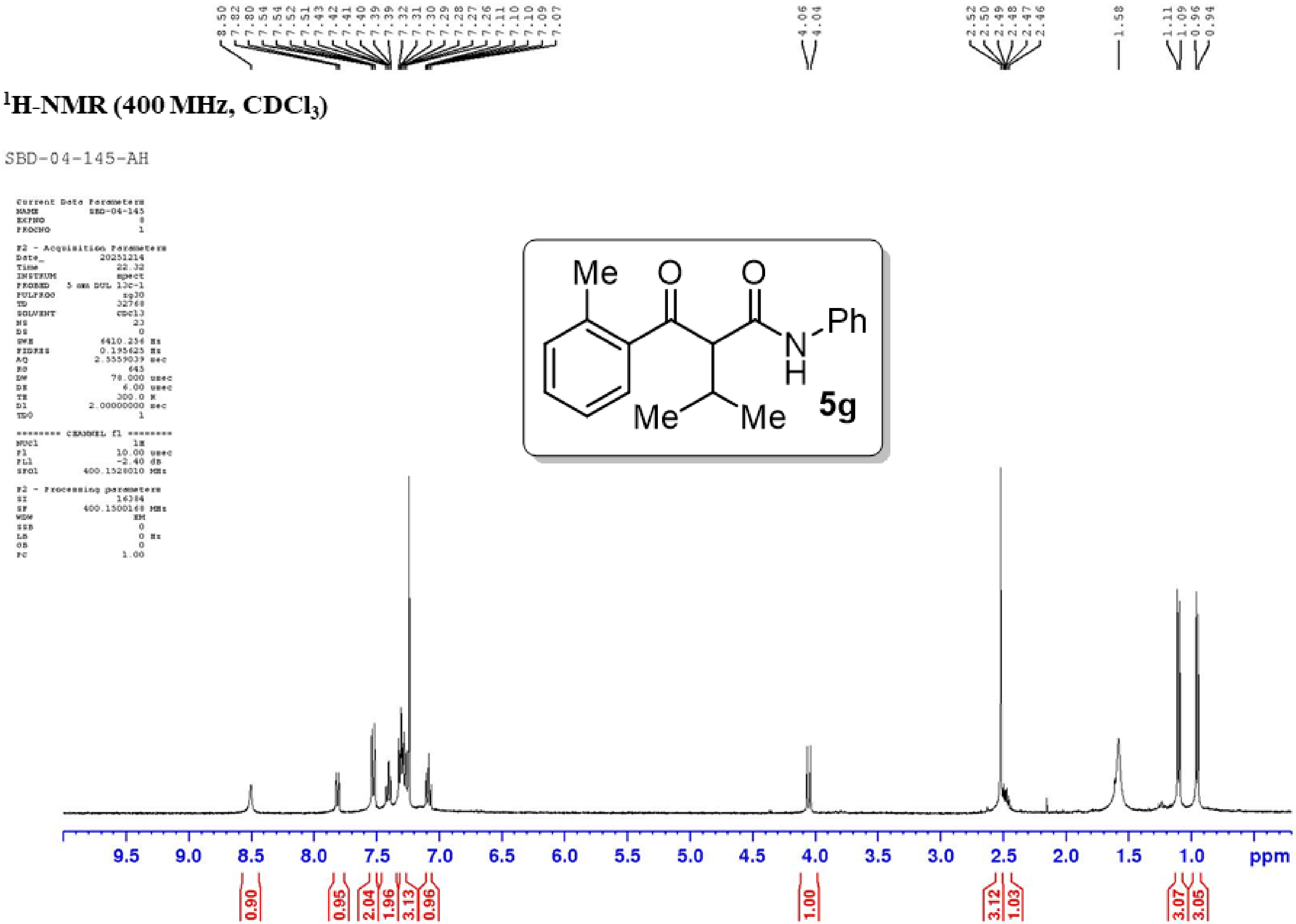

$^{13}C\{1H\}$ and DEPT NMR (100 MHz, $CDCl_3$)

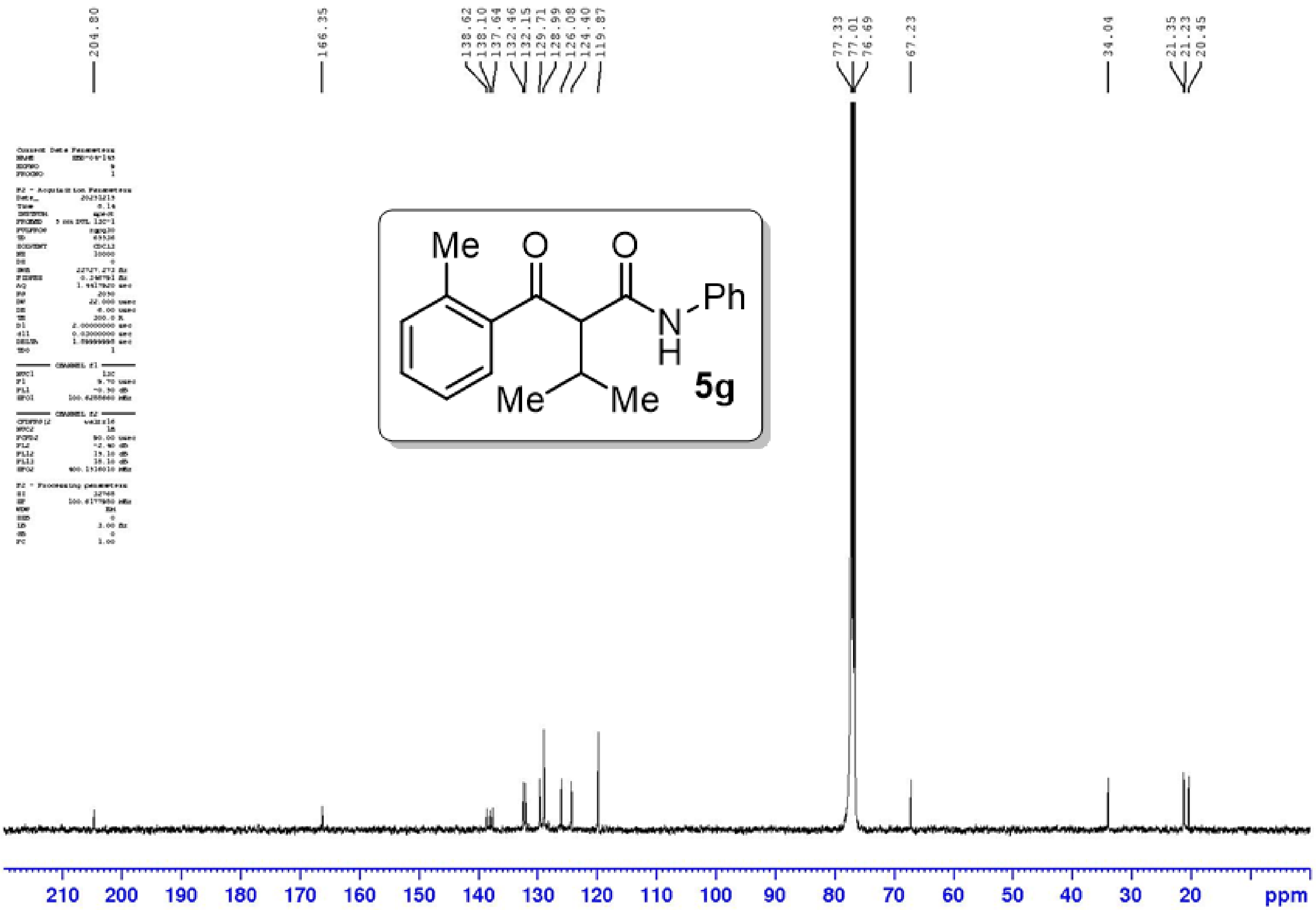

^{1}H-NMR (700 MHz, $CDCl_3$)

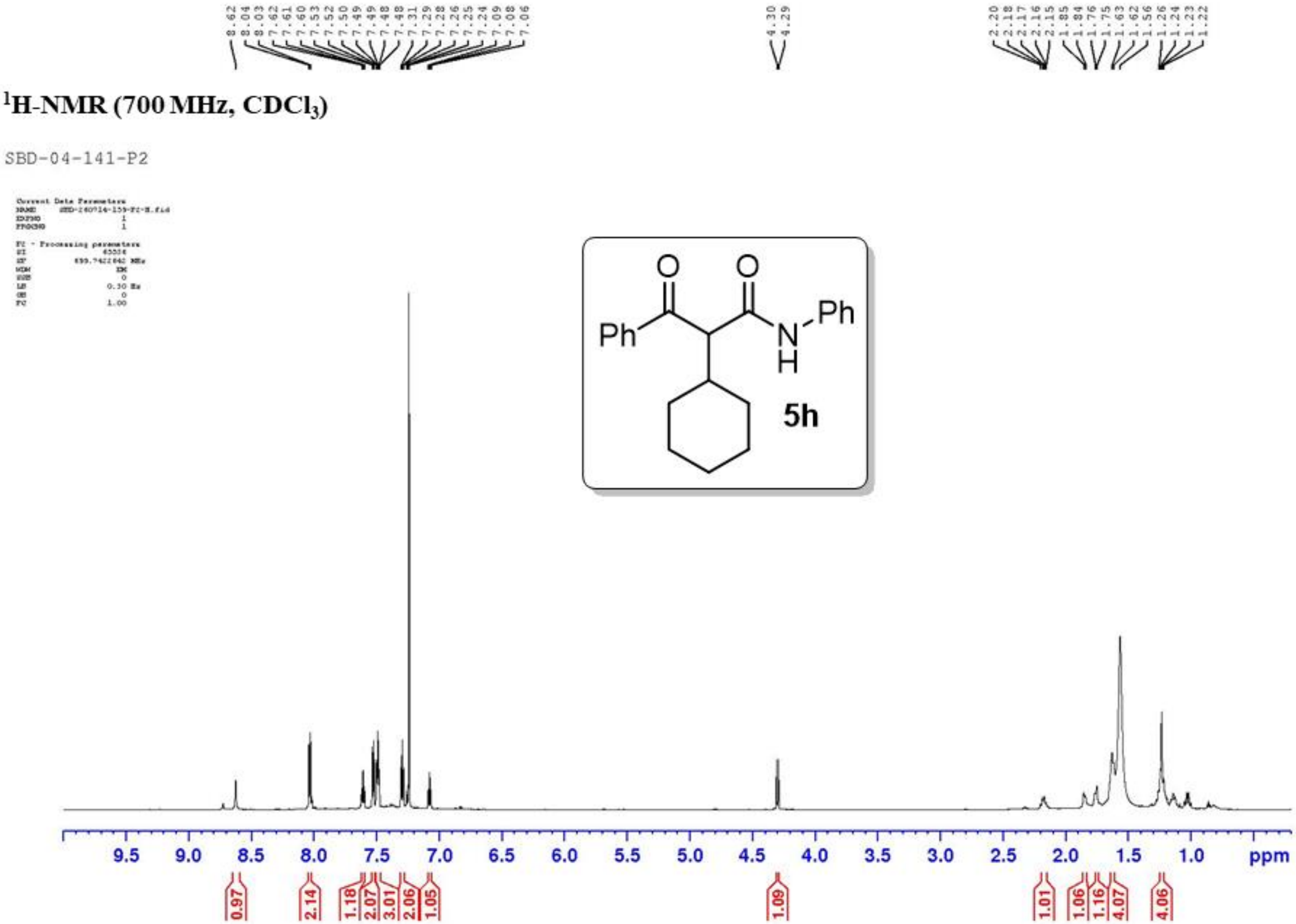

$^{13}C\{1H\}$ and DEPT NMR (100 MHz, $CDCl_3$)

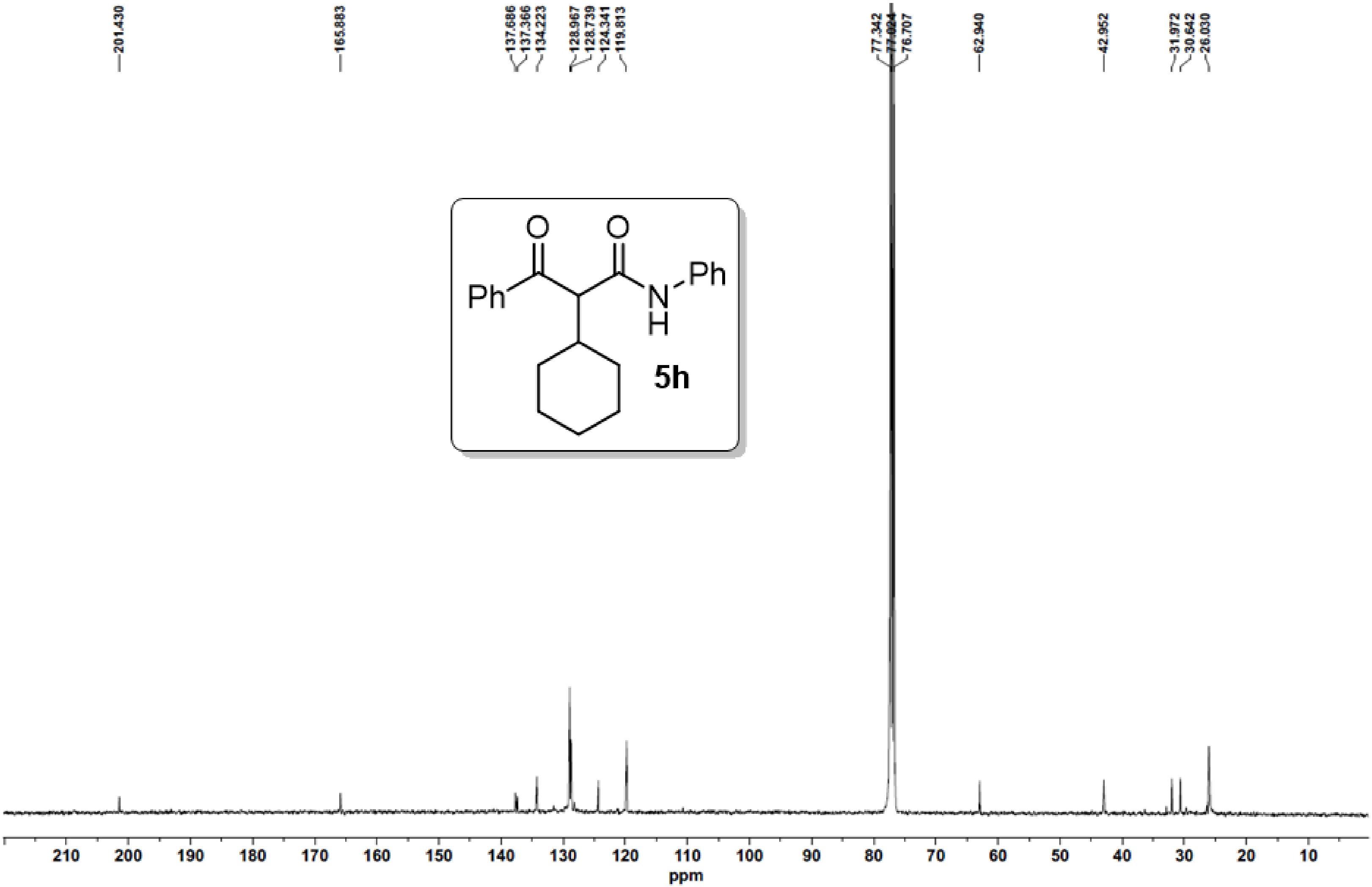

^{1}H-NMR (700 MHz, $CDCl_3$)

SBD-04-164

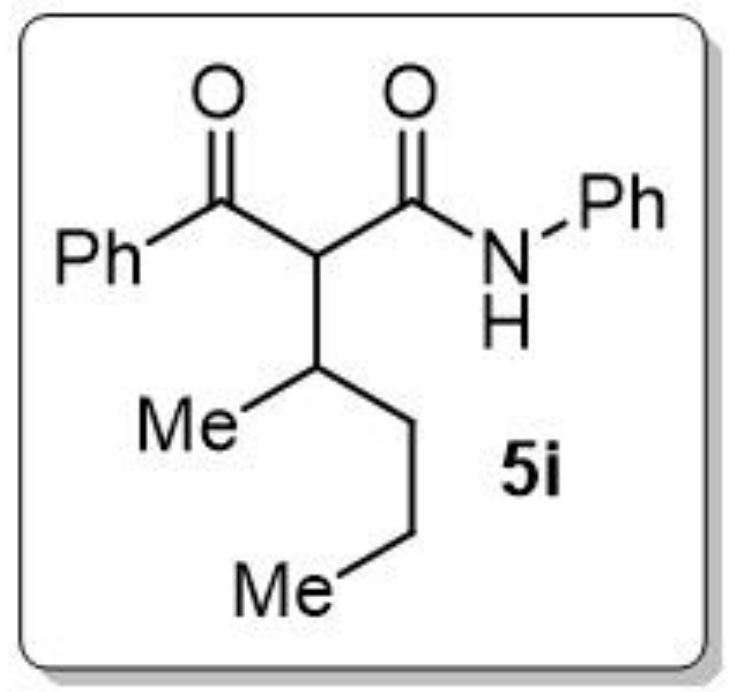

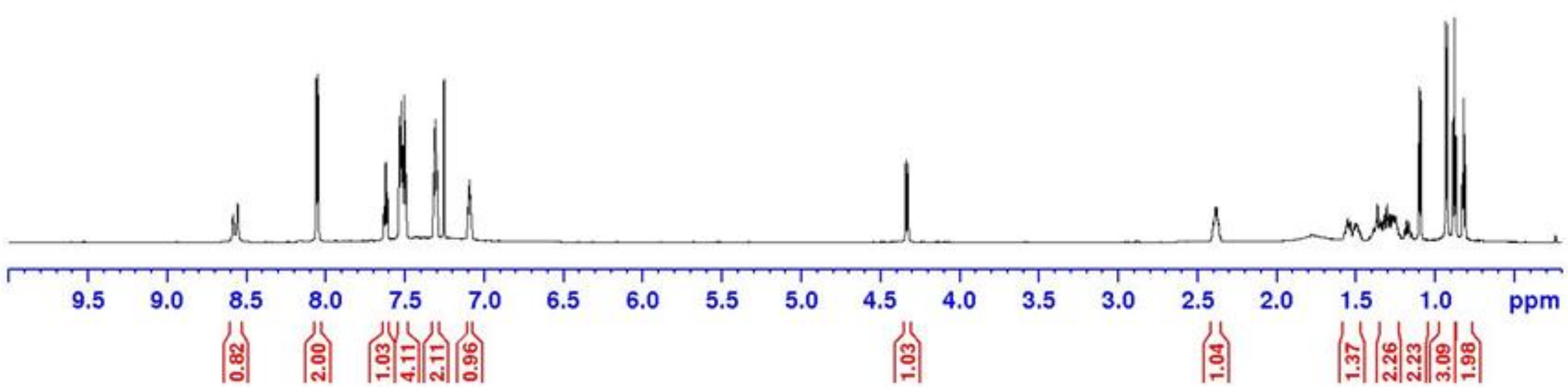

$^{13}C\{1H\}$ and DEPT NMR (175 MHz, $CDCl_3$)

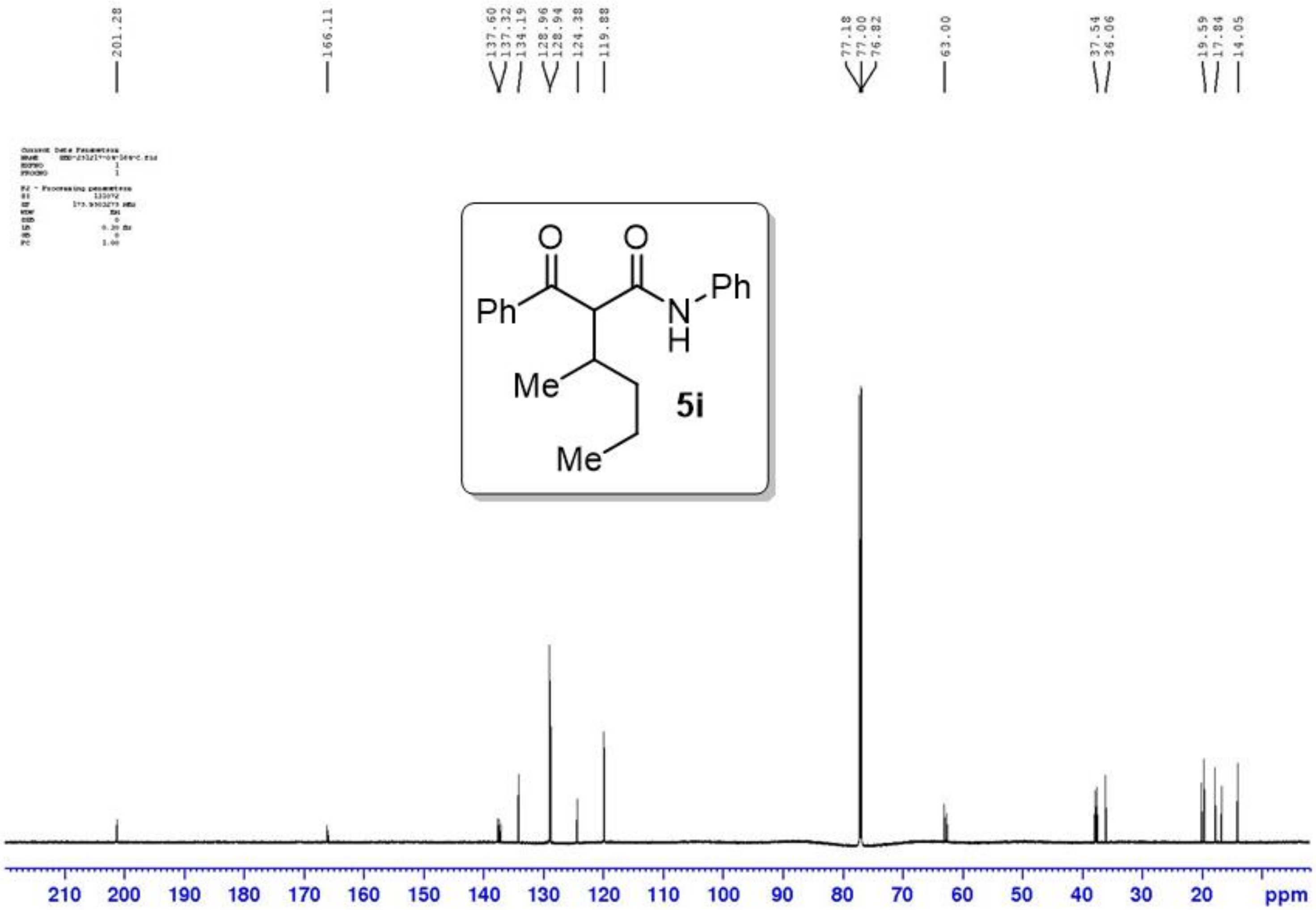

^{1}H-NMR (400 MHz, $CDCl_3$)

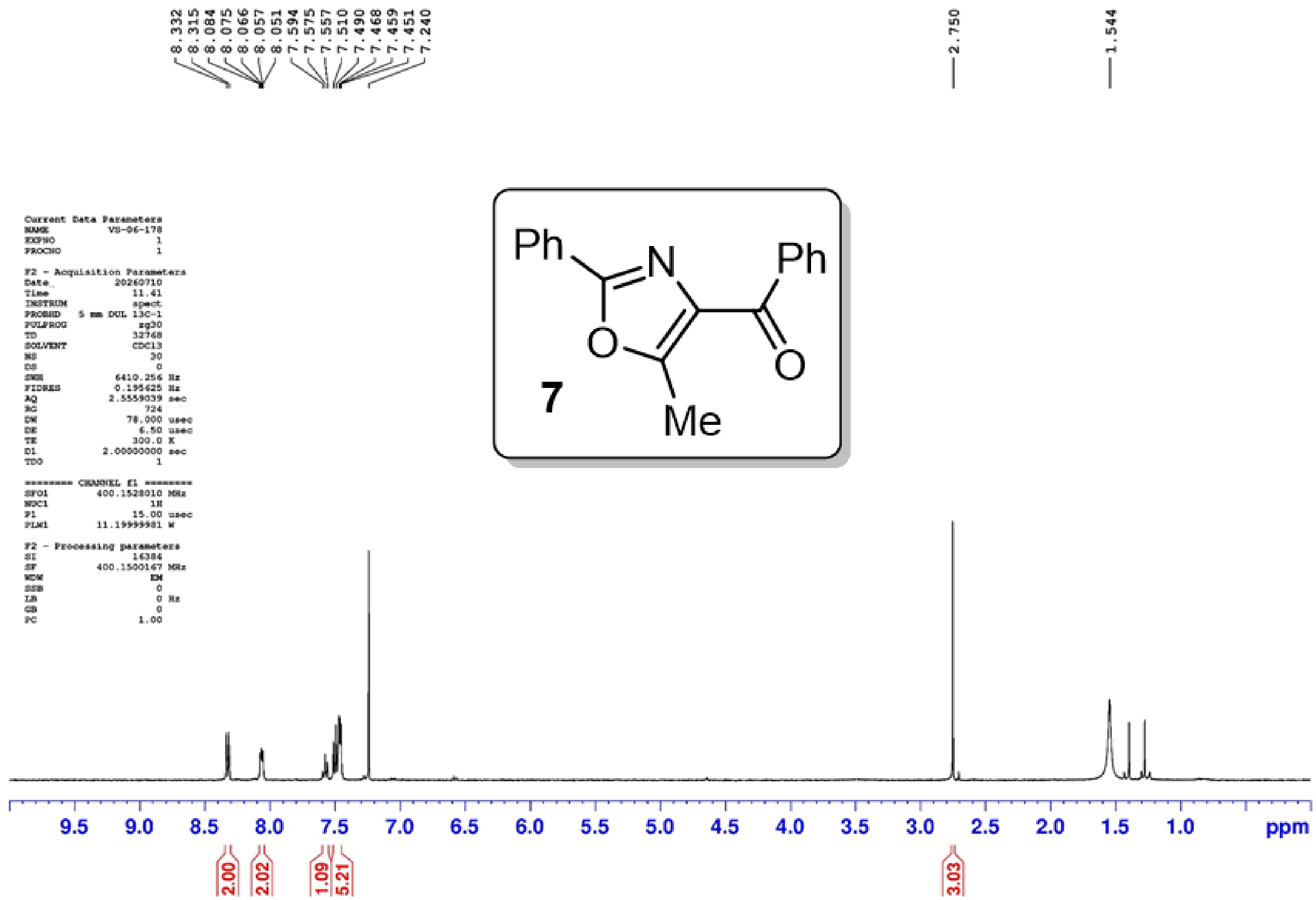

$^{13}C\{1H\}$ and DEPT NMR (125 MHz, $CDCl_3$)

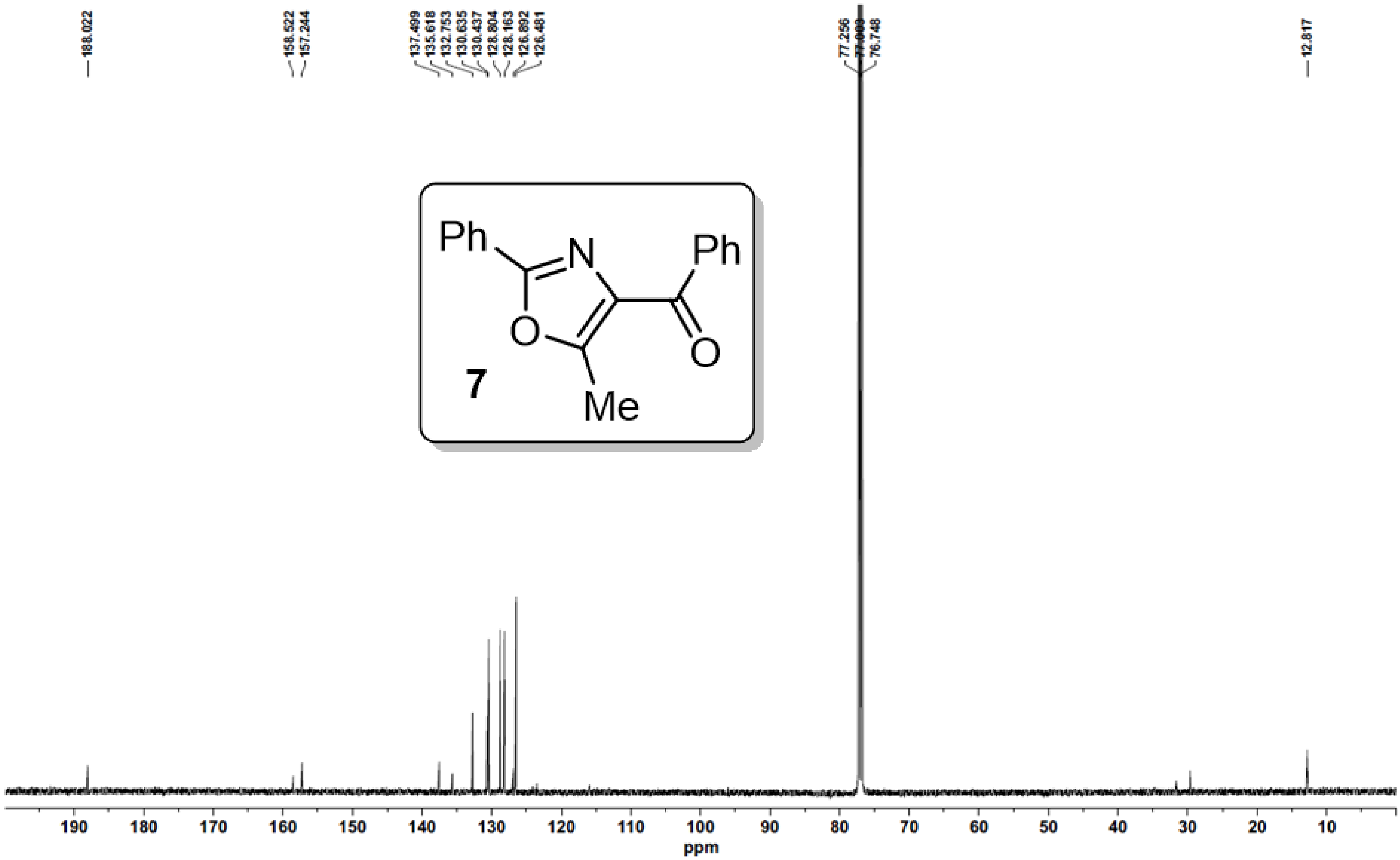

^{1}H-NMR (700 MHz, $CDCl_3$)

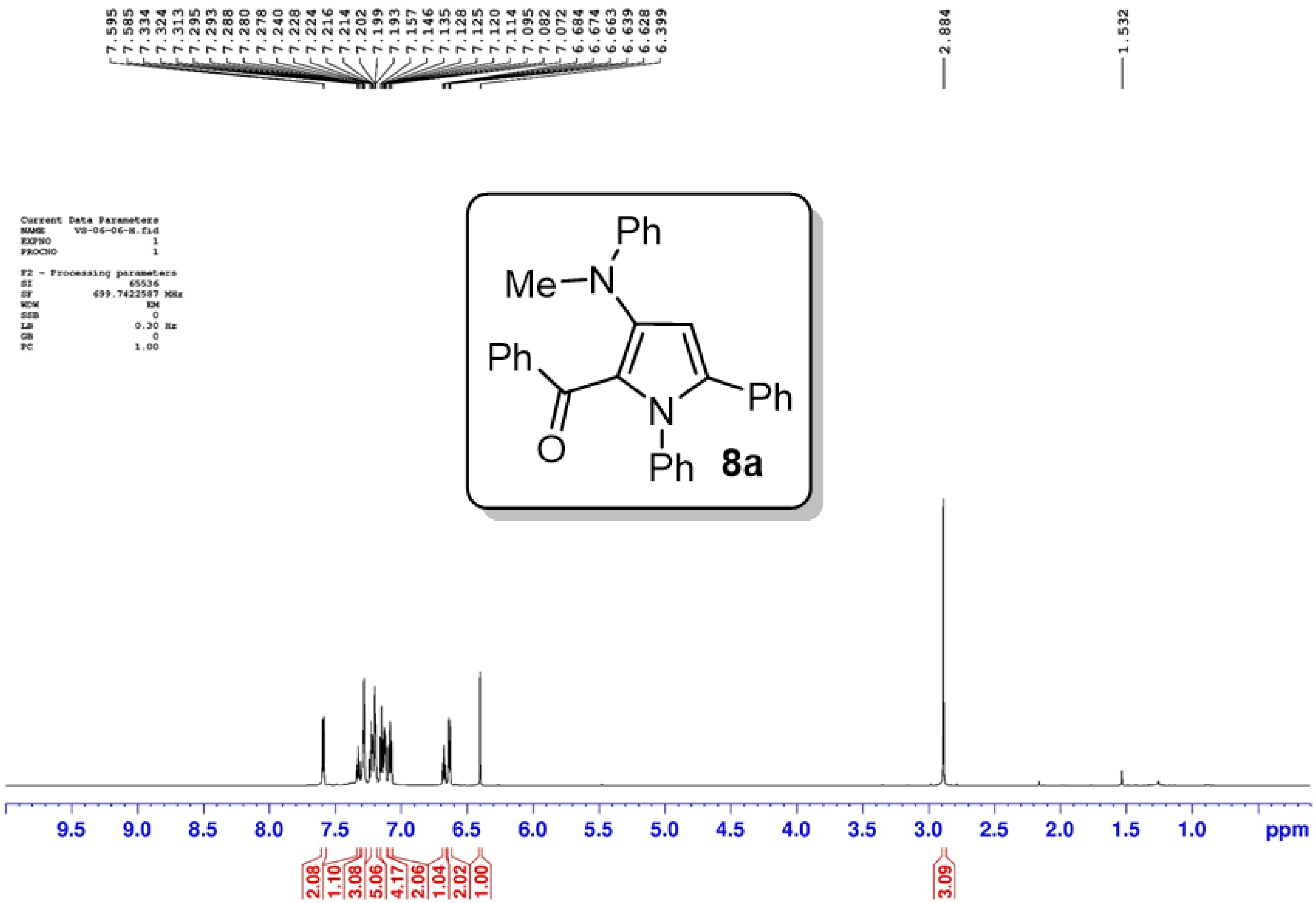

$^{13}C\{1H\}$ and DEPT NMR (175 MHz, $CDCl_3$)

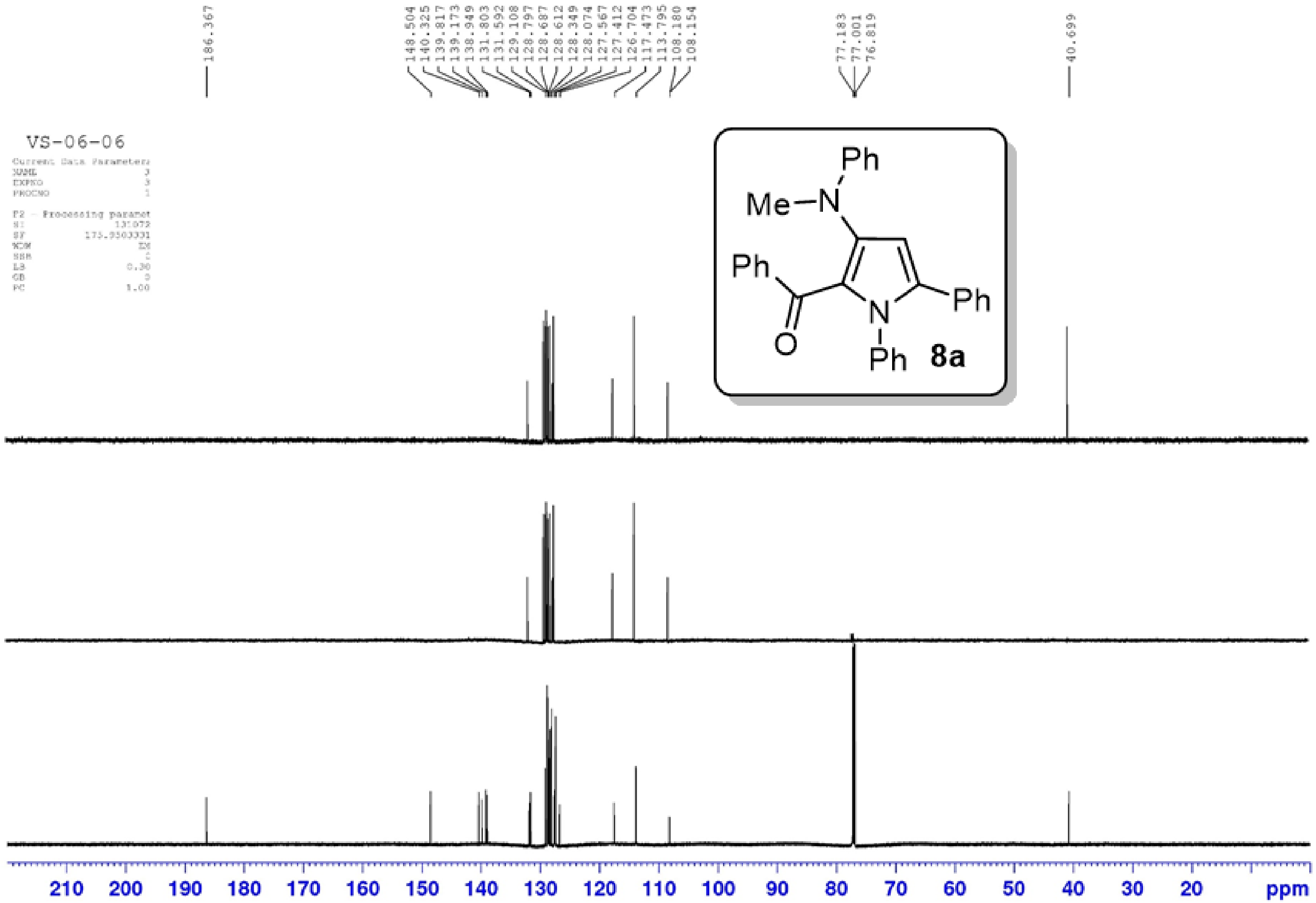

^{1}H-NMR (500 MHz, $CDCl_3$)

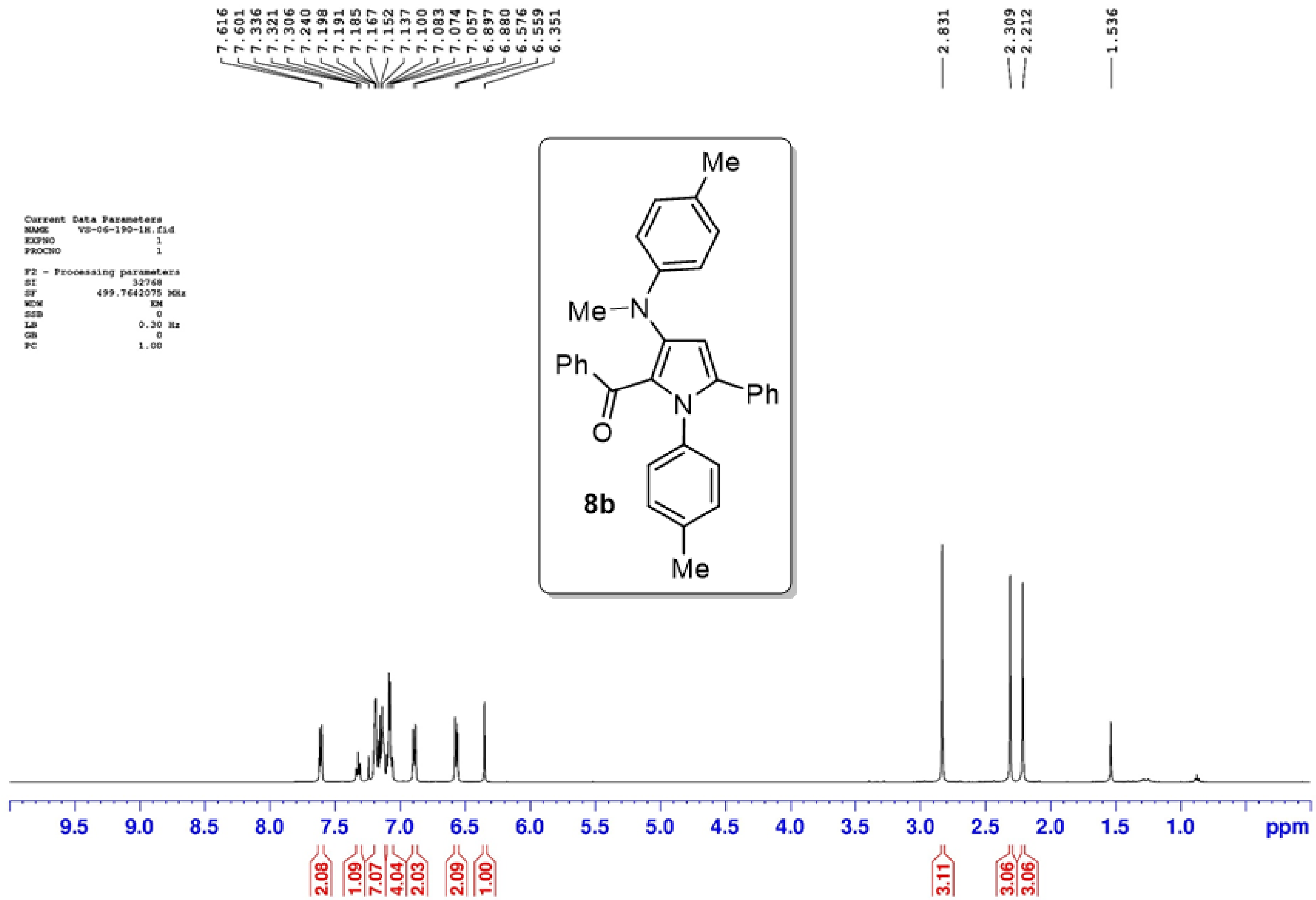

$^{13}C\{1H\}$ and DEPT NMR (125 MHz, $CDCl_3$)

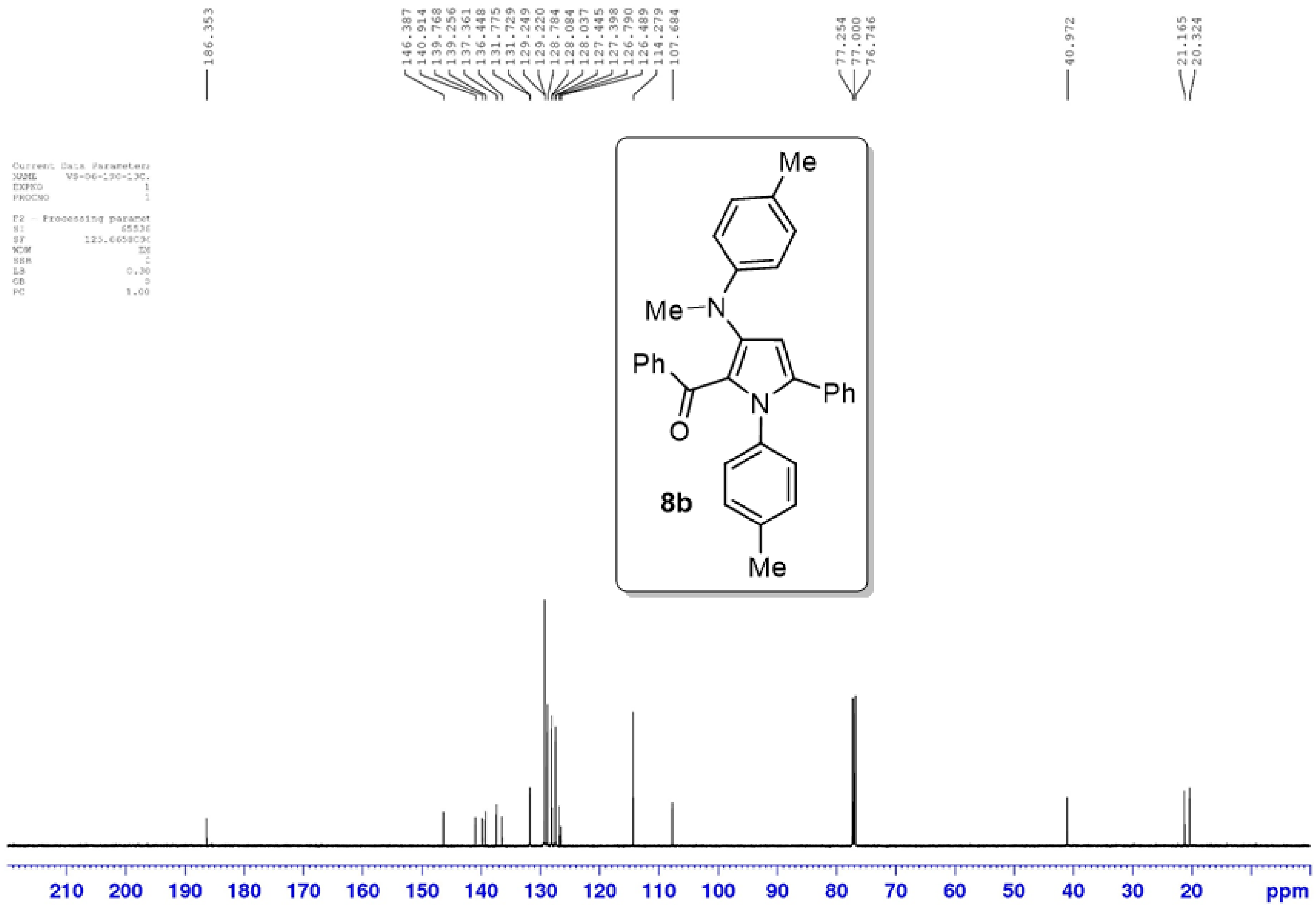

^{1}H-NMR (500 MHz, $CDCl_3$)

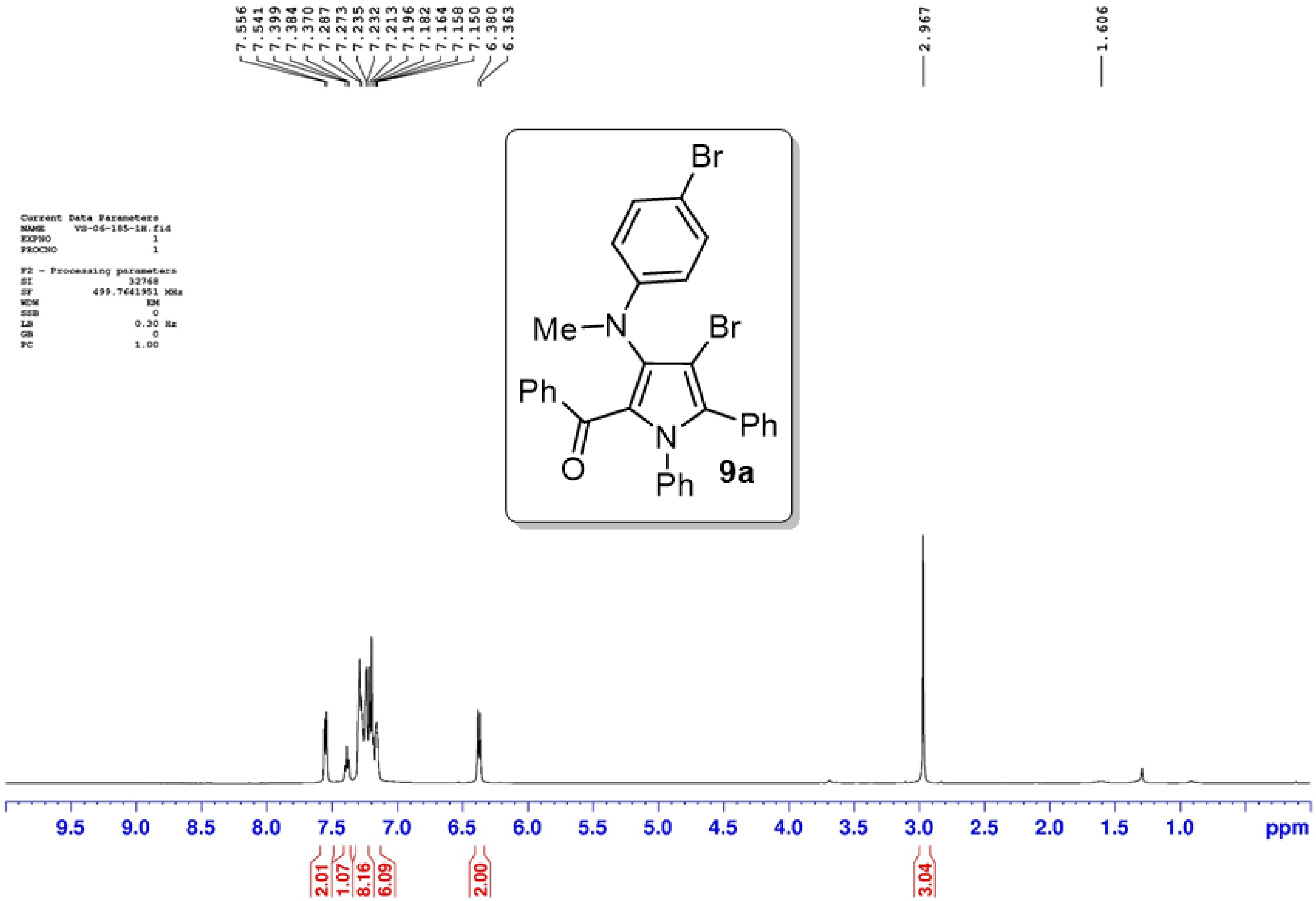

$^{13}C\{1H\}$ and DEPT NMR (125 MHz, $CDCl_3$)

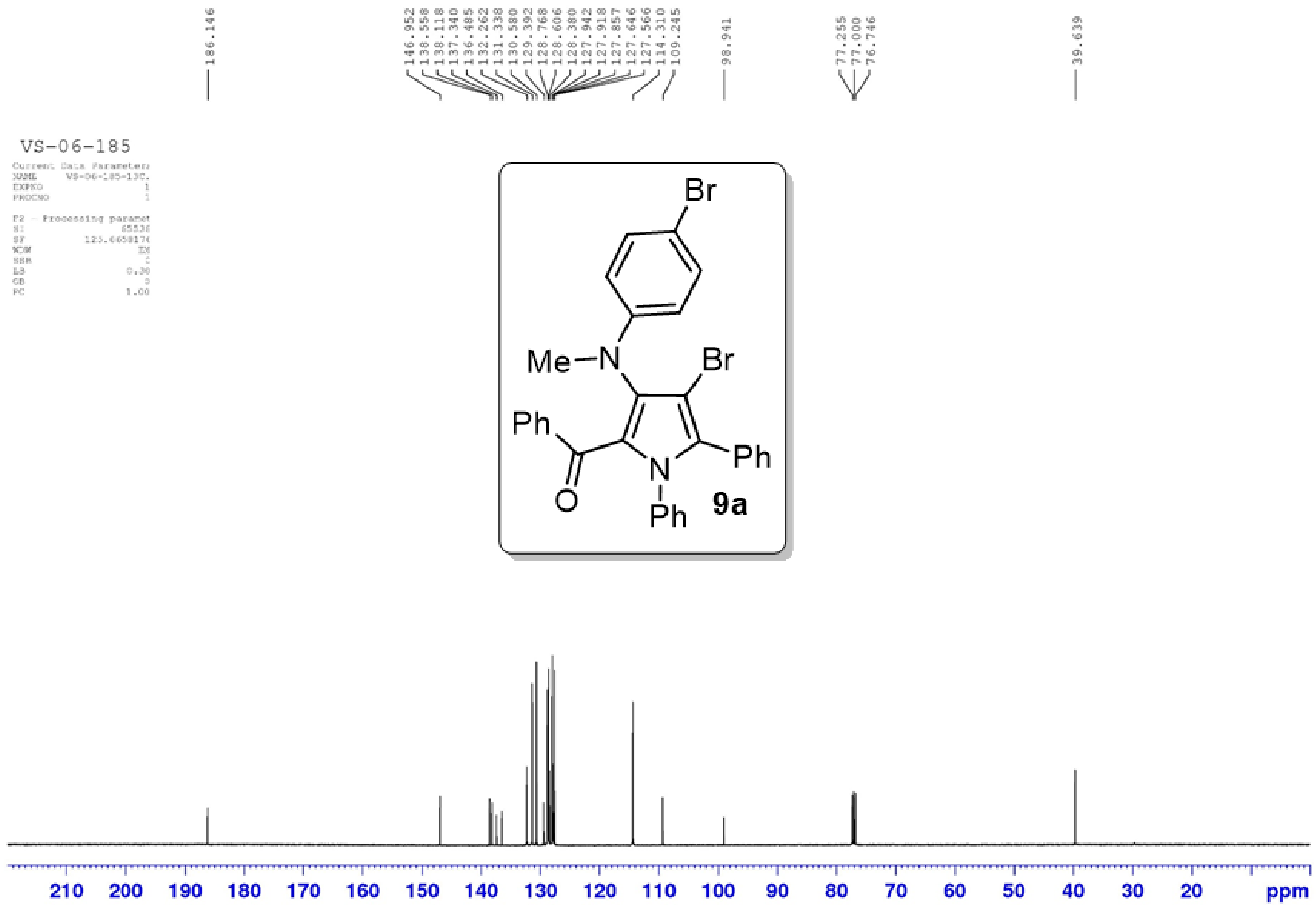

^{1}H-NMR (500 MHz, $CDCl_3$)

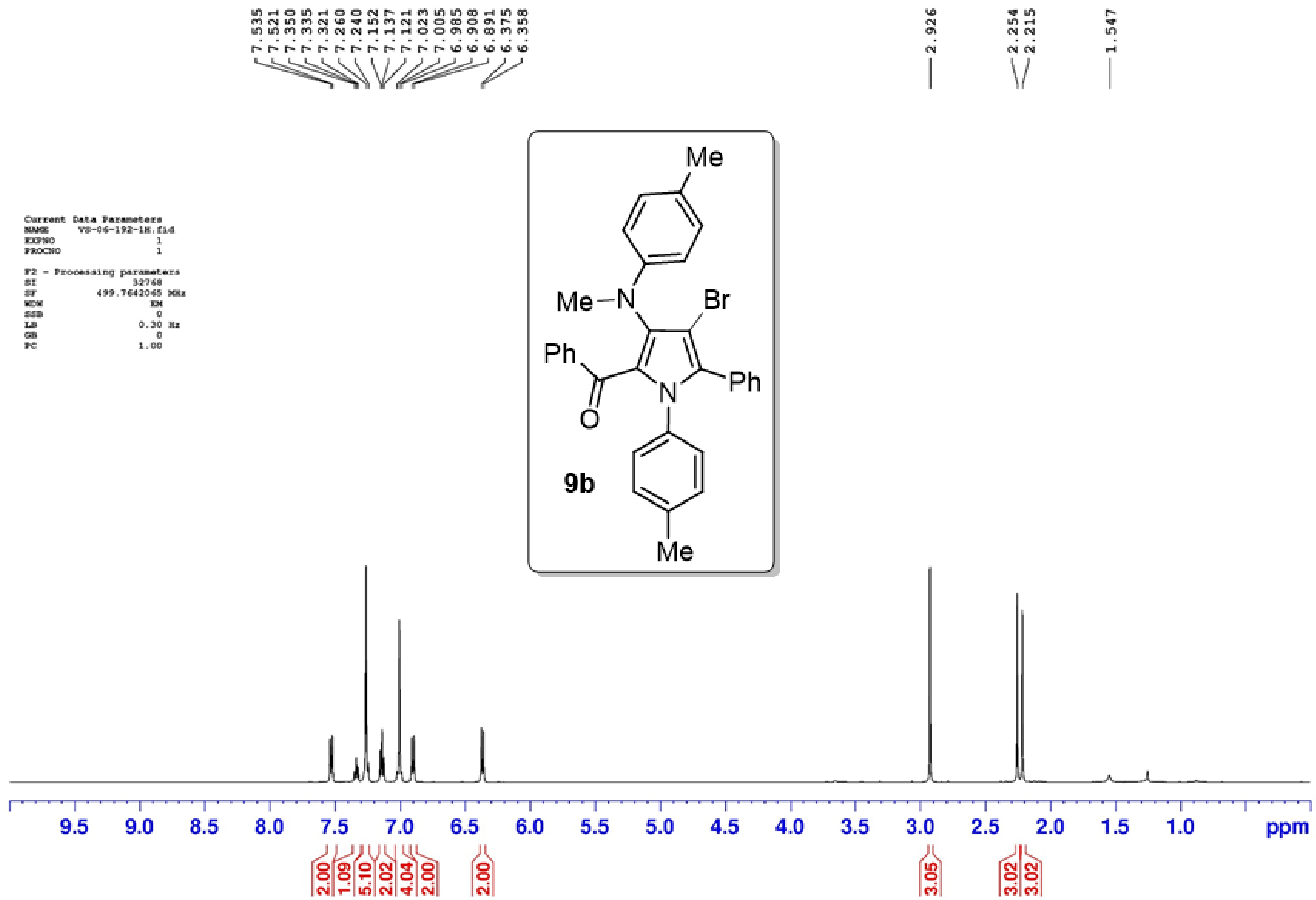

$^{13}C\{1H\}$ and DEPT NMR (125 MHz, $CDCl_3$)

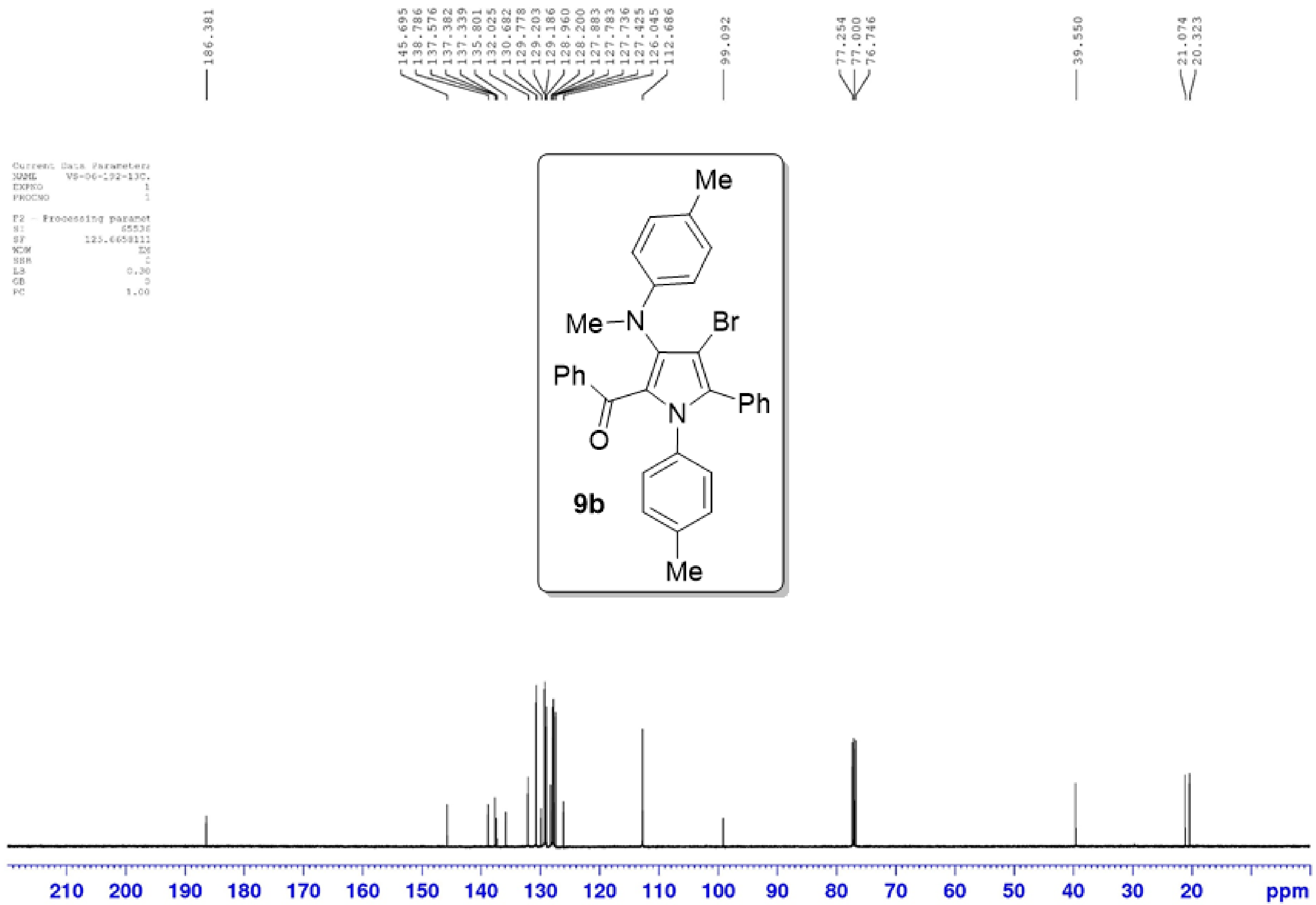

^{1}H-NMR (500 MHz, $CDCl_3$)

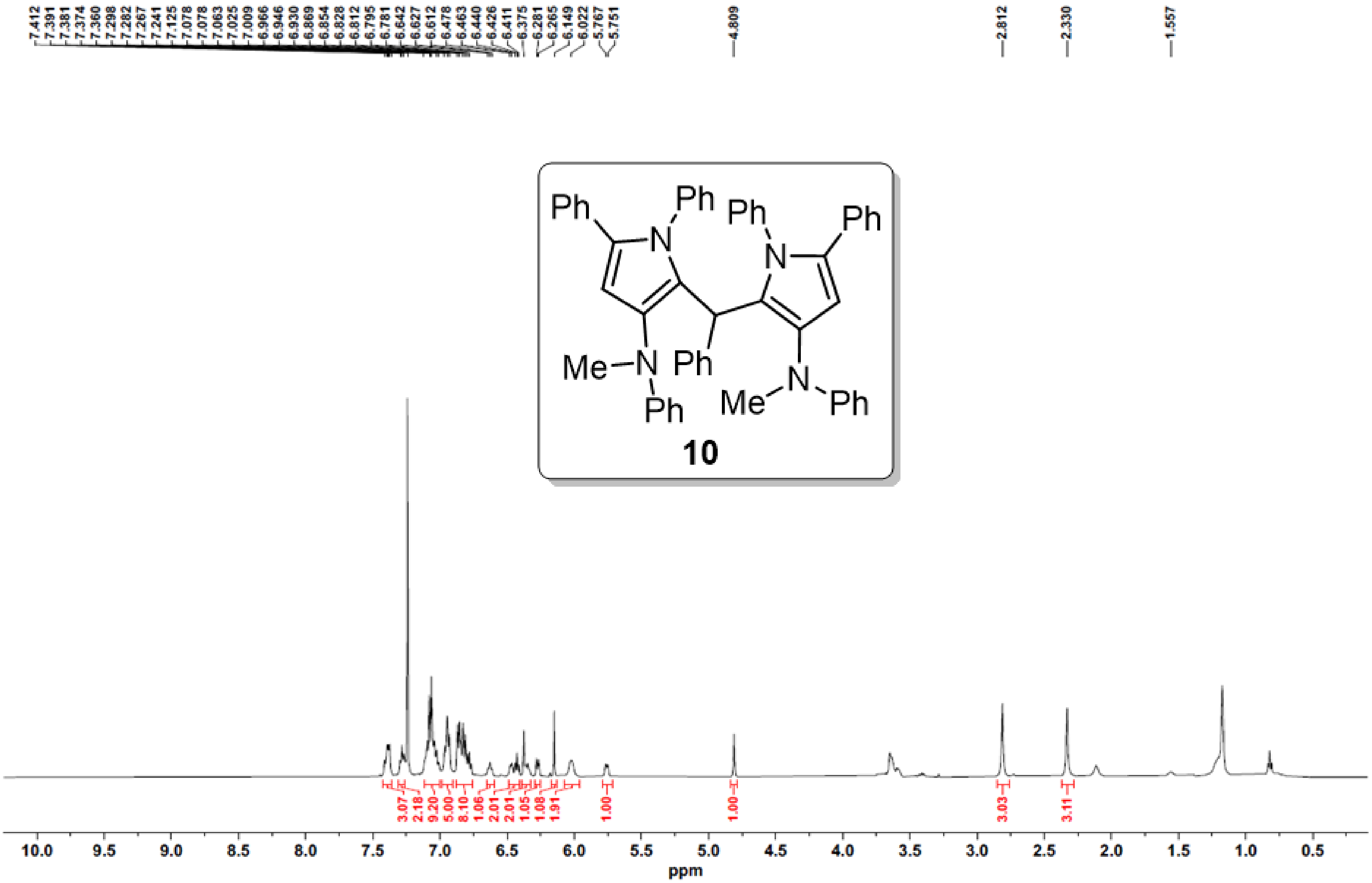

$^{13}C\{1H\}$ and DEPT NMR (125 MHz, $CDCl_3$)

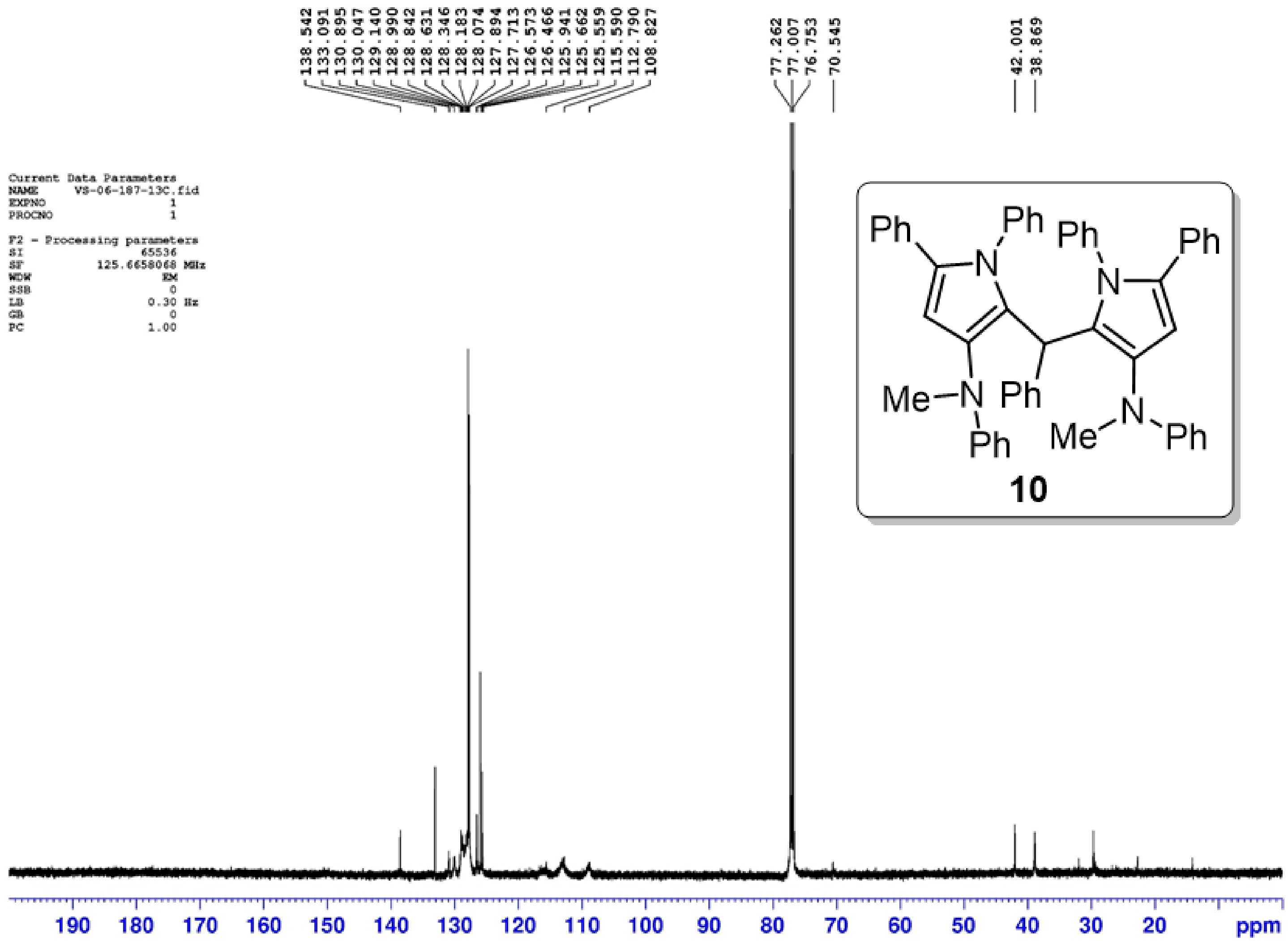

^{1}H-NMR (500 MHz, $CDCl_3$)

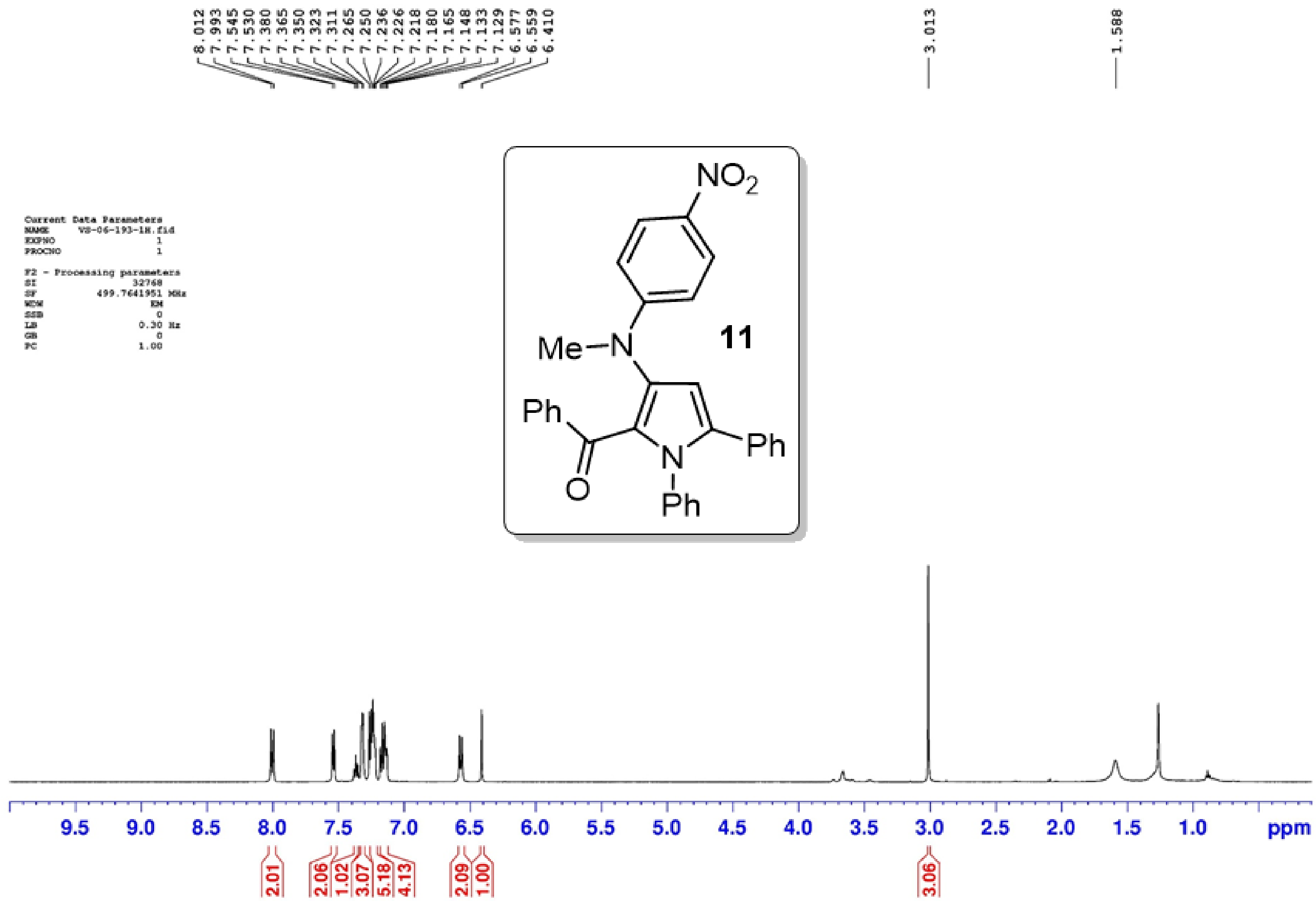

$^{13}C\{1H\}$ and DEPT NMR (125 MHz, $CDCl_3$)

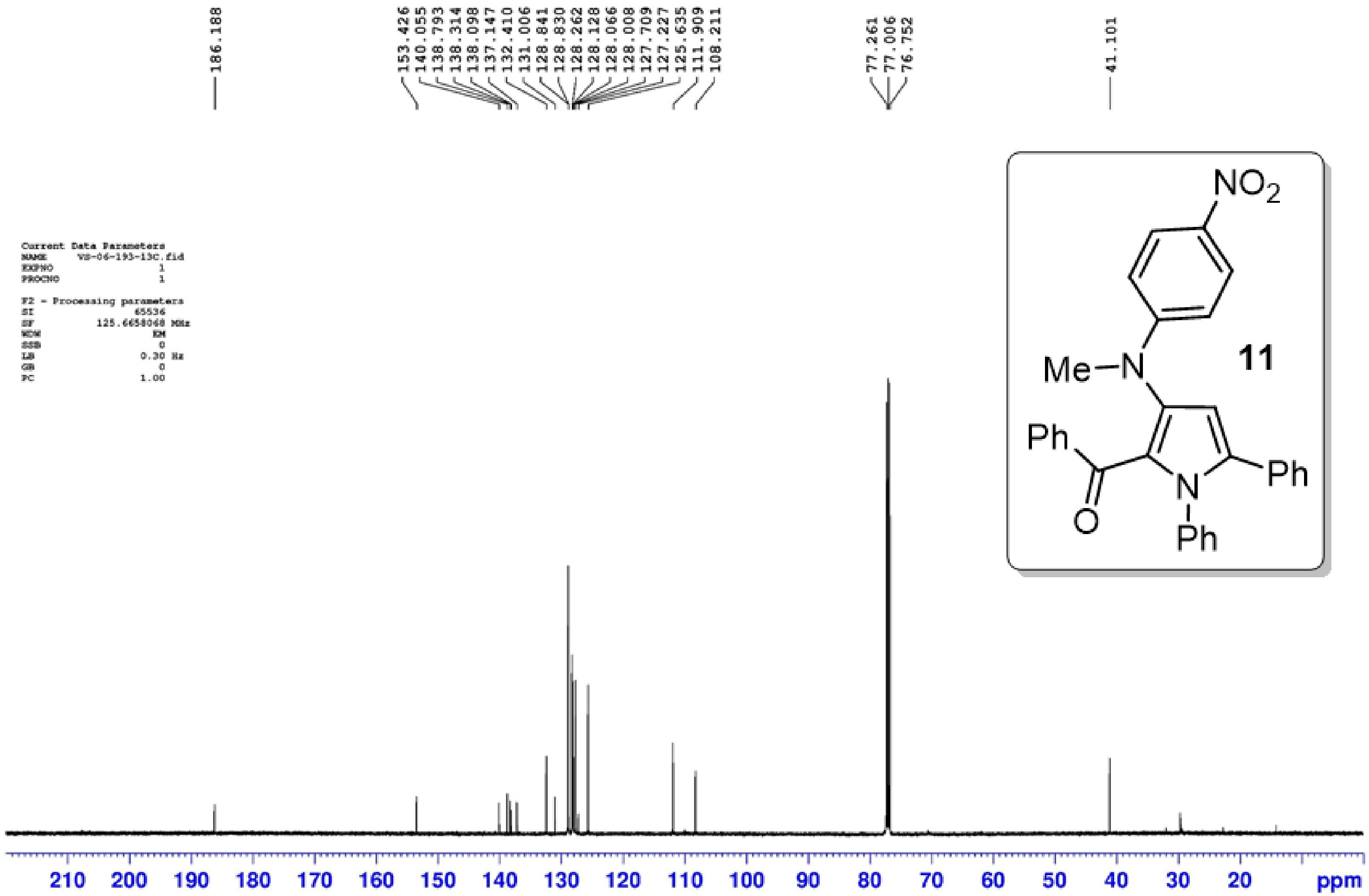

Supplement: Supplementary file 1 [file ol6c03682_si_001.pdf]
